# AI-Assisted Tools for Scientific Review Writing: Opportunities and Cautions

**DOI:** 10.1021/acsami.5c08837

**Published:** 2025-08-13

**Authors:** Julio C. M. C. Silva, Rafael P. Gouveia, Kallil M. C. Zielinski, Maria Cristina F. Oliveira, Diego R. Amancio, Odemir M. Bruno, Osvaldo N. Oliveira

**Affiliations:** † 28133 Sao Carlos Institute of Physics, University of São Paulo, 13560-970 São Carlos, SP, Brazil; ‡ Institute of Mathematics and Computer Sciences, University of São Paulo, 13560-970 São Carlos, SP, Brazil

**Keywords:** AI, large language models, machine written, scientific review writing

## Abstract

The evolution of large language models (LLMs) is reshaping the landscape of scientific writing, enabling the generation of machine-written review papers with minimal human intervention. This paper presents a pipeline for the automated production of scientific survey articles using Retrieval-Augmented Generation (RAG) and modular LLM agents. The pipeline processes user-selected literature or citation network-derived corpora through vectorized content, reference, and figure databases to generate structured, citation-rich reviews. Two distinct strategies are evaluated: one based on manually curated literature and the other on papers selected through citation network analysis. Results demonstrate that increasing the input materials’ diversity and quantity improves the generated output’s depth and coherence. Although current iterations produce promising drafts, they fail to meet top-tier publication standards, particularly in critical analysis and originality. Results were obtained for a case study on a particular topic, namely, Langmuir and Langmuir–Blodgett films, but the proposed pipeline applies to any user-selected topic. The paper concludes with suggestions of how the system could be enhanced through specialized modules and discusses broader implications for scientific publishing, including ethical considerations, authorship attribution, and the risk of review proliferation. This work represents an opportunity to discuss the advantages and pitfalls introduced by the possibility of using AI assistants to support scientific knowledge synthesis.

## Graphical abstract

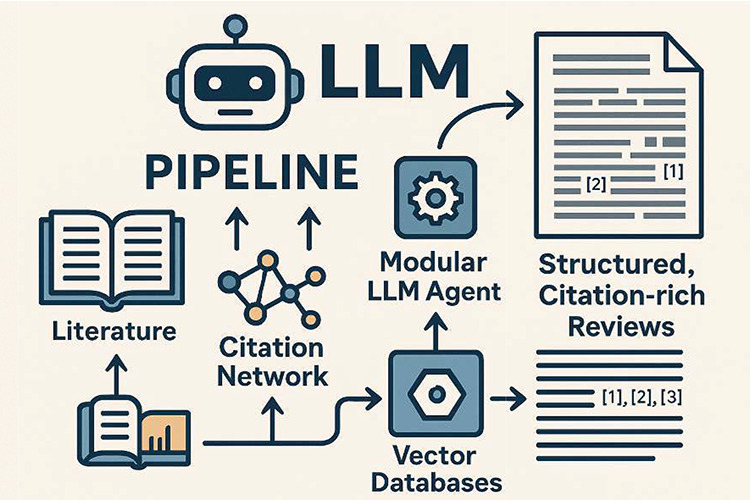

## 1 Introduction

Recent developments in natural language processing (NLP) with large language models^1–3^ can be seen as evidence that the fifth paradigm of knowledge generation—machine-generated knowledge^4,5^ is approaching. Under this paradigm, machines are expected to be capable of raising research hypotheses, devising and implementing research projects, and ultimately producing scientific papers reporting original results autonomously. Indeed, several proof-of-concept studies have demonstrated AI systems performing tasks resembling various stages of scientific research and production,^6,7^ including academic writing.^8,9^ For instance, researchers have utilized LLMs to generate scientific abstracts and introductory sections, even listing AI models as coauthors.^10^

Several attempts to prepare Systematic Literature Reviews (SLRs) have employed the Retrieval-Augmented Generation (RAG) technique, explained in detail in the Supporting Information. For example,^11^ introduces a fine-tuned domain-specific LLM framework that automates research synthesis by generating structured question-and-answer (Q & A) data sets from SLR corpora. LitLLM^12^ focuses on literature retrieval and summarization by extracting keywords from user-provided abstracts and employs a reranking mechanism to refine the relevance of retrieved papers. Designed for biomedical literature recommendations, RefAI^13^ uses a multivariate ranking system that incorporates multiple metrics, such as journal impact factors, citation counts, and embedding-based similarity measures, to improve literature selection and summarization. Han et al.^8^ proposed a framework integrating retrieval, augmentation, and generation across the entire SLR process. Wu et al. presented a fully automated review generation based on LLMs,^9^ capable of processing hundreds of research articles in seconds and generating comprehensive reviews across multiple topics. Similar systems to generate summaries or surveys of the literature include InteractiveSurvey,^14^ Ai2ScholarQA,^15^ SurveyX,^16^ and AutoSurvey.^17^

In line with the increasing usage of LLMs in preparing scientific articles,^18^ recent advancements indicate that at least one AI system has successfully generated original research work capable of passing peer review at an academic workshop associated with a prestigious conference.^19,20^ Nonetheless, there is much criticism about the quality of the outcomes regarding both content and presentation accuracy, e.g., factual or conceptual errors occur, as well as mistakes in handling references, figures, and other textual elements. We argue that these are not due to an inherent limitation of current NLP technology. Instead, autonomous knowledge generation—reporting novel ideas, analyses, and/or results in a scientific paper—remains unproven due to a lack of targeted investments in this specific capability. An apt analogy is that of capable individuals who require training to become competent scientists. From this perspective, there is room for developing AI systems designed specifically for academic knowledge generation, even within the current NLP technological framework.

Moreover, the formal publication and ethical acceptance of fully autonomous AI-generated research must be debated in light of ethical concerns. For example, issues to consider include credibility, accuracy, and comprehensiveness. Is it reasonable to delegate to an AI the task of studying the literature to compile existing knowledge? To what extent can we trust that its analysis is correct? A competent scientist can identify methodological flaws and other issues—can an AI be trusted to do the same? While these are questions without clear answers today, another, more pressing question arises: Is it possible to fully automate the process of generating a literature review on a given topic?

In this paper, we argue that it will soon be possible to generate survey (or review) papers automatically. We introduce a pipeline through which an AI agent could perform this task. The focus on this type of scientific paper is intended to avoid the additional challenge of assessing whether the output contains sufficient original content, as would be required for a standard research article. The chosen topic—Langmuir and Langmuir–Blodgett (LB) films—is one of the authors’ areas of expertise, facilitating decisions regarding input materials and evaluating generated texts. A discussion of the quality of different versions of the generated survey paper is followed by the prospects for further development and implications for scientific publishing. In this discussion, we use a rubric to evaluate the coverage and appropriateness of the sections and subsections in the generated survey papers, whose quality was assessed by multiple independent experts.

## 2 Pipeline for an AI Survey Writer

A human author may employ several strategies when preparing a survey (or review) paper. Naturally, these strategies can now be adapted to exploit the vast processing power of large language models (LLMs), which may also motivate entirely novel strategies. In this work, we automatically generate surveys based on two distinct approaches. The first aims to replicate human experts’ frequent strategy when writing surveys. Typically, they begin by studying multiple papers in the literature related to the target topic(s), including previous reviews, while incorporating their expertise and delimiting areas of interest. We simulate this process by selecting scientific papers from the literature, including review articles, as input for generating the survey. As for integrating prior expert knowledge, we argue that a similar mechanism exists in LLMs, which draw upon their training corpus plus additional information via embedding-based retrieval techniques. The second strategy involves identifying the topic landscape from the literature through network analysis and natural language processing (NLP).^21,22^ The resulting citation network structure is used to select papers as input to generate a survey, as described in Section 3.

Figure 1 depicts the complete AI-driven workflow, illustrating the pipeline from research subject selection to the final LaTeX output. The process begins with user-defined research topics and selected scientific papers, which are processed into three specialized RAG databases. The research topics and the scientific papers provide the foundation to generate a paper outline organized in sections, which is then enriched with the RAG databases by synthesizing content and integrating references and figures. Subsequently, the text is refined, and an abstract and title are generated. Finally, the system reviews LaTeX syntax to ensure the LLMs have not used a syntax not recognizable by a LaTeX compiler, such as Markdown, before outputting a complete.tex file accompanying a .bib reference file. We run the LLM agents on an in-house cluster with two RTX 4090 GPUs, a Ryzen 9 7900X CPU, 92GB of RAM, and 2TB of SSD.

**Figure 1:**
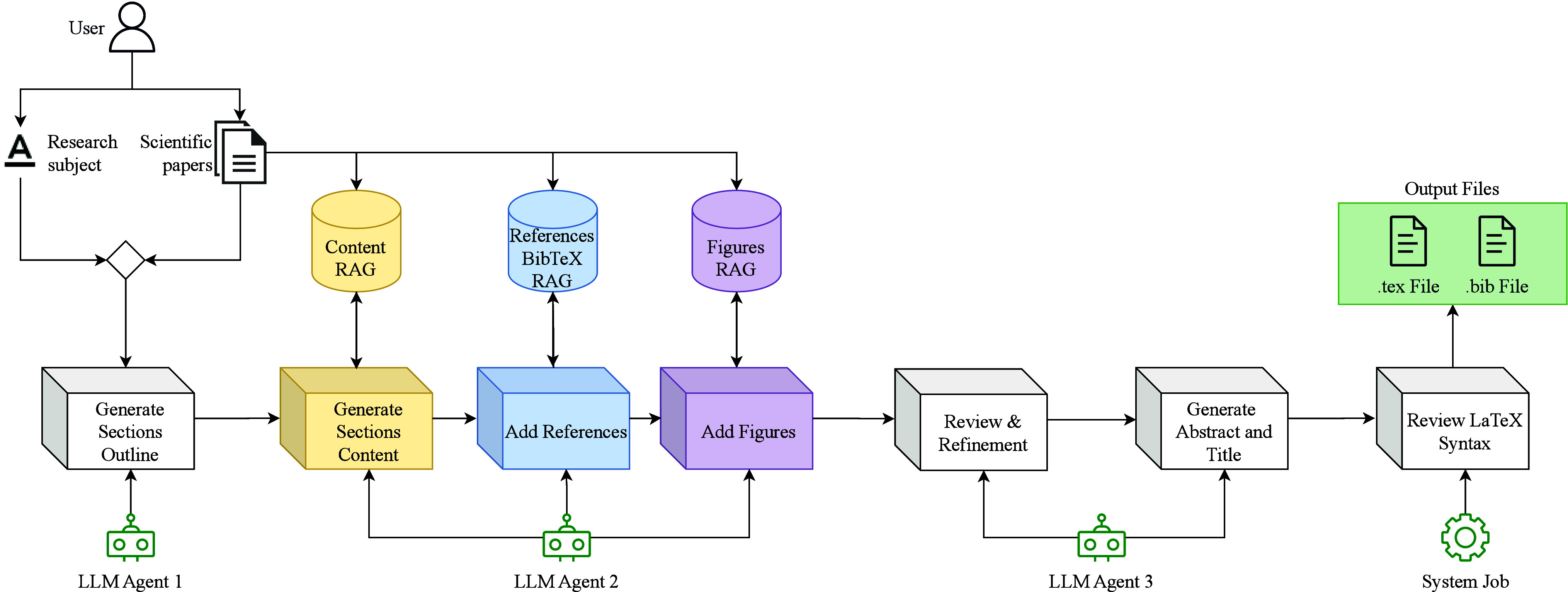
Complete AI Survey Writer pipeline, from research subject selection to the final LaTeX documents. The process starts with input from the user, and the selected scientific papers are processed into three RAG databases (Content, References BibTeX, and Figures). LLM agents generate the paper’s section outline, retrieve and synthesize relevant information, and integrate references and figures. The final review stage ensures coherence, and an automated system job validates LaTeX syntax before outputting the.tex and .bib files.

The following sections detail each stage of the AI Survey Writer, from constructing the RAG databases to finalizing the survey paper.

### 2.1 Creating RAG Databases

We construct three distinct RAG databases before generating each paper section. These vector databases are fundamental in retrieving relevant information and ensuring content accuracy, citation integrity, and structured referencing. Each RAG is a vector-based retrieval system built using FAISS (Facebook AI Similarity Search),^23^ an efficient library for similarity search in high-dimensional spaces designed to facilitate retrieving semantically similar contexts. Unlike traditional keyword-based search, FAISS stores and indexes vector embeddings, enabling semantic search, where conceptually related information is retrieved even if exact keywords are not matched. Using vector embeddings in RAG databases is crucial to enhance retrieval quality. Instead of relying on literal text matches, embeddings allow similar terms to be recognized, improving the accuracy of scientific literature content retrieval. We employ Snowflake’s Arctic-embed-l-v2.0 model^24^ to generate the embeddings, demonstrating high precision in scientific document retrieval. Additionally, we apply a confidence threshold of 0.9 to filter out low-relevance retrievals. The three RAG databases illustrated in Figure 2 serve the purposes of addressing the retrieval of Content, References, and Figures, respectively. The **Content RAG** stores vector embeddings of textual content extracted from user-provided reference PDFs. This RAG is the primary knowledge source for AI-generated text, ensuring retrieved content remains contextually relevant to each section. The retrieval process involves the following steps:

1. A query is constructed using the section description.
2. The system retrieves the top-k most relevant text chunks using semantic similarity search.
3. The retrieved chunks are used as context for generating the section text.

**Figure 2:**
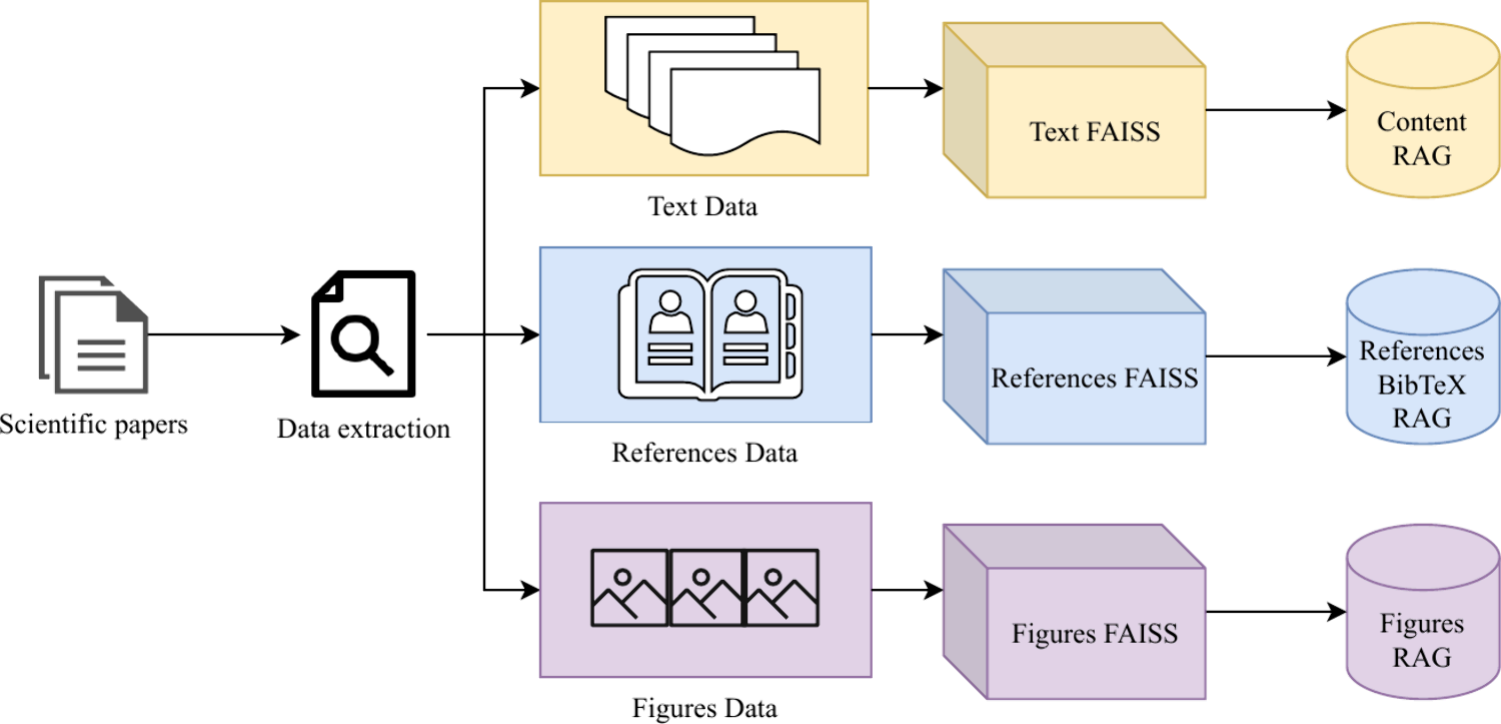
Three RAG databases in the AI Survey Writer pipeline. Scientific papers undergo data extraction, where textual content, references, and figures are separately processed using FAISS. The extracted data is stored in the Content RAG, References BibTeX RAG, and Figures RAG databases, ensuring accurate and structural retrieval for the AI survey writer.

This approach reduces token usage per API request while ensuring that the LLM focuses only on relevant content for each section, preventing the inclusion of irrelevant or hallucinated information. The **References BibTeX RAG** is designed to ensure accurate citation retrieval. Since LLMs often struggle with citation formatting and integrity, this database ensures that references are accurately extracted, stored, and retrieved. The construction process is as follows:

1. Extracting the bibliography section from each reference PDF.
2. Identifying titles and authors of all cited works using an LLM.
3. Querying the Crossref API to retrieve corresponding BibTeX entries.^25^

Once the entries are retrieved, duplicate records are filtered, and each entry is stored with its DOI, title, abstract, and keywords, allowing for fast and precise citation retrieval during text generation. Finally, the **Figures RAG** is designed to manage images and figures extracted from the reference PDFs. Figures often contain key insights, making their inclusion in AI-generated survey papers essential. By storing figures with corresponding captions, this RAG enables the LLM to retrieve and reference figures accurately, preserving scientific integrity and proper attribution. The extraction process is as follows:

1. Detecting figures within PDFs using Layout Parser and Detectron2.^26^
2. Assigning a unique ID to each extracted figure.
3. Matching the extracted figure with its original caption based on its position within the document.

Figure 3 presents a high-level overview of the final architecture of each RAG database, highlighting the structure and contents stored within them, including both the data and associated metadata for each item.

**Figure 3:**
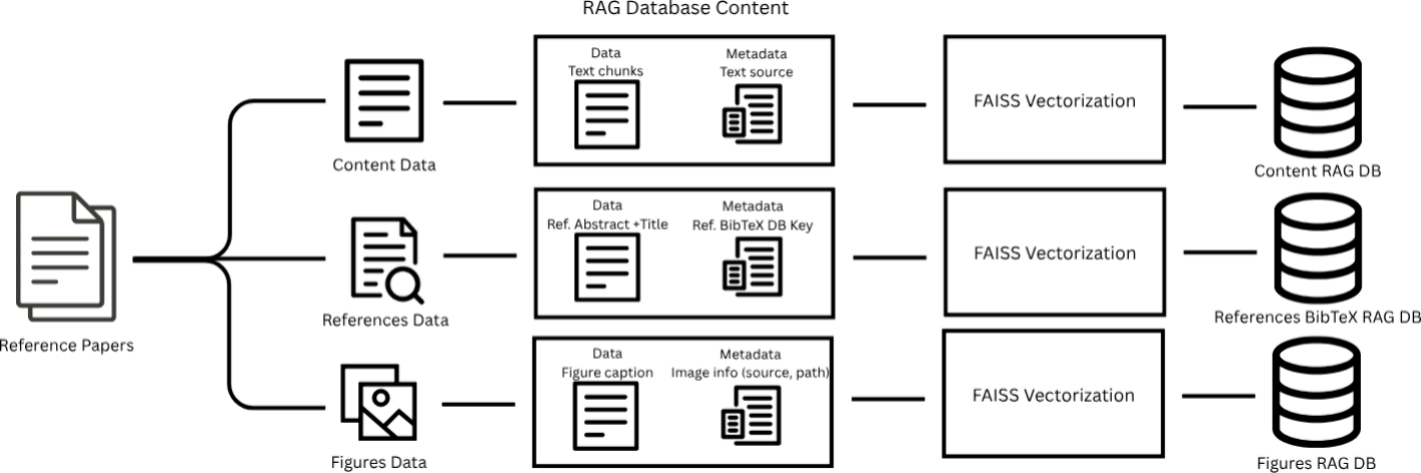
High-level overview of the architecture of each RAG database.

The choice of the retrieval thresholds k was primarily guided by practical constraints related to token limits. Since all retrieved chunks must fit within a single prompt alongside other necessary context, the value of k must be set to ensure that the input fits the model’s processing capacity. This constraint reflects a real limitation in current LLM-based workflows and is central to the solution’s design. To complement this practical constraint with empirical validation, we use grounded examples from real papers to evaluate the retrieval quality. In our pipeline, we use the retrieval system by including the section description and the paper subject as queries. We thus use section descriptions from actual papers as queries and assess whether the retrieved chunks include the original content associated with those sections. Standard retrieval metrics (e.g., precision@k, recall@k, hit@k) can be computed to measure how well the retrieval aligns with human-authored structure. This balance between token-aware design and empirical evaluation ensures that the top-k retrieval remains both feasible and effective. Empirical investigations showed that increasing k generally improves the hit rate and brings in a broader range of relevant content from diverse sources. These findings support our strategy of setting the top-k threshold based on the token limitations, which naturally controls the trade-off between coverage and input size.

### 2.2 Generating the Sections Outline

The first step in the AI-assisted survey writing process is establishing the document structure by generating an outline of sections and subsections. This step defines the logical flow of the paper and ensures that the final content is well-organized and comprehensive. To create an outline that represents the full scope of the reference papers, an LLM agent processes the entire text of all reference PDFs. Unlike in later stages where a RAG is employed for content generation, this step does not use RAG. Instead, it requires the LLM to analyze the entire corpus directly, synthesizing its structure without relying on segmented retrieval. We had two major criteria for choosing a model for this task: it had to be free and offer a sufficient token limit for processing large text volumes. Based on these requirements, we selected Gemini 2.0 Flash, which is optimized for efficient processing of long input sequences and offers the largest freely available context window—up to 1,000,000 tokens. This allows for the direct inclusion of complete reference content without relying on a RAG-based strategy to minimize token usage. As a result, the model benefits from full-context awareness across all provided sources. Although we experimented with RAG in combination with other, more powerful LLMs to optimize token efficiency, the reduced context coverage led to inferior outputs—specifically, outlines that were narrower in scope and lacked detail. Large language models are evolving at a very fast pace, and other free options will likely become available in the near future.

The output of this step is a structured JSON object, which serves as the foundation for subsequent content generation. The JSON format ensures machine readability and allows downstream models to follow the outline systematically. Each section in the JSON object contains:

- **title** - The section title.
- **description** - A brief overview of the section, often including a breakdown of its subsections.

This structured representation of the paper enables precise control over content organization and guarantees that each section aligns with the core themes extracted from the reference documents.

### 2.3 Generating Section Content

The next step involves generating content for each section. This is achieved with a second LLM agent, responsible for synthesizing relevant information retrieved from the Content RAG. Unlike the previous step, where the LLM processes the full text of the reference PDFs, RAG is employed in this step to ensure that each section is grounded in relevant, high-quality, and reliable sources. The process follows three stages:

1. **Query Construction**: The section descriptions obtained in the step detailed in Section 2.2 are used as a query for retrieving relevant text chunks from the Content RAG.
2. **Similarity-Based Retrieval**: The query is processed through FAISS, retrieving the top-k most relevant text chunks using vector similarity search.
3. **LLM-Based Content Generation**: The retrieved text chunks provide the context for the LLM to generate a coherent, well-structured section.

This RAG-based retrieval ensures that the LLM remains grounded in factual information, improving the accuracy of citations and reducing the risk of hallucination. This approach maximizes coherence, factual accuracy, and contextual relevance in AI-generated scientific survey papers by leveraging structured section descriptions, efficient vector retrieval, and an advanced LLM model for content generation.

### 2.4 Adding References and Figures

The text is then enhanced by incorporating references and figures to ensure the survey paper is well-supported with relevant citations and visual elements, improving clarity and reinforcing key points. Figures are essential in scientific writing, particularly in survey papers where visual representations help readers grasp complex concepts more efficiently. An LLM-driven postprocessing phase is applied to integrate relevant figures into the paper. This process begins with an LLM agent analyzing the completed section alongside the full reference PDFs. Suppose the model identifies a potential benefit in including a visual representation. In that case, it generates a LaTeX figure block containing a figure name and a caption formulated based on existing references to figures within the text. A semantic similarity search with the caption is performed using the Figures RAG to retrieve the most relevant image. The query for retrieval is constructed based on the generated caption, allowing the system to identify conceptually similar figures rather than relying solely on keyword matching. The best-matching image is selected based on its vector similarity score, ensuring that only highly relevant figures are incorporated. After choosing the most suitable figure, the system automatically inserts the LaTeX figure block into the corresponding section. The final insertion includes the local path of the image, ensuring proper document formatting. Using RAG-based retrieval, this approach aligns figures accurately with textual content, reducing hallucination risks and preventing mismatched visual elements.

Proper citation is also essential in academic writing, ensuring that claims made in the paper are traceable, credible, and well-supported. LLMs often generate incorrect or fabricated references, so we apply a structured retrieval-based approach to maintain citation integrity. The References BibTeX RAG is queried using each paragraph from the generated text as input to associate the top-k relevant references with each section. This FAISS semantic similarity search enables the retrieval of contextually aligned citations rather than relying on lexical keyword matching. The strategy ensures that references retrieved are directly relevant to the section’s content, reducing the risk of including irrelevant sources or misattributing them. The appropriate references retrieved are sent to an LLM agent, which is prompted to include a portion of the relevant citations at the correct locations within the text. If multiple references are considered highly relevant, the LLM may group citations to provide comprehensive attribution while adhering to academic referencing standards. This structured approach ensures that all citations are correctly formatted and accurately represent the paper sources. Systematically integrating figures and references ensures the generated survey paper meets rigorous academic standards, providing visually informative content and reliable source attribution. Empirical investigations indicated that setting k = 50 yielded adequate results in general.

### 2.5 Review and Refinement

The pipeline includes an iterative review step to improve the general text’s clarity, completeness, and coherence, ensuring that the final survey paper meets academic writing standards. The process comprises three stages. First, a writer agent generates a preliminary “draft” version for each section, grounded solely in the provided references. Then, a reviewer agent is prompted to assess each section by comparing it to the source material and producing structured review directives. These directives are guided by a prompt designed to elicit focused feedback, such as identifying missing yet relevant topics, detecting redundancy, suggesting simplifications for clarity, and flagging any factual inaccuracies or hallucinations. Finally, a second writer agent incorporates the review directives to revise and improve the draft. This decomposition of the writing task into smaller, focused steps—mirroring advisor-advisee dynamics in academic writing leads to markedly improved outputs. By isolating planning, critique, and rewriting, each model operates under more constrained, objective subgoals, which mitigates the failure modes often encountered when attempting to fulfill multiple complex objectives within a single-generation prompt. The nature of the task makes it difficult to conduct a formal quantitative evaluation; instead, our assessment of results relied on detailed qualitative comparisons between the scratch and the reviewed versions, as well as close monitoring of the review directives themselves. These assessments consistently show improvements in coherence, coverage, and factual consistency. In particular, the review phase plays a crucial role in identifying and eliminating hallucinated content, confirming the practical value of this iterative refinement strategy.

The LLM analyzes each section alongside additional reference materials retrieved from the Content RAG to identify areas for improvement. By examining this contextual information, the model identifies potential weaknesses in the section’s depth, clarity, and structure. The system outputs structured review directives for necessary refinements. These directives typically involve expanding discussions, clarifying ambiguous statements, or even enhancing the insertion of figures and citations where appropriate. A key advantage of this approach is its ability to contextually assess whether a section is sufficiently detailed or if additional explanations are required. For example, if a particular subsection references a complex scientific concept, the review agent may suggest further elaboration or the inclusion of a supporting reference. Similarly, if a figure is added but lacks adequate discussion in the text, the system may recommend a more in-depth analysis of the visual data. This automated review process ensures that the generated survey is structurally sound and rich in analytical depth and academic rigor.

With the review directives established, the refinement phase starts. An LLM refinement agent processes the suggested improvements and the same reference materials, ensuring that modifications remain grounded in relevant and accurate information. This iterative approach allows targeted revisions, improving the final paper’s flow, coherence, and factual integrity. During this phase, special attention is given to maintaining consistency across sections. Since the paper is generated in modular parts, refinements ensure that terminology, tone, and writing style remain uniform throughout the document. Additionally, any overlapping content between sections is restructured to eliminate redundancy while preserving critical insights. This review process is necessary to ensure the survey is an insightful and well-organized document, not just a compilation of retrieved knowledge.

### 2.6 Generating the Abstract and Title

The abstract and title are generated using an LLM agent that processes the entire document in a single pass, also requiring a large context window agent, similar to the step detailed in Section 2.2. Unlike the earlier steps, this step does not rely on RAGs. To generate the abstract, the entire paper text is provided as input to the LLM, which is prompted to produce a concise yet comprehensive summary. The abstract must capture the core contributions, methodology, and findings, and the content must align with academic writing conventions. Additionally, the assigned title must reflect the paper’s central theme while preserving clarity. Since abstract writing is a summarization task, the LLM model is guided with specific constraints to ensure the output is succinct, typically limited to 200–300 words, and structured to emphasize six aspects of the work: Introduction, Context, Research Gap, Methodology, Results, and Discussion.

### 2.7 Reviewing LaTeX Syntax and Final Paper Output

With the entire text and references in place, the system performs an automated review of the LaTeX source code to ensure correct formatting. Unlike previous steps, which rely on LLMs for content refinement, this stage is handled purely by Python utilities and regular expression validation techniques. To prevent syntax errors and inconsistencies, the system applies LaTeX-specific filtering by

- Eliminating Markdown artifacts that LLMs may introduce, such as unnecessary code block markers (e.g., “latex”).
- Validating citation keys to ensure all citation commands refer to existing entries in the .bib file.
- Ensuring proper BibTeX formatting using bibtexparser, preventing issues with malformed references.
- Verifying figure paths to confirm that all inserted images are correctly referenced within the document.

The final output consists of a fully formatted LaTeX project, including a.tex file containing the entire paper with adequately structured sections, references, and figures, and a .bib file storing the retrieved and formatted BibTeX references.

In summary, we employed separate RAGs to accommodate the distinct types of metadata associated with each database. For example, the Content RAG stores textual chunks extracted from article PDFs, enriched with metadata such as authors, BibTeX entry keys, file paths, and titles. The Figure RAG, by contrast, stores only figure captions, along with metadata indicating the image file path and the source PDF. Finally, the References BibTeX RAG includes paper abstracts and titles as primary content, with the BibTeX entry key as metadata. This separation promotes modularity and clarity within the system, as each RAG is tailored to handle a specific data modality and its corresponding context.

## 3 Automated Generation of Review Papers

As a proof of concept, we employed the methodology outlined in the previous section to generate review papers on Langmuir and Langmuir–Blodgett films. Our system was designed to produce review papers on a specific subject using limited in-house computational resources and relying primarily on commercial LLM tools. Note that the system was not trained or explicitly instructed on how to produce a high-quality review. For example, we implemented mechanisms to obtain texts of appropriate length for a review paper—something not feasible through direct interaction with commercial LLM tools. We also introduced steps to enable the system to include figures and references; however, it was not guided on what constitutes an appropriate figure or reference. In other words, no human input was provided to train the system in producing excellent review papers. Since we adopted two distinct strategies, the results will be presented separately in Sections 3.1 and 3.2.

### 3.1 AI Writer: Input from User-Selected Papers

We conducted three experiments using this strategy: (i) input given by five published review papers on the topic; (ii) input given by 21 papers that included the five reviews from the previous experiment; and (iii) input given by 20 papers,^27–46^ which are the same from the previous experiment, but excluding one review paper that appeared to dominate the content of one generated version. The generated papers are labeled as sections S4 through S6 in the Supporting Information, and the input references for each experiment are listed in Table S4. In all cases, the abstract, the selection of topics included in the review paper, and the coverage of the field’s fundamentals were considered appropriate by the expert author and according to the rubric described in the Supporting Information. The rubric contained 19 items describing the sections and subsections expected from a comprehensive review on Langmuir and Langmuir–Blodgett films, and the review papers covered between 15 to 17 of them. They described the history of the films, the experimental methods for film fabrication and characterization, applications, and future directions. It is worth mentioning that the challenges and suggestions for further work, particularly those aimed at making these films suitable for real-life applications, were all well-founded. Some variations in the overall structure were observed. For example, the version obtained with five papers had an entire section on layer-by-layer (LbL) films, which is difficult to justify in a review paper on Langmuir and Langmuir–Blodgett films. However, a comparison between Langmuir–Blodgett and LbL would be expected. The figures and references were, for the most part, adequate in all versions, though we did not check all the references individually, as we shall comment upon later. No signs of hallucinations were apparent. Although the contents of the versions varied, they were appropriate for the subject. In summary, each version can be considered an excellent starting point for producing a high-quality review paper.

Let us now comment on the central issues identified in a human expert’s inspection of the generated versions. The intended subject included Langmuir and Langmuir–Blodgett films, but the importance of Langmuir films as models of cell membranes was not addressed in any of the versions. There were *en passant* mentions, but no elaboration. There were also stylistic problems—for example, the introductions tended to be longer than necessary, and in two versions, they included figures, which is unusual. These figures would be better placed in other sections of the review. All versions included a section on future directions and perspectives, which is appropriate; however, the content of these sections often overlapped with the Conclusions, resulting in some repetition that could have been avoided. Repetition also occurred elsewhere in the text, despite the refining step introduced in the pipeline. It is possible that this refining process was insufficient to eliminate the limitation of generating content in segments, since commercial LLM tools cannot produce long texts in a single step, as previously noted. None of the versions achieved the in-depth literature discussion essential for a review paper, though some hint of critical analysis could be perceived in the section on future directions. As is typical of text and summaries generated by commercial LLM tools, the descriptions tended to be generic and superficial. There were also minor issues in the generated review papers, which, while not critical, would not be acceptable in a high-quality publication. For instance, some figures were misplaced, or their numbering did not follow the order of appearance. In a few cases, inappropriate figures were generated; in one case, a figure was incorrectly described in the text. In Section 4 we discuss how these problems can be addressed to produce higher-quality versions, since many of the limitations mentioned here also apply to the reviews generated using the second strategy.

The review versions had no significant differences, apart from one of them including a section on LbL films; in this case, the outcome was apparently strongly influenced by one of the input review papers. This suggests that only a few review papers may be sufficient for the system to generate reasonably good reviews. However, as discussed in Section 3.2, we obtained considerably superior versions using a much larger number of papers extracted from citation networks.

### 3.2 AI Writer: Input from Citation Networks

The strategy described in Section 3.1 emulates the steps of a human author writing a review paper, namely, selecting multiple representative papers in the field to guide the writing, from topic selection to the paper’s outline. Because the number of input papers is undoubtedly limited to tens or hundreds, there is an inevitable bias in the selection. Furthermore, the selected papers may not cover the subject in its entirety. To mitigate this limitation, we considered starting from a literature landscape on the chosen topic, as in previous work,^22^ and selecting representative papers from this landscape as input. We employed the method proposed in^21^ to obtain this landscape for Langmuir and Langmuir–Blodgett films. In this approach, a citation network is built from a pool of papers retrieved from a search on the topic. Then, clusters of densely connected papers are identified in the giant component of the network, and keywords characteristic of each cluster are also identified. The strategy is to select papers from highly relevant nodes in the network as input for the LLM to generate the review paper outline and, eventually, the entire survey paper.

A search in the OpenAlex repository on February 19, 2025, using the query *((langmuir OR langmuir-blodgett OR blodgett) AND (monolayer OR film) NOT adsorption NOT probe))* retrieved 51,330 papers, of which 49,494 were written in English and were, therefore, used in the experiment. The resulting citation network, depicted in Figure S2 in the Supporting Information alongside its detected clusters, had a giant component with 30,954 papers. The network clustering procedure uses the Infomap community detection method, based on the Map Equation.^47,48^ Then, the representative keywords for each resulting cluster are identified. For this purpose, keyword scores are computed as the maximum difference between the keyword frequency in the paper abstracts within the cluster versus their frequency in the paper abstracts of all the remaining network clusters. A selected number of top-scoring keywords are taken as the cluster representatives, see Table S1 in the Supporting Information.

A further filtering step was necessary to exclude papers unrelated to Langmuir or Langmuir–Blodgett films, as the query retrieved some papers related to probes and adsorption due to their association with the scientist Irving Langmuir. This can be observed in the cluster keywords in Figure S2 in the Supporting Information, e.g., a representative keyword for cluster C is “sorption”. The filtering step consisted of multiple iterations of clustering the network and removing those clusters with representative keywords that suggested a diverging topic until only relevant clusters remained. This assessment of clusters’ topics was done in an AI-assisted fashion, with an LLM agent interpreting the clusters’ topics from the associated keywords and indicating the most relevant ones. The resulting network consisted of 27,233 highly relevant papers split into five clusters, shown in Figure 4. Two filtering iterations were sufficient to obtain this network. Further details about the clusters in the original network (Figure S2), the network obtained after the first filtering step and its clusters, and the clusters in the final network (Figure 4) are provided in the Supporting Information as, respectively, Table S1, Figure S3, Table S2, and Table S3.

**Figure 4:**
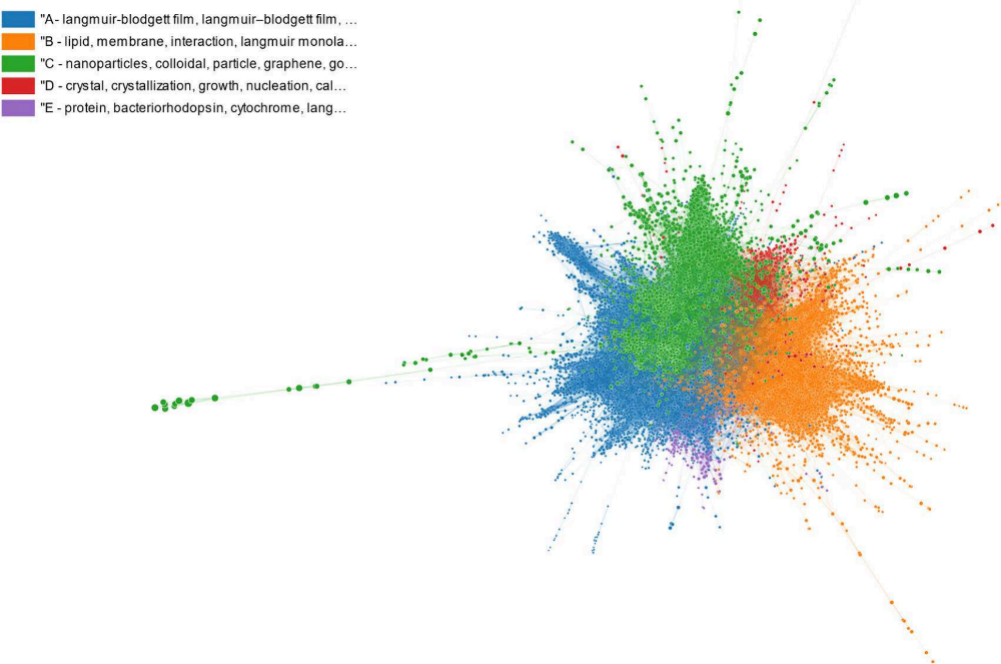
Final citation network after filtering, and its five clusters.

We generated two review papers using this second strategy, which are presented in sections S6 and S7 in the Supporting Information. The criterion for selecting the input papers was to identify, within each cluster, the nodes with the highest number of ingoing links - that is, the papers most cited by other papers in the same cluster. In a few cases where the corresponding paper was not accessible to the authors, it was replaced by the paper associated with the next node in the degree rank. We selected a number of papers proportional to cluster size, in order to preserve the relative topic importances. Using this strategy, 138 papers were selected from the network shown in Figure 4 and taken as input to produce the first paper. The AI Writer system suggested the title “Langmuir and Langmuir-Blodgett Films: A Survey of Fundamentals, Advances, and Applications in Molecular Assembly,” which reflects the content of the generated paper. The abstract is excellent, and the overall structure is appropriate, as the paper contents include 18 items of the rubric. Unlike the reviews obtained with the first strategy, which focused primarily on Langmuir–Blodgett films, this version emphasizes Langmuir films. Three sections are dedicated to fabrication and characterization methods and materials. These sections offer detailed descriptions, which are appropriate—albeit heavily based on a comprehensive, highly cited previous review.^49^ Advanced topics and emerging trends are discussed in a separate section, including some applications. This latter section successfully highlights significant recent developments and correctly identifies trending topics. The figures and references are largely adequate. Due to the reference selection method employed — all references include their DOI numbers—hallucinations in the bibliography were effectively avoided. While the text remains somewhat superficial, as noted in the versions produced using the first strategy, it does include some in-depth analysis in the section on emerging trends. With regard to the critical analysis expected in review papers, a major challenge for LLM-based tools will be handling competing views in the literature. These tools will likely default to the most prevailing opinion due to statistical dominance. However, this challenge may also be mitigated by training the tools to highlight the existence of divergent perspectives when they are present.

Overall, the generated review paper is an excellent starting point for developing a high-quality manuscript, mainly focusing on Langmuir films. Some stylistic issues noted in earlier versions also appeared in this one. For example, the Introduction was longer than usual and included figures, which is uncommon. Since this version was based on papers published over several decades, many figures and references are outdated. Ideally, the review paper should prioritize more recent publications.

The observation that the review content seemed heavily influenced by a particular source^49^ motivated us to exclude that specific reference and generate another version using the remaining 137 papers. This second version, titled “Langmuir and Langmuir–Blodgett Films: Foundations, Frontiers, and Future Directions in Interfacial Molecular Engineering,” is the highest quality among all versions produced. It included 17 items of the rubric in its contents. The abstract and outline are excellent and provide a well-balanced overview of the field. Besides sections on fundamentals, fabrication, and characterization methods for Langmuir and Langmuir–Blodgett films, the paper included a substantial section on applications. Two additional sections covered advanced topics, emerging trends, challenges, and future directions. These latter sections offered a critical field analysis, as expected from a high-quality review. It is worth noting, however, that this analysis was primarily based on the perspectives of two prolific authors in the field. The first and second versions differed considerably. We found it intriguing that removing a single paper from the input could impact the output as much. One hypothesis is that the paper removed was introducing a RAG retrieval bias. Because of vocabulary overlap, when the RAG retrieved chunks using similarity search, this single paper was ranked at the top for many queries, substantially impacting the output paper.

As for deficiencies, the Introduction was again longer than usual and included figures, which is uncommon. Some content repetition from the challenges section was observed in the Conclusion; ideally, these two sections should be merged and streamlined. More recent references should have been prioritized, as observed with the first version produced using this strategy.

The higher quality of the versions generated using 137 and 138 papers, compared to those using 21 or fewer, can likely be explained by the following. Using a RAG-based model, the system can access a database containing all available articles. Before the LLM generates the text, it searches this database to identify the most relevant articles to the content under production. In other words, the LLM relies directly on these documents to compose the final response or the generated text. Therefore, the more extensive and diverse the article database, the higher the chances that the search will return relevant texts, resulting in a more coherent output. In summary, the versions with more articles produced better texts because the database was richer, increasing the likelihood of retrieving valuable documents during the generation process.

## 4 Analysis of Machine-Generated Reviews

The analysis of the papers generated using the two strategies reveals that, in their current state, automatically generated review papers would not meet the most stringent review standards of a prestigious journal. This is confirmed by feedback from experts, as discussed below. This is not a surprising conclusion, given the original intention: to produce review papers using in-house computing resources and commercial LLM tools, rather than training a system with human input to generate high-quality reviews. Despite these limitations, the machine-generated reviews were considered excellent starting points for writing a review paper by nine experts in Langmuir and Langmuir–Blodgett films. They were asked to evaluate the version generated with 137 papers — which we considered the best among the five versions produced—and to complete the evaluation form provided in the Supporting Information.

The questions mimicked the review forms used by academic journals, beginning with an overall recommendation offering four options: accept as is, minor revision, major revision, or do not publish. Other questions addressed the expected coverage of the review paper, as well as the quality and accuracy of figures and references. Experts were also asked whether the generated paper could be considered as a good starting point, and how its quality compared to a first draft typically produced by a student or postdoctoral researcher. There was consensus among the experts: all agreed that the generated version was a good starting point and that its coverage was appropriate for the field. Notably, eight out of the nine experts judged the generated paper to be superior to what they would expect from a student or postdoc draft. Recommendations ranged from major to minor revision, and all reviewers offered suggestions for improvement. The most common criticisms concerned the figures, many of which were taken from older papers and lacked visual appeal. Additional criticism focused on the insufficient emphasis given to some important topics.

It is worth mentioning that the observed flaws are not due to inherent limitations of the LLM models or conceptual flaws in natural language processing. They can all be tackled with existing technologies. In the following, we illustrate two scenarios for addressing the limitations.

In the first scenario, we assume that research groups, like ours, may use commercial LLM tools to implement multiple independent modules that together compose an intelligent system that produces high-quality review papers. For instance, a figure selection module could use supervised learning to train a model on a large data set of figures chosen from published reviews, relying on essential human expert feedback. In fact, we found the choice of the figures included in the several generated versions quite disappointing in terms of the quality of the drawings and images, an issue that could be tackled with such a figure selection module. When selecting references, avoiding hallucinations is a critical issue. It would also be possible to implement a reference-checking module that verifies the references against literature databases such as OpenAlex, and could also search for suitable alternatives for references not identified. Ultimately, it could include a validation step to check whether the contents of a particular reference indeed support any statements linked to it in the text. Such a solution is feasible, although it may be costly for the many references typical in review papers.

The most challenging limitation in generated review papers is the lack of in-depth, critical field analysis. A module designed for this task would require substantial human input combined with supervised machine learning. Lastly, an overarching strategy may guide the development and evaluation of the various modules. This would involve generating multiple review papers for a given field, executing the pipeline in Figure 1 (or an equivalent one) with varying parameters. For example, one may vary the number and types of input papers, or include additional information obtainable from citation networks, as discussed in Section 3.2. An independent module could then compare these multiple versions to eliminate deficiencies such as inappropriate references, figures, or statements, and to aim for a deeper level of analysis.

In a second, more optimiztic scenario, publishing houses might collaborate with LLM tool developers to overcome the current limitations and streamline the entire pipeline. All the improvements described in the first scenario would be feasible, with enhanced capabilities for generating long, coherent texts without segmentation. It could significantly facilitate the comparison of multiple review versions, enabling supervised machine learning to better address the challenge of achieving in-depth, critical analysis. The pipeline in Figure 1 could be seamlessly integrated into a review-generating tool, including the more demanding strategies based on network analysis for input selection. The modules described in the first scenario would remain relevant in this integrated approach.

Let us now dwell upon the advantages of having such a review-generating tool. The first obvious advantage is to speed up the preparation of review papers, particularly considering that the proposed methodology is applicable to any subject. There are also significant advantages regarding the topic’s breadth of coverage. Rather than a limited number of papers from the literature, an automatic generation process can consider thousands or tens of thousands of papers. Here, we have exploited citation networks only to select relevant papers, but additional useful information can be extracted from the networks and their topology.

Furthermore, generating several distinct versions of a review paper may contribute to reducing the inevitable human bias. Besides, the future generation of LLM-based tools will likely address currently unsolved issues, such as fact-checking and attaining high accuracy. Indeed, we mentioned that we did not check all the references from all the versions individually. Usually, even human experts spend limited time on this type of verification. As for fact-checking, the limited human text processing capability hampers extensive in-depth analyses of large quantities of text. AI will likely handle these issues in the future, contributing to improved reliability of science and technology documentation.

The pipeline introduced here—demonstrated through a proof-of-concept review paper on Langmuir and Langmuir–Blodgett films—can be applied to any scientific topic. This is illustrated by a review on nuclear magnetic resonance, generated using the same procedures as Strategy 2. A citation network was built using the query “nuclear magnetic resonance” and “porous or pore,” and 58 of the most connected papers were selected as input for the pipeline to generate a review using Gemini-2.5-Flash. The resulting paper, included in the Supporting Information, was evaluated by an expert in the field. This expert expressed opinions similar to those of the reviewers of the Langmuir and Langmuir–Blodgett review, emphasizing that the coverage was adequate and that the generated paper serves as an excellent starting point for a high-quality review.

As for the next steps in the use of AI for academic publishing, we anticipate the development of dedicated tools to (i) select appropriate figures for inclusion; (ii) verify that references are suitable and correctly placed; and (iii) perform fact-checking. The latter is undoubtedly the most challenging and will likely require diverse strategies implemented in multiple agents to cross-validate outputs.

## 5 Implications for Scientific Publishing

The various versions of an automatically generated review paper presented here suggest we are approaching a key milestone in developing machine-generated scientific writing. It is worth noting that these versions were produced using limited computational resources and commercial LLM tools. It is reasonable to assume that using dedicated, specialized LLMs could mitigate the deficiencies identified in the generated papers. Once this becomes possible, it will have profound implications for scientific publishing. Some positive outcomes include the accelerated creation of review papers, unbiased knowledge synthesis, and increased accessibility through AI-generated content tailored to different audiences, such as students, specialists, or policymakers. Additionally, AI systems could produce dynamic reviews that are automatically updated as new research is published.

However, several important issues must be considered, in line with the concerns already expressed in the introduction of this paper. First, the issue of authorship arises: who should receive credit for the review paper? This also raises concerns about accountability, intellectual ownership, and proper citation. A shift in authorship criteria should perhaps be discussed shortly. Moreover, the training corpus may include flawed or retracted papers, leading the system to learn from incorrect sources. Identifying who should be held accountable for factual errors or omissions in AI-generated reviews will be challenging. Given the overwhelming volume of literature published daily, manual verification may be virtually impossible. As mentioned above, we anticipate that, in the future, new AI tools will be developed specifically for fact-checking, as only such tools will be capable of processing the vast amounts of text in the scientific literature. Other ethical challenges include the potential for “review spam,” in which AI mass-produces papers, flooding the literature with superficial or redundant content. Publishing models would also be affected, particularly for journals specializing in review articles. The impact of tools capable of automatically generating papers with original content—which are also under development—is more difficult to assess. For one, it will be challenging to determine whether the ideas introduced are truly original. Ensuring that the research methodology and results are sound and accurate is even more difficult. In fields such as applied materials, these AI agents are unlikely to have a major impact, given the experimental nature of most research in the area. Instead, they may serve as assistants for literature surveys and data analysis, without replacing human expertise in most aspects of the research process. Nevertheless, relying on such agents will still require accounting for previously discussed ethical concerns.

## Supplementary Material



## Notes

Description of RAGs, Rubric to analyze paper contents, evaluation forms for experts, automatically generated versions of review papers (PDF)
