# Supplementary material for: AI-Assisted Tools for Scientific Review Writing: Opportunities and Cautions
Source: ACS Appl Mater Interfaces. 2025 Aug 13;17(34):47795–805. doi: 10.1021/acsami.5c08837 (PMC12400276; doi:10.1021/acsami.5c08837)
Supplement: Supplementary file 1 [file am5c08837_si_001.pdf]

Supporting Information - AI-assisted tools for scientific review writing: opportunities and cautions

Julio C. M. C. Silva[1], Rafael P. Gouveia[2], Kallil M. C. Zielinski[1], Maria Cristina F. Oliveira[2], Diego R. Amancio[2], Odemir M. Bruno[1], and Osvaldo N. Oliveira Jr.[1]

[1]Sao Carlos Institute of Physics, University of São Paulo, 13560-970, São Carlos, SP, Brazil
[2]Institute of Mathematics and Computer Sciences, University of São Paulo, 13560-970, São Carlos, SP, Brazil

June 29, 2025

S1 Rubric to evaluate the sections of a review paper on Langmuir and LB films

- History
- Fabrication and Characterization methods
  - For Langmuir film
    - Langmuir trough and fabrication procedure
    - Materials employed
    - Surface Pressure
    - Surface Potential
    - Microscospies
    - Spectroscopies
    - Structural Characterization
  - For LB films
    - Types of LB films
    - Materials Employed
    - Microscopies
    - Spectroscopies

    * Structural Characterization

- Applications
  - Langmuir monolayers as cell membrane model
  - Sensors and biosensors
  - Electronic devices and molecular electronics
  - Nanoarchitectonic
- Prospects and Conclusion

S2 Evaluation Sheet for the Review Paper Generated Automatically

Please consider the manuscript attached which was written as a review paper on Langmuir monolayers and Langmuir-Blodgett Films. Below is a list of evaluation items for the various aspects of the manuscript. Please assume that the manuscript was submitted for a prestigious journal.

1) General Evaluation

( ) Accept as is. No changes are required

( ) Minor Revision

( ) Major Revision

( ) Do not publish

Comment if you wish:

2) Are the sections appropriate for a review on the subject of Langmuir and LB films?

( ) Yes

( ) No

( ) Partially

Comment if you wish:

3) Are the contents of the sections appropriate for the subject?

( ) Yes

( ) No

( ) Partially

Comment if you wish:

4) Are the contents of the manuscript accurate for the subject?

( ) Yes

( ) No

( ) Partially

( ) Could not check

Comment if you wish:

5) Are the figures in the manuscript appropriate for the subject?

( ) Yes

( ) No

( ) Partially

Comment if you wish:

6) Are the references in the manuscript appropriate for the subject?

( ) Yes

( ) No

( ) Partially

Comment if you wish:

7) In case the manuscript is considered unacceptable in the present form, do you consider it provides a good starting point to write a review paper on Langmuir and LB films?

( ) Yes

( ) No

Comment if you wish:

8) How do you compare the quality of this manuscript with a first version of a manuscript on the topic produced by one of your students or post-docs?

( ) Better than produced by a student or post-doc

( ) Worse

( ) Similar level

Comment if you wish:

S3 RAG: Retrieval-Augmented Generation

Retrieval-Augmented Generation (RAG) is a technique that enhances the capabilities of LLMs by integrating them with external information retrieval systems. While traditional LLMs generate responses based solely on the knowledge encoded in their training data, RAG introduces a retrieval mechanism that allows the model to fetch relevant documents, passages, or structured data from an external knowledge base. This additional step helps ground the model's responses in factual data [1]. By dynamically incorporating retrieved knowledge, RAG systems can bridge gaps in the model's pre-trained dataset, making them particularly effective for tasks that require access to current events, evolving scientific research, or domain-specific expertise.

The RAG framework consists of two main stages, as depicted in Figure S1:

1. **Document Processing Pipeline (Figure S1.a:** This stage involves converting raw documents into a structured format suitable for retrieval. The documents are split into smaller chunks, which are then transformed into vector embeddings using a neural encoder. These embeddings are then stored in a vector database, enabling efficient retrieval based on semantic similarity;

2. **Retrieval and Generation Process (Figure S1.b):** In response to a user query, the Retrieval Module is responsible for searching the vector database to retrieve the k most similar documents using similarity search techniques such as FAISS [2] or BM25 [3]. These retrieved documents are used to construct a richer prompt, which is passed to the Generator Module. The Generator then synthesizes a response by incorporating the retrieved information and the model's pre-trained knowledge, enhancing factual accuracy and reducing hallucinations.

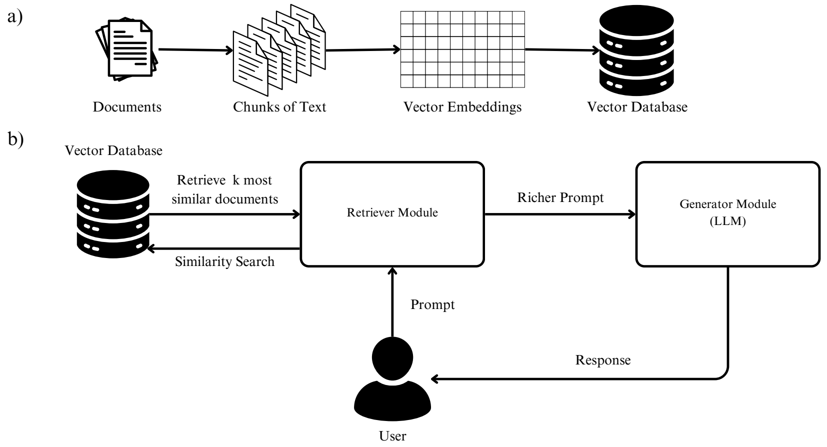

Figure S1: The RAG framework consists of two main stages. a) The Document Processing Pipeline converts documents into vector embeddings and stores them in a vector database for efficient retrieval. b) The Retrieval and Generation Process retrieves relevant documents using similarity search, refines the user prompt with the retriever data, and generates a fact-based response.

This approach is particularly beneficial when up-to-date information is crucial or the model's training data may not encompass recent developments. For example, RAG has been employed in applications requiring real-time data access, such as text summarization [4], healthcare [5], and question-answering systems [1].

S4 Network and clusters

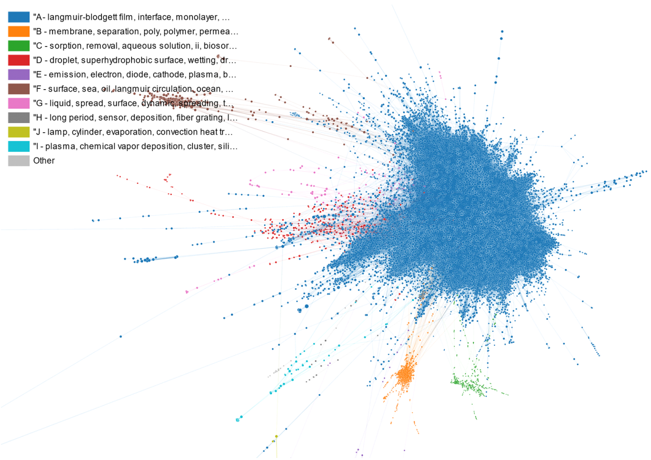

Figure S2: Giant component of the citation network created from the papers retrieved from the OpenAlex repository, with ten major clusters identified.

Table S1: Clusters of the network in Figure S2, along with the number of papers, their description provided by ChatGPT and the main keywords (with their importance values).

Cluster	Name	Size	Description	Keywords and Scores
A	Langmuir-Blodgett Films and Molecular Interfaces	28,650	Focuses on Langmuir-Blodgett films, monolayers, and molecular structures at interfaces. Includes discussions on lipid films, thin films, and crystallization, emphasizing their structural properties and interactions.	langmuir-blodgett film (0.273), interface (0.144), monolayer (0.127), molecular (0.107), langmuir–blodgett film (0.098), structure (0.094), langmuir monolayers (0.089), lipid (0.088), crystal (0.073), thin (0.072)
B	Membrane-Based Separation and Transport	627	Explores membrane technologies for separation processes, including polymer-based membranes, permeation, diffusion, and gas transport. Also covers sorption and water transport phenomena.	membrane (0.172), separation (0.135), poly (0.120), polymer (0.109), permeation (0.101), diffusion (0.094), gas transport (0.086), effect (0.080), sorption transport (0.077), water (0.073)
C	Sorption and Contaminant Removal	445	Examines sorption processes and their role in removing contaminants from aqueous solutions. Topics include biosorption, ion interactions, dye adsorption, and equilibrium studies.	sorption (0.161), removal (0.104), aqueous solution (0.081), ii (0.069), biosorption (0.052), ion (0.050), dye (0.048), onto (0.045), vi (0.042), equilibrium (0.037)
D	Droplet Dynamics and Superhydrophobic Surfaces	376	Covers droplet behavior on superhydrophobic surfaces, including wetting, impact, condensation, and coating applications. Explores interactions between liquids and surfaces with different properties.	droplet (0.218), superhydrophobic surface (0.131), wetting (0.116), drop (0.098), condensation (0.096), impact (0.095), water (0.080), coating (0.075), liquid (0.073), contact (0.071)
E	Electron Emission and Plasma Technologies	248	Investigates electron emission, cathodes, diodes, and plasma-related phenomena. Covers ion and beam dynamics, thermionic emission, and materials like tungsten used in these applications.	emission (0.299), electron (0.277), diode (0.215), cathode (0.169), plasma (0.137), beam (0.135), ion (0.113), tungsten (0.113), current (0.111), thermionic (0.102)
F	Oceanic Surface Processes and Oil Slicks	198	Addresses ocean surface interactions, including Langmuir circulation, oil slicks, and wind-driven water movement. Covers marine and oceanic processes affecting water surfaces.	surface (0.244), sea (0.244), oil (0.219), langmuir circulation (0.171), ocean (0.146), water (0.138), marine (0.120), oceanic (0.098), wind (0.087), slick (0.073)
G	Liquid Spreading and Surface Tension Dynamics	132	Focuses on the dynamics of liquid spreading on surfaces, including surface tension effects, contact line motion, and the role of water in these processes.	liquid (0.259), spread (0.254), surface (0.214), dynamic (0.213), spreading (0.148), tension (0.147), water (0.142), lens (0.138), line (0.138), contact (0.138)
H	Optical Fiber Sensors and Coatings	108	Explores optical fiber-based sensing technologies, including long-period fiber gratings, refractive index variations, and deposition techniques for sensor applications.	long period (0.142), sensor (0.131), deposition (0.109), fiber grating (0.106), long-period fiber (0.091), index (0.077), coating (0.075), fibre (0.066), optical fiber (0.060), refractive (0.050)
I	Plasma-Assisted Nanomaterial Deposition	47	Investigates plasma-based chemical vapor deposition, nanoparticle formation, and charge interactions in silicon and graphene-based materials.	plasma (0.170), chemical vapor deposition (0.127), cluster (0.123), silicon (0.106), nanoparticles (0.106), charge (0.105), graphene (0.105), generation charged (0.085), thermal evaporation (0.085), particle (0.084)

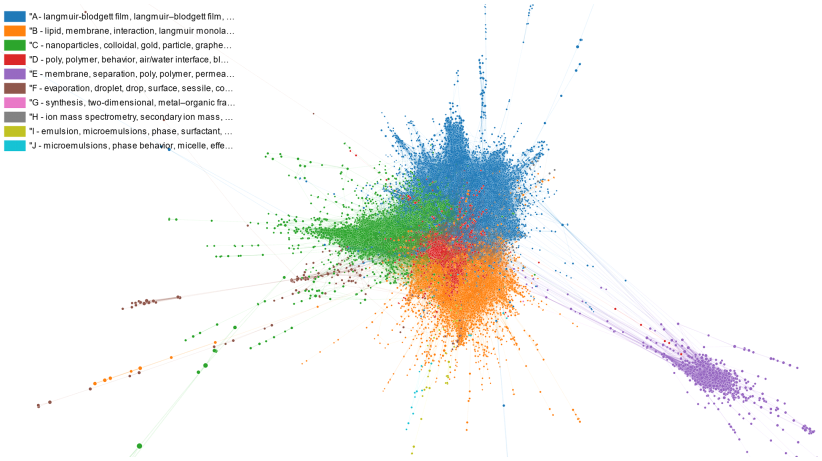

Figure S3: The citation network after the first filtering (made by only keeping clusters A and B from the original network).

Table S2: Clusters from the network in Figure S3, along with the number of papers, description and keywords.

Cluster	Name	Size	Description	Keywords and Score
**A**	Langmuir-Blodgett Films and Optical Properties	15,898	Focuses on Langmuir-Blodgett films, their optical characteristics, and molecular interactions. Topics include organic and polymeric materials, thin-film deposition, dye-based systems, and molecular complexes, highlighting their potential applications in optics and material sciences.	langmuir-blodgett film (0.095), langmuir–blodgett film (0.087), optical (0.081), derivative (0.079), organic (0.071), molecular (0.057), complex (0.055), dye (0.047), thin (0.037), polymer (0.033)
**B**	Lipid Membranes and Molecular Interactions	7,266	Explores the behavior of lipid membranes, their interactions at interfaces, and their phase properties. Includes studies on Langmuir monolayers, phospholipids, and surfactants, with a focus on model systems used to understand biological and synthetic membranes.	lipid (0.294), membrane (0.274), interaction (0.192), langmuir monolayers (0.173), interface (0.147), phase (0.127), model (0.127), phospholipid (0.104), langmuir monolayer (0.104), surfactant (0.092)
**C**	Nanoparticles and Colloidal Systems	4,069	Covers nanoparticles, colloidal systems, and nanostructured materials such as gold nanoparticles, graphene, and carbon-based materials. Topics include particle fabrication, oxide systems, and array formation, emphasizing their structural and functional properties.	nanoparticles (0.122), colloidal (0.074), gold (0.059), particle (0.057), graphene (0.055), nanoparticle (0.053), array (0.053), oxide (0.051), carbon (0.049), fabrication (0.045)
**D**	Polymers at Interfaces	927	Examines the behavior of polymers at the air/water interface, including block copolymers and amphiphilic molecules. Discusses polymer brushes, diblock copolymers, and surface interactions, contributing to the understanding of self-assembly and interfacial phenomena.	poly (0.433), polymer (0.349), behavior (0.200), air/water interface (0.197), block copolymer (0.196), surface (0.193), amphiphilic (0.183), diblock copolymer (0.150), brush (0.148), /i (0.100)
**E**	Membrane-Based Separation Processes	621	Focuses on polymeric membranes and their role in separation technologies. Topics include permeation, diffusion, gas transport, sorption, and water filtration, with an emphasis on the influence of polymer properties on membrane performance.	membrane (0.117), separation (0.050), poly (0.045), polymer (0.033), permeation (0.030), diffusion (0.030), gas transport (0.029), effect (0.027), sorption transport (0.027), water (0.026)
**F**	Droplet Evaporation and Surface Interactions	221	Studies evaporation dynamics, droplet behavior, and molecular distillation. Includes sessile droplet interactions, surface effects, bubble dynamics, and computational simulations, contributing to the understanding of fluid behavior on surfaces.	evaporation (0.159), droplet (0.137), drop (0.089), surface (0.072), sessile (0.072), contact (0.059), bubble (0.053), molecular distillation (0.050), line (0.043), simulation (0.041)
**G**	Synthesis of Two-Dimensional and MOFs	113	Investigates the synthesis of two-dimensional materials, metal-organic frameworks (MOFs), and covalent organic frameworks (COFs). Topics include thin-film fabrication, polymer integration, and the structural complexity of these advanced materials.	synthesis (0.223), two-dimensional (0.149), metal–organic framework (0.118), covalent organic framework (0.108), complex (0.099), 2D (0.094), metal-organic framework (0.089), polymer (0.075), thin film (0.073), metal (0.065)
**H**	Mass Spectrometry and Molecular Analysis	102	Explores mass spectrometry techniques, particularly secondary ion mass spectrometry (SIMS) and time-of-flight SIMS (TOF-SIMS). Covers applications in molecular analysis, depth profiling, and imaging for organic and inorganic systems.	ion mass spectrometry (0.269), secondary ion mass (0.145), analysis (0.127), molecular (0.104), time-of-flight secondary ion (0.097), depth profiling (0.093), TOF-SIMS (0.090), imaging (0.090), organic (0.074), c (0.073)
**I**	Emulsions, Microemulsions, and Surfactant Behavior	40	Examines emulsions and microemulsions, including phase behavior, surfactant interactions, and nonionic systems. Covers kinetics, foam formation, and microemulsion stability in various applications.	emulsion (0.183), microemulsions (0.143), phase (0.124), surfactant (0.088), nonionic (0.087), kinetics (0.082), interaction (0.080), microemulsion system (0.079), foam (0.076), formation (0.071)
**J**	Surfactant Systems and Solubilization	20	Focuses on surfactant-based systems, including micelles, solubilization mechanisms, and phase behavior. Discusses water-oil interactions, trisiloxane surfactants, and mixed reverse systems, highlighting their roles in colloidal and interfacial chemistry.	surfactant (0.143), microemulsions (0.115), solubilization (0.076), phase behavior (0.075), micelle (0.072), effect (0.070), water (0.054), trisiloxane (0.048), oil (0.047), mixed reverse (0.047)

Table S3: Clusters from the final network (made by only keeping clusters **A**, **B** and **C** from the intermediary network), seen in Figure 3, along with the number of papers, description and keywords.

Cluster	Name	Size	Description	Keywords and Scores
A	Langmuir-Blodgett Films and Polymer-Based Optical Materials	16077	This cluster focuses on Langmuir-Blodgett films, a method for depositing molecular layers onto surfaces, with emphasis on polymers and organic thin films. Topics include their optical properties, molecular structures, and potential applications in dye-based and polymeric systems.	langmuir-blodgett film (0.1083), langmuir–blodgett film (0.1064), polymer (0.0878), optical (0.0870), thin (0.0751), organic (0.0645), molecular (0.0634), derivative (0.0510), dye (0.0393), poly (0.0362)
B	Lipid Membranes and Molecular Interactions	6214	This cluster examines lipid membranes, their interactions, and the role of Langmuir monolayers in studying membrane behavior. It covers topics such as membrane-phase behavior, phospholipids, surfactants, and interfacial properties relevant to biological and model membrane systems.	lipid (0.1697), membrane (0.1445), interaction (0.0753), langmuir monolayers (0.0651), interface (0.0575), model (0.0546), phase (0.0493), phospholipid (0.0392), langmuir monolayer (0.0341), surfactant (0.0301)
C	Nanoparticles and Colloidal Systems	3845	This cluster explores nanoparticles and colloidal materials, including their synthesis, structural properties, and functional applications. Key topics include gold and carbon-based nanomaterials, graphene, nanotubes, and oxide-based nanostructures, with emphasis on their assembly and behavior in arrays.	nanoparticles (0.1282), colloidal (0.0784), particle (0.0618), graphene (0.0581), gold (0.0571), array (0.0551), nanoparticle (0.0546), carbon (0.0499), oxide (0.0478), nanotube (0.0451)
D	Crystallization and Nucleation Processes	732	This cluster focuses on crystallization mechanisms, nucleation processes, and the formation of crystalline structures. Topics include calcium carbonate formation, biomimetic crystallization, template-directed growth, and studies on specific crystalline phases such as calcite and oxalate.	crystal (0.1106), crystallization (0.0498), growth (0.0449), nucleation (0.0412), calcium carbonate (0.0340), calcite (0.0323), template (0.0321), monolayers (0.0307), oxalate (0.0298), biomimetic (0.0293)
E	Protein Films and Biomolecular Crystallization	365	This cluster covers protein-based Langmuir-Blodgett films and biomolecular crystallization, particularly in membrane-associated proteins like bacteriorhodopsin and cytochrome. Additional topics include photosynthetic reaction centers, retinal-binding proteins, and structural analysis techniques such as X-ray crystallography.	protein (0.1190), bacteriorhodopsin (0.1056), cytochrome (0.0952), langmuir-blodgett film (0.0830), purple membrane (0.0805), retinal (0.0532), photosynthetic reaction center (0.0532), crystallization (0.0468), x-ray (0.0435)

S5 References used to generate each paper (corresponding references in the generated paper).

Survey paper	References used to prompt the model
Strategy 1 - Paper 1	[6–10]
Strategy 1 - Paper 2	[6–26]
Strategy 1 - Paper 3	[6–8, 10–26]
Strategy 2 - Paper 1	[6–143]
Strategy 2 - Paper 2	[6–26, 28–143]

Table S4: Primary sources input for the model for each generated survey paper.

S6 First Paper generated using Strategy 1 (5 input papers)

Langmuir and Langmuir-Blodgett Films: A Comprehensive Survey of Principles, Techniques, Applications, and Future Perspectives

April 23, 2025

Contents

1 Introduction 3

2 Fundamentals of Langmuir and Langmuir-Blodgett Films 6
2.1 Langmuir Monolayers: Formation and Properties 6
2.2 Langmuir-Blodgett Transfer: Techniques and Parameters . . . 11
2.3 Materials for Langmuir and Langmuir-Blodgett Films 13

3 Advanced LB Film Fabrication Techniques 16
3.1 High-Temperature Langmuir-Blodgett Method 18
3.2 Vortex-Langmuir-Blodgett Method 20
3.3 Liquid-Liquid Interfacial Nanoarchitectonics 22

4 Layer-by-Layer (LbL) Assembly: Advances and Extensions 25
4.1 DNA-Directed LbL Assembly 26
4.2 LbL Assembly with External Manipulation 27
4.3 One-Pot Self-Assembly Process 28
4.4 Incorporation of Functional Units into LbL Structures 29

5 **Characterization Techniques for Langmuir and Langmuir-Blodgett Films** **30**
5.1 Surface Manometry and Rheology 31
5.2 Microscopic Techniques 35
5.3 Spectroscopic Techniques 36
5.4 Structural Analysis Techniques 38

6 **Applications of Langmuir and Langmuir-Blodgett Films** **41**
6.1 Sensors 41
6.2 Molecular and Organic Electronics 44
6.3 Energy Storage 45
6.4 Biomedical Applications 46

7 **Challenges and Future Directions** **47**
7.1 Addressing Current Limitations 49
7.2 Overcoming Challenges through Innovation 51
7.3 Emerging Trends and Opportunities 52
7.4 Future Research Perspectives 54

8 **Conclusion** **56**

Abstract

Langmuir and Langmuir-Blodgett (LB) technology provides a powerful methodology for fabricating highly ordered, ultra-thin films with molecular-level precision. This survey presents a comprehensive overview of the field, encompassing both Langmuir films formed at the air-water interface and Langmuir-Blodgett films transferred onto solid substrates. We trace the historical development and elucidate the fundamental principles governing the self-assembly of amphiphilic molecules, monolayer phase behavior characterized by surface pressure-area isotherms, and the mechanics of film transfer using the Langmuir trough. The review covers advancements in fabrication techniques and the expanding range of materials utilized beyond traditional amphiphiles, including polymers, nanoparticles, 2D materials, and complex biomolecules. Key characterization methods essential for probing film structure, morphology, and properties at the interface and on substrates are discussed. Furthermore, we highlight the diverse and evolving applications of Langmuir and LB films, with particular emphasis on recent progress in gas sensors, electrochemical devices, and biomimetic

systems, often within the conceptual framework of nanoarchitectonics. The survey also critically addresses persistent challenges, such as film stability and scalability, and discusses future perspectives, underscoring the enduring significance of LB technology for fundamental research and the development of advanced functional materials and devices where precise molecular arrangement is paramount.

1 Introduction

Langmuir-Blodgett (LB) film technology represents a cornerstone technique in materials science and nanotechnology for the fabrication of highly ordered, ultra-thin films with molecular-level precision [1]. This method involves the controlled transfer of a monomolecular layer, known as a Langmuir film, formed at a gas-liquid interface (typically air-water), onto a solid substrate. The resulting LB films possess unique structural characteristics, including controllable thickness down to the nanometer scale and highly anisotropic lamellar arrangements, making them invaluable tools for molecular engineering and the development of functional surfaces and devices [2, 3]. The ability to precisely manipulate molecular architecture at interfaces has positioned LB technology as a critical enabler across diverse fields, including optics, electrochemistry, sensor technology, biomimicry, and nanotechnology [4]. Its significance lies in providing a pathway to construct complex supramolecular systems by assembling molecules with specific functions in a predetermined manner, offering unparalleled control compared to many other thin-film deposition techniques [5, 6]. Both the Langmuir films residing at the interface and the transferred Langmuir-Blodgett films on solid supports offer unique platforms for fundamental studies and technological applications [7].

The historical roots of this technology extend back over a century, originating from early observations of oil spreading on water, famously investigated by Benjamin Franklin. However, the scientific foundation was laid by Agnes Pockels , who developed rudimentary methods for studying surface films in the late 19th century. Irving Langmuir significantly advanced the field in the early 20th century by introducing the Langmuir trough, a device equipped with a movable barrier to compress the surface film, allowing for the systematic study of monomolecular layers of amphiphilic molecules at the air-water interface and the measurement of surface pressure-area isotherms. This work established the fundamental principles of Langmuir film formation

and behaviour. Subsequently, Katharine Blodgett expanded upon Langmuir's work by demonstrating the sequential transfer of these monolayers onto solid substrates, creating multilayer structures known as Langmuir-Blodgett films [2, 8, 9]. This breakthrough opened the door to constructing well-defined, multi-component thin films. In the latter half of the 20th century, researchers like Hans Kuhn [6,14-16] further explored the potential of LB films for building complex, functional molecular assemblies, envisioning applications in molecular electronics and energy transfer systems. The establishment of the International Conference on Organized Molecular Films (ICOMF) series further stimulated research and development in the field, fostering collaboration and innovation. Despite the emergence of alternative techniques like self-assembled monolayers (SAMs) and layer-by-layer (LbL) assembly [5, 10, 11], LB technology retains its importance, particularly for applications demanding exquisite control over molecular order and film architecture in both Langmuir and Langmuir-Blodgett formats [1, 7, 3, 6].

Understanding the fundamental principles established by these pioneers is crucial to appreciating the capabilities of LB technology. The core principle underlying both Langmuir and LB films is the self-assembly of amphiphilic molecules at an interface [2]. These molecules typically possess a hydrophilic (water-attracting) head group and a hydrophobic (water-repelling) tail. When dissolved in a volatile, water-immiscible organic solvent and spread onto an aqueous subphase surface within a Langmuir trough, the solvent evaporates, leaving the amphiphilic molecules oriented at the air-water interface, with their hydrophilic heads immersed in the water and hydrophobic tails directed towards the air [12, 13]. Initially, at low surface densities, the molecules behave like a two-dimensional gas. As the available surface area is reduced by moving a barrier across the trough, the molecules are compressed, passing through various two-dimensional phases (such as gas (G), liquid-expanded (LE), liquid-condensed (LC), and solid (S)) analogous to three-dimensional states of matter [2, 14]. Controlling these phases via surface pressure adjustments is key to manipulating molecular packing density, orientation, and ultimately, the physicochemical properties of the resulting Langmuir film . This compression process allows for the formation of a densely packed, ordered monolayer (Langmuir film) at a specific surface pressure, which is monitored using a Wilhelmy plate or Langmuir balance. The sophisticated control achievable at the air-water interface even allows for complex fabrication and patterning, as illustrated conceptually in Figure 2, hinting at advanced possibilities beyond simple monolayer formation.

The transfer of this organized Langmuir monolayer onto a solid substrate is typically achieved by vertically dipping the substrate through the monolayer into the subphase and withdrawing it, creating an LB film [3, 2, 1]. During withdrawal (or immersion, depending on the substrate and molecular properties), the monolayer adheres to the substrate surface. Repeating this dipping cycle allows for the deposition of multiple layers, building up LB multilayers with controlled thickness and structure (e.g., X-, Y-, or Z-type deposition depending on transfer during upstroke, downstroke, or both). An alternative transfer method, Langmuir-Schaefer (LS) deposition, involves horizontally touching the substrate to the monolayer. The primary advantages of the LB technique stem from this controlled process: unparalleled precision in film thickness (controllable down to a single molecular layer, typically a few nanometers), the ability to create highly ordered and oriented molecular arrangements, and the versatility to incorporate a wide range of materials, including lipids, polymers, proteins, nanoparticles, and other nanomaterials, often in complex hetero-structures [1, 10, 6].

This survey aims to provide a comprehensive overview of Langmuir and Langmuir-Blodgett film technology, encompassing its fundamental principles, advanced fabrication techniques, characterization methods, and diverse applications. We will delve into the physicochemical phenomena governing monolayer formation and stability at the air-water interface (Langmuir films), explore variations and advancements in deposition methodologies for creating LB films, and discuss the complementary technique of Layer-by-Layer (LbL) assembly [5, 15]. A significant portion will be dedicated to the characterization techniques employed to probe the structure, morphology, and properties of these ultra-thin films, both at the interface and on solid supports [2]. Furthermore, the survey will highlight recent progress and key applications of LB films, with a particular focus on their use in gas sensors [16, 17], electrochemical devices (including batteries) [18, 19], and biomimetic systems, reflecting current research trends [20]. The objectives are to consolidate knowledge on the state-of-the-art in LB technology, critically evaluate its strengths and limitations in various application contexts, identify ongoing challenges, and discuss promising future directions and the evolving role of Langmuir and LB films in nanotechnology and materials science, particularly within the framework of nanoarchitectonics [Ariga2023]. The concept of nanoarchitectonics, visually outlined in Figure 1, emphasizes the deliberate construction of functional materials systems from nanoscale building blocks, a paradigm where the precise control offered by LB methods is particularly relevant [4,

7, 21].

The structure of this survey is organized to guide the reader from foundational concepts to contemporary research frontiers. Following this introduction (Section 1), Section 2 will elaborate on the Fundamentals of Langmuir and Langmuir-Blodgett Films, detailing the behaviour of molecules at interfaces and the thermodynamics of monolayer formation. Section 3 will explore Advanced LB Film Fabrication Techniques, discussing modifications and novel approaches that extend the capabilities of the traditional method [1, 4]. Section 4 will cover Layer-by-Layer (LbL) Assembly, examining its relationship with LB techniques and recent advances [5, 15, 22]. Section 5 will focus on Characterization Techniques specifically relevant to Langmuir monolayers and LB films. Section 6 will showcase the diverse Applications of Langmuir and Langmuir-Blodgett Films, emphasizing recent developments in sensors, electrochemistry, and biomimetics [3, 23, 24]. Section 7 will address the current Challenges and Future Directions, outlining obstacles and potential growth areas for the field. Finally, Section 8 will provide concluding remarks, summarizing the key aspects discussed throughout the survey. This structure aims to provide a logical progression, building a thorough understanding of LB technology and its impact on modern science and engineering.

2 Fundamentals of Langmuir and Langmuir-Blodgett Films

The Langmuir-Blodgett (LB) technique provides a powerful methodology for constructing highly ordered ultrathin films with molecular-level precision [1, 3]. Its foundation lies in the controlled manipulation of insoluble amphiphilic molecules spread at an air-water interface, forming a Langmuir monolayer, which is subsequently transferred onto a solid substrate [2, 25]. Understanding the principles governing monolayer formation, its characteristic properties, the transfer mechanisms, and the diverse range of materials amenable to this technique is crucial for harnessing its full potential in materials science, nanotechnology, and beyond [4, 7].

2.1 Langmuir Monolayers: Formation and Properties

The formation of a stable Langmuir monolayer hinges on the unique properties of amphiphilic molecules. These molecules possess distinct hydrophilic

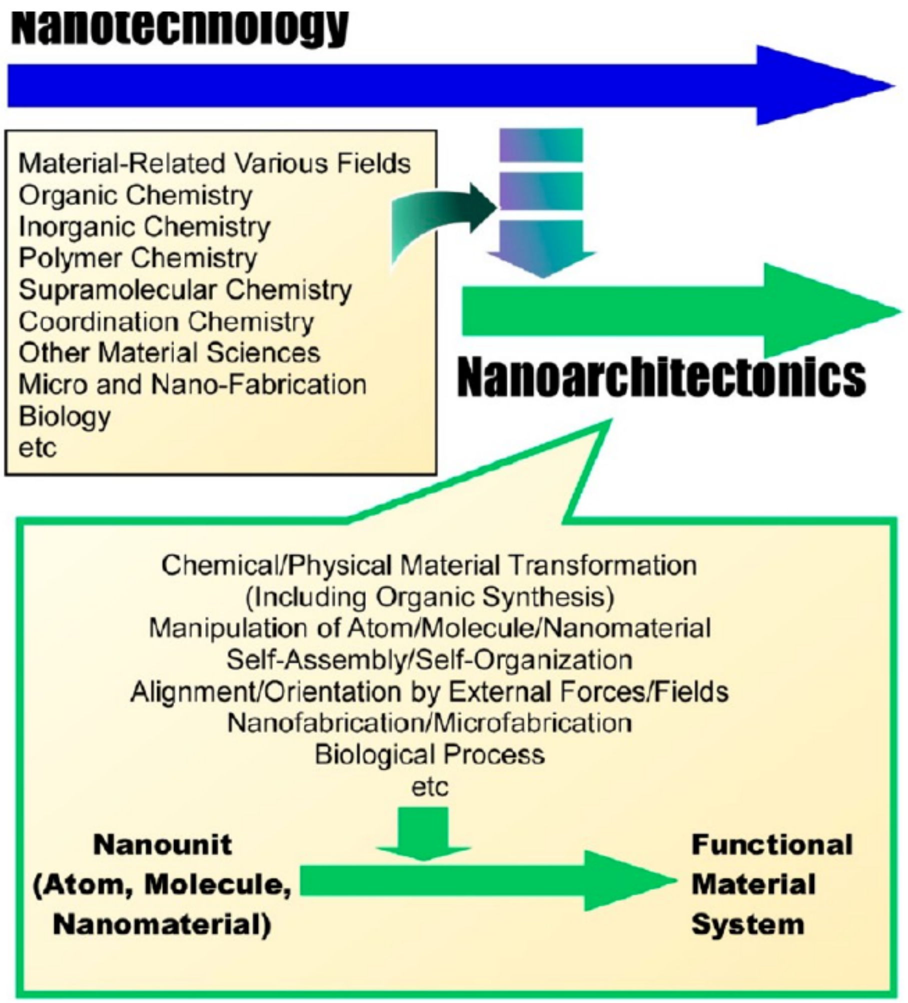

Figure 1: Outline of nanoarchitectonics concept. Adapted from Ariga, K., 2023 [6].

(water-loving) and hydrophobic (water-repelling) moieties. Classic examples include fatty acids, which have a polar carboxyl head group and a long nonpolar alkyl chain, and phospholipids, central components of biological membranes. To form a monolayer, these amphiphiles are first dissolved in a volatile organic solvent that readily spreads on the water surface. This requires the solvent to have a positive spreading coefficient, ensuring spontaneous coverage of the available water surface area. A small volume of this solution is carefully deposited onto the pristine surface of an aqueous sub-

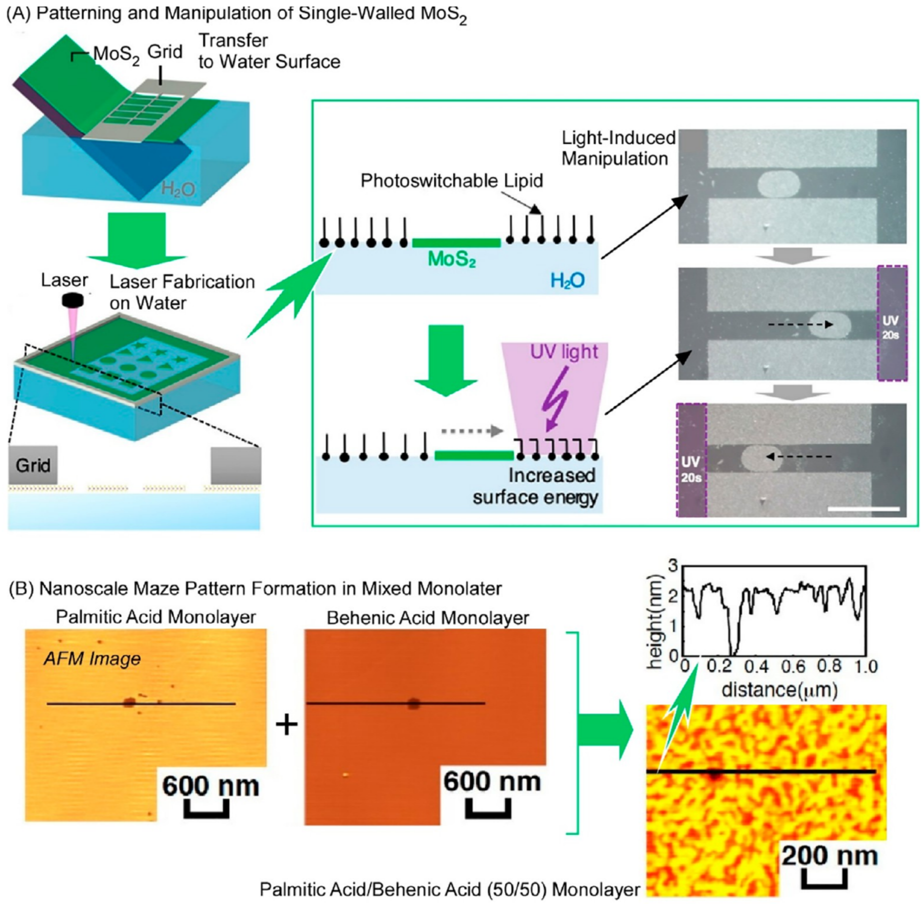

Figure 2: Fabrication and patterning at the air—water interface. (A) Laser-induced structural fabrication of single-walled MoS, nanosheets for controls of mechanical motion of floating MoS, piece. (B) Spontaneous formation of two-dimensional maze pattern in mixed monolayer morphologies of palmitic acid (C,) and behenic acid (C,). Adapted from Ariga, K., 2023 [6].

phase contained within a specialized apparatus known as a Langmuir trough.

As the volatile solvent evaporates, the amphiphilic molecules remain at the air-water interface [26, 13]. Due to their dual nature, they spontaneously orient themselves: the hydrophilic head groups immerse into the aqueous subphase, anchored by favorable interactions like dipole-dipole forces or hydrogen bonding [27, 28, 29], while the hydrophobic tails project upwards into the air, minimizing unfavorable contact with water [30]. Initially, if

the surface area available per molecule is large, the molecules are sparsely distributed and move relatively freely, behaving akin to a two-dimensional gas.

The Langmuir trough is equipped with one or more movable barriers that allow for the controlled reduction of the surface area available to the monolayer. As the barriers compress the film laterally, the molecules are forced closer together, increasing the surface density and leading to interactions between the hydrophobic tails (e.g., van der Waals forces) [31]. This compression process is monitored by measuring the surface pressure (), which is defined as the reduction in surface tension of the pure subphase () caused by the presence of the monolayer (= -). Surface pressure is typically measured using the Wilhelmy plate method, where the force exerted on a plate (often filter paper or platinum) partially immersed through the interface is measured by a sensitive balance.

Plotting the surface pressure () as a function of the area available per molecule (A) during compression at a constant temperature yields the surface pressure-area (-A) isotherm, illustrated schematically in Figure 3(a). This isotherm is a fundamental characteristic of a Langmuir monolayer, providing invaluable information about its phase behavior, compressibility, and stability. Several distinct phases can often be identified on the -A isotherm, analogous to the three-dimensional states of matter:

- **Gaseous (G) Phase:** At very large areas per molecule, interactions are minimal, and the monolayer behaves like a 2D gas. The surface pressure is very low.

- **Liquid-Expanded (LE) Phase:** As the area is reduced, molecules begin to interact more significantly, but the hydrophobic tails remain relatively disordered and mobile. The monolayer exhibits liquid-like fluidity, and the surface pressure starts to rise more steeply.

- **Liquid-Condensed (LC) Phase:** Further compression leads to a more ordered state where the alkyl chains become more closely packed and largely aligned, although often tilted with respect to the surface normal. This phase is less compressible than the LE phase, resulting in a steeper slope on the isotherm. The transition between LE and LC phases is often observed as a plateau or region of reduced slope, indicative of a first-order phase transition where the two phases coexist.

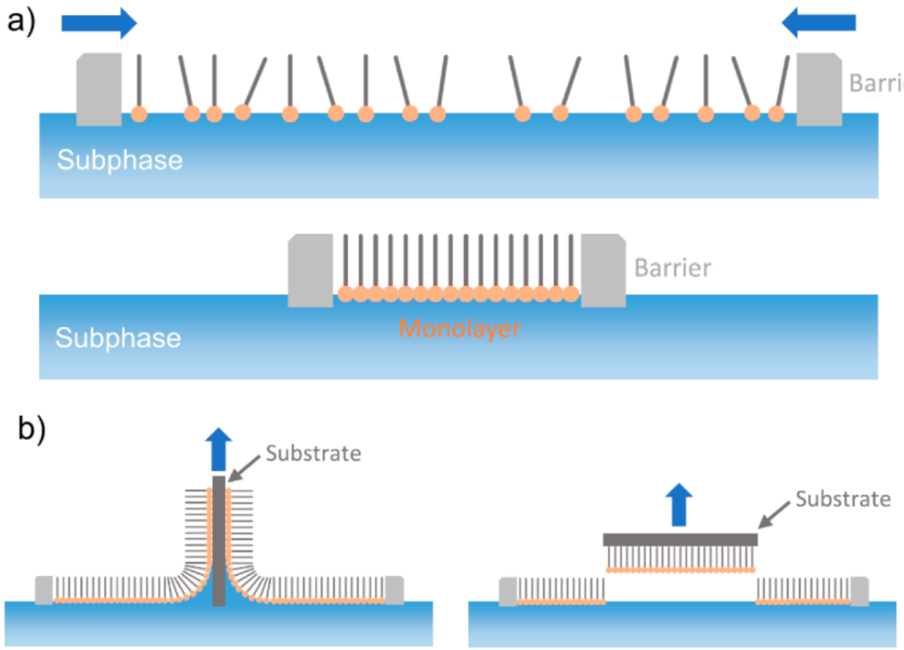

Figure 3: Schematic illustration of fundamental concepts in Langmuir and Langmuir-Blodgett films. (a) A typical surface pressure-area (Π-A) isotherm showing different monolayer phases (G: Gas, LE: Liquid-Expanded, LC: Liquid-Condensed, S: Solid) and collapse. (b) The vertical Langmuir-Blodgett (LB) transfer method, illustrating deposition during downstroke and upstroke for Y-type film formation. (c) The horizontal Langmuir-Schaefer (LS) transfer method. Adapted from Fang, C., et al., 2022 [18].

- **Solid (S) Phase:** At minimum area per molecule before collapse, the monolayer reaches a highly ordered, quasi-crystalline state. The molecules are tightly packed with maximal chain ordering, often oriented nearly perpendicular to the interface. This phase exhibits very low compressibility (very steep slope).

The compressibility of the monolayer in different phases can be quantified by the surface compressional modulus ($Cs^1 = -A\ (d/dA)T$), which is the inverse of the compressibility. Typical ranges of Cs^1, consistent with authoritative sources such as Oliveira et al. (2022), are used to classify the phases: 12.5-50 mN/m for LE, 100-250 mN/m for LC, and ¿250 mN/m for S.

If compression continues beyond the solid phase, the monolayer becomes

unstable and collapses. Collapse is an irreversible process where the 2D structure breaks down, and molecules are forced out of the interface into three-dimensional aggregates, bilayers, or multilayers, often leading to a plateau or decrease in surface pressure [32, 33]. The collapse pressure represents the maximum surface pressure the monolayer can withstand [34].

The precise nature of the -A isotherm and the phases observed depend critically on factors such as the molecular structure of the amphiphile (chain length, head group type, unsaturation), temperature (affecting molecular motion and interactions) [35], and the composition of the aqueous subphase (pH influencing head group ionization, presence of ions affecting intermolecular forces and stability). For instance, divalent cations in the subphase can bridge negatively charged head groups, significantly altering packing and stability. Studying monolayers composed of two or more components (mixed monolayers) provides insights into molecular interactions, miscibility, and phase separation within the film [36], often analyzed using the additivity principle for molecular area and thermodynamic excess functions.

2.2 Langmuir-Blodgett Transfer: Techniques and Parameters

While Langmuir monolayers confined to the air-water interface are valuable model systems, the Langmuir-Blodgett technique extends their utility by enabling their transfer onto solid supports, creating well-defined ultrathin films (LB films). This transfer process allows the preservation of the molecular order achieved at the interface within a solid-state structure. The transfer is typically carried out at a constant surface pressure, chosen from a stable region of the -A isotherm (usually in a condensed phase, below the collapse pressure) to ensure consistent film structure.

The most common transfer method is the vertical dipping technique, pioneered by Katharine Blodgett, depicted in Figure 3(b) [2, 37]. In this method, a solid substrate is vertically immersed into and/or withdrawn from the subphase through the compressed Langmuir monolayer.

- **Deposition Process:** During the upstroke (withdrawal), if the substrate surface is hydrophobic, the hydrophobic tails of the amphiphiles adhere to it, transferring one layer. During the downstroke (immersion), if the substrate surface is hydrophilic (or has become hydrophilic

after the first layer deposition), the hydrophilic head groups adhere, transferring another layer.

- **Film Types:** Repeated dipping cycles allow the buildup of multilayer films [38, 39]. The structure depends on whether transfer occurs on the upstroke, downstroke, or both. Y-type deposition, where transfer occurs during both immersion and withdrawal, is the most common and typically results in a centrosymmetric head-to-head and tail-to-tail arrangement. X-type (transfer only on downstroke) and Z-type (transfer only on upstroke) films are non-centrosymmetric and less common.

- **Transfer Ratio (TR):** The quality of the transfer is assessed by the transfer ratio, defined as the ratio of the decrease in the monolayer area on the trough (maintained at constant pressure by the barriers) to the area of the substrate coated during a stroke. A TR close to unity indicates successful and uniform deposition. Deviations from unity can signal incomplete transfer or monolayer reorganization during dipping.

An alternative method is the horizontal lifting technique, also known as the Langmuir-Schaefer (LS) method, shown in Figure 3(c). Here, the substrate is brought into contact horizontally with the monolayer surface. The film adheres to the substrate, which is then lifted away. This technique is particularly useful for transferring rigid monolayers that might be disrupted by vertical dipping or when only a single layer deposition is desired. As noted by Oliveira et al. (2022) and Fang et al. (2022), LS deposition typically transfers the monolayer such that the orientation at the air-water interface is preserved relative to the substrate (e.g., hydrophobic tails facing away from the substrate after horizontal contact and lifting) [40, 41]. Multilayers can also be built up by repeating the LS process, sometimes involving drying steps between depositions. Combinations of LB and LS methods can be employed to create complex architectures, such as supported lipid bilayers used as cell membrane models [42, 43].

The success and quality of both LB and LS transfer depend critically on several experimental parameters:

- **Surface Pressure:** The chosen deposition pressure influences the packing density and order of the transferred film [2]. It must be carefully selected based on the -A isotherm for optimal stability and structure.

- **Dipping/Lifting Speed:** The speed at which the substrate moves through or contacts the interface must be controlled. Typical speeds range from 1 to 10 mm/min. Too fast a speed can lead to defects and poor transfer, while too slow a speed can be impractical and allow for monolayer relaxation or contamination.

- **Subphase Composition:** Temperature, pH, and ionic strength of the subphase affect monolayer properties (stability, charge, packing) and its interaction with the substrate. Specific ions can sometimes mediate adhesion between the monolayer and the substrate.

- **Substrate Properties:** The nature of the solid support (e.g., glass, silicon, quartz, mica, gold) is crucial. Its surface energy (hydrophilicity/hydrophobicity), cleanliness, smoothness, and chemical reactivity determine the adhesion and quality of the first layer and subsequent layers. Substrates often require rigorous cleaning and sometimes surface modification (e.g., silanization) to promote uniform deposition.

- **Molecular Structure:** The chemical nature of the amphiphile dictates its intermolecular forces, rigidity, and interaction with the substrate, directly impacting transferability.

- **Environmental Conditions:** Factors like ambient temperature, humidity, and vibrational stability must be controlled to ensure reproducible results. Drying conditions between layers in multilayer deposition can also influence film structure.

Careful optimization of these parameters is essential for fabricating high-quality LB films with desired thickness, uniformity, and molecular organization [1, 3]. Difficulties in transfer, such as poor adhesion or removal of previously deposited layers (negative transfer ratio), often require systematic tuning of these conditions.

2.3 Materials for Langmuir and Langmuir-Blodgett Films

A significant strength of the Langmuir-Blodgett technique is its versatility regarding the types of materials that can be organized into ultrathin films. While initially developed for classical amphiphiles like fatty acids, the method has been successfully extended to a wide array of substances, including polymers, nanoparticles, and complex biomolecules. The fundamental

requirement is the ability of the material, either inherently or through modification or co-spreading, to form a stable film at the air-water interface that can withstand transfer.

Classical Amphiphiles: These remain cornerstone materials for fundamental studies and certain applications.

- *Fatty Acids and Alcohols:* Molecules like stearic acid (CH(CH)COOH) are well-characterized, exhibiting distinct phases [34] and providing simple models for studying monolayer behavior and transfer mechanisms.

- *Phospholipids:* Lipids such as dipalmitoylphosphatidylcholine (DPPC) [44, 45], dioleoylphosphatidylcholine (DOPC), dipalmitoylphosphatidic acid (DPPA) [46], and sphingomyelin are crucial for creating biomimetic membranes [47]. Their phase behavior (e.g., LE-LC coexistence in DPPC) is physiologically relevant [48]. Mixed monolayers, often including cholesterol [49, 50, 51], are used to mimic the complexity of cell membranes, allowing investigation of lipid-lipid and lipid-protein interactions [52, 53, 54, 55].

These materials typically form well-defined monolayers and transfer readily under appropriate conditions, but the resulting films can sometimes lack mechanical or long-term stability.

Polymers: Polymeric materials offer enhanced mechanical robustness, stability, and diverse functionalities compared to small-molecule amphiphiles. LB films of polymers can be fabricated using several approaches:

- *Preformed Polymers:* Amphiphilic polymers or polymers soluble in spreading solvents can be directly spread at the interface [10]. Examples include poly(methyl methacrylate) (PMMA), polyaniline (PANI), poly(vinylidene fluoride) (PVDF) [56], and various copolymers designed with hydrophilic and hydrophobic blocks. Semiconducting polymers (e.g., polythiophenes like PBTTT) have been processed via LB for electronic applications [57, 58]. Achieving highly oriented films of such polymers can be challenging due to aggregation and rigidity, sometimes necessitating specialized techniques like high-temperature spreading onto non-aqueous subphases (e.g., ethylene glycol at 100°C or higher), as detailed by Oliveira et al. (2022) and Song et al. (2024) [59, 40].

- *In situ Polymerization:* Monomeric precursors are spread to form a Langmuir monolayer, and polymerization is initiated at the air-water interface (e.g., via UV irradiation or chemical initiators in the subphase) before or during transfer. Alternatively, monomers can be transferred as LB films and polymerized subsequently on the substrate. This approach can yield highly stable, cross-linked films.

Polymeric LB films find use in sensors [60], dielectric layers, and protective coatings.

Nanoparticles and Nanomaterials: The integration of nanomaterials into LB films has opened up vast possibilities for creating functional nanostructured surfaces [1, 7, 6, 61].

- *Metallic Nanoparticles:* Gold (AuNPs) and silver (AgNPs) nanoparticles are widely used for their plasmonic properties (SERS, sensors) [62, 63, 64] and catalytic activity. They are often functionalized with ligands to aid spreading or co-spread with traditional amphiphiles.

- *Semiconductor Nanomaterials:* Quantum dots (e.g., CdS, CdSe) and nanowires (e.g., ZnO) can be assembled using LB techniques for applications in optoelectronics, photovoltaics, and sensing.

- *Carbon Nanomaterials:* Fullerenes (e.g., C), carbon nanotubes (CNTs), graphene, and graphene oxide (GO) have been incorporated into LB films for electronic devices [65], transparent conductors, sensors [66], and composites [11]. Spreading these materials often requires surface functionalization, dispersion aids (e.g., surfactants), or co-spreading with matrix molecules like polymers or lipids. Achieving uniform, well-ordered films can be challenging due to the inherent tendency of many nanomaterials to aggregate and difficulties in achieving stable spreading at the interface, issues discussed by Oliveira et al. (2022) and Fang et al. (2022).

- *Other Nanomaterials:* Inorganic materials like silica particles, clay nanosheets, black phosphorus nanosheets [60], metal-organic frameworks (MOFs) [67], and covalent organic frameworks (COFs) [68] have also been successfully deposited using LB or LS methods, enabling applications in catalysis, sensing, separation, and energy storage [19, 69].

The LB method allows for precise control over the density and arrangement of nanoparticles on a substrate, which is often difficult to achieve with other deposition techniques.

Biomolecules: The LB technique provides a gentle method for organizing biological macromolecules at interfaces and on surfaces, relevant for biosensors, biocompatible coatings, and fundamental studies of biological interactions [70, 71].

- *Proteins and Peptides:* While challenging due to potential denaturation at the interface, some proteins and peptides can be spread or adsorbed onto lipid monolayers [53, 72]. This is used to study protein-membrane interactions [73, 74] or create bioactive surfaces [22].
- *DNA:* Nucleic acids can be complexed with cationic lipids or polymers to facilitate spreading and transfer, enabling studies of DNA condensation or fabrication of DNA-based sensors [75].

Maintaining the biological activity and native conformation of biomolecules during the LB process remains a key challenge.

The choice of material profoundly influences the properties and potential applications of the resulting LB film. Fatty acid films might serve as simple insulating layers, while phospholipid films model cell membranes [76]. Polymer films offer robustness for sensors or coatings. Nanoparticle-containing films exhibit unique electronic, optical, or catalytic properties [77, 78]. Biomolecular films are targeted towards biointerfaces and sensing [20, 79, 80]. Often, hybrid films combining different material classes (e.g., lipid-nanoparticle, polymer-CNT) are fabricated to achieve synergistic functionalities [81, 66, 69]. However, extending the LB technique to non-traditional, often non-amphiphilic or rigid materials frequently requires innovative strategies to overcome challenges in spreading, monolayer stability, and transfer efficiency.

3 Advanced LB Film Fabrication Techniques

While the fundamental principles of the Langmuir-Blodgett technique, involving the compression and transfer of molecular monolayers from an air-water interface, have remained largely unchanged for over a century, the continuous drive to expand its applicability to a wider range of materials and

Table 1: Overview of Materials Used in Langmuir and Langmuir-Blodgett Films.

**Material Class**	**Examples**	**Key Properties / Characteristics**	**Common Application Areas**
Classical Amphiphiles	Fatty acids (Stearic Acid), Phospholipids (DPPC, DOPC), Cholesterol	Well-defined phases, Biomimetic nature (lipids), Relatively easy transfer	Fundamental studies, Biomembrane models, Basic insulating layers
Polymers	PMMA, PANI, PVDF, PBTTT, Polymerizable monomers	Mechanical robustness, Stability, Diverse functionalities (conducting, dielectric)	Sensors, Electronics (dielectrics, semiconductors), Protective coatings
Nanoparticles / Nanomaterials	AuNPs, AgNPs, QDs (CdS), CNTs, Graphene/GO, MOFs, ZnO NWs	Unique optical/electronic properties, High surface area, Catalytic activity, Porosity (MOFs)	Plasmonics (SERS), Sensors, Electronics, Catalysis, Energy storage, Composites
Biomolecules	Proteins, Peptides, DNA	Biological activity, Specific recognition capabilities	Biosensors, Biocompatible coatings, Drug delivery studies, Biointerface research
Hybrid Materials	Lipid-Nanoparticle, Polymer-CNT, MOF-Polymer	Combined functionalities, Synergistic effects	Advanced sensors, Multifunctional devices, Targeted drug delivery

functionalities has spurred significant innovation [3, 1]. The limitations inherent in the conventional approach, particularly concerning materials with high cohesive forces, low solubility, or complex aggregation behaviours at room temperature, have necessitated the development of advanced fabrication methodologies. These advanced techniques often involve deliberate deviations from established protocols, such as operating under non-standard temperature conditions [59, 40], employing dynamic subphase environments,

or utilizing alternative interfacial systems beyond the traditional air-water boundary [82, 4]. Such common-sense breaking strategies have opened new avenues for creating highly ordered, functional thin films from previously inaccessible molecular and nanomaterial building blocks, pushing the frontiers of two-dimensional nanoarchitectonics [61, 83, 84]. This section delves into some prominent examples of these advanced LB methods, highlighting their underlying principles, unique capabilities, and potential applications.

3.1 High-Temperature Langmuir-Blodgett Method

A significant challenge in conventional LB film fabrication arises when dealing with materials possessing strong intermolecular interactions, such as many functional conjugated polymers or highly cohesive inorganic precursors. These materials often exhibit poor spreading characteristics and tend to form uncontrolled aggregates on the water surface at ambient temperatures, hindering the formation of uniform, well-ordered monolayers [2, 1]. The limited thermal energy available at room temperature is insufficient to overcome these strong cohesive forces or to provide the necessary molecular mobility for rearrangement into a stable, condensed film upon compression. Furthermore, the relatively high vapor pressure of water near room temperature can interfere with film stability and transfer processes, especially during prolonged experiments.

To address these limitations, the high-temperature Langmuir-Blodgett (HT-LB) method has emerged as a powerful technique [40, 59]. This approach involves performing the monolayer formation and compression steps at significantly elevated temperatures, often ranging from 80°C up to 200°C or even higher. Operating at such temperatures necessitates replacing the conventional water subphase with liquids possessing much lower vapor pressures and higher boiling points to prevent rapid evaporation and ensure stable experimental conditions.

Ethylene glycol, with its high boiling point (197°C) and low vapor pressure, was one of the first successful alternative subphases demonstrated for HT-LB deposition of polymer semiconductors like poly[2,5-bis(3-tetradecylthiophen-2-yl)thieno(3,2-b)-thiophene] (PBTTT) at 100°C. More recently, ionic liquids, known for their negligible vapor pressure even at high temperatures, have been employed, further extending the accessible temperature range (e.g., up to 140°C for donor-acceptor copolymers).

The enhanced thermal energy in the HT-LB method promotes better

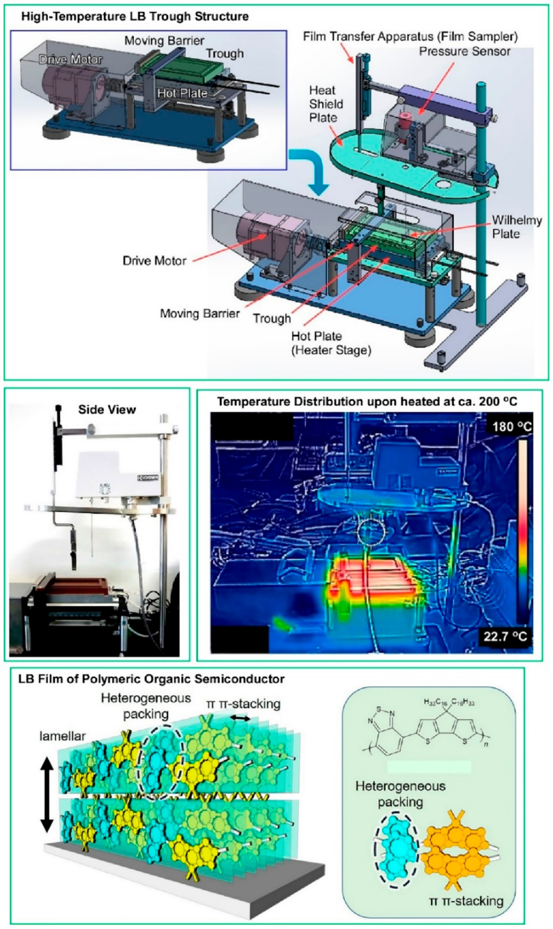

Figure 4: Ultrahigh-temperature LB machine up to at least about 200 °C equipped with a Wilhelmy plate connected to a pressure sensor, automatic membrane compression, and Langmuir—Schaefer type membrane transfer: (top) trough design; (middle) trough photo and thermal distributions; (bottom) expected images of highly oriented LB films of polymeric semiconductor molecules. Adapted from Ariga, K., 2023 [6].

spreading of cohesive materials, counteracts aggregation, and facilitates molecular rearrangement during compression, leading to more stable and highly ordered packing [59, 40]. For polymer semiconductors, this often results in semicrystalline films with significant uniaxial alignment, enhancing anisotropic

properties like charge transport and yielding higher mobilities compared to conventional films.

Implementing HT-LB requires specialized troughs with robust, uniform heating stages and modified Wilhelmy balances with heat-shielded sensors for accurate surface pressure measurement, as depicted in Figure 4. Automated compression and transfer systems (often Langmuir-Schaefer type) are crucial for reproducibility. The conceptual illustration in Figure 5 clarifies this working principle, showing how heating the subphase enables the formation and compression of an ordered monolayer from initially cohesive molecules, leading to highly oriented films upon transfer [59, 40]. Such automated HT-LB systems facilitate uniform, large-area film creation, broadening the scope of LB deposition to challenging functional polymers and other materials, making the technique potentially scalable [3, 1].

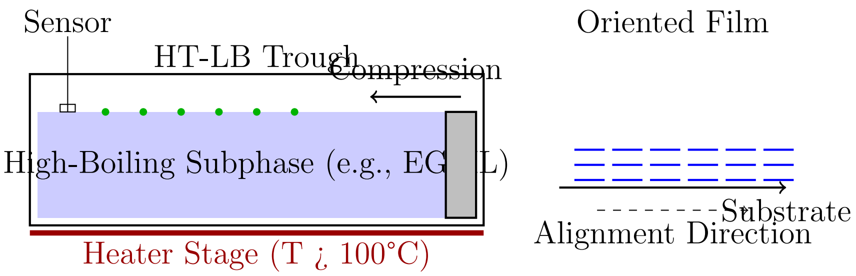

Figure 5: Conceptual illustration of the High-Temperature Langmuir-Blodgett (HT-LB) method. A trough containing a high-boiling point subphase is heated, allowing for the spreading and compression of highly cohesive molecules (green circles) into an ordered monolayer. Subsequent transfer can yield highly oriented films (right), beneficial for applications like organic electronics.

3.2 Vortex-Langmuir-Blodgett Method

Conventional wisdom in LB technology dictates that the subphase surface must be kept perfectly quiescent, free from vibrations or external disturbances, to allow for the delicate self-assembly process and prevent disruption of the fragile monolayer. However, challenging this paradigm, the vortex-Langmuir-Blodgett (Vortex-LB) method intentionally introduces controlled fluid dynamics into the system [4]. In this technique, the subphase within

the trough is subjected to stirring or rotation, generating a stable vortex flow on the liquid surface [85]. This dynamic environment fundamentally alters the forces acting on the molecules or nanomaterials spread at the interface.

The rationale behind the Vortex-LB method is that the shear forces and controlled flow patterns generated by the vortex can assist in the organization and assembly of materials that are otherwise difficult to process using standard static compression. For molecules that might aggregate undesirably or fail to form a stable, continuous film under simple compression, the dynamic forces within the vortex can provide subtle intermolecular guidance, potentially breaking up ill-formed aggregates and promoting the formation of uniform, large-area assemblies [85, 86]. The flow field can influence molecular orientation and packing in ways not achievable through quasi-static compression alone. This principle has been effectively demonstrated in the fabrication of novel carbon nanomaterials [86, 87].

A notable application of the Vortex-LB method involves the fabrication of ultrathin carbon nanosheets from non-traditional precursors like carbon nanoring molecules (e.g., cycloparaphenylenes), as conceptually depicted in Figure 6 [86]. Spreading such molecules onto a rotating water subphase results in the formation of a uniform molecular assembly across the surface, driven by the interplay between the vortex flow [85] and intermolecular forces. This assembled layer can then be transferred onto a solid substrate using standard LB or LS techniques [1, 3]. Subsequent high-temperature treatment (carbonization) under an inert atmosphere (like nitrogen, which can also lead to doping if nitrogen-containing precursors or atmospheres are used) converts the molecular assembly into a nanometer-thick, uniform carbon nanosheet [86, 88]. For instance, using pyridine during the spreading of specific nanorings on the vortex surface has led to nitrogen-doped carbon nanosheets exhibiting enhanced electrical conductivity [86, 89].

The Vortex-LB method represents a significant departure from traditional LB protocols, transforming a potential source of disruption (subphase movement) into a tool for controlled assembly [85, 86]. It expands the repertoire of LB techniques, offering a pathway to fabricate thin films from complex molecules or nanomaterials that may not respond well to conventional monolayer manipulation.

The resulting films, such as conductive carbon nanosheets, hold promise for applications in areas like catalysis (e.g., fuel cells), energy storage, and electronics. Further exploration of the interplay between fluid dynamics, molecular structure, and interfacial assembly in the Vortex-LB system may

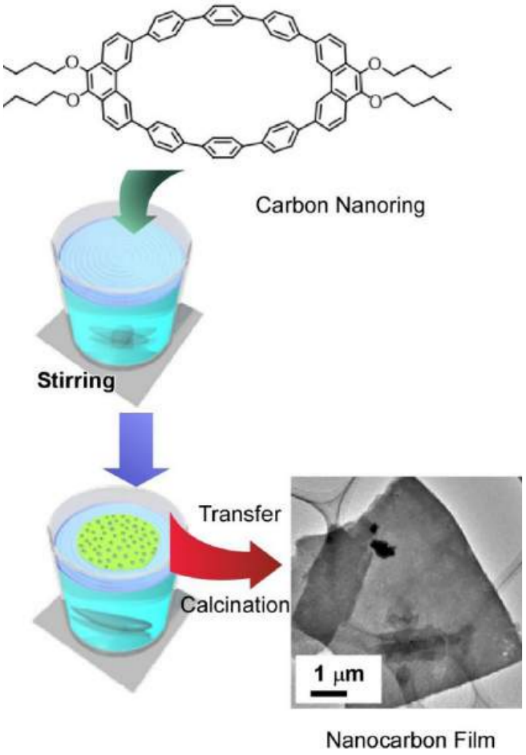

Figure 6: Vortex LB technique for fabrication of carbon nanosheets with carbon nanoring molecules. Adapted from Oliveira, O., et al., 2022 [90].

unlock new possibilities for nanoarchitectonics.

3.3 Liquid-Liquid Interfacial Nanoarchitectonics

The vast majority of Langmuir and Langmuir-Blodgett studies utilize the air-water interface as the platform for molecular assembly [13, 12]. This interface provides a sharp discontinuity in polarity and phase, driving amphiphilic molecules to orient and assemble. However, the interface between two immiscible liquids offers an alternative and equally versatile environment for two-dimensional nanoarchitectonics [4, 82]. Replacing the air phase with a second liquid phase (typically an organic solvent immiscible with the aqueous subphase, such as oil, alkanes, or aromatic solvents like xylene) creates a liquid-liquid interface with distinct characteristics, including different interfacial tension, dielectric properties, and potential for specific solute par-

titioning compared to the air-water interface.

Nanoarchitectonics at liquid-liquid interfaces can proceed via several mechanisms. One established approach is liquid-liquid interfacial precipitation (LLIP), where reactants dissolved in the two separate immiscible phases diffuse towards the interface and react, leading to the precipitation and crystallization of products directly at the boundary [4]. This has been used to grow crystals of various functional materials [91, 92]. Beyond crystallization, supramolecular assembly strategies can be effectively employed at liquid-liquid interfaces [93]. Molecules designed or found to possess amphiphilicity relative to the two liquid phases can spontaneously accumulate and organize at the interface, driven by a combination of forces including hydrophobic/hydrophilic interactions relative to the two phases, van der Waals forces, and potentially π-stacking for aromatic systems [94, 14].

A compelling example of supramolecular assembly at a liquid-liquid interface is the formation of large-area nanofilms from fullerene derivatives [4]. Fullerene (C60) itself is hydrophobic, but it can be rendered interfacially active. For instance, reacting C60 with ethylenediamine can produce partially derivatized, amphiphilic adducts. When introduced into a biphasic system, such as water and xylene, these adducts preferentially migrate to the interface and self-assemble into stable, ordered structures, often resembling bilayers or closely packed monolayers, depending on the specific derivative and conditions [95]. To generate macroscopic films, this assembly process can be scaled up by creating a large interfacial area, for example, by forming an emulsion of the two liquids and allowing the assembled film to coalesce as the emulsion slowly breaks or by carefully spreading the components [91, 92].

These interfacially assembled fullerene derivative films can serve as precursors for novel two-dimensional carbon materials [95]. By transferring the assembled film onto a substrate and subjecting it to high-temperature pyrolysis in an inert atmosphere, the molecular structure is carbonized, yielding a continuous, large-area carbon nanosheet, termed 'fullerphene' (a graphene-like material derived from fullerene assembly) [88, 86]. If nitrogen-containing species like ethylenediamine are involved in the initial assembly, the resulting fullerphene becomes nitrogen-doped, which can enhance its electronic and chemical properties [89].

A unique structural feature of fullerphene arises from the inherent packing of the spherical fullerene precursors. Even in a close-packed assembly, interstitial voids exist between the spheres. Upon carbonization, remnants

of these voids are preserved within the carbon nanosheet structure, creating intrinsic sub-nanometer pores or "subnanospaces" [88]. These subnanospaces can act as highly selective molecular recognition sites [96, 97]. Fullerphene films used in quartz crystal microbalance (QCM) sensors have demonstrated remarkable selectivity for small analyte molecules whose size precisely matches these subnanospaces. For example, such sensors exhibit exceptionally high sensitivity and selectivity towards formic acid vapor, effectively distinguishing it from acetic acid, which differs by only a single carbon atom [88]. This highlights the potential of liquid-liquid interfacial nanoarchitectonics not only to create large-area 2D materials [4, 82] but also to engineer materials with precisely controlled porosity at the sub-nanometer level for advanced sensing and separation applications. This approach complements air-water interfacial methods, providing access to different assembly pathways and material structures.

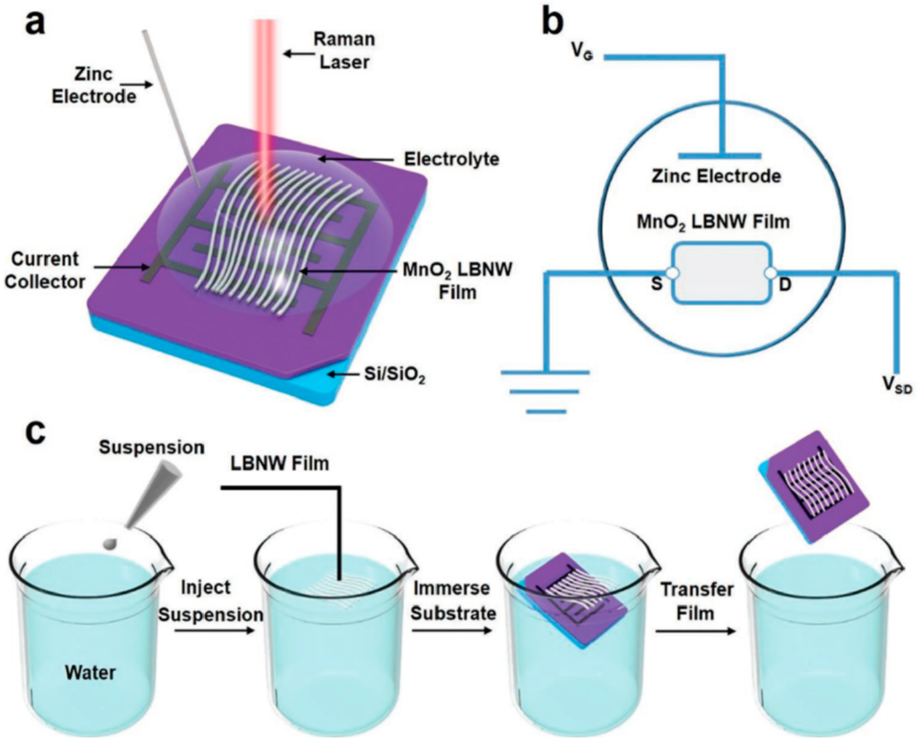

Figure 7: Schematic demonstration of the configuration of a Langmuir-Blodgett Nanowire (LBNW) device and depiction of the LBNW film formation and transfer processes. Adapted from Fang, C., et al., 2022 [18].

It should be noted that Figure 7, while potentially related to advanced LB film fabrication involving nanomaterials, illustrates the specific preparation and transfer of nanowire films for device construction, which is distinct from the liquid-liquid interfacial assembly mechanism discussed in this subsection. Its relevance might be more direct in sections discussing LB films of nanomaterials or specific device applications.

4 Layer-by-Layer (LbL) Assembly: Advances and Extensions

The Layer-by-Layer (LbL) assembly technique emerged approximately three decades ago as a powerful method for constructing nanostructured thin films [22]. While often compared to the Langmuir-Blodgett (LB) technique [98, 7], LbL offers distinct advantages and disadvantages. Both methods allow for the creation of organized multilayer structures with precisely controlled thickness determined by the number of deposition cycles. However, LbL typically utilizes water-soluble materials interacting through forces like electrostatic attraction or hydrogen bonding [11, 38, 39], whereas LB relies on insoluble amphiphiles spread at the air-water interface [58]. While LbL may not achieve the same degree of molecular orientation and structural finesse as LB films, it offers advantages in procedural simplicity, the wide variety of applicable materials (including functional polymers, proteins, biomolecules, quantum materials, colloidal nanoparticles, MOFs, COFs, and nanosheets) [10, 99, 100], and film robustness. These attributes make LbL particularly attractive for potential industrial applications and have spurred significant research into extending its capabilities beyond conventional approaches [5, 101, 102]. Initially termed self-assembly or electrostatic self-assembly, the LbL designation became prevalent as the technique expanded to encompass driving forces beyond electrostatics, such as hydrogen bonding. The versatility and simplicity of LbL have made it an essential technology for nanoarchitectonics [84, 4], facilitating the creation of thin-film materials with diverse functionalities. Recent advancements focus on enhancing specificity, incorporating external control mechanisms, simplifying fabrication, and integrating complex functional units, pushing the boundaries of what can be achieved with this versatile technique.

4.1 DNA-Directed LbL Assembly

Traditional LbL assembly often relies on relatively non-specific interactions, such as electrostatic attraction between oppositely charged polyelectrolytes or hydrogen bonding. While effective for building multilayer structures, these methods offer limited control over the precise placement and recognition between specific components within the layers. To achieve a higher degree of specificity and programmability in LbL nanoarchitectonics, researchers have turned to biomolecular recognition motifs, particularly DNA hybridization. DNA-directed LbL assembly utilizes the highly specific base-pairing interactions between complementary DNA strands to guide the sequential deposition of materials. Instead of relying on generic forces, components functionalized with specific single-stranded DNA sequences are assembled layer by layer through the hybridization of complementary linker DNA chains.

An illustrative example involves the creation of multicomponent plasmonic nanoparticle membranes using DNA-directed LbL nanoarchitectonics, as depicted in Figure 9A [103]. Gold nanospheres and gold nanorods, each possessing distinct surface plasmon resonance properties, were functionalized with different complementary linker DNA strands. By alternately exposing a substrate to solutions containing these DNA-grafted nanoparticles, specific LbL assembly was achieved based solely on DNA hybridization. This approach allows for the precise positioning of different nanoparticle types at specific layers within the film. Furthermore, the distinct optical properties and differential susceptibility of the gold nanospheres and nanorods to external stimuli, such as photothermal effects, enable advanced functionalities. For instance, direct photothermal writing can be employed to pattern the assembled film laterally by selectively disrupting the DNA linkages at desired locations, also illustrated conceptually in Figure 9A [103]. By strategically placing nanoparticles with different sensitivities at different vertical positions within the LbL stack, photopatterning can be achieved in selective domains with precisely controlled vertical depths [103]. This modular approach allows designing functional units (e.g., nanoparticles of varying shapes/compositions) with distinct properties and stimulus sensitivities, enabling their programmed stacking into well-defined, complex organizations. This high degree of control opens possibilities for creating sophisticated materials, potentially leading to adaptive micromachines capable of sensing and responding to multiple types of external stimuli in a pre-programmed manner, mimicking the complexity found in biological systems but using synthetic

components guided by the precise language of DNA base-pairing [104, 105, 106].

4.2 LbL Assembly with External Manipulation

While LbL assembly inherently builds structures layer by layer in the vertical direction, controlling the lateral organization or inducing anisotropy within the layers often requires additional strategies. Conventional dipping or spraying methods typically result in isotropic films unless specific molecular interactions or template effects dictate otherwise. Introducing external physical manipulation during the LbL deposition process represents an innovative approach to impart structural order and anisotropy into the films. One such method involves incorporating a simple brushing operation during LbL deposition, as schematically shown in Figure 9B.

Boulmedais and co-workers demonstrated this concept by depositing LbL structures composed of hydrogen-bonded tannic acid and collagen using a manual brushing operation with a standard nylon paintbrush [107]. This seemingly simple modification to the deposition protocol resulted in a hierarchical structure significantly different from films prepared by conventional dipping. Analysis revealed that collagen fibers, with diameters around 60 nm, were preferentially oriented along the direction of brushing. This induced anisotropy at the nanoscale had profound effects on biological interactions at the macroscale. When human myoblasts were cultured on these brushed LbL films, with collagen as the outermost layer, the cells aligned themselves along the brushing direction (Figure 9B). Furthermore, after extended culture in a differentiation medium, these aligned myoblasts differentiated into long, aligned myotubes. This enhanced differentiation was attributed to the combination of the oriented collagen fiber structure providing topographical cues and the presence of tannic acid, known to promote differentiation. This example highlights how a straightforward mechanical operation, integrated into the LbL process, can effectively nanoarchitectonically control the structure of the film and subsequently direct cell behavior, including alignment, differentiation, and organization [108, 71, 84]. Such techniques hold promise for developing anisotropic tissues under in vitro conditions, mimicking the complexity of native tissues more closely than isotropic scaffolds. Potential applications include anisotropic tissue regeneration, wound healing therapies, disease modeling, and platforms for pharmacological studies where tissue architecture plays a critical role [22]. This approach demonstrates that extend-

ing LbL beyond simple immersion can unlock new possibilities for controlling film structure and function through external physical cues.

4.3 One-Pot Self-Assembly Process

The conventional LbL method is characterized by its stepwise nature, involving alternating exposure of a substrate to solutions containing the interacting components, typically with intermediate washing steps. While highly versatile, this sequential process can be time-consuming and may not be optimal for all material systems or desired architectures. Breaking from this convention, alternative strategies have been developed that achieve LbL-like structures through different assembly mechanisms, sometimes even in a single process step, often referred to as "one-pot" methods. These approaches leverage specific chemical interactions and self-assembly principles to spontaneously form alternating layered structures without the need for iterative deposition.

Yamauchi and co-workers reported such a method for creating nanometer-thick alternating layer thin films via a one-pot self-assembly process, deviating significantly from the standard stepwise LbL protocol, illustrated in Figure 10. They demonstrated the synthesis of LbL structures comprising graphene oxide (GO) nanosheets and nickel carbide nanosheets [100]. The process begins with GO nanosheets acting as templates or nucleation sites for the two-dimensional crystal growth of Ni-based cyano-bridged coordination polymer (CP) nanoflakes directly onto their surface. These CP-coated GO nanosheets then spontaneously self-assemble in solution or suspension, driven by interactions that favor stacking into a lamellar organization consisting of alternating layers of GO nanosheets and CP nanoflakes [100]. This self-assembly process generates the layered hetero-structure directly, bypassing the need for sequential deposition onto a substrate. Subsequently, this precursor LbL structure is subjected to heat treatment under an inert atmosphere (nitrogen). During pyrolysis, the CP nanoflakes are converted into nickel carbide (Ni3C) nanosheets. Crucially, the intercalated GO nanosheet layers play a vital role by inhibiting three-dimensional nucleation and random fusion of the metal components, thereby preserving the two-dimensional morphology of the carbide nanosheets derived from the CP flakes [100]. The resulting nanoarchitectonized hetero-LbL membrane composite, consisting of alternating GO and Ni3C layers, exhibited excellent electrocatalytic activity and superior durability for the oxygen reduction reaction (ORR). This strat-

egy, combining templated growth and spontaneous self-assembly, offers a potentially simpler and more efficient route to ordered LbL structures for certain material combinations [5, 101, 15]. It is envisioned that this nanoarchitectonics approach could be extended to fabricate a variety of other inorganic-organic hybrid materials with ordered LbL structures, potentially leading to new functional composites for catalysis, energy storage, and other applications [84, 109, 4].

4.4 Incorporation of Functional Units into LbL Structures

A major driving force in materials science is the integration of molecular-level functionality into macroscopic materials. LbL assembly provides a versatile platform for incorporating diverse components, and recent efforts have focused on embedding highly functional units, such as molecular machines, within LbL architectures. Molecular machines are molecules or molecular assemblies capable of performing machine-like actions, such as rotation or translation, in response to external stimuli (e.g., light, chemical changes, heat). Integrating these dynamic components into ordered LbL films could lead to materials with switchable properties and advanced functions.

Heinke and co-workers demonstrated the incorporation of molecular motors into an LbL structure by utilizing Metal-Organic Frameworks (MOFs), as shown in Figure 11 [110, 111]. They assembled a surface-mounted MOF (SURMOF) structure on a substrate using an LbL approach [112, 5]. The key innovation was the use of a specifically designed ligand containing a photoresponsive molecular motor moiety as a side chain. This ligand, along with metal ions (e.g., Cu(II)), was used in a stepwise LbL deposition process (liquid-phase epitaxy) to grow the MOF film layer by layer [112]. The resulting thin film structure is advantageous for operating photoresponsive molecular machines because it allows light penetration. Within the MOF structure, the molecular motor units were densely immobilized and tightly organized. Importantly, the MOF framework provided the necessary spatial arrangement and constraints: the stator part of the molecular motor was oriented perpendicular to the substrate surface, while the rotor part was situated within a pore large enough to permit light-induced molecular rotation [113]. This precise nanoarchitectonics allowed the researchers to trigger the rotational motion of the immobilized motors by switching between metastable

states using light irradiation. Crucially, this controlled molecular motion at the nanoscale was shown to influence macroscopic properties, specifically by controlling the adsorption and diffusion of guest molecules within the MOF pores [110]. This work demonstrates that molecular motors can be integrated into LbL-assembled structures while maintaining their functionality, provided their arrangement allows sufficient steric freedom for motion. Such systems represent a significant step towards the development of smart materials where molecular motion can be harnessed to control properties like membrane permeation for molecules and ions, selective catalysis, or information storage at the molecular level [96, 105]. The combination of LbL assembly for structural control and molecular machines for dynamic function opens exciting avenues for creating responsive and adaptive materials [114, 115, 116].

Further developments in MOF-based LbL assembly continue to refine the ability to create high-quality, functional LbL films incorporating complex units like MOFs and molecular machines [110]. Various advanced techniques are being explored, such as spray-based LbL liquid-phase epitaxy which allows for rapid deposition (Figure 8A) [112], solution atomic layer deposition (sALD) which offers excellent control over film thickness and uniformity at room temperature (Figure 8B) [117], and molecular layer deposition (MLD) which utilizes gas-phase precursors for stepwise, self-saturating growth of crystalline MOF films (Figure 8C) [118]. These methods address challenges like film uniformity, deposition speed, and precursor compatibility, expanding the scope of MOF LbL nanoarchitectonics [5, 101].

5 Characterization Techniques for Langmuir and Langmuir-Blodgett Films

Characterizing Langmuir monolayers at fluid interfaces and Langmuir-Blodgett (LB) films on solid substrates requires diverse techniques to probe their structure, morphology, composition, and properties across multiple length scales [2, 3, 119]. These techniques range from macroscopic measurements of interfacial thermodynamics and rheology to microscopic visualization and spectroscopic analysis, providing complementary information crucial for understanding film formation, organization, and function. Characterizing Langmuir monolayers *in situ* at the air-water interface is particularly challeng-

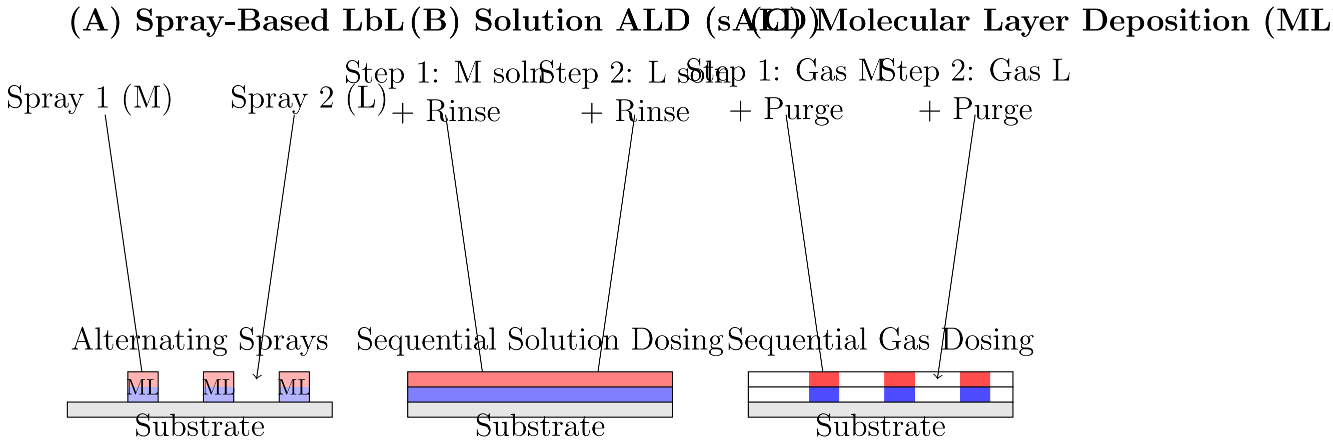

Figure 8: Schematic illustration of advanced LbL methods for MOF thin film fabrication. (A) Spray-based LbL using alternating sprays of precursor solutions. (B) Solution Atomic Layer Deposition (sALD) involving sequential immersion in precursor solutions with intermediate rinsing steps for high uniformity. (C) Molecular Layer Deposition (MLD) using sequential doses of gas-phase precursors with intermediate purging steps for crystalline film growth.

ing due to their ephemeral nature and monomolecular thickness, requiring surface-specific methods [13, 120]. Transferred LB films, being more stable and accessible, can be analyzed using a broader range of surface science techniques, similar to those employed for other ultrathin films.

5.1 Surface Manometry and Rheology

The fundamental characterization of Langmuir monolayers involves measuring the surface pressure (Π) as a function of the area available per molecule (A) [31]. Surface pressure is defined as the reduction in surface tension of the pure subphase (γ_0) caused by the presence of the monolayer (γ), i.e., $\Pi = \gamma_0 - \gamma$. It is typically measured using the Wilhelmy plate method [121], where the force exerted on a plate (often filter paper or platinum) partially immersed through the interface is monitored by a sensitive balance. Assuming complete wetting (zero contact angle), the surface tension is directly proportional to the measured force per wetted length of the plate. By compressing the monolayer laterally using movable barriers within a Langmuir

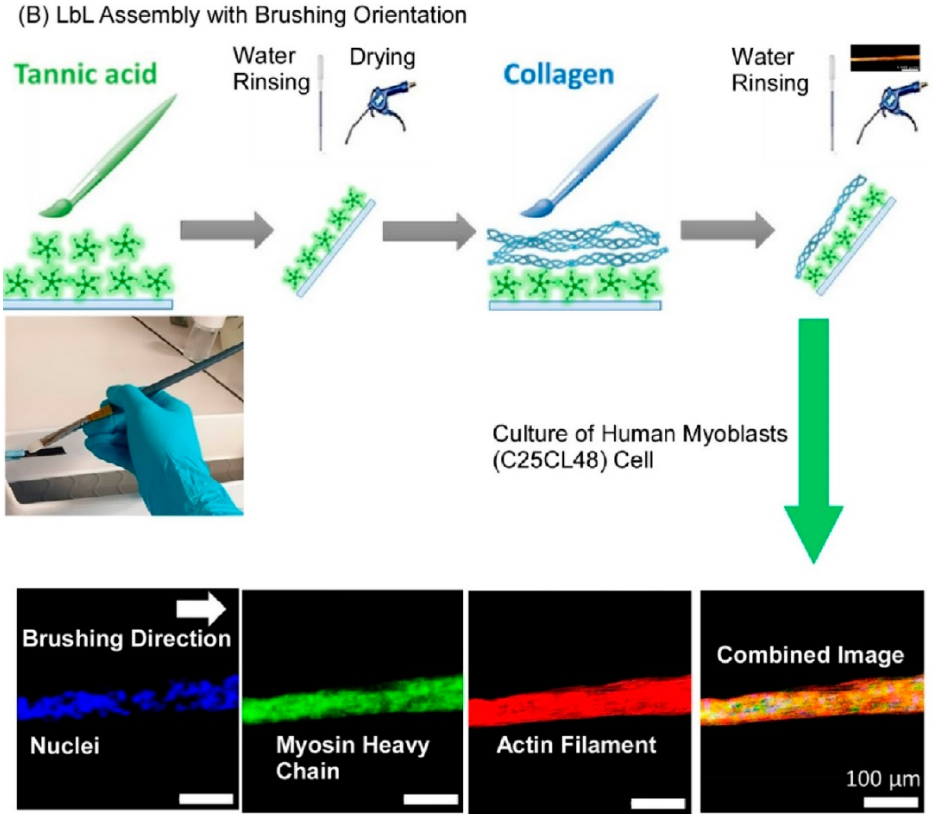

Figure 9: Patterning and orientation controls of LbL assembly. (A) Photopatterning of DNA-directed LbL assembly. Adapted from Ariga, K., 2023 [6].

trough, a surface pressure-area ($\Pi - A$) isotherm is recorded at constant temperature.

The $\Pi - A$ isotherm is analogous to a pressure-volume isotherm for a three-dimensional system and provides critical thermodynamic information about the monolayer's phase behavior and compressibility [35, 122]. Different phases can be identified based on the slope and features of the isotherm, including the gaseous (G) phase at large areas per molecule, the liquid-expanded (LE) phase, the liquid-condensed (LC) phase, and the solid (S) phase at higher pressures. Transitions between these phases often appear as kinks or plateau regions in the isotherm [48, 123]. The monolayer's resistance to compression is quantified by the surface compressional modulus

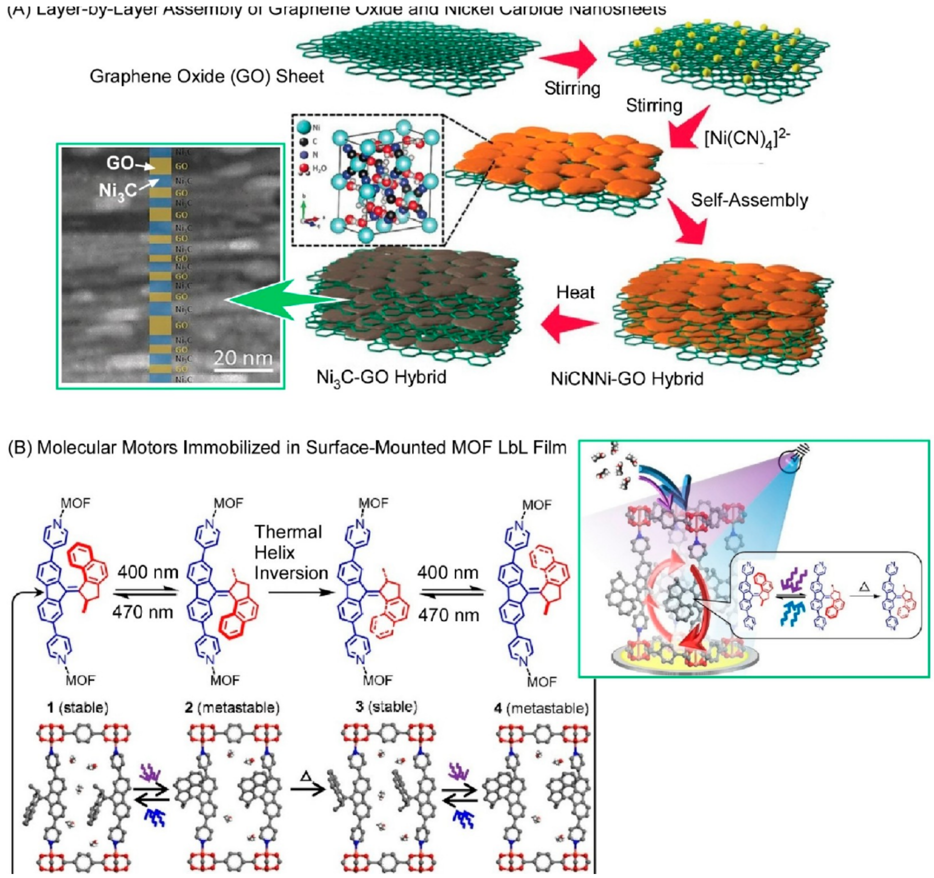

Figure 10: (A) Layer-by-Layer Assembly of Graphene Oxide and Nickel Carbide Nanosheets. Adapted from Ariga, K., 2023 [6].

(also known as the modulus of elasticity), defined as $K_s = -A(\partial\Pi/\partial A)_T$. This quantity, often denoted C_s^{-1} in the literature, represents the inverse of the surface compressibility $C_s = -(1/A)(\partial A/\partial\Pi)_T$. Its value varies significantly between different phases, being low in fluid phases (G, LE) and high in condensed phases (LC, S) [124]. The isotherm also allows for the determination of the limiting molecular area by extrapolating the steepest part of the curve (typically the LC or S phase) to zero pressure, providing insights into molecular packing. Furthermore, $\Pi - A$ isotherms are invaluable for studying interactions in mixed monolayers composed of two or more components [125, 54] and for monitoring the adsorption or penetration of molecules from the aqueous subphase into the monolayer [126, 127, 128]. The analysis of

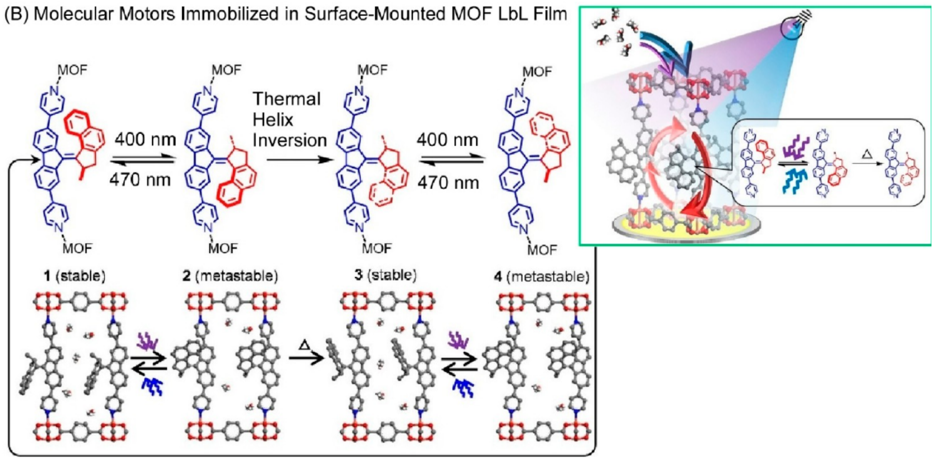

Figure 11: (B) Molecular motors immobilized in surface mounted MOF LbL film. Adapted from Ariga, K., 2023 [6].

surface pressure changes upon injection of substances into the subphase can yield parameters like the maximum insertion pressure (MIP), indicating the monolayer pressure above which insertion is thermodynamically unfavorable [129].

Beyond thermodynamics, the mechanical properties of Langmuir monolayers can be investigated using surface rheology techniques [130, 131]. These methods probe the monolayer's response to applied stress or strain, revealing its viscoelastic characteristics [132]. Dilatational rheology involves oscillating the area of the monolayer (e.g., using oscillating barriers) and measuring the corresponding change in surface pressure. The dynamic dilatational modulus (E) can be calculated, which is generally a complex quantity, $E = E' + iE''$. The real part, E', represents the elastic (storage) modulus, while the imaginary part, E'', represents the viscous (loss) modulus. The phase angle between the applied strain and the measured stress provides information about the relative contributions of elastic and viscous behavior. Shear rheology, often performed using a magnetic needle [133] or rotating ring geometry, measures the monolayer's resistance to shear deformation, providing the surface shear viscosity and shear modulus [134, 135]. Rheological properties are crucial for understanding monolayer stability, flow behavior during transfer, and interactions within the film, particularly for complex systems like polymer or nanoparticle monolayers [136].

5.2 Microscopic Techniques

Visualizing the morphology and structure of Langmuir monolayers and LB films at the mesoscopic and microscopic scales provides direct evidence of domain formation, phase coexistence, defects, and overall film homogeneity [2, 9, 137]. Several microscopy techniques are employed for this purpose.

Brewster Angle Microscopy (BAM) is a powerful non-invasive optical technique for imaging Langmuir monolayers directly at the air-water interface (*in situ*) [138, 139]. It exploits the principle that p-polarized light incident at the Brewster angle (approximately 53° for the air-water interface) is not reflected from the pure water surface. However, the presence of a monolayer changes the local refractive index, leading to some reflectivity. The intensity of the reflected light is detected by a CCD camera, creating an image where variations in intensity correspond to differences in film thickness, density, or molecular orientation. BAM does not require any labeling probes and provides real-time visualization of monolayer structure during compression, revealing domain shapes, sizes, phase transitions (e.g., LE-LC coexistence), and the influence of subphase conditions or adsorbed species [140, 141]. The spatial resolution is typically in the micrometer range. Coupling BAM with ellipsometry allows for quantitative measurements of domain thickness and optical anisotropy [142].

Fluorescence Microscopy (FM) is another optical technique used for *in situ* monolayer visualization at the air-water interface [138, 143, 144]. It requires the addition of a small amount of fluorescent probe molecules to the monolayer. These probes preferentially partition into specific phases (e.g., more soluble in the LE phase than the LC phase) or orient differently depending on the local environment [26, 113]. Upon excitation with appropriate wavelength light, the emitted fluorescence is imaged. FM provides high contrast between different phases or domains, allowing detailed studies of phase separation, domain growth kinetics, and molecular organization [145]. A major limitation is the potential perturbation of the monolayer structure and properties by the fluorescent probe itself. Careful selection of probes and low concentrations are necessary to minimize artifacts.

Atomic Force Microscopy (AFM) has become an indispensable tool for characterizing the topography and nanomechanical properties of transferred LB films on solid substrates (*ex situ*) [42, 146]. AFM operates by scanning a sharp tip attached to a cantilever across the sample surface. Feedback mechanisms maintain either a constant force or constant height, allowing

the reconstruction of a high-resolution topographical map, often achieving sub-nanometer vertical resolution and nanometer lateral resolution. AFM can image LB films under ambient conditions or even in liquid environments. Beyond topography, AFM can operate in various modes to map other properties, such as friction (Lateral Force Microscopy), adhesion, and viscoelasticity (Force Modulation or Tapping Mode Phase Imaging). AFM has been used extensively to assess film uniformity, measure layer thickness, visualize molecular-scale ordering [36], identify defects, and study the structure of complex LB films containing nanoparticles or biomolecules. Furthermore, AFM tips can be functionalized to perform single-molecule force spectroscopy (SMFS), measuring interaction forces between the tip and specific molecules within the LB film [147].

Electron Microscopy techniques, namely Scanning Electron Microscopy (SEM) and Transmission Electron Microscopy (TEM), are powerful tools for high-resolution imaging. Unlike BAM and FM which probe the monolayer *in situ*, SEM and TEM are typically employed for *ex situ* analysis of the morphology and structure of LB films after transfer to a solid support. SEM provides high-resolution images of the surface morphology, revealing features like film continuity, cracks, aggregation [148], or the alignment of anisotropic components like nanowires or nanotubes within the film, often requiring conductive coatings. TEM offers even higher resolution and can provide information about the internal structure and crystallinity of the film components, especially for LB films incorporating nanoparticles or crystalline domains, usually requiring transfer onto specialized TEM grids. Both SEM and TEM provide valuable structural information complementary to AFM and optical microscopy, particularly for characterizing nanomaterial-based LB films.

5.3 Spectroscopic Techniques

Spectroscopic methods provide detailed information about the chemical composition [149], molecular orientation [150, 151], conformation [45], and electronic structure of Langmuir monolayers and LB films.

UV-Visible (UV-Vis) Absorption and Fluorescence Spectroscopy are used to study the electronic properties of chromophoric molecules within the films. For Langmuir monolayers, reflection spectroscopy or specialized trough setups are needed for *in situ* measurements. For transferred LB films, standard transmission or reflection measurements can be performed (*ex situ*).

UV-Vis spectra reveal information about electronic transitions, molecular aggregation (e.g., formation of H- or J-aggregates characterized by blue or red shifts in absorption bands, respectively) [152], and photochemical reactions. Fluorescence spectroscopy provides complementary information on emission properties, excited-state dynamics [26], and molecular environment. These techniques are particularly useful for characterizing LB films containing dyes [17, 63], conjugated polymers [57, 153], or quantum dots, and for applications in optical sensing or optoelectronics.

Vibrational Spectroscopies, including Infrared (IR) and Raman spectroscopy, probe the vibrational modes of molecules, offering fingerprints for chemical identification and sensitivity to molecular structure, conformation, orientation, and intermolecular interactions [154]. Fourier Transform Infrared (FTIR) spectroscopy is widely used. For Langmuir monolayers at the air-water interface, surface-specific IR techniques like Polarization-Modulated Infrared Reflection-Absorption Spectroscopy (PM-IRRAS) [155, 156, 157] or Grazing Incidence Reflection Absorption FTIR (GIRAS) are employed for *in situ* analysis. PM-IRRAS provides high sensitivity and allows for the subtraction of background signals (water vapor, bulk water) [158], yielding spectra primarily from the monolayer. Analysis of peak positions, intensities, and dichroism (using polarized IR light) provides information on the conformation of alkyl chains (e.g., ratio of trans to gauche conformers), hydration of polar headgroups, hydrogen bonding, and the average orientation of specific molecular groups relative to the surface normal [27]. For transferred LB films, standard transmission or Attenuated Total Reflectance (ATR-FTIR) [159] can be used for *ex situ* characterization. Sum-Frequency Generation (SFG) spectroscopy is another powerful surface-specific vibrational technique used for *in situ* studies of interfaces [150, 160]. It is inherently sensitive only to interfaces where inversion symmetry is broken, providing information similar to IR spectroscopy regarding molecular orientation and order but often with higher surface specificity. Raman spectroscopy offers complementary vibrational information, particularly for molecules with weak IR absorption or for studies in aqueous environments where water's IR absorption is strong [161]. Standard Raman is typically applied *ex situ* to transferred LB films. Surface-Enhanced Raman Scattering (SERS) can significantly enhance the Raman signal by adsorbing molecules onto or near plasmonic nanostructures (e.g., Au or Ag nanoparticles incorporated into LB films) [62, 63], enabling highly sensitive detection and characterization, often used in sensing applications with transferred films.

X-ray Photoelectron Spectroscopy (XPS), also known as Electron Spectroscopy for Chemical Analysis (ESCA), is a surface-sensitive technique used *ex situ* to determine the elemental composition and chemical states of atoms within the top few nanometers of transferred LB films [149, 162]. By irradiating the sample with X-rays, core-level electrons are emitted, and their kinetic energies are measured. The binding energies are characteristic of specific elements and their chemical environment (oxidation state, bonding partners). XPS provides quantitative elemental analysis and valuable information about surface chemistry, functionalization, and the composition of complex LB assemblies. Angle-resolved XPS (ARXPS) can provide non-destructive depth profiling information.

5.4 Structural Analysis Techniques

Determining the precise arrangement and packing of molecules within Langmuir monolayers and LB films requires techniques sensitive to structural order on the atomic and molecular scale [9, 8]. X-ray and neutron scattering methods are paramount for this purpose [137, 163].

X-ray Reflectivity (XRR) and Neutron Reflectivity (NR) are powerful techniques for probing the structure of thin films perpendicular to the surface plane, applicable both *in situ* (at liquid interfaces with specialized setups) and *ex situ* (on transferred films) [164]. They measure the intensity of specularly reflected X-rays or neutrons as a function of the incident angle (or momentum transfer, Q_z) [165, 166, 163]. The resulting reflectivity profile contains information about the film's total thickness, the thickness and density (electron density for XRR, scattering length density for NR) of individual layers within a multilayer structure, and the roughness of the interfaces. NR is particularly advantageous for studying organic or biological films, as the contrast between different components can be dramatically enhanced by selective deuteration (replacing hydrogen with deuterium), exploiting the large difference in neutron scattering lengths between H and D isotopes [167]. This allows, for instance, distinguishing hydrocarbon chains from headgroups or determining the location of water within the film structure.

Grazing Incidence X-ray Diffraction (GIXD) is the primary technique for determining the in-plane crystalline structure and molecular packing of Langmuir monolayers at the air-water interface (*in situ*) and in transferred LB films (*ex situ*) [137, 163]. X-rays are incident on the surface at a very small angle (below the critical angle for total external reflection), creating an

evanescent wave that propagates along the interface and probes the monolayer structure. If the molecules form a two-dimensional ordered lattice, diffraction peaks will be observed at specific in-plane scattering angles. The positions of these peaks provide information about the lattice parameters and symmetry of the 2D unit cell. The intensity distribution along the diffraction rods (out-of-plane scans) provides information about the molecular tilt angle, orientation, and conformational order within the unit cell, as well as the coherence length of the ordered domains. Synchrotron X-ray sources are often required for GIXD studies of monolayers due to the weak scattering signal, providing high flux and resolution [145, 165]. GIXD has been instrumental in identifying different condensed phases (LC, S) and characterizing the detailed molecular arrangement in numerous amphiphilic systems [168, 169].

Table 2 provides a summary of the main characterization techniques discussed [164, 170, 171, 172].

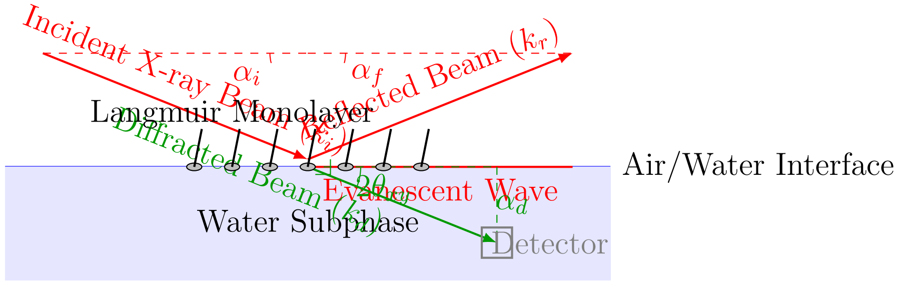

Figure 12: Schematic illustration of the Grazing Incidence X-ray Diffraction (GIXD) geometry for studying Langmuir monolayers at the air-water interface. The incident beam (k_i) strikes the surface at a shallow angle α_i. The specularly reflected beam (k_r) exits at $\alpha_f = \alpha_i$. An evanescent wave propagates along the interface, probing the monolayer. If the monolayer is ordered, it diffracts the X-rays. The in-plane component of the diffracted beam (k_d) is measured at an angle $2\theta_{xy}$ relative to the transmitted beam direction, and its out-of-plane angle is α_d.

Collectively, these diverse characterization techniques provide a comprehensive understanding of Langmuir and Langmuir-Blodgett films [25], from their macroscopic thermodynamic behavior at interfaces [46, 173, 50] to their microscopic structure, morphology, and chemical nature [9, 153]. The appropriate choice and combination of techniques depend on the specific system

Table 2: Summary of Key Characterization Techniques for Langmuir and Langmuir-Blodgett Films.

**Category**	**Technique**	**Information Obtained / Typical Application**
**Manometry & Rheology**	$\Pi - A$ Isotherms	Phase behavior, compressibility, molecular area, stability, intermolecular interactions, adsorption kinetics (*in situ* monolayers).
	Surface Rheology	Viscoelastic properties (elastic/loss moduli, viscosity), film fluidity, mechanical stability (*in situ* monolayers).
**Microscopy**	BAM	*In situ* morphology, domain structure, phase transitions, thickness/orientation variations (probe-free, monolayers).
	Fluorescence Microscopy	*In situ* morphology, phase coexistence, domain dynamics (requires fluorescent probe, monolayers).
	AFM	Topography (nm resolution), thickness, roughness, nanomechanical properties, friction, adhesion (*ex situ*, transferred films).
	SEM / TEM	Surface morphology, defects, alignment (SEM), internal structure, crystallinity (TEM) (*ex situ*, transferred films).
**Spectroscopy**	UV-Vis / Fluorescence	Electronic transitions, aggregation (H/J), chromophore orientation, sensing response (*in situ* reflection / *ex situ* transmission/reflection).
	IR (PM-IRRAS, ATR-FTIR)	Chemical composition, molecular conformation (trans/gauche), orientation, H-bonding, hydration (PM-IRRAS: *in situ* monolayers; ATR-FTIR: *ex situ* films).
	SFG	Surface-specific vibrational structure, molecular orientation at interfaces (*in situ* monolayers).
	Raman / SERS	Complementary vibrational information, enhanced sensitivity for sensing (SERS) (Raman: typically *ex situ* films; SERS: *ex situ* films).
	XPS	Elemental composition, chemical states, surface chemistry (top few nm) (*ex situ*, transferred films).
**Structural Analysis**	XRR / NR	Film thickness, layer structure, density profiles (electron/scattering

under investigation and the properties of interest, distinguishing between the analysis of dynamic monolayers at fluid interfaces and static films transferred onto solid supports [119].

6 Applications of Langmuir and Langmuir-Blodgett Films

Langmuir-Blodgett (LB) film technology, encompassing both the formation of ordered Langmuir monolayers at fluid interfaces and their subsequent transfer onto solid substrates as LB films, offers exceptional capacity for precise molecular assembly and control over film thickness and architecture [6, 7, 25]. This control has enabled applications across a diverse range of scientific and technological fields [3]. The ability to create highly ordered, ultra-thin films from a variety of materials—including small molecules, complex macromolecules like the bottlebrush polymers shown in Figure 15, nanoparticles, and biomolecules—makes LB technology a versatile platform for developing advanced functional materials and devices [98, 174]. Furthermore, the underlying principles of interfacial assembly can be extended to organize larger entities, such as colloidal particles, into ordered arrays for applications like photonic crystals or templating, as illustrated conceptually in Figure 16. Key application areas for Langmuir and LB films include sensors [24, 175, 176], molecular electronics [177, 65], energy storage [19, 178], and biomedical engineering [75, 20, 179], where their unique structural characteristics offer significant advantages over conventional thin-film fabrication methods.

6.1 Sensors

The fabrication of sensors, encompassing gas sensors, chemical sensors, and biosensors, represents a major application area where both Langmuir monolayers and transferred LB films provide distinct advantages. The performance metrics of sensors—sensitivity, selectivity, response time, and stability—are critically dependent on the structure and properties of the sensing layer. LB technology offers unparalleled control over these aspects. The highly ordered molecular arrangement achievable enhances the interaction between the sensing material and the target analyte, leading to improved sensitivity and selectivity. Furthermore, the ultra-thin nature of LB films facilitates

rapid analyte diffusion and charge transport, reducing response and recovery times.

Gas Sensors: LB films have been extensively developed for detecting various gases. Early work often utilized materials like conducting polymers [180], phthalocyanines [24, 23], and porphyrins. More recent examples leverage diverse materials and structures:

- *Fatty Acids:* LB films composed of arachidic acid (AA) and stearic acid (SA) integrated with surface acoustic wave (SAW) devices enable selective chloroform detection, outperforming responses to toluene and ethanol [176].

- *Polymer/Nanotube Composites:* Composite LB films, such as those made from polyaniline-functionalized multi-walled carbon nanotubes (PANI@MWCNTs), exhibit highly ordered structures enhancing ammonia (NH3) sensing [181]. The directional assembly via LB deposition minimizes aggregation and optimizes sensing site exposure, as conceptually shown in Figure 13.

- *Metal-Organic Frameworks (MOFs):* Known for high porosity, MOFs assembled into LB films detect gases like NO2 and humidity [182]. These films can be deposited on interdigitated electrodes and even flexible fabric substrates for wearable sensors.

- *2D Materials and Dyes:* Composite LB films combining modified black phosphorus nanosheets (BPNS) and dyes create sensors responsive to acid (HCl) and alkali (NH3) gases [60, 183, 184], sometimes enabling visual detection through color changes [16].

- *Metal Oxides:* Heterostructures like SnO2/TiO2 formed via LB deposition leverage synergistic effects at the n-n heterojunction for sensitive NO2 detection [185]. Amphiphilic iron(II) complexes assembled into LB monolayers have shown potential for nitric oxide (NO) detection [186].

Chemical Sensors (Liquid Phase): LB technology also facilitates sensing in liquids. Highly uniform and stable LB films functionalized with specific recognition elements detect ions and molecules with high sensitivity [3].

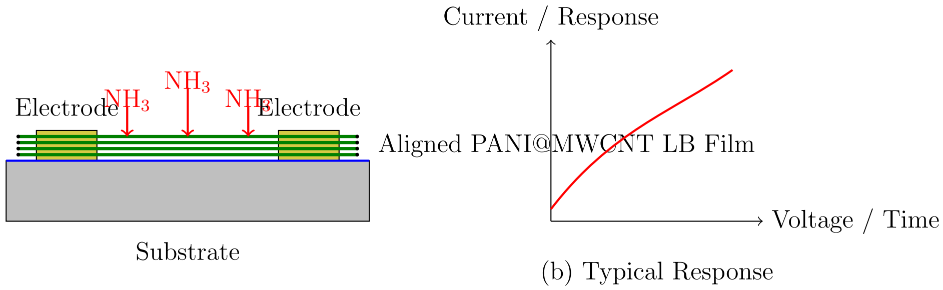

(a) Sensor Structure and Gas Interaction

(b) Typical Response

Figure 13: Schematic representation of a gas sensor based on aligned PANI@MWCNT LB films (a), illustrating the interaction with analyte gas molecules (e.g., NH3) leading to a measurable change in electrical properties, often depicted as a response curve (b). Inspired by studies on LB-based gas sensors.

- *Luminescent Probes:* LB films incorporating Eu3+ complex functionalized silica nanocomposites act as luminescent sensors for toxic anions (Cr2O72-, MnO4-, PO43-) in water, offering visual detection and low detection limits due to the ordered structure enhancing emission [187].

- *Electrocatalytic Sensors:* Nanostructured electrodes prepared by LB deposition of materials like functionalized gold nanoparticles and lutetium bisphthalocyanine exhibit synergistic electrocatalytic effects [188, 79], improving hydroquinone detection. Similarly, carboxylated multi-walled carbon nanotube (c-MWCNT)-Nafion LB films show superior electrocatalytic performance for detecting codeine [189].

Biosensors: This is a particularly significant domain, leveraging the ability of LB films to mimic biological environments and immobilize biomolecules while preserving activity.

- *Enzyme-Based Sensors:* Glucose oxidase (GOx) immobilized in LB films [190], often combined with AuNPs [191], enables sensitive glucose detection. Other enzymes like polyphenol oxidase (PPO), tyrosinase (Tyr) [192], asparaginase (ASNase) [193], penicillinase [194], and urease [81, 195] have been incorporated into LB films (often within lipid, polymer, or CNT matrices) to detect analytes like pyrocatechol [192, 196], polyphenols, aspartate, penicillin, and urea. The organized

proteo-lipidic nanostructures provide a crucial biomimetic environment [20, 197]. Incorporating nanomaterials like CNTs or graphene oxide (GO) enhances sensitivity, stability, and electron transfer [198].

- *Immunosensors:* LB films serve as platforms for antibody immobilization [199]. For example, MoS2/AuNP composite LB films provide substrates for immobilizing antibodies specific to dengue NS1 antigen, enabling dengue detection [200].

The precise control offered by LB methods over film structure and composition is key to advancing sensor performance across these diverse applications.

6.2 Molecular and Organic Electronics

The quest for miniaturized electronics fueled interest in molecular electronics, where molecules perform electronic functions. LB technology, with its precise control over molecular arrangement, was initially seen as ideal for fabricating devices like molecular rectifiers. Early studies indeed showed rectifier-like behavior in metal/LB film/metal structures. However, practical application faces challenges: the fragility of simple amphiphile films, defects over large areas, and difficulty in making reliable top contacts.

Despite these hurdles, LB films remain valuable for research in organic electronics, enabling proof-of-concept devices and studies of charge transport in ordered molecular systems [201, 177]. Organic field-effect transistors (OFETs) are a key example. High-quality monolayers, such as octadecyltrichlorosilane (ODTS), transferred via LB techniques onto substrates like silicon wafers, can serve as crucial surface modification layers atop primary dielectrics (e.g., SiO2) or potentially as ultra-thin dielectric layers themselves. This precise interfacial control, as demonstrated in studies using ODTS monolayers, can lead to OFETs with uniform performance over large areas by improving the semiconductor-dielectric interface. The organized structure imparted by LB deposition is also beneficial for photoelectrochemical devices; combining materials like methylene blue (MB) dye with modified black phosphorus nanosheets (BPNSs) in LB films enhances performance through efficient charge separation and transport in the ordered assembly [60, 202]. Furthermore, LB assembly of uniform nanocrystal floating gates facilitates the development of memory devices, potentially integrated into wearable sensing platforms. While facing competition from more scalable methods like SAMs and LbL for industrial use [5, 101, 22], LB technology

remains important for fabricating large-area devices, especially on flexible substrates, and for fundamental investigations into structure-property relationships in highly organized molecular electronic systems.

6.3 Energy Storage

The demand for high-performance energy storage devices like lithium-ion batteries (LIBs), lithium-sulfur (Li-S) batteries, and supercapacitors drives research into novel electrode materials and architectures. LB technology offers a unique platform for fabricating nanostructured electrodes and functional interlayers with precisely controlled composition and morphology, potentially overcoming limitations of conventional methods.

LB films have been used to create advanced electrode materials. For example, hybrid multilayer electrodes of graphene nanosheets (GNS) and single-walled carbon nanotubes (SWNTs) fabricated via LB deposition exhibit controlled nanoscale architecture [203]. These electrodes show high specific capacity and good cycling performance as LIB anodes due to synergistic effects. Binder-free anodes from SnO2/reduced graphene oxide (SnO2/RGO) nanocomposites, prepared by sequential LB deposition, demonstrate high initial capacity [204], with the layered structure accommodating SnO2 volume changes while maintaining conductivity. Graphene-like porous 2D Co3O4 assemblies, templated using GO LB films, also show high reversible capacity as LIB anodes [205].

Beyond active materials, LB films function as coatings or interlayers. In Li-S batteries, composite films of polyethylenimine (PEI) and multi-walled carbon nanotubes (MWCNTs) deposited onto separators via Langmuir-Blodgett-Schaefer (LBS) technique effectively trap polysulfides, enhancing rate performance and stability [206, 207]. Artificial solid electrolyte interphase (SEI) layers, such as those made from lithium-terminated sulfonated titania (LTST) nanoparticles assembled via LB and transferred onto Li metal, can stabilize the anode surface [208, 209, 210]. Supercapacitors also benefit from LB-fabricated electrodes; nanocomplexes of graphene oxide (GO), MnO2, and dimyristoylphosphatidic acid (DMPA) assembled onto conductive electrodes show promise for bio-devices [69, 66]. The precise structural control makes LB technology valuable for engineering interfaces and designing advanced materials for next-generation energy storage, offering advantages in creating layered heterostructures [5, 6] and potentially enabling large-scale fabrication via roll-to-roll adaptations [101].

6.4 Biomedical Applications

Beyond the biosensing applications detailed earlier (Section 6.1), LB technology is highly relevant for biomimetic membrane studies, drug delivery, and tissue engineering, leveraging its ability to mimic biological structures, control surface properties molecularly, and incorporate bioactive molecules.

LB films composed of lipids, such as phospholipids, serve as excellent models for cell membranes [211, 12]. These artificial membranes facilitate controlled studies of fundamental biological processes like protein interactions [53, 212], ion transport, and drug effects on membrane structure [213, 214]. Langmuir monolayers at the air-water interface, the precursors to LB films, are themselves powerful tools for studying molecular interactions (e.g., drug-lipid binding) by monitoring surface pressure or potential changes [215, 216]. Increasingly realistic models are built by varying lipid composition and incorporating components like cholesterol [49, 51] or proteins. Even entire cell membrane extracts, like bovine erythrocyte membranes (BEMs), have been formed into LB films to enhance membrane enzyme activity for applications like pesticide detection.

While less emphasized in the provided references, the controlled layering of LB films holds potential for drug delivery systems [22, 217, 218], enabling precise loading and potentially stimuli-responsive release, particularly for localized therapies.

In tissue engineering, LB technology's ability to create ordered nanoscale structures is advantageous for fabricating scaffolds mimicking the extracellular matrix to guide cell behavior [219, 11, 76]. For instance, aligned C60 nanowhiskers assembled via LB technique served as scaffolds that maintained the self-renewal and multipotency of human mesenchymal stem cells [220, 221], highlighting the role of topography in directing cell fate, as conceptually illustrated in Figure 14. Biocompatibility is crucial, and many materials used (lipids, peptides, polysaccharides, selected polymers) are inherently biocompatible. However, long-term stability and degradation in physiological environments require careful material selection and architectural design. The versatility in material choice and structural control makes LB technology a valuable platform for advancing biomedical research and developing novel therapeutic and diagnostic tools [22, 5, 101].

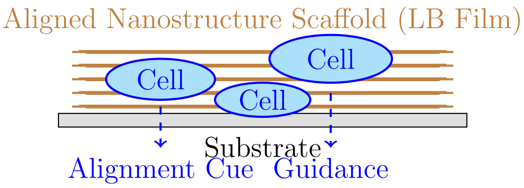

Figure 14: Conceptual illustration of cells cultured on an aligned nanostructure scaffold fabricated using the LB technique. The ordered topography can influence cell alignment and behavior, relevant for tissue engineering applications.

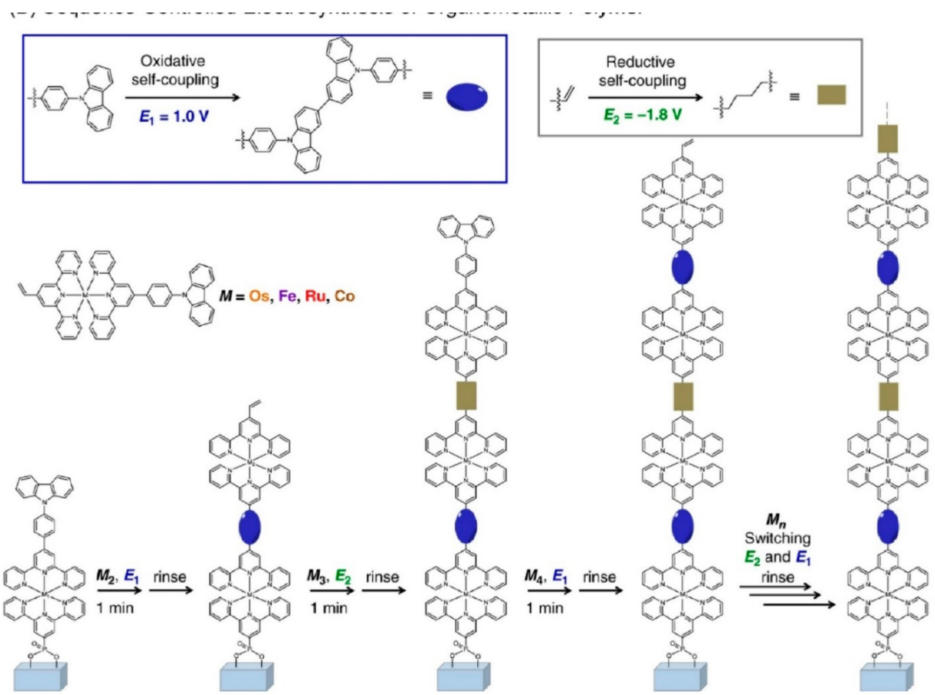

Figure 15: LB films with molecularly nanoarchitected materials: monolayers of bottlebrush polymers with topologically precise and perfectly discrete structures. Adapted from Ariga, K., 2023 [6].

7 Challenges and Future Directions

Despite its century-long history and demonstrated capability for precise molecular assembly, Langmuir-Blodgett (LB) technology faces several persistent

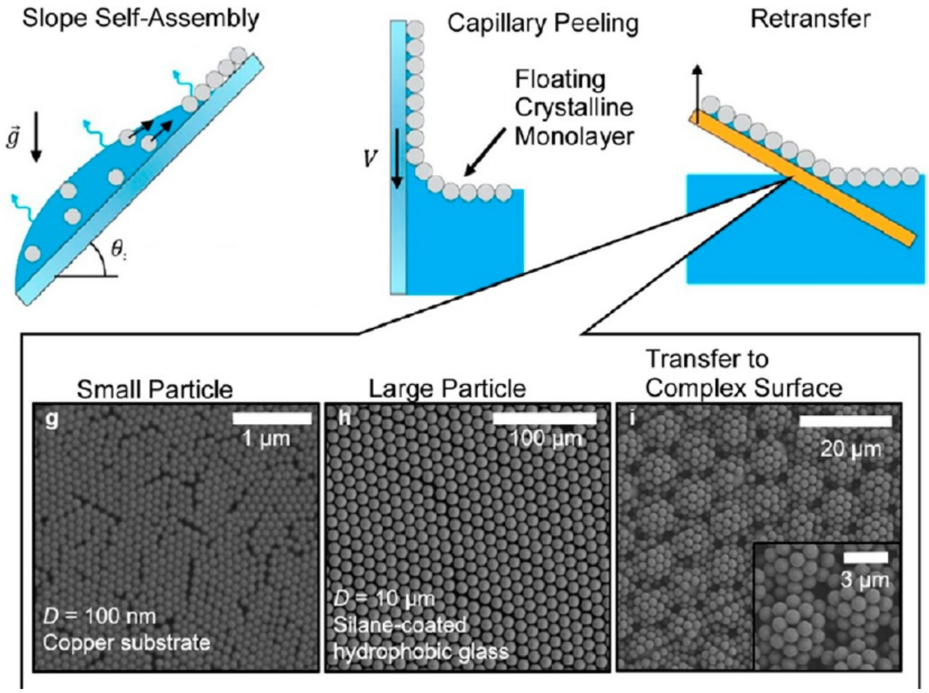

Figure 16: Two-dimensional colloidal nanoarchitectonics illustrating principles related to interfacial assembly, including slope self-assembly, capillary peeling, and retransfer onto arbitrary substrates. These techniques allow immobilization of colloidal spheres (100 nm to 10 μm) and conformal coating. Adapted from Ariga, K., 2023 [6].

challenges that have limited its widespread industrial adoption [25, 222]. However, ongoing research efforts, driven by advancements in materials science, nanotechnology, and instrumentation, are continuously addressing these limitations and opening up exciting new avenues for future development and application [101, 21]. The unique control offered by the Langmuir technique over monolayer structure at the air-liquid interface, and the LB technique over transferred film structure at the molecular level, remains a powerful incentive for overcoming inherent difficulties, positioning it as a key enabling technology for future innovations across diverse scientific and technological domains [98, 7]. The journey from fundamental exploration to practical implementation necessitates a clear understanding of the current hurdles and a strategic focus on emerging opportunities and future research directions.

7.1 Addressing Current Limitations

One of the most significant limitations historically associated with LB films, particularly those derived from traditional small amphiphilic molecules, is their lack of mechanical robustness and long-term stability. These films can be fragile and susceptible to damage during handling, processing, or operation, hindering their use in demanding applications. The inherent fragility stems from the weak van der Waals forces often holding the amphiphiles together in the monolayer. While techniques exist to transfer these delicate monolayers, maintaining their integrity and defect-free nature on solid supports, especially over large areas, remains challenging.

Scalability and production cost represent another major hurdle for the industrial translation of LB technology. The conventional LB deposition process, involving slow, meticulous transfer from an air-water interface within a specialized trough, is inherently low-throughput compared to other thin-film deposition techniques like spin-coating or dip-coating. Fabricating large-area films with uniform quality using standard LB troughs is difficult and time-consuming [177]. Figure 17 illustrates an attempt to address this challenge by transferring an octadecyltrichlorosilane (ODTS) monolayer onto a relatively large (3-inch) silicon wafer. The subsequent fabrication of an array of organic field-effect transistors (OFETs) and the mapping of their mobilities (Figure 17c) demonstrate the potential for achieving functional device uniformity over larger areas. However, such efforts highlight the complexity and specialized equipment required, contributing to the high cost and difficulty in scaling up LB processes for mass production. The requirement for high-purity subphases (typically ultrapure water), volatile organic solvents for spreading, stringent environmental controls (vibration isolation, dust-free atmosphere, precise temperature control), and sophisticated instrumentation further contributes significantly to the overall complexity and cost.

Reproducibility can also be a concern. The final film quality is highly sensitive to numerous experimental parameters, including subphase purity and temperature, spreading solvent volatility, compression speed and final surface pressure [33], deposition speed, substrate surface chemistry [164], and ambient conditions. Slight variations in any of these parameters can lead to inconsistencies in film structure, thickness, and properties, making reliable and reproducible fabrication challenging, especially when scaling up or transferring processes between different laboratories or manufacturing settings.

Furthermore, the range of materials suitable for classical LB deposition

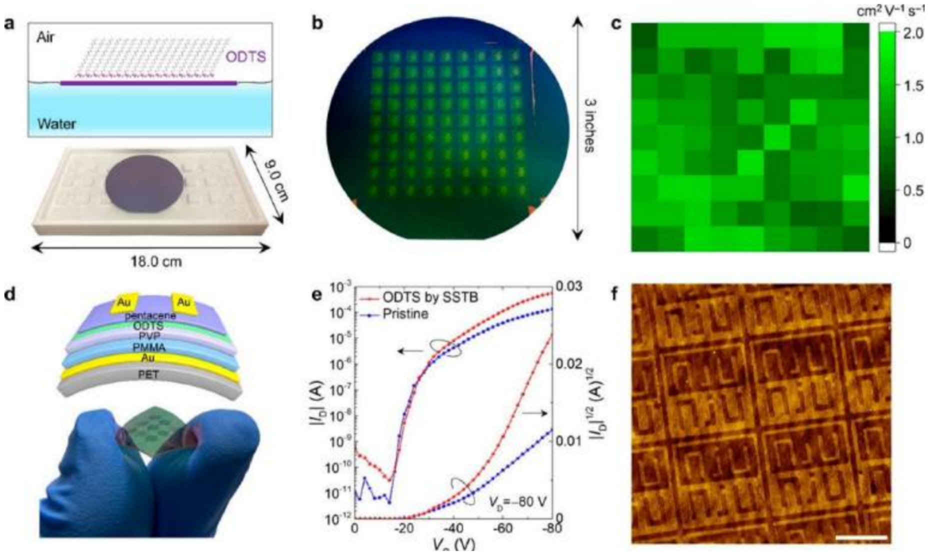

Figure 17: Schematic diagram of the trough used to transfer the ODTS monolayer onto solid substrates, an adaptation of a Langmuir trough. The monolayer was transferred onto a 3 in. silicon wafer. (b) Photo of the array of OFET's, whose mobilities are mapped in (c). The architecture of the OFETs is shown in (d), while the current—voltage curves are given in (e). A pattern (NJU) was written in an AFM image, with a scale bar = 10 μm and Z-scale = 12 nm. Adapted from Oliveira, O., et al., 2022 [90].

was initially limited to amphiphilic molecules capable of forming stable Langmuir monolayers at the air-water interface [12]. While this range has been significantly expanded, incorporating non-amphiphilic materials often requires chemical modification (e.g., attaching hydrophobic chains) or co-spreading with helper amphiphiles, adding complexity to the process. Some materials, particularly rigid nanoparticles or polymers, may form aggregates or domains at the interface rather than true monolayers [33], potentially leading to defects or non-uniformity upon transfer. The transfer process itself can be problematic for certain materials or substrates; rigid films might crack during transfer, and achieving high transfer ratios (close to unity) consistently is not always guaranteed [223, 224].

The emergence of alternative techniques like self-assembled monolayers (SAMs) and layer-by-layer (LbL) assembly presented significant competition. SAMs, introduced by Sagiv and others [225], form robust chemisorbed

layers, offering greater stability than typical physisorbed LB films. The LbL technique, pioneered by Decher and others [38, 39], provides a simpler, more versatile method for constructing multilayers from a wide variety of water-soluble charged materials (polyelectrolytes, nanoparticles, biomolecules) [99, 10]. LbL assembly is generally less demanding in terms of equipment and procedure, more easily scalable [5, 15], and often yields more robust films, making it attractive for many industrial applications, even though the degree of molecular order achieved may be lower than in ideal LB films. These factors contributed to a shift in focus for some applications previously envisioned for LB films.

7.2 Overcoming Challenges through Innovation

Recognizing the limitations outlined above, researchers have actively pursued innovative strategies focusing on materials development, advanced fabrication techniques, and process optimization, specifically aiming to enhance robustness, broaden the material scope, and improve scalability. A major thrust has been the incorporation of more robust molecular and nanoscale building blocks to directly address the fragility of traditional LB films. Polymers, both pre-formed and generated via *in situ* polymerization, have been extensively investigated. *In situ* polymerization typically involves either (i) polymerizing monomers spread at the air-water interface before transferring the resulting polymer Langmuir film, or (ii) transferring a monomer monolayer onto the solid substrate first, followed by inducing polymerization (e.g., via UV light or heat) on the support. These polymeric LB films exhibit enhanced mechanical strength, thermal stability, and environmental resistance due to the covalent linkages within the polymer chains, offering significantly greater cohesion compared to non-covalent interactions in small-molecule amphiphile films.

Similarly, the integration of nanomaterials—such as carbon nanotubes (CNTs) [194, 226, 81], graphene and its derivatives (like graphene oxide) [66], nanoparticles (metallic, semiconducting, magnetic) [77], nanowires [178], and 2D materials like black phosphorus nanosheets (BPNS) [78, 60, 202], metal-organic frameworks (MOFs) [227], and covalent organic frameworks (COFs) [68]—has yielded composite LB films. This approach tackles both fragility, by reinforcing the film structure, and limited functionality, by imparting desirable electrical, optical, or mechanical characteristics. These nanomaterials can form stable Langmuir layers, sometimes with the aid of surfactants or

functionalization. Strategies to functionalize non-amphiphilic materials, such as alkylation of chitosans or nucleotides, have also successfully rendered them amenable to Langmuir film formation and subsequent LB transfer, directly expanding the range of materials beyond classical amphiphiles.

Advancements in instrumentation and deposition techniques are crucial for overcoming scalability and complexity issues. Efforts to develop larger troughs and automated systems aim for higher throughput and better process control. Novel LB-type methods have emerged to address specific material challenges. For instance, the high-temperature LB technique, using low-vapor-pressure subphases like ethylene glycol or ionic liquids, directly tackles the problem of undesirable aggregation of molecules like conjugated polymers at room temperature [59, 40]. Operating at elevated temperatures enhances molecular mobility, leading to more ordered and crystalline films with improved properties (e.g., charge transport), essential for applications like organic electronics. The Vortex LB method, employing rotational flow, represents a departure from the traditional quiescent interface requirement and offers alternative assembly mechanisms. The Langmuir-Schaefer (LS) method (horizontal transfer) provides an alternative to vertical dipping, advantageous for depositing onto hydrophobic surfaces or transferring rigid monolayers prone to disruption by meniscus forces [42]. Combining LB deposition with other techniques, like using LB films as masks for reactive ion etching (RIE) or integrating LB layers within LbL assemblies [22, 11, 15, 101], allows for complex hierarchical structures, leveraging the strengths of multiple methods to overcome the limitations of LB alone [84].

Improved process control and characterization are essential for enhancing reproducibility. Real-time monitoring and advanced *in situ* characterization (discussed in Section 5) provide deeper insights into film formation, allowing finer control [228, 154]. A better fundamental understanding of interfacial interactions guides parameter optimization, leading to more reliable film quality [229, 97]. Careful substrate preparation remains critical for uniform adhesion and transfer [164].

7.3 Emerging Trends and Opportunities

The field of Langmuir-Blodgett films is continually evolving, driven by the convergence of materials science, nanotechnology, and interfacial science. A prominent emerging trend is the deeper integration of LB technology within the paradigm of nanoarchitectonics. This concept emphasizes the deliber-

ate construction of functional materials systems from nanoscale components, moving beyond simple monolayer or multilayer deposition towards the creation of complex, hierarchical structures with precisely controlled architectures. LB films serve as ideal building blocks or templates in nanoarchitectonics, enabling the arrangement of diverse components—molecules, polymers, nanoparticles, biomolecules—with molecular-level precision. The air-water interface itself is increasingly viewed as a unique reaction field for "interfacial nanoarchitectonics," where molecular recognition events, supramolecular assembly, and even chemical reactions can be controlled and studied with high precision before transfer.

The exploration of new and unconventional materials continues to expand the horizons of LB technology. Beyond polymers and nanoparticles, researchers are actively investigating LB films based on emerging nanomaterials like MOFs and COFs [68, 230, 231]. These porous crystalline materials offer vast surface areas and tunable functionalities. As illustrated in Figure 18, complex structures like porphyrin-based MOFs can be formed as Langmuir films at the air-water interface, demonstrating the feasibility of using LB techniques to create highly oriented, thin films of these materials for applications in sensing, catalysis, and separation [112, 232, 227]. Two-dimensional materials beyond graphene, such as transition metal dichalcogenides (TMDs), black phosphorus [60, 78, 205], and MXenes, are also being incorporated into LB films. Biomolecules, including peptides, proteins, DNA [233], and lipid mixtures mimicking cell membranes [66], are assembled using LB techniques for biosensing, drug delivery [22], and fundamental biophysical studies. Hybrid films combining inorganic nanomaterials with organic or biomolecules open pathways to multifunctional systems [179].

This leads to another significant trend: the development of multifunctional LB films [77, 207, 184]. By carefully selecting and arranging different components, researchers can design materials that perform multiple tasks simultaneously, such as sensor platforms combining recognition and transduction elements [17, 24, 23], or catalytic surfaces integrated into devices [188, 227]. The precise layering capability of the LB technique is key here.

Furthermore, the integration of LB films with other micro- and nanotechnologies is unlocking new possibilities, including patterning via lithography [103], use as high-resolution masks, and combination with SAMs or LbL assemblies for complex hybrid structures [22, 11, 10, 6].

These trends create significant opportunities for LB technology. In energy, applications include organic solar cells [234], advanced battery compo-

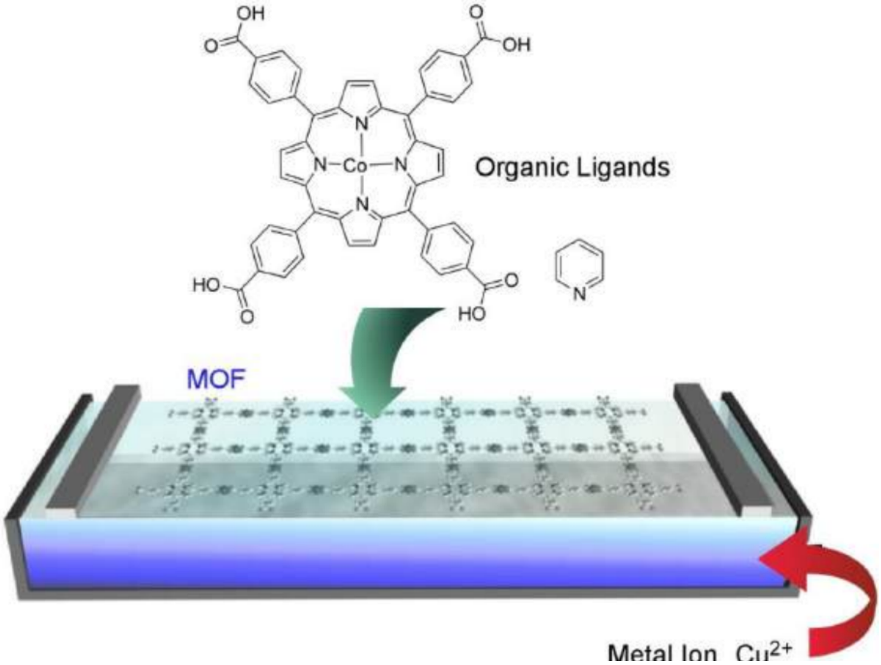

Figure 18: Porphyrin-based MOF film formed at the air—water interface. Adapted from Oliveira, O., et al., 2022 [90].

nents (electrodes [19], separators, solid-state electrolytes), and electrocatalysts [227]. In environmental applications, high-performance chemical and gas sensors [60, 233] and potentially water purification membranes are being developed. In healthcare, LB films provide platforms for biomimetic membrane studies [235, 76] crucial for drug design, highly sensitive biosensors [75, 190, 194, 79], and precisely engineered tissue engineering scaffolds [22, 236]. The ability to control surface chemistry and topography with LB films offers potential for guiding cell behavior (adhesion, proliferation, differentiation) – a concept referred to as "life control," highlighting the potential to direct biological processes using nanoarchitected surfaces [108, 71, 237].

7.4 Future Research Perspectives

To fully realize the potential of Langmuir-Blodgett technology, future research should focus on several key areas. A deeper fundamental understanding of the processes occurring at the air-liquid interface remains crucial.

Advanced *in situ* characterization techniques, combined with sophisticated computational modeling (like molecular dynamics simulations), are needed to elucidate the complex interplay of intermolecular forces, molecular conformation, domain formation, phase transitions, and dynamic processes during Langmuir monolayer compression and LB transfer. Understanding how different molecules and nanomaterials self-assemble at the interface under varying conditions is essential for rationally designing and controlling film structure and properties.

Developing more efficient, scalable, and cost-effective fabrication methods is paramount. Research into continuous or roll-to-roll LB deposition [223], automated systems with real-time monitoring and feedback control, and alternative, environmentally benign solvents/subphases is needed. Continued innovation in advanced LB techniques (e.g., high-temperature [59, 40], vortex) may expand the range of processable materials. Standardization of protocols and characterization methods would improve reliability and facilitate comparison across studies. Ensuring the quality and uniformity of the fabricated films is critical for reliable device performance; techniques like Scanning Electron Microscopy (SEM), as shown in Figure 19, are indispensable for characterizing the morphology and homogeneity of LB films, thereby validating the effectiveness of fabrication processes and control strategies.

The exploration of novel materials and complex architectures should remain a central theme, including new molecules designed for LB assembly, emerging 2D materials [238, 6], MOFs, COFs, biomolecular systems [70], and robust, defect-tolerant films. Designing sophisticated 3D nanoarchitectures, possibly using LB with other techniques [11], and fabricating heterostructures with precisely controlled interfaces are exciting frontiers.

Application-driven research is essential, tailoring LB film properties for specific targets in electronics [239], sensing [240, 241, 24], energy [242], catalysis [234], or biomedicine [22, 236]. Developing stable films for harsh environments (e.g., batteries) or biocompatible films for tissue engineering requires specific material selection and interface engineering. Robust characterization of film performance *within* the intended application environment is critical for validation.

Finally, advancing the field will increasingly rely on interdisciplinary collaborations spanning physics, chemistry, materials science, engineering, and biology. Synergistic efforts combining expertise in synthesis, interfacial science, nanofabrication, device engineering, modeling, and biological evaluation are key to overcoming remaining hurdles and unlocking the full potential

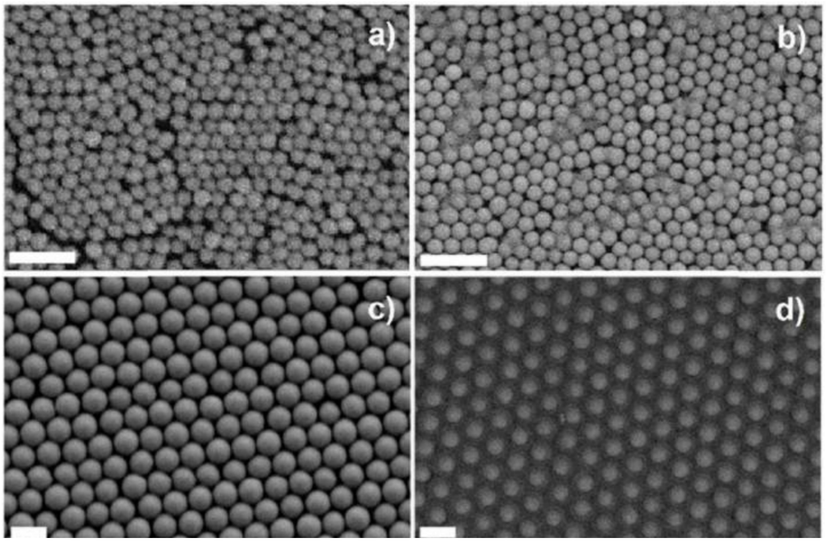

Figure 19: Uniformity of the thicker LB films is apparent by comparing the SEM images for: LB 120 nm (a); LB 120 nm/PVA (b); LB 740 nm (c); and LB 740 nm/PVA (4d). In the images, the scale bars correspond to 1 μm. Adapted from Oliveira, O., et al., 2022 [90].

of this versatile molecular assembly technique. The rich history of Langmuir and Langmuir-Blodgett films provides a solid foundation, but their future lies in continued innovation, integration with other fields, and a focus on addressing real-world problems.

8 Conclusion

This survey reviewed the history, fundamental principles, advanced methodologies, characterization techniques, diverse applications, and persistent challenges associated with Langmuir and Langmuir-Blodgett (LB) film technology. Originating over a century ago, the LB technique was the first method enabling the controlled assembly of molecules into highly ordered, ultra-thin films [7, 25, 98]. We explored the core process, which involves forming a Langmuir monolayer at the air-water interface through the self-assembly of suitable molecules or nanomaterials [174, 13], followed by its systematic transfer onto solid substrates [223, 243, 56]. This process creates LB films with precise control over thickness and architecture, often down to the

single-monolayer level. A fundamental understanding of surface pressure-area isotherms, monolayer phase behavior [14], and transfer mechanics remains essential for optimizing film quality.

The evolution of the LB technique was highlighted, demonstrating its expansion beyond traditional amphiphiles. A wide array of materials can now be incorporated into Langmuir and LB films [7, 25, 98]. These include polymers [57, 58], various nanoparticles (such as metallic, semiconducting, and magnetic types) [64, 62, 103, 77], and carbon nanomaterials like graphene, nanotubes, and fullerenes [189, 78]. Furthermore, two-dimensional materials, including black phosphorus [202, 60], metal-organic frameworks (MOFs) [112], covalent organic frameworks (COFs) [68], and complex biomolecules, are increasingly utilized. Advanced fabrication approaches, such as modified trough designs and high-temperature deposition [40, 223, 56, 77], aim to overcome limitations. Comparisons with related thin-film methods like self-assembled monolayers (SAMs) [244] and layer-by-layer (LbL) assembly [10, 15, 11, 101, 38] were discussed. While SAMs provide robust chemisorbed monolayers and LbL offers versatility for water-soluble materials, the LB technique retains unique advantages in achieving highly ordered structures and controlling molecular orientation, particularly for insoluble materials spread at the air-water interface [245]. The importance of sophisticated characterization techniques—spanning surface manometry, microscopy (AFM, BAM, SEM, TEM) [148, 143, 14], spectroscopy (FTIR, Raman, XPS), and scattering methods (X-ray, neutron)—was emphasized for understanding the structure, morphology, and properties of both Langmuir monolayers and transferred LB films.

A significant portion of this survey detailed the diverse applications where LB films show considerable potential. In gas sensing, the ordered, ultra-thin nature of LB films allows for enhanced sensitivity and selectivity. Integrating advanced materials, such as MOFs, specific nanomaterials, conducting polymers, and phthalocyanines, enables tailored sensor responses to various analytes like VOCs and toxic gases. In electrochemistry, LB films serve as model systems for studying interfacial electron transfer and modifying electrode surfaces. The precise control over film structure facilitates the creation of well-defined architectures for catalysis and energy storage components, incorporating materials like graphene or nanoparticles. Furthermore, the unique ability of Langmuir monolayers to mimic biological membranes at the air-water interface makes this technology invaluable for biophysical studies and developing biomimetic films and biosensors. Applications in molecular

electronics continue to leverage LB films for proof-of-concept devices, despite practical hurdles. The concept of nanoarchitectonics frequently utilizes the LB method as a key tool for precise two-dimensional organization, building functional systems from nanoscale components.

Despite its unique capabilities, the LB technique faces challenges limiting widespread industrial adoption. Key limitations include the mechanical fragility of some films, particularly those from small amphiphiles, and difficulties in achieving large-area, defect-free deposition consistently. The relatively slow, complex, and often costly nature of the process, requiring specialized equipment and careful control, also hinders broader application, alongside concerns about long-term stability. Efforts to address these issues using polymeric or nanoparticle-based films and improved instrumentation are ongoing.

In conclusion, Langmuir-Blodgett technology remains a cornerstone technique in surface science and nanotechnology, offering unparalleled precision in constructing molecularly organized ultra-thin films at the air-water interface (Langmuir films) and on solid supports (LB films). Its primary advantages lie in the exquisite control over film thickness, molecular arrangement, and orientation. While challenges related to robustness, scalability, and cost persist, the unique strengths of the LB method ensure its continued importance in fundamental research exploring interfacial phenomena, molecular recognition, biomimetic systems, and self-assembly. It is an indispensable tool for creating model systems and proof-of-concept devices where precise structural control is paramount. The future likely involves synergistic approaches, combining LB methods with other fabrication techniques, exploring novel functional materials amenable to interfacial assembly, and focusing on applications where molecular-level precision offers distinct advantages—such as advanced sensors, model biological interfaces, and components for molecular-scale devices. Continued innovation in materials science, instrumentation, and the understanding of interfacial nanoarchitectonics is crucial to overcome existing hurdles and fully realize the potential of Langmuir and Langmuir-Blodgett films across diverse scientific and technological fields.

References

[1] Katsuhiko Ariga et al. "25th Anniversary Article: What Can Be Done with the Langmuir-Blodgett Method? Recent Developments and its Critical Role in Materials Science". In: *Advanced Materials* 25.45 (2013), 6477–6512. ISSN: 1521-4095. DOI: 10.1002/adma.201302283. URL: http://dx.doi.org/10.1002/adma.201302283.

[2] J. A. Zasadzinski et al. "Langmuir-Blodgett films". In: *Science* 263.5154 (1994), 1726–1733. ISSN: 1095-9203. DOI: 10.1126/science.8134836. URL: http://dx.doi.org/10.1126/science.8134836.

[3] Wenhui Gu et al. "Recent Progress in the Applications of Langmuir–Blodgett Film Technology". In: *Nanomaterials* 14.12 (2024), p. 1039. ISSN: 2079-4991. DOI: 10.3390/nano14121039. URL: http://dx.doi.org/10.3390/nano14121039.

[4] Katsuhiko Ariga et al. "Materials nanoarchitectonics at two-dimensional liquid interfaces". In: *Beilstein Journal of Nanotechnology* 10 (2019), 1559–1587. ISSN: 2190-4286. DOI: 10.3762/bjnano.10.153. URL: http://dx.doi.org/10.3762/bjnano.10.153.

[5] Katsuhiko Ariga et al. "Layer-by-layer Nanoarchitectonics: Invention, Innovation, and Evolution". In: *Chemistry Letters* 43.1 (2013), 36–68. ISSN: 1348-0715. DOI: 10.1246/cl.130987. URL: http://dx.doi.org/10.1246/cl.130987.

[6] Katsuhiko Ariga. "Chemistry of Materials Nanoarchitectonics for Two-Dimensional Films: Langmuir–Blodgett, Layer-by-Layer Assembly, and Newcomers". In: *Chemistry of Materials* 35.14 (2023), 5233–5254. ISSN: 1520-5002. DOI: 10.1021/acs.chemmater.3c01291. URL: http://dx.doi.org/10.1021/acs.chemmater.3c01291.

[7] Katsuhiko Ariga. "Don't Forget Langmuir–Blodgett Films 2020: Interfacial Nanoarchitectonics with Molecules, Materials, and Living Objects". In: *Langmuir* 36.26 (2020), 7158–7180. ISSN: 1520-5827. DOI: 10.1021/acs.langmuir.0c01044. URL: http://dx.doi.org/10.1021/acs.langmuir.0c01044.

[8] R H Tredgold. "The physics of Langmuir-Blodgett films". In: *Reports on Progress in Physics* 50.12 (1987), 1609–1656. ISSN: 1361-6633. DOI: 10.1088/0034-4885/50/12/002. URL: http://dx.doi.org/10.1088/0034-4885/50/12/002.

[9] Daniel K. Schwartz. “Langmuir-Blodgett film structure”. In: *Surface Science Reports* 27.7–8 (1997), 245–334. ISSN: 0167-5729. DOI: 10.1016/s0167-5729(97)00003-4. URL: http://dx.doi.org/10.1016/s0167-5729(97)00003-4.

[10] Gero Decher. “Fuzzy Nanoassemblies: Toward Layered Polymeric Multicomposites”. In: *Science* 277.5330 (1997), 1232–1237. ISSN: 1095-9203. DOI: 10.1126/science.277.5330.1232. URL: http://dx.doi.org/10.1126/science.277.5330.1232.

[11] Mitsuru Akashi and Takami Akagi. “Composite Materials by Building Block Chemistry Using Weak Interaction”. In: *Bulletin of the Chemical Society of Japan* 94.7 (2021), 1903–1921. ISSN: 1348-0634. DOI: 10.1246/bcsj.20210089. URL: http://dx.doi.org/10.1246/bcsj.20210089.

[12] B. Gzyl and M. Paluch. “Langmuir monolayers of lipids at the water/air interface”. In: *Trends in Colloid and Interface Science XVI*. Springer Berlin Heidelberg, 2004, 245–250. ISBN: 9783540364627. DOI: 10.1007/978-3-540-36462-7_53. URL: http://dx.doi.org/10.1007/978-3-540-36462-7_53.

[13] Katsuhiko Ariga and Jonathan P. Hill. “Monolayers at air-water interfaces: from origins-of-life to nanotechnology”. In: *The Chemical Record* 11.4 (2011), 199–211. ISSN: 1528-0691. DOI: 10.1002/tcr.201100004. URL: http://dx.doi.org/10.1002/tcr.201100004.

[14] Da Shi et al. “Interfacial Behavior of Oligo(Ethylene Glycol) Dendrons Spread Alone and in Combination with a Phospholipid as Langmuir Monolayers at the Air/Water Interface”. In: *Molecules* 24.22 (2019), p. 4114. ISSN: 1420-3049. DOI: 10.3390/molecules24224114. URL: http://dx.doi.org/10.3390/molecules24224114.

[15] Katsuhiko Ariga, Jonathan P. Hill, and Qingmin Ji. “Layer-by-layer assembly as a versatile bottom-up nanofabrication technique for exploratory research and realistic application”. In: *Physical Chemistry Chemical Physics* 9.19 (2007), p. 2319. ISSN: 1463-9084. DOI: 10.1039/b700410a. URL: http://dx.doi.org/10.1039/b700410a.

[16] Na Li et al. “Gas-Responsive and Self-Powered Visual Composite Langmuir–Blodgett Films for Ultrathin Gas Sensors”. In: *Langmuir* 38.21 (2022), 6761–6770. ISSN: 1520-5827. DOI: 10.1021/acs.langmuir.

2c00835. URL: http://dx.doi.org/10.1021/acs.langmuir.2c00835.

[17] Na Li et al. "Chemical gas sensor, surface enhanced Raman scattering and photoelectrics of composite Langmuir-Blodgett films consisting of polypeptide and dye molecules". In: *Colloids and Surfaces A: Physicochemical and Engineering Aspects* 663 (2023), p. 131067. ISSN: 0927-7757. DOI: 10.1016/j.colsurfa.2023.131067. URL: http://dx.doi.org/10.1016/j.colsurfa.2023.131067.

[18] Chen Fang et al. "Recent Applications of Langmuir–Blodgett Technique in Battery Research". In: *ACS Applied Materials amp; Interfaces* 14.2 (2022), 2431–2439. ISSN: 1944-8252. DOI: 10.1021/acsami.1c19064. URL: http://dx.doi.org/10.1021/acsami.1c19064.

[19] Muthukumar Divagar, Nagamony Ponpandian, and Chinnuswamy Viswanathan. "Langmuir-Blodgett deposited Na3V2(PO4)3-MnO2 nanocomposite thin film electrodes for hybrid energy storage application". In: *Materials Science and Engineering: B* 270 (2021), p. 115229. ISSN: 0921-5107. DOI: 10.1016/j.mseb.2021.115229. URL: http://dx.doi.org/10.1016/j.mseb.2021.115229.

[20] Agnès P. Girard-Egrot, Stéphanie Godoy, and Loïc J. Blum. "Enzyme association with lipidic Langmuir–Blodgett films: Interests and applications in nanobioscience". In: *Advances in Colloid and Interface Science* 116.1–3 (2005), 205–225. ISSN: 0001-8686. DOI: 10.1016/j.cis.2005.04.006. URL: http://dx.doi.org/10.1016/j.cis.2005.04.006.

[21] Katsuhiko Ariga. "Langmuir–Blodgett Nanoarchitectonics, Out of the Box". In: *Accounts of Materials Research* 3.4 (2021), 404–410. ISSN: 2643-6728. DOI: 10.1021/accountsmr.1c00240. URL: http://dx.doi.org/10.1021/accountsmr.1c00240.

[22] João Borges et al. "Recent Developments in Layer-by-Layer Assembly for Drug Delivery and Tissue Engineering Applications". In: *Advanced Healthcare Materials* 13.8 (2024). ISSN: 2192-2659. DOI: 10.1002/adhm.202302713. URL: http://dx.doi.org/10.1002/adhm.202302713.

[23] Gabriele Giancane and Ludovico Valli. “State of art in porphyrin Langmuir–Blodgett films as chemical sensors”. In: *Advances in Colloid and Interface Science* 171–172 (2012), 17–35. ISSN: 0001-8686. DOI: 10.1016/j.cis.2012.01.001. URL: http://dx.doi.org/10.1016/j.cis.2012.01.001.

[24] Ludovico Valli. “Phthalocyanine-based Langmuir–Blodgett films as chemical sensors”. In: *Advances in Colloid and Interface Science* 116.1–3 (2005), 13–44. ISSN: 0001-8686. DOI: 10.1016/j.cis.2005.04.008. URL: http://dx.doi.org/10.1016/j.cis.2005.04.008.

[25] Osvaldo N. Oliveira and Diogo Volpati. “Langmuir and Langmuir–Blodgett (LB) Films”. In: *Encyclopedia of Membranes*. Springer Berlin Heidelberg, 2016, 1089–1089. ISBN: 9783662443248. DOI: 10.1007/978-3-662-44324-8_1525. URL: http://dx.doi.org/10.1007/978-3-662-44324-8_1525.

[26] Waka Nakanishi et al. “Monitoring Fluorescence Response of Amphiphilic Flapping Molecules in Compressed Monolayers at the Air–Water Interface”. In: *Chemistry – An Asian Journal* 14.16 (2019), 2869–2876. ISSN: 1861-471X. DOI: 10.1002/asia.201900769. URL: http://dx.doi.org/10.1002/asia.201900769.

[27] Xuezhong Du, Wangen Miao, and Yingqiu Liang. “IRRAS Studies on Chain Orientation in the Monolayers of Amino Acid Amphiphiles at the AirWater Interface Depending on Metal Complex and Hydrogen Bond Formation with the Headgroups”. In: *The Journal of Physical Chemistry B* 109.15 (2005), 7428–7434. ISSN: 1520-5207. DOI: 10.1021/jp0441700. URL: http://dx.doi.org/10.1021/jp0441700.

[28] Qun Huo, K. C. Russell, and Roger M. Leblanc. “Effect of Complementary Hydrogen Bonding Additives in Subphase on the Structure and Properties of the 2-Amino-4,6-dioctadecylamino-1,3,5-triazine Amphiphile at the AirWater Interface: Studies by UltravioletVisible Absorption Spectroscopy and Brewster Angle Microscopy”. In: *Langmuir* 14.8 (1998), 2174–2186. ISSN: 1520-5827. DOI: 10.1021/la971329+. URL: http://dx.doi.org/10.1021/la971329.

[29] Masanari Okuno et al. “Hydrogen Bonds and Molecular Orientations of Supramolecular Structure between Barbituric Acid and Melamine Derivative at the Air/Water Interface Revealed by Heterodyne-Detected

Vibrational Sum Frequency Generation Spectroscopy”. In: *The Journal of Physical Chemistry Letters* 11.7 (2020), 2422–2429. ISSN: 1948-7185. DOI: 10.1021/acs.jpclett.0c00329. URL: http://dx.doi.org/10.1021/acs.jpclett.0c00329.

[30] M Sakurai et al. “Molecular dynamics simulation of water between hydrophobic surfaces. Implication for the long-range hydrophobic force”. In: *Chemical Physics Letters* 289.5–6 (1998), 567–571. ISSN: 0009-2614. DOI: 10.1016/s0009-2614(98)00462-x. URL: http://dx.doi.org/10.1016/s0009-2614(98)00462-x.

[31] Michael Dennin. “Langmuir monolayers”. In: *Experimental and Computational Techniques in Soft Condensed Matter Physics*. Cambridge University Press, 2010, 97–120. DOI: 10.1017/cbo9780511760549.004. URL: http://dx.doi.org/10.1017/cbo9780511760549.004.

[32] Herman E. Ries and Hewson Swift. “Twisted double-layer ribbons and the mechanism for monolayer collapse”. In: *Langmuir* 3.5 (1987), 853–855. ISSN: 1520-5827. DOI: 10.1021/la00077a048. URL: http://dx.doi.org/10.1021/la00077a048.

[33] Ka Yee C. Lee. “Collapse Mechanisms of Langmuir Monolayers”. In: *Annual Review of Physical Chemistry* 59.1 (2008), 771–791. ISSN: 1545-1593. DOI: 10.1146/annurev.physchem.58.032806.104619. URL: http://dx.doi.org/10.1146/annurev.physchem.58.032806.104619.

[34] Jose Luis Fidalgo Rodriguez et al. “Phase transition beyond the monolayer collapse – The case of stearic acid spread at the air/water interface”. In: *Colloids and Surfaces A: Physicochemical and Engineering Aspects* 623 (2021), p. 126781. ISSN: 0927-7757. DOI: 10.1016/j.colsurfa.2021.126781. URL: http://dx.doi.org/10.1016/j.colsurfa.2021.126781.

[35] William D. Harkins, T. Fraser Young, and Edward Boyd. “The Thermodynamics of Films: Energy and Entropy of Extension and Spreading of Insoluble Monolayers”. In: *The Journal of Chemical Physics* 8.12 (1940), 954–965. ISSN: 1089-7690. DOI: 10.1063/1.1750610. URL: http://dx.doi.org/10.1063/1.1750610.

[36] Takamasa Ishikawa et al. "Maze Pattern at Nanometer-Scale in a Mixed Langmuir Monolayer of Fatty Acids". In: *Bulletin of the Chemical Society of Japan* 94.12 (2021), 2967–2969. ISSN: 1348-0634. DOI: 10.1246/bcsj.20210335. URL: http://dx.doi.org/10.1246/bcsj.20210335.

[37] Katharine B. Blodgett. "Films Built by Depositing Successive Monomolecular Layers on a Solid Surface". In: *Journal of the American Chemical Society* 57.6 (1935), 1007–1022. ISSN: 1520-5126. DOI: 10.1021/ja01309a011. URL: http://dx.doi.org/10.1021/ja01309a011.

[38] Gero Decher and Jong-Dal Hong. "Buildup of ultrathin multilayer films by a self-assembly process, 1 consecutive adsorption of anionic and cationic bipolar amphiphiles on charged surfaces". In: *Makromolekulare Chemie. Macromolecular Symposia* 46.1 (1991), 321–327. ISSN: 0258-0322. DOI: 10.1002/masy.19910460145. URL: http://dx.doi.org/10.1002/masy.19910460145.

[39] G. Decher, J.D. Hong, and J. Schmitt. "Buildup of ultrathin multilayer films by a self-assembly process: III. Consecutively alternating adsorption of anionic and cationic polyelectrolytes on charged surfaces". In: *Thin Solid Films* 210–211 (1992), 831–835. ISSN: 0040-6090. DOI: 10.1016/0040-6090(92)90417-a. URL: http://dx.doi.org/10.1016/0040-6090(92)90417-a.

[40] Masato Ito et al. "Hyper 100 °C Langmuir–Blodgett (Langmuir–Schaefer) Technique for Organized Ultrathin Film of Polymeric Semiconductors". In: *Langmuir* 38.17 (2021), 5237–5247. ISSN: 1520-5827. DOI: 10.1021/acs.langmuir.1c02596. URL: http://dx.doi.org/10.1021/acs.langmuir.1c02596.

[41] Issei Kitamura et al. "Photo-triggered large mass transport driven only by a photoresponsive surface skin layer". In: *Scientific Reports* 10.1 (2020). ISSN: 2045-2322. DOI: 10.1038/s41598-020-69605-8. URL: http://dx.doi.org/10.1038/s41598-020-69605-8.

[42] Ming Li et al. "AFM Studies of Solid-Supported Lipid Bilayers Formed at a Au(111) Electrode Surface Using Vesicle Fusion and a Combination of Langmuirâˆ'Blodgett and Langmuirâˆ'Schaefer Techniques". In: *Langmuir* 24.18 (2008), 10313â€"10323. ISSN: 1520-5827. DOI: 10.1021/la800800m. URL: http://dx.doi.org/10.1021/la800800m.

[43] James Kurniawan et al. “Preparation and Characterization of Solid-Supported Lipid Bilayers Formed by Langmuir–Blodgett Deposition: A Tutorial”. In: *Langmuir* 34.51 (2018), 15622–15639. ISSN: 1520-5827. DOI: 10.1021/acs.langmuir.8b03504. URL: http://dx.doi.org/10.1021/acs.langmuir.8b03504.

[44] K.J. Klopfer and T.K. Vanderlick. “Isotherms of Dipalmitoylphosphatidylcholine (DPPC) Monolayers: Features Revealed and Features Obscured”. In: *Journal of Colloid and Interface Science* 182.1 (1996), 220–229. ISSN: 0021-9797. DOI: 10.1006/jcis.1996.0454. URL: http://dx.doi.org/10.1006/jcis.1996.0454.

[45] Naoki Takeshita, Masanari Okuno, and Taka-aki Ishibashi. “Molecular conformation of DPPC phospholipid Langmuir and Langmuir–Blodgett monolayers studied by heterodyne-detected vibrational sum frequency generation spectroscopy”. In: *Physical Chemistry Chemical Physics* 19.3 (2017), 2060–2066. ISSN: 1463-9084. DOI: 10.1039/c6cp07800a. URL: http://dx.doi.org/10.1039/c6cp07800a.

[46] Yuh-Lang Lee, Jing-Yi Lin, and Chien-Hsiang Chang. “Thermodynamic characteristics and Langmuir–Blodgett deposition behavior of mixed DPPA/DPPC monolayers at air/liquid interfaces”. In: *Journal of Colloid and Interface Science* 296.2 (2006), 647–654. ISSN: 0021-9797. DOI: 10.1016/j.jcis.2005.09.050. URL: http://dx.doi.org/10.1016/j.jcis.2005.09.050.

[47] Adrià Botet-Carreras et al. “Characterization of monolayers and liposomes that mimic lipid composition of HeLa cells”. In: *Colloids and Surfaces B: Biointerfaces* 196 (2020), p. 111288. ISSN: 0927-7765. DOI: 10.1016/j.colsurfb.2020.111288. URL: http://dx.doi.org/10.1016/j.colsurfb.2020.111288.

[48] Alfred Blume. “A comparative study of the phase transitions of phospholipid bilayers and monolayers”. In: *Biochimica et Biophysica Acta (BBA) - Biomembranes* 557.1 (1979), 32–44. ISSN: 0005-2736. DOI: 10.1016/0005-2736(79)90087-7. URL: http://dx.doi.org/10.1016/0005-2736(79)90087-7.

[49] J. Miñones et al. “Interactions between membrane sterols and phospholipids in model mammalian and fungi cellular membranes — A Langmuir monolayer study”. In: *Biophysical Chemistry* 140.1–3 (2009),

69–77. ISSN: 0301-4622. DOI: 10.1016/j.bpc.2008.11.011. URL: http://dx.doi.org/10.1016/j.bpc.2008.11.011.

[50] Małgorzata Jurak. "Thermodynamic Aspects of Cholesterol Effect on Properties of Phospholipid Monolayers: Langmuir and Langmuir–Blodgett Monolayer Study". In: *The Journal of Physical Chemistry B* 117.13 (2013), 3496–3502. ISSN: 1520-5207. DOI: 10.1021/jp401182c. URL: http://dx.doi.org/10.1021/jp401182c.

[51] Justyna Mildner, Anita Wnetrzak, and Patrycja Dynarowicz-Latka. "Cholesterol and Cardiolipin Importance in Local Anesthetics–Membrane Interactions: The Langmuir Monolayer Study". In: *The Journal of Membrane Biology* 252.1 (2018), 31–39. ISSN: 1432-1424. DOI: 10.1007/s00232-018-0055-6. URL: http://dx.doi.org/10.1007/s00232-018-0055-6.

[52] H Mohwald. "Phospholipid and Phospholipid-Protein Monolayers at the Air/Water Interface". In: *Annual Review of Physical Chemistry* 41.1 (1990), 441–476. ISSN: 1545-1593. DOI: 10.1146/annurev.pc.41.100190.002301. URL: http://dx.doi.org/10.1146/annurev.pc.41.100190.002301.

[53] H BROCKMAN. "Lipid monolayers: why use half a membrane to characterize protein-membrane interactions?" In: *Current Opinion in Structural Biology* 9.4 (1999), 438–443. ISSN: 0959-440X. DOI: 10.1016/s0959-440x(99)80061-x. URL: http://dx.doi.org/10.1016/s0959-440x(99)80061-x.

[54] E.J. Grasso, R.G. Oliveira, and B. Maggio. "Surface interactions, thermodynamics and topography of binary monolayers of Insulin with dipalmitoylphosphatidylcholine and 1-palmitoyl-2-oleoylphosphatidylcholine at the air/water interface". In: *Journal of Colloid and Interface Science* 464 (2016), 264–276. ISSN: 0021-9797. DOI: 10.1016/j.jcis.2015.11.034. URL: http://dx.doi.org/10.1016/j.jcis.2015.11.034.

[55] Carlos M. N. Mendonça et al. "Understanding the interactions of imidazolium-based ionic liquids with cell membrane models". In: *Physical Chemistry Chemical Physics* 20.47 (2018), 29764–29777. ISSN: 1463-9084. DOI: 10.1039/c8cp05035j. URL: http://dx.doi.org/10.1039/c8cp05035j.

[56] Huie Zhu, Masaya Mitsuishi, and Tokuji Miyashita. “Facile Preparation of Highly Oriented Poly(vinylidene fluoride) Langmuir–Blodgett Nanofilms Assisted by Amphiphilic Polymer Nanosheets”. In: *Macromolecules* 45.22 (2012), 9076–9084. ISSN: 1520-5835. DOI: 10.1021/ma301711g. URL: http://dx.doi.org/10.1021/ma301711g.

[57] Rebeca da Rocha Rodrigues et al. “Conjugated polymers as Langmuir and Langmuir-Blodgett films: Challenges and applications in nanostructured devices”. In: *Advances in Colloid and Interface Science* 285 (2020), p. 102277. ISSN: 0001-8686. DOI: 10.1016/j.cis.2020.102277. URL: http://dx.doi.org/10.1016/j.cis.2020.102277.

[58] Niels Reitzel et al. “Self-Assembly of Conjugated Polymers at the Air/Water Interface. Structure and Properties of Langmuir and LangmuirBlodgett Films of Amphiphilic Regioregular Polythiophenes”. In: *Journal of the American Chemical Society* 122.24 (2000), 5788–5800. ISSN: 1520-5126. DOI: 10.1021/ja9924501. URL: http://dx.doi.org/10.1021/ja9924501.

[59] Masato Ito et al. “100 °C-Langmuir–Blodgett Method for Fabricating Highly Oriented, Ultrathin Films of Polymeric Semiconductors”. In: *ACS Applied Materials amp; Interfaces* 12.50 (2020), 56522–56529. ISSN: 1944-8252. DOI: 10.1021/acsami.0c18349. URL: http://dx.doi.org/10.1021/acsami.0c18349.

[60] Cheng Qian et al. “Facile preparation of self-assembled black phosphorus-based composite LB films as new chemical gas sensors”. In: *Colloids and Surfaces A: Physicochemical and Engineering Aspects* 608 (2021), p. 125616. ISSN: 0927-7757. DOI: 10.1016/j.colsurfa.2020.125616. URL: http://dx.doi.org/10.1016/j.colsurfa.2020.125616.

[61] Katsuhiko Ariga and Rawil Fakhrullin. “Materials Nanoarchitectonics from Atom to Living Cell: A Method for Everything”. In: *Bulletin of the Chemical Society of Japan* 95.5 (2022), 774–795. ISSN: 1348-0634. DOI: 10.1246/bcsj.20220071. URL: http://dx.doi.org/10.1246/bcsj.20220071.

[62] Somsubhra Saha, Manash Ghosh, and Joydeep Chowdhury. “Infused self-assembly on Langmuir–Blodgett Film: Fabrication of highly efficient SERS active substrates with controlled plasmonic aggregates”. In: *Journal of Raman Spectroscopy* 50.3 (2018), 330–344. ISSN: 1097-

4555. DOI: 10.1002/jrs.5529. URL: http://dx.doi.org/10.1002/jrs.5529.

[63] Denis M. Krichevsky et al. "Resonant Plasmon-Enhanced Absorption of Charge Transfer Complexes in a Metal–Organic Monolayer". In: *Advanced Optical Materials* 9.11 (2021). ISSN: 2195-1071. DOI: 10.1002/adom.202100065. URL: http://dx.doi.org/10.1002/adom.202100065.

[64] Joydeep Chowdhury, Somsubhra Saha, and Manash Ghosh. "Self-assembly of metal nanocolloids entrapped in Langmuir Blodgett Film templates: Evidence of efficient SERS sensing platforms". In: *Materials Today: Proceedings* 5.3 (2018), 10071–10076. ISSN: 2214-7853. DOI: 10.1016/j.matpr.2017.11.001. URL: http://dx.doi.org/10.1016/j.matpr.2017.11.001.

[65] Soraya Sangiao et al. "All-Carbon Electrode Molecular Electronic Devices Based on Langmuir–Blodgett Monolayers". In: *Small* 13.7 (2016). ISSN: 1613-6829. DOI: 10.1002/smll.201603207. URL: http://dx.doi.org/10.1002/smll.201603207.

[66] Thiers M. Uehara et al. "Nanostructured scaffolds containing graphene oxide for nanomedicine applications". In: *Polymers for Advanced Technologies* 33.2 (2021), 591–600. ISSN: 1099-1581. DOI: 10.1002/pat.5541. URL: http://dx.doi.org/10.1002/pat.5541.

[67] Alowasheeir Azhar et al. "Nanoarchitectonics: A New Materials Horizon for Prussian Blue and Its Analogues". In: *Bulletin of the Chemical Society of Japan* 92.4 (2019), 875–904. ISSN: 1348-0634. DOI: 10.1246/bcsj.20180368. URL: http://dx.doi.org/10.1246/bcsj.20180368.

[68] Bin Bai, Dong Wang, and Li-Jun Wan. "Synthesis of Covalent Organic Framework Films at Interfaces". In: *Bulletin of the Chemical Society of Japan* 94.3 (2021), 1090–1098. ISSN: 1348-0634. DOI: 10.1246/bcsj.20200391. URL: http://dx.doi.org/10.1246/bcsj.20200391.

[69] Fábio A. Scholl et al. "Exploring Langmuir-Blodgett films with phospholipid-graphene oxide/MnO2 as a hybrid nanostructured interface for supercapacitor applications". In: *Colloids and Surfaces A: Physicochemical and Engineering Aspects* 664 (2023), p. 131128. ISSN: 0927-7757. DOI:

10.1016/j.colsurfa.2023.131128. URL: http://dx.doi.org/10.1016/j.colsurfa.2023.131128.

[70] Katsuhiko Ariga. "Interfaces Working for Biology: Solving Biological Mysteries and Opening Up Future Nanoarchitectonics". In: *ChemNanoMat* 2.5 (2016), 333–343. ISSN: 2199-692X. DOI: 10.1002/cnma.201600053. URL: http://dx.doi.org/10.1002/cnma.201600053.

[71] Jingwen Song, Xiaofang Jia, and Katsuhiko Ariga. "Methods with Nanoarchitectonics for Small Molecules and Nanostructures to Regulate Living Cells". In: *Small Methods* 4.10 (2020). ISSN: 2366-9608. DOI: 10.1002/smtd.202000500. URL: http://dx.doi.org/10.1002/smtd.202000500.

[72] Luciano Caseli et al. "Effect of Molecular Surface Packing on the Enzymatic Activity Modulation of an Anchored Protein on Phospholipid Langmuir Monolayers". In: *Langmuir* 21.9 (2005), 4090–4095. ISSN: 1520-5827. DOI: 10.1021/la047292s. URL: http://dx.doi.org/10.1021/la047292s.

[73] Régine Maget-Dana. "The monolayer technique: a potent tool for studying the interfacial properties of antimicrobial and membrane-lytic peptides and their interactions with lipid membranes". In: *Biochimica et Biophysica Acta (BBA) - Biomembranes* 1462.1–2 (1999), 109–140. ISSN: 0005-2736. DOI: 10.1016/s0005-2736(99)00203-5. URL: http://dx.doi.org/10.1016/s0005-2736(99)00203-5.

[74] Stefanie Roes, Ulrich Seydel, and Thomas Gutsmann. "Probing the Properties of Lipopolysaccharide Monolayers and Their Interaction with the Antimicrobial Peptide Polymyxin B by Atomic Force Microscopy". In: *Langmuir* 21.15 (2005), 6970–6978. ISSN: 1520-5827. DOI: 10.1021/la048218c. URL: http://dx.doi.org/10.1021/la048218c.

[75] Fatma Gür et al. "Preparation of bio-electrodes via Langmuir-Blodgett technique for pharmaceutical and waste industries and their biosensor application". In: *Colloids and Surfaces A: Physicochemical and Engineering Aspects* 583 (2019), p. 124005. ISSN: 0927-7757. DOI: 10.1016/j.colsurfa.2019.124005. URL: http://dx.doi.org/10.1016/j.colsurfa.2019.124005.

[76] Anna Vikulina et al. "A lipid membrane supported on an artificial extracellular matrix made of polyelectrolyte multilayers: towards nanoarchitectonics at the cellular interface". In: *Nanoscale* 15.5 (2023), 2197–2205. ISSN: 2040-3372. DOI: 10.1039/d2nr05186a. URL: http://dx.doi.org/10.1039/d2nr05186a.

[77] M. S. Kim et al. "Fabricating multifunctional nanoparticle membranes by a fast layer-by-layer Langmuir–Blodgett process: application in lithium–sulfur batteries". In: *Journal of Materials Chemistry A* 4.38 (2016), 14709–14719. ISSN: 2050-7496. DOI: 10.1039/c6ta06018h. URL: http://dx.doi.org/10.1039/c6ta06018h.

[78] Ran Wang et al. "Self-Assembled Black Phosphorus-Based Composite Langmuir–Blodgett Films with an Enhanced Photocurrent Generation Capability and Surface-Enhanced Raman Scattering Properties". In: *ACS Omega* 6.6 (2021), 4430–4439. ISSN: 2470-1343. DOI: 10.1021/acsomega.0c05832. URL: http://dx.doi.org/10.1021/acsomega.0c05832.

[79] C. Medina-Plaza, J.A. de Saja, and M.L. Rodriguez-Mendez. "Bioelectronic tongue based on lipidic nanostructured layers containing phenol oxidases and lutetium bisphthalocyanine for the analysis of grapes". In: *Biosensors and Bioelectronics* 57 (2014), 276–283. ISSN: 0956-5663. DOI: 10.1016/j.bios.2014.02.023. URL: http://dx.doi.org/10.1016/j.bios.2014.02.023.

[80] Luciano Caseli et al. "Fabrication of Phytic Acid Sensor Based on Mixed Phytaseâˆ'Lipid Langmuirâˆ'Blodgett Films". In: *Langmuir* 22.20 (2006), 8501â€"8508. ISSN: 1520-5827. DOI: 10.1021/la061799g. URL: http://dx.doi.org/10.1021/la061799g.

[81] Raul T. Rodrigues et al. "Carbon Nanotubes and Algal Polysaccharides To Enhance the Enzymatic Properties of Urease in Lipid Langmuir–Blodgett Films". In: *Langmuir* 34.9 (2018), 3082–3093. ISSN: 1520-5827. DOI: 10.1021/acs.langmuir.7b04317. URL: http://dx.doi.org/10.1021/acs.langmuir.7b04317.

[82] Katsuhiko Ariga. "Materials nanoarchitectonics in a two-dimensional world within a nanoscale distance from the liquid phase". In: *Nanoscale* 14.30 (2022), 10610–10629. ISSN: 2040-3372. DOI: 10.1039/d2nr02513b. URL: http://dx.doi.org/10.1039/d2nr02513b.

[83] Katsuhiko Ariga and Yusuke Yamauchi. “Nanoarchitectonics from Atom to Life”. In: *Chemistry – An Asian Journal* 15.6 (2020), 718–728. ISSN: 1861-471X. DOI: 10.1002/asia.202000106. URL: http://dx.doi.org/10.1002/asia.202000106.

[84] Katsuhiko Ariga et al. “Nanoarchitectonics beyond Self-Assembly: Challenges to Create Bio-Like Hierarchic Organization”. In: *Angewandte Chemie International Edition* 59.36 (2020), 15424–15446. ISSN: 1521-3773. DOI: 10.1002/anie.202000802. URL: http://dx.doi.org/10.1002/anie.202000802.

[85] Takatoshi Maeda et al. “Vortex Flow-controlled Circularly Polarized Luminescence of Achiral Pt(II) Complex Aggregates Assembled at the Air-Water Interface”. In: *Small Methods* 6.12 (2022). ISSN: 2366-9608. DOI: 10.1002/smtd.202200936. URL: http://dx.doi.org/10.1002/smtd.202200936.

[86] Taizo Mori et al. “Carbon Nanosheets by Morphology-Retained Carbonization of Two-Dimensional Assembled Anisotropic Carbon Nanorings”. In: *Angewandte Chemie* 130.31 (2018), 9827–9831. ISSN: 1521-3757. DOI: 10.1002/ange.201803859. URL: http://dx.doi.org/10.1002/ange.201803859.

[87] Peng Xiao et al. “Ultrafast Formation of Free-Standing 2D Carbon Nanotube Thin Films through Capillary Force Driving Compression on an Air/Water Interface”. In: *Chemistry of Materials* 28.19 (2016), 7125–7133. ISSN: 1520-5002. DOI: 10.1021/acs.chemmater.6b03420. URL: http://dx.doi.org/10.1021/acs.chemmater.6b03420.

[88] Jingwen Song et al. “Fullerphene Nanosheets: A Bottom-Up 2D Material for Single-Carbon-Atom-Level Molecular Discrimination”. In: *Advanced Materials Interfaces* 9.11 (2022). ISSN: 2196-7350. DOI: 10.1002/admi.202102241. URL: http://dx.doi.org/10.1002/admi.202102241.

[89] Donghui Guo et al. “Active sites of nitrogen-doped carbon materials for oxygen reduction reaction clarified using model catalysts”. In: *Science* 351.6271 (2016), 361–365. ISSN: 1095-9203. DOI: 10.1126/science.aad0832. URL: http://dx.doi.org/10.1126/science.aad0832.

[90] In: *POLYMERS* 73.3 (2024), 95–95. ISSN: 2185-9825. DOI: 10.1295/kobunshi.73.3_95. URL: http://dx.doi.org/10.1295/kobunshi.73.3_95.

[91] K. Miyazawa et al. "C60 Nanowhiskers Formed by the Liquid–liquid Interfacial Precipitation Method". In: *Journal of Materials Research* 17.1 (2002), 83–88. ISSN: 2044-5326. DOI: 10.1557/jmr.2002.0014. URL: http://dx.doi.org/10.1557/jmr.2002.0014.

[92] Guoping Chen et al. "Hollow Spherical Fullerene Obtained by Kinetically Controlled Liquid-Liquid Interfacial Precipitation". In: *Chemistry – An Asian Journal* 17.20 (2022). ISSN: 1861-471X. DOI: 10.1002/asia.202200756. URL: http://dx.doi.org/10.1002/asia.202200756.

[93] Jeffrey T. Culp et al. "Supramolecular Assembly at Interfaces: Formation of an Extended Two-Dimensional Coordinate Covalent Square Grid Network at the AirWater Interface". In: *Journal of the American Chemical Society* 124.34 (2002), 10083–10090. ISSN: 1520-5126. DOI: 10.1021/ja026312e. URL: http://dx.doi.org/10.1021/ja026312e.

[94] Junya Adachi et al. "Coordination Amphiphile: Design of Planar-Coordinated Platinum Complexes for Monolayer Formation at an Air-Water Interface Based on Ligand Characteristics and Molecular Topology". In: *Bulletin of the Chemical Society of Japan* 95.6 (2022), 889–897. ISSN: 1348-0634. DOI: 10.1246/bcsj.20220086. URL: http://dx.doi.org/10.1246/bcsj.20220086.

[95] Guoping Chen, Lok Kumar Shrestha, and Katsuhiko Ariga. "Zero-to-Two Nanoarchitectonics: Fabrication of Two-Dimensional Materials from Zero-Dimensional Fullerene". In: *Molecules* 26.15 (2021), p. 4636. ISSN: 1420-3049. DOI: 10.3390/molecules26154636. URL: http://dx.doi.org/10.3390/molecules26154636.

[96] Katsuhiko Ariga. "Molecular Tuning Nanoarchitectonics for Molecular Recognition and Molecular Manipulation". In: *ChemNanoMat* 6.6 (2020), 870–880. ISSN: 2199-692X. DOI: 10.1002/cnma.202000137. URL: http://dx.doi.org/10.1002/cnma.202000137.

[97] Katsuhiko Ariga. “Molecular recognition at the air–water interface: nanoarchitectonic design and physicochemical understanding”. In: *Physical Chemistry Chemical Physics* 22.43 (2020), 24856–24869. ISSN: 1463-9084. DOI: 10.1039/d0cp04174b. URL: http://dx.doi.org/10.1039/d0cp04174b.

[98] Kazue Kurihara. “Langmuir-Blodgett films in advanced molecular engineering”. In: *Colloids and Surfaces A: Physicochemical and Engineering Aspects* 123–124 (1997), 425–432. ISSN: 0927-7757. DOI: 10.1016/s0927-7757(96)03831-9. URL: http://dx.doi.org/10.1016/s0927-7757(96)03831-9.

[99] Yuri Lvov, Katsuhiko Ariga, and Toyoki Kunitake. “Layer-by-Layer Assembly of Alternate Protein/Polyion Ultrathin Films”. In: *Chemistry Letters* 23.12 (1994), 2323–2326. ISSN: 1348-0715. DOI: 10.1246/cl.1994.2323. URL: http://dx.doi.org/10.1246/cl.1994.2323.

[100] Mohamed B. Zakaria et al. “Self-Construction from 2D to 3D: One-Pot Layer-by-Layer Assembly of Graphene Oxide Sheets Held Together by Coordination Polymers”. In: *Angewandte Chemie International Edition* 55.29 (2016), 8426–8430. ISSN: 1521-3773. DOI: 10.1002/anie.201603223. URL: http://dx.doi.org/10.1002/anie.201603223.

[101] Katsuhiko Ariga, Yuri Lvov, and Gero Decher. “There is still plenty of room for layer-by-layer assembly for constructing nanoarchitectonics-based materials and devices”. In: *Physical Chemistry Chemical Physics* 24.7 (2022), 4097–4115. ISSN: 1463-9084. DOI: 10.1039/d1cp04669a. URL: http://dx.doi.org/10.1039/d1cp04669a.

[102] Joseph J. Richardson et al. “Innovation in Layer-by-Layer Assembly”. In: *Chemical Reviews* 116.23 (2016), 14828–14867. ISSN: 1520-6890. DOI: 10.1021/acs.chemrev.6b00627. URL: http://dx.doi.org/10.1021/acs.chemrev.6b00627.

[103] Jongwook Kim et al. “Shape-Changing DNA-Linked Nanoparticle Films Dictated by Lateral and Vertical Patterns”. In: *Advanced Materials* 34.13 (2022). ISSN: 1521-4095. DOI: 10.1002/adma.202109091. URL: http://dx.doi.org/10.1002/adma.202109091.

[104] Makoto Komiyama et al. “Chemistry Can Make Strict and Fuzzy Controls for Bio-Systems: DNA Nanoarchitectonics and Cell-Macromolecular Nanoarchitectonics”. In: *Bulletin of the Chemical Society of Japan*

90.9 (2017), 967–1004. ISSN: 1348-0634. DOI: 10.1246/bcsj.20170156. URL: http://dx.doi.org/10.1246/bcsj.20170156.

[105] Xingguo Liang et al. "Dynamism of Supramolecular DNA/RNA Nanoarchitectonics: From Interlocked Structures to Molecular Machines". In: *Bulletin of the Chemical Society of Japan* 93.4 (2020), 581–603. ISSN: 1348-0634. DOI: 10.1246/bcsj.20200012. URL: http://dx.doi.org/10.1246/bcsj.20200012.

[106] Sanjay Kosara, Ramesh Singh, and Dhiraj Bhatia. "Structural DNA nanotechnology at the nexus of next-generation bio-applications: challenges and perspectives". In: *Nanoscale Advances* 6.2 (2024), 386–401. ISSN: 2516-0230. DOI: 10.1039/d3na00692a. URL: http://dx.doi.org/10.1039/d3na00692a.

[107] Muhammad Haseeb Iqbal et al. "Brush-Induced Orientation of Collagen Fibers in Layer-by-Layer Nanofilms: A Simple Method for the Development of Human Muscle Fibers". In: *ACS Nano* 16.12 (2022), 20034–20043. ISSN: 1936-086X. DOI: 10.1021/acsnano.2c06329. URL: http://dx.doi.org/10.1021/acsnano.2c06329.

[108] Katsuhiko Ariga et al. "Materials Nanoarchitectonics as Cell Regulators". In: *ChemNanoMat* 5.6 (2019), 692–702. ISSN: 2199-692X. DOI: 10.1002/cnma.201900207. URL: http://dx.doi.org/10.1002/cnma.201900207.

[109] Gaulthier Rydzek et al. "Electrochemical nanoarchitectonics and layer-by-layer assembly: From basics to future". In: *Nano Today* 10.2 (2015), 138–167. ISSN: 1748-0132. DOI: 10.1016/j.nantod.2015.02.008. URL: http://dx.doi.org/10.1016/j.nantod.2015.02.008.

[110] Yunzhe Jiang et al. "Nanoporous Films with Oriented Arrays of Molecular Motors for Photoswitching the Guest Adsorption and Diffusion". In: *Angewandte Chemie International Edition* 62.3 (2022). ISSN: 1521-3773. DOI: 10.1002/anie.202214202. URL: http://dx.doi.org/10.1002/anie.202214202.

[111] Katsuhiko Ariga et al. "Nanoarchitectonics for Dynamic Functional Materials from Atomic-/Molecular-Level Manipulation to Macroscopic Action". In: *Advanced Materials* 28.6 (2015), 1251–1286. ISSN: 1521-4095. DOI: 10.1002/adma.201502545. URL: http://dx.doi.org/10.1002/adma.201502545.

[112] Rui Zheng et al. “The Growth Mechanism of a Conductive MOF Thin Film in Spray-based Layer-by-layer Liquid Phase Epitaxy”. In: *Angewandte Chemie* 134.43 (2022). ISSN: 1521-3757. DOI: 10.1002/ange.202212797. URL: http://dx.doi.org/10.1002/ange.202212797.

[113] Taizo Mori et al. “Molecular rotors confined at an ordered 2D interface”. In: *Physical Chemistry Chemical Physics* 20.5 (2018), 3073–3078. ISSN: 1463-9084. DOI: 10.1039/c7cp04256f. URL: http://dx.doi.org/10.1039/c7cp04256f.

[114] Katsuhiko Ariga. “The evolution of molecular machines through interfacial nanoarchitectonics: from toys to tools”. In: *Chemical Science* 11.39 (2020), 10594–10604. ISSN: 2041-6539. DOI: 10.1039/d0sc03164j. URL: http://dx.doi.org/10.1039/d0sc03164j.

[115] Katsuhiko Ariga, Masaki Ishii, and Taizo Mori. “Interfacial nanoarchitectonics for molecular manipulation and molecular machine operation”. In: *Current Opinion in Colloid amp; Interface Science* 44 (2019), 1–13. ISSN: 1359-0294. DOI: 10.1016/j.cocis.2019.08.004. URL: http://dx.doi.org/10.1016/j.cocis.2019.08.004.

[116] Katsuhiko Ariga. “Liquid interfacial nanoarchitectonics: Molecular machines, organic semiconductors, nanocarbons, stem cells, and others”. In: *Current Opinion in Colloid amp; Interface Science* 63 (2023), p. 101656. ISSN: 1359-0294. DOI: 10.1016/j.cocis.2022.101656. URL: http://dx.doi.org/10.1016/j.cocis.2022.101656.

[117] Maïssa K. S. Barr et al. “Solution Atomic Layer Deposition of Smooth, Continuous, Crystalline Metal–Organic Framework Thin Films”. In: *Chemistry of Materials* 34.22 (2022), 9836–9843. ISSN: 1520-5002. DOI: 10.1021/acs.chemmater.2c01102. URL: http://dx.doi.org/10.1021/acs.chemmater.2c01102.

[118] Jorid Smets et al. “Molecular Layer Deposition of Zeolitic Imidazolate Framework-8 Films”. In: *Chemistry of Materials* 35.4 (2023), 1684–1690. ISSN: 1520-5002. DOI: 10.1021/acs.chemmater.2c03439. URL: http://dx.doi.org/10.1021/acs.chemmater.2c03439.

[119] Geoffrey Barnes and Ian Gentle. *Interfacial Science: An Introduction*. Oxford University Press, 2011. ISBN: 9780198905509. DOI: 10.1093/hesc/9780199571185.001.0001. URL: http://dx.doi.org/10.1093/hesc/9780199571185.001.0001.

[120] James P. Bareman and Michael L. Klein. "Molecular Dynamics Simulation of A Langmuir Monolayer". In: *MRS Proceedings* 237 (1991). ISSN: 1946-4274. DOI: 10.1557/proc-237-271. URL: http://dx.doi.org/10.1557/proc-237-271.

[121] Zhenglong Chen et al. "Determination of rheology and surface tension of airway surface liquid: a review of clinical relevance and measurement techniques". In: *Respiratory Research* 20.1 (2019). ISSN: 1465-993X. DOI: 10.1186/s12931-019-1229-1. URL: http://dx.doi.org/10.1186/s12931-019-1229-1.

[122] D. G. Dervichian. "Changes of Phase and Transformations of Higher Order in Monolayers". In: *The Journal of Chemical Physics* 7.10 (1939), 931–948. ISSN: 1089-7690. DOI: 10.1063/1.1750347. URL: http://dx.doi.org/10.1063/1.1750347.

[123] A. Fischer et al. "On the nature of the lipid monolayer phase transition". In: *Journal de Physique Lettres* 45.16 (1984), 785–791. ISSN: 0302-072X. DOI: 10.1051/jphyslet:019840045016078500. URL: http://dx.doi.org/10.1051/jphyslet:019840045016078500.

[124] C.A. Helm et al. "Phospholipid monolayers between fluid and solid states". In: *Biophysical Journal* 52.3 (1987), 381–390. ISSN: 0006-3495. DOI: 10.1016/s0006-3495(87)83226-5. URL: http://dx.doi.org/10.1016/s0006-3495(87)83226-5.

[125] George L. Gaines. "Thermodynamic relationships for mixed insoluble monolayers". In: *Journal of Colloid and Interface Science* 21.3 (1966), 315–319. ISSN: 0021-9797. DOI: 10.1016/0095-8522(66)90015-8. URL: http://dx.doi.org/10.1016/0095-8522(66)90015-8.

[126] Xiao Cha, Katsuhiko Ariga, and Toyoki Kunitake. "Multi-Site Binding of Aqueous Dipeptides by Mixed Monolayers at the Air-Water Interface". In: *Chemistry Letters* 25.1 (1996), 73–74. ISSN: 1348-0715. DOI: 10.1246/cl.1996.73. URL: http://dx.doi.org/10.1246/cl.1996.73.

[127] Yasuhiro Ikeura, Kazue Kurihara, and Toyoki Kunitake. "Molecular recognition at the air-water interface. Specific binding of nitrogen aromatics and amino acids by monolayers of long-chain derivatives of Kemp's acid". In: *Journal of the American Chemical Society* 113.19 (1991), 7342–7350. ISSN: 1520-5126. DOI: 10.1021/ja00019a035. URL: http://dx.doi.org/10.1021/ja00019a035.

[128] K Kurihara et al. “Binding of sugars and water-soluble polymers to a monolayer of cyclic resorcinol tetramer at the air-water interface”. In: *Thin Solid Films* 179.1–2 (1989), 21–26. ISSN: 0040-6090. DOI: 10.1016/0040-6090(89)90160-0. URL: http://dx.doi.org/10.1016/0040-6090(89)90160-0.

[129] Philippe Calvez et al. “Parameters modulating the maximum insertion pressure of proteins and peptides in lipid monolayers”. In: *Biochimie* 91.6 (2009), 718–733. ISSN: 0300-9084. DOI: 10.1016/j.biochi.2009.03.018. URL: http://dx.doi.org/10.1016/j.biochi.2009.03.018.

[130] S. R. Derkach, J. Krägel, and R. Miller. “Methods of measuring rheological properties of interfacial layers (Experimental methods of 2D rheology)”. In: *Colloid Journal* 71.1 (2009), 1–17. ISSN: 1608-3067. DOI: 10.1134/s1061933x09010013. URL: http://dx.doi.org/10.1134/s1061933x09010013.

[131] M. Karbaschi et al. “Rheology of interfacial layers”. In: *Current Opinion in Colloid amp; Interface Science* 19.6 (2014), 514–519. ISSN: 1359-0294. DOI: 10.1016/j.cocis.2014.08.003. URL: http://dx.doi.org/10.1016/j.cocis.2014.08.003.

[132] Leon F. Scherz et al. “Molecularly Designed Interfacial Viscoelasticity by Dendronized Polymers: From Flexible Macromolecules to Colloidal Objects”. In: *ACS Nano* 13.12 (2019), 14217–14229. ISSN: 1936-086X. DOI: 10.1021/acsnano.9b07142. URL: http://dx.doi.org/10.1021/acsnano.9b07142.

[133] Sven Reynaert et al. “Analysis of the magnetic rod interfacial stress rheometer”. In: *Journal of Rheology* 52.1 (2008), 261–285. ISSN: 1520-8516. DOI: 10.1122/1.2798238. URL: http://dx.doi.org/10.1122/1.2798238.

[134] D. Langevin. “Surface shear rheology of monolayers at the surface of water”. In: *Advances in Colloid and Interface Science* 207 (2014), 121–130. ISSN: 0001-8686. DOI: 10.1016/j.cis.2013.10.030. URL: http://dx.doi.org/10.1016/j.cis.2013.10.030.

[135] Carlton F. Brooks et al. “An Interfacial Stress Rheometer To Study Rheological Transitions in Monolayers at the AirWater Interface”. In: *Langmuir* 15.7 (1999), 2450–2459. ISSN: 1520-5827. DOI: 10.1021/la980465r. URL: http://dx.doi.org/10.1021/la980465r.

[136] Eduardo Guzmán et al. "DPPC–DOPC Langmuir monolayers modified by hydrophilic silica nanoparticles: Phase behaviour, structure and rheology". In: *Colloids and Surfaces A: Physicochemical and Engineering Aspects* 413 (2012), 174–183. ISSN: 0927-7757. DOI: 10.1016/j.colsurfa.2011.12.059. URL: http://dx.doi.org/10.1016/j.colsurfa.2011.12.059.

[137] J.K. Basu and M.K. Sanyal. "Ordering and growth of Langmuir–Blodgett films: X-ray scattering studies". In: *Physics Reports* 363.1 (2002), 1–84. ISSN: 0370-1573. DOI: 10.1016/s0370-1573(01)00083-7. URL: http://dx.doi.org/10.1016/s0370-1573(01)00083-7.

[138] S. Hénon and J. Meunier. "Microscope at the Brewster angle: Direct observation of first-order phase transitions in monolayers". In: *Review of Scientific Instruments* 62.4 (1991), 936–939. ISSN: 1089-7623. DOI: 10.1063/1.1142032. URL: http://dx.doi.org/10.1063/1.1142032.

[139] J. Meunier. "Why a Brewster angle microscope?" In: *Colloids and Surfaces A: Physicochemical and Engineering Aspects* 171.1–3 (2000), 33–40. ISSN: 0927-7757. DOI: 10.1016/s0927-7757(99)00555-5. URL: http://dx.doi.org/10.1016/s0927-7757(99)00555-5.

[140] Weiam Daear, Mark Mahadeo, and Elmar J. Prenner. "Applications of Brewster angle microscopy from biological materials to biological systems". In: *Biochimica et Biophysica Acta (BBA) - Biomembranes* 1859.10 (2017), 1749–1766. ISSN: 0005-2736. DOI: 10.1016/j.bbamem.2017.06.016. URL: http://dx.doi.org/10.1016/j.bbamem.2017.06.016.

[141] Cristina Roldán-Carmona et al. "Revisiting the Brewster Angle Microscopy: The relevance of the polar headgroup". In: *Advances in Colloid and Interface Science* 173 (2012), 12–22. ISSN: 0001-8686. DOI: 10.1016/j.cis.2012.02.002. URL: http://dx.doi.org/10.1016/j.cis.2012.02.002.

[142] Lucile Gambut et al. "Ellipsometry, Brewster Angle Microscopy, and Thermodynamic Studies of Monomolecular Films of Cryptophanes at the AirWater Interface". In: *Langmuir* 12.22 (1996), 5407–5412. ISSN: 1520-5827. DOI: 10.1021/la960245i. URL: http://dx.doi.org/10.1021/la960245i.

[143] Benjamin L. Stottrup, Andrew H. Nguyen, and Erkan Tüzel. "Taking another look with fluorescence microscopy: Image processing techniques in Langmuir monolayers for the twenty-first century". In: *Biochimica et Biophysica Acta (BBA) - Biomembranes* 1798.7 (2010), 1289–1300. ISSN: 0005-2736. DOI: 10.1016/j.bbamem.2010.01.003. URL: http://dx.doi.org/10.1016/j.bbamem.2010.01.003.

[144] Brian Moore et al. "Studies of phase transitions in Langmuir monolayers by fluorescence microscopy". In: *Journal of the Chemical Society, Faraday Transactions 2* 82.10 (1986), p. 1753. ISSN: 0300-9238. DOI: 10.1039/f29868201753. URL: http://dx.doi.org/10.1039/f29868201753.

[145] K. Kjaer et al. "Ordering in Lipid Monolayers Studied by Synchrotron X-Ray Diffraction and Fluorescence Microscopy". In: *Physical Review Letters* 58.21 (1987), 2224–2227. ISSN: 0031-9007. DOI: 10.1103/physrevlett.58.2224. URL: http://dx.doi.org/10.1103/physrevlett.58.2224.

[146] Annia H. Kycia et al. "Atomic Force Microscopy Studies of a Floating-Bilayer Lipid Membrane on a Au(111) Surface Modified with a Hydrophilic Monolayer". In: *Langmuir* 27.17 (2011), 10867–10877. ISSN: 1520-5827. DOI: 10.1021/la2016269. URL: http://dx.doi.org/10.1021/la2016269.

[147] Carlos Marcuello et al. "Langmuir–Blodgett Procedure to Precisely Control the Coverage of Functionalized AFM Cantilevers for SMFS Measurements: Application with Cellulose Nanocrystals". In: *Langmuir* 34.32 (2018), 9376–9386. ISSN: 1520-5827. DOI: 10.1021/acs.langmuir.8b01892. URL: http://dx.doi.org/10.1021/acs.langmuir.8b01892.

[148] Changxin Zhu et al. "Morphology of monolayer LB film by STM". In: *Vacuum* 43.11 (1992), 1111–1113. ISSN: 0042-207X. DOI: 10.1016/0042-207x(92)90345-w. URL: http://dx.doi.org/10.1016/0042-207x(92)90345-w.

[149] Kazue Kurihara et al. "Elemental Analysis of Langmuir-Blodgett Films by X-ray Photoelectron Spectroscopy". In: *Langmuir* 11.4 (1995), 1408–1411. ISSN: 1520-5827. DOI: 10.1021/la00004a057. URL: http://dx.doi.org/10.1021/la00004a057.

[150] P. Guyot-Sionnest, J. H. Hunt, and Y. R. Shen. "Sum-frequency vibrational spectroscopy of a Langmuir film: Study of molecular orientation of a two-dimensional system". In: *Physical Review Letters* 59.14 (1987), 1597–1600. ISSN: 0031-9007. DOI: 10.1103/physrevlett.59.1597. URL: http://dx.doi.org/10.1103/physrevlett.59.1597.

[151] Nobutaka Shioya et al. "Multiple-angle incidence resolution spectrometry: applications in nanoarchitectonics and applied physics". In: *Japanese Journal of Applied Physics* 63.6 (2024), p. 060102. ISSN: 1347-4065. DOI: 10.35848/1347-4065/ad4ad8. URL: http://dx.doi.org/10.35848/1347-4065/ad4ad8.

[152] Miharu Eguchi et al. "Adsorchromism: Molecular Nanoarchitectonics at 2D Nanosheets—Old Chemistry for Advanced Chromism". In: *Advanced Science* 8.14 (2021). ISSN: 2198-3844. DOI: 10.1002/advs.202100539. URL: http://dx.doi.org/10.1002/advs.202100539.

[153] Andrei Sakai, Laura O. Péres, and Luciano Caseli. "Langmuir and Langmuir-Blodgett films of Cl-PPV mixed with stearic acid: implication of the morphology on the surface and spectroscopy properties". In: *Colloid and Polymer Science* 293.3 (2014), 883–890. ISSN: 1435-1536. DOI: 10.1007/s00396-014-3477-4. URL: http://dx.doi.org/10.1007/s00396-014-3477-4.

[154] Diogo Volpati et al. "Vibrational spectroscopy for probing molecular-level interactions in organic films mimicking biointerfaces". In: *Advances in Colloid and Interface Science* 207 (2014), 199–215. ISSN: 0001-8686. DOI: 10.1016/j.cis.2014.01.014. URL: http://dx.doi.org/10.1016/j.cis.2014.01.014.

[155] D. Blaudez et al. "Polarization modulation FTIR spectroscopy at the air-water interface". In: *Thin Solid Films* 242.1–2 (1994), 146–150. ISSN: 0040-6090. DOI: 10.1016/0040-6090(94)90519-3. URL: http://dx.doi.org/10.1016/0040-6090(94)90519-3.

[156] Luciano Caseli et al. "Investigation of the Conformational Changes of a Conducting Polymer in Gas Sensor Active Layers by Means of Polarization-Modulation Infrared Reflection Absorption Spectroscopy (PM-IRRAS)." In: *Langmuir* 29.8 (2013), 2640–2645. ISSN: 1520-5827. DOI: 10.1021/la3050797. URL: http://dx.doi.org/10.1021/la3050797.

[157] Christophe Daniel and Margherita Lardone. "Characterization of Syndiotactic Polystyrene Nanofilms by PM-IRRAS Spectroscopy". In: *Macromolecular Symposia* 359.1 (2016), 24–31. ISSN: 1521-3900. DOI: 10.1002/masy.201500073. URL: http://dx.doi.org/10.1002/masy.201500073.

[158] D. Blaudez et al. "Polarization-Modulated FT-IR Spectroscopy of a Spread Monolayer at the Air/Water Interface". In: *Applied Spectroscopy* 47.7 (1993), 869–874. ISSN: 1943-3530. DOI: 10.1366/0003702934415273. URL: http://dx.doi.org/10.1366/0003702934415273.

[159] Fumiko Kimura, Junzo Umemura, and Tohru Takenaka. "FTIR-ATR studies on Langmuir-Blodgett films of stearic acid with 1-9 monolayers". In: *Langmuir* 2.1 (1986), 96–101. ISSN: 1520-5827. DOI: 10.1021/la00067a017. URL: http://dx.doi.org/10.1021/la00067a017.

[160] Yuwei Liu, Joshua Jasensky, and Zhan Chen. "Molecular Interactions of Proteins and Peptides at Interfaces Studied by Sum Frequency Generation Vibrational Spectroscopy". In: *Langmuir* 28.4 (2011), 2113–2121. ISSN: 1520-5827. DOI: 10.1021/la203823t. URL: http://dx.doi.org/10.1021/la203823t.

[161] Yuqing Wu et al. "Raman spectroscopic investigation of the single-monolayer Langmuir–Blodgett film of C16NaphOH and C10AzoNaphC4N-SDS". In: *Spectrochimica Acta Part A: Molecular and Biomolecular Spectroscopy* 59.6 (2003), 1171–1176. ISSN: 1386-1425. DOI: 10.1016/s1386-1425(02)00297-4. URL: http://dx.doi.org/10.1016/s1386-1425(02)00297-4.

[162] Frédéric Raynal et al. "Quantitative analysis and thickness dependence study of Langmuir-Blodgett films of functionalized platinum nanoparticles by X-ray photoelectron spectroscopy". In: *Applied Surface Science* 236.1–4 (2004), 198–207. ISSN: 0169-4332. DOI: 10.1016/j.apsusc.2004.04.028. URL: http://dx.doi.org/10.1016/j.apsusc.2004.04.028.

[163] K. Kjaer et al. "An X-ray scattering study of lipid monolayers at the air-water interface and on solid supports". In: *Thin Solid Films* 159.1–2 (1988), 17–28. ISSN: 0040-6090. DOI: 10.1016/0040-6090(88)90613-x. URL: http://dx.doi.org/10.1016/0040-6090(88)90613-x.

[164] David L. Allara and Ping Zhang. *Characterization of Surfaces, Interfaces, and Thin Films of Organic Materials.* 2006. DOI: 10.1002/9783527603978.mst0030. URL: http://dx.doi.org/10.1002/9783527603978.mst0030.

[165] Cristina Stefaniu and Gerald Brezesinski. "X-ray investigation of monolayers formed at the soft air/water interface". In: *Current Opinion in Colloid amp; Interface Science* 19.3 (2014), 216–227. ISSN: 1359-0294. DOI: 10.1016/j.cocis.2014.01.004. URL: http://dx.doi.org/10.1016/j.cocis.2014.01.004.

[166] P. Dutta et al. "X-Ray Diffraction Studies of Organic Monolayers on the Surface of Water". In: *Physical Review Letters* 58.21 (1987), 2228–2231. ISSN: 0031-9007. DOI: 10.1103/physrevlett.58.2228. URL: http://dx.doi.org/10.1103/physrevlett.58.2228.

[167] R. M. Richardson and S. J. Roser. "Neutron reflection studies of spread monolayers of docosanoic acid and pentadecanoic acid on water". In: *Langmuir* 7.7 (1991), 1458–1467. ISSN: 1520-5827. DOI: 10.1021/la00055a029. URL: http://dx.doi.org/10.1021/la00055a029.

[168] Katarzyna Hac-Wydro et al. "Properties of -sitostanol/DPPC monolayers studied with Grazing Incidence X-ray Diffraction (GIXD) and Brewster Angle Microscopy". In: *Journal of Colloid and Interface Science* 364.1 (2011), 133–139. ISSN: 0021-9797. DOI: 10.1016/j.jcis.2011.08.030. URL: http://dx.doi.org/10.1016/j.jcis.2011.08.030.

[169] C.A. Helm et al. "Phases of phosphatidyl ethanolamine monolayers studied by synchrotron x-ray scattering". In: *Biophysical Journal* 60.6 (1991), 1457–1476. ISSN: 0006-3495. DOI: 10.1016/s0006-3495(91)82182-8. URL: http://dx.doi.org/10.1016/s0006-3495(91)82182-8.

[170] ARNOLD WEISSBERGER. "Physical Methods of Organic Chemistry". In: *Soil Science* 70.2 (1950), p. 167. ISSN: 0038-075X. DOI: 10.1097/00010694-195008000-00025. URL: http://dx.doi.org/10.1097/00010694-195008000-00025.

[171] A. W. Adamson and Julius Klerer. "Physical Chemistry of Surfaces". In: *Journal of The Electrochemical Society* 124.5 (1977), pp. 192C–192C. ISSN: 1945-7111. DOI: 10.1149/1.2133374. URL: http://dx.doi.org/10.1149/1.2133374.

[172] J. T. Davies, E. K. Rideal, and Max Bender. "Interfacial Phenomena". In: *Journal of The Electrochemical Society* 109.7 (1962), p. 175C. ISSN: 0013-4651. DOI: 10.1149/1.2425494. URL: http://dx.doi.org/10.1149/1.2425494.

[173] A.K. Panda et al. "Thermodynamic studies on mixed molecular langmuir films". In: *Colloids and Surfaces A: Physicochemical and Engineering Aspects* 247.1–3 (2004), 9–17. ISSN: 0927-7757. DOI: 10.1016/j.colsurfa.2004.07.008. URL: http://dx.doi.org/10.1016/j.colsurfa.2004.07.008.

[174] Jin Young Park and Rigoberto C. Advincula. "Nanostructuring polymers, colloids, and nanomaterials at the air–water interface through Langmuir and Langmuir–Blodgett techniques". In: *Soft Matter* 7.21 (2011), p. 9829. ISSN: 1744-6848. DOI: 10.1039/c1sm05750b. URL: http://dx.doi.org/10.1039/c1sm05750b.

[175] Inci Capan et al. "Sensing behaviors of lipophilic calix[4]arene phosphonate based Langmuir-Blodgett thin films for detection of volatile organic vapors". In: *Sensors and Actuators A: Physical* 347 (2022), p. 113947. ISSN: 0924-4247. DOI: 10.1016/j.sna.2022.113947. URL: http://dx.doi.org/10.1016/j.sna.2022.113947.

[176] Ilya Gorbachev et al. "Langmuir-Blodgett Films of Arachidic and Stearic Acids as Sensitive Coatings for Chloroform HF SAW Sensors". In: *Sensors* 23.1 (2022), p. 100. ISSN: 1424-8220. DOI: 10.3390/s23010100. URL: http://dx.doi.org/10.3390/s23010100.

[177] Lucía Herrer, Santiago Martín, and Pilar Cea. "Nanofabrication Techniques in Large-Area Molecular Electronic Devices". In: *Applied Sciences* 10.17 (2020), p. 6064. ISSN: 2076-3417. DOI: 10.3390/app10176064. URL: http://dx.doi.org/10.3390/app10176064.

[178] Qin Liu et al. "Langmuir–Blodgett Nanowire Devices for In Situ Probing of Zinc-Ion Batteries". In: *Small* 15.30 (2019). ISSN: 1613-6829. DOI: 10.1002/smll.201902141. URL: http://dx.doi.org/10.1002/smll.201902141.

[179] Camila Gouveia Barbosa et al. "Copolymers and enzymes blended as LB films changing the bioelectronics properties of interfaces". In: *Colloid and Interface Science Communications* 27 (2018), 40–44. ISSN: 2215-0382. DOI: 10.1016/j.colcom.2018.10.001. URL: http://dx.doi.org/10.1016/j.colcom.2018.10.001.

[180] N.E. Agbor, M.C. Petty, and A.P. Monkman. "Polyaniline thin films for gas sensing". In: *Sensors and Actuators B: Chemical* 28.3 (1995), 173–179. ISSN: 0925-4005. DOI: 10.1016/0925-4005(95)01725-9. URL: http://dx.doi.org/10.1016/0925-4005(95)01725-9.

[181] Sukhananazerin Abdulla and Biji Pullithadathil. "Unidirectional Langmuir–Blodgett-Mediated Alignment of Polyaniline-Functionalized Multiwalled Carbon Nanotubes for NH3 Gas Sensor Applications". In: *Langmuir* 36.39 (2020), 11618–11628. ISSN: 1520-5827. DOI: 10.1021/acs.langmuir.0c02200. URL: http://dx.doi.org/10.1021/acs.langmuir.0c02200.

[182] Miguel A. Andrés et al. "Methanol and Humidity Capacitive Sensors Based on Thin Films of MOF Nanoparticles". In: *ACS Applied Materials amp; Interfaces* 12.3 (2020), 4155–4162. ISSN: 1944-8252. DOI: 10.1021/acsami.9b20763. URL: http://dx.doi.org/10.1021/acsami.9b20763.

[183] Ying He et al. "Facile Preparation of Self-Assembled Layered Double Hydroxide-Based Composite Dye Films As New Chemical Gas Sensors". In: *ACS Sustainable Chemistry amp; Engineering* 7.12 (2019), 10888–10899. ISSN: 2168-0485. DOI: 10.1021/acssuschemeng.9b01780. URL: http://dx.doi.org/10.1021/acssuschemeng.9b01780.

[184] Ying He et al. "Facile Synthesis of Self-Assembled NiFe Layered Double Hydroxide-Based Azobenzene Composite Films with Photoisomerization and Chemical Gas Sensor Performances". In: *ACS Omega* 5.7 (2020), 3689–3698. ISSN: 2470-1343. DOI: 10.1021/acsomega.9b04290. URL: http://dx.doi.org/10.1021/acsomega.9b04290.

[185] Piotr Nowak et al. "SnO2/TiO2 Thin Film n-n Heterostructures of Improved Sensitivity to NO2". In: *Sensors* 20.23 (2020), p. 6830. ISSN: 1424-8220. DOI: 10.3390/s20236830. URL: http://dx.doi.org/10.3390/s20236830.

[186] Lennart Graewe et al. "Supramolecular Control of Photonic Response and Sensing of Nitricoxide using Iron(III) Corrole Monolayers and Their Stacks". In: *ChemPlusChem* 88.3 (2023). ISSN: 2192-6506. DOI: 10.1002/cplu.202200260. URL: http://dx.doi.org/10.1002/cplu.202200260.

[187] Yue-Yang Ma and Dong-Jin Qian. “Visual Luminescent Probes Constructed by Eu3+ Complex-Functionalized Silica Nanocomposites and Their Langmuir–Blodgett Films at Interfaces”. In: *Langmuir* 36.46 (2020), 14092–14103. ISSN: 1520-5827. DOI: 10.1021/acs.langmuir.0c02728. URL: http://dx.doi.org/10.1021/acs.langmuir.0c02728.

[188] C. Medina-Plaza et al. “Synergistic electrocatalytic effect of nanostructured mixed films formed by functionalised gold nanoparticles and bisphthalocyanines”. In: *Analytica Chimica Acta* 851 (2014), 95–102. ISSN: 0003-2670. DOI: 10.1016/j.aca.2014.08.049. URL: http://dx.doi.org/10.1016/j.aca.2014.08.049.

[189] Yanju Wu et al. “Langmuir-Blodgett Assembly of Carboxylic Multi-walled Carbon Nanotubes-Nafion for Amperometric Sensing of Codeine”. In: *Journal of The Electrochemical Society* 166.13 (2019), H592–H597. ISSN: 1945-7111. DOI: 10.1149/2.0371913jes. URL: http://dx.doi.org/10.1149/2.0371913jes.

[190] Ilya Gorbachev et al. “Langmuir–Blodgett Films with Immobilized Glucose Oxidase Enzyme Molecules for Acoustic Glucose Sensor Application”. In: *Sensors* 23.11 (2023), p. 5290. ISSN: 1424-8220. DOI: 10.3390/s23115290. URL: http://dx.doi.org/10.3390/s23115290.

[191] Ke-Hsuan Wang et al. “Architecture effects of glucose oxidase/Au nanoparticle composite Langmuir-Blodgett films on glucose sensing performance”. In: *Applied Surface Science* 366 (2016), 202–209. ISSN: 0169-4332. DOI: 10.1016/j.apsusc.2016.01.047. URL: http://dx.doi.org/10.1016/j.apsusc.2016.01.047.

[192] F. J. Pavinatto et al. “Optimized architecture for Tyrosinase-containing Langmuir–Blodgett films to detect pyrogallol”. In: *Journal of Materials Chemistry* 21.13 (2011), p. 4995. ISSN: 1364-5501. DOI: 10.1039/c0jm03864d. URL: http://dx.doi.org/10.1039/c0jm03864d.

[193] Carlos da Rocha Junior and Luciano Caseli. “Adsorption and enzyme activity of asparaginase at lipid Langmuir and Langmuir-Blodgett films”. In: *Materials Science and Engineering: C* 73 (2017), 579–584. ISSN: 0928-4931. DOI: 10.1016/j.msec.2016.12.041. URL: http://dx.doi.org/10.1016/j.msec.2016.12.041.

[194] Fabio A. Scholl et al. "Carbon Nanotubes Arranged As Smart Interfaces in Lipid Langmuir–Blodgett Films Enhancing the Enzymatic Properties of Penicillinase for Biosensing Applications". In: *ACS Applied Materials amp; Interfaces* 9.36 (2017), 31054–31066. ISSN: 1944-8252. DOI: 10.1021/acsami.7b08095. URL: http://dx.doi.org/10.1021/acsami.7b08095.

[195] Caio Vinícius Teles Rossini, Celso Molina, and Luciano Caseli. "Immobilization of urease in Langmuir-Blodgett films of di-ureasil hybrid compounds". In: *Thin Solid Films* 670 (2019), 17–23. ISSN: 0040-6090. DOI: 10.1016/j.tsf.2018.12.006. URL: http://dx.doi.org/10.1016/j.tsf.2018.12.006.

[196] Priscila Alessio et al. "Detection of catechol using mixed Langmuir–Blodgett films of a phospholipid and phthalocyanines as voltammetric sensors". In: *The Analyst* 135.10 (2010), p. 2591. ISSN: 1364-5528. DOI: 10.1039/c0an00159g. URL: http://dx.doi.org/10.1039/c0an00159g.

[197] LUCIANO CASELI. "Enzymes immobilized in Langmuir-Blodgett films: Why determining the surface properties in Langmuir monolayer is important?" In: *Anais da Academia Brasileira de Ciências* 90.1 suppl 1 (2018), 631–644. ISSN: 0001-3765. DOI: 10.1590/0001-3765201720170453. URL: http://dx.doi.org/10.1590/0001-3765201720170453.

[198] Shalini devi Kalyana Sundaram et al. "Enzyme Cascade Electrode Reactions with Nanomaterials and Their Applicability towards Biosensor and Biofuel Cells". In: *Biosensors* 13.12 (2023), p. 1018. ISSN: 2079-6374. DOI: 10.3390/bios13121018. URL: http://dx.doi.org/10.3390/bios13121018.

[199] Celina Massumi Miyazaki et al. "Improved antibody loading on self-assembled graphene oxide films for using in surface plasmon resonance immunosensors". In: *Applied Surface Science* 490 (2019), 502–509. ISSN: 0169-4332. DOI: 10.1016/j.apsusc.2019.06.095. URL: http://dx.doi.org/10.1016/j.apsusc.2019.06.095.

[200] Shipra Solanki et al. "Langmuir–Blodgett Nanoassemblies of the MoS2–Au Composite at the Air–Water Interface for Dengue Detection". In: *ACS Applied Materials amp; Interfaces* 10.3 (2018), 3020–3028. ISSN: 1944-8252. DOI: 10.1021/acsami.7b14391. URL: http://dx.doi.org/10.1021/acsami.7b14391.

[201] Richard L. McCreery and Adam Johan Bergren. “Progress with Molecular Electronic Junctions: Meeting Experimental Challenges in Design and Fabrication”. In: *Advanced Materials* 21.43 (2009), 4303–4322. ISSN: 1521-4095. DOI: 10.1002/adma.200802850. URL: http://dx.doi.org/10.1002/adma.200802850.

[202] Jian Mao et al. “Langmuir–Blodgett fabrication of large-area black phosphorus-C60 thin films and heterojunction photodetectors”. In: *Nanoscale* 12.38 (2020), 19814–19823. ISSN: 2040-3372. DOI: 10.1039/d0nr04537c. URL: http://dx.doi.org/10.1039/d0nr04537c.

[203] Hyeri Kim et al. “Spontaneous hybrids of graphene and carbon nanotube arrays at the liquid–gas interface for Li-ion battery anodes”. In: *Chemical Communications* 54.41 (2018), 5229–5232. ISSN: 1364-548X. DOI: 10.1039/c8cc02148a. URL: http://dx.doi.org/10.1039/c8cc02148a.

[204] Sriramprabha Ramasamy, Ponpandian Nagamony, and Viswanathan Chinnuswamy. “Self-assembled SnO2/reduced graphene oxide nanocomposites via Langmuir-Blodgett technique as anode materials for Li-ion batteries”. In: *Materials Letters* 218 (2018), 295–298. ISSN: 0167-577X. DOI: 10.1016/j.matlet.2018.01.177. URL: http://dx.doi.org/10.1016/j.matlet.2018.01.177.

[205] Wonsik Eom et al. “Graphene-Mimicking 2D Porous Co3O4 Nanofoils for Lithium Battery Applications”. In: *Advanced Functional Materials* 26.42 (2016), 7605–7613. ISSN: 1616-3028. DOI: 10.1002/adfm.201602320. URL: http://dx.doi.org/10.1002/adfm.201602320.

[206] Chen Fang et al. “Recent advances in polysulfide mediation of lithium-sulfur batteries via facile cathode and electrolyte modification”. In: *APL Materials* 7.8 (2019). ISSN: 2166-532X. DOI: 10.1063/1.5110525. URL: http://dx.doi.org/10.1063/1.5110525.

[207] Mun Sek Kim et al. “Multifunctional Separator Coatings for High-Performance Lithium–Sulfur Batteries”. In: *Advanced Materials Interfaces* 3.22 (2016). ISSN: 2196-7350. DOI: 10.1002/admi.201600450. URL: http://dx.doi.org/10.1002/admi.201600450.

[208] Xin-Bing Cheng et al. “A Review of Solid Electrolyte Interphases on Lithium Metal Anode”. In: *Advanced Science* 3.3 (2015). ISSN: 2198-3844. DOI: 10.1002/advs.201500213. URL: http://dx.doi.org/10.1002/advs.201500213.

[209] Mun Sek Kim et al. “Langmuir–Blodgett artificial solid-electrolyte interphases for practical lithium metal batteries”. In: *Nature Energy* 3.10 (2018), 889–898. ISSN: 2058-7546. DOI: 10.1038/s41560-018-0237-6. URL: http://dx.doi.org/10.1038/s41560-018-0237-6.

[210] Junhyeok Kim et al. “Controllable Solid Electrolyte Interphase in Nickel-Rich Cathodes by an Electrochemical Rearrangement for Stable Lithium-Ion Batteries”. In: *Advanced Materials* 30.5 (2017). ISSN: 1521-4095. DOI: 10.1002/adma.201704309. URL: http://dx.doi.org/10.1002/adma.201704309.

[211] Cristina Stefaniu, Gerald Brezesinski, and Helmuth Möhwald. “Langmuir monolayers as models to study processes at membrane surfaces”. In: *Advances in Colloid and Interface Science* 208 (2014), 197–213. ISSN: 0001-8686. DOI: 10.1016/j.cis.2014.02.013. URL: http://dx.doi.org/10.1016/j.cis.2014.02.013.

[212] Martyna Krajewska, Katarzyna Dopierała, and Krystyna Prochaska. “Lipid–Protein Interactions in Langmuir Monolayers under Dynamically Varied Conditions”. In: *The Journal of Physical Chemistry B* 124.1 (2019), 302–311. ISSN: 1520-5207. DOI: 10.1021/acs.jpcb.9b10351. URL: http://dx.doi.org/10.1021/acs.jpcb.9b10351.

[213] André C. Machado and Luciano Caseli. “Interaction of nitrofurantoin with lipid langmuir monolayers as cellular membrane models distinguished with tensiometry and infrared spectroscopy”. In: *Colloids and Surfaces B: Biointerfaces* 188 (2020), p. 110794. ISSN: 0927-7765. DOI: 10.1016/j.colsurfb.2020.110794. URL: http://dx.doi.org/10.1016/j.colsurfb.2020.110794.

[214] Karolina Weder et al. “Studies on the interactions of anticancer drug - Minerval - with membrane lipids in binary and ternary Langmuir monolayers”. In: *Biochimica et Biophysica Acta (BBA) - Biomembranes* 1860.11 (2018), 2329–2336. ISSN: 0005-2736. DOI: 10.1016/j.bbamem.2018.05.019. URL: http://dx.doi.org/10.1016/j.bbamem.2018.05.019.

[215] Gerald Brezesinski and Helmuth Möhwald. “Langmuir monolayers to study interactions at model membrane surfaces”. In: *Advances in Colloid and Interface Science* 100–102 (2003), 563–584. ISSN: 0001-8686. DOI: 10.1016/s0001-8686(02)00071-4. URL: http://dx.doi.org/10.1016/s0001-8686(02)00071-4.

[216] João Victor N. Ferreira et al. "Mechanism of Action of Thymol on Cell Membranes Investigated through Lipid Langmuir Monolayers at the Air–Water Interface and Molecular Simulation". In: *Langmuir* 32.13 (2016), 3234–3241. ISSN: 1520-5827. DOI: 10.1021/acs.langmuir.6b00600. URL: http://dx.doi.org/10.1021/acs.langmuir.6b00600.

[217] Shan Mohanan et al. "A Dual Protective Drug Delivery System Based on Lipid Coated Core-Shell Mesoporous Silica for Efficient Delivery of Cabazitaxel to Prostate Cancer Cells". In: *Bulletin of the Chemical Society of Japan* 96.10 (2023), 1188–1195. ISSN: 1348-0634. DOI: 10.1246/bcsj.20230167. URL: http://dx.doi.org/10.1246/bcsj.20230167.

[218] Shayna Sharma et al. "Extracellular Vesicle Nanoarchitectonics for Novel Drug Delivery Applications". In: *Small* 17.42 (2021). ISSN: 1613-6829. DOI: 10.1002/smll.202102220. URL: http://dx.doi.org/10.1002/smll.202102220.

[219] Jingwen Song et al. "Bio-gel nanoarchitectonics in tissue engineering". In: *Nanoscale* 16.28 (2024), 13230–13246. ISSN: 2040-3372. DOI: 10.1039/d4nr00609g. URL: http://dx.doi.org/10.1039/d4nr00609g.

[220] Jingwen Song et al. "Large-Area Aligned Fullerene Nanocrystal Scaffolds as Culture Substrates for Enhancing Mesenchymal Stem Cell Self-Renewal and Multipotency". In: *ACS Applied Nano Materials* 3.7 (2020), 6497–6506. ISSN: 2574-0970. DOI: 10.1021/acsanm.0c00973. URL: http://dx.doi.org/10.1021/acsanm.0c00973.

[221] Venkata Krishnan et al. "Vortex-Aligned Fullerene Nanowhiskers as a Scaffold for Orienting Cell Growth". In: *ACS Applied Materials amp; Interfaces* 7.28 (2015), 15667–15673. ISSN: 1944-8252. DOI: 10.1021/acsami.5b04811. URL: http://dx.doi.org/10.1021/acsami.5b04811.

[222] Osvaldo N. Oliveira, Luciano Caseli, and Katsuhiko Ariga. "The Past and the Future of Langmuir and Langmuir–Blodgett Films". In: *Chemical Reviews* 122.6 (2022), 6459–6513. ISSN: 1520-6890. DOI: 10.1021/acs.chemrev.1c00754. URL: http://dx.doi.org/10.1021/acs.chemrev.1c00754.

[223] Luzhu Xu et al. "Continuous Langmuir–Blodgett Deposition and Transfer by Controlled Edge-to-Edge Assembly of Floating 2D Materials". In: *Langmuir* 35.1 (2018), 51–59. ISSN: 1520-5827. DOI: 10.1021/acs.langmuir.8b03173. URL: http://dx.doi.org/10.1021/acs.langmuir.8b03173.

[224] B.P. Binks. "Insoluble monolayers of weakly ionising low molar mass materials and their deposition to form Langmuir-Blodgett multilayers". In: *Advances in Colloid and Interface Science* 34 (1991), 343–432. ISSN: 0001-8686. DOI: 10.1016/0001-8686(91)80053-m. URL: http://dx.doi.org/10.1016/0001-8686(91)80053-m.

[225] LUCY NETZER, RADU ISCOVICI, and JACOB SAGIV. "ADSORBED MONOLAYERS VERSUS LANGMUIR–BLODGETT MONOLAYERS—WHY AND HOW? I: FROM MONOLAYER TO MULTILAYER, BY ADSORPTION". In: *Langmuir–Blodgett Films, 1982*. Elsevier, 1983, 235–241. ISBN: 9780444421739. DOI: 10.1016/b978-0-444-42173-9.50040-7. URL: http://dx.doi.org/10.1016/b978-0-444-42173-9.50040-7.

[226] Luiz Henrique Rodrigues Rola Possarle, José Roberto Siqueira Junior, and Luciano Caseli. "Insertion of carbon nanotubes in Langmuir-Blodgett films of stearic acid and asparaginase enhancing the catalytic performance". In: *Colloids and Surfaces B: Biointerfaces* 192 (2020), p. 111032. ISSN: 0927-7765. DOI: 10.1016/j.colsurfb.2020.111032. URL: http://dx.doi.org/10.1016/j.colsurfb.2020.111032.

[227] Tong Zhang et al. "Fewer-layer conductive metal-organic Langmuir-Blodgett films as electrocatalysts enable an ultralow detection limit of H2O2". In: *Applied Surface Science* 539 (2021), p. 148255. ISSN: 0169-4332. DOI: 10.1016/j.apsusc.2020.148255. URL: http://dx.doi.org/10.1016/j.apsusc.2020.148255.

[228] Max J. Hülsey et al. "In situ spectroscopy-guided engineering of rhodium single-atom catalysts for CO oxidation". In: *Nature Communications* 10.1 (2019). ISSN: 2041-1723. DOI: 10.1038/s41467-019-09188-9. URL: http://dx.doi.org/10.1038/s41467-019-09188-9.

[229] Guilherme Nuñez Jaroque, Patrícia Sartorelli, and Luciano Caseli. "Interfacial vibrational spectroscopy and Brewster angle microscopy distinguishing the interaction of terpineol in cell membrane models at the air-water interface". In: *Biophysical Chemistry* 246 (2019), 1–7.

ISSN: 0301-4622. DOI: 10.1016/j.bpc.2018.12.003. URL: http://dx.doi.org/10.1016/j.bpc.2018.12.003.

[230] Adrien P. Cote et al. "Porous, Crystalline, Covalent Organic Frameworks". In: *Science* 310.5751 (2005), 1166–1170. ISSN: 1095-9203. DOI: 10.1126/science.1120411. URL: http://dx.doi.org/10.1126/science.1120411.

[231] Watcharop Chaikittisilp, Yusuke Yamauchi, and Katsuhiko Ariga. "Material Evolution with Nanotechnology, Nanoarchitectonics, and Materials Informatics: What will be the Next Paradigm Shift in Nanoporous Materials?" In: *Advanced Materials* 34.7 (2022). ISSN: 1521-4095. DOI: 10.1002/adma.202107212. URL: http://dx.doi.org/10.1002/adma.202107212.

[232] Zhiyong Wang et al. "Interfacial Synthesis of Layer-Oriented 2D Conjugated Metal–Organic Framework Films toward Directional Charge Transport". In: *Journal of the American Chemical Society* 143.34 (2021), 13624–13632. ISSN: 1520-5126. DOI: 10.1021/jacs.1c05051. URL: http://dx.doi.org/10.1021/jacs.1c05051.

[233] Tomohiro Murata et al. "Nanometer-Flat DNA-Featured Thin Films Prepared via Laser Molecular Beam Deposition under High-Vacuum for Selective Methanol Sensing". In: *Bulletin of the Chemical Society of Japan* 96.1 (2022), 29–34. ISSN: 1348-0634. DOI: 10.1246/bcsj.20220303. URL: http://dx.doi.org/10.1246/bcsj.20220303.

[234] Pengfei Bian et al. "Interfacial aggregation behavior of novel carbazole-based composite Langmuir-Blodgett films for photoelectric conversion and catalytic performance". In: *Colloids and Surfaces A: Physicochemical and Engineering Aspects* 656 (2023), p. 130460. ISSN: 0927-7757. DOI: 10.1016/j.colsurfa.2022.130460. URL: http://dx.doi.org/10.1016/j.colsurfa.2022.130460.

[235] Dibyendu Das, Sumyra Sidiq, and Santanu Kumar Pal. "Design of bio-molecular interfaces using liquid crystals demonstrating endotoxin interactions with bacterial cell wall components". In: *RSC Advances* 5.81 (2015), 66476–66486. ISSN: 2046-2069. DOI: 10.1039/c5ra09640e. URL: http://dx.doi.org/10.1039/c5ra09640e.

[236] Rikako Hama et al. "Recent Developments in Biopolymer-Based Hydrogels for Tissue Engineering Applications". In: *Biomolecules* 13.2 (2023), p. 280. ISSN: 2218-273X. DOI: 10.3390/biom13020280. URL: http://dx.doi.org/10.3390/biom13020280.

[237] Katsuhiko Ariga et al. "Life science nanoarchitectonics at interfaces". In: *Materials Chemistry Frontiers* 5.3 (2021), 1018–1032. ISSN: 2052-1537. DOI: 10.1039/d0qm00615g. URL: http://dx.doi.org/10.1039/d0qm00615g.

[238] T. Govindaraju and M. B. Avinash. "Two-dimensional nanoarchitectonics: organic and hybrid materials". In: *Nanoscale* 4.20 (2012), p. 6102. ISSN: 2040-3372. DOI: 10.1039/c2nr31167d. URL: http://dx.doi.org/10.1039/c2nr31167d.

[239] Luz M. Ballesteros et al. "Directionally Oriented LB Films of an OPE Derivative: Assembly, Characterization, and Electrical Properties". In: *Langmuir* 27.7 (2011), 3600–3610. ISSN: 1520-5827. DOI: 10.1021/la104734j. URL: http://dx.doi.org/10.1021/la104734j.

[240] Cansu Ozkaya Erdogan et al. "Sensor application of pyridine modified calix[4]arene Langmuir-Blodgett thin film". In: *Optik* 265 (2022), p. 169492. ISSN: 0030-4026. DOI: 10.1016/j.ijleo.2022.169492. URL: http://dx.doi.org/10.1016/j.ijleo.2022.169492.

[241] Yaser Acikbas et al. "Optical and Vapor Sensing Properties of Calix[4]arene Langmuir-Blodgett Thin Films with Host–Guest Principles". In: *Journal of Macromolecular Science, Part A* 55.7 (2018), 526–532. ISSN: 1520-5738. DOI: 10.1080/10601325.2018.1476824. URL: http://dx.doi.org/10.1080/10601325.2018.1476824.

[242] Juan M. Giussi et al. "Practical use of polymer brushes in sustainable energy applications: interfacial nanoarchitectonics for high-efficiency devices". In: *Chemical Society Reviews* 48.3 (2019), 814–849. ISSN: 1460-4744. DOI: 10.1039/c8cs00705e. URL: http://dx.doi.org/10.1039/c8cs00705e.

[243] Masaru Muramatsu et al. "Fabrication of Densely Packed Titania Nanosheet Films on Solid Surface by Use of LangmuirBlodgett Deposition Method without Amphiphilic Additives". In: *Langmuir* 21.14 (2005), 6590–6595. ISSN: 1520-5827. DOI: 10.1021/la050293f. URL: http://dx.doi.org/10.1021/la050293f.

[244] Samuel H. Gyepi-Garbrah and Roberta Šilerová. "The first direct comparison of self-assembly and Langmuir–Blodgett deposition techniques: Two routes to highly organized monolayers". In: *Physical Chemistry Chemical Physics* 4.14 (2002), 3436–3442. ISSN: 1463-9084. DOI: 10.1039/b201698b. URL: http://dx.doi.org/10.1039/b201698b.

[245] Syed Arshad Hussain et al. "Unique supramolecular assembly through Langmuir – Blodgett (LB) technique". In: *Heliyon* 4.12 (2018), e01038. ISSN: 2405-8440. DOI: 10.1016/j.heliyon.2018.e01038. URL: http://dx.doi.org/10.1016/j.heliyon.2018.e01038.

S7 Second Paper generated using Strategy 1 (21 input papers)

Langmuir and Langmuir-Blodgett Films: Fundamentals, Frontiers, and Applications – A Comprehensive Survey

April 23, 2025

Contents

1 Introduction 2

2 Fundamentals of Langmuir and Langmuir-Blodgett Films 6
2.1 Amphiphilic Molecules and Interfacial Self-Assembly 6
2.2 Langmuir Monolayer Formation and Characterization 8
2.3 Monolayer Transfer: The Langmuir-Blodgett and Langmuir-Schaefer Techniques 11

3 Materials for Langmuir and Langmuir-Blodgett Films 15
3.1 Traditional Amphiphiles 16
3.2 Novel Materials 19
3.3 Hybrid and Composite Materials 21

4 Characterization Techniques for Langmuir and Langmuir-Blodgett Films 23
4.1 Surface Pressure-Area Isotherms 25
4.2 Surface Potential Measurements 29
4.3 Microscopic Techniques 29
4.4 Spectroscopic Techniques 31
4.5 Diffraction Techniques 33

5 **Applications of Langmuir and Langmuir-Blodgett Films** **34**
5.1 Molecular Electronics 36
5.2 Sensors 37
5.3 Biomedical Applications 38
5.4 Optical and Photonic Devices 39

6 **Challenges and Future Directions** **40**
6.1 Addressing the Limitations of LB Films 40
6.2 Emerging Trends and Opportunities 42
6.3 Future Research Directions 45

7 **Conclusion** **47**

Abstract

Langmuir (L) and Langmuir-Blodgett (LB) technology provides a powerful methodology for fabricating ultrathin molecular films with exceptional control over thickness, structure, and molecular orientation, often down to the single-molecule level. This survey presents a comprehensive overview of the field, beginning with the fundamental principles governing the formation and phase behavior of Langmuir monolayers—organized molecular assemblies formed at the air-water interface—and their subsequent transfer onto solid substrates to create Langmuir-Blodgett films via vertical or horizontal deposition techniques. We explore the remarkable versatility of the technique, encompassing a diverse range of materials from classical amphiphiles and polymers to contemporary nanomaterials, including nanoparticles, carbon allotropes, and biomacromolecules. Essential characterization methods for probing film properties both at the interface and on substrates are reviewed. The survey highlights the broad spectrum of applications driven by the unique architectural control offered by LB films, including advanced sensors, molecular electronics components, biomimetic systems, and energy storage/conversion devices. Furthermore, we discuss the inherent challenges, such as film stability and process scalability, in comparison with alternative thin-film techniques. Finally, this review examines the future perspectives and ongoing innovations in the field, emphasizing its continued relevance in nanoarchitectonics and its potential for enabling future technological breakthroughs through precise molecular assembly at interfaces.

1 Introduction

Langmuir-Blodgett (LB) film technology represents a powerful method for the fabrication of highly ordered, ultra-thin molecular films with precision control at the molecular level. This technique involves the meticulous manipulation and transfer of monomolecular layers, initially formed at an air-water interface, onto solid substrates. The resulting films, known as Langmuir-Blodgett films, can range from single monolayers to complex multilayer structures, exhibiting remarkable uniformity and controlled architecture. The significance of LB technology stems from its unique ability to assemble diverse molecular species, including lipids, polymers, nanoparticles, and biomolecules, into functional thin films with tailored properties. Controlling molecular orientation at the interface, such as achieving the edge-on or face-on arrangements depicted for macrocycles in Figure 1, is a key feature allowing for the fine-tuning of film characteristics like electronic or optical response. This capability has positioned LB films as crucial components in various scientific and technological domains, including materials science, nanotechnology, molecular electronics, sensor development, and biomimetic systems. The precise control over film thickness, molecular orientation, and composition offered by the LB method allows for the engineering of surfaces and interfaces with specific functionalities, driving innovation across these fields.

The fundamental principle underlying the formation of Langmuir and LB films relies on the self-assembly behavior of amphiphilic molecules at an interface [1]. Amphiphilic molecules possess distinct hydrophilic (water-attracting) and hydrophobic (water-repelling) parts. When dissolved in a volatile, water-immiscible organic solvent and spread onto an aqueous subphase surface, these molecules spontaneously arrange themselves at the air-water interface upon solvent evaporation, with hydrophilic heads anchored to the water and hydrophobic tails oriented towards the air. Initially forming a disordered, two-dimensional 'gaseous' phase, these molecules are compressed using a movable barrier within a Langmuir trough. This compression reduces the available surface area, increases the surface pressure (the reduction in surface tension caused by the monolayer), and forces the molecules through more ordered liquid and solid-like phases. The relationship between surface pressure and the area occupied per molecule is captured in a surface pressure-area (π-A) isotherm, such as the example shown in Figure 2, which provides critical information about the phase behavior, compressibility, and packing density of the monolayer, known as a Langmuir film, at the interface. Com-

plementary techniques like Brewster Angle Microscopy (BAM), exemplified by the images in Figure 3, allow direct visualization of the film's morphology, homogeneity, and domain formation during this compression process. Once a stable and ordered Langmuir film is formed at a desired surface pressure, it can be transferred onto a solid substrate [2]. The classical LB deposition involves vertically dipping and withdrawing the substrate through the monolayer, allowing layer-by-layer adhesion [3, 4, 5]. Repeating this process builds multilayer films with controlled thickness and structure (e.g., X-, Y-, or Z-type deposition). An alternative horizontal transfer method, known as the Langmuir-Schaefer (LS) technique, involves touching the substrate horizontally to the monolayer.

The historical roots of LB technology extend back over a century. Early observations of oil spreading on water date back centuries, with Benjamin Franklin conducting some of the first scientific experiments in the 18th century. However, the scientific foundation was laid by Agnes Pockels in the late 19th century, who developed rudimentary techniques to study surface films. Her work, recognized by Lord Rayleigh, was published in 1891. Irving Langmuir significantly advanced the field in the early 20th century, developing the Langmuir trough and systematically studying amphiphilic molecules at the air-water interface, earning him the Nobel Prize in Chemistry in 1932. Katharine Blodgett, his assistant, further refined the technique in the 1930s, demonstrating the controlled deposition of successive monolayers onto solid substrates, creating the multilayer films that now bear both their names. The potential for creating complex molecular assemblies was later envisioned by researchers like Hans Kuhn in the 1960s, paving the way for concepts in molecular electronics. The establishment of regular international conferences, starting as the LB Conferences in 1983 and evolving into the International Conference on Organized Molecular Films (ICOMF), has fostered continuous development and collaboration in the field, reflecting its enduring importance [6]. Despite the emergence of other thin-film fabrication techniques like self-assembled monolayers (SAMs) and layer-by-layer (LbL) assembly [3, 7, 4], LB technology remains unique for its ability to create highly ordered films from pre-organized interfacial monolayers [8, 2].

The objective of this survey is to provide a comprehensive overview of the field of Langmuir and Langmuir-Blodgett films, encompassing fundamental principles, material diversity, characterization methodologies, and contemporary applications [2, 9, 8]. We aim to highlight the versatility and enduring relevance of this technique in modern science and technology. The scope

includes a discussion of the underlying physical chemistry of monolayer formation and transfer, an exploration of the wide range of materials amenable to LB deposition (from classical amphiphiles to complex nanomaterials) [10], a review of the essential techniques used to characterize these ultra-thin films both at the interface and on substrates [11, 12], and an examination of their application in areas such as sensors [13, 14, 15], electronics [16], biomimicry, and energy storage [17, 18]. Furthermore, we will address the inherent challenges associated with the LB technique and discuss future directions and potential advancements.

This survey paper is structured to guide the reader from foundational concepts to advanced applications and future perspectives. Section 2 delves into the Fundamentals of Langmuir and Langmuir-Blodgett Films, detailing the thermodynamics and phase behavior of monolayers at the air-water interface and the mechanics of film transfer [8, 19, 20]. Section 3 explores the diverse range of Materials used for Langmuir and Langmuir-Blodgett Films, covering traditional amphiphiles, polymers, nanoparticles, biomolecules, and hybrid systems [21, 22, 23]. Section 4 focuses on Characterization Techniques for Langmuir and Langmuir-Blodgett Films, describing methods used to probe film structure, morphology, and properties [12, 24]. Section 5 showcases the various Applications of Langmuir and Langmuir-Blodgett Films in different technological fields [2, 25, 18, 26]. Section 6 addresses the current Challenges and Future Directions, discussing limitations and potential innovations [9, 6]. Finally, Section 7 provides a concluding summary of the state of the art and the outlook for LB technology.

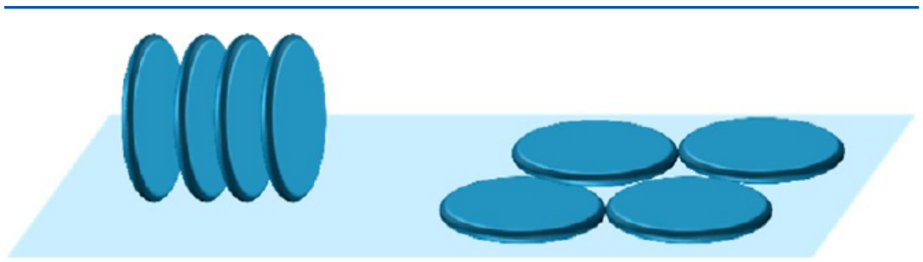

Figure 1: Schematic representation of the edge-on (left) and face-on (right) orientations of a macrocycle at the air—water interface. Adapted from Alinia, Z., et al., 2024 [27].

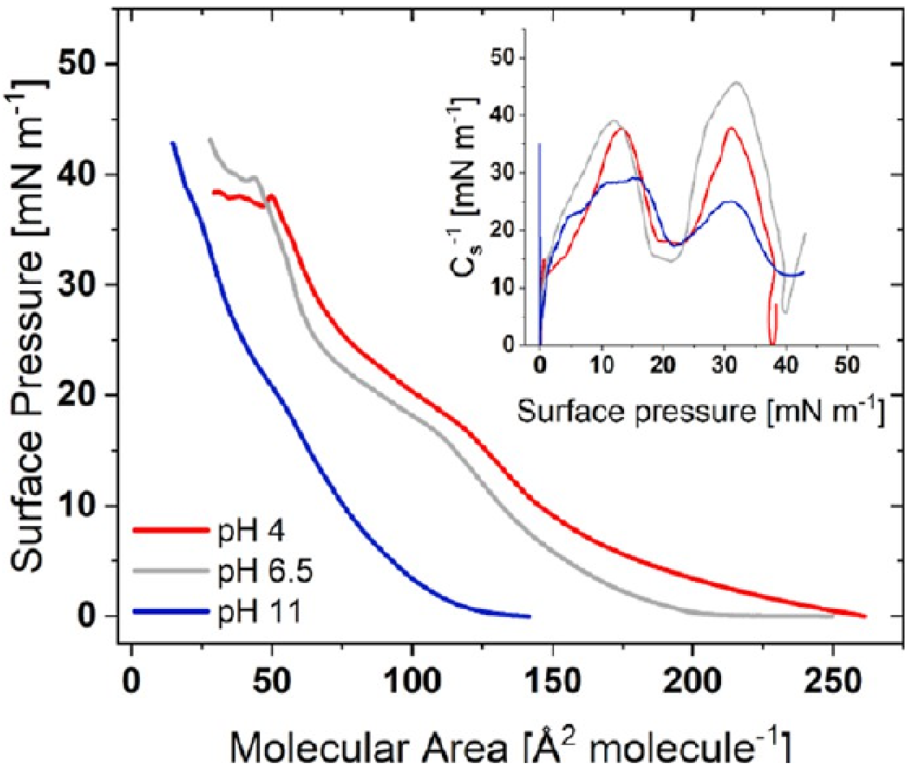

Figure 2: Example of a surface pressure—molecular area isotherm and compressibility modulus plot (inset) for a porphyrin derivative at different subphase pH values, illustrating the data obtained during monolayer compression. Adapted from Alinia, Z., et al., 2024 [27].

2 Fundamentals of Langmuir and Langmuir-Blodgett Films

The formation and manipulation of molecularly thin films at interfaces represent a cornerstone of surface science and nanotechnology. Among the earliest and most versatile techniques developed for this purpose are the Langmuir and Langmuir-Blodgett (LB) methods. These techniques rely on the unique properties of amphiphilic molecules at the air-water interface to create highly ordered, two-dimensional arrays (Langmuir films), which can subsequently be transferred onto solid supports as single or multiple layers (Langmuir-Blodgett films). Understanding the fundamental principles governing the

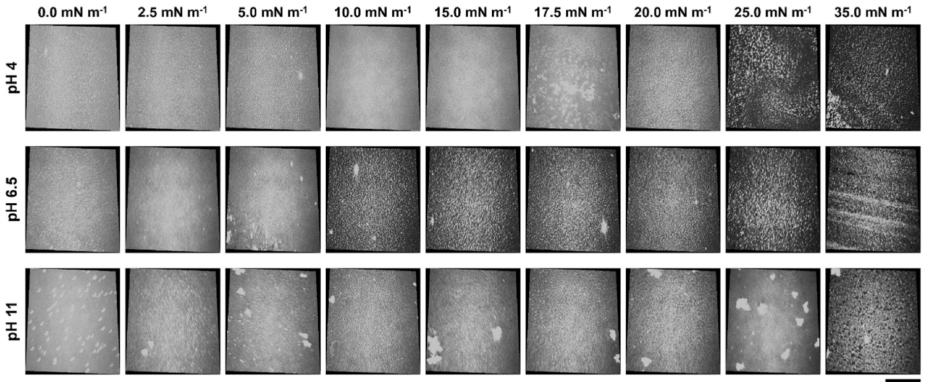

Figure 3: Representative Brewster Angle Microscopy (BAM) images showing the morphology of a porphyrin derivative monolayer at the air-water interface at different surface pressures and subphase pH values. Adapted from Alinia, Z., et al., 2024 [27].

behavior of amphiphiles at interfaces, the formation of Langmuir monolayers, and the transfer process is crucial for harnessing the full potential of LB technology in various applications, ranging from biomimetic membranes to advanced electronic and optical devices.

2.1 Amphiphilic Molecules and Interfacial Self-Assembly

The foundation of Langmuir and LB film technology lies in the specific properties of amphiphilic molecules. These molecules possess a dual chemical nature, characterized by a distinct hydrophilic (water-loving) part and a hydrophobic (water-fearing) part within the same structure. The hydrophilic moiety, often referred to as the "head group," typically consists of polar or charged functional groups (e.g., carboxyl -COOH, hydroxyl -OH, phosphate -PO_4^-, amine -NH_3^+) that exhibit strong affinity for water through electrostatic interactions, ion-dipole forces, dipole-dipole interactions, or hydrogen bonding. Conversely, the hydrophobic moiety, commonly called the "tail," is usually a long, nonpolar hydrocarbon chain (e.g., alkyl or acyl chains) or a fluorocarbon chain, which is repelled by the aqueous environment. Classic examples of amphiphiles used in early studies and still widely employed include fatty acids (like stearic acid, $CH_3(CH_2)_{16}COOH$) and phospholipids (major components of biological membranes, such as dipalmitoylphosphatidylcholine, DPPC).

A critical requirement for forming stable Langmuir monolayers is that the amphiphilic substance must be essentially insoluble in the aqueous subphase. The hydrophobic tail provides this necessary insolubility, preventing the molecules from dissolving into the bulk water. Simultaneously, the hydrophilic head group must possess sufficient affinity for water to anchor the molecule firmly at the air-water interface [1]. When such an amphiphile is introduced onto the water surface, typically dissolved in a volatile organic solvent that spreads rapidly and then evaporates, the molecules spontaneously arrange themselves to minimize the overall free energy of the system. The hydrophilic heads immerse themselves in the water, maximizing favorable interactions, while the hydrophobic tails orient themselves away from the water, extending into the air phase to minimize unfavorable contact with the polar water molecules.

This spontaneous arrangement is a form of self-assembly driven by a complex interplay of forces [5, 28]. The primary driving force is the hydrophobic effect, which arises not from direct repulsion between water and nonpolar tails, but rather from the entropic gain associated with minimizing the disruption of the hydrogen-bonded network of water molecules surrounding the hydrophobic tails [29, 30]. By segregating the tails away from the water, the system maximizes the entropy of the water. Additionally, van der Waals attractive forces between the hydrophobic tails become significant when the molecules are brought into close proximity, promoting ordering and packing within the monolayer. The anchoring of the head groups involves specific interactions with water molecules, whose strength is characteristic of physisorption processes. The balance between the adhesion forces (head group-water, tail-air) and cohesion forces (tail-tail, head-head) dictates the structure and stability of the resulting monolayer. For spontaneous spreading to occur across the entire available interface, adhesion forces must dominate over cohesion forces, allowing the molecules to behave like a two-dimensional gas initially.

While simple linear amphiphiles like fatty acids tend to adopt relatively predictable orientations with tails directed away from the water, more complex amphiphilic structures can exhibit diverse arrangements at the air-water interface [31, 32]. The specific molecular architecture, including the presence of multiple hydrophilic or hydrophobic groups, rigid cores, bulky side chains, or specific functional moieties, dictates the possible conformations and packing modes [33, 34, 35]. Figures 6 and 7 illustrate this complexity. Figure 6 shows potential "face-on" conformers for large, multi-acid tetraazaporphyrin

(TAP) derivatives at the interface, highlighting how the molecule might orient its planar core relative to the water surface, influenced by the positioning of its multiple hydrophilic acid groups and hydrophobic alkyl chains [36]. Figure 7 further explores possible interfacial orientations for one such complex amphiphile (4b), depicting scenarios where the molecule might lie flat, tilt, or adopt other arrangements depending on the balance of interactions between its different parts and the interface. These examples underscore how the principles of hydrophilic/hydrophobic segregation and self-assembly manifest in varied ways for structurally complex amphiphiles, influencing the resulting monolayer's properties [37, 38].

2.2 Langmuir Monolayer Formation and Characterization

Langmuir monolayers are prepared and studied using a specialized apparatus known as a Langmuir trough. A typical trough consists of a shallow rectangular compartment, usually made of an inert material like polytetrafluoroethylene (PTFE) to prevent contamination and ensure high surface tension of the water subphase. The trough is filled with ultrapure water (or an aqueous solution containing specific ions, buffers, or other additives) until a positive meniscus is formed above the edges. One or two movable barriers, also made of an inert material (e.g., PTFE or hydrophilic polyacetal), span the width of the trough and rest on its edges. These barriers can slide across the water surface, allowing for controlled compression or expansion of the monolayer film residing at the air-water interface.

The formation process begins by dissolving the chosen amphiphile in a suitable spreading solvent. This solvent must meet several criteria: it should readily dissolve the amphiphile, be immiscible with water, have a high volatility to ensure rapid evaporation after spreading, and possess a positive spreading coefficient (S) [39, 40]. The spreading coefficient, defined as $S = \gamma_{w/a} - \gamma_{s/a} - \gamma_{w/s}$ (where γ represents interfacial tension and subscripts w, a, and s denote water, air, and solvent, respectively), ensures that the solvent spreads spontaneously across the water surface, carrying the amphiphile molecules with it. Chloroform is a commonly used spreading solvent. A precise volume of the amphiphilic solution is carefully deposited dropwise onto the clean water surface using a microsyringe. The solvent spreads, distributing the amphiphile molecules, and then evaporates, leaving behind a layer of

amphiphiles at the interface. Initially, if the surface area is large enough, the molecules are far apart and interact minimally, forming a two-dimensional gaseous phase [41].

The key parameter used to characterize the state of the Langmuir monolayer is the surface pressure (). It represents the reduction in the surface tension of the pure subphase (γ_0) caused by the presence of the monolayer (γ): $= \gamma_0 - \gamma$. Surface pressure is the two-dimensional analogue of pressure in three dimensions and is typically measured in units of millinewtons per meter (mN/m). It quantifies the repulsive force exerted by the monolayer molecules against confinement. The most common method for measuring surface pressure is the Wilhelmy plate method [42]. A thin plate (often made of platinum or filter paper to ensure zero contact angle, $\theta = 0$) is suspended from a sensitive microbalance and partially immersed through the interface. The downward force (F) exerted on the plate due to surface tension is measured, and the surface tension is calculated as $\gamma = F/(l \cos \theta)$, where l is the wetted perimeter of the plate. Assuming $\theta = 0$, $\gamma = F/l$. By measuring the force before and after spreading the monolayer, the surface pressure can be determined.

By systematically moving the barriers inwards, the area available to the amphiphiles is reduced, forcing them closer together. Recording the surface pressure as a function of the area per molecule (A) during this compression process yields a surface pressure-area (-A) isotherm. This isotherm provides invaluable information about the phase behavior, packing density, and compressibility of the monolayer at a constant temperature [43, 41]. Several distinct phases can typically be identified on a -A isotherm, analogous to the states of matter in 3D:

- **Gaseous (G) phase:** At very large areas per molecule, is close to zero. Molecules are far apart, move freely, and interactions are negligible.

- **Liquid-Expanded (LE) phase:** As the area is reduced, begins to rise. Molecules start to interact, but the hydrophobic tails remain relatively disordered and have significant motional freedom. The isotherm slope is relatively shallow.

- **Liquid-Condensed (LC) phase:** Further compression leads to a steeper increase in . Molecules are more closely packed, and the hydrophobic tails become more ordered, often tilted with respect to the surface normal. This phase is less compressible than the LE phase.

The transition between LE and LC phases often manifests as a region of near-constant surface pressure (a plateau, characteristic of a first-order phase transition) or a distinct change in the isotherm slope, reflecting the coexistence or transformation between these two liquid phases, as schematically shown in Figure 4.

- **Solid (S) phase:** At smaller areas per molecule, the isotherm becomes very steep, indicating low compressibility. Molecules are tightly packed in a quasi-crystalline arrangement, with tails often oriented nearly perpendicular to the interface [44].
- **Collapse phase:** Beyond a certain critical pressure (collapse pressure), the monolayer structure becomes unstable. Further compression forces molecules out of the monolayer plane, leading to the formation of 3D structures like bilayers or aggregates, or causing the film to buckle and fold [45, 46]. The surface pressure may drop, plateau, or fluctuate erratically in this region.

The compressibility or elasticity of the monolayer in its plane can be quantified by the surface compressional modulus, Cs^{1}, which is the inverse of the surface compressibility (Cs), and is defined at a constant temperature T as Cs^{1} = -A $(d/dA)_T$. It represents the resistance of the monolayer to compression. Cs^{1} values vary significantly between phases: very low for the G phase, intermediate for LE and LC phases (typically 12.5-50 mN/m for LE, 100-250 mN/m for LC) [41, 43], and very high for the S phase (¿ 250 mN/m). These ranges provide a quantitative basis for phase identification.

In addition to surface pressure measurements, other techniques are employed to characterize Langmuir monolayers. For instance, Brewster Angle Microscopy (BAM) is a powerful optical technique that allows for the direct visualization of the monolayer morphology at the air-water interface without the need for labels [47]. BAM can reveal domain structures, phase coexistence, and defects within the monolayer, providing visual confirmation of the different phases (G, LE, LC, S) inferred from the -A isotherm [48, 38]. Figure 8 provides an example of BAM images, illustrating how the monolayer's morphology (e.g., domain size, shape, homogeneity) can change with surface pressure and how it can be significantly affected by experimental conditions such as the pH of the subphase [49]. A detailed discussion of various characterization techniques, including BAM [50], surface potential measurements, spectroscopic methods, and scattering techniques, will be presented

in Section 4.

The stability, organization, and phase behavior of Langmuir monolayers are sensitive to several experimental parameters. Temperature affects molecular motion and fluidity, influencing phase transition points and monolayer stability [51, 43]. The pH of the aqueous subphase is critical for ionizable amphiphiles like fatty acids, as it determines the degree of head group ionization, which in turn affects intermolecular electrostatic interactions and interactions with the subphase [52]. The ionic strength and the presence of specific ions (e.g., divalent cations like Ca^{2} or Cd^{2}) in the subphase can significantly alter monolayer properties by screening charges, binding to head groups, forming salt bridges [53, 54], and influencing molecular packing and stability [55]. Additives dissolved in the subphase or co-spread with the primary amphiphile can also modify the monolayer structure and properties [56, 57, 58].

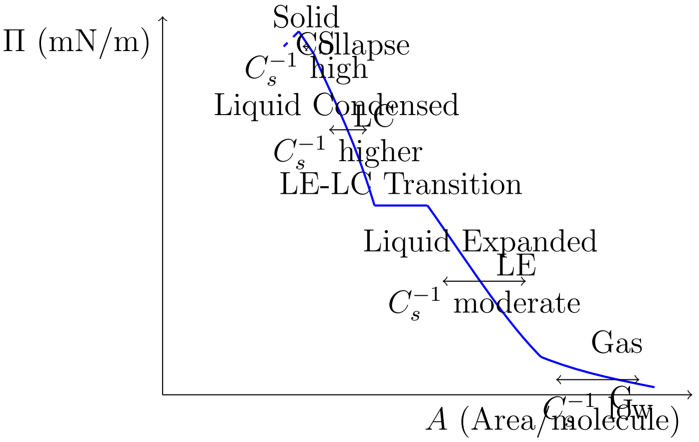

Figure 4: Schematic representation of a typical surface pressure-area (Π-A) isotherm for a Langmuir monolayer, showing different two-dimensional phases (Gas - G, Liquid Expanded - LE, Liquid Condensed - LC, Solid - S) and phase transitions. The relative compressional modulus (C_s^{-1}) increases as the monolayer becomes more condensed.

2.3 Monolayer Transfer: The Langmuir-Blodgett and Langmuir-Schaefer Techniques

Once a stable Langmuir monolayer is formed and compressed to a desired surface pressure (typically within a condensed phase like LC or S, where the film is coherent but below the collapse pressure), it can be transferred onto a solid substrate. This transfer process is the essence of the Langmuir-Blodgett (LB) technique, pioneered by Katharine Blodgett. The most common method is vertical deposition. A solid substrate (e.g., glass, silicon wafer, quartz, mica) is slowly dipped into and withdrawn from the subphase, passing vertically through the monolayer at the air-water interface. During this process, the monolayer adheres to the substrate surface. To ensure continuous and uniform transfer, the surface pressure of the monolayer is usually maintained constant by actively adjusting the position of the movable barriers to compensate for the area lost to the substrate.

The nature of the first layer transferred depends on the hydrophilicity of the substrate. For a hydrophilic substrate (e.g., clean glass or silicon), the first transfer typically occurs during the upward stroke (withdrawal or emersion) [5, 28]. The hydrophilic head groups of the amphiphiles adhere to the hydrophilic substrate surface, leaving the hydrophobic tails exposed to the air. Subsequent dipping (downstroke or immersion) transfers another layer with tails contacting the previously transferred tails, and the next withdrawal (upstroke) adds another layer with heads contacting the substrate-bound heads. For a hydrophobic substrate, the first layer is transferred during the initial downstroke, with the hydrophobic tails adhering to the surface, leaving the hydrophilic heads exposed to the water [59].

The efficiency of the transfer is quantified by the transfer ratio (TR), defined as the ratio of the decrease in the monolayer area on the trough surface (measured by barrier movement) to the surface area of the substrate coated during a single pass. A TR value close to unity indicates a successful, complete transfer of the monolayer without significant rearrangement or loss. Ideally, Y-type deposition yields TR 1 for both downstroke and upstroke, while perfect X-type deposition shows TR 1 for the downstroke and TR 0 for the upstroke, and ideal Z-type deposition results in TR 0 for the downstroke and TR 1 for the upstroke. A TR significantly different from the ideal values suggests incomplete transfer, film rearrangement, or potential issues with adhesion [60].

By repeating the dipping and withdrawal cycles, multilayer films can be

constructed on the substrate [61]. The precise architecture of the multilayer film depends on whether the transfer occurs during the downstroke, upstroke, or both. This leads to different structural types:

- **Y-type deposition:** Transfer occurs on both the downstroke and the upstroke. This is the most common type for typical amphiphiles on hydrophilic substrates and results in a centrosymmetric bilayer structure with head-to-head and tail-to-tail arrangement between adjacent layers. Figure 5 schematically illustrates this process.

- **X-type deposition:** Transfer occurs only during the immersion (downstroke). This leads to a non-centrosymmetric structure where, after the first layer, subsequent layers are added with the same orientation relative to the substrate (e.g., all head groups pointing away from the substrate if the first layer had tails down, resulting in a net dipole moment perpendicular to the substrate).

- **Z-type deposition:** Transfer occurs only during the emersion (upstroke). This also results in a non-centrosymmetric film, but with the molecular orientation opposite to that of an X-type film formed under similar initial conditions (e.g., all head groups pointing towards the substrate if the first layer had heads down, leading to an opposite net dipole moment compared to X-type).

The type of deposition depends on the specific amphiphile, substrate, subphase conditions (pH, ions), and deposition parameters (speed, pressure). X- and Z-type films are particularly interesting for applications requiring non-centrosymmetric structures, such as pyroelectric or piezoelectric devices and second-harmonic generation [62].

An alternative transfer method is the Langmuir-Schaefer (LS) technique, or horizontal deposition [63, 64]. In this method, the substrate is held horizontally and brought into contact with the surface of the compressed monolayer. The monolayer adheres to the substrate upon contact. The substrate is then carefully lifted away. The LS method is particularly useful for transferring rigid monolayers that might be damaged by the shear forces involved in vertical dipping, or when only a single layer deposition is desired with a specific orientation (typically tails down onto a hydrophobic substrate or heads down onto a hydrophilic substrate touched from below). Multilayer LS films can also be built by repeating the horizontal touching procedure, often with drying steps in between.

The success and quality of both LB and LS transfer depend critically on several factors. The chemical structure of the amphiphile influences its rigidity and intermolecular interactions, affecting film cohesion and adhesion. The composition of the subphase (pH, ionic strength, specific ions) plays a crucial role by modifying head group interactions and potentially mediating adhesion to the substrate (e.g., via ionic bridges) [65]. The properties of the solid substrate, including its chemical nature (hydrophilicity/hydrophobicity) [59], cleanliness, and surface roughness, are paramount for good adhesion. Operational parameters such as the dipping or lifting speed and the surface pressure at which the transfer is performed must be carefully optimized. Too high a speed can lead to defects, while incorrect surface pressure can result in poor transfer or film instability. Sometimes, drying the substrate between successive depositions is necessary to improve multilayer formation. Challenges can arise, such as poor adhesion leading to previously deposited layers being removed during subsequent passes (negative transfer ratio), requiring careful tuning of experimental conditions. Controlling these factors is essential for fabricating uniform, well-ordered LB films with desired molecular orientations and thicknesses [2, 9, 3].

3 Materials for Langmuir and Langmuir-Blodgett Films

The formation of stable Langmuir monolayers at the air-water interface and their subsequent transfer onto solid substrates as Langmuir-Blodgett (LB) films critically depend on the molecular or particulate nature of the film-forming material. Historically, the development of the field was intrinsically linked to the study of simple amphiphilic molecules. However, the quest for enhanced film properties, robustness, and specific functionalities for diverse applications has driven the exploration and incorporation of a vast array of novel and hybrid materials into the Langmuir and LB repertoire. The choice of material dictates not only the feasibility of film formation and transfer but also the ultimate structure, properties, and potential applications of the resulting ultrathin films.

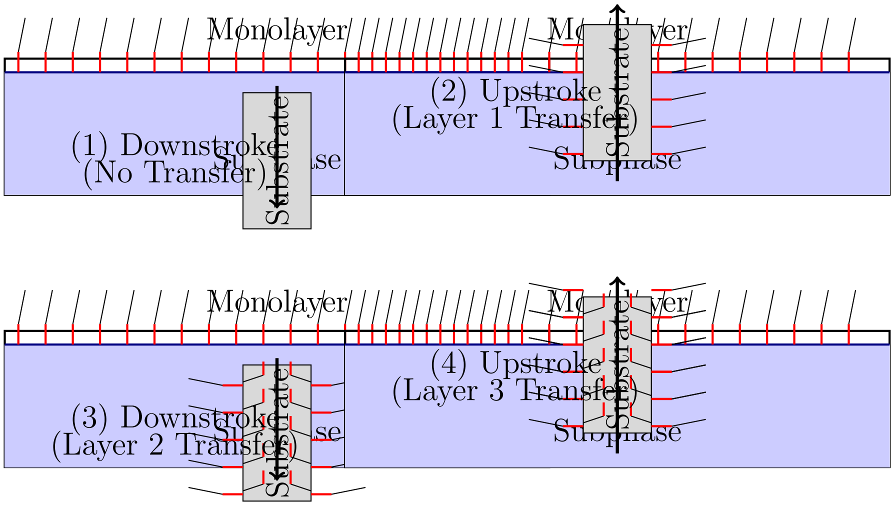

Figure 5: Schematic illustration of the Langmuir-Blodgett (LB) vertical deposition process for forming a Y-type multilayer film on a hydrophilic substrate. (1) Initial downstroke often results in no transfer. (2) First upstroke transfers the initial monolayer (Layer 1) with head groups towards the substrate. (3) Subsequent downstroke transfers Layer 2 with hydrophobic tails facing Layer 1 tails. (4) Following upstroke transfers Layer 3 with head groups facing Layer 2 head groups, establishing the head-to-head and tail-to-tail Y-type structure.

3.1 Traditional Amphiphiles

The foundational materials for Langmuir and LB studies are traditional amphiphiles. These molecules possess a dual chemical nature, characterized by a hydrophilic (water-loving) head group and a hydrophobic (water-repelling) tail, typically a long hydrocarbon chain. This amphiphilicity is the key requirement for forming stable monolayers at the air-water interface: the hydrophilic head group anchors the molecule to the water subphase through interactions like hydrogen bonding or dipole-dipole forces, while the hydrophobic tail extends away from the water, usually into the air. For a stable Langmuir monolayer to form, the molecule must also be largely insoluble in the subphase, preventing dissolution and ensuring it remains confined to the interface.

The earliest and most extensively studied traditional amphiphiles include fatty acids and their derivatives [66]. Fatty acids are aliphatic monocar-

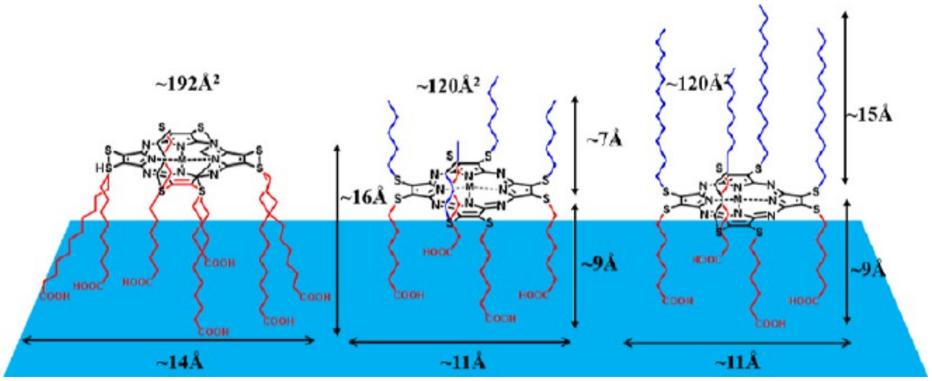

Figure 6: Proposed face-on conformers at the air—water interface of a previously reported octaacid TAP (left) with alkyl spacers of 10 methylene groups and the two tetraacid TAPs 4a (center) and 4b (right) reported here. Estimated chain lengths and TAP surface areas are taken from previous studies. The side-chain and spacer lengths represent maximum values for fully extended (crystalline) alkyl chains. Side chains in an amorphous state have a 10—20% shorter effective length. Adapted from Alinia, Z., et al., 2024 [27].

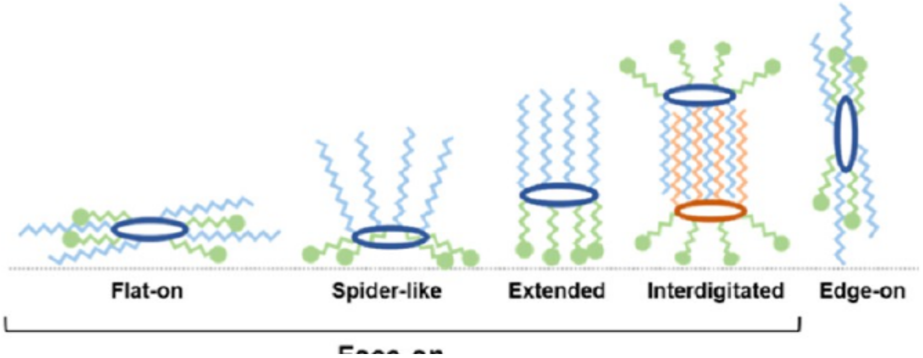

Figure 7: Schematic representation of possible interfacial orientations for 4b. Adapted from Alinia, Z., et al., 2024 [27].

boxylic acids, commonly featuring unbranched hydrocarbon chains ranging from 4 to 28 carbon atoms. Examples include stearic acid (SA) and arachidic acid (AA), which have been fundamental materials since the inception of the technique. Their structure, a polar carboxylic acid head group attached to a nonpolar alkyl chain, perfectly embodies the amphiphilic re-

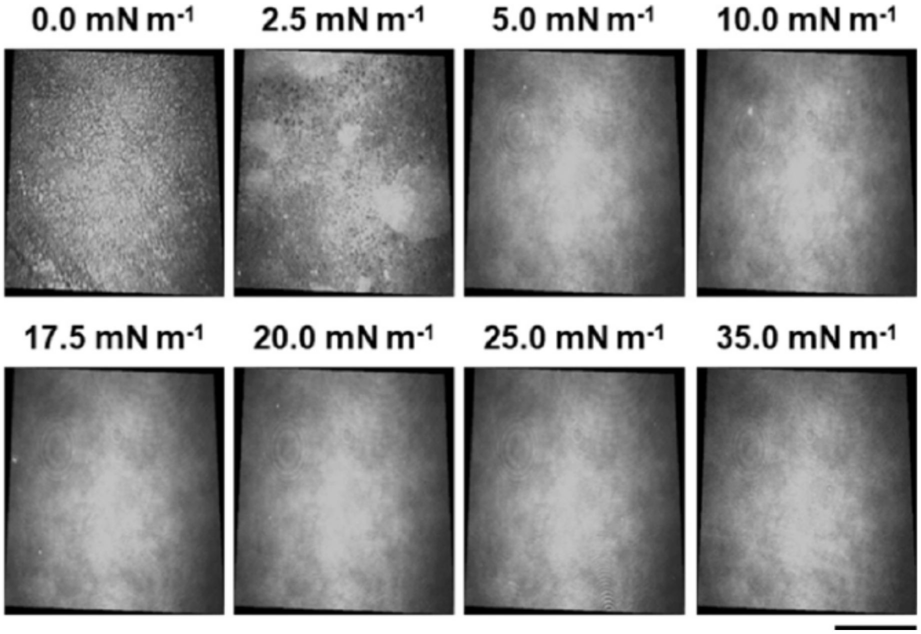

Figure 8: Representative BAM images (620 μm X 520 μm) of 4b at different subphase pH values of 4 (top), 6.5 (middle), and 11 (bottom) and surface pressures from 0.0 to 35.0 mN mˆ! (brightness of –30% and contrast of +20% modification). Adapted from Alinia, Z., et al., 2024 [27].

quirement. Variations in chain length and the presence of double or triple bonds (unsaturation) significantly influence the packing behavior, phase transitions, and fluidity of the resulting Langmuir monolayers [67]. Phospholipids, the primary components of biological cell membranes, represent another crucial class of traditional amphiphiles [68, 69, 70]. Molecules like 1,2-dipalmitoyl-sn-glycero-3-phosphocholine (DPPC) [58, 71, 72, 73], 1,2-dipalmitoyl-sn-glycero-3-phosphoethanolamine (DPPE), and 1,2-dioleoyl-sn-glycero-3-phosphocholine (DOPC) [74] consist of a hydrophilic phosphate-containing head group and two hydrophobic fatty acid tails. Their behavior at the air-water interface has been instrumental in developing Langmuir monolayers as simplified models for biological membranes [75]. Other lipids, such as cholesterol (Chol) [72, 76] and sphingomyelin (SM), often incorporated into mixed monolayers to mimic membrane complexity, also fall under this category. Surfactants, broadly defined as molecules that lower surface tension, encompass many traditional amphiphiles used in Langmuir and LB

work, including fatty acids and synthetic molecules designed with specific head and tail groups [77].

The primary advantage of using traditional amphiphiles lies in their well-characterized behavior at the air-water interface. Decades of research have provided a deep understanding of their phase behavior, packing arrangements, and interactions [8], allowing for the formation of highly ordered, densely packed Langmuir monolayers with predictable properties [78]. Their inherent biocompatibility, particularly for lipids and fatty acids [66], makes them ideal for applications in biomimetic systems [70, 58], biosensors [79, 80], and studies of biomolecular interactions at interfaces [81, 82, 83]. However, LB films derived solely from these small amphiphilic molecules often suffer from significant limitations. They tend to be mechanically fragile, lacking the robustness required for many practical device applications. Furthermore, their intrinsic electrical and optical properties are often insufficient for advanced electronic or photonic functionalities. These drawbacks spurred the investigation of alternative materials capable of overcoming these limitations while leveraging the organizational control offered by the LB technique [9, 7, 35].

3.2 Novel Materials

The limitations inherent in traditional amphiphiles prompted researchers to extend the Langmuir and LB techniques to a diverse range of materials beyond classical small amphiphiles, often termed ”nontypical amphiphiles” or even materials not inherently amphiphilic. This expansion was largely driven by the need for enhanced mechanical stability, tailored electronic or optical properties, and specific functionalities required for advanced applications in sensors, electronics, and nanotechnology. This category includes polymers, various nanomaterials, framework compounds, and other functional molecules. Some of these materials, like graphene oxide (GO), possess inherent amphiphilic characteristics due to oxygen-containing functional groups introduced during synthesis, facilitating their processing at the air-water interface. Others, which are intrinsically nonamphiphilic or poorly soluble, often require chemical modification (e.g., functionalization of carbon nanotubes with polymers like PANI or alkylation of hydrophilic molecules) or co-spreading with traditional amphiphiles or surfactants (”helper molecules”) to induce interfacial activity and enable the formation of stable Langmuir films.

Polymers emerged as highly attractive candidates from the 1980s onwards, primarily due to the potential for creating mechanically robust and functional films. Both preformed polymers and polymerizable monomers have been successfully employed. Preformed polymers, if possessing suitable solubility and interfacial activity (sometimes induced by modification with hydrophobic moieties), can be spread directly. Alternatively, monomers can be spread and subsequently polymerized either at the air-water interface before transfer (in situ polymerization) or on the solid substrate after transfer. This versatility allows for the incorporation of various functionalities. Conducting polymers, such as poly(p-phenylene vinylene) (PPV) and polyaniline (PANI), were extensively studied for applications in molecular electronics and sensors [84]. Other polymers like poly(vinylidene fluoride) (PVDF) [85] and polyfluorenes have been used for dielectric or light-emitting applications. The LB technique also enables the assembly of supramolecular polymers, which are formed through non-covalent interactions [86, 87, 88]. This is significant for nanoarchitectonics [89, 3, 35], allowing the construction of dynamic and potentially stimuli-responsive architectures at interfaces [90], for example, in creating electrocatalytic surfaces using materials like triphenylene-fused nickel bis(dithiolene) polymers [91]. While offering enhanced stability and functionality, polymers can present challenges in terms of solubility in appropriate volatile solvents (like chloroform), achieving uniform spreading without aggregation, and potential film rigidity hindering efficient transfer.

The rise of nanotechnology has introduced a plethora of nanomaterials into the realm of LB films. Carbon-based nanomaterials, including single-walled and multi-walled carbon nanotubes (SWCNTs, MWCNTs), graphene, graphene oxide (GO), and fullerenes, have been widely explored [92]. GO, with its oxygen-containing functional groups, exhibits amphiphilic character, facilitating its dispersion and organization at the air-water interface [93], although its conductivity is lower than pristine graphene. Carbon nanotubes often require functionalization or co-spreading with matrix molecules (like polymers or surfactants) to form stable Langmuir films. These materials offer exceptional electrical, thermal, and mechanical properties for applications in composites, sensors [94], and energy storage [95]. Inorganic nanomaterials constitute another significant category. Metal nanoparticles (e.g., gold (Au), silver (Ag)) are used for plasmonic sensing (SERS) [96] and catalysis. Semiconductor nanoparticles or quantum dots (e.g., Cadmium Sulfide (CdS), Tin Oxide (SnO2)) offer tunable optical and electronic properties for sensors

and optoelectronics. Metal oxides (e.g., Manganese Dioxide (MnO2), Cobalt Oxide (Co3O4), tungsten trioxide) find use in energy storage and electrocatalysis. Silica (SiO2) nanoparticles have been employed, for instance, as templates for creating nanostructured surfaces. Two-dimensional materials beyond graphene, such as molybdenum disulfide (MoS2) and black phosphorus nanosheets (BPNS), offer unique layer-dependent electronic and optical characteristics attractive for electronics and sensing [97, 98]. Other nanostructures like inorganic nanowires (e.g., Zinc Oxide (ZnO)) and clay nanosheets have also been successfully incorporated into LB films, often exhibiting alignment induced by the compression and transfer process.

Emerging porous materials like metal-organic frameworks (MOFs) [90, 99, 100] and covalent organic frameworks (COFs) [101, 102, 103, 104] represent a newer frontier for LB films. Their high surface area and tunable pore structures make them promising for applications in gas sensing, separation, and catalysis. Integrating these crystalline frameworks into ordered thin films via LB methods is an active area of research [35, 105, 106]. Other nontypical materials successfully formed into Langmuir and LB films include macrocycles like phthalocyanines [14] and porphyrins [13, 107, 108] (often used in sensors), dendrimers [109], functionalized ionic liquids, and organic-inorganic hybrids like diureasils.

Fabricating Langmuir and LB films from these novel materials often involves overcoming significant challenges. Many require strategies to induce interfacial activity. Achieving true monolayer formation can be difficult, as cohesive forces may lead to the formation of 3D aggregates rather than a uniform 2D film at the interface [45]. Characterization techniques are crucial to understand the behavior of these materials at the interface. For example, surface pressure-area isotherms, as shown for molecule '4b' in Figure 9, reveal the different phases (gas, liquid-expanded, liquid-condensed, solid) the material passes through upon compression at the air-water interface. The shape of the isotherm and the compressibility modulus (inset of Figure 9) provide information about the packing density, stability, and phase transitions, which can be influenced by factors like subphase pH [49]. Brewster Angle Microscopy (BAM) provides direct visualization of the monolayer morphology during compression [47]. The BAM images in Figure 10 for molecule '4b' show the evolution of domains and film homogeneity at different surface pressures and pH values, complementing the isotherm data. Ensuring efficient and uniform transfer onto solid substrates can also be problematic, especially if the interfacial film is too rigid or poorly ordered. Atomic Force Microscopy

(AFM) is essential for characterizing the morphology of the transferred LB film [110, 111]. Figure 11 shows AFM images of molecule '4a' transferred onto mica at increasing surface pressures. These images reveal how the film structure, domain size, and surface coverage on the solid substrate evolve with the deposition pressure, illustrating the degree of organization achieved in the final LB film. Despite the hurdles, the successful incorporation of these diverse materials has vastly expanded the functional capabilities of LB films.

3.3 Hybrid and Composite Materials

A powerful strategy to further enhance the functionality and performance of LB films involves the creation of hybrid or composite structures, where two or more distinct materials are combined within the same film. This approach allows for the synergistic integration of properties from different components, leading to materials with tailored characteristics that may not be achievable with single-component films. Hybrid LB films can be fabricated by co-spreading a mixture of components dissolved in the spreading solvent onto the air-water interface to form a mixed Langmuir monolayer, or by sequential deposition of different monolayers layer-by-layer onto the solid substrate.

One major category involves combining polymers with nanomaterials. For instance, polymer matrices can improve the dispersion and film-forming ability of nanomaterials like carbon nanotubes or graphene, while the nanomaterials impart enhanced electrical conductivity, mechanical strength, or sensing capabilities to the polymer film [112, 97]. Examples include composites of PANI with MWCNTs (PANI@MWCNTs), SWCNTs embedded in polymer matrices [113], reduced graphene oxide (RGO) combined with SnO2 nanoparticles for battery anodes [95], and PVDF films incorporating inorganic nanosheets for dielectric applications [114, 85]. Traditional amphiphiles like stearic acid have also been used as structure-directing agents or matrices for polymers like PPV, facilitating the formation of stable, ordered Langmuir films for optical applications [115].

Lipid-based hybrid films are particularly relevant for biomimetic applications and biosensing [116, 117]. Incorporating proteins, enzymes, or antibodies into lipid monolayers or bilayers allows for the creation of functional biointerfaces [118, 119]. For example, glucose oxidase (GOx) has been incorporated with gold nanoparticles (AuNP) [120] or within phospholipid (e.g., DPPE) monolayers to create glucose sensors [121, 122]. The lipid matrix

provides a biocompatible environment that helps maintain the activity of the embedded biomolecules [123]. Mixed lipid systems, incorporating cholesterol [72, 76] or different phospholipids [69], are used to create more realistic cell membrane models for studying drug interactions or membrane protein behavior [124].

Organic-inorganic hybrid films combine the functionalities of organic molecules (e.g., dyes, polymers, ligands) with inorganic components (e.g., nanoparticles, nanosheets, metal complexes) [125, 126]. Examples include composite films of MoS2 nanosheets and gold nanoparticles for biosensing, black phosphorus nanosheets (BPNS) functionalized with dye molecules for gas sensing, functionalized gold nanoparticles combined with lutetium bisphthalocyanine for electrocatalysis [127], and carbazole derivatives complexed with nickel phthalocyanine (NiPc) for photoelectrochemical applications. Heterogeneous films containing platinum (Pt) nanoclusters and graphene oxide (GO) have been developed for electrocatalysis [128]. Mixtures of different amphiphiles, such as fatty acids and perfluorinated fatty acids, have been used to control the structure and properties of polymerized films like polydiacetylenes [129].

The development of fatty acid-based hybrid materials has garnered significant recent interest [66, 130, 131, 132]. This includes mixing fatty acids with polymers [80, 133, 134, 135], nanoparticles [136, 17, 137, 138], or other surfactants to tailor Langmuir film properties and the characteristics of the resulting LB films for applications ranging from optical devices [26, 139, 140] and energy storage [17] to environmental remediation and biomedical studies [118, 141, 142, 74].

Hybrid LB films offer numerous advantages. They allow for the combination of structural integrity from one component (e.g., a polymer or lipid matrix) with the specific functionality of another (e.g., the catalytic activity of nanoparticles, the sensing capability of a dye, or the biological activity of an enzyme). They can lead to enhanced performance through synergistic effects between components. Furthermore, the LB technique provides precise control over the composition and architecture of these multicomponent films at the nanoscale, both in the Langmuir monolayer at the interface and in the transferred LB film, enabling the rational design of complex functional interfaces and nanoarchitectures.

Table 1: Overview of Material Classes Used in Langmuir and Langmuir-Blodgett Films.

**Material Class**	**Examples**	**Key Features / Applications**
Traditional Amphiphiles	Fatty acids (Stearic Acid, Arachidic Acid), Phospholipids (DPPC, DPPE, DOPC), Cholesterol, Sphingomyelin, Surfactants	Well-defined amphiphilicity, Ordered monolayers, Biomembrane models, Biocompatibility
Polymers	PPV, PANI, PVDF, Polydiacetylenes, Polyfluorenes, Supramolecular polymers	Mechanical robustness, Diverse functionalities (conducting, optical, dielectric), Processability challenges, Nanoarchitectonics
Carbon Nanomaterials	CNTs (SWCNT, MWCNT), Graphene, Graphene Oxide (GO), Fullerenes	High conductivity, Mechanical strength, Large surface area, Electronics, Composites, Sensors
Inorganic Nanoparticles	Au, Ag, CdS, SnO2, MnO2, Co3O4, SiO2, ZnO, Tungsten trioxide	Plasmonics (SERS), Catalysis, Sensing, Energy storage, Optoelectronics, Templating
2D Materials	MoS2, Black Phosphorus Nanosheets (BPNS), Clay nanosheets	Layer-dependent electronic/optical properties, Sensing, Electronics, Catalysis
Framework Materials	MOFs, COFs	High porosity, Tunable structures, Gas sensing, Separation, Catalysis
Other Nontypicals	Phthalocyanines, Porphyrins, Dendrimers, Ionic Liquids, Diureasils	Specific molecular recognition, Sensing, Catalysis, Molecular electronics
Hybrid Materials	Polymer/NP composites, Lipid/Protein films, Organic/Inorganic heterostructures, Fatty acid hybrids (e.g., RGO/SnO2, BPNS/dye, GOx/AuNP)	Synergistic properties, Tailored functionality, Enhanced stability, Complex nanoarchitectures, Biosensing, Energy

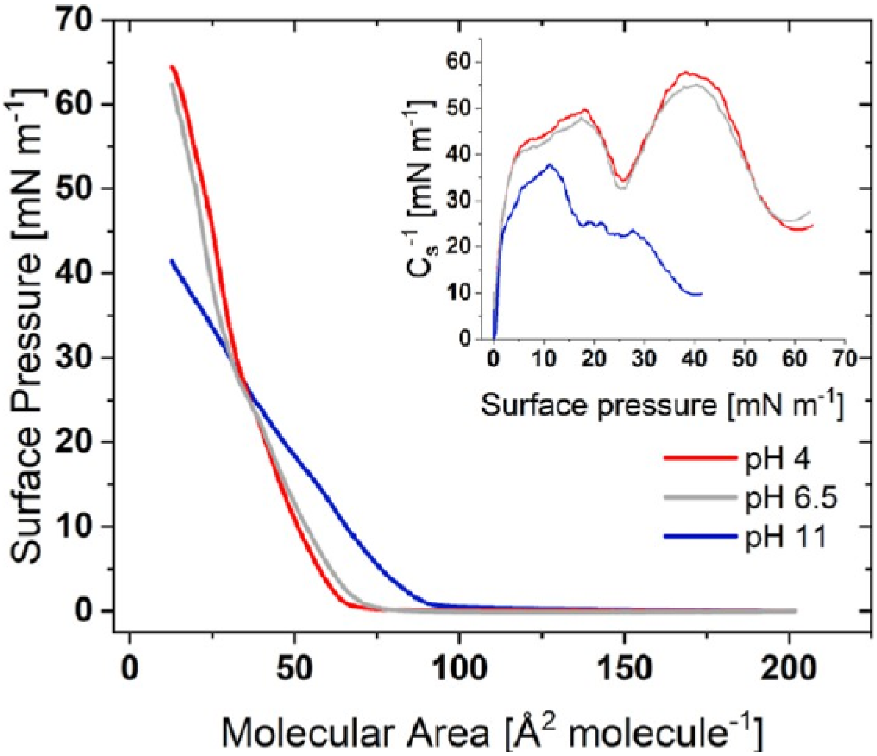

Figure 9: Surface pressure—molecular area isotherms of 4b at subphase pHs of 4, 6.5, and 11 at a compression rate of 10 cm^2 min^{-1}. Compressibility modulus plot (inset) of 4b at different subphase pH with a compression rate of 10 cm^2 min^{-1}, 22 °C. Adapted from Alinia, Z., et al., 2024 [27].

4 Characterization Techniques for Langmuir and Langmuir-Blodgett Films

The successful fabrication and application of Langmuir and Langmuir-Blodgett (LB) films rely heavily on comprehensive characterization techniques [2, 9, 8]. These methods provide crucial insights into the film's properties at various stages, from the floating monolayer at the air-water interface to the transferred film on a solid substrate. Characterization encompasses the determination of thermodynamic properties, morphology, chemical composition, molecular orientation [11], and structural order [78]. A combination of techniques is often necessary to obtain a complete understanding of these

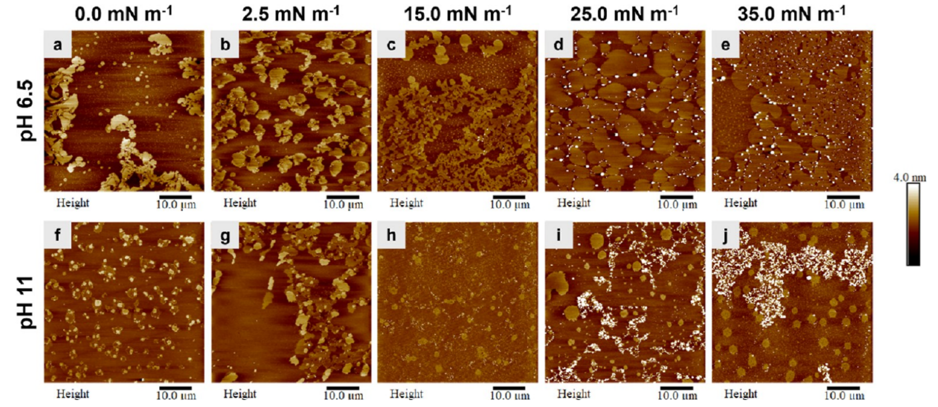

Figure 10: Representative BAM images (620 μm X 520 μm) of 4b at different subphase pH values of 4 (top), 6.5 (middle), and 11 (bottom) and surface pressures from 0.0 to 35.0 mN m^{-1} (brightness of —30% and contrast of +20% modification). Adapted from Alinia, Z., et al., 2024 [27].

complex, ultra-thin systems. Key methods include surface pressure-area isotherm measurements, surface potential measurements [143], various microscopic and spectroscopic approaches [144, 145], and diffraction techniques [12, 49, 146], each offering unique information about the film's characteristics relevant to both Langmuir monolayers and transferred LB films.

4.1 Surface Pressure-Area Isotherms

The primary characterization method for Langmuir monolayers at the air-water interface is the measurement of the surface pressure-area (-A) isotherm [56]. This technique provides fundamental thermodynamic information about the monolayer's behavior during compression. Surface pressure () is defined as the reduction in surface tension of the pure subphase () caused by the presence of the monolayer (), i.e., = - . It is typically measured using the Wilhelmy plate method [42], where a plate (often filter paper or platinum) attached to a sensitive balance measures the force exerted by the interface. Assuming complete wetting (contact angle = 0), the surface tension is calculated from the measured force (F) and the wetted perimeter (l) of the plate using the Wilhelmy equation, = F / l. This method provides the basis for surface manometry or tensiometry in Langmuir film studies.

The -A isotherm is obtained by continuously monitoring the surface pres-

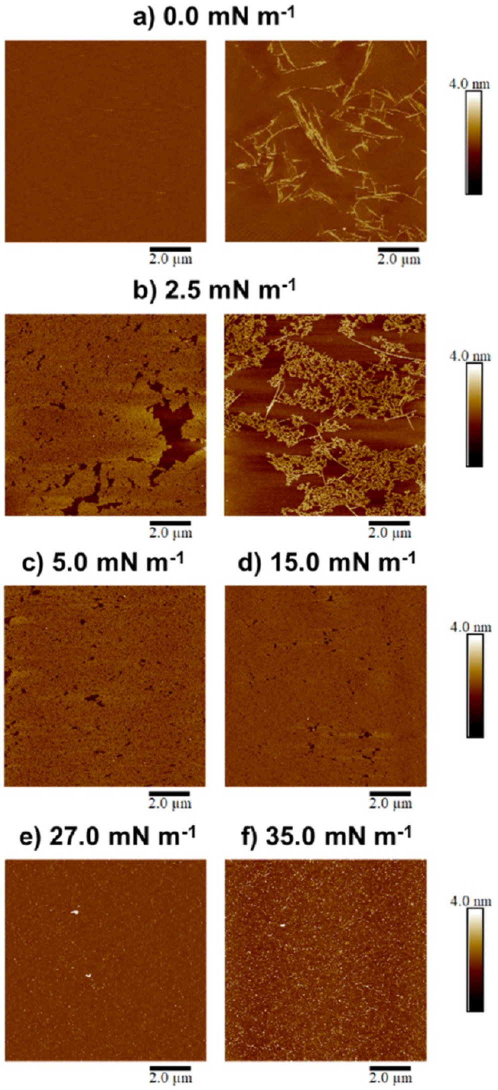

Figure 11: AFM images of 4a deposited onto mica at different surface pressures at a subphase pH of 6.5. Surface pressures from top left: (a) 0.0, (b) 2.5, (c) 5.0, (d) 15.0, (e) 25.0, and (f) 35.0 mN m^{-1}. Adapted from Alinia, Z., et al., 2024 [27].

sure as the area available to the monolayer molecules is systematically reduced by moving compressive barriers, usually at a constant temperature. The resulting plot of surface pressure versus the area per molecule (A) re-

veals distinct regions corresponding to different two-dimensional phases of the monolayer [41, 43]. At large areas per molecule, the film exists in a gaseous (G) phase, where molecules are far apart and interact weakly, resulting in near-zero surface pressure. As the area is reduced, the pressure begins to rise, potentially transitioning through a liquid-expanded (LE) phase, characterized by disordered alkyl chains and significant molecular motion, and then a liquid-condensed (LC) phase, where molecules are more closely packed with increased chain order but still possess some fluidity [147, 148]. Further compression leads to a solid (S) phase, representing a highly ordered, densely packed state analogous to a 2D crystal [44]. Beyond the solid phase, further compression causes the monolayer to collapse, often forming 3D structures, micelles, or bilayers [45, 46], indicated by a plateau or decrease in surface pressure at a high value (collapse pressure).

The shape of the -A isotherm provides valuable information about the packing, stability, and phase transitions of the monolayer [43, 148]. The compressibility modulus (), also known as the surface elasticity, can be calculated from the isotherm using the equation = -A (d/dA). This parameter quantifies the monolayer's resistance to compression and is particularly useful for identifying phase transitions. Typical values distinguish between LE (12.5–50 mN/m), LC (100–250 mN/m), and S (¿ 250 mN/m) phases for conventional amphiphiles like fatty acids or phospholipids. Isotherms are also crucial for studying interactions in mixed monolayers, where deviations from ideal mixing behavior can indicate miscibility, phase separation, or complex formation between components [56, 72]. Furthermore, -A isotherms help determine the optimal surface pressure for transferring the monolayer onto a solid substrate to form an LB film, typically chosen within a stable condensed phase (LC or S) before collapse [78]. Figure 13 illustrates typical -A isotherms for compound 4b at different subphase pH values, demonstrating how subphase conditions can influence monolayer behavior, such as the area per molecule and the collapse pressure. The inset showing the compressibility modulus () versus surface pressure () further aids in identifying phase transitions and assessing film stability under varying conditions.

However, conventional -A and - isotherm analysis has limitations, particularly for soft, flexible materials like graphene oxide (GO). GO monolayers often exhibit continuous increases in surface pressure without distinct phase transitions identifiable by plateaus or sharp changes in compressibility, as seen in Figure 13 for a more conventional amphiphile. The observed isotherms for GO can be attributed to the bending and structural deforma-

tion of the flexible GO sheets under compression, rather than traditional phase changes [149, 150]. Hysteresis between compression and expansion cycles is common, indicating irreversibility due to factors like sheet overlap or buckling [56]. In such cases, alternative analyses, such as measuring the elastic tensile modulus during expansion (——- isotherms), have been proposed by Park et al. to identify phase transitions (e.g., solid-to-liquid) marked by clear inflection points, potentially reducing the reliance on microscopic analysis for phase identification.

4.2 Surface Potential Measurements

Alongside surface pressure, surface potential (V) measurements provide crucial information about the electrical properties of Langmuir monolayers [151]. The surface potential is defined as the difference in potential between the monolayer-covered surface and the bare subphase surface. It arises from the collective contribution of the permanent electric dipole moments of the amphiphilic molecules forming the film and the reorientation of water molecules at the interface induced by the monolayer [152, 153, 154]. Measurements are typically performed using a non-contact vibrating plate electrode (Kelvin probe method) positioned above the interface and a reference electrode immersed in the subphase. Changes in surface potential during monolayer compression (V-A isotherms) complement -A isotherms, offering insights into molecular orientation, packing density, phase transitions, and interactions between monolayer components or with ions in the subphase [155]. The magnitude and sign of the surface potential depend on the orientation and magnitude of the molecular dipoles perpendicular to the interface.

4.3 Microscopic Techniques

Microscopic techniques are indispensable for visualizing the morphology, structure, and homogeneity of Langmuir monolayers at the air-water interface [156, 157] and transferred LB films on solid substrates [111, 158, 110, 159]. They provide direct spatial information at various length scales, complementing the spatially averaged data obtained from isotherms and spectroscopy.

Brewster Angle Microscopy (BAM) is a powerful, non-invasive technique for imaging Langmuir monolayers *in situ* at the air-water interface without the need for labeling probes [47, 160, 161, 162]. It operates based on the principle that p-polarized light incident at the Brewster angle (approximately 53°

for the air-water interface) is not reflected by the pure water surface. However, the presence of a monolayer changes the local refractive index, leading to some reflection. The intensity of the reflected light, captured by a camera, depends on the monolayer's thickness, density, and molecular orientation. BAM allows visualization of domain formation [67, 49], phase coexistence (e.g., LE-LC transitions in DPPC monolayers, as illustrated in Figure 9 of Oliveira Jr. et al.), film homogeneity, topography, and the adsorption of materials from the subphase [163, 164]. Different phases and domains appear with varying contrast [38, 165]. BAM can be coupled with ellipsometry [48] to provide quantitative information on domain thickness and molecular tilt angles.

Fluorescence Microscopy is another technique used for *in situ* visualization of Langmuir monolayers [166, 167, 168], but it requires the addition of a small amount of fluorescent probe molecules to the monolayer [169, 170, 171]. The probe's fluorescence emission is observed under excitation light. Contrast arises if the probe's solubility or fluorescence properties differ between monolayer phases [172, 173, 174]. For example, in DPPC monolayers undergoing an LE-LC transition, probes soluble in the LE phase but excluded from the LC phase allow visualization of dark, growing LC domains within a bright LE background. While providing valuable morphological information, a key disadvantage is that the fluorescent probe itself can potentially perturb the monolayer's structure and properties.

Atomic Force Microscopy (AFM) is a standard technique for characterizing the topography of transferred LB films on solid substrates with high spatial resolution (nanometer scale) [158, 175]. It operates by scanning a sharp tip attached to a cantilever across the sample surface. Tip-sample interactions cause cantilever deflection, which is measured and used to reconstruct a 3D topographical map. AFM can reveal surface morphology, roughness, domain structures [110, 49], film thickness (by imaging step edges), defects (pinholes, cracks), and the presence of aggregates or collapsed structures. It is particularly useful for examining the morphology of LB films made from nanomaterials, such as the wrinkled or overlapped structures observed in GO LB films (as studied by Park et al.), which can be influenced by factors like GO sheet size distribution and transfer pressure (see Figure 6 in Park et al.). AFM can also be used in specialized modes, such as single-molecule force spectroscopy (SMFS) with functionalized tips, to measure local adhesion forces, for instance, between cellulose nanocrystals deposited via LB onto AFM levers, as demonstrated by Gu et al. [176].

Electron Microscopy, including Scanning Electron Microscopy (SEM) and Transmission Electron Microscopy (TEM), provides even higher resolution imaging of transferred LB films. SEM scans a focused electron beam across the sample surface, detecting secondary or backscattered electrons to form an image of the surface topography [177]. It is widely used to observe the overall morphology, coverage, and large-scale features like wrinkles or overlaps in LB films (e.g., GO films studied by Park et al., see Figures 4 and 5). SEM typically requires samples to be conductive (often necessitating a thin coating) and under vacuum. TEM transmits an electron beam through an ultra-thin sample (or a replica), providing information about the internal structure and morphology at very high resolution [178, 179]. It is less commonly used for standard LB film characterization unless specific internal structural details are required or the LB film itself is composed of electron-transparent nanomaterials prepared on suitable grids. Both SEM and TEM are mentioned as common tools for LB film characterization by Fang et al.

4.4 Spectroscopic Techniques

Spectroscopic techniques probe the interactions of electromagnetic radiation with the film, providing information about chemical composition [180], molecular structure [181], orientation [182, 11], and electronic properties [183].

UV-Visible (UV-Vis) Absorption and Fluorescence Spectroscopy are used to study molecules containing chromophores or fluorophores [171]. UV-Vis absorption measures the attenuation of light passing through the sample as a function of wavelength, revealing electronic transitions [184, 185]. For Langmuir monolayers, reflectance spectroscopy can be performed *in situ* using fiber optic probes positioned above the interface. Changes in absorption spectra (peak position, intensity, shape) can indicate molecular aggregation (e.g., formation of H- or J-aggregates in dyes or phthalocyanines) [172, 186, 187, 188], changes in molecular environment [189], or chemical reactions. Fluorescence spectroscopy measures the light emitted by fluorescent molecules after excitation [171], providing complementary information about the electronic structure, molecular environment [169, 190, 191, 170, 172], and aggregation state [192, 193]. It is highly sensitive but often requires fluorescent probes for non-fluorescent molecules, with the associated risk of perturbation mentioned earlier. Both techniques are routinely applied to transferred LB films [134].

Infrared (IR) Spectroscopy probes molecular vibrations, providing a fingerprint of the chemical functional groups present in the film. Fourier Transform Infrared (FTIR) spectroscopy is commonly used. For Langmuir monolayers, surface-sensitive techniques like Polarization-Modulated Infrared Reflection Absorption Spectroscopy (PM-IRRAS) are employed [194, 195, 196]. PM-IRRAS measures the differential reflection of s- and p-polarized IR light, enhancing sensitivity to interfacial species and providing information on molecular orientation (e.g., tilt angle of alkyl chains) and conformation. For transferred LB films, transmission or reflection-absorption FTIR [11, 197], as well as Attenuated Total Reflectance (ATR)-FTIR [144], can be used to analyze chemical composition and molecular orientation. FTIR has been used, for example, to study the secondary structure of proteins or peptides interacting with lipid monolayers or incorporated into LB films, as shown in examples by Gu et al. (Figures 12c and 13b).

Sum-Frequency Generation (SFG) Spectroscopy is another surface-specific vibrational spectroscopy technique [198, 199]. It is inherently sensitive only to interfaces where inversion symmetry is broken, making it ideal for studying air-water or solid-air interfaces without bulk interference [200, 201]. SFG provides information about molecular orientation [202], order, and chemical identity of species at the interface [203, 204]. Techniques like PM-IRRAS [194, 195] and SFG are crucial for determining the specific orientations molecules adopt at interfaces, such as the possible arrangements depicted for a macrocycle in Figure 12. Understanding these orientations is vital as they directly influence the film's properties and interactions.

Raman Spectroscopy also probes vibrational modes but relies on inelastic scattering of light. It is complementary to IR spectroscopy, as different vibrational modes may be active in Raman versus IR. While conventional Raman scattering from monolayers is weak, Surface-Enhanced Raman Scattering (SERS) can provide enormous signal enhancement. SERS-active LB films can be fabricated by incorporating plasmonic nanoparticles (e.g., gold or silver) into the film structure, enabling highly sensitive detection and characterization of molecules within the film, as noted by Oliveira Jr. et al. and Fang et al.

X-ray Photoelectron Spectroscopy (XPS), also known as Electron Spectroscopy for Chemical Analysis (ESCA), is a surface-sensitive technique used to determine the elemental composition and chemical states of atoms within the top few nanometers of a transferred LB film. It involves irradiating the sample with X-rays and analyzing the kinetic energy of emitted photoelec-

trons. XPS is valuable for confirming film composition, identifying contaminants, analyzing surface modifications, or studying chemical changes like the reduction of graphene oxide in rGO LB films, as employed by Park et al. and mentioned by Fang et al.

4.5 Diffraction Techniques

Diffraction techniques utilize the scattering of waves (typically X-rays or neutrons) to probe the periodic arrangement of atoms or molecules, providing detailed information about the crystalline structure and molecular packing within Langmuir monolayers and LB films.

Grazing Incidence X-ray Diffraction (GIXD) is the primary technique for determining the in-plane structural order of Langmuir monolayers at the air-water interface and thin LB films on substrates. In GIXD, a highly collimated monochromatic X-ray beam impinges on the interface at a very small angle (below the critical angle for total external reflection). This creates an evanescent wave that propagates along the interface, probing primarily the monolayer structure. If the monolayer possesses two-dimensional crystalline order, the X-rays are diffracted at specific angles according to Bragg's law for a 2D lattice. Analyzing the positions and intensities of the diffraction peaks allows determination of the unit cell dimensions, lattice symmetry, coherence length of ordered domains, and information about molecular orientation, such as the tilt angle and direction of alkyl chains relative to the surface normal, complementing information from spectroscopic techniques like PM-IRRAS or SFG regarding molecular arrangement (e.g., Figure 12). Due to the small amount of scattering material in a monolayer, high-intensity synchrotron X-ray sources are often required for GIXD measurements.

X-ray Reflectivity (XRR) measures the intensity of specularly reflected X-rays as a function of the incidence angle (or momentum transfer perpendicular to the surface). The resulting reflectivity profile is sensitive to the electron density profile along the surface normal. By fitting the experimental data to theoretical models, one can determine crucial parameters such as the total film thickness, the thickness of individual layers in multilayers, surface and interfacial roughness, and electron density (related to mass density) of the film. XRR can be performed using conventional X-ray sources or synchrotrons and is applicable to both Langmuir monolayers and transferred LB films.

Neutron Reflectivity (NR) is analogous to XRR but uses neutrons in-

stead of X-rays. Neutrons interact with atomic nuclei rather than electrons. A key advantage of NR is the significant difference in neutron scattering length between hydrogen and its isotope deuterium. By selectively deuterating parts of the amphiphilic molecules or using DO instead of HO as the subphase, specific components of the monolayer or multilayer structure can be highlighted, providing enhanced contrast and detailed information about the film's composition profile, thickness, and interfacial structure. This is particularly useful for studying complex systems like lipid-protein interactions or interdiffusion in multilayer films.

Conventional X-ray Diffraction (XRD) in reflection or transmission geometry is typically used for analyzing the structure of bulk materials or thicker films. While less common for single monolayers due to weak signals, it can be applied to multilayer LB films to probe their out-of-plane structure (lamellar spacing) or identify 3D crystalline phases if present.

The combination of these diverse characterization techniques provides a powerful toolkit for understanding the complex structure-property relationships in Langmuir monolayers at interfaces and transferred Langmuir-Blodgett films, enabling their rational design and optimization for various applications.

5 Applications of Langmuir and Langmuir-Blodgett Films

The Langmuir-Blodgett technique, offering unparalleled control over molecular arrangement, film thickness, and composition at the nanoscale, has paved the way for a diverse range of applications across multiple scientific and technological domains. The ability to construct highly ordered, ultra-thin films from a wide variety of materials, including small molecules, polymers, nanoparticles, and biomolecules, allows for the tailoring of film properties to meet specific functional requirements. This section explores some of the most prominent application areas where Langmuir monolayers and transferred LB films have demonstrated significant potential or have been successfully implemented, including molecular electronics, sensing platforms, biomedical engineering, and optical/photonic devices. The unique characteristics of these films, such as precise thickness control, high degree of molecular order, potential for creating layered heterostructures, and compatibility with various

Table 2: Summary of Key Characterization Techniques for Langmuir and LB Films.

**Technique Category**	**Specific Method**	**Information Obtained**
Surface Manometry	-A Isotherms	Phase behavior (G, LE: 12.5–50, LC: 100–250, S: ¿250 mN/m), compressibility, stability, molecular area, optimal transfer pressure, component interactions.
Electrical Properties	Surface Potential (V-A Isotherms)	Molecular dipole orientation, packing density changes, phase transitions, interfacial charging, ion binding.
Microscopy	BAM	*In situ* monolayer morphology, phase coexistence, domains, homogeneity, thickness/density variations (probe-free).
	Fluorescence Microscopy	*In situ* monolayer morphology, phase coexistence, domains (requires fluorescent probe).
	AFM	Transferred film topography, roughness, thickness, domains, defects, nanostructure morphology (e.g., wrinkles).
	SEM / TEM	Transferred film morphology, coverage, defects (SEM); internal structure (TEM) at high resolution.
Spectroscopy	UV-Vis / Fluorescence	Electronic transitions, aggregation (H/J), molecular environment (requires chromophore/fluorophore or probe).
	FTIR (PM-IRRAS, ATR)	Chemical composition (functional groups), molecular orientation, conformation, secondary structure (proteins).
	SFG	Surface-specific vibrational information, molecular orientation at interfaces.
	Raman (SERS)	Vibrational modes, chemical identification (enhanced sensitivity with SERS).
	XPS	Elemental composition, chemical states, surface stoichiometry (transferred films).
Diffraction / Scattering	GIXD	In-plane crystalline structure, unit cell, molecular packing, tilt angles (monolayers, thin films).

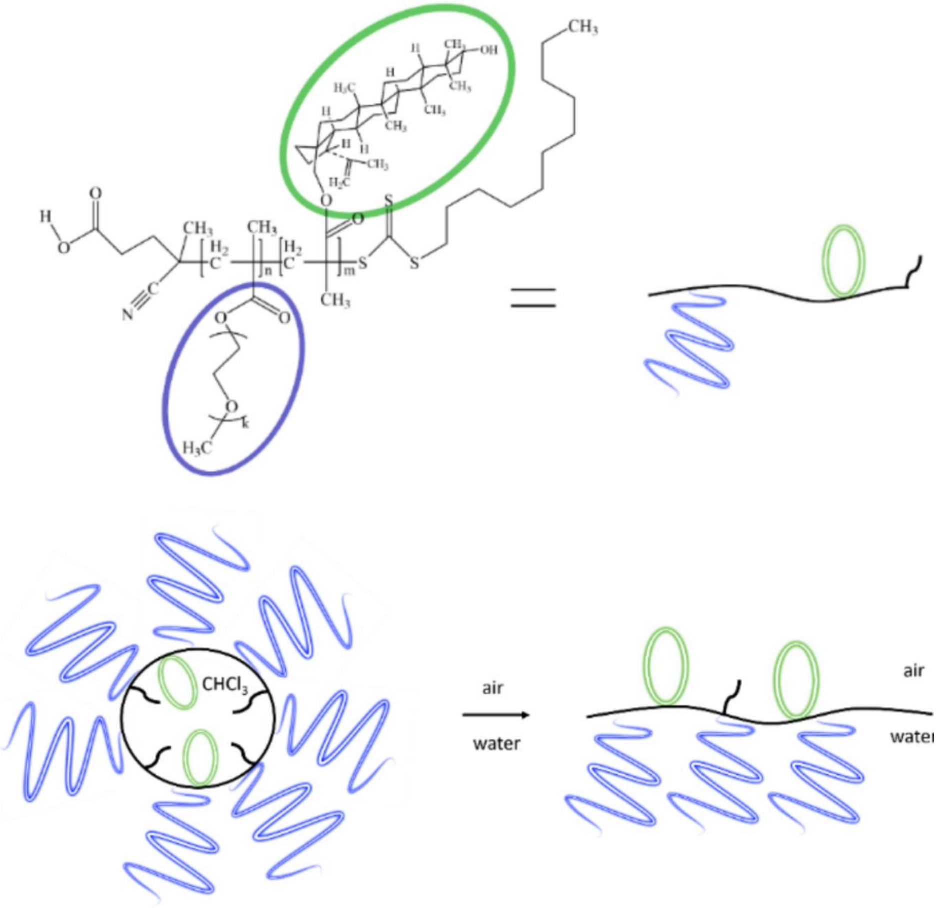

Figure 12: (right) orientations of a macrocycle at the air—water interface. Such orientations can be probed by techniques like PM-IRRAS, SFG, and GIXD. Adapted from Zamyshlyayeva, O., et al., 2024 [205].

substrates, underpin their utility in these fields.

5.1 Molecular Electronics

Interest in molecular-scale electronic components drove early research into LB films as a fabrication method offering precise molecular control. While initial concepts like molecular rectifiers demonstrated proof-of-principle, practical challenges related to defects, stability, and contacting hindered widespread adoption. Nonetheless, LB films remain relevant in organic electronics, par-

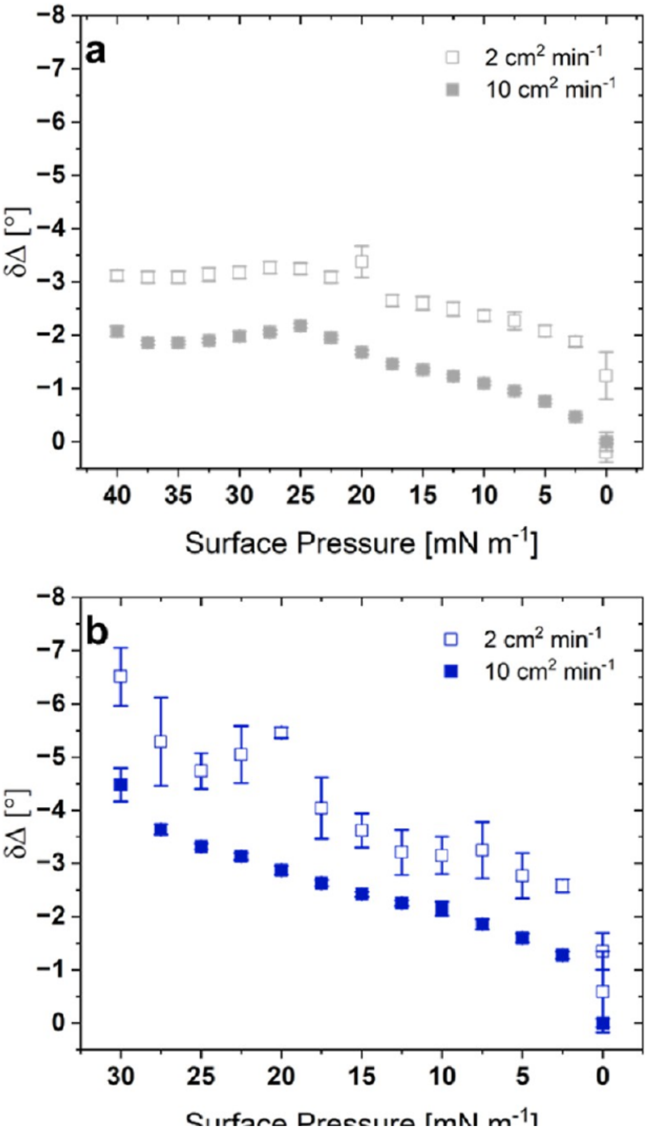

Figure 13: Surface pressure—molecular area isotherms of 4b at room temperature and at a compression rate of 10 cm^2 min^{-1}. Inset: Compressibility modulus plot (inset) of 4b at different subphase pH with a compression rate of 10 cm^2 min^{-1}, 22 C. These plots illustrate how thermodynamic data (-A) and compressibility analysis (-) reveal monolayer phases and the influence of experimental conditions like pH. Adapted from Alinia, Z., et al., 2024 [27].

ticularly for organic field-effect transistors (OFETs). The performance of OFETs is highly dependent on the structural order of the organic semiconductor layer. The LB technique enables the deposition of highly organized monolayers or multilayers, which can lead to significantly improved charge carrier mobility compared to less ordered films formed by other methods. This improvement arises because the high degree of molecular order achievable with LB deposition promotes efficient intermolecular orbital overlap, reduces grain boundaries and defects that trap or scatter charges, thus facilitating charge transport along the semiconductor channel. For instance, highly uniform and defect-minimized monolayers of materials like octadecyltrichlorosilane (ODTS) transferred via modified LB methods have enabled the fabrication of high-performance OFET arrays. Figure 14 illustrates a typical OFET architecture where the LB film serves as the crucial active semiconductor layer. This ordered molecular layer is positioned between the source and drain electrodes, and its conductivity, which dictates the current flow between these electrodes, is modulated by the electric field applied via the gate electrode through the dielectric layer. The ability to control molecular orientation (edge-on vs. face-on) within the LB film further allows for tuning charge transport properties. Moreover, the layer-by-layer nature of LB deposition facilitates the construction of complex heterostructures and is compatible with flexible substrates, opening avenues for flexible electronics, although scalability and robustness remain key considerations.

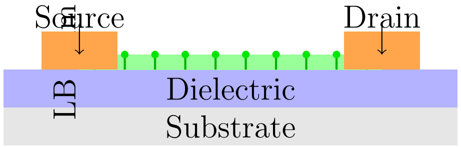

(Gate Electrode typically below substrate)

Figure 14: Schematic representation of an Organic Field-Effect Transistor (OFET) structure incorporating an LB film as the active semiconductor layer. The ordered molecular arrangement within the LB film influences charge transport between the source and drain electrodes, controlled by the gate voltage.

5.2 Sensors

Langmuir and LB films provide a versatile foundation for fabricating highly sensitive chemical and biological sensors. Their inherent high surface-area-to-volume ratio, precisely controllable nanometer-scale thickness, and ordered structure are advantageous for detecting analytes. In gas sensing, LB films allow room-temperature operation using materials like conducting polymers, phthalocyanines, carbon nanotubes (e.g., PANI@MWCNTs for ammonia detection), MOFs, or fatty acids (e.g., on SAW devices for chloroform). The ordered structure enhances interaction with gas molecules, causing detectable changes in the film's electrical, optical, or mass properties. Composite LB films further enable synergistic sensing capabilities for various gases. Figure 15 depicts the general working principle: target analytes interact with the specialized LB sensing film, inducing a change that is measured by a transducer. This interaction can range from gas molecules adsorbing onto the surface of a PANI@MWCNT film, altering its conductivity, to the specific binding of glucose molecules to glucose oxidase enzymes immobilized within the LB film matrix in a biosensor.

Chemical sensors for liquid environments also utilize LB films, for instance, in ion-sensitive field-effect transistors or as coatings on mass-sensitive devices [2]. Arrays of LB film sensors (electronic tongues) have been developed for classifying complex liquids based on their chemical profiles [206, 207, 208]. Biosensors represent a major application, leveraging the ability of LB films to create stable, biomimetic environments [25]. Lipid-based LB films effectively mimic cell membranes, providing matrices to immobilize enzymes, antibodies, or DNA while preserving their activity [118]. In enzyme-based biosensors (e.g., for glucose, urea, phenols, pesticides), the organized LB matrix plays a crucial role [121, 209, 210]. It not only provides a stabilizing biomimetic environment that helps maintain the enzyme's native conformation and activity [123, 80, 211] but can also enhance performance by facilitating efficient electron transfer between the enzyme's redox center and the transducer electrode, especially when incorporating conductive nanomaterials like gold nanoparticles (AuNPs) or carbon nanotubes (CNTs) [79]. Immunosensors similarly benefit from the controlled immobilization of antibodies on LB film surfaces for specific antigen detection (e.g., dengue NS1 antigen) [212, 213]. The precise deposition control offered by the LB technique is also instrumental in surface-enhanced Raman scattering (SERS) sensors, enabling optimized placement of analytes or plasmonic structures for

ultra-sensitive detection [214]. Furthermore, the adaptability of LB deposition allows for the creation of flexible and wearable sensors, such as MOF-based humidity sensors on fabrics.

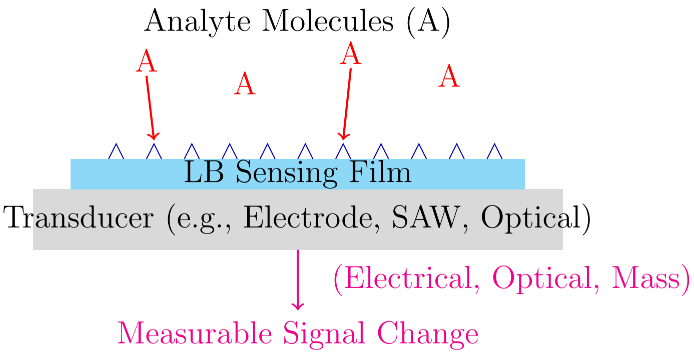

Figure 15: General principle of an LB film-based sensor. Target analyte molecules interact with the specifically designed LB sensing layer (containing receptor molecules, catalysts, or sensitive materials), causing a change in the physical or chemical properties of the film, which is detected by the underlying transducer.

5.3 Biomedical Applications

The ability to mimic biological interfaces and control nanoscale structure makes Langmuir and LB films valuable in biomedicine. A key application is the creation of model cell membranes. Langmuir monolayers, formed at the air-water interface using lipids, sterols, and proteins, serve as dynamic two-dimensional platforms for fundamental studies. They allow researchers to investigate membrane structure, fluidity, and specific molecular interactions with drugs, peptides, or nanoparticles under controlled surface pressure and composition. By transferring these Langmuir monolayers onto solid supports using the LB technique, more robust and stable biomimetic surfaces are created. These transferred LB films are suitable for integration into biosensor devices, drug screening platforms, or for more detailed structural analysis using surface-sensitive techniques.

In drug delivery, the layer-by-layer construction capability of the LB method allows for the fabrication of multilayer films containing entrapped therapeutic agents [74]. The release kinetics can potentially be tuned by

controlling the film's composition (e.g., using biodegradable lipids or polymers) [4, 215], thickness, and architecture. Such ultra-thin coatings could modify implant surfaces for localized drug release. Biocompatibility is often achievable using natural lipids or specific biocompatible polymers as film-forming materials.

Tissue engineering applications benefit from the controlled surface properties offered by LB films [2, 9]. Scaffolds coated with LB films possessing specific chemical functionalities or precisely defined nanotopographies can influence cell behavior, including adhesion, proliferation, and differentiation [216, 217, 218]. For instance, aligned nanomaterials deposited via LB methods have shown promise as scaffolds for guiding stem cell fate in regenerative medicine [219, 220, 221]. Patterned LB films could further direct tissue formation.

Bioimaging can also utilize LB films incorporating fluorescent probes [170], quantum dots [222], or contrast agents with controlled density and organization, potentially enhancing imaging sensitivity or enabling targeted imaging applications. Despite ongoing challenges related to stability in complex biological media and scalability, the precision inherent in Langmuir and LB techniques continues to drive innovation in biomedical research [25, 118, 80].

5.4 Optical and Photonic Devices

The precise control over thickness, composition, and molecular orientation afforded by the LB technique is highly advantageous for optical and photonic applications. Ultra-thin LB films serve as effective anti-reflection coatings or components in multilayer dielectric mirrors due to their well-defined refractive indices. Crucially, the orientation of optically anisotropic molecules, such as porphyrins or dyes, within the film dictates the macroscopic optical response. Controlling deposition parameters allows for inducing specific orientations, such as edge-on or face-on relative to the substrate (orientations shown in Figure **??**). This control is vital for optimizing performance in devices like OLEDs and OPVs, where molecular alignment impacts light absorption/emission and charge transport. For example, achieving a face-on orientation of porphyrin macrocycles can enhance light harvesting in photovoltaic applications.

LB films can function as planar waveguides or form part of one-dimensional photonic crystals by stacking layers with alternating refractive indices. The

technique also enables the construction of non-centrosymmetric multilayers from nonlinear optical (NLO) chromophores, essential for applications like frequency conversion (e.g., second-harmonic generation) [223, 224]. Incorporating photochromic molecules allows for light-controlled optical switching or data storage concepts [225, 226, 227]. Furthermore, the ordered assembly of photoactive molecules (dyes, complexes) in LB films facilitates the study and application of energy and electron transfer processes, relevant to artificial photosynthesis and photoelectrochemical devices [108, 228], where organized interfaces, like those in methylene blue/black phosphorus nanosheet composite LB films, can enhance performance [229, 230].

6 Challenges and Future Directions

Despite its century-long history and demonstrated capability for precise molecular assembly, Langmuir-Blodgett (LB) technology faces several significant challenges that have limited its widespread industrial application. Concurrently, ongoing research continues to unveil new strategies to overcome these limitations and explore exciting future prospects across diverse scientific and technological domains. The path forward involves addressing inherent weaknesses while leveraging the unique strengths of the technique, particularly its unparalleled control over film architecture at the molecular level, both for Langmuir monolayers at interfaces and transferred LB films.

6.1 Addressing the Limitations of LB Films

One of the most frequently cited limitations of traditional LB films, particularly those derived from small amphiphilic molecules like fatty acids, is their lack of mechanical robustness and long-term stability (Oliveira et al., 2022). These films can be susceptible to physical damage, delamination, and environmental degradation, hindering their use in demanding applications. Furthermore, the classical LB method is often perceived as complex, requiring meticulous control over experimental parameters such as subphase purity, temperature, deposition speed, and surface pressure, which contributes to challenges in reproducibility and consistency, especially over large areas. The process can be slow and difficult to scale up for mass production, making it less competitive economically compared to other thin-film deposition techniques like spin-coating, layer-by-layer (LbL) assembly, or self-assembled

monolayers (SAMs) for certain applications where molecular precision might be less critical than throughput, cost, or inherent robustness (Oliveira et al., 2022; Fang et al., 2022).

The range of materials readily amenable to forming stable Langmuir monolayers and subsequently high-quality LB films has also been a constraint. While ideal for amphiphilic molecules, spreading and organizing non-amphiphilic materials, polymers with rigid backbones, or pre-formed nanomaterials at the air-water interface can be challenging [10, 35]. Issues such as poor solubility in appropriate volatile solvents, aggregation at the interface instead of forming a true monolayer, film rigidity hindering uniform transfer, or unfavorable interactions with the subphase can arise [45]. These difficulties limit the palette of materials that can be easily incorporated into LB structures, potentially restricting the achievable functionalities. Compared to methods like LbL assembly [4, 3, 231], which can incorporate a vast array of charged water-soluble materials, or SAMs, which rely on specific chemical anchoring, the LB technique's reliance on interfacial self-assembly of spreadable materials presents distinct material selection challenges (Oliveira et al., 2022).

Significant research efforts have focused on overcoming these limitations. To enhance mechanical stability and robustness, researchers have extensively explored the use of pre-formed polymers or the in-situ polymerization of monomers within the Langmuir monolayer before or after transfer [7]. Incorporating nanoparticles, such as carbon nanotubes, graphene sheets, metal oxides, or quantum dots, into the film structure can also improve mechanical properties and add new functionalities [232, 140]. Crosslinking strategies, applied either at the air-water interface or after film deposition, can create more durable, covalently linked networks within the film. Addressing scalability and throughput has involved the development of novel trough designs, including multi-compartment systems, continuous deposition mechanisms [233], and attempts at adapting LB principles for larger substrates or even roll-to-roll processing, although significant hurdles remain for true industrial-scale production. Efforts to broaden the material scope include functionalizing non-amphiphilic molecules with hydrophobic chains to induce interfacial activity, using mixed solvent systems, employing specialized subphases, or developing alternative deposition modes like the Langmuir-Schaefer (horizontal lifting) method, which can be gentler for certain film types [63]. Advanced instrumentation, such as high-temperature LB troughs, allows for the processing of materials that require elevated temperatures for

spreading or ordering (Ariga et al., 2023). Furthermore, developing more sophisticated in-situ characterization techniques provides better feedback for optimizing deposition conditions and ensuring film quality, thereby improving reproducibility [12].

6.2 Emerging Trends and Opportunities

Despite the challenges, Langmuir and LB technology continues to find relevance and generate excitement in several emerging areas, largely driven by its unique ability to create highly ordered structures with nanoscale precision. The concept of "nanoarchitectonics," which aims to build functional material systems from nanoscale components in an organized fashion, frequently leverages the LB technique (Oliveira et al., 2022; Ariga et al., 2023). LB films provide an ideal platform for arranging molecules, polymers, nanoparticles, and 2D materials like graphene or transition metal dichalcogenides into well-defined layers and heterostructures, enabling the exploration of synergistic effects and novel functionalities arising from controlled spatial arrangement. An example of such advanced assembly is the creation of two-dimensional colloidal crystals using interfacial methods, which can then be transferred to various substrates, as illustrated in Figure 16. This demonstrates the potential for extending LB-like principles to build complex architectures from pre-formed nanoscale building blocks. This precise architectural control is crucial for applications in molecular electronics, where LB films have been used to create proof-of-concept devices like diodes and transistors, and to study fundamental charge transport phenomena across molecular layers involving complex functional molecules, such as those exhibiting redox isomerization shown in Figure 17.

The biomedical field represents a significant growth area for LB applications [15, 25]. Langmuir monolayers serve as excellent, controllable models for studying biological membranes [157, 75, 234, 70]. By varying lipid composition, incorporating proteins or peptides, and introducing drugs or other bioactive molecules into the monolayer or subphase, researchers can investigate membrane structure, fluidity, permeability, and interactions at the molecular level [235, 81, 236]. This provides invaluable insights for drug discovery, understanding disease mechanisms (like amyloid aggregation in Alzheimer's disease) [163], and designing biomimetic systems [116]. LB films themselves are being explored for creating biocompatible coatings [74], platforms for biosensors with enhanced sensitivity and selectivity due to the

Table 3: Summary of Key Challenges in LB Film Technology and Corresponding Mitigation Strategies.

Challenge	Description	Mitigation Strategies
Mechanical Instability	Films, especially from small amphiphiles, lack robustness and long-term stability.	Use of polymers, macromolecules; incorporation of nanoparticles; crosslinking techniques; composite film formation.
Material Limitations	Difficulty spreading/transferring non-amphiphilic, rigid, or poorly soluble materials.	Material functionalization (e.g., alkylation); use of solvent mixtures; specialized subphases; advanced LB troughs (e.g., high-temp); Langmuir-Schaefer deposition.
Scalability & Throughput	Traditional LB is slow, difficult for large areas or mass production.	Development of continuous deposition systems; novel large-area trough designs; multi-barrier systems; automation.
Cost & Complexity	Equipment and precise operation can be expensive and require expertise.	Simplification of instrumentation (where possible); development of user-friendly protocols; exploring cost-effective film-forming materials.
Reproducibility & Defects	Achieving consistent, defect-free films over large areas remains challenging.	Strict control over experimental parameters (purity, temp, speed); improved substrate preparation; advanced in-situ characterization and feedback loops.
Competition from Alternatives	Techniques like LbL or SAMs offer advantages in simplicity, cost, or robustness for some applications.	Focus on applications requiring LB's unique precision/order; development of hybrid approaches combining LB with other techniques; highlighting LB's versatility with diverse materials.

ordered immobilization of bioreceptors [121, 79], and scaffolds for tissue engineering where controlled surface topography and chemistry can influence cell adhesion, proliferation, and differentiation [237]. The potential to create ultrathin, ordered films on various substrates makes LB attractive for developing advanced drug delivery systems or functionalizing medical implants.

Energy storage and conversion is another promising frontier. LB techniques are being employed to fabricate ultrathin electrode materials for batteries and supercapacitors, potentially enhancing power density and cycle life [2, 137]. For instance, ordered layers of graphene [238, 113, 239], carbon nanotubes [113], or metal oxides prepared by LB can serve as high-surface-area electrodes or conductive additives. The precise control offered by LB allows for the construction of well-defined interfaces, which is critical for studying and engineering the solid-electrolyte interphase (SEI) in batteries [240, 241, 242]. Functional coatings prepared by LB could act as protective layers for electrodes, mitigating degradation mechanisms. Furthermore, the assembly of photoactive or electrocatalytic materials into ordered LB films holds potential for improving the efficiency of solar cells and electrocatalytic processes, such as water splitting or fuel oxidation, by optimizing charge separation and transport pathways [243, 244, 245].

The development of flexible and wearable electronics also presents opportunities for LB films. The ability to deposit ultrathin, ordered functional layers onto flexible substrates using modified LB techniques opens possibilities for creating sensors, transistors, and displays integrated into wearable devices or conformable surfaces [246]. Gas sensors [98, 247], humidity sensors [248], and strain sensors [249, 250, 251] based on LB films incorporating conductive polymers, nanoparticles, or 2D materials benefit from the high surface-to-volume ratio and controlled morphology achievable with the technique, leading to potentially faster response times and higher sensitivity. Combining LB with other nanofabrication methods, such as photolithography, etching, or LbL assembly [3, 252, 231], allows for the creation of more complex, hierarchical structures, integrating the molecular-level precision of LB with micro- or macro-scale patterning for sophisticated device architectures [253].

6.3 Future Research Directions

The continued evolution and impact of Langmuir and Langmuir-Blodgett technology will depend on focused research efforts addressing current limi-

tations and exploring new frontiers. A primary direction involves expanding the library of materials suitable for processing via Langmuir monolayers and subsequent LB transfer. While significant progress has been made with polymers and nanoparticles, further exploration of complex macromolecules, biomolecules (maintaining their activity), metal-organic frameworks (MOFs), covalent organic frameworks (COFs), and emerging 2D materials beyond graphene is crucial (Ariga et al., 2023). Developing strategies to form stable, transferable Langmuir films from these diverse materials, perhaps through chemical modification, innovative spreading techniques, or tailored subphase chemistry, will unlock new functionalities and applications. Research into hybrid films, combining different material classes within a single layer or in multilayer architectures, holds promise for creating multifunctional materials with synergistic properties.

Improving the practicality and scalability of the LB technique remains a critical goal for enabling wider adoption [2, 9]. This requires innovations in instrumentation, moving towards more automated, higher-throughput systems capable of handling larger substrate areas or continuous processing [254]. Reducing the cost and complexity of the equipment and operation without sacrificing precision is essential. Furthermore, enhancing the robustness and long-term stability of LB films through novel crosslinking methods, protective coatings, or embedding within more resilient matrices needs continued investigation [255]. Achieving better control over defects and improving film uniformity over large areas are persistent challenges that require ongoing research into the fundamental mechanisms of film formation at the air-water interface, monolayer collapse [45], and transfer processes.

Advanced characterization techniques are indispensable for progress. There is a need for more powerful in-situ methods to monitor Langmuir monolayer formation, structure, and dynamics directly at the air-water interface in real-time [156, 256]. Techniques that can probe the structure and properties of the buried interfaces within multilayer LB films are also crucial. Correlating film structure at the nanoscale with macroscopic properties and device performance requires a multi-technique approach, combining microscopy (AFM, SEM, TEM, Brewster angle microscopy), spectroscopy (FTIR, Raman, XPS, synchrotron X-ray scattering) [194, 11], and functional measurements. Integrating experimental work with advanced computational modeling and simulations (e.g., molecular dynamics) can provide deeper insights into molecular interactions, self-assembly processes at the interface [257], and structure-property relationships, guiding experimental design (Oliveira et al., 2022).

Developing standardized protocols and characterization metrics would also aid in comparing results across different laboratories and improving reproducibility.

Exploring novel application domains and pushing the boundaries of existing ones is key to the future vitality of the field. This includes developing highly sophisticated sensors (e.g., electronic noses/tongues) [258], next-generation energy storage devices (particularly solid-state batteries where interfaces are critical (Fang et al., 2022)) [18, 259], advanced biomimetic systems for regenerative medicine or fundamental biological studies (Oliveira et al., 2022) [260, 261], and functional components for molecular or organic electronics [262]. The unique capabilities of LB in controlling molecular orientation and supramolecular arrangement should be further exploited for applications in nonlinear optics [125], chiral recognition [263, 264], and catalysis. The integration of LB with artificial intelligence and machine learning could potentially accelerate materials discovery and process optimization.

Finally, fostering interdisciplinary collaboration is paramount. Advancing Langmuir and LB technology requires synergistic efforts from chemists, physicists, materials scientists, biologists, and engineers. Bridging fundamental interfacial science with applied device engineering will be essential for translating laboratory discoveries into practical technologies. Increased international collaboration can also pool expertise and resources, accelerating progress in this multifaceted field [66]. The rich history of Langmuir monolayers and LB films provides a strong foundation [265], but continued innovation, driven by addressing challenges and embracing new opportunities through interdisciplinary research, will shape its future trajectory (Oliveira et al., 2022; Gu et al., 2024).

7 Conclusion

This survey has reviewed the history, fundamental principles, diverse material scope, characterization methodologies, burgeoning applications, and persistent challenges associated with Langmuir and Langmuir-Blodgett (LB) film technology. Originating from foundational studies on surface tension and molecular behavior at interfaces over a century ago, the LB technique has evolved into a sophisticated method for fabricating ultrathin films with unparalleled control over molecular organization and film thickness, often down to the single-molecule level. We have explored the underlying physic-

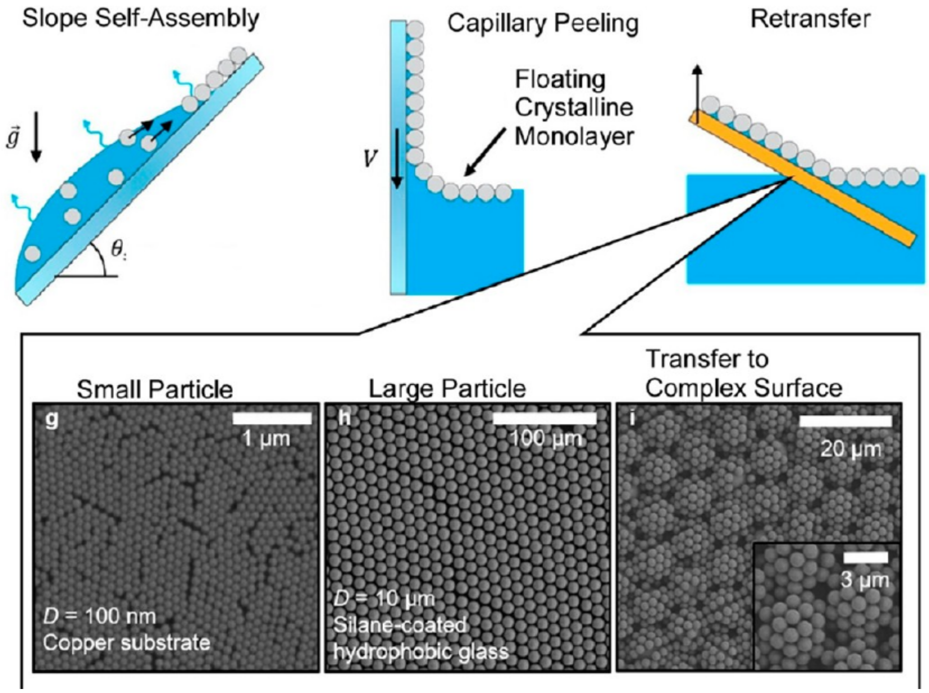

Figure 16: Illustration of two-dimensional colloidal nanoarchitectonics involving slope self-assembly, capillary peeling, and subsequent retransfer onto an arbitrary final substrate. This method allows immobilization of 2D crystals of colloidal spheres (100 nm to 10 μm diameter) and conformal coating. Adapted from Ariga, K., 2023 [266].

ochemical principles governing the formation of Langmuir monolayers at the air-water interface, characterized by surface pressure-area isotherms, and the subsequent transfer processes, primarily vertical dipping (Langmuir-Blodgett) and horizontal touching (Langmuir-Schaefer), onto solid substrates to create LB films. The ability to construct multilayer assemblies with precisely defined architectures, layer-by-layer, remains a hallmark of this technology.

The versatility of the LB technique is evident in the vast array of materials that can be structured into thin films [9, 2]. While classical amphiphiles like fatty acids and lipids were the initial focus, providing crucial insights into molecular packing and phase behavior [66, 70], the field has expanded dramatically to encompass polymers [7], biomacromolecules (pro-

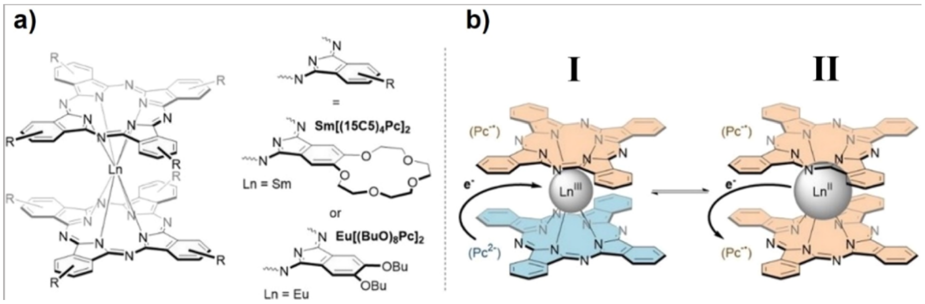

Figure 17: (a) Chemical structure of studied phthalocyanine compounds. (b) Proposed pathway of redox isomerization showing two distinct redox isomeric states (I and II) accessible for the europium complex. Adapted from Arakcheev, A., 2025 [267].

teins, peptides, DNA) [4, 253, 268], and various nanomaterials (nanoparticles, carbon allotropes like fullerenes, nanotubes, graphene, and graphene oxide [243, 92, 237], MOFs, COFs) [100, 269], alongside other functional molecules and nanostructures [89]. This material diversity allows for the tailoring of film properties for specific functions, leveraging the unique characteristics of each component within the highly ordered LB structure. We have detailed the essential characterization techniques, ranging from macroscopic measurements like surface manometry and surface potential to microscopic and spectroscopic methods such as Brewster angle microscopy, atomic force microscopy [111, 270, 49], scanning electron microscopy, infrared and Raman spectroscopy [11], and X-ray/neutron scattering [271]. These methods provide critical information about film morphology, molecular orientation, chemical composition, and structural integrity. Computational methods, particularly molecular dynamics simulations, increasingly complement experimental studies, offering deeper insights into molecular interactions and film formation mechanisms.

The applications of LB films span numerous scientific and technological domains, underscoring their significance [2, 9]. In sensing, the high surface area, ordered structure, and tunable surface chemistry of LB films enable the development of highly sensitive and selective gas sensors [98, 94], chemical sensors [14, 13], and biosensors, including electronic noses and tongues [272, 258]. The precise control over film architecture is particularly advantageous for optimizing interactions with target analytes and enhancing transduction signals [26]. In electrochemistry, LB films serve as model systems for studying

interfacial electron transfer, modifying electrode surfaces to enhance catalytic activity, and fabricating components for energy storage devices like batteries, where they can function as active electrode materials, protective coatings, or artificial solid-electrolyte interphases [273]. The ability to create biomimetic membranes using LB films, often incorporating lipids, proteins, and other biologically relevant molecules, provides invaluable platforms for studying membrane structure-function relationships, drug-membrane interactions, and developing biocompatible surfaces for medical implants or tissue engineering scaffolds [74, 237]. Furthermore, LB films have been instrumental in proof-of-concept studies for molecular electronics [16, 262].

Despite these successes, the transition of LB technology from laboratory research to widespread industrial application faces hurdles, primarily related to the mechanical robustness of some films (especially those made from small amphiphiles), the relatively slow deposition speed, and the complexities associated with scaling up the process for large-area, high-throughput manufacturing. Competing techniques like self-assembled monolayers (SAMs) and layer-by-layer (LbL) assembly offer advantages in terms of simplicity, robustness, and scalability for certain applications, leading to shifts in research focus over the past decades; however, the unparalleled precision in molecular arrangement offered by LB ensures its continued relevance for applications demanding high structural order.

Looking ahead, the future of Langmuir and Langmuir-Blodgett films appears promising, driven by innovation and interdisciplinary collaboration. The concept of "nanoarchitectonics," which emphasizes the rational design and construction of functional materials from nanoscale components, finds a powerful tool in the LB technique. Continued research into novel materials, including advanced 2D materials and hybrid systems, combined with refined deposition strategies, aims specifically to overcome limitations in film stability and functionality, thereby addressing the robustness challenge. Exploiting phenomena at the air-water interface itself, beyond simple film transfer, offers unique opportunities for studying molecular recognition, controlling chemical reactions in 2D confinement, and assembling complex supramolecular structures. The synergy between LB methods and other fabrication techniques, as well as advancements in characterization tools capable of probing interfaces with higher resolution, will further expand the possibilities. Addressing the challenges of scalability and deposition speed through innovative engineering solutions and material design remains crucial for unlocking broader practical applications. In essence, Langmuir and Langmuir-Blodgett technology, while

mature in its foundations, continues to be a vibrant field of research, offering indispensable tools for fundamental science and holding significant potential for future technological breakthroughs, particularly in areas demanding exquisite control at the molecular level. Its enduring legacy lies not only in the specific applications it enables but also in its foundational contribution to our understanding and manipulation of matter at the nanoscale.

References

[1] Katsuhiko Ariga and Jonathan P. Hill. "Monolayers at air-water interfaces: from origins-of-life to nanotechnology". In: *The Chemical Record* 11.4 (2011), 199–211. ISSN: 1528-0691. DOI: 10.1002/tcr.201100004. URL: http://dx.doi.org/10.1002/tcr.201100004.

[2] Wenhui Gu et al. "Recent Progress in the Applications of Langmuir–Blodgett Film Technology". In: *Nanomaterials* 14.12 (2024), p. 1039. ISSN: 2079-4991. DOI: 10.3390/nano14121039. URL: http://dx.doi.org/10.3390/nano14121039.

[3] Katsuhiko Ariga et al. "Layer-by-layer Nanoarchitectonics: Invention, Innovation, and Evolution". In: *Chemistry Letters* 43.1 (2013), 36–68. ISSN: 1348-0715. DOI: 10.1246/cl.130987. URL: http://dx.doi.org/10.1246/cl.130987.

[4] Mitsuru Akashi and Takami Akagi. "Composite Materials by Building Block Chemistry Using Weak Interaction". In: *Bulletin of the Chemical Society of Japan* 94.7 (2021), 1903–1921. ISSN: 1348-0634. DOI: 10.1246/bcsj.20210089. URL: http://dx.doi.org/10.1246/bcsj.20210089.

[5] Gero Decher and Jong-Dal Hong. "Buildup of ultrathin multilayer films by a self-assembly process, 1 consecutive adsorption of anionic and cationic bipolar amphiphiles on charged surfaces". In: *Makromolekulare Chemie. Macromolecular Symposia* 46.1 (1991), 321–327. ISSN: 0258-0322. DOI: 10.1002/masy.19910460145. URL: http://dx.doi.org/10.1002/masy.19910460145.

[6] Katsuhiko Ariga. “Don't Forget Langmuir–Blodgett Films 2020: Interfacial Nanoarchitectonics with Molecules, Materials, and Living Objects”. In: *Langmuir* 36.26 (2020), 7158–7180. ISSN: 1520-5827. DOI: 10.1021/acs.langmuir.0c01044. URL: http://dx.doi.org/10.1021/acs.langmuir.0c01044.

[7] Gero Decher. “Fuzzy Nanoassemblies: Toward Layered Polymeric Multicomposites”. In: *Science* 277.5330 (1997), 1232–1237. ISSN: 1095-9203. DOI: 10.1126/science.277.5330.1232. URL: http://dx.doi.org/10.1126/science.277.5330.1232.

[8] Michael C. Petty. *Langmuir-Blodgett Films: An Introduction.* Cambridge University Press, 1996. ISBN: 9780511622519. DOI: 10.1017/cbo9780511622519. URL: http://dx.doi.org/10.1017/cbo9780511622519.

[9] Katsuhiko Ariga et al. “25th Anniversary Article: What Can Be Done with the Langmuir-Blodgett Method? Recent Developments and its Critical Role in Materials Science”. In: *Advanced Materials* 25.45 (2013), 6477–6512. ISSN: 1521-4095. DOI: 10.1002/adma.201302283. URL: http://dx.doi.org/10.1002/adma.201302283.

[10] Jin Young Park and Rigoberto C. Advincula. “Nanostructuring polymers, colloids, and nanomaterials at the air–water interface through Langmuir and Langmuir–Blodgett techniques”. In: *Soft Matter* 7.21 (2011), p. 9829. ISSN: 1744-6848. DOI: 10.1039/c1sm05750b. URL: http://dx.doi.org/10.1039/c1sm05750b.

[11] Junzo. Umemura et al. “Quantitative evaluation of molecular orientation in thin Langmuir-Blodgett films by FT-IR transmission and reflection-absorption spectroscopy”. In: *The Journal of Physical Chemistry* 94.1 (1990), 62–67. ISSN: 1541-5740. DOI: 10.1021/j100364a009. URL: http://dx.doi.org/10.1021/j100364a009.

[12] Alvina V. Alexandrova et al. “Structure affinity of the Langmuir monolayer and the corresponding Langmuir–Blodgett film revealed by X-ray techniques”. In: *Soft Matter* 20.43 (2024), 8601–8609. ISSN: 1744-6848. DOI: 10.1039/d4sm01050g. URL: http://dx.doi.org/10.1039/d4sm01050g.

[13] Gabriele Giancane and Ludovico Valli. “State of art in porphyrin Langmuir–Blodgett films as chemical sensors”. In: *Advances in Colloid and Interface Science* 171–172 (2012), 17–35. ISSN: 0001-8686.

DOI: 10.1016/j.cis.2012.01.001. URL: http://dx.doi.org/10.1016/j.cis.2012.01.001.

[14] Ludovico Valli. "Phthalocyanine-based Langmuir–Blodgett films as chemical sensors". In: *Advances in Colloid and Interface Science* 116.1–3 (2005), 13–44. ISSN: 0001-8686. DOI: 10.1016/j.cis.2005.04.008. URL: http://dx.doi.org/10.1016/j.cis.2005.04.008.

[15] Masuo AIZAWA. "LB Film Technology for Biosensors". In: *Journal of Japan Oil Chemists' Society* 39.3 (1990), 166–170. ISSN: 1884-2003. DOI: 10.5650/jos1956.39.3_166. URL: http://dx.doi.org/10.5650/jos1956.39.3_166.

[16] Soraya Sangiao et al. "All-Carbon Electrode Molecular Electronic Devices Based on Langmuir–Blodgett Monolayers". In: *Small* 13.7 (2016). ISSN: 1613-6829. DOI: 10.1002/smll.201603207. URL: http://dx.doi.org/10.1002/smll.201603207.

[17] Muthukumar Divagar, Nagamony Ponpandian, and Chinnuswamy Viswanathan. "Langmuir-Blodgett deposited Na3V2(PO4)3-MnO2 nanocomposite thin film electrodes for hybrid energy storage application". In: *Materials Science and Engineering: B* 270 (2021), p. 115229. ISSN: 0921-5107. DOI: 10.1016/j.mseb.2021.115229. URL: http://dx.doi.org/10.1016/j.mseb.2021.115229.

[18] Chen Fang et al. "Recent Applications of Langmuir–Blodgett Technique in Battery Research". In: *ACS Applied Materials amp; Interfaces* 14.2 (2022), 2431–2439. ISSN: 1944-8252. DOI: 10.1021/acsami.1c19064. URL: http://dx.doi.org/10.1021/acsami.1c19064.

[19] R H Tredgold. "The physics of Langmuir-Blodgett films". In: *Reports on Progress in Physics* 50.12 (1987), 1609–1656. ISSN: 1361-6633. DOI: 10.1088/0034-4885/50/12/002. URL: http://dx.doi.org/10.1088/0034-4885/50/12/002.

[20] Daniel K. Schwartz. "Langmuir-Blodgett film structure". In: *Surface Science Reports* 27.7–8 (1997), 245–334. ISSN: 0167-5729. DOI: 10.1016/s0167-5729(97)00003-4. URL: http://dx.doi.org/10.1016/s0167-5729(97)00003-4.

[21] Rajkumar Sunil Singh. “Langmuir and Langmuir–Blodgett films of aromatic amphiphiles”. In: *Soft Materials* 20.1 (2021), 57–98. ISSN: 1539-4468. DOI: 10.1080/1539445x.2021.1922443. URL: http://dx.doi.org/10.1080/1539445x.2021.1922443.

[22] P.J.W. Stone et al. “Polysiloxanes for Langmuir-Blodgett applications”. In: *Thin Solid Films* 244.1–2 (1994), 1012–1017. ISSN: 0040-6090. DOI: 10.1016/0040-6090(94)90621-1. URL: http://dx.doi.org/10.1016/0040-6090(94)90621-1.

[23] Syed Hussain, Sekhar Chakraborty, and Debajyoti Bhattacharjee. “Nano Dimensional Hybrid Organo-clay Langmuir-Blodgett Films”. In: *Current Physical Chemistry* 3.3 (2013), 322–332. ISSN: 1877-9468. DOI: 10.2174/1877946811303030008. URL: http://dx.doi.org/10.2174/1877946811303030008.

[24] J.K. Basu and M.K. Sanyal. “Ordering and growth of Langmuir–Blodgett films: X-ray scattering studies”. In: *Physics Reports* 363.1 (2002), 1–84. ISSN: 0370-1573. DOI: 10.1016/s0370-1573(01)00083-7. URL: http://dx.doi.org/10.1016/s0370-1573(01)00083-7.

[25] Epameinondas Leontidis. *Langmuir–Blodgett Films: Sensor and Biomedical Applications and Comparisons with the Layer-by-Layer Method.* 2016. DOI: 10.1002/9783527698813.ch5. URL: http://dx.doi.org/10.1002/9783527698813.ch5.

[26] Heng Lin et al. “Controllable-assembled functional monolayers by the Langmuir–Blodgett technique for optoelectronic applications”. In: *Journal of Materials Chemistry C* 12.4 (2024), 1177–1210. ISSN: 2050-7534. DOI: 10.1039/d3tc03591c. URL: http://dx.doi.org/10.1039/d3tc03591c.

[27] Zahra Alinia et al. “Amphiphilicity of Tetraazaporphyrins Containing Four Terminal Carboxylic Acid and Four Alkyl Groups Promotes Face-On Orientation in Langmuir Films”. In: *Langmuir* 40.50 (2024). PMID: 39623767, pp. 26672–26684. DOI: 10.1021/acs.langmuir.4c03800. eprint: https://doi.org/10.1021/acs.langmuir.4c03800. URL: https://doi.org/10.1021/acs.langmuir.4c03800.

[28] G. Decher, J.D. Hong, and J. Schmitt. “Buildup of ultrathin multilayer films by a self-assembly process: III. Consecutively alternating adsorption of anionic and cationic polyelectrolytes on charged surfaces”. In: *Thin Solid Films* 210–211 (1992), 831–835. ISSN: 0040-6090.

DOI: 10.1016/0040-6090(92)90417-a. URL: http://dx.doi.org/10.1016/0040-6090(92)90417-a.

[29] M Sakurai et al. "Molecular dynamics simulation of water between hydrophobic surfaces. Implication for the long-range hydrophobic force". In: *Chemical Physics Letters* 289.5–6 (1998), 567–571. ISSN: 0009-2614. DOI: 10.1016/s0009-2614(98)00462-x. URL: http://dx.doi.org/10.1016/s0009-2614(98)00462-x.

[30] Wei-Tao Liu, Luning Zhang, and Y. R. Shen. "Interfacial structures of methanol:water mixtures at a hydrophobic interface probed by sum-frequency vibrational spectroscopy". In: *The Journal of Chemical Physics* 125.14 (2006). ISSN: 1089-7690. DOI: 10.1063/1.2354088. URL: http://dx.doi.org/10.1063/1.2354088.

[31] G. Brezesinski et al. "Morphology and structures in double-, triple- and quadruple-chain phospholipid monolayers at the air/water interface". In: *Trends in Colloid and Interface Science IX*. Steinkopff, 255–262. ISBN: 9783798510319. DOI: 10.1007/bfb0115249. URL: http://dx.doi.org/10.1007/bfb0115249.

[32] H M McConnell. "Structures and Transitions in Lipid Monolayers at the Air-Water Interface". In: *Annual Review of Physical Chemistry* 42.1 (1991), 171–195. ISSN: 1545-1593. DOI: 10.1146/annurev.pc.42.100191.001131. URL: http://dx.doi.org/10.1146/annurev.pc.42.100191.001131.

[33] Junya Adachi et al. "Coordination Amphiphile: Design of Planar-Coordinated Platinum Complexes for Monolayer Formation at an Air-Water Interface Based on Ligand Characteristics and Molecular Topology". In: *Bulletin of the Chemical Society of Japan* 95.6 (2022), 889–897. ISSN: 1348-0634. DOI: 10.1246/bcsj.20220086. URL: http://dx.doi.org/10.1246/bcsj.20220086.

[34] Hiroshi Koyano et al. "Effect of Melamine-Amphiphile Structure on the Extent of Two-Dimensional Hydrogen-Bonded Networks Incorporating Barbituric Acid". In: *Chemistry – A European Journal* 3.7 (1997), 1077–1082. ISSN: 1521-3765. DOI: 10.1002/chem.19970030715. URL: http://dx.doi.org/10.1002/chem.19970030715.

[35] Katsuhiko Ariga et al. "Materials nanoarchitectonics at two-dimensional liquid interfaces". In: *Beilstein Journal of Nanotechnology* 10 (2019), 1559–1587. ISSN: 2190-4286. DOI: 10.3762/bjnano.10.153. URL: http://dx.doi.org/10.3762/bjnano.10.153.

[36] Mohamed Ahmida et al. "Face- and edge-on orientations of octa-acid and -alcohol substituted tetraazaporphyrins in Langmuir and Langmuir–Blodgett monolayers". In: *Soft Matter* 9.3 (2013), 811–819. ISSN: 1744-6848. DOI: 10.1039/c2sm27064a. URL: http://dx.doi.org/10.1039/c2sm27064a.

[37] Shigeru Negi et al. "Highly Ordered Monolayers of an Optically Active Amphiphilic Pyrene Derivative at the Air–Water Interface". In: *Bulletin of the Chemical Society of Japan* 95.11 (2022), 1537–1545. ISSN: 1348-0634. DOI: 10.1246/bcsj.20220233. URL: http://dx.doi.org/10.1246/bcsj.20220233.

[38] Shigeru Negi et al. "Monolayer Formation and Chiral Recognition of Binaphthyl Amphiphiles at the Air–Water Interface". In: *Bulletin of the Chemical Society of Japan* 96.1 (2022), 48–56. ISSN: 1348-0634. DOI: 10.1246/bcsj.20220286. URL: http://dx.doi.org/10.1246/bcsj.20220286.

[39] B.D. Summ, V.S. Yushchenko, and E.D. Shchukin. "Hydrodynamic and physicochemical aspects of spreading". In: *Colloids and Surfaces* 27.4 (1987), 43–55. ISSN: 0166-6622. DOI: 10.1016/0166-6622(87)80132-4. URL: http://dx.doi.org/10.1016/0166-6622(87)80132-4.

[40] Matthew J. Large et al. "Understanding Solvent Spreading for Langmuir Deposition of Nanomaterial Films: A Hansen Solubility Parameter Approach". In: *Langmuir* 33.51 (2017), 14766–14771. ISSN: 1520-5827. DOI: 10.1021/acs.langmuir.7b03867. URL: http://dx.doi.org/10.1021/acs.langmuir.7b03867.

[41] William D. Harkins, T. Fraser Young, and Edward Boyd. "The Thermodynamics of Films: Energy and Entropy of Extension and Spreading of Insoluble Monolayers". In: *The Journal of Chemical Physics* 8.12 (1940), 954–965. ISSN: 1089-7690. DOI: 10.1063/1.1750610. URL: http://dx.doi.org/10.1063/1.1750610.

[42] Siji Sudheesh and Jamil Ahmad. “Effect of Wilhelmy Plate Material on Hysteresis of Langmuir Film Isotherms”. In: *Asian Journal of Chemistry* 25.7 (2013), 3535–3538. ISSN: 0975-427X. DOI: 10.14233/ajchem.2013.13213. URL: http://dx.doi.org/10.14233/ajchem.2013.13213.

[43] D. G. Dervichian. “Changes of Phase and Transformations of Higher Order in Monolayers”. In: *The Journal of Chemical Physics* 7.10 (1939), 931–948. ISSN: 1089-7690. DOI: 10.1063/1.1750347. URL: http://dx.doi.org/10.1063/1.1750347.

[44] C. Naselli, J. F. Rabolt, and J. D. Swalen. “Order–disorder transitions in Langmuir–Blodgett monolayers. I. Studies of two-dimensional melting by infrared spectroscopy”. In: *The Journal of Chemical Physics* 82.4 (1985), 2136–2140. ISSN: 1089-7690. DOI: 10.1063/1.448351. URL: http://dx.doi.org/10.1063/1.448351.

[45] Ka Yee C. Lee. “Collapse Mechanisms of Langmuir Monolayers”. In: *Annual Review of Physical Chemistry* 59.1 (2008), 771–791. ISSN: 1545-1593. DOI: 10.1146/annurev.physchem.58.032806.104619. URL: http://dx.doi.org/10.1146/annurev.physchem.58.032806.104619.

[46] Christophe Ybert et al. “Collapse of a Monolayer by Three Mechanisms”. In: *The Journal of Physical Chemistry B* 106.8 (2002), 2004–2008. ISSN: 1520-5207. DOI: 10.1021/jp013173z. URL: http://dx.doi.org/10.1021/jp013173z.

[47] S. Hénon and J. Meunier. “Microscope at the Brewster angle: Direct observation of first-order phase transitions in monolayers”. In: *Review of Scientific Instruments* 62.4 (1991), 936–939. ISSN: 1089-7623. DOI: 10.1063/1.1142032. URL: http://dx.doi.org/10.1063/1.1142032.

[48] Lucile Gambut et al. “Ellipsometry, Brewster Angle Microscopy, and Thermodynamic Studies of Monomolecular Films of Cryptophanes at the AirWater Interface”. In: *Langmuir* 12.22 (1996), 5407–5412. ISSN: 1520-5827. DOI: 10.1021/la960245i. URL: http://dx.doi.org/10.1021/la960245i.

[49] Th. Geue et al. “Investigations of pH-dependent domain structure of lead arachidate Langmuir-Blodgett films by means of x-ray specular and diffuse scattering and atomic force microscopy”. In: *The Journal of Chemical Physics* 110.16 (1999), 8104–8111. ISSN: 1089-7690. DOI: 10.1063/1.478713. URL: http://dx.doi.org/10.1063/1.478713.

[50] Gary Marshall, Michael Dennin, and Charles M. Knobler. “A compact Brewster-angle microscope for use in Langmuir–Blodgett deposition”. In: *Review of Scientific Instruments* 69.10 (1998), 3699–3700. ISSN: 1089-7623. DOI: 10.1063/1.1149162. URL: http://dx.doi.org/10.1063/1.1149162.

[51] M M Qassime et al. “A studying of subphase temperature and dissolved ascorbic acid concentration influence on the process of Langmuir monolayer formation”. In: *Journal of Physics: Conference Series* 1124 (2018), p. 031010. ISSN: 1742-6596. DOI: 10.1088/1742-6596/1124/3/031010. URL: http://dx.doi.org/10.1088/1742-6596/1124/3/031010.

[52] Gennady B. Khomutov et al. “Langmuir monolayers and Langmuir-Blodgett films of pH-sensitive lipid”. In: *Colloids and Surfaces A: Physicochemical and Engineering Aspects* 532 (2017), 150–154. ISSN: 0927-7757. DOI: 10.1016/j.colsurfa.2017.05.070. URL: http://dx.doi.org/10.1016/j.colsurfa.2017.05.070.

[53] Larbi Eddaif, Abdul Shaban, and István Szendro. “Calix[4]Resorcinarene Macrocycles Interactions with Cd2+, Hg2+, Pb2+, and Cu2+ Cations: A QCM-I and Langmuir Ultra-thin Monolayers Study”. In: *Electroanalysis* 32.4 (2019), 755–766. ISSN: 1521-4109. DOI: 10.1002/elan.201900651. URL: http://dx.doi.org/10.1002/elan.201900651.

[54] Ayumi Kamino et al. “Control of the Molecular Packing in Guanidinium Monolayers through Binding with Aqueous Polycarboxylates”. In: *Bulletin of the Chemical Society of Japan* 69.12 (1996), 3619–3631. ISSN: 1348-0634. DOI: 10.1246/bcsj.69.3619. URL: http://dx.doi.org/10.1246/bcsj.69.3619.

[55] Naveen Kumar et al. “Salt Dependent Stability of Stearic Acid Langmuir–Blodgett Films Exposed to Aqueous Electrolytes”. In: *Langmuir* 29.17 (2013), 5150–5159. ISSN: 1520-5827. DOI: 10.1021/la400615j. URL: http://dx.doi.org/10.1021/la400615j.

[56] Siji Sudheesh, Jamil Ahmad, and Girija S. Singh. "Hysteresis of Isotherms of Mixed Monolayers of N-Octadecyl-N-phenylthiourea and Stearic Acid at Air/Water Interface". In: *ISRN Physical Chemistry* 2012 (2012), 1–6. ISSN: 2090-7761. DOI: 10.5402/2012/835397. URL: http://dx.doi.org/10.5402/2012/835397.

[57] Alfred Yeboah and Matthew F. Paige. "Effect of perfluorotetradecanoic acid on the morphology of a photopolymerizable phospholipid monolayer at the air-water interface". In: *Journal of Surfactants and Detergents* 26.6 (2023), 779–787. ISSN: 1558-9293. DOI: 10.1002/jsde.12700. URL: http://dx.doi.org/10.1002/jsde.12700.

[58] M M Qassime et al. "Studies the interactions of ascorbic acid isoforms with a simple model of DPPC monolayer as a biomimetic membrane by Langmuir-Blodgett technique". In: *Journal of Physics: Conference Series* 1853.1 (2021), p. 012035. ISSN: 1742-6596. DOI: 10.1088/1742-6596/1853/1/012035. URL: http://dx.doi.org/10.1088/1742-6596/1853/1/012035.

[59] S. Garoff et al. "The effects of substrate roughness on ultrathin water films". In: *The Journal of Chemical Physics* 90.12 (1989), 7505–7515. ISSN: 1089-7690. DOI: 10.1063/1.456184. URL: http://dx.doi.org/10.1063/1.456184.

[60] Simona Bettarini et al. "Characterization of the monolayer as the basis of Langmuir-Blodgett film formation: metal derivatives of behenic acid". In: *Langmuir* 7.6 (1991), 1082–1087. ISSN: 1520-5827. DOI: 10.1021/la00054a010. URL: http://dx.doi.org/10.1021/la00054a010.

[61] Katharine B. Blodgett. "Films Built by Depositing Successive Monomolecular Layers on a Solid Surface". In: *Journal of the American Chemical Society* 57.6 (1935), 1007–1022. ISSN: 1520-5126. DOI: 10.1021/ja01309a011. URL: http://dx.doi.org/10.1021/ja01309a011.

[62] Geoffrey J. Ashwell, Paul D. Jackson, and Wendy A. Crossland. "Noncentrosymmetry and second-harmonic generation in Z-type Langmuir–Blodgett films". In: *Nature* 368.6470 (1994), 438–440. ISSN: 1476-4687. DOI: 10.1038/368438a0. URL: http://dx.doi.org/10.1038/368438a0.

[63] Masato Ito et al. “Hyper 100 °C Langmuir–Blodgett (Langmuir–Schaefer) Technique for Organized Ultrathin Film of Polymeric Semiconductors”. In: *Langmuir* 38.17 (2021), 5237–5247. ISSN: 1520-5827. DOI: 10.1021/acs.langmuir.1c02596. URL: http://dx.doi.org/10.1021/acs.langmuir.1c02596.

[64] Issei Kitamura et al. “Photo-triggered large mass transport driven only by a photoresponsive surface skin layer”. In: *Scientific Reports* 10.1 (2020). ISSN: 2045-2322. DOI: 10.1038/s41598-020-69605-8. URL: http://dx.doi.org/10.1038/s41598-020-69605-8.

[65] V. Marchi-Artzner et al. “Molecular Recognition between 2,4,6-Triaminopyrimidine Lipid Monolayers and Complementary Barbituric Molecules at the Air/Water Interface: Effects of Hydrophilic Spacer, Ionic Strength, and pH”. In: *Langmuir* 14.18 (1998), 5164–5171. ISSN: 1520-5827. DOI: 10.1021/la971192n. URL: http://dx.doi.org/10.1021/la971192n.

[66] Thi Thao Vu et al. “EVALUATION OF RESEARCH PROGRESS, TRENDS, AND APPLICATIONS OF LANGMUIR-BLODGETT FILMS OF FATTY ACIDS”. In: *ChemChemTech* 68.2 (2024), 6–45. ISSN: 0579-2991. DOI: 10.6060/ivkkt.20256802.7002. URL: http://dx.doi.org/10.6060/ivkkt.20256802.7002.

[67] Takamasa Ishikawa et al. “Maze Pattern at Nanometer-Scale in a Mixed Langmuir Monolayer of Fatty Acids”. In: *Bulletin of the Chemical Society of Japan* 94.12 (2021), 2967–2969. ISSN: 1348-0634. DOI: 10.1246/bcsj.20210335. URL: http://dx.doi.org/10.1246/bcsj.20210335.

[68] Monika Rojewska et al. “Langmuir Monolayer Techniques for the Investigation of Model Bacterial Membranes and Antibiotic Biodegradation Mechanisms”. In: *Membranes* 11.9 (2021), p. 707. ISSN: 2077-0375. DOI: 10.3390/membranes11090707. URL: http://dx.doi.org/10.3390/membranes11090707.

[69] Andreas Santamaria et al. “Investigation on the relationship between lipid composition and structure in model membranes composed of extracted natural phospholipids”. In: (2022). DOI: 10.26434/chemrxiv-2022-mrfmx. URL: http://dx.doi.org/10.26434/chemrxiv-2022-mrfmx.

[70] Xueying Guo and Wuge H. Briscoe. "Molecular interactions, elastic properties, and nanostructure of Langmuir bacterial-lipid monolayers: Towards solving the mystery in bacterial membrane asymmetry". In: *Current Opinion in Colloid amp; Interface Science* 67 (2023), p. 101731. ISSN: 1359-0294. DOI: 10.1016/j.cocis.2023.101731. URL: http://dx.doi.org/10.1016/j.cocis.2023.101731.

[71] Naoki Takeshita, Masanari Okuno, and Taka-aki Ishibashi. "Molecular conformation of DPPC phospholipid Langmuir and Langmuir–Blodgett monolayers studied by heterodyne-detected vibrational sum frequency generation spectroscopy". In: *Physical Chemistry Chemical Physics* 19.3 (2017), 2060–2066. ISSN: 1463-9084. DOI: 10.1039/c6cp07800a. URL: http://dx.doi.org/10.1039/c6cp07800a.

[72] Victoria M. Katata et al. "The Role of Cholesterol in the Interaction of the Lipid Monolayer with the Endocrine Disruptor Bisphenol-A". In: *Membranes* 12.8 (2022), p. 729. ISSN: 2077-0375. DOI: 10.3390/membranes12080729. URL: http://dx.doi.org/10.3390/membranes12080729.

[73] Eduardo Guzmán et al. "Mixed DPPC–cholesterol Langmuir monolayers in presence of hydrophilic silica nanoparticles". In: *Colloids and Surfaces B: Biointerfaces* 105 (2013), 284–293. ISSN: 0927-7765. DOI: 10.1016/j.colsurfb.2013.01.020. URL: http://dx.doi.org/10.1016/j.colsurfb.2013.01.020.

[74] Klaudia Szafran et al. "Surface Properties of the Polyethylene Terephthalate (PET) Substrate Modified with the Phospholipid-Polypeptide-Antioxidant Films: Design of Functional Biocoatings". In: *Pharmaceutics* 14.12 (2022), p. 2815. ISSN: 1999-4923. DOI: 10.3390/pharmaceutics14122815. URL: http://dx.doi.org/10.3390/pharmaceutics14122815.

[75] Gerald Brezesinski and Helmuth Möhwald. "Langmuir monolayers to study interactions at model membrane surfaces". In: *Advances in Colloid and Interface Science* 100–102 (2003), 563–584. ISSN: 0001-8686. DOI: 10.1016/s0001-8686(02)00071-4. URL: http://dx.doi.org/10.1016/s0001-8686(02)00071-4.

[76] J. Miñones et al. "Interactions between membrane sterols and phospholipids in model mammalian and fungi cellular membranes — A Langmuir monolayer study". In: *Biophysical Chemistry* 140.1–3 (2009),

69–77. ISSN: 0301-4622. DOI: 10.1016/j.bpc.2008.11.011. URL: http://dx.doi.org/10.1016/j.bpc.2008.11.011.

[77] Richard A. Campbell et al. "Structure of surfactant and phospholipid monolayers at the air/water interface modeled from neutron reflectivity data". In: *Journal of Colloid and Interface Science* 531 (2018), 98–108. ISSN: 0021-9797. DOI: 10.1016/j.jcis.2018.07.022. URL: http://dx.doi.org/10.1016/j.jcis.2018.07.022.

[78] Dawn Y. Takamoto et al. "Stable Ordering in Langmuir-Blodgett Films". In: *Science* 293.5533 (2001), 1292–1295. ISSN: 1095-9203. DOI: 10.1126/science.1060018. URL: http://dx.doi.org/10.1126/science.1060018.

[79] Fabio A. Scholl et al. "Carbon Nanotubes Arranged As Smart Interfaces in Lipid Langmuir–Blodgett Films Enhancing the Enzymatic Properties of Penicillinase for Biosensing Applications". In: *ACS Applied Materials amp; Interfaces* 9.36 (2017), 31054–31066. ISSN: 1944-8252. DOI: 10.1021/acsami.7b08095. URL: http://dx.doi.org/10.1021/acsami.7b08095.

[80] Felipe Tejada Araujo, Laura Oliveira Peres, and Luciano Caseli. "Conjugated Polymers Blended with Lipids and Galactosidase as Langmuir–Blodgett Films To Control the Biosensing Properties of Nanostructured Surfaces". In: *Langmuir* 35.22 (2019), 7294–7303. ISSN: 1520-5827. DOI: 10.1021/acs.langmuir.9b00536. URL: http://dx.doi.org/10.1021/acs.langmuir.9b00536.

[81] Go Watanabe et al. "Biomolecular Binding at Aqueous Interfaces of Langmuir Monolayers of Bioconjugated Amphiphilic Mesogenic Molecules: A Molecular Dynamics Study". In: *Langmuir* 36.41 (2020), 12281–12287. ISSN: 1520-5827. DOI: 10.1021/acs.langmuir.0c02191. URL: http://dx.doi.org/10.1021/acs.langmuir.0c02191.

[82] T.S. Berzina et al. "Langmuir-Blodgett films composed of monolayers of amphiphilic molecules and adsorbed soluble proteins". In: *Thin Solid Films* 284–285 (1996), 757–761. ISSN: 0040-6090. DOI: 10.1016/s0040-6090(95)08439-8. URL: http://dx.doi.org/10.1016/s0040-6090(95)08439-8.

[83] Katarzyna Pastuszak et al. "Influence of the Antimicrobial LL-37 Peptide on Legionella dumoffii Phospholipids Adsorbed at the Air–Liquid Interface". In: *Sustainability* 15.8 (2023), p. 6670. ISSN: 2071-1050. DOI: 10.3390/su15086670. URL: http://dx.doi.org/10.3390/su15086670.

[84] J. H. Cheung, R. B. Rosner, and M. F. Rubner. "New Strategies for Preparing Electrically Conductive Langmuir-Blodgett Films". In: *MRS Proceedings* 247 (1992). ISSN: 1946-4274. DOI: 10.1557/proc-247-859. URL: http://dx.doi.org/10.1557/proc-247-859.

[85] Huie Zhu, Masaya Mitsuishi, and Tokuji Miyashita. "Facile Preparation of Highly Oriented Poly(vinylidene fluoride) Langmuir–Blodgett Nanofilms Assisted by Amphiphilic Polymer Nanosheets". In: *Macromolecules* 45.22 (2012), 9076–9084. ISSN: 1520-5835. DOI: 10.1021/ma301711g. URL: http://dx.doi.org/10.1021/ma301711g.

[86] T. Aida, E. W. Meijer, and S. I. Stupp. "Functional Supramolecular Polymers". In: *Science* 335.6070 (2012), 813–817. ISSN: 1095-9203. DOI: 10.1126/science.1205962. URL: http://dx.doi.org/10.1126/science.1205962.

[87] P.K. Hashim et al. "Supramolecular Polymerization: A Conceptual Expansion for Innovative Materials". In: *Progress in Polymer Science* 105 (2020), p. 101250. ISSN: 0079-6700. DOI: 10.1016/j.progpolymsci.2020.101250. URL: http://dx.doi.org/10.1016/j.progpolymsci.2020.101250.

[88] Jean-Marie Lehn. "Supramolecular Chemistry—Scope and Perspectives Molecules, Supermolecules, and Molecular Devices (Nobel Lecture)". In: *Angewandte Chemie International Edition in English* 27.1 (1988), 89–112. ISSN: 0570-0833. DOI: 10.1002/anie.198800891. URL: http://dx.doi.org/10.1002/anie.198800891.

[89] Katsuhiko Ariga et al. "Nanoarchitectonics for Dynamic Functional Materials from Atomic-/Molecular-Level Manipulation to Macroscopic Action". In: *Advanced Materials* 28.6 (2015), 1251–1286. ISSN: 1521-4095. DOI: 10.1002/adma.201502545. URL: http://dx.doi.org/10.1002/adma.201502545.

[90] Nobuhiko Hosono. “Design of Porous Coordination Materials with Dynamic Properties”. In: *Bulletin of the Chemical Society of Japan* 94.1 (2020), 60–69. ISSN: 1348-0634. DOI: 10.1246/bcsj.20200242. URL: http://dx.doi.org/10.1246/bcsj.20200242.

[91] Renhao Dong et al. “Large-Area, Free-Standing, Two-Dimensional Supramolecular Polymer Single-Layer Sheets for Highly Efficient Electrocatalytic Hydrogen Evolution”. In: *Angewandte Chemie International Edition* 54.41 (2015), 12058–12063. ISSN: 1521-3773. DOI: 10.1002/anie.201506048. URL: http://dx.doi.org/10.1002/anie.201506048.

[92] Nik J Walch et al. “Characterisation of thin films of graphene–surfactant composites produced through a novel semi-automated method”. In: *Beilstein Journal of Nanotechnology* 7 (2016), 209–219. ISSN: 2190-4286. DOI: 10.3762/bjnano.7.19. URL: http://dx.doi.org/10.3762/bjnano.7.19.

[93] Yan Nie et al. “Controlled fabrication of biocompatible graphene oxide Langmuir–Blodgett films by size and surface property manipulation”. In: *Journal of Dispersion Science and Technology* 43.12 (2021), 1747–1754. ISSN: 1532-2351. DOI: 10.1080/01932691.2021.1880430. URL: http://dx.doi.org/10.1080/01932691.2021.1880430.

[94] Na Li et al. “Chemical gas sensor, surface enhanced Raman scattering and photoelectrics of composite Langmuir-Blodgett films consisting of polypeptide and dye molecules”. In: *Colloids and Surfaces A: Physicochemical and Engineering Aspects* 663 (2023), p. 131067. ISSN: 0927-7757. DOI: 10.1016/j.colsurfa.2023.131067. URL: http://dx.doi.org/10.1016/j.colsurfa.2023.131067.

[95] Sriramprabha Ramasamy, Ponpandian Nagamony, and Viswanathan Chinnuswamy. “Self-assembled SnO2/reduced graphene oxide nanocomposites via Langmuir-Blodgett technique as anode materials for Li-ion batteries”. In: *Materials Letters* 218 (2018), 295–298. ISSN: 0167-577X. DOI: 10.1016/j.matlet.2018.01.177. URL: http://dx.doi.org/10.1016/j.matlet.2018.01.177.

[96] Somsubhra Saha et al. “Self-assembly of silver nanocolloids in the Langmuir–Blodgett Film of stearic acid: Evidence of an efficient SERS sensing platform”. In: *Journal of Raman Spectroscopy* 47.2 (2015),

168–176. ISSN: 1097-4555. DOI: 10.1002/jrs.4771. URL: http://dx.doi.org/10.1002/jrs.4771.

[97] Ali Hossain Khan et al. "Two-Dimensional (2D) Nanomaterials towards Electrochemical Nanoarchitectonics in Energy-Related Applications". In: *Bulletin of the Chemical Society of Japan* 90.6 (2017), 627–648. ISSN: 1348-0634. DOI: 10.1246/bcsj.20170043. URL: http://dx.doi.org/10.1246/bcsj.20170043.

[98] Cheng Qian et al. "Facile preparation of self-assembled black phosphorus-based composite LB films as new chemical gas sensors". In: *Colloids and Surfaces A: Physicochemical and Engineering Aspects* 608 (2021), p. 125616. ISSN: 0927-7757. DOI: 10.1016/j.colsurfa.2020.125616. URL: http://dx.doi.org/10.1016/j.colsurfa.2020.125616.

[99] Alowasheeir Azhar et al. "Nanoarchitectonics: A New Materials Horizon for Prussian Blue and Its Analogues". In: *Bulletin of the Chemical Society of Japan* 92.4 (2019), 875–904. ISSN: 1348-0634. DOI: 10.1246/bcsj.20180368. URL: http://dx.doi.org/10.1246/bcsj.20180368.

[100] Rui Zheng et al. "The Growth Mechanism of a Conductive MOF Thin Film in Spray-based Layer-by-layer Liquid Phase Epitaxy". In: *Angewandte Chemie* 134.43 (2022). ISSN: 1521-3757. DOI: 10.1002/ange.202212797. URL: http://dx.doi.org/10.1002/ange.202212797.

[101] Adrien P. Cote et al. "Porous, Crystalline, Covalent Organic Frameworks". In: *Science* 310.5751 (2005), 1166–1170. ISSN: 1095-9203. DOI: 10.1126/science.1120411. URL: http://dx.doi.org/10.1126/science.1120411.

[102] Bin Bai, Dong Wang, and Li-Jun Wan. "Synthesis of Covalent Organic Framework Films at Interfaces". In: *Bulletin of the Chemical Society of Japan* 94.3 (2021), 1090–1098. ISSN: 1348-0634. DOI: 10.1246/bcsj.20200391. URL: http://dx.doi.org/10.1246/bcsj.20200391.

[103] Donglin Jiang. "Covalent Organic Frameworks: A Molecular Platform for Designer Polymeric Architectures and Functional Materials". In: *Bulletin of the Chemical Society of Japan* 94.4 (2021), 1215–1231. ISSN: 1348-0634. DOI: 10.1246/bcsj.20200389. URL: http://dx.doi.org/10.1246/bcsj.20200389.

[104] Haruna Mabuchi et al. “Covalent Organic Frameworks: Cutting-Edge Materials for Carbon Dioxide Capture and Water Harvesting from Air”. In: *Chemistry – A European Journal* 30.6 (2024). ISSN: 1521-3765. DOI: 10.1002/chem.202303474. URL: http://dx.doi.org/10.1002/chem.202303474.

[105] Arkaprabha Giri et al. “Transformation of an Imine Cage to a Covalent Organic Framework Film at the Liquid–Liquid Interface”. In: *Angewandte Chemie* 135.23 (2023). ISSN: 1521-3757. DOI: 10.1002/ange.202219083. URL: http://dx.doi.org/10.1002/ange.202219083.

[106] Jeremy I. Feldblyum et al. “Few-layer, large-area, 2D covalent organic framework semiconductor thin films”. In: *Chemical Communications* 51.73 (2015), 13894–13897. ISSN: 1364-548X. DOI: 10.1039/c5cc04679c. URL: http://dx.doi.org/10.1039/c5cc04679c.

[107] Corrado Di Natale et al. “Pattern recognition approach to the study of the interactions between metalloporphyrin Langmuir–Blodgett films and volatile organic compounds”. In: *Analytica Chimica Acta* 384.3 (1999), 249–259. ISSN: 0003-2670. DOI: 10.1016/s0003-2670(98)00783-1. URL: http://dx.doi.org/10.1016/s0003-2670(98)00783-1.

[108] Xiaoyu Li et al. “Porphyrin-Based Covalent Organic Frameworks: Design, Synthesis, Photoelectric Conversion Mechanism, and Applications”. In: *Biomimetics* 8.2 (2023), p. 171. ISSN: 2313-7673. DOI: 10.3390/biomimetics8020171. URL: http://dx.doi.org/10.3390/biomimetics8020171.

[109] Da Shi et al. “Interfacial Behavior of Oligo(Ethylene Glycol) Dendrons Spread Alone and in Combination with a Phospholipid as Langmuir Monolayers at the Air/Water Interface”. In: *Molecules* 24.22 (2019), p. 4114. ISSN: 1420-3049. DOI: 10.3390/molecules24224114. URL: http://dx.doi.org/10.3390/molecules24224114.

[110] X. M. Yang et al. “Domain structures of phospholipid monolayer Langmuir-Blodgett films determined by atomic force microscopy”. In: *Applied Physics A Solids and Surfaces* 59.2 (1994), 139–143. ISSN: 1432-0630. DOI: 10.1007/bf00332206. URL: http://dx.doi.org/10.1007/bf00332206.

[111] Changxin Zhu et al. "Morphology of monolayer LB film by STM". In: *Vacuum* 43.11 (1992), 1111–1113. ISSN: 0042-207X. DOI: 10.1016/0042-207x(92)90345-w. URL: http://dx.doi.org/10.1016/0042-207x(92)90345-w.

[112] Ahmad Shakeel et al. "Advanced polymeric/inorganic nanohybrids: An integrated platform for gas sensing applications". In: *Chemosphere* 294 (2022), p. 133772. ISSN: 0045-6535. DOI: 10.1016/j.chemosphere.2022.133772. URL: http://dx.doi.org/10.1016/j.chemosphere.2022.133772.

[113] Hyeri Kim et al. "Spontaneous hybrids of graphene and carbon nanotube arrays at the liquid–gas interface for Li-ion battery anodes". In: *Chemical Communications* 54.41 (2018), 5229–5232. ISSN: 1364-548X. DOI: 10.1039/c8cc02148a. URL: http://dx.doi.org/10.1039/c8cc02148a.

[114] Hexing Liu et al. "Greatly enhanced energy density in polymer nanocomposites coated with high-k perovskite nanosheets". In: *Materials Today Energy* 31 (2023), p. 101213. ISSN: 2468-6069. DOI: 10.1016/j.mtener.2022.101213. URL: http://dx.doi.org/10.1016/j.mtener.2022.101213.

[115] Shama Perween et al. "PVA-PDMS-Stearic acid composite nanofibrous mats with improved mechanical behavior for selective filtering applications". In: *Scientific Reports* 8.1 (2018). ISSN: 2045-2322. DOI: 10.1038/s41598-018-34440-5. URL: http://dx.doi.org/10.1038/s41598-018-34440-5.

[116] Anna Vikulina et al. "A lipid membrane supported on an artificial extracellular matrix made of polyelectrolyte multilayers: towards nanoarchitectonics at the cellular interface". In: *Nanoscale* 15.5 (2023), 2197–2205. ISSN: 2040-3372. DOI: 10.1039/d2nr05186a. URL: http://dx.doi.org/10.1039/d2nr05186a.

[117] Edward T. Castellana and Paul S. Cremer. "Solid-Supported Lipid Bilayers: From Biophysical Studies to Sensor Design". In: *ChemInform* 38.14 (2007). ISSN: 1522-2667. DOI: 10.1002/chin.200714253. URL: http://dx.doi.org/10.1002/chin.200714253.

[118] Agnès P. Girard-Egrot, Stéphanie Godoy, and Loïc J. Blum. "Enzyme association with lipidic Langmuir–Blodgett films: Interests and applications in nanobioscience". In: *Advances in Colloid and Interface Science* 116.1–3 (2005), 205–225. ISSN: 0001-8686. DOI: 10.1016/j.cis.2005.04.006. URL: http://dx.doi.org/10.1016/j.cis.2005.04.006.

[119] José R. Siqueira et al. "Immobilization of biomolecules on nanostructured films for biosensing". In: *Biosensors and Bioelectronics* 25.6 (2010), 1254–1263. ISSN: 0956-5663. DOI: 10.1016/j.bios.2009.09.043. URL: http://dx.doi.org/10.1016/j.bios.2009.09.043.

[120] Ke-Hsuan Wang et al. "Architecture effects of glucose oxidase/Au nanoparticle composite Langmuir-Blodgett films on glucose sensing performance". In: *Applied Surface Science* 366 (2016), 202–209. ISSN: 0169-4332. DOI: 10.1016/j.apsusc.2016.01.047. URL: http://dx.doi.org/10.1016/j.apsusc.2016.01.047.

[121] Ilya Gorbachev et al. "Langmuir–Blodgett Films with Immobilized Glucose Oxidase Enzyme Molecules for Acoustic Glucose Sensor Application". In: *Sensors* 23.11 (2023), p. 5290. ISSN: 1424-8220. DOI: 10.3390/s23115290. URL: http://dx.doi.org/10.3390/s23115290.

[122] Ke-Hsuan Wang et al. "Immobilization of glucose oxidase by Langmuir–Blodgett technique for fabrication of glucose biosensors: Headgroup effects of template monolayers". In: *Sensors and Actuators B: Chemical* 164.1 (2012), 29–36. ISSN: 0925-4005. DOI: 10.1016/j.snb.2012.01.056. URL: http://dx.doi.org/10.1016/j.snb.2012.01.056.

[123] Camila Gouveia Barbosa et al. "Copolymers and enzymes blended as LB films changing the bioelectronics properties of interfaces". In: *Colloid and Interface Science Communications* 27 (2018), 40–44. ISSN: 2215-0382. DOI: 10.1016/j.colcom.2018.10.001. URL: http://dx.doi.org/10.1016/j.colcom.2018.10.001.

[124] R. Verger and F. Pattus. "Lipid-protein interactions in monolayers". In: *Chemistry and Physics of Lipids* 30.2–3 (1982), 189–227. ISSN: 0009-3084. DOI: 10.1016/0009-3084(82)90052-4. URL: http://dx.doi.org/10.1016/0009-3084(82)90052-4.

[125] Masahiro Yamashita. "Next Generation Multifunctional Nano-Science of Advanced Metal Complexes with Quantum Effect and Nonlinearity". In: *Bulletin of the Chemical Society of Japan* 94.1 (2020), 209–264. ISSN: 1348-0634. DOI: 10.1246/bcsj.20200257. URL: http://dx.doi.org/10.1246/bcsj.20200257.

[126] Katsuhiko Ariga. "Materials Nanoarchitectonics: Collaboration between Chem, Nano and Mat". In: *ChemNanoMat* 9.7 (2023). ISSN: 2199-692X. DOI: 10.1002/cnma.202300120. URL: http://dx.doi.org/10.1002/cnma.202300120.

[127] C. Medina-Plaza et al. "Synergistic electrocatalytic effect of nanostructured mixed films formed by functionalised gold nanoparticles and bisphthalocyanines". In: *Analytica Chimica Acta* 851 (2014), 95–102. ISSN: 0003-2670. DOI: 10.1016/j.aca.2014.08.049. URL: http://dx.doi.org/10.1016/j.aca.2014.08.049.

[128] Mingshan Zhu et al. "Photocatalytic Hydrogen Evolution Based on Efficient Energy and Electron Transfers in Donor–Bridge–Acceptor Multibranched-Porphyrin-Functionalized Platinum Nanocomposites". In: *Chemistry – A European Journal* 18.14 (2012), 4367–4374. ISSN: 1521-3765. DOI: 10.1002/chem.201102595. URL: http://dx.doi.org/10.1002/chem.201102595.

[129] Robert W Carpick et al. "Polydiacetylene films: a review of recent investigations into chromogenic transitions and nanomechanical properties". In: *Journal of Physics: Condensed Matter* 16.23 (2004), R679–R697. ISSN: 1361-648X. DOI: 10.1088/0953-8984/16/23/r01. URL: http://dx.doi.org/10.1088/0953-8984/16/23/r01.

[130] Naureen Akhtar et al. "Structure and Electrical Conductivity of Hybrid Langmuir–Blodgett Films from BEDO-TTF and Fatty Acid". In: *The Journal of Physical Chemistry C* 116.45 (2012), 24130–24135. ISSN: 1932-7455. DOI: 10.1021/jp307702k. URL: http://dx.doi.org/10.1021/jp307702k.

[131] H Ohnuki et al. "Metallic Langmuir and Langmuir–Blodgett films based on TTF derivatives and fatty acid". In: *Materials Science and Engineering: C* 22.2 (2002), 227–232. ISSN: 0928-4931. DOI: 10.1016/s0928-4931(02)00170-4. URL: http://dx.doi.org/10.1016/s0928-4931(02)00170-4.

[132] Ikbal Ahmed et al. "Chitosan-fatty acid interaction mediated growth of Langmuir monolayer and Langmuir-Blodgett films". In: *Journal of Colloid and Interface Science* 514 (2018), 433–442. ISSN: 0021-9797. DOI: 10.1016/j.jcis.2017.12.037. URL: http://dx.doi.org/10.1016/j.jcis.2017.12.037.

[133] Rebeca da Rocha Rodrigues et al. "Conjugated polymers as Langmuir and Langmuir-Blodgett films: Challenges and applications in nanostructured devices". In: *Advances in Colloid and Interface Science* 285 (2020), p. 102277. ISSN: 0001-8686. DOI: 10.1016/j.cis.2020.102277. URL: http://dx.doi.org/10.1016/j.cis.2020.102277.

[134] Andrei Sakai, Laura O. Péres, and Luciano Caseli. "Langmuir and Langmuir-Blodgett films of Cl-PPV mixed with stearic acid: implication of the morphology on the surface and spectroscopy properties". In: *Colloid and Polymer Science* 293.3 (2014), 883–890. ISSN: 1435-1536. DOI: 10.1007/s00396-014-3477-4. URL: http://dx.doi.org/10.1007/s00396-014-3477-4.

[135] Thays C. F. Santos et al. "Mixing Alternating Copolymers Containing Fluorenyl Groups with Phospholipids to Obtain Langmuir and LangmuirBlodgett Films". In: *Langmuir* 26.8 (2009), 5869–5875. ISSN: 1520-5827. DOI: 10.1021/la9038107. URL: http://dx.doi.org/10.1021/la9038107.

[136] Thiago E. Goto et al. "Langmuir–Blodgett films based on poly(p-phenylene vinylene) and protein-stabilised palladium nanoparticles: Implications in luminescent and conducting properties". In: *Thin Solid Films* 540 (2013), 202–207. ISSN: 0040-6090. DOI: 10.1016/j.tsf.2013.05.106. URL: http://dx.doi.org/10.1016/j.tsf.2013.05.106.

[137] M. S. Kim et al. "Fabricating multifunctional nanoparticle membranes by a fast layer-by-layer Langmuir–Blodgett process: application in lithium–sulfur batteries". In: *Journal of Materials Chemistry A* 4.38 (2016), 14709–14719. ISSN: 2050-7496. DOI: 10.1039/c6ta06018h. URL: http://dx.doi.org/10.1039/c6ta06018h.

[138] Pengfei Bian et al. "Interfacial aggregation behavior of novel carbazole-based composite Langmuir-Blodgett films for photoelectric conversion and catalytic performance". In: *Colloids and Surfaces A: Physicochemical and Engineering Aspects* 656 (2023), p. 130460. ISSN: 0927-

7757. DOI: 10.1016/j.colsurfa.2022.130460. URL: http://dx.doi.org/10.1016/j.colsurfa.2022.130460.

[139] S.A. Hussain, P.K. Paul, and D. Bhattacharjee. “Role of various LB parameters on the optical characteristics of mixed Langmuir–Blodgett films”. In: *Journal of Physics and Chemistry of Solids* 67.12 (2006), 2542–2549. ISSN: 0022-3697. DOI: 10.1016/j.jpcs.2006.07.011. URL: http://dx.doi.org/10.1016/j.jpcs.2006.07.011.

[140] Inseok Chae et al. “Anisotropic Optical and Frictional Properties of Langmuir–Blodgett Film Consisting of Uniaxially-Aligned Rod-Shaped Cellulose Nanocrystals”. In: *Advanced Materials Interfaces* 7.9 (2020). ISSN: 2196-7350. DOI: 10.1002/admi.201902169. URL: http://dx.doi.org/10.1002/admi.201902169.

[141] Rafael Leonardo Cruz Gomes da Silva et al. “Surface Chemistry Studies on the Formation of Mixed Stearic Acid/Phenylalanine Dehydrogenase Langmuir and Langmuir–Blodgett Films”. In: *Langmuir* 37.25 (2021), 7771–7779. ISSN: 1520-5827. DOI: 10.1021/acs.langmuir.1c00934. URL: http://dx.doi.org/10.1021/acs.langmuir.1c00934.

[142] Luciano Caseli et al. “Fabrication of Phytic Acid Sensor Based on Mixed Phytaseâˆ’Lipid Langmuirâˆ’Blodgett Films”. In: *Langmuir* 22.20 (2006), 8501â€“8508. ISSN: 1520-5827. DOI: 10.1021/la061799g. URL: http://dx.doi.org/10.1021/la061799g.

[143] Florencio Peñacorada et al. “Brewster angle microscopy and surface potential measurements of Langmuir–Blodgett films of zinc tri(tert-butyl)-4-sulphophthalocyanine”. In: *Applied Surface Science* 246.4 (2005), 425–429. ISSN: 0169-4332. DOI: 10.1016/j.apsusc.2004.11.047. URL: http://dx.doi.org/10.1016/j.apsusc.2004.11.047.

[144] Fumiko Kimura, Junzo Umemura, and Tohru Takenaka. “FTIR-ATR studies on Langmuir-Blodgett films of stearic acid with 1-9 monolayers”. In: *Langmuir* 2.1 (1986), 96–101. ISSN: 1520-5827. DOI: 10.1021/la00067a017. URL: http://dx.doi.org/10.1021/la00067a017.

[145] B. Lecourt, D. Blaudez, and J.M. Turlet. “Anisotropy in Langmuir–Blodgett films studied by generalized spectroscopic ellipsometry”. In: *Thin Solid Films* 313–314 (1998), 790–794. ISSN: 0040-6090. DOI: 10.1016/s0040-6090(97)00996-6. URL: http://dx.doi.org/10.1016/s0040-6090(97)00996-6.

[146] Toshihide Kamata, Junzo Umemura, and Tohru Takenaka. "Structure Study of Langmuir–Blodgett Films of Stearic Acid and Cadmium Stearate Deposited by Different Techniques". In: *Chemistry Letters* 17.7 (1988), 1231–1234. ISSN: 1348-0715. DOI: 10.1246/cl.1988.1231. URL: http://dx.doi.org/10.1246/cl.1988.1231.

[147] C.A. Helm et al. "Phospholipid monolayers between fluid and solid states". In: *Biophysical Journal* 52.3 (1987), 381–390. ISSN: 0006-3495. DOI: 10.1016/s0006-3495(87)83226-5. URL: http://dx.doi.org/10.1016/s0006-3495(87)83226-5.

[148] Alfred Blume. "A comparative study of the phase transitions of phospholipid bilayers and monolayers". In: *Biochimica et Biophysica Acta (BBA) - Biomembranes* 557.1 (1979), 32–44. ISSN: 0005-2736. DOI: 10.1016/0005-2736(79)90087-7. URL: http://dx.doi.org/10.1016/0005-2736(79)90087-7.

[149] Jaemyung Kim, Laura J. Cote, and Jiaxing Huang. "Two Dimensional Soft Material: New Faces of Graphene Oxide". In: *Accounts of Chemical Research* 45.8 (2012), 1356–1364. ISSN: 1520-4898. DOI: 10.1021/ar300047s. URL: http://dx.doi.org/10.1021/ar300047s.

[150] Mahdi Tavakol et al. "Mechanical properties of graphene oxide: The impact of functional groups". In: *Applied Surface Science* 525 (2020), p. 146554. ISSN: 0169-4332. DOI: 10.1016/j.apsusc.2020.146554. URL: http://dx.doi.org/10.1016/j.apsusc.2020.146554.

[151] D.M. Taylor and G.F. Bayes. "The surface potential of Langmuir monolayers". In: *Materials Science and Engineering: C* 8–9 (1999), 65–71. ISSN: 0928-4931. DOI: 10.1016/s0928-4931(99)00064-8. URL: http://dx.doi.org/10.1016/s0928-4931(99)00064-8.

[152] H M McConnell and Y B Bazaliy. "Lipid monolayer image dipoles." In: *Proceedings of the National Academy of Sciences* 92.19 (1995), 8823–8825. ISSN: 1091-6490. DOI: 10.1073/pnas.92.19.8823. URL: http://dx.doi.org/10.1073/pnas.92.19.8823.

[153] Richard J Demchak and Tomlinson Fort. "Surface dipole moments of close-packed un-ionized monolayers at the air-water interface". In: *Journal of Colloid and Interface Science* 46.2 (1974), 191–202. ISSN: 0021-9797. DOI: 10.1016/0021-9797(74)90002-2. URL: http://dx.doi.org/10.1016/0021-9797(74)90002-2.

[154] Howard Brockman. “Dipole potential of lipid membranes”. In: *Chemistry and Physics of Lipids* 73.1–2 (1994), 57–79. ISSN: 0009-3084. DOI: 10.1016/0009-3084(94)90174-0. URL: http://dx.doi.org/10.1016/0009-3084(94)90174-0.

[155] Darryl Y. Masayuki et al. “The interaction of a guanidinium monolayer with ATP and AMP, as revealed by surface potential and UV absorption measurements”. In: *Thin Solid Films* 210–211 (1992), 776–779. ISSN: 0040-6090. DOI: 10.1016/0040-6090(92)90401-v. URL: http://dx.doi.org/10.1016/0040-6090(92)90401-v.

[156] Patrycja Dynarowicz-Łatka, Anantharaman Dhanabalan, and Osvaldo N. Oliveira. “Modern physicochemical research on Langmuir monolayers”. In: *Advances in Colloid and Interface Science* 91.2 (2001), 221–293. ISSN: 0001-8686. DOI: 10.1016/s0001-8686(99)00034-2. URL: http://dx.doi.org/10.1016/s0001-8686(99)00034-2.

[157] Juan J. Giner-Casares, Gerald Brezesinski, and Helmuth Möhwald. “Langmuir monolayers as unique physical models”. In: *Current Opinion in Colloid amp; Interface Science* 19.3 (2014), 176–182. ISSN: 1359-0294. DOI: 10.1016/j.cocis.2013.07.006. URL: http://dx.doi.org/10.1016/j.cocis.2013.07.006.

[158] J. Garnaes et al. “Nano scale defects in langmuir-blodgett film observed by atomic force microscopy”. In: *Synthetic Metals* 57.1 (1993), 3795–3800. ISSN: 0379-6779. DOI: 10.1016/0379-6779(93)90515-x. URL: http://dx.doi.org/10.1016/0379-6779(93)90515-x.

[159] Keerti Choudhary, Manjuladevi V., and R. K. Gupta. “Studies on morphology of Langmuir-Blodgett films of stearic acid deposited with different orientation of substrates with respect to compression”. In: *AIP Conference Proceedings*. Vol. 1728. Author(s), 2016, p. 020159. DOI: 10.1063/1.4946210. URL: http://dx.doi.org/10.1063/1.4946210.

[160] J. Meunier. “Why a Brewster angle microscope?” In: *Colloids and Surfaces A: Physicochemical and Engineering Aspects* 171.1–3 (2000), 33–40. ISSN: 0927-7757. DOI: 10.1016/s0927-7757(99)00555-5. URL: http://dx.doi.org/10.1016/s0927-7757(99)00555-5.

[161] Weiam Daear, Mark Mahadeo, and Elmar J. Prenner. "Applications of Brewster angle microscopy from biological materials to biological systems". In: *Biochimica et Biophysica Acta (BBA) - Biomembranes* 1859.10 (2017), 1749–1766. ISSN: 0005-2736. DOI: 10.1016/j.bbamem.2017.06.016. URL: http://dx.doi.org/10.1016/j.bbamem.2017.06.016.

[162] Cristina Roldán-Carmona et al. "Revisiting the Brewster Angle Microscopy: The relevance of the polar headgroup". In: *Advances in Colloid and Interface Science* 173 (2012), 12–22. ISSN: 0001-8686. DOI: 10.1016/j.cis.2012.02.002. URL: http://dx.doi.org/10.1016/j.cis.2012.02.002.

[163] Elena Maltseva et al. "Adsorption of Amyloid (1–40) Peptide at Phospholipid Monolayers". In: *ChemBioChem* 6.10 (2005), 1817–1824. ISSN: 1439-7633. DOI: 10.1002/cbic.200500116. URL: http://dx.doi.org/10.1002/cbic.200500116.

[164] Adrien Sthoer et al. "La3+ and Y3+ interactions with the carboxylic acid moiety at the liquid/vapour interface: identification of binding complexes, charge reversal, and detection limits." In: (2021). DOI: 10.26434/chemrxiv-2021-28p74. URL: http://dx.doi.org/10.26434/chemrxiv-2021-28p74.

[165] Katarzyna Hac-Wydro et al. "Properties of -sitostanol/DPPC monolayers studied with Grazing Incidence X-ray Diffraction (GIXD) and Brewster Angle Microscopy". In: *Journal of Colloid and Interface Science* 364.1 (2011), 133–139. ISSN: 0021-9797. DOI: 10.1016/j.jcis.2011.08.030. URL: http://dx.doi.org/10.1016/j.jcis.2011.08.030.

[166] Benjamin L. Stottrup, Andrew H. Nguyen, and Erkan Tüzel. "Taking another look with fluorescence microscopy: Image processing techniques in Langmuir monolayers for the twenty-first century". In: *Biochimica et Biophysica Acta (BBA) - Biomembranes* 1798.7 (2010), 1289–1300. ISSN: 0005-2736. DOI: 10.1016/j.bbamem.2010.01.003. URL: http://dx.doi.org/10.1016/j.bbamem.2010.01.003.

[167] Brian Moore et al. "Studies of phase transitions in Langmuir monolayers by fluorescence microscopy". In: *Journal of the Chemical Society, Faraday Transactions 2* 82.10 (1986), p. 1753. ISSN: 0300-9238. DOI:

10.1039/f29868201753. URL: http://dx.doi.org/10.1039/f29868201753.

[168] Daniel K. Schwartz and Charles M. Knobler. "Direct observations of transitions between condensed Langmuir monolayer phases by polarized fluorescence microscopy". In: *The Journal of Physical Chemistry* 97.35 (1993), 8849–8851. ISSN: 1541-5740. DOI: 10.1021/j100137a005. URL: http://dx.doi.org/10.1021/j100137a005.

[169] Kannan Ayyavoo and Praveena Velusamy. "Pyrene based materials as fluorescent probes in chemical and biological fields". In: *New Journal of Chemistry* 45.25 (2021), 10997–11017. ISSN: 1369-9261. DOI: 10.1039/d1nj00158b. URL: http://dx.doi.org/10.1039/d1nj00158b.

[170] Yi Chen. "Recent Advances in Excimer-Based Fluorescence Probes for Biological Applications". In: *Molecules* 27.23 (2022), p. 8628. ISSN: 1420-3049. DOI: 10.3390/molecules27238628. URL: http://dx.doi.org/10.3390/molecules27238628.

[171] Bernard Valeur. *Molecular Fluorescence.* 2009. DOI: 10.1002/3527600434.eap684. URL: http://dx.doi.org/10.1002/3527600434.eap684.

[172] Maria Rosaria di Nunzio et al. "Interrogating the Behaviour of a Styryl Dye Interacting with a Mesoscopic 2D-MOF and Its Luminescent Vapochromic Sensing". In: *International Journal of Molecular Sciences* 23.1 (2021), p. 330. ISSN: 1422-0067. DOI: 10.3390/ijms23010330. URL: http://dx.doi.org/10.3390/ijms23010330.

[173] Mark A. Haidekker et al. "New fluorescent probes for the measurement of cell membrane viscosity". In: *Chemistry amp; Biology* 8.2 (2001), 123–131. ISSN: 1074-5521. DOI: 10.1016/s1074-5521(00)90061-9. URL: http://dx.doi.org/10.1016/s1074-5521(00)90061-9.

[174] Waka Nakanishi et al. "Monitoring Fluorescence Response of Amphiphilic Flapping Molecules in Compressed Monolayers at the Air–Water Interface". In: *Chemistry – An Asian Journal* 14.16 (2019), 2869–2876. ISSN: 1861-471X. DOI: 10.1002/asia.201900769. URL: http://dx.doi.org/10.1002/asia.201900769.

[175] D Vu and K Birdi. "Application of Scanning Probe Microscopy (Scanning Tunneling Microscopy and Atomic Force Microscopy) in Colloid and Surface Chemistry". In: *Handbook of Surface and Colloid Chemistry, Second Edition.* CRC Press, 2002. ISBN: 9781420040944. DOI:

10.1201/9781420040944.ch15. URL: http://dx.doi.org/10.1201/9781420040944.ch15.

[176] Carlos Marcuello et al. "Langmuir–Blodgett Procedure to Precisely Control the Coverage of Functionalized AFM Cantilevers for SMFS Measurements: Application with Cellulose Nanocrystals". In: *Langmuir* 34.32 (2018), 9376–9386. ISSN: 1520-5827. DOI: 10.1021/acs.langmuir.8b01892. URL: http://dx.doi.org/10.1021/acs.langmuir.8b01892.

[177] Fumiko Yano and Setsuo Nomura. "Deconvolution of scanning electron microscopy images". In: *Scanning* 15.1 (1993), 19–24. ISSN: 1932-8745. DOI: 10.1002/sca.4950150103. URL: http://dx.doi.org/10.1002/sca.4950150103.

[178] Dmitri Golberg and et al. et al. "ChemInform Abstract: Nanomaterial Engineering and Property Studies in a Transmission Electron Microscope". In: *ChemInform* 43.9 (2012). ISSN: 1522-2667. DOI: 10.1002/chin.201209238. URL: http://dx.doi.org/10.1002/chin.201209238.

[179] P Bajaj et al. "Transmission Electron Microscopy and Three-Dimensional Tomography of Peptide-Coated Single-Walled Carbon Nanotubes". In: *Microscopy and Microanalysis* 17.S2 (2011), 1008–1009. ISSN: 1435-8115. DOI: 10.1017/s1431927611005915. URL: http://dx.doi.org/10.1017/s1431927611005915.

[180] David L. Allara and Ping Zhang. *Characterization of Surfaces, Interfaces, and Thin Films of Organic Materials*. 2006. DOI: 10.1002/9783527603978.mst0030. URL: http://dx.doi.org/10.1002/9783527603978.mst0030.

[181] Gerhard Herzberg and Bryce L. Crawford. "Infrared and Raman Spectra of Polyatomic Molecules." In: *The Journal of Physical Chemistry* 50.3 (1946), 288–288. ISSN: 1541-5740. DOI: 10.1021/j150447a021. URL: http://dx.doi.org/10.1021/j150447a021.

[182] Nobutaka Shioya et al. "Multiple-angle incidence resolution spectrometry: applications in nanoarchitectonics and applied physics". In: *Japanese Journal of Applied Physics* 63.6 (2024), p. 060102. ISSN: 1347-4065. DOI: 10.35848/1347-4065/ad4ad8. URL: http://dx.doi.org/10.35848/1347-4065/ad4ad8.

[183] Asra Siddique, Ambreen Akram, and Yasir Jamil. "Fine structure in atomic spectra: electron spin and energy". In: *Modern Luminescence from Fundamental Concepts to Materials and Applications*. Elsevier, 2023, 51–84. ISBN: 9780323899543. DOI: 10.1016/b978-0-323-89954-3.00002-8. URL: http://dx.doi.org/10.1016/b978-0-323-89954-3.00002-8.

[184] Andreas Klamt. "Calculation of UV/Vis Spectra in Solution". In: *The Journal of Physical Chemistry* 100.9 (1996), 3349–3353. ISSN: 1541-5740. DOI: 10.1021/jp950607f. URL: http://dx.doi.org/10.1021/jp950607f.

[185] Zhao Qing-nan et al. "UV-vis and photoluminescent spectra of TiO2 films". In: *Journal of Wuhan University of Technology-Mater. Sci. Ed.* 18.3 (2003), 36–39. ISSN: 1993-0437. DOI: 10.1007/bf02838454. URL: http://dx.doi.org/10.1007/bf02838454.

[186] José F. Marco, Claudio Gutiérrez, and María Soledad Ureta-Zañartu. "Study by XPS and UVVisible and DRIFT Spectroscopies of Electropolymerized Films of Substituted Ni(II)-p-Phenylporphyrins and -Phthalocyanines". In: *The Journal of Physical Chemistry B* 112.40 (2008), 12644–12649. ISSN: 1520-5207. DOI: 10.1021/jp8046455. URL: http://dx.doi.org/10.1021/jp8046455.

[187] Denis M. Krichevsky et al. "Resonant Plasmon-Enhanced Absorption of Charge Transfer Complexes in a Metal–Organic Monolayer". In: *Advanced Optical Materials* 9.11 (2021). ISSN: 2195-1071. DOI: 10.1002/adom.202100065. URL: http://dx.doi.org/10.1002/adom.202100065.

[188] Takashi Sato, Yukihiro Ozaki, and Keiji Iriyama. "Molecular Aggregation and Photoisomerization of Langmuir-Blodgett Films of Azobenzene-Containing Long-Chain Fatty Acids and Their Salts Studied by Ultraviolet-Visible and Infrared Spectroscopies". In: *Langmuir* 10.7 (1994), 2363–2369. ISSN: 1520-5827. DOI: 10.1021/la00019a055. URL: http://dx.doi.org/10.1021/la00019a055.

[189] Miharu Eguchi et al. "Adsorchromism: Molecular Nanoarchitectonics at 2D Nanosheets—Old Chemistry for Advanced Chromism". In: *Advanced Science* 8.14 (2021). ISSN: 2198-3844. DOI: 10.1002/advs.202100539. URL: http://dx.doi.org/10.1002/advs.202100539.

[190] Yang Yu et al. “Exploration of Optical Properties of Novel Pyrene Derivatives Modified by Click Functionalization”. In: *Crystals* 12.9 (2022), p. 1295. ISSN: 2073-4352. DOI: 10.3390/cryst12091295. URL: http://dx.doi.org/10.3390/cryst12091295.

[191] Hajime Maeda et al. “UV Absorption and Fluorescence Properties of Pyrene Derivatives Having Trimethylsilyl, Trimethylgermyl, and Trimethylstannyl Groups”. In: *Chemistry Letters* 30.12 (2001), 1224–1225. ISSN: 1348-0715. DOI: 10.1246/cl.2001.1224. URL: http://dx.doi.org/10.1246/cl.2001.1224.

[192] Geeta A. Zalmi et al. “Recent Advances in Aggregation-Induced Emission Active Materials for Sensing of Biologically Important Molecules and Drug Delivery System”. In: *Molecules* 27.1 (2021), p. 150. ISSN: 1420-3049. DOI: 10.3390/molecules27010150. URL: http://dx.doi.org/10.3390/molecules27010150.

[193] Ming Hui Chua et al. “Recent advances in aggregation-induced emission (AIE)-based chemosensors for the detection of organic small molecules”. In: *Materials Chemistry Frontiers* 7.22 (2023), 5561–5660. ISSN: 2052-1537. DOI: 10.1039/d3qm00679d. URL: http://dx.doi.org/10.1039/d3qm00679d.

[194] D. Blaudez et al. “Polarization-Modulated FT-IR Spectroscopy of a Spread Monolayer at the Air/Water Interface”. In: *Applied Spectroscopy* 47.7 (1993), 869–874. ISSN: 1943-3530. DOI: 10.1366/0003702934415273. URL: http://dx.doi.org/10.1366/0003702934415273.

[195] Christophe Daniel and Margherita Lardone. “Characterization of Syndiotactic Polystyrene Nanofilms by PM-IRRAS Spectroscopy”. In: *Macromolecular Symposia* 359.1 (2016), 24–31. ISSN: 1521-3900. DOI: 10.1002/masy.201500073. URL: http://dx.doi.org/10.1002/masy.201500073.

[196] Luciano Caseli et al. “Investigation of the Conformational Changes of a Conducting Polymer in Gas Sensor Active Layers by Means of Polarization-Modulation Infrared Reflection Absorption Spectroscopy (PM-IRRAS).” In: *Langmuir* 29.8 (2013), 2640–2645. ISSN: 1520-5827. DOI: 10.1021/la3050797. URL: http://dx.doi.org/10.1021/la3050797.

[197] Takeshi Hasegawa et al. “Thermal Stability of Metal Stearate LB Films Studied by Infrared Reflection–Absorption Spectroscopy”. In: *Chemistry Letters* 19.9 (1990), 1543–1546. ISSN: 1348-0715. DOI: 10.1246/cl.1990.1543. URL: http://dx.doi.org/10.1246/cl.1990.1543.

[198] Y. R. Shen. “Surface properties probed by second-harmonic and sum-frequency generation”. In: *Nature* 337.6207 (1989), 519–525. ISSN: 1476-4687. DOI: 10.1038/337519a0. URL: http://dx.doi.org/10.1038/337519a0.

[199] Mitsumasa Iwamoto, Takaaki Manaka, and Eunju Lim. *Surface Second Harmonic and Sum-Frequency Generation*. 2009. DOI: 10.1002/3527600434.eap689. URL: http://dx.doi.org/10.1002/3527600434.eap689.

[200] P. B. Miranda and Y. R. Shen. “Liquid Interfaces: A Study by Sum-Frequency Vibrational Spectroscopy”. In: *The Journal of Physical Chemistry B* 103.17 (1999), 3292–3307. ISSN: 1520-5207. DOI: 10.1021/jp9843757. URL: http://dx.doi.org/10.1021/jp9843757.

[201] Marie C. Messmer, John C. Conboy, and Geraldine L. Richmond. “Observation of Molecular Ordering at the Liquid-Liquid Interface by Resonant Sum Frequency Generation”. In: *Journal of the American Chemical Society* 117.30 (1995), 8039–8040. ISSN: 1520-5126. DOI: 10.1021/ja00135a032. URL: http://dx.doi.org/10.1021/ja00135a032.

[202] X. Zhuang et al. “Mapping molecular orientation and conformation at interfaces by surface nonlinear optics”. In: *Physical Review B* 59.19 (1999), 12632–12640. ISSN: 1095-3795. DOI: 10.1103/physrevb.59.12632. URL: http://dx.doi.org/10.1103/physrevb.59.12632.

[203] Hirotsugu KIKUCHI. “Sum-Frequency Generation Vibrational Spectroscopy”. In: *Kobunshi* 51.10 (2002), 833–833. ISSN: 2185-9825. DOI: 10.1295/kobunshi.51.833. URL: http://dx.doi.org/10.1295/kobunshi.51.833.

[204] Shen YE and Aimin GE. “Sum Frequency GenerationSFGVibrational Spectroscopy”. In: *Journal of the Japan Society of Colour Material* 87.2 (2014), 64–71. ISSN: 1883-2199. DOI: 10.4011/shikizai.87.64. URL: http://dx.doi.org/10.4011/shikizai.87.64.

[205] Olga Zamyshlyayeva et al. "The surface behavior of mixed monolayers and LB films of betulin-containing polymers with lipids at the air-Ce3+ aqueous solution interface and on solid substrate". In: *Polymer Bulletin* 82.6 (2025), pp. 1799–1823. ISSN: 1436-2449. DOI: 10.1007/s00289-024-05573-7. URL: https://doi.org/10.1007/s00289-024-05573-7.

[206] Yu. Vlasov et al. "Nonspecific sensor arrays ("electronic tongue") for chemical analysis of liquids (IUPAC Technical Report)". In: *Pure and Applied Chemistry* 77.11 (2005), 1965–1983. ISSN: 0033-4545. DOI: 10.1351/pac200577111965. URL: http://dx.doi.org/10.1351/pac200577111965.

[207] Elizabeth A. Baldwin et al. "Electronic Noses and Tongues: Applications for the Food and Pharmaceutical Industries". In: *Sensors* 11.5 (2011), 4744–4766. ISSN: 1424-8220. DOI: 10.3390/s110504744. URL: http://dx.doi.org/10.3390/s110504744.

[208] Cátia Magro et al. "Overview of electronic tongue sensing in environmental aqueous matrices: potential for monitoring emerging organic contaminants". In: *Environmental Reviews* 27.2 (2019), 202–214. ISSN: 1208-6053. DOI: 10.1139/er-2018-0019. URL: http://dx.doi.org/10.1139/er-2018-0019.

[209] Joanna Cabaj, Jadwiga Sołoducho, and Anna Nowakowska-Oleksy. "Langmuir–Blodgett film based biosensor for estimation of phenol derivatives". In: *Sensors and Actuators B: Chemical* 143.2 (2010), 508–515. ISSN: 0925-4005. DOI: 10.1016/j.snb.2009.09.047. URL: http://dx.doi.org/10.1016/j.snb.2009.09.047.

[210] Rahul Singhal et al. "Immobilization of urease on poly(N-vinyl carbazole)/stearic acid Langmuir–Blodgett films for application to urea biosensor". In: *Biosensors and Bioelectronics* 17.8 (2002), 697–703. ISSN: 0956-5663. DOI: 10.1016/s0956-5663(02)00020-9. URL: http://dx.doi.org/10.1016/s0956-5663(02)00020-9.

[211] Shalini devi Kalyana Sundaram et al. "Enzyme Cascade Electrode Reactions with Nanomaterials and Their Applicability towards Biosensor and Biofuel Cells". In: *Biosensors* 13.12 (2023), p. 1018. ISSN: 2079-6374. DOI: 10.3390/bios13121018. URL: http://dx.doi.org/10.3390/bios13121018.

[212] Yuliang Zhao et al. “Glycated Hemoglobin Electrochemical Immunosensor Based on Screen-Printed Electrode”. In: *Biosensors* 12.10 (2022), p. 902. ISSN: 2079-6374. DOI: 10.3390/bios12100902. URL: http://dx.doi.org/10.3390/bios12100902.

[213] Li Ruiyi et al. “Electrochemical immunosensor for ultrasensitive detection of microcystin-LR based on graphene–gold nanocomposite/functional conducting polymer/gold nanoparticle/ionic liquid composite film with electrodeposition”. In: *Biosensors and Bioelectronics* 44 (2013), 235–240. ISSN: 0956-5663. DOI: 10.1016/j.bios.2013.01.007. URL: http://dx.doi.org/10.1016/j.bios.2013.01.007.

[214] Somsubhra Saha, Manash Ghosh, and Joydeep Chowdhury. “Infused self-assembly on Langmuir–Blodgett Film: Fabrication of highly efficient SERS active substrates with controlled plasmonic aggregates”. In: *Journal of Raman Spectroscopy* 50.3 (2018), 330–344. ISSN: 1097-4555. DOI: 10.1002/jrs.5529. URL: http://dx.doi.org/10.1002/jrs.5529.

[215] Carla Ivonne La Fuente Arias et al. “Bio-based multilayer films: A review of the principal methods of production and challenges”. In: *Critical Reviews in Food Science and Nutrition* 63.14 (2021), 2260–2276. ISSN: 1549-7852. DOI: 10.1080/10408398.2021.1973955. URL: http://dx.doi.org/10.1080/10408398.2021.1973955.

[216] Katsuhiko Ariga et al. “Materials Nanoarchitectonics as Cell Regulators”. In: *ChemNanoMat* 5.6 (2019), 692–702. ISSN: 2199-692X. DOI: 10.1002/cnma.201900207. URL: http://dx.doi.org/10.1002/cnma.201900207.

[217] Katsuhiko Ariga et al. “Life science nanoarchitectonics at interfaces”. In: *Materials Chemistry Frontiers* 5.3 (2021), 1018–1032. ISSN: 2052-1537. DOI: 10.1039/d0qm00615g. URL: http://dx.doi.org/10.1039/d0qm00615g.

[218] Xiaofang Jia et al. “Engineering dynamic and interactive biomaterials using material nanoarchitectonics for modulation of cellular behaviors”. In: *Cell Reports Physical Science* 4.2 (2023), p. 101251. ISSN: 2666-3864. DOI: 10.1016/j.xcrp.2023.101251. URL: http://dx.doi.org/10.1016/j.xcrp.2023.101251.

[219] Xiaofang Jia et al. “Adaptive Liquid Interfacially Assembled Protein Nanosheets for Guiding Mesenchymal Stem Cell Fate”. In: *Advanced Materials* 32.4 (2019). ISSN: 1521-4095. DOI: 10.1002/adma.201905942. URL: http://dx.doi.org/10.1002/adma.201905942.

[220] Wei Hu et al. “Regulation of stem cell fate and function by using bioactive materials with nanoarchitectonics for regenerative medicine”. In: *Science and Technology of Advanced Materials* 23.1 (2022), 393–412. ISSN: 1878-5514. DOI: 10.1080/14686996.2022.2082260. URL: http://dx.doi.org/10.1080/14686996.2022.2082260.

[221] Xiaofang Jia et al. “Adaptive liquid interfaces induce neuronal differentiation of mesenchymal stem cells through lipid raft assembly”. In: *Nature Communications* 13.1 (2022). ISSN: 2041-1723. DOI: 10.1038/s41467-022-30622-y. URL: http://dx.doi.org/10.1038/s41467-022-30622-y.

[222] Jianmin Xu et al. “Langmuir and Langmuir–Blodgett films of quantum dots”. In: *Colloids and Surfaces A: Physicochemical and Engineering Aspects* 284–285 (2006), 35–42. ISSN: 0927-7757. DOI: 10.1016/j.colsurfa.2005.11.046. URL: http://dx.doi.org/10.1016/j.colsurfa.2005.11.046.

[223] Geoffrey J. Ashwell et al. “Dipole reversal in Langmuir–Blodgett films of an optically nonlinear dye and its effect on the polarity for molecular rectification”. In: *Journal of Materials Chemistry* 15.39 (2005), p. 4203. ISSN: 1364-5501. DOI: 10.1039/b506765k. URL: http://dx.doi.org/10.1039/b506765k.

[224] David O. Oluwole et al. “First Example of Nonlinear Optical Materials Based on Nanoconjugates of Sandwich Phthalocyanines with Quantum Dots”. In: *Chemistry – A European Journal* 23.12 (2017), 2820–2830. ISSN: 1521-3765. DOI: 10.1002/chem.201604401. URL: http://dx.doi.org/10.1002/chem.201604401.

[225] Yongli Duan et al. “Controlling Isomerization of Photoswitches to Modulate 2D Logic-in-Memory Devices by Organic–Inorganic Interfacial Strategy”. In: *Advanced Science* 10.13 (2023). ISSN: 2198-3844. DOI: 10.1002/advs.202207443. URL: http://dx.doi.org/10.1002/advs.202207443.

[226] Na Li, Zhi-Gang Gu, and Jian Zhang. "Erasable Photopatterning of Stilbene-Based Metal-Organic Framework Films". In: *Small Methods* 7.6 (2023). ISSN: 2366-9608. DOI: 10.1002/smtd.202201231. URL: http://dx.doi.org/10.1002/smtd.202201231.

[227] Yang Wang, Evan L. Runnerstrom, and Delia J. Milliron. "Switchable Materials for Smart Windows". In: *Annual Review of Chemical and Biomolecular Engineering* 7.1 (2016), 283–304. ISSN: 1947-5446. DOI: 10.1146/annurev-chembioeng-080615-034647. URL: http://dx.doi.org/10.1146/annurev-chembioeng-080615-034647.

[228] Ryota Sakamoto et al. "A photofunctional bottom-up bis(dipyrrinato)zinc(II) complex nanosheet". In: *Nature Communications* 6.1 (2015). ISSN: 2041-1723. DOI: 10.1038/ncomms7713. URL: http://dx.doi.org/10.1038/ncomms7713.

[229] Ran Wang et al. "Self-Assembled Black Phosphorus-Based Composite Langmuir–Blodgett Films with an Enhanced Photocurrent Generation Capability and Surface-Enhanced Raman Scattering Properties". In: *ACS Omega* 6.6 (2021), 4430–4439. ISSN: 2470-1343. DOI: 10.1021/acsomega.0c05832. URL: http://dx.doi.org/10.1021/acsomega.0c05832.

[230] Jian Mao et al. "Langmuir–Blodgett fabrication of large-area black phosphorus-C60 thin films and heterojunction photodetectors". In: *Nanoscale* 12.38 (2020), 19814–19823. ISSN: 2040-3372. DOI: 10.1039/d0nr04537c. URL: http://dx.doi.org/10.1039/d0nr04537c.

[231] Katsuhiko Ariga, Yuri Lvov, and Gero Decher. "There is still plenty of room for layer-by-layer assembly for constructing nanoarchitectonics-based materials and devices". In: *Physical Chemistry Chemical Physics* 24.7 (2022), 4097–4115. ISSN: 1463-9084. DOI: 10.1039/d1cp04669a. URL: http://dx.doi.org/10.1039/d1cp04669a.

[232] Shujuan Huang et al. "Experimental conditions for a highly ordered monolayer of gold nanoparticles fabricated by the Langmuir–Blodgett method". In: *Journal of Vacuum Science amp; Technology B: Microelectronics and Nanometer Structures Processing, Measurement, and Phenomena* 19.6 (2001), 2045–2049. ISSN: 1520-8567. DOI: 10.1116/1.1410943. URL: http://dx.doi.org/10.1116/1.1410943.

[233] Luzhu Xu et al. "Continuous Langmuir–Blodgett Deposition and Transfer by Controlled Edge-to-Edge Assembly of Floating 2D Materials". In: *Langmuir* 35.1 (2018), 51–59. ISSN: 1520-5827. DOI: 10.1021/acs.langmuir.8b03173. URL: http://dx.doi.org/10.1021/acs.langmuir.8b03173.

[234] Cristina Stefaniu, Gerald Brezesinski, and Helmuth Möhwald. "Langmuir monolayers as models to study processes at membrane surfaces". In: *Advances in Colloid and Interface Science* 208 (2014), 197–213. ISSN: 0001-8686. DOI: 10.1016/j.cis.2014.02.013. URL: http://dx.doi.org/10.1016/j.cis.2014.02.013.

[235] Thatyane M. Nobre et al. "Interactions of bioactive molecules amp; nanomaterials with Langmuir monolayers as cell membrane models". In: *Thin Solid Films* 593 (2015), 158–188. ISSN: 0040-6090. DOI: 10.1016/j.tsf.2015.09.047. URL: http://dx.doi.org/10.1016/j.tsf.2015.09.047.

[236] André C. Machado and Luciano Caseli. "Interaction of nitrofurantoin with lipid langmuir monolayers as cellular membrane models distinguished with tensiometry and infrared spectroscopy". In: *Colloids and Surfaces B: Biointerfaces* 188 (2020), p. 110794. ISSN: 0927-7765. DOI: 10.1016/j.colsurfb.2020.110794. URL: http://dx.doi.org/10.1016/j.colsurfb.2020.110794.

[237] Thiers M. Uehara et al. "Nanostructured scaffolds containing graphene oxide for nanomedicine applications". In: *Polymers for Advanced Technologies* 33.2 (2021), 591–600. ISSN: 1099-1581. DOI: 10.1002/pat.5541. URL: http://dx.doi.org/10.1002/pat.5541.

[238] Hee-Sung Jeong et al. "Oriented layered assemblies of graphene nanosheets/Fe3O4 nanoparticles as a superior anode material for lithium ion batteries". In: *Applied Surface Science* 508 (2020), p. 144416. ISSN: 0169-4332. DOI: 10.1016/j.apsusc.2019.144416. URL: http://dx.doi.org/10.1016/j.apsusc.2019.144416.

[239] Wonsik Eom et al. "Graphene-Mimicking 2D Porous Co3O4 Nanofoils for Lithium Battery Applications". In: *Advanced Functional Materials* 26.42 (2016), 7605–7613. ISSN: 1616-3028. DOI: 10.1002/adfm.201602320. URL: http://dx.doi.org/10.1002/adfm.201602320.

[240] Xin-Bing Cheng et al. "A Review of Solid Electrolyte Interphases on Lithium Metal Anode". In: *Advanced Science* 3.3 (2015). ISSN: 2198-3844. DOI: 10.1002/advs.201500213. URL: http://dx.doi.org/10.1002/advs.201500213.

[241] Junhyeok Kim et al. "Controllable Solid Electrolyte Interphase in Nickel-Rich Cathodes by an Electrochemical Rearrangement for Stable Lithium-Ion Batteries". In: *Advanced Materials* 30.5 (2017). ISSN: 1521-4095. DOI: 10.1002/adma.201704309. URL: http://dx.doi.org/10.1002/adma.201704309.

[242] Di Zhang et al. "Horizontal Growth of Lithium on Parallelly Aligned MXene Layers towards Dendrite-Free Metallic Lithium Anodes". In: *Advanced Materials* 31.33 (2019). ISSN: 1521-4095. DOI: 10.1002/adma.201901820. URL: http://dx.doi.org/10.1002/adma.201901820.

[243] Vengadesh Periasamy et al. "Langmuir–Blodgett Graphene-Based Films for Algal Biophotovoltaic Fuel Cells". In: *Nanomaterials* 12.5 (2022), p. 840. ISSN: 2079-4991. DOI: 10.3390/nano12050840. URL: http://dx.doi.org/10.3390/nano12050840.

[244] Virender et al. "Recent Breakthroughs and Future Potential of Inorganic Nanocomposites in Hydrogen Generation". In: *ChemistrySelect* 9.25 (2024). ISSN: 2365-6549. DOI: 10.1002/slct.202301571. URL: http://dx.doi.org/10.1002/slct.202301571.

[245] Ajay Kumar et al. "Frontier nanoarchitectonics of graphitic carbon nitride based plasmonic photocatalysts and photoelectrocatalysts for energy, environment and organic reactions". In: *Materials Chemistry Frontiers* 7.7 (2023), 1197–1247. ISSN: 2052-1537. DOI: 10.1039/d2qm01064j. URL: http://dx.doi.org/10.1039/d2qm01064j.

[246] Omar Azzaroni et al. "Field-effect transistors engineered via solution-based layer-by-layer nanoarchitectonics". In: *Nanotechnology* 34.47 (2023), p. 472001. ISSN: 1361-6528. DOI: 10.1088/1361-6528/acef26. URL: http://dx.doi.org/10.1088/1361-6528/acef26.

[247] Na Li et al. "Gas-Responsive and Self-Powered Visual Composite Langmuir–Blodgett Films for Ultrathin Gas Sensors". In: *Langmuir* 38.21 (2022), 6761–6770. ISSN: 1520-5827. DOI: 10.1021/acs.langmuir.2c00835. URL: http://dx.doi.org/10.1021/acs.langmuir.2c00835.

[248] Miguel A. Andrés et al. "Methanol and Humidity Capacitive Sensors Based on Thin Films of MOF Nanoparticles". In: *ACS Applied Materials amp; Interfaces* 12.3 (2020), 4155–4162. ISSN: 1944-8252. DOI: 10.1021/acsami.9b20763. URL: http://dx.doi.org/10.1021/acsami.9b20763.

[249] S Salomon, J Eymery, and E Pauliac-Vaujour. "GaN wire-based Langmuir–Blodgett films for self-powered flexible strain sensors". In: *Nanotechnology* 25.37 (2014), p. 375502. ISSN: 1361-6528. DOI: 10.1088/0957-4484/25/37/375502. URL: http://dx.doi.org/10.1088/0957-4484/25/37/375502.

[250] A V Alaferdov et al. "A wearable, highly stable, strain and bending sensor based on high aspect ratio graphite nanobelts". In: *Nanotechnology* 27.37 (2016), p. 375501. ISSN: 1361-6528. DOI: 10.1088/0957-4484/27/37/375501. URL: http://dx.doi.org/10.1088/0957-4484/27/37/375501.

[251] Jinling Zhang et al. "Highly flexible and stretchable strain sensors based on conductive whisker carbon nanotube films". In: *Carbon* 176 (2021), 139–147. ISSN: 0008-6223. DOI: 10.1016/j.carbon.2021.01.130. URL: http://dx.doi.org/10.1016/j.carbon.2021.01.130.

[252] Katsuhiko Ariga, Jonathan P. Hill, and Qingmin Ji. "Layer-by-layer assembly as a versatile bottom-up nanofabrication technique for exploratory research and realistic application". In: *Physical Chemistry Chemical Physics* 9.19 (2007), p. 2319. ISSN: 1463-9084. DOI: 10.1039/b700410a. URL: http://dx.doi.org/10.1039/b700410a.

[253] Jongwook Kim et al. "Shape-Changing DNA-Linked Nanoparticle Films Dictated by Lateral and Vertical Patterns". In: *Advanced Materials* 34.13 (2022). ISSN: 1521-4095. DOI: 10.1002/adma.202109091. URL: http://dx.doi.org/10.1002/adma.202109091.

[254] KATSUMI YONEDA. "The Latest LB Film Deposition Apparatus". In: *Sen'i Gakkaishi* 46.8 (1990), P340–P347. ISSN: 1884-2259. DOI: 10.2115/fiber.46.8_p340. URL: http://dx.doi.org/10.2115/fiber.46.8_p340.

[255] Tianshui Zheng et al. "Stability of Langmuir-Blodgett film/alumina, and Langmuir-Blodgett film/gold multilayer structures". In: *Thin Solid Films* 197.1–2 (1991), 327–333. ISSN: 0040-6090. DOI: 10.1016/0040-

6090(91)90243-q. URL: http://dx.doi.org/10.1016/0040-6090(91)90243-q.

[256] Cristina Stefaniu and Gerald Brezesinski. "X-ray investigation of monolayers formed at the soft air/water interface". In: *Current Opinion in Colloid amp; Interface Science* 19.3 (2014), 216–227. ISSN: 1359-0294. DOI: 10.1016/j.cocis.2014.01.004. URL: http://dx.doi.org/10.1016/j.cocis.2014.01.004.

[257] James P. Bareman and Michael L. Klein. "Molecular Dynamics Simulation of A Langmuir Monolayer". In: *MRS Proceedings* 237 (1991). ISSN: 1946-4274. DOI: 10.1557/proc-237-271. URL: http://dx.doi.org/10.1557/proc-237-271.

[258] C. Medina-Plaza, J.A. de Saja, and M.L. Rodriguez-Mendez. "Bioelectronic tongue based on lipidic nanostructured layers containing phenol oxidases and lutetium bisphthalocyanine for the analysis of grapes". In: *Biosensors and Bioelectronics* 57 (2014), 276–283. ISSN: 0956-5663. DOI: 10.1016/j.bios.2014.02.023. URL: http://dx.doi.org/10.1016/j.bios.2014.02.023.

[259] Xin Su et al. "Silicon-Based Nanomaterials for Lithium-Ion Batteries: A Review". In: *Advanced Energy Materials* 4.1 (2013). ISSN: 1614-6840. DOI: 10.1002/aenm.201300882. URL: http://dx.doi.org/10.1002/aenm.201300882.

[260] Katsuhiko Ariga. "Interfaces Working for Biology: Solving Biological Mysteries and Opening Up Future Nanoarchitectonics". In: *ChemNanoMat* 2.5 (2016), 333–343. ISSN: 2199-692X. DOI: 10.1002/cnma.201600053. URL: http://dx.doi.org/10.1002/cnma.201600053.

[261] Chiara Ausilio et al. "Concealing Organic Neuromorphic Devices with Neuronal-Inspired Supported Lipid Bilayers". In: *Advanced Science* 11.27 (2024). ISSN: 2198-3844. DOI: 10.1002/advs.202305860. URL: http://dx.doi.org/10.1002/advs.202305860.

[262] Lucía Herrer, Santiago Martín, and Pilar Cea. "Nanofabrication Techniques in Large-Area Molecular Electronic Devices". In: *Applied Sciences* 10.17 (2020), p. 6064. ISSN: 2076-3417. DOI: 10.3390/app10176064. URL: http://dx.doi.org/10.3390/app10176064.

[263] Katsuhiko Ariga et al. "Supramolecular Chiral Nanoarchitectonics". In: *Advanced Materials* 32.41 (2020). ISSN: 1521-4095. DOI: 10.1002/adma.201905657. URL: http://dx.doi.org/10.1002/adma.201905657.

[264] Claudine Fouquey, Jean-Marie Lehn, and Anne-Marie Levelut. "Molecular recognition directed self-assembly of supramolecular liquid crystalline polymers from complementary chiral components". In: *Advanced Materials* 2.5 (1990), 254–257. ISSN: 1521-4095. DOI: 10.1002/adma.19900020506. URL: http://dx.doi.org/10.1002/adma.19900020506.

[265] Osvaldo N. Oliveira, Luciano Caseli, and Katsuhiko Ariga. "The Past and the Future of Langmuir and Langmuir–Blodgett Films". In: *Chemical Reviews* 122.6 (2022), 6459–6513. ISSN: 1520-6890. DOI: 10.1021/acs.chemrev.1c00754. URL: http://dx.doi.org/10.1021/acs.chemrev.1c00754.

[266] Katsuhiko Ariga. "Chemistry of Materials Nanoarchitectonics for Two-Dimensional Films: Langmuir–Blodgett, Layer-by-Layer Assembly, and Newcomers". In: *Chemistry of Materials* 35.14 (2023), pp. 5233–5254. DOI: 10.1021/acs.chemmater.3c01291. eprint: https://doi.org/10.1021/acs.chemmater.3c01291. URL: https://doi.org/10.1021/acs.chemmater.3c01291.

[267] Andrey V. Arakcheev et al. "X-Ray induced redox-isomeric transformations of lanthanide bis-phthalocyaninates at the air-water interface". In: *Surfaces and Interfaces* 56 (2025), p. 105682. ISSN: 2468-0230. DOI: https://doi.org/10.1016/j.surfin.2024.105682. URL: https://www.sciencedirect.com/science/article/pii/S2468023024018376.

[268] Tomohiro Murata et al. "Nanometer-Flat DNA-Featured Thin Films Prepared via Laser Molecular Beam Deposition under High-Vacuum for Selective Methanol Sensing". In: *Bulletin of the Chemical Society of Japan* 96.1 (2022), 29–34. ISSN: 1348-0634. DOI: 10.1246/bcsj.20220303. URL: http://dx.doi.org/10.1246/bcsj.20220303.

[269] Jingwen Song et al. "Fullerphene Nanosheets: A Bottom-Up 2D Material for Single-Carbon-Atom-Level Molecular Discrimination". In: *Advanced Materials Interfaces* 9.11 (2022). ISSN: 2196-7350. DOI: 10.

1002/admi.202102241. URL: http://dx.doi.org/10.1002/admi.202102241.

[270] Ruibo Lu et al. “AFM investigation of polymer LB films on the alignment of ferroelectric liquid crystal”. In: *Physics Letters A* 260.5 (1999), 417–423. ISSN: 0375-9601. DOI: 10.1016/s0375-9601(99)00522-8. URL: http://dx.doi.org/10.1016/s0375-9601(99)00522-8.

[271] Theyencheri Narayanan and Oleg Konovalov. “Synchrotron Scattering Methods for Nanomaterials and Soft Matter Research”. In: *Materials* 13.3 (2020), p. 752. ISSN: 1996-1944. DOI: 10.3390/ma13030752. URL: http://dx.doi.org/10.3390/ma13030752.

[272] Antonio Riul et al. “Nano-Assembled Films for Taste Sensor Application”. In: *Artificial Organs* 27.5 (2003), 469–472. ISSN: 1525-1594. DOI: 10.1046/j.1525-1594.2003.07243.x. URL: http://dx.doi.org/10.1046/j.1525-1594.2003.07243.x.

[273] Fábio A. Scholl et al. “Exploring Langmuir-Blodgett films with phospholipid-graphene oxide/MnO2 as a hybrid nanostructured interface for supercapacitor applications”. In: *Colloids and Surfaces A: Physicochemical and Engineering Aspects* 664 (2023), p. 131128. ISSN: 0927-7757. DOI: 10.1016/j.colsurfa.2023.131128. URL: http://dx.doi.org/10.1016/j.colsurfa.2023.131128.

S8 Third Paper generated using Strategy 1 (20 input papers)

Langmuir and Langmuir-Blodgett Films: A Comprehensive Survey of Principles, Fabrication, Characterization, and Applications

April 23, 2025

Contents

1 Introduction **2**

2 Fundamentals of Langmuir and Langmuir-Blodgett Films **6**
2.1 Langmuir Monolayers 7
2.2 Langmuir-Blodgett Transfer 9
2.3 Molecular Organization and Orientation 13

3 Materials for Langmuir and Langmuir-Blodgett Films **15**
3.1 Amphiphilic Molecules 16
3.2 Nanomaterials 19
3.3 Emerging Materials 21

4 Fabrication Techniques and Parameters **23**
4.1 Langmuir Trough Design and Operation 24
4.2 Spreading Techniques 27
4.3 Compression and Transfer Parameters 28

5 Characterization Techniques for Langmuir and Langmuir-Blodgett Films **31**
5.1 In-situ Characterization at the Air-Water Interface 32

5.1.1 Surface Pressure-Area (π-A) Isotherms 32
5.1.2 Optical Microscopy Techniques 33
5.2 Ex-situ Characterization of Langmuir-Blodgett Films 34
5.2.1 Microscopic Techniques 34
5.2.2 Spectroscopic Techniques 35
5.2.3 X-ray Scattering Techniques 35
5.2.4 Other Techniques 36
5.3 Molecular Orientation 36

6 Applications of Langmuir and Langmuir-Blodgett Films 37
6.1 Sensing Applications 39

7 Challenges and Future Directions 40

8 Conclusion 44

Abstract

Langmuir and Langmuir-Blodgett (LB) technologies provide unparalleled control over the fabrication of ultrathin films with molecular-level precision, enabling the construction of highly organized mono- and multilayer structures. This survey comprehensively reviews the principles, advancements, and applications of Langmuir and LB films. It begins with the fundamentals of forming Langmuir monolayers via molecular self-assembly at fluid interfaces and their subsequent transfer onto solid substrates using LB or Langmuir-Schaefer methods. The review highlights the remarkable material versatility of the technique, extending beyond classical amphiphiles to include polymers, nanoparticles, biomolecules, carbon nanomaterials, metal-organic frameworks, and complex hybrid systems. Critical aspects of fabrication control, including the influence of experimental parameters (subphase composition, surface pressure, temperature, deposition mechanics) on film quality, are discussed alongside essential in-situ and ex-situ characterization techniques (surface pressure-area isotherms, microscopy, spectroscopy, diffraction/reflectivity) used to elucidate film structure, morphology, and properties. Furthermore, the survey explores the broad spectrum of applications in areas such as sensing, electrochemistry, biomimetics, optics, tribology, and nanotechnology. Finally, current challenges related to film stability, defect density, scalability, and reproducibility are addressed, concluding with future perspectives on

the enduring role of LB technology in advancing molecular engineering and nanoarchitectonics.

1 Introduction

Langmuir and Langmuir-Blodgett (LB) film technologies represent cornerstone methodologies in surface science and nanotechnology, enabling the fabrication of highly organized, ultra-thin films with molecular-level precision. Originating from the pioneering studies on surface tension and molecular behaviour at interfaces by Agnes Pockels, Irving Langmuir, and Katharine Blodgett nearly a century ago, these techniques have evolved significantly, finding applications across a diverse spectrum of scientific and technological domains. A Langmuir film is essentially a monomolecular layer formed by amphiphilic molecules self-assembling at a gas-liquid interface, typically air-water. By carefully controlling parameters such as surface pressure, temperature, and subphase composition, these molecules can be organized into specific two-dimensional structures. Figure 1 schematically illustrates this fundamental process, showing the formation of a dense monolayer of amphiphilic molecules at the air-liquid interface upon barrier compression (Figure 1a) and the subsequent transfer of this monolayer onto a solid substrate via vertical or horizontal deposition (Figure 1b). The subsequent transfer of these ordered Langmuir films onto solid substrates results in Langmuir-Blodgett films, which can range from single monolayers to complex multilayer assemblies with precisely defined architectures.

The significance of LB technology stems from its unparalleled ability to control the structure and thickness of thin films down to the nanometer scale [1, 2, 3]. Unlike many other thin-film deposition techniques, the LB method allows for the deliberate arrangement of molecules, offering control over orientation and packing density. This precise structural control is crucial for tailoring the physical and chemical properties of the resulting films. LB films typically exhibit highly anisotropic lamellar structures and can, in principle, achieve monomolecular layers with minimal defects, making them ideal platforms for fundamental studies of interfacial phenomena and for the development of functional materials and devices [1, 4, 3]. The versatility of the technique is further underscored by the wide range of materials that can be assembled into LB films, including traditional amphiphiles like fatty acids and lipids [3], as well as polymers, nanoparticles [5], proteins, DNA

[6], graphene oxide (GO) [7, 8, 9], metal-organic frameworks (MOFs) [10], and other nanomaterials. Figure 2 provides a specific example of this versatility, illustrating how LB deposition of silica nanospheres can be integrated into a nanofabrication process to create vertical nanochannels in a polymer film. Furthermore, Figure 3 exemplifies the assembly of advanced nanomaterial composites, specifically single-walled carbon nanotubes (SWNTs) and graphene nanostructures (GNS), using the LB technique (Figure 3a,b) and shows typical characterization data in the form of a surface pressure-area (π-A) isotherm (Figure 3c). This material flexibility allows for the creation of hybrid and composite films with tailored functionalities, positioning LB technology as an indispensable tool in molecular engineering and nanoarchitectonics – the deliberate construction of functional materials from nanoscale components [11, 12, 13, 14].

Despite their numerous advantages, the fabrication and application of LB films are not without challenges. Achieving large-area, defect-free films remains a significant hurdle, often limited by impurities, environmental factors like dust and vibrations, and the inherent fragility of molecular monolayers [15]. The long-term stability of LB films, both mechanical and chemical, can also be a concern, particularly under harsh operating conditions or prolonged storage [16, 17]. Controlling the precise molecular orientation within the film, especially for complex or non-amphiphilic molecules, requires careful optimization of deposition parameters and can be difficult to achieve consistently. Furthermore, scaling up the LB process for industrial manufacturing faces hurdles regarding reproducibility, throughput, and cost-effectiveness, despite the technique's inherent compatibility with large-area substrates [18]. Substrate compatibility and the influence of the substrate surface on film structure and properties also require careful consideration during the design and fabrication process. Addressing these limitations is crucial for the broader adoption and practical implementation of LB film technology.

This survey aims to provide a comprehensive overview of the current state of Langmuir and Langmuir-Blodgett film research. It delves into the fundamental principles governing the formation and behaviour of these films, explores the diverse range of materials employed, details the various fabrication techniques and critical parameters influencing film quality, and discusses the advanced characterization methods used to probe their structure and properties. Furthermore, it highlights the burgeoning applications of LB films in diverse fields such as sensors, electronics, electrochemistry, energy storage,

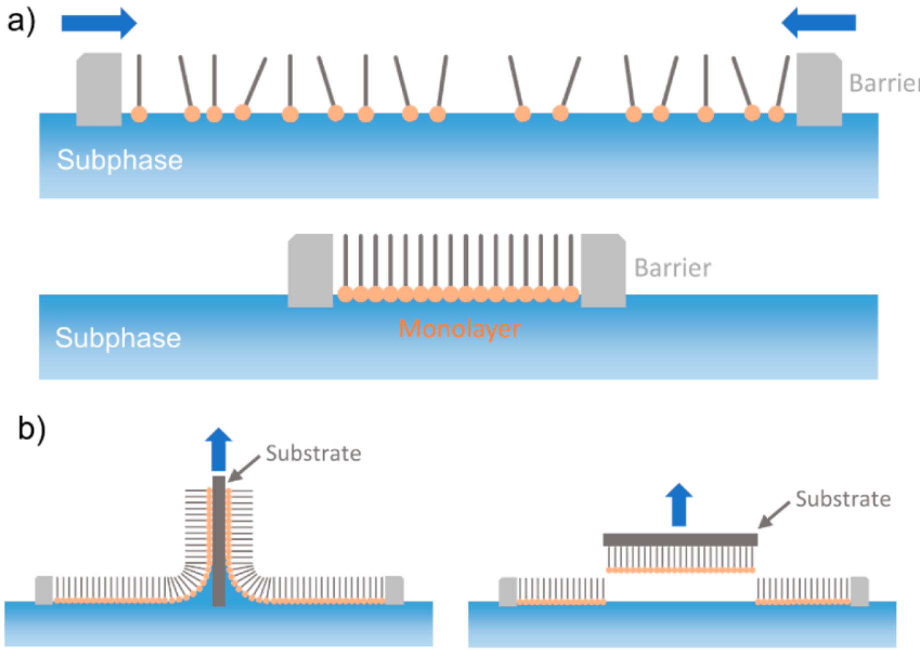

Figure 1: Schematic illustration of basic principles of Langmuir-Blodgett-type methods. (a) Formation of dense monolayer at air-liquid interface (commonly using water as subphase) with amphiphilic molecules (orange, hydrophilic head; gray, hydrophobic tail) upon barrier compression. (b) Transfer of monolayer from liquid surface to substrate through vertical (left) or horizontal deposition (right). Adapted from Fang, C., et al., 2022 [19].

optics, tribology, and biomimetic systems.

Finally, the survey will address the persistent challenges and discuss promising future directions for research and development in this dynamic field.

The structure of this survey is organized as follows: Section 2 will elaborate on the fundamental principles underlying Langmuir monolayer formation at the air-water interface, including thermodynamics, phase behaviour, and surface pressure-area isotherms, as well as the mechanics of Langmuir-Blodgett transfer [20]. Section 3 will provide an overview of the various classes of materials suitable for LB film fabrication, discussing their molecular design requirements and properties [21]. Section 4 will detail the experimental techniques for LB film preparation, including trough design, deposition

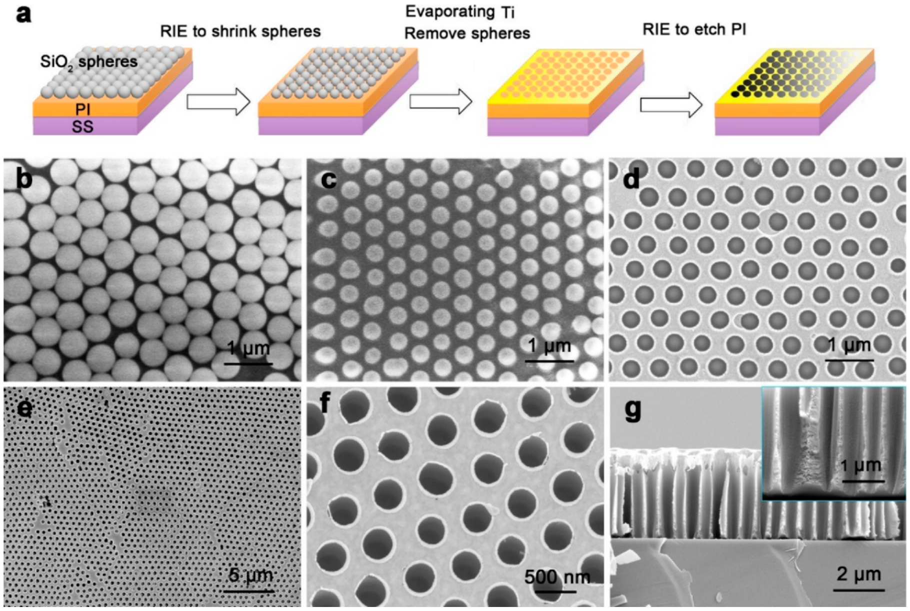

Figure 2: Schematic presentation of the fabrication process of vertical channels in a PI film. Silica nanospheres on a PI film (b) after LB deposition and (c) after CHF_3-based RIE shrinking treatment. (d) PI film with Ti mask after removal of silica nanospheres. (e-g) Vertical nanochannels after etching of Ti-masked PI film (e,f) top view and (g) cross sectional view. Adapted from Fang, C., et al., 2022 [19].

methods (vertical dipping, horizontal lifting/Langmuir-Schaefer), and the influence of key experimental parameters [22]. Section 5 will cover the essential characterization techniques used to analyze the structure, morphology, thickness, and properties of Langmuir and LB films [23, 24, 25]. Section 6 will showcase the diverse applications of LB films, drawing examples from recent research in sensing, electronics, energy, and biomedical fields [2, 26]. Section 7 will critically evaluate the current challenges facing LB technology and explore potential future research avenues and technological advancements. Finally, Section 8 will offer concluding remarks, summarizing the key aspects discussed throughout the survey.

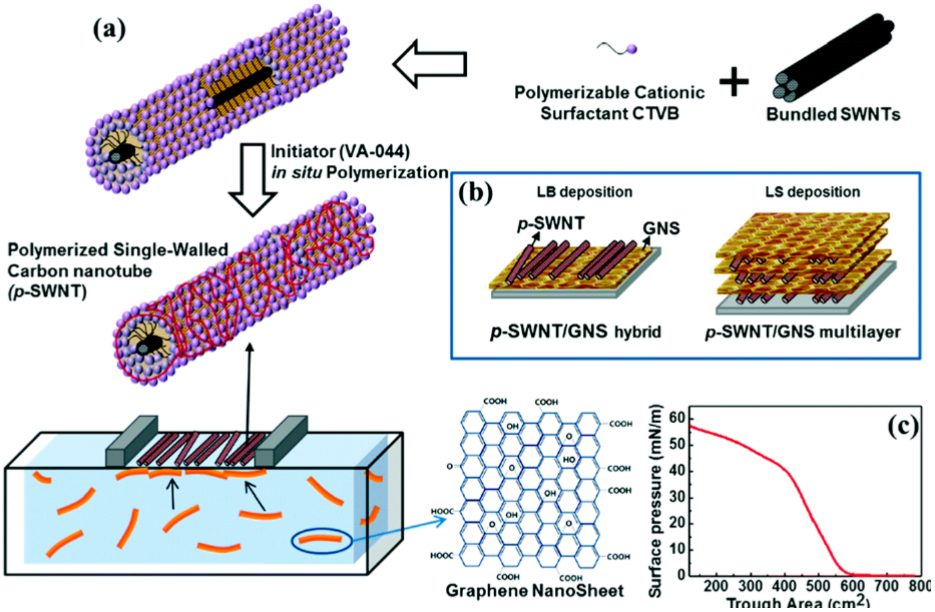

Figure 3: Schematic demonstration of fabrication principle of p-SWNT/GNS monolayer at the water surface. (b) Preparation of layered SWNT/GNS composite via repeated deposition of single layers. (c) Surface pressure-area (π-A) isotherm profile of p-SWNTs. Adapted from Fang, C., et al., 2022 [19].

2 Fundamentals of Langmuir and Langmuir-Blodgett Films

The Langmuir-Blodgett (LB) technique is a sophisticated method for fabricating highly ordered thin films with molecular-level precision, building upon the fundamental principles of interfacial science. The process begins with the formation of a Langmuir monolayer at a gas-liquid interface, typically air-water, followed by the controlled transfer of this monolayer onto a solid substrate. Understanding the behavior of molecules at this interface and the mechanics of the transfer process is crucial for controlling the structure and properties of the final LB film.

2.1 Langmuir Monolayers

The formation of Langmuir monolayers relies on the unique properties of amphiphilic molecules. These molecules possess distinct hydrophilic (water-attracting) and hydrophobic (water-repelling) parts. A classic example is a fatty acid, which has a polar carboxylic acid head group (hydrophilic) and a long, nonpolar hydrocarbon tail (hydrophobic). When a dilute solution of such molecules in a volatile, water-immiscible solvent (like chloroform) is carefully spread onto the surface of an aqueous subphase contained within a Langmuir trough, the solvent evaporates, leaving the amphiphilic molecules at the air-water interface. Due to their dual nature, these molecules spontaneously orient themselves: the hydrophilic head groups immerse themselves in the water subphase, while the hydrophobic tails extend upwards into the air, minimizing unfavorable interactions with the water. Initially, at large surface areas, the molecules are far apart and behave like a two-dimensional gas, moving relatively freely across the interface with minimal intermolecular interaction.

The Langmuir trough is the essential apparatus for studying and manipulating these monolayers [27, 28]. It typically consists of a shallow trough made of an inert material (like PTFE), equipped with one or more movable barriers that span the width of the trough and rest on its edges. These barriers allow for the controlled compression or expansion of the monolayer, effectively changing the surface area available to the amphiphilic molecules. A key parameter characterizing the state of the monolayer is the surface pressure (). Surface pressure is defined as the reduction in surface tension of the pure subphase () caused by the presence of the monolayer (): = - . It represents the two-dimensional analogue of pressure in a three-dimensional system and quantifies the repulsive forces between the molecules confined at the interface. Surface pressure is typically measured using a Wilhelmy plate method [29], where a plate (often made of platinum or filter paper) is partially immersed in the subphase and connected to a sensitive microbalance. The force exerted on the plate due to surface tension is measured, and changes in this force upon monolayer compression directly relate to the surface pressure.

As the barriers are slowly moved to compress the monolayer, the area per molecule decreases, forcing the molecules closer together and increasing the surface pressure. The relationship between surface pressure () and the area per molecule (A) at a constant temperature is represented by the sur-

face pressure-area (-A) isotherm, as exemplified in Figure 6. This isotherm provides critical information about the phase behavior and packing characteristics of the monolayer [30, 31]. Several distinct phases can typically be identified as the monolayer is compressed:

- **Gaseous (G) Phase:** At very large areas per molecule, the surface pressure is near zero. The molecules are far apart, with negligible interactions, behaving like a 2D gas.

- **Liquid-Expanded (LE) Phase:** As the area decreases, the surface pressure begins to rise. Molecules come into closer contact, exhibiting increased intermolecular interactions but still possessing significant conformational freedom and mobility. The hydrophobic tails may not be fully ordered or vertically oriented.

- **Liquid-Condensed (LC) Phase:** Further compression leads to a steeper increase in surface pressure. The molecules are more closely packed, and the hydrophobic tails become more ordered and oriented more vertically. The monolayer exhibits reduced compressibility compared to the LE phase. There might be coexistence regions (e.g., LE-LC transition) characterized by a plateau or change in slope in the isotherm.

- **Solid (S) Phase:** At even smaller areas per molecule, the monolayer reaches its most densely packed state. The molecules are highly ordered, often forming a 2D crystalline or quasi-crystalline structure. The isotherm becomes very steep, indicating extremely low compressibility. The molecules are typically oriented nearly perpendicular to the interface.

- **Collapse Phase:** If compression continues beyond the solid phase, the monolayer structure becomes unstable and collapses [32]. The 2D structure breaks down, and molecules are forced out of the monolayer into three-dimensional aggregates, bilayers, micelles, or the film may undergo buckling and folding. This is often observed as a plateau or decrease in surface pressure at a high compression state, indicating the disruption of the uniform monolayer integrity.

The specific phases observed, the transition pressures, and the limiting area per molecule (area extrapolated to zero pressure from the steepest part of

the isotherm) are characteristic properties of the amphiphile, subphase composition (pH, ionic strength), and temperature. The compressional modulus (), also known as the surface elasticity, provides a measure of the monolayer's rigidity or resistance to compression in different phases. It is calculated from the slope of the -A isotherm using the formula = -A (d/dA). The inset in Figure 6 shows an example of how the compressional modulus changes with surface pressure. Typically, is low in the G and LE phases, increases significantly in the LC phase, and reaches its highest values in the S phase, indicating increasing rigidity and lower compressibility. For instance, values might range from ¡12.5 mN/m (G/LE) to 100-250 mN/m (LC) and ¿250 mN/m (S) for typical amphiphiles. This modulus is a valuable tool for identifying phase transitions. However, its effectiveness can vary; while useful for 'hard' materials showing distinct phase changes, it may be less informative for 'soft' materials like graphene oxide, where phase transitions might be less pronounced in the profile [33, 8]. Understanding these phases and their mechanical properties via the -A isotherm and compressional modulus is crucial for selecting the appropriate conditions, particularly the target surface pressure, for subsequent transfer onto a solid substrate.

2.2 Langmuir-Blodgett Transfer

Once a stable Langmuir monolayer has been formed and compressed to a desired surface pressure (typically within the LC or S phase where the film is coherent and well-ordered), it can be transferred onto a solid substrate to create a Langmuir-Blodgett (LB) film. This transfer process allows the precise architecture built at the air-water interface to be captured in a solid-state thin film. The quality and structure of the resulting LB film depend heavily on the transfer method and the parameters employed.

Two primary methods are used for transferring Langmuir monolayers:

- **Vertical Dipping (Langmuir-Blodgett Method):** This is the classic LB technique [1, 2]. A solid substrate is vertically immersed into the subphase through the monolayer and then withdrawn back through the interface. During this process, the Langmuir trough's barriers are typically moved inwards automatically to maintain a constant surface pressure, compensating for the molecules being transferred. The nature of the transfer depends on the substrate's surface properties (hydrophilic or hydrophobic) and the interactions between the substrate,

the amphiphile's head groups, and the subphase [34]. Depending on these interactions and whether transfer occurs primarily on the upstroke, downstroke, or both, different multilayer structures (commonly denoted Y, X, or Z-type) can be formed, as detailed in Section 2.3.

- **Horizontal Lifting (Langmuir-Schaefer Method):** In this method, the substrate is brought into contact with the monolayer horizontally. The substrate touches the monolayer surface, and the film adheres to it. The substrate is then lifted away. This method typically transfers only one layer per contact, regardless of the substrate's hydrophilicity. It is often preferred for depositing rigid monolayers or materials that might be disrupted by the meniscus forces during vertical dipping. It can also be useful for creating non-centrosymmetric film structures or depositing onto pre-structured substrates.

Table 1 summarizes the key differences between the Langmuir-Blodgett (vertical) and Langmuir-Schaefer (horizontal) techniques [22, 35].

Several factors critically influence the success and quality of the transfer process:

- **Transfer Surface Pressure:** The surface pressure must be maintained constant during deposition. It is typically chosen within the condensed phase (LC or S) to ensure a stable, coherent film. The chosen pressure affects the packing density, molecular orientation, and morphology of the transferred film. Studies using techniques like Atomic Force Microscopy (AFM) have demonstrated that varying the transfer pressure can significantly alter the surface structure, roughness, and homogeneity of the resulting LB films, as observed for materials like P3HT/stearic acid blends.

- **Substrate Properties:** The substrate must be meticulously cleaned to ensure proper adhesion. Its surface energy (hydrophilicity/hydrophobicity) dictates the interaction with the amphiphilic molecules and influences the type of deposition (Y, X, or Z in vertical transfer). Surface roughness can also affect film uniformity [36].

- **Dipping/Lifting Speed:** The speed at which the substrate moves through or contacts the interface must be carefully controlled. If the speed is too high, the monolayer may not have sufficient time to rearrange and adhere uniformly, leading to defects or incomplete transfer.

Table 1: Comparison between Langmuir-Blodgett (LB) and Langmuir-Schaefer (LS) Transfer Techniques.

**Feature**	**Langmuir-Blodgett (LB)**	**Langmuir-Schaefer (LS)**
Principle	Vertical transfer via dipping/withdrawal	Horizontal transfer via surface contact
Transfer Process	Substrate moves vertically through interface	Substrate touches interface horizontally
Film Structure Control	Precise multilayer control (Y, X, Z types possible)	Primarily monolayer transfer per step; multilayer buildup possible but less common for complex structures
Application Fields	Electronics, sensors, optics, complex structures	Surface modification, biofilm modeling, simpler structures, rigid films
Advantages	High precision, complex multilayer structures, good control over thickness and orientation	Simpler operation, suitable for fragile/rigid monolayers, less dependent on substrate hydrophilicity for transfer
Disadvantages	Sensitive to vibrations, substrate properties, dipping speed; potential for meniscus disruption	Less precise control over multilayer structure, potential for trapping water/air pockets

If it is too slow, the process becomes time-consuming, and monolayer stability over time might become an issue. Optimal speeds depend on the specific molecule, subphase viscosity, and temperature.

- **Subphase Conditions:** Temperature affects molecular mobility and monolayer stability [37, 38]. The pH of the subphase is crucial, especially for ionizable amphiphiles like fatty acids, as it determines the degree of head group ionization, influencing intermolecular electrostatic interactions and interactions with the substrate [39]. Investigations have shown that subphase pH can alter monolayer condensation and the stability of transferred films, impacting properties like sensor response [40]. The presence and type of ions (e.g., divalent cations like Ca^{2} or Cd^{2} versus monovalent ions like Na) in the subphase can significantly alter monolayer properties by binding to head groups, affecting packing, stability, and transfer behavior [39]. For example, studies have demonstrated that Ca^{2} ions can enhance the stability of fatty acid LB films against desorption in aqueous environments compared to Na ions, likely due to stronger binding and cross-linking effects [41].

- **Molecular Concentration and Compression Rate:** The initial concentration of the spreading solution can influence the initial state of the monolayer and potentially the formation of aggregates even before compression. The rate at which the monolayer is compressed to the target pressure also plays a role; very fast compression might trap defects or prevent molecules from reaching their equilibrium packing arrangement, while very slow compression increases experiment time but generally allows for better molecular organization.

The transfer ratio (TR), defined as the ratio of the decrease in monolayer area on the trough to the area of the substrate coated, is often monitored during deposition. A TR close to unity generally indicates successful and complete transfer.

2.3 Molecular Organization and Orientation

A key advantage of the LB technique is the ability to control the molecular organization and orientation within the thin film. This control stems from the self-assembly process at the air-water interface and the ordered transfer onto the substrate. The final structure is determined by a combination

of the intrinsic properties of the amphiphilic molecule and the processing conditions.

The chemical structure of the amphiphile is paramount. The nature of the hydrophilic head group (e.g., -COOH, -OH, -NH, charged groups) and the hydrophobic tail (e.g., length, saturation, branching, presence of aromatic rings) dictates the molecule's packing behavior, stability, and interaction with the subphase and substrate [42, 43]. For instance, longer alkyl chains generally lead to stronger van der Waals interactions and more condensed, stable monolayers at lower temperatures [44]. The size and charge of the head group influence the limiting area per molecule and the electrostatic interactions within the layer and with ions in the subphase [39, 45].

Vertical LB deposition typically leads to specific multilayer structures denoted as X-, Y-, or Z-type, as illustrated schematically in Figure 4. The type of deposition depends on the balance of forces between the substrate, head groups, tail groups, and subphase during the immersion and withdrawal cycles:

- **Y-type films:** This is the most common type, typically formed when transfer occurs during both the upstroke and downstroke, particularly on hydrophilic substrates. Molecules in successive layers are arranged tail-to-tail and head-to-head. This results in a centrosymmetric structure, meaning it possesses a center of inversion symmetry.

- **X-type films:** Transfer occurs predominantly or exclusively during the downstroke (immersion). All layers have the same orientation relative to the substrate, typically with head groups pointing towards the substrate. This results in a non-centrosymmetric structure.

- **Z-type films:** Transfer occurs predominantly or exclusively during the upstroke (withdrawal). All layers again have the same orientation, but typically with tails pointing towards the substrate. This also results in a non-centrosymmetric structure.

The ability to form non-centrosymmetric X- or Z-type films is particularly important for applications requiring properties like pyroelectricity or second-harmonic generation. Langmuir-Schaefer deposition, being a horizontal contact method, inherently transfers layers with the same orientation relative to the substrate surface (e.g., hydrophobic tails exposed if transferred from the air-water interface) [22].

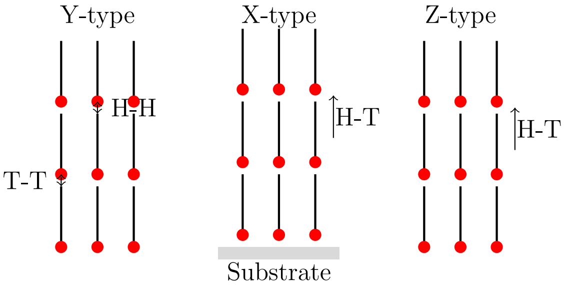

Figure 4: Schematic representation of idealized molecular arrangements in Y-type, X-type, and Z-type Langmuir-Blodgett multilayer films on a solid substrate. Red circles represent hydrophilic head groups, and black lines represent hydrophobic tails. T-T indicates tail-to-tail interaction, H-H indicates head-to-head interaction, and H-T indicates the head-to-tail interface between layers in X and Z types.

The precise orientation of molecules within each layer is governed by a delicate balance of intermolecular forces. Van der Waals interactions between hydrophobic tails promote close packing and ordering, particularly in the condensed phases. Hydrogen bonding can occur between suitable head groups (e.g., -COOH, -OH) or between head groups and water molecules, influencing packing and stability [39]. Electrostatic interactions are significant for charged head groups and can be modulated by subphase pH and the presence of counterions [40, 46]. For molecules containing large planar systems, such as porphyrins or phthalocyanines, - stacking interactions can play a crucial role in determining orientation relative to the interface or substrate [47]. As depicted in Figure 5, these macrocycles can adopt orientations described as 'edge-on' (plane of the macrocycle perpendicular or tilted relative to the substrate) or 'face-on' (plane of the macrocycle parallel to the substrate) [48, 49, 50]. Controlling this orientation is critical for applications in electronics and photonics, as it directly influences properties like charge transport and light absorption [51]. By carefully selecting the molecular building blocks and controlling the deposition parameters (surface pressure, subphase composition, temperature, transfer method), the LB technique allows for the engineering of thin films with tailored molecular architectures, enabling the fine-tuning of their physical and chemical properties for specific applications. The ability to create such ordered structures, layer by layer, with control down to the molecular scale, remains a defining feature and strength of the

Langmuir-Blodgett methodology [1, 21, 2].

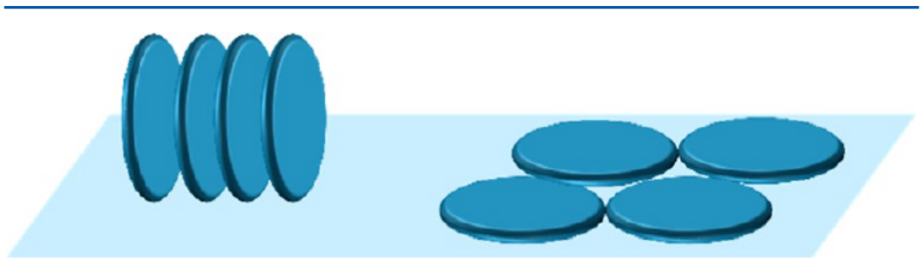

Figure 5: Schematic representation of the edge-on (left) and face-on (right) orientations of a macrocycle at the air—water interface. Adapted from Alinia, Z., et al., 2024 [52].

3 Materials for Langmuir and Langmuir-Blodgett Films

The versatility of the Langmuir-Blodgett (LB) technique stems significantly from the wide array of materials that can be assembled into highly ordered thin films. The fundamental requirement for forming a stable Langmuir film at an air-liquid interface is typically amphiphilicity. However, methods have been developed to handle non-amphiphilic materials as well; common strategies include co-spreading the material with a traditional amphiphile (like a fatty acid, which acts as a matrix), using specialized spreading solvents that facilitate monolayer formation, or modifying the surface of the material (e.g., functionalizing nanoparticles) to impart amphiphilic character. The choice of material is paramount as it dictates the structural characteristics, stability, and ultimate functionality of the resulting LB film. Materials range from classical small organic molecules to complex macromolecules, nanomaterials, and novel framework structures, enabling applications across diverse fields such as electronics, sensing, energy storage, and biomimicry. This section explores the primary categories of materials employed in LB film fabrication, including traditional amphiphiles, incorporated nanomaterials, and emerging material systems.

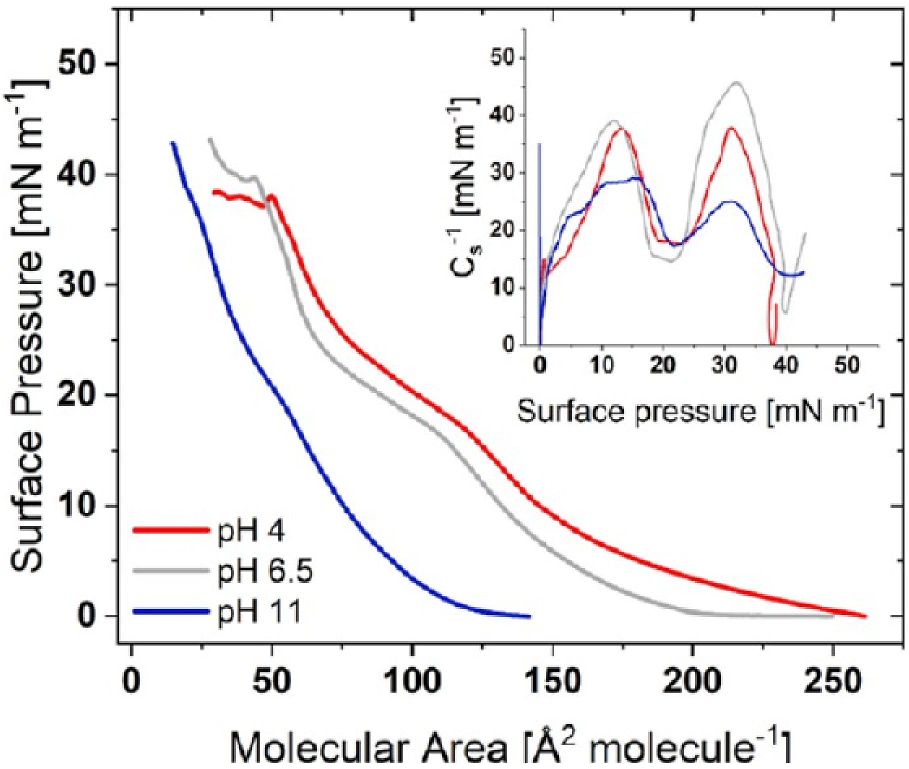

Figure 6: Example of a surface pressure—molecular area (Π-A) isotherm and the corresponding compressional modulus (ε) plot (inset) for an amphiphilic compound at the air-water interface, showing different phases upon compression. Adapted from Alinia, Z., et al., 2024 [52].

3.1 Amphiphilic Molecules

Amphiphilic molecules form the bedrock of Langmuir and Langmuir-Blodgett film technology. Their defining characteristic is a dual chemical nature, possessing both a hydrophilic (water-attracting) head group and a hydrophobic (water-repelling) tail group. When spread onto an aqueous subphase, these molecules spontaneously orient themselves at the air-water interface, minimizing unfavorable interactions by directing their hydrophilic heads towards the water and their hydrophobic tails towards the air. Compression of these molecules on the subphase surface leads to the formation of ordered monomolecular layers, or Langmuir films, exhibiting distinct phase behaviors analogous to three-dimensional matter (gas, liquid-expanded, liquid-

condensed, solid). Figure 7 provides a schematic illustration of this fundamental amphiphilic structure and its orientation at the air-water interface.

Fatty acids are among the earliest and most extensively studied amphiphiles in LB research [3, 53]. They consist of a hydrophilic carboxylic acid (-COOH) head group attached to a long, hydrophobic alkyl chain. Variations in chain length (typically C4 to C28), saturation (presence or absence of double/triple bonds), and head group modifications significantly influence the packing density, phase transition pressures, stability, and mechanical properties of the resulting films. For instance, longer saturated chains generally lead to more condensed and stable films at room temperature due to stronger van der Waals interactions between the tails [54]. Unsaturated fatty acids introduce kinks in the alkyl chains, resulting in less dense packing and more fluid films. Fatty acid LB films have found applications as model biological membranes [55, 56], lubricants, metal binders [39], and structural templates for other materials. The ionization state of the carboxylic head group, controlled by the subphase pH and the presence of specific ions (e.g., divalent cations like Ca^{2+} or Cd^{2+}), can drastically alter intermolecular interactions, leading to the formation of fatty acid salts with different packing arrangements and stability [40, 57].

Lipids, particularly phospholipids like dipalmitoylphosphatidylcholine (DPPC), are another crucial class of amphiphiles, central to biological membrane structure and function. Phospholipids typically feature a hydrophilic phosphate-containing head group and two hydrophobic fatty acid tails. Their behavior at the air-water interface closely mimics that of biological membranes [58, 27, 28], making Langmuir monolayers of lipids invaluable models for studying membrane biophysics [30], protein-lipid interactions [59], drug delivery mechanisms [60], and the effects of various molecules (e.g., cholesterol [61, 56, 62], sterols, anesthetics) on membrane properties. The phase behavior of lipid monolayers is complex, often exhibiting multiple condensed phases with distinct molecular tilts and packing arrangements [63]. LB films derived from lipids are employed in biosensor development and as biocompatible coatings [64].

Surfactants, broadly defined as molecules that lower surface or interfacial tension, encompass a wide range of structures beyond fatty acids and lipids, including cationic, anionic, non-ionic, and zwitterionic types [65]. Their general structure comprises a hydrophilic head and a hydrophobic tail. They are extensively used in LB technology [2, 4, 1], not only as the primary film-forming material but also as dispersing agents or stabilizers to facili-

tate the formation of Langmuir films from non-amphiphilic materials like nanoparticles or polymers [33, 66]. The choice of surfactant influences the surface pressure-area isotherm characteristics and the properties of the final deposited film [67, 68].

Polymers represent a diverse category of materials used for LB film formation. Amphiphilic polymers, containing both hydrophilic and hydrophobic segments, can form stable Langmuir films [69]. Examples include poly(vinylidene fluoride) (PVDF) [70], polyaniline (PANI) [71, 72, 73], and poly(p-phenylene vinylene) (PPV) [74]. Non-amphiphilic polymers can sometimes be processed using the LB technique, often with the aid of surfactants or by spreading from specific solvent systems. Polymer LB films offer advantages like enhanced mechanical stability and diverse functionalities (e.g., PVDF for its piezoelectric properties, PANI for its electrical conductivity [71], PPV for its luminescence). For instance, highly oriented films of semiconducting polymers like poly[2,5-bis(3-tetradecylthiophen-2-yl)thieno(3,2-b)-thiophene] (PBTTT) have been fabricated using high-temperature LB techniques with ethylene glycol subphases, enabling anisotropic charge transport. Mixtures of amphiphilic molecules, such as fatty acids mixed with functional polymers (e.g., PPV) or other lipids, are often employed to create composite films with tailored properties, combining the structural organization provided by the amphiphile matrix with the specific functions of the incorporated molecules [75, 76].

The behavior of complex amphiphilic molecules at the air-water interface can be intricate, influenced by molecular structure, intermolecular interactions, and subphase conditions [77]. Tetraazaporphyrins (TAPs) serve as an example of such complex systems [50, 78]. Figure 8 illustrates proposed molecular conformations for different TAP derivatives at the interface, highlighting how variations in side chains and spacer lengths affect their packing. The study of their Langmuir films often involves detailed analysis of surface pressure-molecular area (π-A) isotherms, which reveal phase transitions and molecular packing changes during compression [79]. Figure 9 shows representative π-A isotherms for a specific TAP derivative (4b) at different subphase pH values. These isotherms, along with the calculated compressibility modulus (inset), demonstrate the significant impact of pH on the film's stability, compressibility, and phase behavior, likely due to changes in the ionization state of the carboxylic acid groups [40, 39]. Visualizing the film morphology directly at the interface provides further insights. Figure 10 presents Brewster Angle Microscopy (BAM) images of the same TAP derivative (4b) at various surface pressures and pH values [80]. These images clearly show

the evolution of the film structure, from gaseous or liquid-expanded phases at low pressures to more condensed and potentially aggregated structures at higher pressures, with distinct morphological differences observed depending on the subphase pH. Such combined analyses are crucial for understanding and controlling the formation of LB films from complex functional molecules.

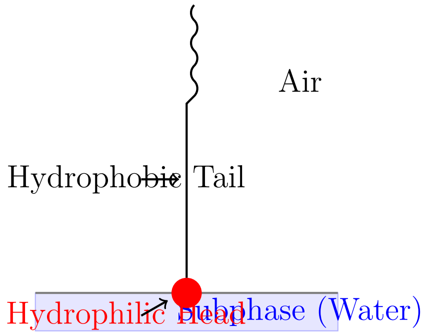

Figure 7: Schematic representation of a generic amphiphilic molecule oriented at the air-water interface. The hydrophilic head interacts favorably with the water subphase, while the hydrophobic tail extends into the air.

3.2 Nanomaterials

The integration of nanomaterials into Langmuir-Blodgett films has opened up new avenues for creating functional thin films with enhanced or entirely novel properties. The LB technique provides a powerful platform for organizing nanoscale objects, such as nanoparticles, nanotubes, nanowires, and nanosheets, into well-defined mono- or multilayer structures with controlled density and orientation, which is often difficult to achieve using other deposition methods like spin-coating or drop-casting.

Various types of nanoparticles, including metallic (e.g., gold (Au), silver (Ag)) [81, 82], semiconductor (e.g., CdS, ZnO), and metal oxide (e.g., TiO_2, SnO_2, MnO_2) [83], have been incorporated into LB films. Often, surface modification of the nanoparticles or the use of auxiliary amphiphilic molecules (like fatty acids or surfactants) is necessary to render them surface-active and facilitate their spreading and stable monolayer formation at the air-water interface. For example, composite LB films of molybdenum disulfide (MoS_2) and gold nanoparticles (AuNPs) have been developed for biosensing applications [84], where the LB deposition ensures a controlled distribution of

the nanomaterials on the electrode surface. Similarly, glucose oxidase enzymes have been co-assembled with AuNPs using the LB method to create sensitive glucose biosensors [85, 86]. Nanocomposites of N-Vinylpyrrolidon (NVP) and manganese dioxide (MnO_2) nanoparticles, as well as graphene oxide (GO)/MnO_2 complexes stabilized by lipids like dimyristoylphosphatidic acid (DMPA), have been fabricated via LB for energy storage applications in supercapacitors [87], leveraging the high surface area and controlled morphology provided by the technique.

Carbon nanomaterials, particularly carbon nanotubes (CNTs) and graphene-based materials (graphene, graphene oxide (GO), reduced graphene oxide (rGO)), are widely used in LB films due to their exceptional electrical, mechanical, and thermal properties. Single-walled carbon nanotubes (SWNTs) and multi-walled carbon nanotubes (MWCNTs) can be dispersed and assembled at the air-water interface, often functionalized or mixed with surfactants or polymers to improve dispersibility and film formation [33]. For instance, polyaniline-functionalized MWCNTs (PANI@MWCNTs) have been assembled using LB to create ordered films for gas sensing applications [88, 73], where the ordered structure potentially enhances sensor response. Hybrid structures combining SWNTs and graphene nanosheets (GNS) have been fabricated layer-by-layer using LB [89], resulting in anode materials for lithium-ion batteries with high specific capacity and excellent cycling performance. Graphene oxide, being amphiphilic due to its oxygen-containing functional groups, readily forms stable Langmuir films [33]. The LB technique allows precise control over the deposition of GO monolayers and multilayers [90, 8, 7], enabling studies of its phase behavior and applications in electronics, sensors, and as templates. For example, tin oxide/reduced graphene oxide (SnO_2/RGO) nanocomposites prepared by LB have shown promise as binder-free anodes for lithium-ion batteries [91].

Two-dimensional (2D) materials beyond graphene, such as transition metal dichalcogenides (e.g., MoS_2, WS_2) and nanosheets derived from layered materials (e.g., $Ca_2Nb_3O_{10}$), are increasingly being incorporated into LB films [92, 21]. The LB method facilitates the alignment and transfer of these nanosheets onto various substrates, preserving their 2D nature and enabling the exploitation of their unique electronic and optical properties [93, 18]. For example, $Ca_2Nb_3O_{10}$ nanosheets deposited onto poly(vinylidene fluoride) films via LB have been shown to enhance the dielectric properties of the polymer.

Furthermore, the Langmuir trough environment can serve as a template

or reaction medium for the synthesis or assembly of nanomaterials [1, 94]. For instance, metal ions complexed with fatty acid monolayers can be used as precursors for the in-situ formation of metal oxide or sulfide nanoparticles within the LB film structure upon subsequent treatment. The ordered structure of LB films can also template the growth of other materials, such as the fabrication of vertical nanochannels in polyimide films using LB-deposited silica nanospheres as masks. The precise control over particle spacing and arrangement offered by the LB technique is advantageous for applications relying on collective phenomena, such as plasmonics or catalysis [95, 81].

Table 2: Examples of Nanomaterials Incorporated into Langmuir-Blodgett Films and Their Applications.

**Nanomaterial Type**	**Specific Example(s)**	**Reported Application(s)**
Metal Nanoparticles	Gold (AuNPs), Silver (AgNPs)	Biosensing (glucose, dengue), SERS substrates, Electrocatalysis
Metal Oxide Nanoparticles	MnO_2, SnO_2, TiO_2	Energy storage (supercapacitors, batteries), Photocatalysis, Gas sensing
Carbon Nanotubes	SWNTs, MWCNTs (often functionalized, e.g., PANI@MWCNTs)	Gas sensing, Battery electrodes (Li-ion), Conductive films
Graphene & Derivatives	Graphene Nanosheets (GNS), Graphene Oxide (GO), Reduced Graphene Oxide (rGO)	Battery electrodes (Li-ion), Supercapacitors, Sensors, Transparent conductive films, Electrocatalysis
Transition Metal Dichalcogenides	MoS_2, WS_2	Biosensing, Electrocatalysis, Optoelectronics
Other Nanosheets	$Ca_2Nb_3O_{10}$	Dielectric materials enhancement
Colloidal Spheres	Silica, Polystyrene	Photonic crystals, Templating for nanostructures
Nanowires	MnO_2	On-chip battery diagnostics

3.3 Emerging Materials

Beyond traditional amphiphiles and common nanomaterials, the scope of LB technology is continually expanding to include novel classes of materials, offering pathways to films with highly sophisticated structures and advanced functionalities. These emerging materials often possess unique characteristics like high porosity, intrinsic catalytic activity, or specific electronic properties, which can be advantageously organized using the precise control afforded by the LB technique.

Metal-Organic Frameworks (MOFs) are crystalline porous materials constructed from metal ions or clusters coordinated to organic ligands [96]. Their high surface area, tunable pore sizes, and chemical versatility make them attractive for applications in gas storage, separation, catalysis, and sensing [97, 98]. While typically synthesized as bulk powders or crystals, fabricating MOFs as thin films is crucial for many device applications. The LB technique has emerged as a viable method to assemble pre-synthesized MOF nanosheets or nanoparticles into ordered films at the air-water interface [99, 100], representing a relatively well-explored area among emerging materials in LB research [1]. For example, 2D MOF nanosheets, such as triphenylene-based nanosheets [101] or $[Co_3(HOB)_2]_n$ films, have been prepared using LB methods [102]. These MOF LB films have demonstrated potential in electrocatalysis (e.g., hydrogen evolution reaction, H_2O_2 reduction) [103, 102] and sensing [104]. The LB approach allows for control over the orientation and packing of the MOF components, potentially influencing substrate accessibility and overall device performance. Combining LB with layer-by-layer growth techniques has also been explored to create well-defined MOF thin films [10, 105, 106, 107, 108].

Covalent Organic Frameworks (COFs) are another class of crystalline porous polymers built from organic building blocks linked by strong covalent bonds [109, 110, 111]. Like MOFs, they offer high porosity and tunable structures but are typically composed of light elements and can exhibit high chemical stability [97, 112]. While the application of LB techniques specifically to COFs is less established compared to MOFs, the principles of assembling pre-formed 2D COF nanosheets [100, 101] or potentially utilizing interfacial polymerization strategies [113, 114, 115] within the Langmuir trough could be explored to create ordered COF thin films [116]. Their development within the LB context is an area of ongoing research.

Other 2D materials beyond graphene and TMDs are also being explored

within the LB context. For instance, nitrogen-doped fullerphene films, described as graphene-like 2D materials derived from fullerene assembly at a liquid-liquid interface (water-xylene) in the presence of ethylenediamine, represent a novel carbon-based nanosheet structure [117]. While synthesized interfacially, the principles of controlled assembly and transfer inherent to LB-type methods are relevant for handling such large-area 2D structures [1, 21, 93]. The unique electronic structure resulting from nitrogen doping could lead to applications in catalysis or electronics [118].

Molecular machines and switches, molecules capable of performing mechanical motion or switching between states in response to external stimuli (light, heat, chemical signals), represent another frontier [119, 120, 121, 122, 123]. Incorporating these functional molecules into ordered LB assemblies could lead to smart surfaces and membranes with controllable properties [124, 2, 4]. However, applying the LB technique directly to create functional films of molecular machines is still a developing area, facing challenges in maintaining functionality and achieving coordinated action within a condensed film. Often, related interfacial assembly methods, like layer-by-layer assembly incorporating molecular motors into MOF structures, are employed [125, 96], demonstrating the potential for precise organization of such complex molecular systems, which shares conceptual similarities with LB approaches [106, 126, 1].

The continuous development of synthetic chemistry provides an ever-expanding library of molecules and nanomaterials with tailored properties. The LB technique remains a relevant and powerful tool for organizing these new building blocks into functional thin-film architectures. The ability to precisely control film thickness, molecular orientation, and packing density at the nanoscale makes LB an indispensable method for exploring the potential of emerging materials in areas ranging from molecular electronics and spintronics to advanced catalysis and biomedical devices. The combination of novel material synthesis with the refined assembly capabilities of the LB method promises continued innovation in the field of functional thin films.

4 Fabrication Techniques and Parameters

The successful creation of well-ordered Langmuir and Langmuir-Blodgett films relies heavily on precise control over the fabrication process, from the initial preparation of the air-liquid interface to the final transfer onto a solid

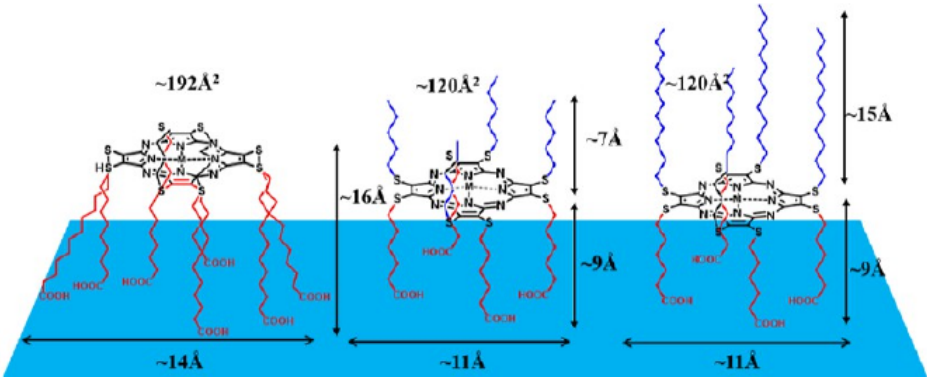

Figure 8: Proposed face-on conformers at the air—water interface of a previously reported octaacid TAP (left) with alkyl spacers of 10 methylene groups and the two tetraacid TAPs 4a (center) and 4b (right) reported here. Estimated chain lengths and TAP surface areas are taken from previous studies. The side-chain and spacer lengths represent maximum values for fully extended (crystalline) alkyl chains. Side chains in an amorphous state have a 10—20% shorter effective length. Adapted from Alinia, Z., et al., 2024 [52].

support. This involves sophisticated instrumentation, careful selection of materials and solvents, and meticulous adjustment of various physical and chemical parameters. Understanding these techniques and parameters is crucial for tailoring film properties such as thickness, molecular packing, orientation, and defect density for specific applications. The versatility of the Langmuir-Blodgett technique stems from its ability to manipulate molecules and nanomaterials at interfaces, allowing for the construction of complex nanoarchitectures layer by layer.

4.1 Langmuir Trough Design and Operation

The cornerstone of Langmuir and Langmuir-Blodgett film fabrication is the Langmuir trough, a specialized apparatus designed to control the formation and manipulation of monomolecular films at a liquid interface, typically the air-water interface. A standard Langmuir trough consists of several key components, as schematically shown in Figure 11. The trough itself is usually machined from a hydrophobic, chemically inert material like Polytetrafluoroethylene (PTFE) to contain the liquid subphase and prevent leakage or

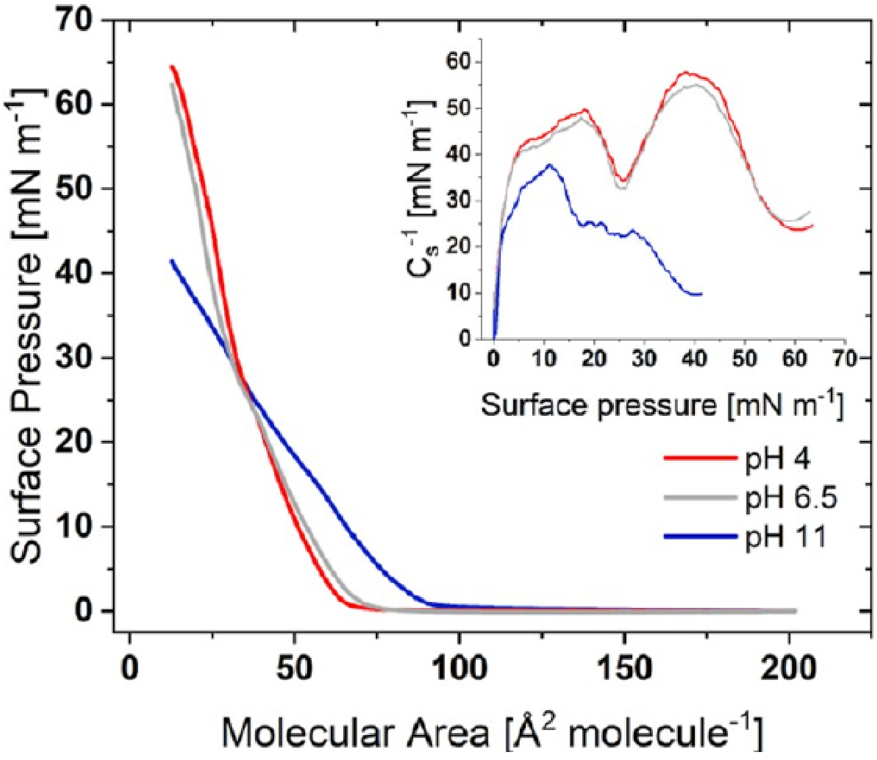

Figure 9: Surface pressure—molecular area isotherms and compressibility modulus plot (inset) of 4b at different subphase pH with a compression rate of 10 cm^2 min^{-1}, 22 °C. Adapted from Alinia, Z., et al., 2024 [52].

contamination. Moveable barriers, often made of hydrophilic materials like polyacetal (Delrin) or hydrophobic PTFE, span the width of the trough and rest on its edges. Hydrophilic barriers are often preferred as they help maintain a continuous water meniscus along the barrier edge, preventing leakage of the monolayer underneath, especially during compression. These barriers are precisely controlled, usually by computer-driven motors, to compress or expand the surface area available to the floating monolayer. The surface pressure (π), defined as the reduction in surface tension of the pure subphase caused by the presence of the monolayer ($\pi = \gamma_0 - \gamma$, where γ_0 is the surface tension of the pure subphase and γ is the surface tension with the film), is continuously monitored. This is most commonly achieved using the Wilhelmy plate method (see Figure 11), where a plate (often made of platinum or filter paper) is partially immersed in the subphase and suspended from a sensitive

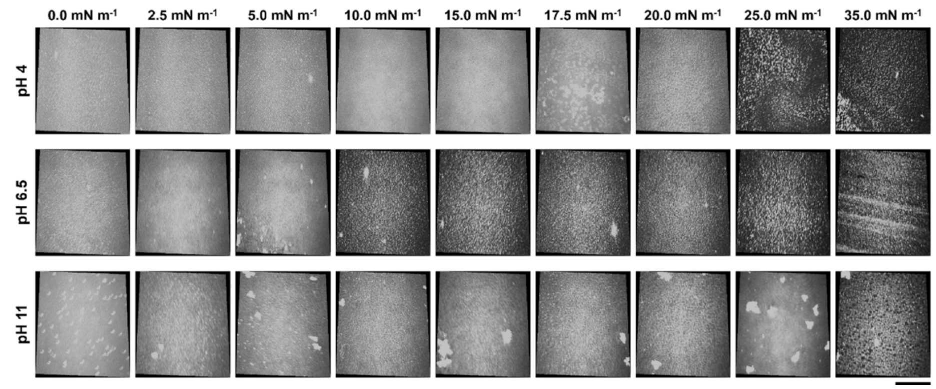

Figure 10: Representative BAM images (620 µm X 520 µm) of 4b at different subphase pH values of 4 (top), 6.5 (middle), and 11 (bottom) and surface pressures from 0.0 to 35.0 mN m^{-1} (brightness of −30% and contrast of +20% modification). Adapted from Alinia, Z., et al., 2024 [52].

microbalance or pressure sensor. The microbalance measures the change in force exerted on the plate due to the change in surface tension (γ) caused by the monolayer, allowing for the direct calculation of surface pressure π. Modern troughs (e.g., KSV NIMA, Kibron) integrate these components with automated control systems for precise barrier movement, pressure monitoring, and data acquisition, enabling the recording of surface pressure-area (π-A) isotherms. For LB deposition, a dipping mechanism is incorporated, allowing a solid substrate to be vertically immersed into and withdrawn from the subphase through the monolayer at a controlled speed.

The operation of a Langmuir trough follows a general procedure. First, the trough and barriers must be meticulously cleaned, typically with high-purity solvents like ethanol and chloroform, followed by rinsing with ultrapure water, to remove any surface-active contaminants. The trough is then filled with the chosen subphase, most commonly ultrapure water (e.g., 18 MΩ·cm Milli-Q water). The surface of the subphase is often cleaned by aspiration or by sweeping the barriers across the surface to remove any residual impurities before the film-forming material is introduced. After spreading the material and allowing sufficient time for solvent evaporation, the barriers are moved inwards at a controlled speed to compress the monolayer [15]. The π-A isotherm is recorded during compression, providing critical information about the film's phase behavior and packing density. Once the desired surface pressure (transfer pressure) is reached and stabilized, the Langmuir mono-

layer can be transferred onto a solid substrate to form a Langmuir-Blodgett film [2, 93].

While ultrapure water is the standard subphase, its composition can be modified to influence Langmuir film formation and structure. Adding ions (e.g., divalent cations like Ca^{2+} or Cd^{2+}) can interact with charged head groups of amphiphiles (like fatty acids), affecting packing, stability, and transfer characteristics [39, 127]. Adjusting the pH or adding buffers (e.g., Phosphate-Buffered Saline, PBS) is also common, particularly for biological molecules or pH-sensitive materials [128, 129]. For materials that are insoluble or unstable on water, or for exploring different interfacial phenomena, non-aqueous subphases can be employed. Recent advancements include high-temperature LB systems utilizing subphases like ethylene glycol or ionic liquids, which have low vapor pressure and allow film formation and transfer at elevated temperatures (up to 200 °C) [22]. This enables the processing of highly condensed materials like conjugated polymers, promoting molecular orientation and crystallinity.

Environmental factors significantly impact the reproducibility and quality of Langmuir and LB films. Temperature control is crucial as it affects molecular motion, phase transitions, subphase viscosity, and evaporation rates. Many troughs incorporate heating or cooling stages to maintain a constant subphase temperature (e.g., 20 ± 1 °C or 37 °C for biomimetic studies) or to study temperature-dependent behavior [17, 130, 38, 37]. Humidity control can also be important, especially for long experiments, to minimize subphase evaporation. Perhaps most critical is the need for a vibration-free and dust-free environment. Troughs are often placed on anti-vibration tables, and experiments are ideally conducted in cleanrooms or under laminar flow hoods to prevent mechanical disturbances and particulate contamination of the delicate monolayer.

4.2 Spreading Techniques

The initial step in forming a Langmuir film is the deposition of the film-forming material onto the subphase surface. This is typically achieved by dissolving the material in a suitable spreading solvent and carefully applying the solution to the interface. The most common method is drop-casting, where a precise volume of the solution is dispensed drop by drop onto the subphase surface using a microsyringe (e.g., Hamilton chromatographic syringe). The choice of spreading solvent is critical. It must effectively dissolve

the material of interest, be immiscible with the subphase (usually water), have a positive spreading coefficient to ensure it spreads rapidly across the surface rather than forming lenses, and be sufficiently volatile to evaporate completely within a reasonable timeframe (typically 10-20 minutes) without leaving residues or disturbing the forming monolayer. Chloroform is a very popular choice due to its ability to dissolve many amphiphiles and its suitable volatility and spreading characteristics. Other common solvents include hexane, toluene, dichloromethane, or mixtures thereof, often chosen based on the solubility of the specific material. Ethanol/water mixtures have also been used, for example, for spreading graphene oxide dispersions.

Several factors influence the quality of the spread Langmuir film. The solvent purity is paramount, as impurities can act as surface contaminants. The concentration of the material in the spreading solution affects the initial density of molecules on the surface after evaporation. Too high a concentration might lead to the formation of aggregates or multilayers even before compression begins. The volume of solution spread determines the initial surface area occupied by the molecules, which should be large enough to ensure the molecules are initially in a gaseous phase with minimal interactions. The solution should be added gently, often near one end of the trough or along the barriers, to minimize disturbance to the subphase surface. The properties of the subphase itself, such as its surface tension, temperature, and pH, can also influence how effectively the solvent spreads and the material arranges itself after evaporation [38]. For instance, the presence of ions or surfactants in the subphase can alter the spreading behavior [39].

While drop-casting is prevalent, alternative methods exist. Spraying the solution can sometimes yield more uniform initial distributions, although controlling the deposition rate and area can be challenging. For particulate materials like colloidal spheres, spreading can sometimes be facilitated by using a hydrophilic glass slide dipped into the suspension and then touched to the subphase surface [131]. Microfluidic techniques are also emerging as a way to deliver materials to the interface with high precision, although they are not yet standard practice in most LB setups. Regardless of the method, the goal is to achieve a uniform, dilute distribution of the film-forming molecules or particles across the air-liquid interface prior to compression, forming the initial Langmuir film.

4.3 Compression and Transfer Parameters

Once the film-forming material is spread and the solvent has evaporated, the Langmuir monolayer is typically compressed by moving the barriers inward. This process reduces the area available per molecule (A), causing the molecules to transition through different two-dimensional phases (gaseous, liquid-expanded, liquid-condensed, solid) accompanied by an increase in surface pressure (π). The relationship between π and A is captured in the π-A isotherm, a characteristic signature of the monolayer material and its interactions at the interface. The compression speed is a critical parameter. Typical speeds range from a few mm/min to tens of mm/min (e.g., 5 mm/min, 10 mm/min, 20 mm/min). If the compression is too rapid, the molecules may not have sufficient time to rearrange into an equilibrium packed structure, potentially leading to film collapse, buckling, or the formation of defects and disordered domains. Conversely, excessively slow compression significantly increases experimental time and may allow for undesirable relaxation processes or dissolution of the monolayer into the subphase for slightly soluble materials. The optimal compression rate depends on the specific material and experimental conditions, aiming for a quasi-equilibrium compression where the isotherm is reproducible.

The target surface pressure, or transfer pressure, is the specific surface pressure at which the Langmuir monolayer is transferred onto the solid substrate to create the LB film. This pressure is chosen based on the π-A isotherm, typically within the liquid-condensed or solid phase where the film is densely packed and stable, but below the collapse pressure where the monolayer structure breaks down into three-dimensional aggregates. The chosen transfer pressure significantly influences the density, order, thickness, and morphology of the deposited LB film [15]. For instance, transferring at higher pressures generally leads to more densely packed films, but may also incorporate defects formed during high compression. Studies have shown that film morphology, such as the presence of voids or multilayer regions, can be highly sensitive to the transfer pressure [40].

The transfer of the organized Langmuir monolayer from the air-liquid interface to a solid substrate is the defining step of the LB and LS techniques. In the Langmuir-Blodgett (LB) method, the substrate is moved vertically through the interface (Figure 11(a)) while the surface pressure is typically kept constant by adjusting the barrier position (feedback control). The dipping speed (both immersion and withdrawal) is a crucial pa-

rameter, usually in the range of 0.1 to 10 mm/min (e.g., 0.3 mm/min, 1.2 mm/min, 2 mm/min). The speed affects the efficiency of transfer [34] and the structure of the deposited film. The direction of transfer relative to the substrate's hydrophilicity determines deposition: typically, hydrophilic substrates pick up the monolayer during withdrawal (head groups interacting with the substrate), while hydrophobic substrates pick up the monolayer during immersion (hydrophobic tails interacting). By repeating the dipping cycle, multilayer films can be constructed. Depending on the transfer characteristics during immersion and withdrawal, different multilayer structures (X-type, Z-type, or more commonly Y-type with alternating orientations) can be formed.

In the Langmuir-Schaefer (LS) method, the substrate is brought into contact horizontally with the monolayer at the target pressure (Figure 11(b)) [22]. This method is often preferred for depositing single layers, for materials that do not transfer well vertically, or when a specific orientation parallel to the substrate is desired [93]. The barrier position is usually kept constant during LS transfer. Other less common transfer methods include the drainage technique, where the subphase is slowly drained away while the substrate rests at the bottom of the trough, allowing the monolayer to deposit onto it. Roll-to-roll techniques are also being developed for continuous, large-area deposition [132].

The efficiency of the transfer process is often quantified by the transfer ratio (TR), calculated as the ratio of the decrease in monolayer area on the trough surface to the area of the substrate coated during one pass. A TR close to unity indicates efficient and complete transfer. Deviations significantly different from unity may suggest incomplete transfer due to issues such as poor adhesion between the film and substrate [34], film rigidity or brittleness leading to fracture, film slippage on the substrate surface, or inappropriate transfer speeds causing hydrodynamic disturbances or incomplete deposition.

The nature of the substrate itself plays a significant role. Its material composition, surface energy (hydrophilicity/hydrophobicity), roughness [36], and cleanliness are critical for film adhesion and quality. Substrates are often rigorously cleaned (e.g., using Piranha solution for silicon, or ultrasonication in solvents for FTO glass) and sometimes pre-treated (e.g., UV/Ozone treatment to enhance hydrophilicity) to ensure proper film deposition. Furthermore, the orientation of the substrate relative to the compression axis during LB transfer can induce anisotropy in the deposited film, particularly for materials with anisotropic domains or flow alignment during compression

[133, 134].

In summary, the meticulous control of parameters such as compression speed, transfer pressure, dipping/transfer speed, subphase composition [40], temperature [135, 130], and substrate properties is essential for fabricating high-quality Langmuir films at the interface and Langmuir-Blodgett films on substrates with desired structural characteristics like controlled thickness, high molecular order, specific orientation, and minimal defects [46]. The interplay between these parameters allows for the fine-tuning of film properties for diverse technological applications [2, 4, 1]. Key fabrication parameters and their typical effects are summarized in Table 3.

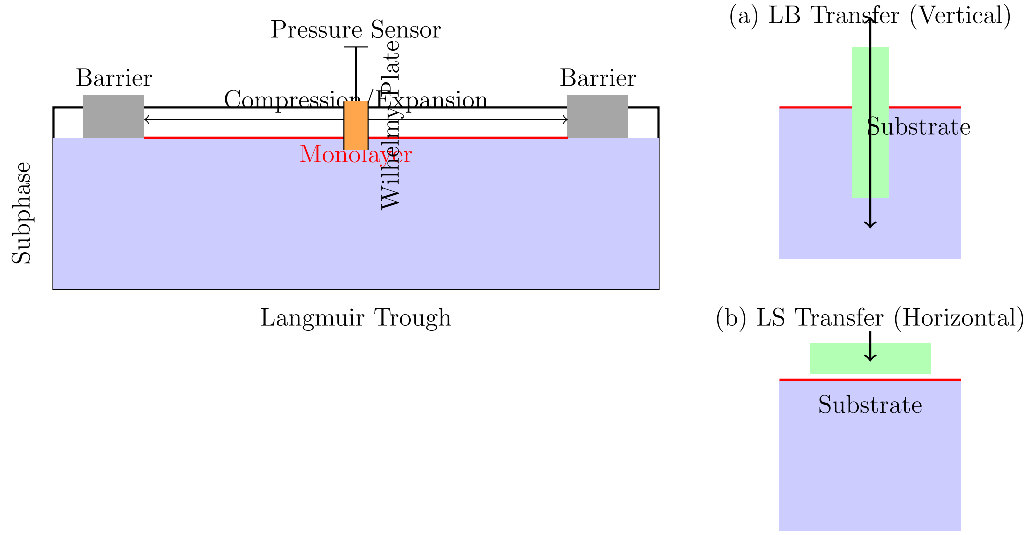

Figure 11: Schematic illustration of a Langmuir trough showing key components (trough, barriers, Wilhelmy plate/sensor) and the monolayer at the air-subphase interface. Also depicted are the two primary transfer methods: (a) Langmuir-Blodgett (LB) vertical dipping and (b) Langmuir-Schaefer (LS) horizontal contact.

5 Characterization Techniques for Langmuir and Langmuir-Blodgett Films

Characterization is indispensable for understanding the properties and behavior of both Langmuir monolayers formed at the air-water interface and the

Table 3: Key Fabrication Parameters and Their Typical Effects on Langmuir-Blodgett Film Properties.

**Parameter**	**Typical Range/Value**	**Primary Effects on Film Properties**
Spreading Solvent	Chloroform, Hexane, Toluene, etc.	Affects initial molecular distribution, potential for residual impurities. Must dissolve material, be volatile, and spread well.
Solution Concentration	0.1 - 1 mg/mL	Influences initial surface density, potential for pre-compression aggregation.
Subphase Composition	Ultrapure Water, Ionic solutions (e.g., $CaCl_2$), pH buffers (e.g., PBS), Non-aqueous (e.g., Ethylene Glycol)	Affects molecular interactions (packing, stability, charge), phase behavior, suitability for specific materials.
Temperature	15 - 40 °C (standard), up to 200 °C (high-temp LB)	Influences molecular mobility, phase transitions, film viscosity, solvent evaporation, stability.
Compression Speed	1 - 50 mm/min	Affects film homogeneity, defect density, relaxation time. Too fast leads to collapse/defects; too slow increases time/dissolution risk.
Transfer Pressure (π)	5 - 40 mN/m (typically in condensed phase)	Determines packing density, molecular orientation, film stability, thickness (indirectly), morphology (voids, domains).
Transfer Speed (LB/LS)	0.1 - 10 mm/min	Influences transfer efficiency (Transfer Ratio), molecular orientation during transfer, potential for defects.
Substrate Properties	Material (Si, Glass, FTO, etc.), Surface Energy, Cleanliness, Orientation	Affects adhesion, film structure, uniformity, potential for induced anisotropy.

subsequently transferred Langmuir-Blodgett (LB) films on solid substrates [2, 93]. A comprehensive analysis provides crucial insights into molecular arrangement, film thickness, surface morphology, structural homogeneity, and chemical composition [136]. These parameters are vital for correlating the fabrication process with the final film structure and its intended functionality. Characterization methods can be broadly categorized into *in-situ* techniques, which probe the monolayer directly at the air-water interface during its formation and compression, and *ex-situ* techniques, which analyze the LB film after its transfer onto a solid support [137].

5.1 In-situ Characterization at the Air-Water Interface

Studying the Langmuir monolayer *in-situ* allows for real-time monitoring of its formation [38], phase transitions [31, 138], and structural evolution under varying surface pressures [139].

5.1.1 Surface Pressure-Area (π-A) Isotherms

The surface pressure-area (π-A) isotherm is the most fundamental *in-situ* characterization technique for Langmuir films [139]. It measures the surface pressure (π), defined as the reduction in surface tension of the pure subphase caused by the monolayer ($\pi = \gamma_0 - \gamma$, where γ_0 is the surface tension of the pure subphase and γ is the surface tension in the presence of the monolayer), as a function of the area available per molecule (A) at a constant temperature. The isotherm is obtained by compressing the monolayer spread on the subphase surface with movable barriers while monitoring the surface pressure, typically using a Wilhelmy plate or Langmuir balance [79, 140].

The π-A isotherm provides valuable information about the phase behavior of the monolayer [30, 31]. As the area per molecule decreases during compression, the monolayer can transition through various two-dimensional phases, analogous to the gas, liquid, and solid phases in three dimensions. Typical phases observed include the gaseous (G) phase, where molecules are far apart and interact weakly; the liquid-expanded (LE) phase, where molecules begin to interact but retain fluidity; the liquid-condensed (LC) phase, characterized by closer packing and reduced compressibility; and the solid (S) phase, where molecules are tightly packed in an ordered arrangement [138]. Further compression beyond the solid phase leads to monolayer collapse [32, 141],

where the 2D structure breaks down, often forming multilayers or aggregates. Figure 13 illustrates typical π-A isotherms for a porphyrin derivative at different subphase pH values. The shape of the isotherm, including the slopes and transition points, reveals the different phases and their stability ranges. Extrapolating the steepest part of the isotherm (often corresponding to the solid or condensed phase) to zero surface pressure yields the limiting molecular area (A_0), providing an estimate of the cross-sectional area occupied by each molecule in its most compressed state [142].

The compressibility modulus (C_s^{-1}), calculated as $C_s^{-1} = -A(\partial\pi/\partial A)_T$, provides quantitative information about the film's elasticity and helps identify phase transitions more clearly. Maxima or distinct regions in the compressibility modulus versus surface pressure plot, such as the inset in Figure 13, correspond to different phases (e.g., LE phase typically has C_s^{-1} between 12.5-50 mN/m, LC phase between 100-250 mN/m, and S phase ¿ 250 mN/m). However, for flexible materials like graphene oxide, conventional π-A and compressibility isotherms may not show distinct transitions, requiring alternative analysis methods like tensile modulus isotherms [143, 144, 145].

5.1.2 Optical Microscopy Techniques

In-situ optical techniques allow direct visualization of the monolayer morphology at the air-water interface during compression. **Brewster Angle Microscopy (BAM)** is a non-invasive technique that utilizes the principle that p-polarized light incident at the Brewster angle of the subphase (e.g., water, $\sim$53.1°) experiences minimal reflection. When a monolayer is present, its different refractive index causes reflection, allowing visualization of the film's morphology, domain formation, phase coexistence, and homogeneity with micrometer resolution [146, 80, 147]. BAM is particularly useful for observing the formation and evolution of condensed phase domains within a liquid-expanded phase during compression.

Ellipsometry measures the change in polarization state of light upon reflection from the interface [148]. By analyzing the ellipsometric angles (Ψ and Δ), information about the monolayer's average thickness and refractive index can be obtained *in-situ* [149]. Ellipsometric isotherms, plotting changes in Δ versus surface pressure, can track changes in film thickness and density during compression, complementing the information from π-A isotherms.

5.2 Ex-situ Characterization of Langmuir-Blodgett Films

Once the Langmuir monolayer is transferred onto a solid substrate, various *ex-situ* techniques are employed to characterize the resulting LB film's structure [20], morphology [150, 133], and composition [151].

5.2.1 Microscopic Techniques

Microscopy provides direct visualization of the film's surface topography and structure. **Atomic Force Microscopy (AFM)** is a powerful technique for imaging the surface topography of LB films with nanometer or even subnanometer resolution [152, 153]. It operates by scanning a sharp tip attached to a cantilever across the sample surface. AFM can provide quantitative data on film roughness, domain size and height, thickness of single or multiple layers [40], and identify defects like pinholes or aggregates [154]. Figure 14 shows AFM images of a porphyrin LB film deposited at different surface pressures and pH values, revealing significant changes in morphology, domain structure, and coverage depending on the deposition conditions. AFM can also be used in modes that probe mechanical properties (e.g., PeakForce Tapping) or surface potential (Kelvin Probe Force Microscopy). Functionalized AFM tips can be used for single-molecule force spectroscopy (SMFS) to measure adhesion forces [155].

Scanning Electron Microscopy (SEM) provides images of the surface morphology over larger areas compared to AFM, typically with slightly lower resolution. SEM is useful for assessing overall film uniformity, coverage, and identifying larger-scale features or defects [156]. It requires the sample to be conductive or coated with a conductive layer.

Transmission Electron Microscopy (TEM) can be used to analyze the cross-section or planar structure of LB films, especially those containing nanoparticles or specific structural features [157], offering very high resolution.

Scanning Tunneling Microscopy (STM) is applicable to conductive LB films deposited on conductive substrates [158, 71, 159], providing atomic-resolution images of the molecular arrangement [160, 150].

5.2.2 Spectroscopic Techniques

Spectroscopy probes the chemical composition, molecular orientation, and electronic structure of LB films. **Fourier Transform Infrared Spec-**

troscopy (FTIR) [23] and **Raman Spectroscopy** [161] are vibrational spectroscopies used to identify chemical bonds and functional groups within the film material [162]. By using specific configurations like grazing angle incidence FTIR [24] or polarized Raman spectroscopy, information about the average molecular orientation within the LB film can be obtained [163]. For example, the orientation of alkyl chains in fatty acid LB films can be determined by analyzing the intensity of specific CH_2 stretching modes [164].

UV-Visible (UV-Vis) Spectroscopy is used to study the electronic absorption properties of LB films, particularly those containing chromophores like porphyrins [165, 166], dyes [104], or conjugated polymers [167, 168]. Changes in absorption spectra compared to the solution state can indicate molecular aggregation (e.g., formation of J- or H-aggregates) [169, 170] and provide insights into intermolecular interactions and molecular packing within the film.

X-ray Photoelectron Spectroscopy (XPS), also known as Electron Spectroscopy for Chemical Analysis (ESCA), provides information about the elemental composition and chemical states of atoms near the surface (top few nanometers) of the LB film [171]. It is a surface-sensitive technique useful for verifying film composition [172, 173] and detecting contaminants.

5.2.3 X-ray Scattering Techniques

X-ray scattering techniques provide detailed structural information about the molecular packing and layering within LB films [136]. **X-ray Reflectivity (XRR)** measures the specular reflection of X-rays from the film surface at grazing incidence angles [174]. Analysis of the resulting interference pattern (Kiessig fringes) allows precise determination of the film's total thickness, electron density profile perpendicular to the surface, and interfacial roughness [36]. It is highly effective for characterizing the layered structure of multilayer LB films [40, 135].

Grazing Incidence X-ray Diffraction (GIXD) probes the in-plane structural order within the LB film [40]. By measuring diffraction peaks at grazing incidence, GIXD can determine the lateral packing arrangement of molecules (unit cell dimensions) and their average tilt angle with respect to the surface normal. GIXD can be performed *in-situ* at the air-water interface using synchrotron radiation sources or *ex-situ* on transferred LB films [136, 135].

5.2.4 Other Techniques

Neutron Reflectivity (NR) is analogous to XRR but uses neutrons instead of X-rays [174, 175, 176, 68]. It is particularly powerful when combined with isotopic substitution (e.g., deuteration), allowing specific components of a complex film or buried interfaces to be highlighted due to the different neutron scattering lengths of isotopes (contrast variation).

5.3 Molecular Orientation

The orientation of molecules within Langmuir and LB films is a critical parameter influencing the film's properties and performance in applications. For instance, in films made of macrocyclic molecules like porphyrins, the orientation can be broadly classified as "edge-on" (macrocycle plane perpendicular or tilted relative to the interface) or "face-on" (macrocycle plane parallel to the interface), as schematically shown in Figure 12. This orientation affects electronic properties, charge transport [177], light absorption, and accessibility for sensing or catalysis. Techniques like polarized UV-Vis spectroscopy, angle-resolved FTIR or Raman spectroscopy [23, 24], and GIXD are instrumental in determining the average molecular orientation within the film [178]. Controlling molecular orientation is a key aspect of designing functional LB films for specific applications [124, 179, 2].

In summary, a combination of *in-situ* and *ex-situ* characterization techniques is typically employed to gain a comprehensive understanding of Langmuir monolayers and Langmuir-Blodgett films [1, 93, 2]. The choice of techniques depends on the specific material system and the properties of interest, ranging from macroscopic phase behavior and morphology to microscopic molecular arrangement and chemical composition.

6 Applications of Langmuir and Langmuir-Blodgett Films

Langmuir-Blodgett (LB) and Langmuir film technologies, offering precise molecular-level assembly and control over film thickness and structure, have enabled extensive applications across diverse scientific and technological fields. The ability to create highly ordered, ultra-thin films from a wide variety of materials—including small molecules, polymers, nanoparticles, and biomolecules—makes

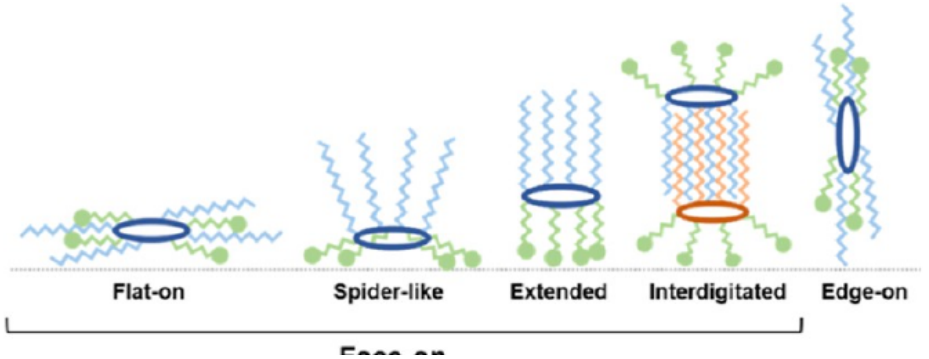

Figure 12: Schematic representation of the edge-on (left) and face-on (right) orientations of a macrocycle at the air—water interface. The specific molecular structure shown is a porphyrin derivative. Adapted from Alinia, Z., et al., 2024 [52].

these techniques uniquely suited for applications where interfacial properties and molecular organization are paramount. Key application domains include high-performance chemical and biological sensing, advanced organic electronics and optoelectronics, biomaterials engineering for mimicking biological systems and interfacing with them, energy storage solutions involving nanostructured electrodes and interfaces, and emerging areas like tailored catalysis and tribology. The versatility of Langmuir and LB methods allows for the customization of film properties to meet specific functional requirements, driving innovation in materials science, nanotechnology, and beyond. This section explores these key application domains, highlighting the underlying principles that enable their utility and showcasing recent advancements facilitated by both Langmuir films at interfaces and transferred LB films on solid supports.

6.1 Sensing Applications

The precise control over molecular arrangement and film thickness afforded by the Langmuir and LB techniques makes them exceptionally valuable for developing high-performance sensors. Both Langmuir films at the air-water interface and transferred LB films serve as ideal platforms for chemical sensors, biosensors, and immunosensors. Their highly ordered structures, large surface-area-to-volume ratios, and tunable surface properties collectively en-

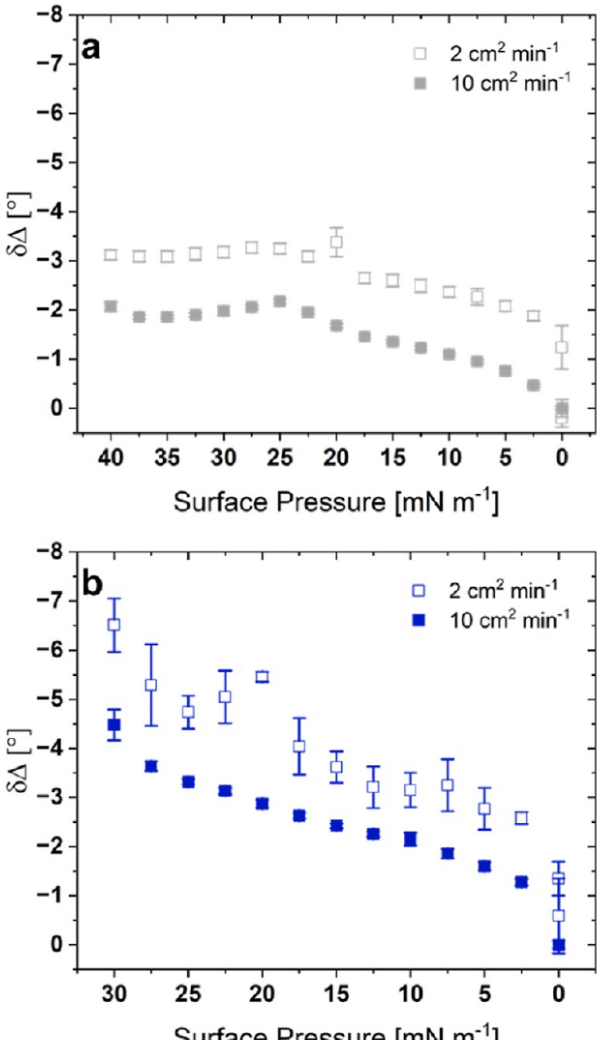

Figure 13: Surface pressure—molecular area (π-A) isotherms of a porphyrin derivative (4b from the source reference) at different subphase pH values (6.5, 8.0, 9.0, 11.0). The inset shows the corresponding compressibility modulus (C_s^{-1}) plotted against surface pressure, highlighting different film phases and their dependence on pH. Compression rate: 10 cm^2 min^{-1}, Temperature: 22 °C. Adapted from Alinia, Z., et al., 2024 [52].

hance sensitivity, selectivity, and response speed.

In the realm of gas sensing, LB films offer significant advantages. The

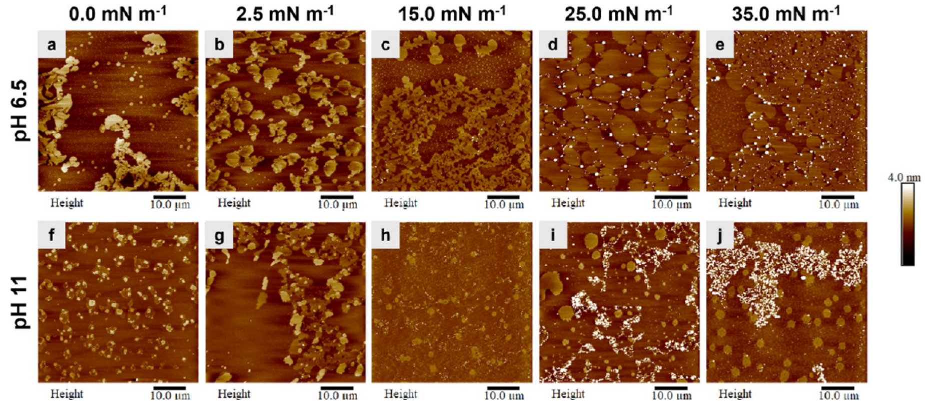

Figure 14: Atomic Force Microscopy (AFM) topography images (5 μm x 5 μm) of Langmuir-Blodgett films of a porphyrin derivative (4b from the source reference) transferred onto mica substrates at different surface pressures and subphase pH values. Top row (a-e): pH 6.5. Bottom row (f-j): pH 11. Surface pressures for transfer (from left to right): 0.0, 2.5, 15.0, 25.0, and 35.0 mN m^{-1}. The images reveal the evolution of film morphology, domain structure, and coverage with increasing surface pressure and changing pH. Adapted from Alinia, Z., et al., 2024 [52].

ultra-thin nature of these films, often ranging from nanometers to tens of nanometers, facilitates rapid diffusion of gas molecules into the sensing layer, leading to faster response times. Furthermore, the ordered molecular assembly allows for optimized interaction between the sensing material and the target analyte, as schematically illustrated in Figure **??**. This controlled structure maximizes the exposure of active sites to the analyte, enhancing the sensor's responsiveness. Researchers have successfully fabricated gas sensors using various materials deposited via LB techniques [2, 4]. For instance, ultra-thin films of polyaniline-functionalized multi-walled carbon nanotubes (PANI@MWCNTs) prepared by LB showed highly ordered structures suitable for gas detection [2]. Fatty acid LB films, such as those composed of arachidic acid (AA) and stearic acid (SA), have been investigated as sensing layers on surface acoustic wave (SAW) devices for the selective detection of volatile organic compounds (VOCs) like chloroform, demonstrating enhanced response compared to other VOCs [2, 180]. The high surface area and reactivity of fatty acid salt LB films have also been exploited for detecting hazardous gases like hydrogen sulfide (H2S) at low concentrations and room tem-

perature, overcoming limitations of traditional inorganic sensors requiring high operating temperatures [181]. Composite LB films incorporating novel molecules, such as carbazole derivatives complexed with dyes (e.g., azobenzene, methyl orange, Congo red), have been developed as visual, self-powered sensors responsive to acidic (HCl) and alkaline (NH3) gases through simple protonation/deprotonation mechanisms, indicated by changes in open-circuit voltage [2, 182, 183]. Metal-organic frameworks (MOFs), known for their high porosity and tunable structures, can also be processed into thin films using LB techniques, offering potential for highly selective gas adsorption and sensing applications [2, 116]. Similarly, LB films based on cuproaromatic compounds have shown sensitivity towards various VOCs, including chlorinated compounds, acetone, methanol, and benzene, enabling label-free, real-time detection [2, 184].

7 Challenges and Future Directions

Langmuir-Blodgett (LB) technology, despite its century-long history and unique capability for fabricating highly ordered ultrathin films with molecular precision [1, 3, 4], faces several significant challenges that currently limit its widespread industrial adoption and application scope. Addressing these limitations is paramount for the continued advancement and exploitation of this powerful nanoarchitectonics tool [185, 186, 179]. Key challenges revolve around film stability [46, 187], process scalability [132], the complexity inherent in achieving consistent structural control [15], material versatility, and the need for more sophisticated characterization methods. Overcoming these hurdles requires concerted research efforts, innovative approaches, and interdisciplinary collaboration, focusing on both the Langmuir monolayer at the interface and the transferred LB film on the substrate [136].

One of the primary concerns regarding LB films is their inherent stability, encompassing mechanical robustness, chemical resistance, and long-term performance [187, 41, 16]. Many LB films, particularly those composed of simple amphiphiles like fatty acids or delicate biological molecules, can be fragile and susceptible to degradation or structural rearrangement upon exposure to harsh environmental conditions, mechanical stress, or even prolonged storage [17, 188]. This lack of robustness poses a significant challenge for practical applications, such as maintaining the integrity and sensitivity of sensor layers under operational conditions or ensuring the durability of

electrode and interface coatings in battery systems subjected to repeated charge-discharge cycles. Research efforts are needed to develop strategies for enhancing film stability, such as incorporating cross-linkable moieties within the film-forming molecules, utilizing more robust materials like polymers or nanoparticles, or creating hybrid structures where the LB film is protected or reinforced by other layers or matrices [126, 189]. Post-deposition treatments could also play a role in improving film durability.

Scalability and cost-effectiveness represent another major bottleneck for the transition of LB technology from laboratory research to industrial manufacturing. The conventional LB deposition process, involving slow compression and dipping cycles within a trough (illustrated conceptually in Figure 15), is often time-consuming and not readily amenable to high-throughput production over large areas. While the technique is compatible with large-area fabrication in principle, practical implementation faces challenges. Future research should focus on developing faster and more continuous deposition methods. Innovations like roll-to-roll compatible LB processes, multi-needle deposition systems, or alternative approaches like the vortex-LB method could offer pathways towards improved scalability. Additionally, the Langmuir-Schaefer (LS) technique, involving horizontal transfer of the monolayer, presents a potentially faster deposition alternative compared to vertical LB dipping for certain applications requiring simpler monolayer or multilayer structures, albeit with potentially different structural outcomes [22]. Furthermore, simplifying the required instrumentation [190, 146] and reducing the cost associated with high-purity subphases and specialized solvents are crucial for making the technology more economically viable.

Achieving precise and reproducible control over the film's structure remains a complex task, despite being a hallmark advantage of the LB technique. The goal is to meticulously control molecular orientation (e.g., edge-on versus face-on), packing density, domain formation, and minimize defects across macroscopic areas. However, this requires careful optimization of numerous influencing factors, including subphase composition (pH, ionic strength, temperature), compression speed, target surface pressure, deposition speed, and substrate properties. This complexity is amplified when dealing with non-ideal film-forming materials, such as flexible polymers [126], soft nanomaterials like graphene oxide (GO) [7], or molecules prone to aggregation. Understanding and controlling the phase transitions of Langmuir films at the air-water interface is critical, particularly for materials like GO where conventional surface pressure-area isotherm analysis proves insufficient

to fully describe the behavior, necessitating alternative approaches like elastic tensile modulus isotherms. Advanced strategies, potentially involving external fields (electric, magnetic) [191], patterned substrates, or unconventional subphases (e.g., ionic liquids, high-temperature liquids), are needed to guide molecular assembly with greater precision. Improving the fundamental understanding of intermolecular and interfacial forces governing film formation is essential for developing predictive models and rational design principles. Ensuring high reproducibility between experiments and across different laboratories also requires standardization of protocols and instrumentation.

The range of materials amenable to LB deposition, while broad, still faces limitations. Traditionally, the technique relies on amphiphilic molecules capable of forming stable monolayers at the air-water interface. Extending the technique to non-amphiphilic molecules, highly cohesive materials, or materials with limited solubility or stability often requires significant modifications or advanced approaches. A particular challenge lies in transferring complex or delicate biological macromolecules, such as enzymes or peptides used in biosensors and biomimetic films, while preserving their intricate three-dimensional structure and biological function [192, 193]. The development of high-temperature LB methods, allowing deposition from non-volatile subphases like ethylene glycol or ionic liquids at elevated temperatures (up to 200 °C, as conceptually shown for polymer semiconductors in related work), represents a significant step towards processing materials like functional conjugated polymers that are difficult to handle at room temperature. Further exploration of novel materials, including metal-organic frameworks (MOFs) [10, 99], covalent organic frameworks (COFs) [116, 113], and hybrid nanocomposites, is crucial for expanding the functional repertoire of LB films. Research into mixed systems, such as fatty acid/nanoparticle composites, requires systematic investigation into how component ratios and deposition parameters influence the final structure and properties [3].

Complementary to material and process development is the need for advanced characterization techniques capable of probing the structure and dynamics of both Langmuir monolayers and transferred LB films with high resolution and specificity. While standard methods like surface pressure-area isotherms, ellipsometry, AFM, SEM, TEM, XPS, and FTIR provide valuable information, they often lack the necessary resolution or are unsuitable for *in situ* monitoring or probing specific functionalities within complex environments. Developing and applying novel analytical tools is essential. For instance, *in situ* techniques are crucial for monitoring dynamic pro-

cesses, such as electrochemical reactions occurring at LB-modified battery electrodes or real-time molecular recognition events in biosensors. Advanced X-ray methods performed *in situ* at the air-water interface, such as X-ray Absorption Near Edge Structure (XANES) [194] illustrated schematically in Figure 15, can provide element-specific information about the chemical state and local environment during monolayer compression. Grazing Incidence X-ray Diffraction (GIXD), as shown in Figure 16, allows for the determination of the in-plane and out-of-plane molecular packing and orientation within the Langmuir monolayer under different conditions (e.g., pH, surface pressure), revealing detailed structural arrangements [40]. Brewster Angle Microscopy (BAM), depicted in Figure 17, provides real-time visualization of the monolayer morphology, domain formation, and phase transitions at the air-water interface during compression [80, 138]. These advanced characterization techniques are vital for addressing the challenge of understanding film formation mechanisms, structural details, and phase behavior, guiding the optimization of fabrication processes. Furthermore, developing characterization strategies specifically tailored for LB films within their target application environments (e.g., electrochemical cells, biological media) is critical. Techniques like single-molecule force spectroscopy (SMFS) using LB-functionalized AFM tips [155] also demonstrate the potential for highly specific surface characterization.

Future progress in LB technology will likely benefit significantly from synergistic combinations with other fabrication paradigms. Integrating LB deposition with layer-by-layer (LbL) assembly [106, 195, 108, 107] or self-assembled monolayers (SAMs) can enable the construction of complex, hierarchical nanostructures with tailored functionalities layer by layer. Using LB films as high-resolution masks for subsequent etching processes (e.g., RIE) provides a route to patterned nanostructures like nanopillars or nanochannels [196, 197, 198, 199]. Combining LB deposition with microfluidics could allow for localized deposition or controlled manipulation of the subphase environment. Similarly, interfacing LB techniques with 3D printing or other additive manufacturing methods could open possibilities for creating complex devices incorporating precisely structured ultrathin films.

Ultimately, advancing the field of Langmuir and Langmuir-Blodgett films necessitates a highly interdisciplinary approach. Tackling the multifaceted challenges related to stability, scalability, control, materials, and characterization requires close collaboration between chemists (synthesizing new molecules), physicists (understanding interfacial phenomena), materials sci-

entists (developing and integrating materials), and engineers (designing processes and devices). The increasing trend towards international research collaboration further underscores the global effort required [3]. By fostering such collaborations and pursuing innovative research directions addressing the current limitations, the scientific community can unlock the vast untapped potential of LB technology for creating next-generation materials and devices across diverse fields, including electronics [124, 71], sensing [4, 200, 201], energy storage [19, 18], catalysis, and biomedicine [202, 26, 108].

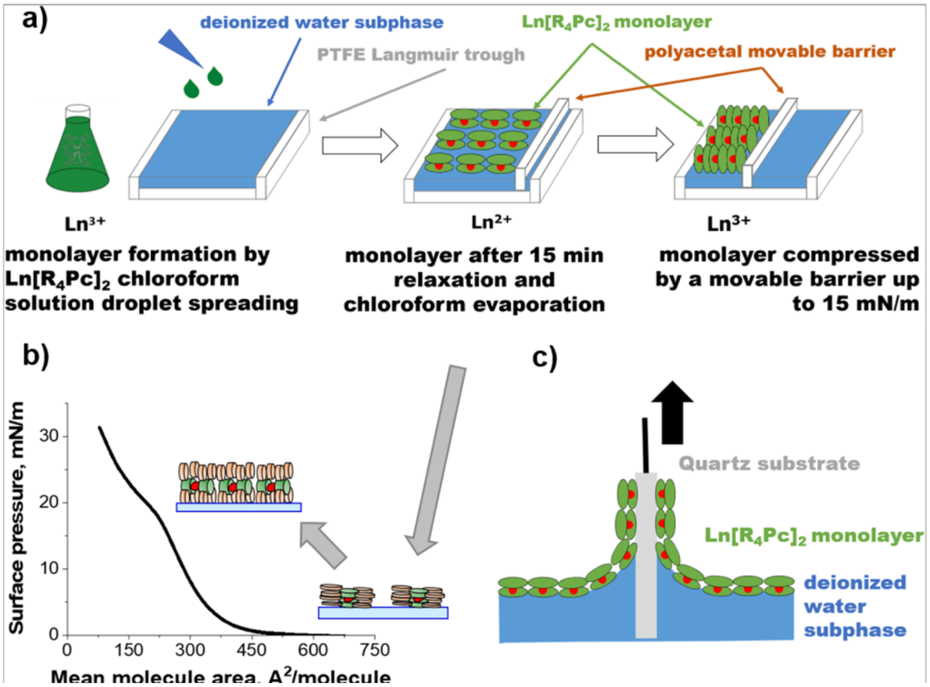

Figure 15: Schematic representations of: a) formation and compression of Langmuir monolayers for UV-Vis and XANES measurements and for subsequent LB films transfer b) corresponding regions on the typical compression isotherm, c) transfers of Langmuir-Blodgett films onto solid substrates. This illustrates the process steps where advanced *in situ* characterization like XANES can provide insights into molecular changes during compression and before/during transfer. Adapted from Arakcheev, A., et al., 2025 [203].

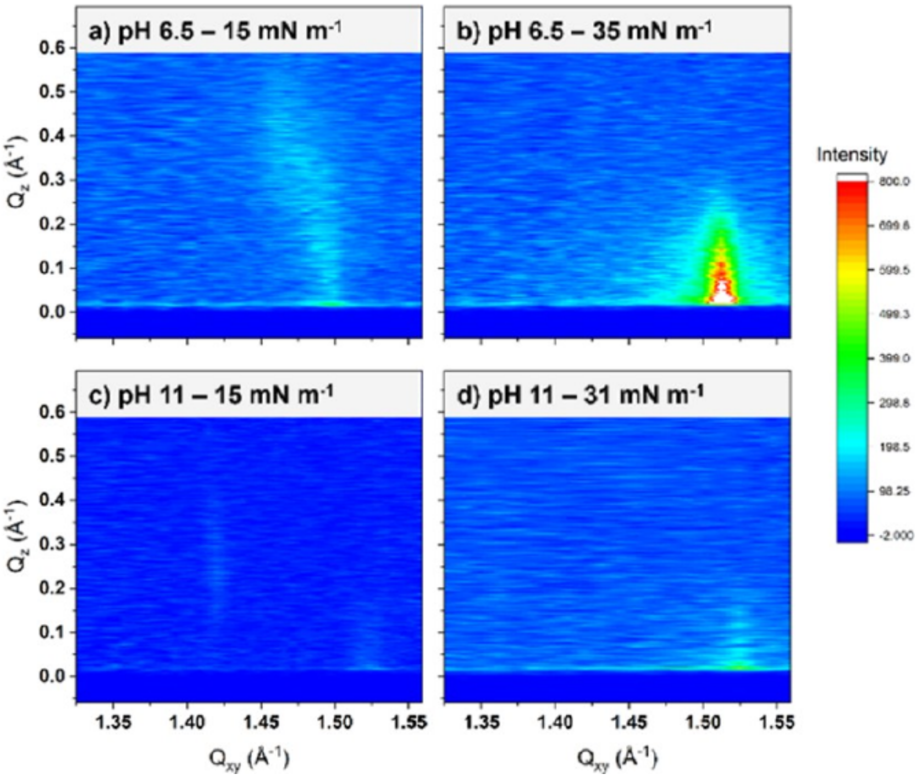

Figure 16: GIXD contour plots of X-ray diffraction intensity as a function of Q,, (in-plane) and Q, (out-of-plane) vector components for an amphiphilic molecule (4b) under different conditions: (a) pH 6.5, 15 mN m^{1}; (b) pH 6.5, 35 mN m^{1}; (c) pH 11, 15 mN m^{1}; and (d) pH 11, 31 mN m^{1}. These plots reveal detailed information about the molecular packing and orientation within the Langmuir monolayer, crucial for understanding structural control. Adapted from Alinia, Z., et al., 2024 [52].

8 Conclusion

This survey has provided a comprehensive overview of Langmuir and Langmuir-Blodgett (LB) film technology, tracing its origins from foundational studies in surface science to its current status as a sophisticated tool for creating highly ordered, ultra-thin molecular films. The fundamental principles underpinning this technique, centered on the formation and manipulation of amphiphilic molecules into Langmuir films at the air-liquid interface using

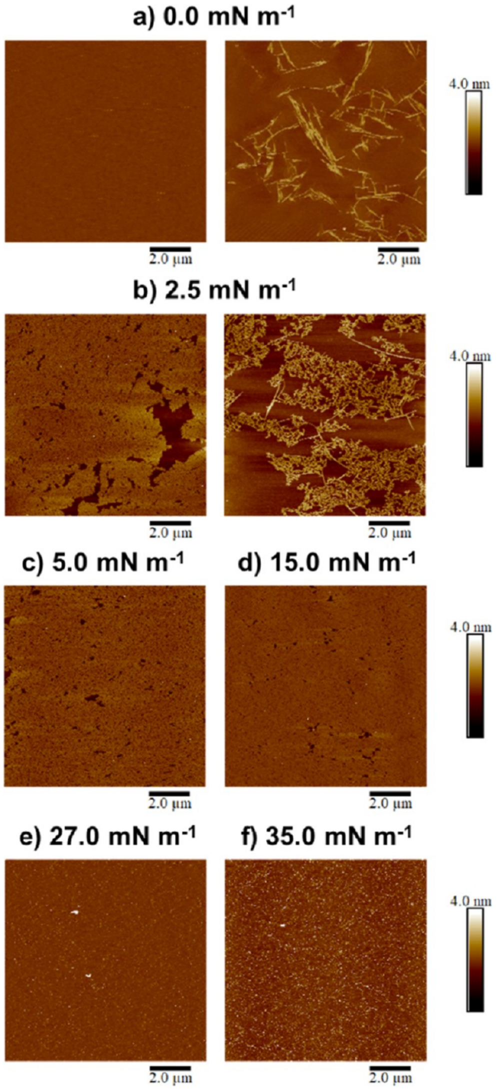

Figure 17: Representative BAM images (620 m X 520 m) of an amphiphilic molecule (4b) at different subphase pH values (4, 6.5, 11) and increasing surface pressures. BAM allows direct visualization of monolayer morphology, domains, and phase transitions at the air-water interface, aiding in the understanding and control of film formation. Adapted from Alinia, Z., et al., 2024 [52].

the Langmuir trough, and their subsequent transfer onto solid substrates as Langmuir-Blodgett films, were detailed. The significance of surface pressure-area isotherms in characterizing the phase behavior of Langmuir films and guiding the deposition process was highlighted, alongside the distinction between vertical (Langmuir-Blodgett) and horizontal (Langmuir-Schaefer) transfer methods.

A key strength of LB technology lies in its remarkable versatility concerning material selection. As discussed throughout this survey, the technique is not limited to classical amphiphiles like fatty acids but extends to a vast array of substances including polymers [126, 189], nanoparticles (such as gold [81, 204, 82], silica, metal oxides), carbon nanomaterials (like graphene oxide [7, 9, 8] and carbon nanotubes), metal-organic frameworks (MOFs) [10, 116], biomolecules (lipids [76, 60], proteins [192, 168], peptides, DNA [6]), and complex hybrid structures. This adaptability allows for the fabrication of films with tailored chemical compositions, structures, and functionalities, enabling precise control at the molecular level [205]. The ability to create mono- or multilayer films with controlled thickness, orientation, and packing density is a defining feature that distinguishes LB films from those produced by other methods like spin coating or drop casting, offering unique advantages for engineering specific material properties.

The importance of meticulous control over fabrication parameters was emphasized, as detailed in Section 4. Factors such as subphase composition (pH, ionic strength, temperature), spreading solvent [206], compression speed, surface pressure during transfer, deposition speed, and substrate properties significantly influence the quality, structure, and morphology of the resulting LB films [207, 133]. Understanding and optimizing these parameters are crucial for achieving reproducible films with minimal defects and desired characteristics [46]. Advanced variations of the LB technique, such as those employing high-temperature troughs (discussed earlier, e.g., [Ariga, Figure 6]) or specialized deposition protocols, further expand the possibilities for fabricating complex nanoarchitectures from less conventional materials.

Characterization techniques, reviewed in Section 5, play a pivotal role in understanding the structure and properties of both Langmuir monolayers at the interface and the transferred LB films on solid substrates. Surface pressure-area isotherms provide fundamental insights into molecular packing and phase transitions. In-situ techniques like Brewster Angle Microscopy (BAM) allow direct visualization of the monolayer morphology at the air-liquid interface. Ex-situ characterization of deposited films employs a suite

of powerful tools, including Atomic Force Microscopy (AFM) and Scanning Electron Microscopy (SEM) for morphological analysis, X-ray Photoelectron Spectroscopy (XPS) and Fourier Transform Infrared Spectroscopy (FTIR) for chemical composition and molecular orientation, and X-ray Diffraction (XRD) or Reflectivity (XRR) for structural analysis and thickness determination. Novel characterization approaches, including the use of elastic tensile modulus isotherms for analyzing soft materials like graphene oxide [Park et al.], demonstrate ongoing innovation in methodologies tailored to the unique aspects of these films.

The survey underscored the broad and growing range of applications for LB films across diverse scientific and technological domains, as explored in Section 6. In sensor technology, the highly ordered structure and large surface area of LB films are exploited to enhance the sensitivity, selectivity, and response time of gas sensors and biosensors. In electrochemistry, LB films serve as precisely controlled electrode modifications for studying interfacial phenomena, enhancing electrocatalytic activity, and developing advanced battery components, including electrodes and functional interlayers. The ability to mimic biological membranes makes LB technology invaluable for creating biomimetic films used in drug delivery studies, biosensing platforms, and fundamental research into membrane biophysics. Furthermore, applications in optics, tribology, and nanotechnology continue to emerge, driven by the unique structural control afforded by the technique.

Despite its numerous advantages and successes, the LB technique faces certain challenges, discussed in detail in Section 7, that currently limit its widespread industrial adoption. These include issues related to the long-term stability of the films, the potential for defects during transfer, the complexity and cost of the equipment, scalability for large-area deposition, and ensuring high reproducibility. Addressing these specific challenges is a key focus of ongoing research. Efforts are directed towards developing more robust film-forming materials, improving deposition techniques to minimize defects, exploring alternative subphases or interfacial systems, and integrating LB methods with other fabrication processes, such as the layer-by-layer assembly or photolithography strategies mentioned earlier in this survey.

In conclusion, Langmuir-Blodgett technology remains a powerful and relevant platform for the bottom-up fabrication of functional thin films with molecular-level precision. Its ability to organize diverse materials into well-defined architectures offers unparalleled control over film structure and properties. Addressing the aforementioned challenges concerning stability, defect

control, cost, scalability, and reproducibility is essential for leveraging LB's precision in demanding future applications, such as advanced electronics, energy storage, biomedical engineering, and environmental monitoring. The concept of nanoarchitectonics, which emphasizes the rational design and construction of functional materials from nanoscale components, finds a strong expression in LB technology. Continued innovation in materials science, instrumentation, and process optimization, coupled with a deeper understanding of interfacial phenomena, promises to overcome existing limitations and unlock new potential, reinforcing the enduring legacy and evolving capabilities of Langmuir and Langmuir-Blodgett films as cornerstones of molecular-level materials engineering.

References

[1] Katsuhiko Ariga et al. "25th Anniversary Article: What Can Be Done with the Langmuir-Blodgett Method? Recent Developments and its Critical Role in Materials Science". In: *Advanced Materials* 25.45 (2013), 6477–6512. ISSN: 1521-4095. DOI: 10.1002/adma.201302283. URL: http://dx.doi.org/10.1002/adma.201302283.

[2] Wenhui Gu et al. "Recent Progress in the Applications of Langmuir–Blodgett Film Technology". In: *Nanomaterials* 14.12 (2024), p. 1039. ISSN: 2079-4991. DOI: 10.3390/nano14121039. URL: http://dx.doi.org/10.3390/nano14121039.

[3] Thi Thao Vu et al. "EVALUATION OF RESEARCH PROGRESS, TRENDS, AND APPLICATIONS OF LANGMUIR-BLODGETT FILMS OF FATTY ACIDS". In: *ChemChemTech* 68.2 (2024), 6–45. ISSN: 0579-2991. DOI: 10.6060/ivkkt.20256802.7002. URL: http://dx.doi.org/10.6060/ivkkt.20256802.7002.

[4] Wenhui Gu et al. "Recent Progress in the Applications of Langmuir–Blodgett Film Technology". In: *Nanomaterials* 14.12 (2024), p. 1039. ISSN: 2079-4991. DOI: 10.3390/nano14121039. URL: http://dx.doi.org/10.3390/nano14121039.

[5] M. S. Kim et al. "Fabricating multifunctional nanoparticle membranes by a fast layer-by-layer Langmuir–Blodgett process: application in lithium–sulfur batteries". In: *Journal of Materials Chemistry A* 4.38

(2016), 14709–14719. ISSN: 2050-7496. DOI: 10.1039/c6ta06018h. URL: http://dx.doi.org/10.1039/c6ta06018h.

[6] Tomohiro Murata et al. "Nanometer-Flat DNA-Featured Thin Films Prepared via Laser Molecular Beam Deposition under High-Vacuum for Selective Methanol Sensing". In: *Bulletin of the Chemical Society of Japan* 96.1 (2022), 29–34. ISSN: 1348-0634. DOI: 10.1246/bcsj.20220303. URL: http://dx.doi.org/10.1246/bcsj.20220303.

[7] Yan Nie et al. "Controlled fabrication of biocompatible graphene oxide Langmuir–Blodgett films by size and surface property manipulation". In: *Journal of Dispersion Science and Technology* 43.12 (2021), 1747–1754. ISSN: 1532-2351. DOI: 10.1080/01932691.2021.1880430. URL: http://dx.doi.org/10.1080/01932691.2021.1880430.

[8] Jongdeok Park et al. "Phase transitions and morphology control of Langmuir-Blodgett (LB) films of graphene oxide". In: *Journal of Colloid and Interface Science* 684 (2025), 215–224. ISSN: 0021-9797. DOI: 10.1016/j.jcis.2025.01.044. URL: http://dx.doi.org/10.1016/j.jcis.2025.01.044.

[9] Thiers M. Uehara et al. "Nanostructured scaffolds containing graphene oxide for nanomedicine applications". In: *Polymers for Advanced Technologies* 33.2 (2021), 591–600. ISSN: 1099-1581. DOI: 10.1002/pat.5541. URL: http://dx.doi.org/10.1002/pat.5541.

[10] Rui Zheng et al. "The Growth Mechanism of a Conductive MOF Thin Film in Spray-based Layer-by-layer Liquid Phase Epitaxy". In: *Angewandte Chemie* 134.43 (2022). ISSN: 1521-3757. DOI: 10.1002/ange.202212797. URL: http://dx.doi.org/10.1002/ange.202212797.

[11] Katsuhiko Ariga. "Nanoarchitectonics Revolution and Evolution: From Small Science to Big Technology". In: *Small Science* 1.1 (2020). ISSN: 2688-4046. DOI: 10.1002/smsc.202000032. URL: http://dx.doi.org/10.1002/smsc.202000032.

[12] Katsuhiko Ariga and Rawil Fakhrullin. "Materials Nanoarchitectonics from Atom to Living Cell: A Method for Everything". In: *Bulletin of the Chemical Society of Japan* 95.5 (2022), 774–795. ISSN: 1348-0634. DOI: 10.1246/bcsj.20220071. URL: http://dx.doi.org/10.1246/bcsj.20220071.

[13] Katsuhiko Ariga and Yusuke Yamauchi. "Nanoarchitectonics from Atom to Life". In: *Chemistry – An Asian Journal* 15.6 (2020), 718–728. ISSN: 1861-471X. DOI: 10.1002/asia.202000106. URL: http://dx.doi.org/10.1002/asia.202000106.

[14] Katsuhiko Ariga et al. "Nanoarchitectonics beyond Self-Assembly: Challenges to Create Bio-Like Hierarchic Organization". In: *Angewandte Chemie International Edition* 59.36 (2020), 15424–15446. ISSN: 1521-3773. DOI: 10.1002/anie.202000802. URL: http://dx.doi.org/10.1002/anie.202000802.

[15] Masami Yanagida, Atsushi Takahara, and Tisato Kajiyama. "Construction of Defect-Diminished Fatty Acid Langmuir–Blodgett Film and Its Optical Waveguide Properties". In: *Bulletin of the Chemical Society of Japan* 72.12 (1999), 2795–2802. ISSN: 1348-0634. DOI: 10.1246/bcsj.72.2795. URL: http://dx.doi.org/10.1246/bcsj.72.2795.

[16] D. Brandl et al. "Vacuum stability of Langmuir—Blodgett films of fatty acids and fatty acid salts". In: *Thin Solid Films* 256.1–2 (1995), 220–225. ISSN: 0040-6090. DOI: 10.1016/0040-6090(94)06312-5. URL: http://dx.doi.org/10.1016/0040-6090(94)06312-5.

[17] Takeshi Hasegawa et al. "Thermal Stability of Metal Stearate LB Films Studied by Infrared Reflection–Absorption Spectroscopy". In: *Chemistry Letters* 19.9 (1990), 1543–1546. ISSN: 1348-0715. DOI: 10.1246/cl.1990.1543. URL: http://dx.doi.org/10.1246/cl.1990.1543.

[18] Vengadesh Periasamy et al. "Langmuir–Blodgett Graphene-Based Films for Algal Biophotovoltaic Fuel Cells". In: *Nanomaterials* 12.5 (2022), p. 840. ISSN: 2079-4991. DOI: 10.3390/nano12050840. URL: http://dx.doi.org/10.3390/nano12050840.

[19] Chen Fang et al. "Recent Applications of Langmuir–Blodgett Technique in Battery Research". In: *ACS Applied Materials amp; Interfaces* 14.2 (2022), 2431–2439. ISSN: 1944-8252. DOI: 10.1021/acsami.1c19064. URL: http://dx.doi.org/10.1021/acsami.1c19064.

[20] Daniel K. Schwartz. "Langmuir-Blodgett film structure". In: *Surface Science Reports* 27.7–8 (1997), 245–334. ISSN: 0167-5729. DOI: 10.1016/s0167-5729(97)00003-4. URL: http://dx.doi.org/10.1016/s0167-5729(97)00003-4.

[21] Katsuhiko Ariga. “Chemistry of Materials Nanoarchitectonics for Two-Dimensional Films: Langmuir–Blodgett, Layer-by-Layer Assembly, and Newcomers”. In: *Chemistry of Materials* 35.14 (2023), 5233–5254. ISSN: 1520-5002. DOI: 10.1021/acs.chemmater.3c01291. URL: http://dx.doi.org/10.1021/acs.chemmater.3c01291.

[22] Masato Ito et al. “Hyper 100 °C Langmuir–Blodgett (Langmuir–Schaefer) Technique for Organized Ultrathin Film of Polymeric Semiconductors”. In: *Langmuir* 38.17 (2021), 5237–5247. ISSN: 1520-5827. DOI: 10.1021/acs.langmuir.1c02596. URL: http://dx.doi.org/10.1021/acs.langmuir.1c02596.

[23] Junzo. Umemura et al. “Quantitative evaluation of molecular orientation in thin Langmuir-Blodgett films by FT-IR transmission and reflection-absorption spectroscopy”. In: *The Journal of Physical Chemistry* 94.1 (1990), 62–67. ISSN: 1541-5740. DOI: 10.1021/j100364a009. URL: http://dx.doi.org/10.1021/j100364a009.

[24] Takeshi Hasegawa, Junzo Umemura, and Tohru Takenaka. “Infrared external reflection study of molecular orientation in thin Langmuir-Blodgett films”. In: *The Journal of Physical Chemistry* 97.35 (1993), 9009–9012. ISSN: 1541-5740. DOI: 10.1021/j100137a028. URL: http://dx.doi.org/10.1021/j100137a028.

[25] Fumiko Kimura, Junzo Umemura, and Tohru Takenaka. “FTIR-ATR studies on Langmuir-Blodgett films of stearic acid with 1-9 monolayers”. In: *Langmuir* 2.1 (1986), 96–101. ISSN: 1520-5827. DOI: 10.1021/la00067a017. URL: http://dx.doi.org/10.1021/la00067a017.

[26] B. Ebert et al. “Development Of Biosensors On The Basis Of Langmuir-Blodgett Films”. In: *Proceedings of the Annual International Conference of the IEEE Engineering in Medicine and Biology Society Volume 13: 1991*. IEEE, 1794–1794. DOI: 10.1109/iembs.1991.684759. URL: http://dx.doi.org/10.1109/iembs.1991.684759.

[27] Cristina Stefaniu, Gerald Brezesinski, and Helmuth Möhwald. “Langmuir monolayers as models to study processes at membrane surfaces”. In: *Advances in Colloid and Interface Science* 208 (2014), 197–213. ISSN: 0001-8686. DOI: 10.1016/j.cis.2014.02.013. URL: http://dx.doi.org/10.1016/j.cis.2014.02.013.

[28] Gerald Brezesinski and Helmuth Möhwald. "Langmuir monolayers to study interactions at model membrane surfaces". In: *Advances in Colloid and Interface Science* 100–102 (2003), 563–584. ISSN: 0001-8686. DOI: 10.1016/s0001-8686(02)00071-4. URL: http://dx.doi.org/10.1016/s0001-8686(02)00071-4.

[29] Iwona Golonka et al. "Evaluation of the Effect of Antibacterial Peptides on Model Monolayers". In: *International Journal of Molecular Sciences* 24.19 (2023), p. 14861. ISSN: 1422-0067. DOI: 10.3390/ijms241914861. URL: http://dx.doi.org/10.3390/ijms241914861.

[30] H M McConnell. "Structures and Transitions in Lipid Monolayers at the Air-Water Interface". In: *Annual Review of Physical Chemistry* 42.1 (1991), 171–195. ISSN: 1545-1593. DOI: 10.1146/annurev.pc.42.100191.001131. URL: http://dx.doi.org/10.1146/annurev.pc.42.100191.001131.

[31] Vladimir M. Kaganer, Helmuth Möhwald, and Pulak Dutta. "Structure and phase transitions in Langmuir monolayers". In: *Reviews of Modern Physics* 71.3 (1999), 779–819. ISSN: 1539-0756. DOI: 10.1103/revmodphys.71.779. URL: http://dx.doi.org/10.1103/revmodphys.71.779.

[32] Christophe Ybert et al. "Collapse of a Monolayer by Three Mechanisms". In: *The Journal of Physical Chemistry B* 106.8 (2002), 2004–2008. ISSN: 1520-5207. DOI: 10.1021/jp013173z. URL: http://dx.doi.org/10.1021/jp013173z.

[33] Laura J. Cote et al. "Graphene oxide as surfactant sheets". In: *Pure and Applied Chemistry* 83.1 (2010), 95–110. ISSN: 0033-4545. DOI: 10.1351/pac-con-10-10-25. URL: http://dx.doi.org/10.1351/pac-con-10-10-25.

[34] J.B. Peng, P. Dutta, and J.B. Ketterson. "Using the transfer of a Langmuir monolayer as a probe of wetting". In: *Thin Solid Films* 159.1–2 (1988), 215–219. ISSN: 0040-6090. DOI: 10.1016/0040-6090(88)90632-3. URL: http://dx.doi.org/10.1016/0040-6090(88)90632-3.

[35] Toshihide Kamata, Junzo Umemura, and Tohru Takenaka. "Structure Study of Langmuir–Blodgett Films of Stearic Acid and Cadmium Stearate Deposited by Different Techniques". In: *Chemistry Letters*

17.7 (1988), 1231–1234. ISSN: 1348-0715. DOI: 10.1246/cl.1988.1231. URL: http://dx.doi.org/10.1246/cl.1988.1231.

[36] S. Garoff et al. "The effects of substrate roughness on ultrathin water films". In: *The Journal of Chemical Physics* 90.12 (1989), 7505–7515. ISSN: 1089-7690. DOI: 10.1063/1.456184. URL: http://dx.doi.org/10.1063/1.456184.

[37] B. Korchowiec and M. Paluch. "Temperature and subphase influence on mixed biosurfactant monolayers formation". In: *Trends in Colloid and Interface Science XI*. Steinkopff, 103–108. ISBN: 9783798510869. DOI: 10.1007/bfb0110971. URL: http://dx.doi.org/10.1007/bfb0110971.

[38] M M Qassime et al. "A studying of subphase temperature and dissolved ascorbic acid concentration influence on the process of Langmuir monolayer formation". In: *Journal of Physics: Conference Series* 1124 (2018), p. 031010. ISSN: 1742-6596. DOI: 10.1088/1742-6596/1124/3/031010. URL: http://dx.doi.org/10.1088/1742-6596/1124/3/031010.

[39] Adrien Sthoer et al. "La3+ and Y3+ interactions with the carboxylic acid moiety at the liquid/vapour interface: identification of binding complexes, charge reversal, and detection limits." In: (2021). DOI: 10.26434/chemrxiv-2021-28p74. URL: http://dx.doi.org/10.26434/chemrxiv-2021-28p74.

[40] Th. Geue et al. "Investigations of pH-dependent domain structure of lead arachidate Langmuir-Blodgett films by means of x-ray specular and diffuse scattering and atomic force microscopy". In: *The Journal of Chemical Physics* 110.16 (1999), 8104–8111. ISSN: 1089-7690. DOI: 10.1063/1.478713. URL: http://dx.doi.org/10.1063/1.478713.

[41] Naveen Kumar et al. "Salt Dependent Stability of Stearic Acid Langmuir–Blodgett Films Exposed to Aqueous Electrolytes". In: *Langmuir* 29.17 (2013), 5150–5159. ISSN: 1520-5827. DOI: 10.1021/la400615j. URL: http://dx.doi.org/10.1021/la400615j.

[42] Junya Adachi et al. "Coordination Amphiphile: Design of Planar-Coordinated Platinum Complexes for Monolayer Formation at an Air-Water Interface Based on Ligand Characteristics and Molecular Topology". In: *Bulletin of the Chemical Society of Japan* 95.6 (2022),

889–897. ISSN: 1348-0634. DOI: 10.1246/bcsj.20220086. URL: http://dx.doi.org/10.1246/bcsj.20220086.

[43] Iwona Golonka et al. "Effect of Newly Synthesized Structures of Peptides on the Stability of the Monolayers Formed". In: *International Journal of Molecular Sciences* 24.5 (2023), p. 4318. ISSN: 1422-0067. DOI: 10.3390/ijms24054318. URL: http://dx.doi.org/10.3390/ijms24054318.

[44] Akihiko Yamagishi et al. "Langmuir-Blodgett Films of Chiral Perfluorinated Gelators: Effects of Chirality and Chain Length on Two-Dimensional Behavior". In: *Bulletin of the Chemical Society of Japan* 94.2 (2020), 377–381. ISSN: 1348-0634. DOI: 10.1246/bcsj.20200245. URL: http://dx.doi.org/10.1246/bcsj.20200245.

[45] H M McConnell and Y B Bazaliy. "Lipid monolayer image dipoles." In: *Proceedings of the National Academy of Sciences* 92.19 (1995), 8823–8825. ISSN: 1091-6490. DOI: 10.1073/pnas.92.19.8823. URL: http://dx.doi.org/10.1073/pnas.92.19.8823.

[46] Dawn Y. Takamoto et al. "Stable Ordering in Langmuir-Blodgett Films". In: *Science* 293.5533 (2001), 1292–1295. ISSN: 1095-9203. DOI: 10.1126/science.1060018. URL: http://dx.doi.org/10.1126/science.1060018.

[47] Andreas T. Haedler et al. "Controlling the -Stacking Behavior of Pyrene Derivatives: Influence of H-Bonding and Steric Effects in Different States of Aggregation". In: *ChemPhysChem* 14.9 (2013), 1818–1829. ISSN: 1439-7641. DOI: 10.1002/cphc.201300242. URL: http://dx.doi.org/10.1002/cphc.201300242.

[48] Kenya Adachi, Takashi Hirose, and Kenji Matsuda. "The polymorphism of porphyrin 2D assemblies at the liquid–graphite interface: the effect of a polar solvent additive and a flexible spacer on the face-on and edge-on type molecular arrangements". In: *Chemical Communications* 55.60 (2019), 8836–8839. ISSN: 1364-548X. DOI: 10.1039/c9cc02579k. URL: http://dx.doi.org/10.1039/c9cc02579k.

[49] Yunshen Zhou et al. "Observation of co-existence of 'face-on' and 'edge-on' stacking styles in a porphyrin monolayer". In: *Chemical Physics Letters* 403.1–3 (2005), 140–145. ISSN: 0009-2614. DOI: 10.1016/j.cplett.2005.01.006. URL: http://dx.doi.org/10.1016/j.cplett.2005.01.006.

[50] Mohamed Ahmida et al. "Face- and edge-on orientations of octa-acid and -alcohol substituted tetraazaporphyrins in Langmuir and Langmuir–Blodgett monolayers". In: *Soft Matter* 9.3 (2013), 811–819. ISSN: 1744-6848. DOI: 10.1039/c2sm27064a. URL: http://dx.doi.org/10.1039/c2sm27064a.

[51] Saqib Javaid and Geunsik Lee. "The impact of molecular orientation on carrier transfer characteristics at a phthalocyanine and halide perovskite interface". In: *RSC Advances* 11.50 (2021), 31776–31782. ISSN: 2046-2069. DOI: 10.1039/d1ra05909b. URL: http://dx.doi.org/10.1039/d1ra05909b.

[52] Zahra Alinia et al. "Amphiphilicity of Tetraazaporphyrins Containing Four Terminal Carboxylic Acid and Four Alkyl Groups Promotes Face-On Orientation in Langmuir Films". In: *Langmuir* 40.50 (2024). PMID: 39623767, pp. 26672–26684. DOI: 10.1021/acs.langmuir.4c03800. eprint: https://doi.org/10.1021/acs.langmuir.4c03800. URL: https://doi.org/10.1021/acs.langmuir.4c03800.

[53] Katharine B. Blodgett. "MONOMOLECULAR FILMS OF FATTY ACIDS ON GLASS". In: *Journal of the American Chemical Society* 56.2 (1934), 495–495. ISSN: 1520-5126. DOI: 10.1021/ja01317a513. URL: http://dx.doi.org/10.1021/ja01317a513.

[54] Viktoryia Akulova et al. "Formation and tribological properties of octacosanoic acid monomolecular Langmuir–Blodgett films". In: *Soft Materials* 20.2 (2021), 161–167. ISSN: 1539-4468. DOI: 10.1080/1539445x.2021.1933034. URL: http://dx.doi.org/10.1080/1539445x.2021.1933034.

[55] Andreas Santamaria et al. "Investigation on the relationship between lipid composition and structure in model membranes composed of extracted natural phospholipids". In: (2022). DOI: 10.26434/chemrxiv-2022-mrfmx. URL: http://dx.doi.org/10.26434/chemrxiv-2022-mrfmx.

[56] Katarzyna Hac-Wydro and Paweł Wydro. "The influence of fatty acids on model cholesterol/phospholipid membranes". In: *Chemistry and Physics of Lipids* 150.1 (2007), 66–81. ISSN: 0009-3084. DOI: 10.1016/j.chemphyslip.2007.06.213. URL: http://dx.doi.org/10.1016/j.chemphyslip.2007.06.213.

[57] J.B Peng, G.T Barnes, and I.R Gentle. “The structures of Langmuir–Blodgett films of fatty acids and their salts”. In: *Advances in Colloid and Interface Science* 91.2 (2001), 163–219. ISSN: 0001-8686. DOI: 10.1016/s0001-8686(99)00031-7. URL: http://dx.doi.org/10.1016/s0001-8686(99)00031-7.

[58] Juan J. Giner-Casares, Gerald Brezesinski, and Helmuth Möhwald. “Langmuir monolayers as unique physical models”. In: *Current Opinion in Colloid amp; Interface Science* 19.3 (2014), 176–182. ISSN: 1359-0294. DOI: 10.1016/j.cocis.2013.07.006. URL: http://dx.doi.org/10.1016/j.cocis.2013.07.006.

[59] Thatyane M. Nobre et al. “Interactions of bioactive molecules amp; nanomaterials with Langmuir monolayers as cell membrane models”. In: *Thin Solid Films* 593 (2015), 158–188. ISSN: 0040-6090. DOI: 10.1016/j.tsf.2015.09.047. URL: http://dx.doi.org/10.1016/j.tsf.2015.09.047.

[60] Klaudia Szafran et al. “Surface Properties of the Polyethylene Terephthalate (PET) Substrate Modified with the Phospholipid-Polypeptide-Antioxidant Films: Design of Functional Biocoatings”. In: *Pharmaceutics* 14.12 (2022), p. 2815. ISSN: 1999-4923. DOI: 10.3390/pharmaceutics14122815. URL: http://dx.doi.org/10.3390/pharmaceutics14122815.

[61] Victoria M. Katata et al. “The Role of Cholesterol in the Interaction of the Lipid Monolayer with the Endocrine Disruptor Bisphenol-A”. In: *Membranes* 12.8 (2022), p. 729. ISSN: 2077-0375. DOI: 10.3390/membranes12080729. URL: http://dx.doi.org/10.3390/membranes12080729.

[62] Justyna Mildner, Anita Wnetrzak, and Patrycja Dynarowicz-Latka. “Cholesterol and Cardiolipin Importance in Local Anesthetics–Membrane Interactions: The Langmuir Monolayer Study”. In: *The Journal of Membrane Biology* 252.1 (2018), 31–39. ISSN: 1432-1424. DOI: 10.1007/s00232-018-0055-6. URL: http://dx.doi.org/10.1007/s00232-018-0055-6.

[63] G. Brezesinski et al. “Morphology and structures in double-, triple- and quadruple-chain phospholipid monolayers at the air/water interface”. In: *Trends in Colloid and Interface Science IX*. Steinkopff, 255–262. ISBN: 9783798510319. DOI: 10.1007/bfb0115249. URL: http://dx.doi.org/10.1007/bfb0115249.

[64] Agnès P. Girard-Egrot and Loïc J. Blum. "Langmuir-Blodgett Technique for Synthesis of Biomimetic Lipid Membranes". In: *Nanobiotechnology of Biomimetic Membranes*. Springer US, 23–74. ISBN: 9780387377407. DOI: 10.1007/0-387-37740-9_2. URL: http://dx.doi.org/10.1007/0-387-37740-9_2.

[65] M. R. Porter. *Handbook Surfactants*. Springer US, 1991. ISBN: 9781461536765. DOI: 10.1007/978-1-4615-3676-5. URL: http://dx.doi.org/10.1007/978-1-4615-3676-5.

[66] Kedar Joshi and James F. Gilchrist. "Effect of added surfactant on convective assembly of monosized microspheres". In: *Applied Physics Letters* 116.8 (2020). ISSN: 1077-3118. DOI: 10.1063/1.5139962. URL: http://dx.doi.org/10.1063/1.5139962.

[67] Srikant Kumar Singh and Matthew F. Paige. "Effect of a fluorinated surfactant on Langmuir monolayer properties of minimal-linker gemini surfactants". In: *Colloids and Surfaces A: Physicochemical and Engineering Aspects* 700 (2024), p. 134767. ISSN: 0927-7757. DOI: 10.1016/j.colsurfa.2024.134767. URL: http://dx.doi.org/10.1016/j.colsurfa.2024.134767.

[68] Richard A. Campbell et al. "Structure of surfactant and phospholipid monolayers at the air/water interface modeled from neutron reflectivity data". In: *Journal of Colloid and Interface Science* 531 (2018), 98–108. ISSN: 0021-9797. DOI: 10.1016/j.jcis.2018.07.022. URL: http://dx.doi.org/10.1016/j.jcis.2018.07.022.

[69] Bernd Lühmann, Heino Finkelmann, and Günther Rehage. "Structure and liquid crystalline phases of amphiphilic side chain polymers in aqueous solution". In: *Die Angewandte Makromolekulare Chemie* 123.1 (1984), 217–227. ISSN: 1522-9505. DOI: 10.1002/apmc.1984.051230109. URL: http://dx.doi.org/10.1002/apmc.1984.051230109.

[70] Huie Zhu, Masaya Mitsuishi, and Tokuji Miyashita. "Facile Preparation of Highly Oriented Poly(vinylidene fluoride) Langmuir–Blodgett Nanofilms Assisted by Amphiphilic Polymer Nanosheets". In: *Macromolecules* 45.22 (2012), 9076–9084. ISSN: 1520-5835. DOI: 10.1021/ma301711g. URL: http://dx.doi.org/10.1021/ma301711g.

[71] J. H. Cheung, R. B. Rosner, and M. F. Rubner. “New Strategies for Preparing Electrically Conductive Langmuir-Blodgett Films”. In: *MRS Proceedings* 247 (1992). ISSN: 1946-4274. DOI: 10.1557/proc-247-859. URL: http://dx.doi.org/10.1557/proc-247-859.

[72] J.H. Cheung and M.F. Rubner. “Fabrication of electrically conductive Langmuir-Blodgett multilayer films of polyaniline”. In: *Thin Solid Films* 244.1–2 (1994), 990–994. ISSN: 0040-6090. DOI: 10.1016/0040-6090(94)90617-3. URL: http://dx.doi.org/10.1016/0040-6090(94)90617-3.

[73] Gurpreet Kaur Bhullar, Ramneek Kaur, and K.K. Raina. “Growth, Morphology, and Electrical Characterization of Polyaniline–ZnO Nanocomposite Langmuir–Blodgett Thin Films”. In: *Journal of Electronic Materials* 44.10 (2015), 3422–3429. ISSN: 1543-186X. DOI: 10.1007/s11664-015-3868-4. URL: http://dx.doi.org/10.1007/s11664-015-3868-4.

[74] Andrei Sakai, Laura O. Péres, and Luciano Caseli. “Langmuir and Langmuir-Blodgett films of Cl-PPV mixed with stearic acid: implication of the morphology on the surface and spectroscopy properties”. In: *Colloid and Polymer Science* 293.3 (2014), 883–890. ISSN: 1435-1536. DOI: 10.1007/s00396-014-3477-4. URL: http://dx.doi.org/10.1007/s00396-014-3477-4.

[75] Felipe Tejada Araujo, Laura Oliveira Peres, and Luciano Caseli. “Conjugated Polymers Blended with Lipids and Galactosidase as Langmuir–Blodgett Films To Control the Biosensing Properties of Nanostructured Surfaces”. In: *Langmuir* 35.22 (2019), 7294–7303. ISSN: 1520-5827. DOI: 10.1021/acs.langmuir.9b00536. URL: http://dx.doi.org/10.1021/acs.langmuir.9b00536.

[76] Olga Zamyshlyayeva et al. “The surface behavior of mixed monolayers and LB films of betulin-containing polymers with lipids at the air-Ce3+ aqueous solution interface and on solid substrate”. In: *Polymer Bulletin* 82.6 (2024), 1799–1823. ISSN: 1436-2449. DOI: 10.1007/s00289-024-05573-7. URL: http://dx.doi.org/10.1007/s00289-024-05573-7.

[77] Katsuhiko Ariga and Jonathan P. Hill. “Monolayers at air-water interfaces: from origins-of-life to nanotechnology”. In: *The Chemical Record*

11.4 (2011), 199–211. ISSN: 1528-0691. DOI: 10.1002/tcr.201100004. URL: http://dx.doi.org/10.1002/tcr.201100004.

[78] Hanming Ding et al. “A physical insight into the gas-sensing properties of copper (II) tetra-(tert-butyl)-5,10,15,20-tetraazaporphyrin Langmuir–Blodgett films”. In: *Thin Solid Films* 379.1–2 (2000), 279–286. ISSN: 0040-6090. DOI: 10.1016/s0040-6090(00)01560-1. URL: http://dx.doi.org/10.1016/s0040-6090(00)01560-1.

[79] W. Rabinovitch, R. F. Robertson, and S. G. Mason. “RELAXATION OF SURFACE PRESSURE AND COLLAPSE OF UNIMOLECULAR FILMS OF STEARIC ACID”. In: *Canadian Journal of Chemistry* 38.10 (1960), 1881–1890. ISSN: 1480-3291. DOI: 10.1139/v60-253. URL: http://dx.doi.org/10.1139/v60-253.

[80] Florencio Peñacorada et al. “Brewster angle microscopy and surface potential measurements of Langmuir–Blodgett films of zinc tri(tert-butyl)-4-sulphophthalocyanine”. In: *Applied Surface Science* 246.4 (2005), 425–429. ISSN: 0169-4332. DOI: 10.1016/j.apsusc.2004.11.047. URL: http://dx.doi.org/10.1016/j.apsusc.2004.11.047.

[81] Michal Swierczewski and Thomas Bürgi. “Langmuir and Langmuir–Blodgett Films of Gold and Silver Nanoparticles”. In: *Langmuir* 39.6 (2023), 2135–2151. ISSN: 1520-5827. DOI: 10.1021/acs.langmuir.2c02715. URL: http://dx.doi.org/10.1021/acs.langmuir.2c02715.

[82] S. Paul et al. “LangmuirBlodgett Film Deposition of Metallic Nanoparticles and Their Application to Electronic Memory Structures”. In: *Nano Letters* 3.4 (2003), 533–536. ISSN: 1530-6992. DOI: 10.1021/nl034008t. URL: http://dx.doi.org/10.1021/nl034008t.

[83] Muthukumar Divagar, Nagamony Ponpandian, and Chinnuswamy Viswanathan. “Langmuir-Blodgett deposited Na3V2(PO4)3-MnO2 nanocomposite thin film electrodes for hybrid energy storage application”. In: *Materials Science and Engineering: B* 270 (2021), p. 115229. ISSN: 0921-5107. DOI: 10.1016/j.mseb.2021.115229. URL: http://dx.doi.org/10.1016/j.mseb.2021.115229.

[84] Shipra Solanki et al. “Langmuir–Blodgett Nanoassemblies of the MoS2–Au Composite at the Air–Water Interface for Dengue Detection”. In: *ACS Applied Materials amp; Interfaces* 10.3 (2018), 3020–3028. ISSN: 1944-8252. DOI: 10.1021/acsami.7b14391. URL: http://dx.doi.org/10.1021/acsami.7b14391.

[85] Ke-Hsuan Wang et al. "Architecture effects of glucose oxidase/Au nanoparticle composite Langmuir-Blodgett films on glucose sensing performance". In: *Applied Surface Science* 366 (2016), 202–209. ISSN: 0169-4332. DOI: 10.1016/j.apsusc.2016.01.047. URL: http://dx.doi.org/10.1016/j.apsusc.2016.01.047.

[86] Ilya Gorbachev et al. "Langmuir–Blodgett Films with Immobilized Glucose Oxidase Enzyme Molecules for Acoustic Glucose Sensor Application". In: *Sensors* 23.11 (2023), p. 5290. ISSN: 1424-8220. DOI: 10.3390/s23115290. URL: http://dx.doi.org/10.3390/s23115290.

[87] Fábio A. Scholl et al. "Exploring Langmuir-Blodgett films with phospholipid-graphene oxide/MnO2 as a hybrid nanostructured interface for supercapacitor applications". In: *Colloids and Surfaces A: Physicochemical and Engineering Aspects* 664 (2023), p. 131128. ISSN: 0927-7757. DOI: 10.1016/j.colsurfa.2023.131128. URL: http://dx.doi.org/10.1016/j.colsurfa.2023.131128.

[88] Sukhananazerin Abdulla and Biji Pullithadathil. "Unidirectional Langmuir–Blodgett-Mediated Alignment of Polyaniline-Functionalized Multiwalled Carbon Nanotubes for NH3 Gas Sensor Applications". In: *Langmuir* 36.39 (2020), 11618–11628. ISSN: 1520-5827. DOI: 10.1021/acs.langmuir.0c02200. URL: http://dx.doi.org/10.1021/acs.langmuir.0c02200.

[89] Hyeri Kim et al. "Spontaneous hybrids of graphene and carbon nanotube arrays at the liquid–gas interface for Li-ion battery anodes". In: *Chemical Communications* 54.41 (2018), 5229–5232. ISSN: 1364-548X. DOI: 10.1039/c8cc02148a. URL: http://dx.doi.org/10.1039/c8cc02148a.

[90] Alexander Holm et al. "Langmuir–Blodgett Deposition of Graphene Oxide—Identifying Marangoni Flow as a Process that Fundamentally Limits Deposition Control". In: *Langmuir* 34.33 (2018), 9683–9691. ISSN: 1520-5827. DOI: 10.1021/acs.langmuir.8b00777. URL: http://dx.doi.org/10.1021/acs.langmuir.8b00777.

[91] Sriramprabha Ramasamy, Ponpandian Nagamony, and Viswanathan Chinnuswamy. "Self-assembled SnO2/reduced graphene oxide nanocomposites via Langmuir-Blodgett technique as anode materials for Li-ion batteries". In: *Materials Letters* 218 (2018), 295–298. ISSN: 0167-577X.

DOI: 10.1016/j.matlet.2018.01.177. URL: http://dx.doi.org/10.1016/j.matlet.2018.01.177.

[92] Ali Hossain Khan et al. "Two-Dimensional (2D) Nanomaterials towards Electrochemical Nanoarchitectonics in Energy-Related Applications". In: *Bulletin of the Chemical Society of Japan* 90.6 (2017), 627–648. ISSN: 1348-0634. DOI: 10.1246/bcsj.20170043. URL: http://dx.doi.org/10.1246/bcsj.20170043.

[93] María Mercedes Velázquez et al. "Langmuir-Blodgett Methodology: A Versatile Technique to Build 2D Material Films". In: *Two-dimensional Materials - Synthesis, Characterization and Potential Applications*. InTech, 2016. ISBN: 9789535125556. DOI: 10.5772/63495. URL: http://dx.doi.org/10.5772/63495.

[94] Jin Young Park and Rigoberto C. Advincula. "Nanostructuring polymers, colloids, and nanomaterials at the air–water interface through Langmuir and Langmuir–Blodgett techniques". In: *Soft Matter* 7.21 (2011), p. 9829. ISSN: 1744-6848. DOI: 10.1039/c1sm05750b. URL: http://dx.doi.org/10.1039/c1sm05750b.

[95] Somsubhra Saha et al. "Self-assembly of silver nanocolloids in the Langmuir–Blodgett Film of stearic acid: Evidence of an efficient SERS sensing platform". In: *Journal of Raman Spectroscopy* 47.2 (2015), 168–176. ISSN: 1097-4555. DOI: 10.1002/jrs.4771. URL: http://dx.doi.org/10.1002/jrs.4771.

[96] Hitoshi Miyasaka. "Charge Manipulation in Metal–Organic Frameworks: Toward Designer Functional Molecular Materials". In: *Bulletin of the Chemical Society of Japan* 94.12 (2021), 2929–2955. ISSN: 1348-0634. DOI: 10.1246/bcsj.20210277. URL: http://dx.doi.org/10.1246/bcsj.20210277.

[97] Haruna Mabuchi et al. "Covalent Organic Frameworks: Cutting-Edge Materials for Carbon Dioxide Capture and Water Harvesting from Air". In: *Chemistry – A European Journal* 30.6 (2024). ISSN: 1521-3765. DOI: 10.1002/chem.202303474. URL: http://dx.doi.org/10.1002/chem.202303474.

[98] Kaushik Dey et al. "Selective Molecular Separation by Interfacially Crystallized Covalent Organic Framework Thin Films". In: *Journal of the American Chemical Society* 139.37 (2017), 13083–13091. ISSN:

1520-5126. DOI: 10.1021/jacs.7b06640. URL: http://dx.doi.org/10.1021/jacs.7b06640.

[99] Takashi Ohata et al. "Influence of the Solvent on the Assembly of Ni3(hexaiminotriphenylene)2 Metal–Organic Framework Nanosheets at the Air/Liquid Interface". In: *Bulletin of the Chemical Society of Japan* 96.3 (2023), 274–282. ISSN: 1348-0634. DOI: 10.1246/bcsj.20220283. URL: http://dx.doi.org/10.1246/bcsj.20220283.

[100] Rie Makiura and Oleg Konovalov. "Interfacial growth of large-area single-layer metal-organic framework nanosheets". In: *Scientific Reports* 3.1 (2013). ISSN: 2045-2322. DOI: 10.1038/srep02506. URL: http://dx.doi.org/10.1038/srep02506.

[101] Kazuaki Tachimoto et al. "Assembling Triphenylene-Based Metal–Organic Framework Nanosheets at the Air/Liquid Interface: Modification by Tuning the Spread Solution Concentration". In: *Langmuir* 39.26 (2023), 8952–8962. ISSN: 1520-5827. DOI: 10.1021/acs.langmuir.2c02685. URL: http://dx.doi.org/10.1021/acs.langmuir.2c02685.

[102] Renhao Dong et al. "Large-Area, Free-Standing, Two-Dimensional Supramolecular Polymer Single-Layer Sheets for Highly Efficient Electrocatalytic Hydrogen Evolution". In: *Angewandte Chemie International Edition* 54.41 (2015), 12058–12063. ISSN: 1521-3773. DOI: 10.1002/anie.201506048. URL: http://dx.doi.org/10.1002/anie.201506048.

[103] Tong Zhang et al. "Fewer-layer conductive metal-organic Langmuir-Blodgett films as electrocatalysts enable an ultralow detection limit of H2O2". In: *Applied Surface Science* 539 (2021), p. 148255. ISSN: 0169-4332. DOI: 10.1016/j.apsusc.2020.148255. URL: http://dx.doi.org/10.1016/j.apsusc.2020.148255.

[104] Maria Rosaria di Nunzio et al. "Interrogating the Behaviour of a Styryl Dye Interacting with a Mesoscopic 2D-MOF and Its Luminescent Vapochromic Sensing". In: *International Journal of Molecular Sciences* 23.1 (2021), p. 330. ISSN: 1422-0067. DOI: 10.3390/ijms23010330. URL: http://dx.doi.org/10.3390/ijms23010330.

[105] Subhadip Goswami et al. "Toward Ideal Metal–Organic Framework Thin-Film Growth via Automated Layer-by-Layer Deposition: Examples Based on Perylene Diimide Linkers". In: *Chemistry of Materials* 34.21 (2022), 9446–9454. ISSN: 1520-5002. DOI: 10.1021/acs.

chemmater.2c01753. URL: http://dx.doi.org/10.1021/acs.chemmater.2c01753.

[106] Katsuhiko Ariga et al. “Layer-by-layer Nanoarchitectonics: Invention, Innovation, and Evolution”. In: *Chemistry Letters* 43.1 (2013), 36–68. ISSN: 1348-0715. DOI: 10.1246/cl.130987. URL: http://dx.doi.org/10.1246/cl.130987.

[107] Omar Azzaroni et al. “Field-effect transistors engineered via solution-based layer-by-layer nanoarchitectonics”. In: *Nanotechnology* 34.47 (2023), p. 472001. ISSN: 1361-6528. DOI: 10.1088/1361-6528/acef26. URL: http://dx.doi.org/10.1088/1361-6528/acef26.

[108] João Borges et al. “Recent Developments in Layer-by-Layer Assembly for Drug Delivery and Tissue Engineering Applications”. In: *Advanced Healthcare Materials* 13.8 (2024). ISSN: 2192-2659. DOI: 10.1002/adhm.202302713. URL: http://dx.doi.org/10.1002/adhm.202302713.

[109] Ning Huang et al. “Multiple-component covalent organic frameworks”. In: *Nature Communications* 7.1 (2016). ISSN: 2041-1723. DOI: 10.1038/ncomms12325. URL: http://dx.doi.org/10.1038/ncomms12325.

[110] Xiaohan Wang et al. “Triple Isomerism in 3D Covalent Organic Frameworks”. In: *Journal of the American Chemical Society* 146.3 (2024), 1832–1838. ISSN: 1520-5126. DOI: 10.1021/jacs.3c13863. URL: http://dx.doi.org/10.1021/jacs.3c13863.

[111] Bing Zhang et al. “Reticular Synthesis of Multinary Covalent Organic Frameworks”. In: *Journal of the American Chemical Society* 141.29 (2019), 11420–11424. ISSN: 1520-5126. DOI: 10.1021/jacs.9b05626. URL: http://dx.doi.org/10.1021/jacs.9b05626.

[112] Youn Charles-Blin et al. “Salt-Assisted Pyrolysis of Covalent Organic Framework for Controlled Active Nitrogen Functionalities for Oxygen Reduction Reaction”. In: *Bulletin of the Chemical Society of Japan* 95.6 (2022), 972–977. ISSN: 1348-0634. DOI: 10.1246/bcsj.20220094. URL: http://dx.doi.org/10.1246/bcsj.20220094.

[113] Deng Zhou et al. “Synthesis of CC Bonded Two-Dimensional Conjugated Covalent Organic Framework Films by Suzuki Polymerization on a Liquid–Liquid Interface”. In: *Angewandte Chemie* 131.5 (2019),

1390–1395. ISSN: 1521-3757. DOI: 10.1002/ange.201811399. URL: http://dx.doi.org/10.1002/ange.201811399.

[114] Arkaprabha Giri et al. “Transformation of an Imine Cage to a Covalent Organic Framework Film at the Liquid–Liquid Interface”. In: *Angewandte Chemie* 135.23 (2023). ISSN: 1521-3757. DOI: 10.1002/ange.202219083. URL: http://dx.doi.org/10.1002/ange.202219083.

[115] Michio Matsumoto et al. “Lewis-Acid-Catalyzed Interfacial Polymerization of Covalent Organic Framework Films”. In: *Chem* 4.2 (2018), 308–317. ISSN: 2451-9294. DOI: 10.1016/j.chempr.2017.12.011. URL: http://dx.doi.org/10.1016/j.chempr.2017.12.011.

[116] Bin Bai, Dong Wang, and Li-Jun Wan. “Synthesis of Covalent Organic Framework Films at Interfaces”. In: *Bulletin of the Chemical Society of Japan* 94.3 (2021), 1090–1098. ISSN: 1348-0634. DOI: 10.1246/bcsj.20200391. URL: http://dx.doi.org/10.1246/bcsj.20200391.

[117] Jingwen Song et al. “Fullerphene Nanosheets: A Bottom-Up 2D Material for Single-Carbon-Atom-Level Molecular Discrimination”. In: *Advanced Materials Interfaces* 9.11 (2022). ISSN: 2196-7350. DOI: 10.1002/admi.202102241. URL: http://dx.doi.org/10.1002/admi.202102241.

[118] Donghui Guo et al. “Active sites of nitrogen-doped carbon materials for oxygen reduction reaction clarified using model catalysts”. In: *Science* 351.6271 (2016), 361–365. ISSN: 1095-9203. DOI: 10.1126/science.aad0832. URL: http://dx.doi.org/10.1126/science.aad0832.

[119] Kazushi Kinbara and Takuzo Aida. “Toward Intelligent Molecular Machines: Directed Motions of Biological and Artificial Molecules and Assemblies”. In: *ChemInform* 36.28 (2005). ISSN: 1522-2667. DOI: 10.1002/chin.200528270. URL: http://dx.doi.org/10.1002/chin.200528270.

[120] Katsuhiko Ariga et al. “Nanoarchitectonics for Dynamic Functional Materials from Atomic-/Molecular-Level Manipulation to Macroscopic Action”. In: *Advanced Materials* 28.6 (2015), 1251–1286. ISSN: 1521-4095. DOI: 10.1002/adma.201502545. URL: http://dx.doi.org/10.1002/adma.201502545.

[121] Katsuhiko Ariga. “Mechano-Nanoarchitectonics: Design and Function”. In: *Small Methods* 6.5 (2022). ISSN: 2366-9608. DOI: 10.1002/smtd.202101577. URL: http://dx.doi.org/10.1002/smtd.202101577.

[122] Katsuhiko Ariga. “The evolution of molecular machines through interfacial nanoarchitectonics: from toys to tools”. In: *Chemical Science* 11.39 (2020), 10594–10604. ISSN: 2041-6539. DOI: 10.1039/d0sc03164j. URL: http://dx.doi.org/10.1039/d0sc03164j.

[123] Katsuhiko Ariga, Jingwen Song, and Kohsaku Kawakami. “Molecular machines working at interfaces: physics, chemistry, evolution and nanoarchitectonics”. In: *Physical Chemistry Chemical Physics* 26.18 (2024), 13532–13560. ISSN: 1463-9084. DOI: 10.1039/d4cp00724g. URL: http://dx.doi.org/10.1039/d4cp00724g.

[124] Heng Lin et al. “Controllable-assembled functional monolayers by the Langmuir–Blodgett technique for optoelectronic applications”. In: *Journal of Materials Chemistry C* 12.4 (2024), 1177–1210. ISSN: 2050-7534. DOI: 10.1039/d3tc03591c. URL: http://dx.doi.org/10.1039/d3tc03591c.

[125] Yunzhe Jiang et al. “Nanoporous Films with Oriented Arrays of Molecular Motors for Photoswitching the Guest Adsorption and Diffusion”. In: *Angewandte Chemie International Edition* 62.3 (2022). ISSN: 1521-3773. DOI: 10.1002/anie.202214202. URL: http://dx.doi.org/10.1002/anie.202214202.

[126] Gero Decher. “Fuzzy Nanoassemblies: Toward Layered Polymeric Multicomposites”. In: *Science* 277.5330 (1997), 1232–1237. ISSN: 1095-9203. DOI: 10.1126/science.277.5330.1232. URL: http://dx.doi.org/10.1126/science.277.5330.1232.

[127] D. K. Schwartz et al. “Influence of cations, alkane chain length, and substrate on molecular order of Langmuir-Blodgett films”. In: *Journal of the American Chemical Society* 115.16 (1993), 7374–7380. ISSN: 1520-5126. DOI: 10.1021/ja00069a040. URL: http://dx.doi.org/10.1021/ja00069a040.

[128] Gennady B. Khomutov et al. “Langmuir monolayers and Langmuir-Blodgett films of pH-sensitive lipid”. In: *Colloids and Surfaces A: Physicochemical and Engineering Aspects* 532 (2017), 150–154. ISSN:

0927-7757. DOI: 10.1016/j.colsurfa.2017.05.070. URL: http://dx.doi.org/10.1016/j.colsurfa.2017.05.070.

[129] JAYASREE NATH et al. "MONOLAYER CHARACTERISTICS OF CHITOSAN ASSEMBLED IN LANGMUIR FILMS MIXED WITH ARACHIDIC ACID". In: *Surface Review and Letters* 21.04 (2014), p. 1450049. ISSN: 1793-6667. DOI: 10.1142/s0218625x14500498. URL: http://dx.doi.org/10.1142/s0218625x14500498.

[130] P. Tippmann-Krayer, R.M. Kenn, and H. Möhwald. "Thickness and temperature dependent structure of Cd arachidate Langmuir-Blodgett films". In: *Thin Solid Films* 210–211 (1992), 577–582. ISSN: 0040-6090. DOI: 10.1016/0040-6090(92)90346-d. URL: http://dx.doi.org/10.1016/0040-6090(92)90346-d.

[131] Markus Retsch et al. "Fabrication of Large-Area, Transferable Colloidal Monolayers Utilizing Self-Assembly at the Air/Water Interface". In: *Macromolecular Chemistry and Physics* 210.3–4 (2009), 230–241. ISSN: 1521-3935. DOI: 10.1002/macp.200800484. URL: http://dx.doi.org/10.1002/macp.200800484.

[132] Luzhu Xu et al. "Continuous Langmuir–Blodgett Deposition and Transfer by Controlled Edge-to-Edge Assembly of Floating 2D Materials". In: *Langmuir* 35.1 (2018), 51–59. ISSN: 1520-5827. DOI: 10.1021/acs.langmuir.8b03173. URL: http://dx.doi.org/10.1021/acs.langmuir.8b03173.

[133] Keerti Choudhary, Manjuladevi V., and R. K. Gupta. "Studies on morphology of Langmuir-Blodgett films of stearic acid deposited with different orientation of substrates with respect to compression". In: *AIP Conference Proceedings*. Vol. 1728. Author(s), 2016, p. 020159. DOI: 10.1063/1.4946210. URL: http://dx.doi.org/10.1063/1.4946210.

[134] Keerti Choudhary et al. "Langmuir–Blodgett films of stearic acid deposited on substrates at different orientations relative to compression direction: alignment layer for nematic liquid crystal". In: *Liquid Crystals* 44.10 (2017), 1592–1599. ISSN: 1366-5855. DOI: 10.1080/02678292.2017.1306890. URL: http://dx.doi.org/10.1080/02678292.2017.1306890.

[135] M.R. Buhaenko et al. "Structure and temperature dependence of fatty acid Langmuir-Blodgett films studied by neutron and X-ray scattering". In: *Thin Solid Films* 159.1–2 (1988), 253–265. ISSN: 0040-6090. DOI: 10.1016/0040-6090(88)90637-2. URL: http://dx.doi.org/10.1016/0040-6090(88)90637-2.

[136] Alvina V. Alexandrova et al. "Structure affinity of the Langmuir monolayer and the corresponding Langmuir–Blodgett film revealed by X-ray techniques". In: *Soft Matter* 20.43 (2024), 8601–8609. ISSN: 1744-6848. DOI: 10.1039/d4sm01050g. URL: http://dx.doi.org/10.1039/d4sm01050g.

[137] Simona Bettarini et al. "Characterization of the monolayer as the basis of Langmuir-Blodgett film formation: metal derivatives of behenic acid". In: *Langmuir* 7.6 (1991), 1082–1087. ISSN: 1520-5827. DOI: 10.1021/la00054a010. URL: http://dx.doi.org/10.1021/la00054a010.

[138] Daniel K. Schwartz and Charles M. Knobler. "Direct observations of transitions between condensed Langmuir monolayer phases by polarized fluorescence microscopy". In: *The Journal of Physical Chemistry* 97.35 (1993), 8849–8851. ISSN: 1541-5740. DOI: 10.1021/j100137a005. URL: http://dx.doi.org/10.1021/j100137a005.

[139] Patrycja Dynarowicz-Łatka, Anantharaman Dhanabalan, and Osvaldo N. Oliveira. "Modern physicochemical research on Langmuir monolayers". In: *Advances in Colloid and Interface Science* 91.2 (2001), 221–293. ISSN: 0001-8686. DOI: 10.1016/s0001-8686(99)00034-2. URL: http://dx.doi.org/10.1016/s0001-8686(99)00034-2.

[140] Siji Sudheesh and Jamil Ahmad. "Effect of Wilhelmy Plate Material on Hysteresis of Langmuir Film Isotherms". In: *Asian Journal of Chemistry* 25.7 (2013), 3535–3538. ISSN: 0975-427X. DOI: 10.14233/ajchem.2013.13213. URL: http://dx.doi.org/10.14233/ajchem.2013.13213.

[141] Thiago E. Goto et al. "Monolayer Collapse Regulating Process of Adsorptionâˆ'Desorption of Palladium Nanoparticles at Fatty Acid Monolayers at the Airâˆ'Water Interface". In: *Langmuir* 27.6 (2011), 2667â€"2675. ISSN: 1520-5827. DOI: 10.1021/la104822r. URL: http://dx.doi.org/10.1021/la104822r.

[142] Siji Sudheesh, Jamil Ahmad, and Girija S. Singh. "Hysteresis of Isotherms of Mixed Monolayers of N-Octadecyl-N-phenylthiourea and Stearic Acid at Air/Water Interface". In: *ISRN Physical Chemistry* 2012 (2012), 1–6. ISSN: 2090-7761. DOI: 10.5402/2012/835397. URL: http://dx.doi.org/10.5402/2012/835397.

[143] Lizhao Liu et al. "Mechanical properties of graphene oxides". In: *Nanoscale* 4.19 (2012), p. 5910. ISSN: 2040-3372. DOI: 10.1039/c2nr31164j. URL: http://dx.doi.org/10.1039/c2nr31164j.

[144] Mahdi Tavakol et al. "Mechanical properties of graphene oxide: The impact of functional groups". In: *Applied Surface Science* 525 (2020), p. 146554. ISSN: 0169-4332. DOI: 10.1016/j.apsusc.2020.146554. URL: http://dx.doi.org/10.1016/j.apsusc.2020.146554.

[145] Katharine L. Harrison, Laura B. Biedermann, and Kevin R. Zavadil. "Mechanical Properties of Water-Assembled Graphene Oxide Langmuir Monolayers: Guiding Controlled Transfer". In: *Langmuir* 31.36 (2015), 9825–9832. ISSN: 1520-5827. DOI: 10.1021/acs.langmuir.5b01994. URL: http://dx.doi.org/10.1021/acs.langmuir.5b01994.

[146] Gary Marshall, Michael Dennin, and Charles M. Knobler. "A compact Brewster-angle microscope for use in Langmuir–Blodgett deposition". In: *Review of Scientific Instruments* 69.10 (1998), 3699–3700. ISSN: 1089-7623. DOI: 10.1063/1.1149162. URL: http://dx.doi.org/10.1063/1.1149162.

[147] Shigeru Negi et al. "Highly Ordered Monolayers of an Optically Active Amphiphilic Pyrene Derivative at the Air–Water Interface". In: *Bulletin of the Chemical Society of Japan* 95.11 (2022), 1537–1545. ISSN: 1348-0634. DOI: 10.1246/bcsj.20220233. URL: http://dx.doi.org/10.1246/bcsj.20220233.

[148] Moriyasu KAWAMURA. "Measurement of film thickness by ellipsometry." In: *Journal of the Surface Finishing Society of Japan* 40.2 (1989), 262–267. ISSN: 1884-3409. DOI: 10.4139/sfj.40.262. URL: http://dx.doi.org/10.4139/sfj.40.262.

[149] H. Knobloch, F. Peñacorada, and L. Brehmer. "Ellipsometric studies on uranyl arachidate Langmuir-Blodgett films". In: *Thin Solid Films* 295.1–2 (1997), 210–213. ISSN: 0040-6090. DOI: 10.1016/s0040-

6090(96)09401-1. URL: http://dx.doi.org/10.1016/s0040-6090(96)09401-1.

[150] Changxin Zhu et al. “Morphology of monolayer LB film by STM”. In: *Vacuum* 43.11 (1992), 1111–1113. ISSN: 0042-207X. DOI: 10.1016/0042-207x(92)90345-w. URL: http://dx.doi.org/10.1016/0042-207x(92)90345-w.

[151] Dong June Ahn and Elias I. Franses. “Compositions of Langmuir Monolayers and Langmuir—Blodgett Films with Mixed Counterions”. In: *Mixed Surfactant Systems*. American Chemical Society, 1992, 342–353. ISBN: 9780841213579. DOI: 10.1021/bk-1992-0501.ch023. URL: http://dx.doi.org/10.1021/bk-1992-0501.ch023.

[152] Nidal Hilal and Daniel Johnson. “Atomic Force Microscopy (AFM)”. In: *Encyclopedia of Membranes*. Springer Berlin Heidelberg, 2014, 1–3. ISBN: 9783642408724. DOI: 10.1007/978-3-642-40872-4_34-1. URL: http://dx.doi.org/10.1007/978-3-642-40872-4_34-1.

[153] D Vu and K Birdi. “Application of Scanning Probe Microscopy (Scanning Tunneling Microscopy and Atomic Force Microscopy) in Colloid and Surface Chemistry”. In: *Handbook of Surface and Colloid Chemistry, Second Edition*. CRC Press, 2002. ISBN: 9781420040944. DOI: 10.1201/9781420040944.ch15. URL: http://dx.doi.org/10.1201/9781420040944.ch15.

[154] J. Garnaes et al. “Nano scale defects in langmuir-blodgett film observed by atomic force microscopy”. In: *Synthetic Metals* 57.1 (1993), 3795–3800. ISSN: 0379-6779. DOI: 10.1016/0379-6779(93)90515-x. URL: http://dx.doi.org/10.1016/0379-6779(93)90515-x.

[155] Carlos Marcuello et al. “Langmuir–Blodgett Procedure to Precisely Control the Coverage of Functionalized AFM Cantilevers for SMFS Measurements: Application with Cellulose Nanocrystals”. In: *Langmuir* 34.32 (2018), 9376–9386. ISSN: 1520-5827. DOI: 10.1021/acs.langmuir.8b01892. URL: http://dx.doi.org/10.1021/acs.langmuir.8b01892.

[156] Fumiko Yano and Setsuo Nomura. “Deconvolution of scanning electron microscopy images”. In: *Scanning* 15.1 (1993), 19–24. ISSN: 1932-8745. DOI: 10.1002/sca.4950150103. URL: http://dx.doi.org/10.1002/sca.4950150103.

[157] P Bajaj et al. “Transmission Electron Microscopy and Three-Dimensional Tomography of Peptide-Coated Single-Walled Carbon Nanotubes”. In: *Microscopy and Microanalysis* 17.S2 (2011), 1008–1009. ISSN: 1435-8115. DOI: 10.1017/s1431927611005915. URL: http://dx.doi.org/10.1017/s1431927611005915.

[158] Naureen Akhtar et al. “Structure and Electrical Conductivity of Hybrid Langmuir–Blodgett Films from BEDO-TTF and Fatty Acid”. In: *The Journal of Physical Chemistry C* 116.45 (2012), 24130–24135. ISSN: 1932-7455. DOI: 10.1021/jp307702k. URL: http://dx.doi.org/10.1021/jp307702k.

[159] Leonid M. Goldenberg et al. “Preparation and characterisation of conductive Langmuir–Blodgett films of a tetrabutylammonium–Ni(dmit)2complex”. In: *J. Mater. Chem.* 6.5 (1996), 699–704. ISSN: 1364-5501. DOI: 10.1039/jm9960600699. URL: http://dx.doi.org/10.1039/jm9960600699.

[160] Jia Lin Zhang et al. “Towards single molecule switches”. In: *Chemical Society Reviews* 44.10 (2015), 2998–3022. ISSN: 1460-4744. DOI: 10.1039/c4cs00377b. URL: http://dx.doi.org/10.1039/c4cs00377b.

[161] Andrea C. Ferrari and Denis M. Basko. “Raman spectroscopy as a versatile tool for studying the properties of graphene”. In: *Nature Nanotechnology* 8.4 (2013), 235–246. ISSN: 1748-3395. DOI: 10.1038/nnano.2013.46. URL: http://dx.doi.org/10.1038/nnano.2013.46.

[162] Gerhard Herzberg and Bryce L. Crawford. “Infrared and Raman Spectra of Polyatomic Molecules.” In: *The Journal of Physical Chemistry* 50.3 (1946), 288–288. ISSN: 1541-5740. DOI: 10.1021/j150447a021. URL: http://dx.doi.org/10.1021/j150447a021.

[163] Nobutaka Shioya et al. “Multiple-angle incidence resolution spectrometry: applications in nanoarchitectonics and applied physics”. In: *Japanese Journal of Applied Physics* 63.6 (2024), p. 060102. ISSN: 1347-4065. DOI: 10.35848/1347-4065/ad4ad8. URL: http://dx.doi.org/10.35848/1347-4065/ad4ad8.

[164] C. Naselli, J. F. Rabolt, and J. D. Swalen. “Order–disorder transitions in Langmuir–Blodgett monolayers. I. Studies of two-dimensional melting by infrared spectroscopy”. In: *The Journal of Chemical Physics* 82.4 (1985), 2136–2140. ISSN: 1089-7690. DOI: 10.1063/1.448351. URL: http://dx.doi.org/10.1063/1.448351.

[165] José F. Marco, Claudio Gutiérrez, and María Soledad Ureta-Zañartu. "Study by XPS and UVVisible and DRIFT Spectroscopies of Electropolymerized Films of Substituted Ni(II)-p-Phenylporphyrins and -Phthalocyanines". In: *The Journal of Physical Chemistry B* 112.40 (2008), 12644–12649. ISSN: 1520-5207. DOI: 10.1021/jp8046455. URL: http://dx.doi.org/10.1021/jp8046455.

[166] Avijit Ghosh et al. "Soft chromophore featured liquid porphyrins and their utilization toward liquid electret applications". In: *Nature Communications* 10.1 (2019). ISSN: 2041-1723. DOI: 10.1038/s41467-019-12249-8. URL: http://dx.doi.org/10.1038/s41467-019-12249-8.

[167] John A. Mikroyannidis, Mihalis Fakis, and Ioakim K. Spiliopoulos. "Photophysical and electrochemical characterization of new poly(arylene vinylene) copolymers containing quinoline or bisquinoline segments". In: *Journal of Polymer Science Part A: Polymer Chemistry* 47.13 (2009), 3370–3379. ISSN: 1099-0518. DOI: 10.1002/pola.23412. URL: http://dx.doi.org/10.1002/pola.23412.

[168] Thiago E. Goto et al. "Langmuir–Blodgett films based on poly(p-phenylene vinylene) and protein-stabilised palladium nanoparticles: Implications in luminescent and conducting properties". In: *Thin Solid Films* 540 (2013), 202–207. ISSN: 0040-6090. DOI: 10.1016/j.tsf.2013.05.106. URL: http://dx.doi.org/10.1016/j.tsf.2013.05.106.

[169] Suqian Ma et al. "Organic molecular aggregates: From aggregation structure to emission property". In: *Aggregate* 2.4 (2021). ISSN: 2692-4560. DOI: 10.1002/agt2.96. URL: http://dx.doi.org/10.1002/agt2.96.

[170] Xing Feng et al. "Aggregation behaviour of pyrene-based luminescent materials, from molecular design and optical properties to application". In: *Chemical Society Reviews* 52.19 (2023), 6715–6753. ISSN: 1460-4744. DOI: 10.1039/d3cs00251a. URL: http://dx.doi.org/10.1039/d3cs00251a.

[171] Hikmet Sezen and Sefik Suzer. "XPS for chemical- and charge-sensitive analyses". In: *Thin Solid Films* 534 (2013), 1–11. ISSN: 0040-6090. DOI: 10.1016/j.tsf.2013.02.002. URL: http://dx.doi.org/10.1016/j.tsf.2013.02.002.

[172] Kristian Myhre et al. “Samarium electrodeposited acetate and oxide thin films on stainless steel substrate characterized by XPS”. In: *Surface Science Spectra* 23.1 (2016), 70–81. ISSN: 1520-8575. DOI: 10.1116/1.4954390. URL: http://dx.doi.org/10.1116/1.4954390.

[173] John P. Baltrus and Murphy J. Keller. “Rare earth oxides Eu2O3 and Nd2O3 analyzed by XPS”. In: *Surface Science Spectra* 26.1 (2019). ISSN: 1520-8575. DOI: 10.1116/1.5085768. URL: http://dx.doi.org/10.1116/1.5085768.

[174] A. Gibaud, M. S. Chebil, and T. Beuvier. “X-Ray Reflectivity”. In: *Surface Science Techniques*. Springer Berlin Heidelberg, 2013, 191–216. ISBN: 9783642342431. DOI: 10.1007/978-3-642-34243-1_7. URL: http://dx.doi.org/10.1007/978-3-642-34243-1_7.

[175] Larissa Braun et al. “Polymers and surfactants at fluid interfaces studied with specular neutron reflectometry”. In: *Advances in Colloid and Interface Science* 247 (2017), 130–148. ISSN: 0001-8686. DOI: 10.1016/j.cis.2017.07.005. URL: http://dx.doi.org/10.1016/j.cis.2017.07.005.

[176] O. Arnold et al. “Mantid—Data analysis and visualization package for neutron scattering and ¡mml:math xmlns:mml=”http://www.w3.org/1998/Math/MathML” altimg=”si0002.gif” overflow=”scroll”¿¡mml:mi mathvariant=”normal”¿¡/mml:mi¿¡/mml:m SR experiments”. In: *Nuclear Instruments and Methods in Physics Research Section A: Accelerators, Spectrometers, Detectors and Associated Equipment* 764 (2014), 156–166. ISSN: 0168-9002. DOI: 10.1016/j.nima.2014.07.029. URL: http://dx.doi.org/10.1016/j.nima.2014.07.029.

[177] Zhiyong Wang et al. “Interfacial Synthesis of Layer-Oriented 2D Conjugated Metal–Organic Framework Films toward Directional Charge Transport”. In: *Journal of the American Chemical Society* 143.34 (2021), 13624–13632. ISSN: 1520-5126. DOI: 10.1021/jacs.1c05051. URL: http://dx.doi.org/10.1021/jacs.1c05051.

[178] X. Zhuang et al. “Mapping molecular orientation and conformation at interfaces by surface nonlinear optics”. In: *Physical Review B* 59.19 (1999), 12632–12640. ISSN: 1095-3795. DOI: 10.1103/physrevb.59.12632. URL: http://dx.doi.org/10.1103/physrevb.59.12632.

[179] Katsuhiko Ariga. “Don’t Forget Langmuir–Blodgett Films 2020: Interfacial Nanoarchitectonics with Molecules, Materials, and Living Objects”. In: *Langmuir* 36.26 (2020), 7158–7180. ISSN: 1520-5827. DOI: 10.1021/acs.langmuir.0c01044. URL: http://dx.doi.org/10.1021/acs.langmuir.0c01044.

[180] Ilya Gorbachev et al. “Langmuir-Blodgett Films of Arachidic and Stearic Acids as Sensitive Coatings for Chloroform HF SAW Sensors”. In: *Sensors* 23.1 (2022), p. 100. ISSN: 1424-8220. DOI: 10.3390/s23010100. URL: http://dx.doi.org/10.3390/s23010100.

[181] Hamdan Et al. “Enhancement of Hydrothermally Co3O4 Thin Films as H2S Gas Sensor by Loading Yttrium Element”. In: *Baghdad Science Journal* 16.1(Suppl.) (2019), p. 0221. ISSN: 2078-8665. DOI: 10.21123/bsj.2019.16.1(suppl.).0221. URL: http://dx.doi.org/10.21123/bsj.2019.16.1(suppl.).0221.

[182] Na Li et al. “Gas-Responsive and Self-Powered Visual Composite Langmuir–Blodgett Films for Ultrathin Gas Sensors”. In: *Langmuir* 38.21 (2022), 6761–6770. ISSN: 1520-5827. DOI: 10.1021/acs.langmuir.2c00835. URL: http://dx.doi.org/10.1021/acs.langmuir.2c00835.

[183] Na Li et al. “Chemical gas sensor, surface enhanced Raman scattering and photoelectrics of composite Langmuir-Blodgett films consisting of polypeptide and dye molecules”. In: *Colloids and Surfaces A: Physicochemical and Engineering Aspects* 663 (2023), p. 131067. ISSN: 0927-7757. DOI: 10.1016/j.colsurfa.2023.131067. URL: http://dx.doi.org/10.1016/j.colsurfa.2023.131067.

[184] Inci Capan et al. “Sensing behaviors of lipophilic calix[4]arene phosphonate based Langmuir-Blodgett thin films for detection of volatile organic vapors”. In: *Sensors and Actuators A: Physical* 347 (2022), p. 113947. ISSN: 0924-4247. DOI: 10.1016/j.sna.2022.113947. URL: http://dx.doi.org/10.1016/j.sna.2022.113947.

[185] Katsuhiko Ariga, Taizo Mori, and Junbai Li. “Langmuir Nanoarchitectonics from Basic to Frontier”. In: *Langmuir* 35.10 (2018), 3585–3599. ISSN: 1520-5827. DOI: 10.1021/acs.langmuir.8b01434. URL: http://dx.doi.org/10.1021/acs.langmuir.8b01434.

[186] Katsuhiko Ariga. “Langmuir–Blodgett Nanoarchitectonics, Out of the Box”. In: *Accounts of Materials Research* 3.4 (2021), 404–410. ISSN: 2643-6728. DOI: 10.1021/accountsmr.1c00240. URL: http://dx.doi.org/10.1021/accountsmr.1c00240.

[187] Tianshui Zheng et al. “Stability of Langmuir-Blodgett film/alumina, and Langmuir-Blodgett film/gold multilayer structures”. In: *Thin Solid Films* 197.1–2 (1991), 327–333. ISSN: 0040-6090. DOI: 10.1016/0040-6090(91)90243-q. URL: http://dx.doi.org/10.1016/0040-6090(91)90243-q.

[188] Koichi Kobayashi, Kyo Takaoka, and Shukichi Ochiai. “Comparison of thermal stability of Langmuir-Blodgett films of icosanoic acid and cadmium icosanoate”. In: *Thin Solid Films* 178.1–2 (1989), 453–458. ISSN: 0040-6090. DOI: 10.1016/0040-6090(89)90337-4. URL: http://dx.doi.org/10.1016/0040-6090(89)90337-4.

[189] Mitsuru Akashi and Takami Akagi. “Composite Materials by Building Block Chemistry Using Weak Interaction”. In: *Bulletin of the Chemical Society of Japan* 94.7 (2021), 1903–1921. ISSN: 1348-0634. DOI: 10.1246/bcsj.20210089. URL: http://dx.doi.org/10.1246/bcsj.20210089.

[190] KATSUMI YONEDA. “The Latest LB Film Deposition Apparatus”. In: *Sen'i Gakkaishi* 46.8 (1990), P340–P347. ISSN: 1884-2259. DOI: 10.2115/fiber.46.8_p340. URL: http://dx.doi.org/10.2115/fiber.46.8_p340.

[191] Cathy E. McNamee et al. “Use of nanoparticle concentration and magnetic fields to control the structures of superparamagnetic Fe3O4 nanoparticle Langmuir films”. In: *Colloid and Interface Science Communications* 64 (2025), p. 100817. ISSN: 2215-0382. DOI: 10.1016/j.colcom.2025.100817. URL: http://dx.doi.org/10.1016/j.colcom.2025.100817.

[192] T.S. Berzina et al. “Langmuir-Blodgett films composed of monolayers of amphiphilic molecules and adsorbed soluble proteins”. In: *Thin Solid Films* 284–285 (1996), 757–761. ISSN: 0040-6090. DOI: 10.1016/s0040-6090(95)08439-8. URL: http://dx.doi.org/10.1016/s0040-6090(95)08439-8.

[193] José R. Siqueira et al. "Immobilization of biomolecules on nanostructured films for biosensing". In: *Biosensors and Bioelectronics* 25.6 (2010), 1254–1263. ISSN: 0956-5663. DOI: 10.1016/j.bios.2009.09.043. URL: http://dx.doi.org/10.1016/j.bios.2009.09.043.

[194] Oleg V. Konovalov et al. "XANES Measurements for Studies of Adsorbed Protein Layers at Liquid Interfaces". In: *Materials* 13.20 (2020), p. 4635. ISSN: 1996-1944. DOI: 10.3390/ma13204635. URL: http://dx.doi.org/10.3390/ma13204635.

[195] Katsuhiko Ariga, Yuri Lvov, and Gero Decher. "There is still plenty of room for layer-by-layer assembly for constructing nanoarchitectonics-based materials and devices". In: *Physical Chemistry Chemical Physics* 24.7 (2022), 4097–4115. ISSN: 1463-9084. DOI: 10.1039/d1cp04669a. URL: http://dx.doi.org/10.1039/d1cp04669a.

[196] Gang Zhang and Dayang Wang. "Colloidal Lithography—The Art of Nanochemical Patterning". In: *Chemistry – An Asian Journal* 4.2 (2009), 236–245. ISSN: 1861-471X. DOI: 10.1002/asia.200800298. URL: http://dx.doi.org/10.1002/asia.200800298.

[197] Chenlong Zhang, Sandra Cvetanovic, and Joshua M. Pearce. "Fabricating ordered 2-D nano-structured arrays using nanosphere lithography". In: *MethodsX* 4 (2017), 229–242. ISSN: 2215-0161. DOI: 10.1016/j.mex.2017.07.001. URL: http://dx.doi.org/10.1016/j.mex.2017.07.001.

[198] John C. Hulteen and Richard P. Van Duyne. "Nanosphere lithography: A materials general fabrication process for periodic particle array surfaces". In: *Journal of Vacuum Science amp; Technology A: Vacuum, Surfaces, and Films* 13.3 (1995), 1553–1558. ISSN: 1520-8559. DOI: 10.1116/1.579726. URL: http://dx.doi.org/10.1116/1.579726.

[199] Abhishek Chandramohan et al. "Model for large-area monolayer coverage of polystyrene nanospheres by spin coating". In: *Scientific Reports* 7.1 (2017). ISSN: 2045-2322. DOI: 10.1038/srep40888. URL: http://dx.doi.org/10.1038/srep40888.

[200] Ludovico Valli. "Phthalocyanine-based Langmuir–Blodgett films as chemical sensors". In: *Advances in Colloid and Interface Science* 116.1–3 (2005), 13–44. ISSN: 0001-8686. DOI: 10.1016/j.cis.2005.04.008. URL: http://dx.doi.org/10.1016/j.cis.2005.04.008.

[201] Dan Xie et al. "Gas sensitive Langmuir–Blodgett films based on erbium bis[octakis(octyloxy)phthalocyaninato] complex". In: *Sensors and Actuators B: Chemical* 77.1–2 (2001), 260–263. ISSN: 0925-4005. DOI: 10.1016/s0925-4005(01)00743-2. URL: http://dx.doi.org/10.1016/s0925-4005(01)00743-2.

[202] Epameinondas Leontidis. *Langmuir–Blodgett Films: Sensor and Biomedical Applications and Comparisons with the Layer-by-Layer Method.* 2016. DOI: 10.1002/9783527698813.ch5. URL: http://dx.doi.org/10.1002/9783527698813.ch5.

[203] Andrey V. Arakcheev et al. "X-Ray induced redox-isomeric transformations of lanthanide bis-phthalocyaninates at the air-water interface". In: *Surfaces and Interfaces* 56 (2025), p. 105682. ISSN: 2468-0230. DOI: 10.1016/j.surfin.2024.105682. URL: http://dx.doi.org/10.1016/j.surfin.2024.105682.

[204] Shujuan Huang et al. "Experimental conditions for a highly ordered monolayer of gold nanoparticles fabricated by the Langmuir–Blodgett method". In: *Journal of Vacuum Science amp; Technology B: Microelectronics and Nanometer Structures Processing, Measurement, and Phenomena* 19.6 (2001), 2045–2049. ISSN: 1520-8567. DOI: 10.1116/1.1410943. URL: http://dx.doi.org/10.1116/1.1410943.

[205] Makoto Komiyama, Taizo Mori, and Katsuhiko Ariga. "Molecular Imprinting: Materials Nanoarchitectonics with Molecular Information". In: *Bulletin of the Chemical Society of Japan* 91.7 (2018), 1075–1111. ISSN: 1348-0634. DOI: 10.1246/bcsj.20180084. URL: http://dx.doi.org/10.1246/bcsj.20180084.

[206] Matthew J. Large et al. "Understanding Solvent Spreading for Langmuir Deposition of Nanomaterial Films: A Hansen Solubility Parameter Approach". In: *Langmuir* 33.51 (2017), 14766–14771. ISSN: 1520-5827. DOI: 10.1021/acs.langmuir.7b03867. URL: http://dx.doi.org/10.1021/acs.langmuir.7b03867.

[207] S.A. Hussain, P.K. Paul, and D. Bhattacharjee. "Role of various LB parameters on the optical characteristics of mixed Langmuir–Blodgett films". In: *Journal of Physics and Chemistry of Solids* 67.12 (2006), 2542–2549. ISSN: 0022-3697. DOI: 10.1016/j.jpcs.2006.07.011. URL: http://dx.doi.org/10.1016/j.jpcs.2006.07.011.

S9 First Paper generated using Strategy 2 (137 input papers)

Langmuir and Langmuir-Blodgett Films: Foundations, Frontiers, and Future Directions in Interfacial Molecular Engineering

April 23, 2025

Contents

1 Introduction 2

2 Fundamentals of Langmuir Monolayers 8
2.1 Formation and Molecular Requirements 8
2.2 Surface Pressure-Area Isotherms 9
2.3 Phases and Phase Transitions 11
2.4 Factors Affecting Monolayer Stability and Organization 13
2.5 Characterization Techniques for Langmuir Monolayers 15

3 Langmuir-Blodgett Film Deposition Techniques 16
3.1 Vertical Dipping Method (Langmuir-Blodgett) 16
3.2 Horizontal Lifting Method (Langmuir-Schaefer) 19
3.3 Factors Influencing LB Film Transfer 19
3.4 Transfer Ratio and its Significance 21
3.5 Techniques for Controlling Film Thickness and Uniformity . . 22

4 Characterization of Langmuir-Blodgett Films 23
4.1 Spectroscopic Techniques 23
4.2 X-ray Scattering and Diffraction Techniques 26
4.3 Microscopic Techniques 28
4.4 Ellipsometry 30
4.5 Other Characterization Methods 30

5 Applications of Langmuir-Blodgett Films 31
5.1 Molecular Electronics 33
5.2 Sensors 35
5.3 Optics 38
5.4 Biomaterials and Biomimicry 40

6 Advanced Topics and Emerging Trends 41
6.1 Novel Materials for Langmuir-Blodgett Films 43
6.2 Advanced Control over Film Architecture 45
6.3 Innovations in Langmuir-Blodgett Deposition Methods 46
6.4 Emerging Technological Frontiers 48
6.5 Synergistic Integration with Other Nanofabrication Techniques 50

7 Challenges and Future Directions 52

8 Conclusion 57

Abstract

This review provides a comprehensive survey of Langmuir (L) and Langmuir-Blodgett (LB) film technology, a cornerstone technique for fabricating highly ordered, ultrathin molecular assemblies with nanoscale precision. Beginning with the fundamental principles governing the behavior of amphiphilic molecules at fluid interfaces (Langmuir films) and their controlled transfer onto solid supports (Langmuir-Blodgett films), we trace the historical development and highlight the defining characteristics of these systems, including precise thickness control, high molecular order, and defined molecular orientation. The paper discusses essential characterization methodologies crucial for probing the structure and properties of these nanostructured films. We critically examine the diverse applications where L/LB technology has enabled significant advancements, spanning chemical and biological sensors, electrochemical interfaces, biomimetic studies of membrane biophysics and drug interactions, and emerging energy storage solutions. While comparing L/LB with alternative methods like self-assembled monolayers (SAMs) and layer-by-layer (LbL) assembly, the unique strengths of Langmuir techniques for studying interfacial phenomena and LB deposition for constructing complex, predefined molecular architectures are emphasized. Finally, we explore the future trajectory of the field, focusing on the integration of novel functional

materials, advancements in instrumentation and characterization, the synergistic potential of computational modeling (MD, DFT) and artificial intelligence (AI/ML) for rational design and optimization, and the enduring role of L/LB films in fundamental nanoscience and specialized, high-value technological applications within the nanoarchitectonics framework.

1 Introduction

Langmuir-Blodgett (LB) film technology represents a sophisticated and historically significant method for fabricating highly ordered molecular assemblies at interfaces and transferring them onto solid supports [1, 2]. The process begins with the formation of a Langmuir film, which is essentially a monomolecular layer of amphiphilic molecules organized at the air-water interface [3, 4]. These molecules, typically possessing a hydrophilic head group and a hydrophobic tail, spontaneously arrange themselves when spread from a volatile solvent onto an aqueous subphase, as depicted in Figure 1(a). Subsequent compression using movable barriers reduces the area available to the molecules, forcing them into increasingly ordered two-dimensional phases, monitored by measuring the surface pressure as a function of the area per molecule (Π-A isotherms). When these precisely organized Langmuir films are systematically transferred, layer by layer, onto a solid substrate withdrawn from or dipped into the subphase (Figure 1(b)), the resulting structures are termed Langmuir-Blodgett films [5, 6]. This technique allows for the construction of ultrathin organic films with thicknesses controlled at the nanometer level, often down to a single molecular layer, exhibiting a high degree of structural order and defined molecular orientation, as shown schematically for multilayer deposition in Figure 1(c) and (d) [7].

The origins of this field can be traced back over a century, building upon initial observations of oil spreading on water, famously documented by Benjamin Franklin. Agnes Pockels, working in her home laboratory in the late 19th century, developed early versions of the trough apparatus and made fundamental observations on the behavior of surface films. Irving Langmuir significantly advanced the field in the early 20th century through systematic studies of monolayer properties, developing the Langmuir trough and providing a theoretical framework for understanding interfacial phenomena, work for which he received the Nobel Prize in Chemistry, as documented in

the historical accounts of the field (e.g., Vu et al.) [6]. His collaborator, Katharine Blodgett, subsequently pioneered the technique for transferring these monolayers onto solid substrates in the 1930s, demonstrating the possibility of building multilayer structures with controlled thickness. Later, researchers like Hans Kuhn expanded the conceptual framework, envisioning the construction of complex supramolecular systems or "organizates" by assembling molecules with specific functions in a predetermined architecture within LB films, paving the way for investigations into energy and electron transfer processes at the molecular level. Despite periods of varying research intensity, interest in LB films has persisted, fueled by their unique capabilities for molecular engineering [1, 8].

The defining characteristics of LB films stem directly from their fabrication method. They are inherently ultrathin, with individual layer thicknesses determined by the length of the constituent molecules, typically ranging from one to several nanometers [9, 10]. The layer-by-layer deposition process allows for precise control over the total film thickness [11, 12] and enables the creation of complex multilayer structures with alternating compositions (heterostructures). Perhaps most importantly, under optimal conditions, LB films exhibit a high degree of molecular order, both within each layer (intralayer order) and in the stacking arrangement between layers (interlayer order) [13, 14]. This results in highly anisotropic films with distinct properties parallel and perpendicular to the substrate surface. The ability to control molecular orientation relative to the substrate is another key feature, often involving amphiphilic molecules with distinct head groups interacting with the substrate or subphase, hydrophobic tails providing cohesive interactions, and potentially a terminal functional group (X) interacting with the ambient phase, as schematically illustrated in Figure 2.

These properties collectively make LB films ideal model systems for studying phenomena in confined dimensions and for designing materials with tailored functionalities based on molecular architecture.

The unique structural control offered by the LB technique has established its importance across a wide spectrum of scientific and technological domains [5, 17, 1]. In molecular and organic electronics, LB films have been explored as ultra-thin gate dielectrics, active layers in field-effect transistors (FETs) [18, 19], components in rectifying diodes, and templates for nanolithography [20]. The precise thickness control and potential for ordered semiconducting or insulating layers were particularly attractive, although practical device integration remains challenging, a point discussed in several reviews assessing

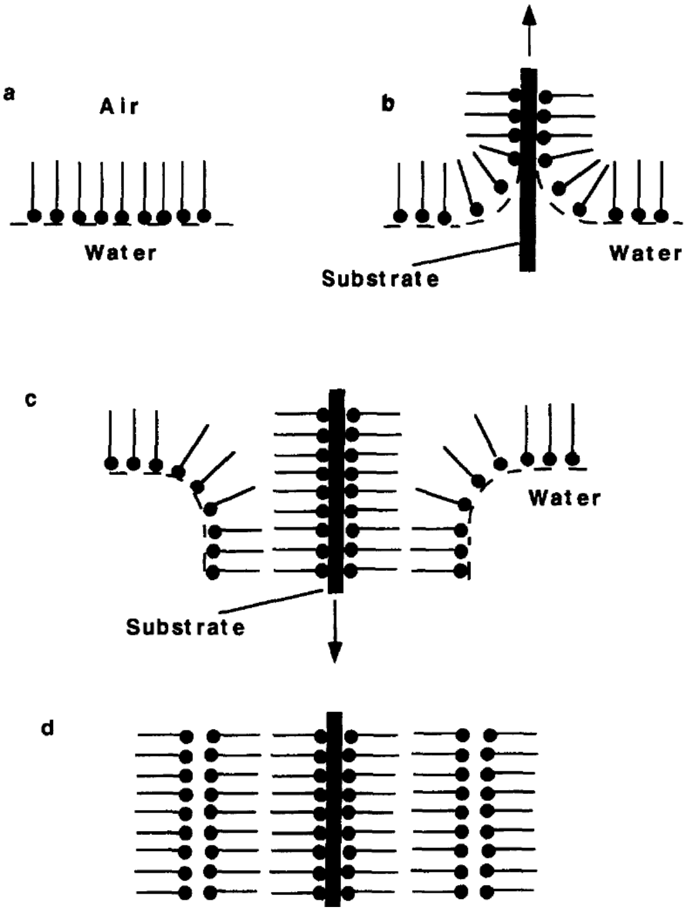

Figure 1: Conventional schematic of the LB technique using a hydrophilic substrate. In the first step (a), a suitable amphiphilic molecule is dissolved in a volatile solvent that is then spread at the air—water interface to form a Langmuir monolayer. With a barrier, the area of the trough can be altered to change the local density of the molecules, and usually the local organization and order as well. To form the LB film (b), a substrate is passed through the interface a given number of times, with each pass adding another monolayer to the LB film with alternating molecular orientations (c) and (d). Adapted from Schwartz, D., 1997 [15].

the applied potential of these films (e.g., Oliveira et al., Schwartz, Vincett). Sensor technology represents another major application area [21, 22]. The high surface-area-to-volume ratio, ordered structure, and ability to incorpo-

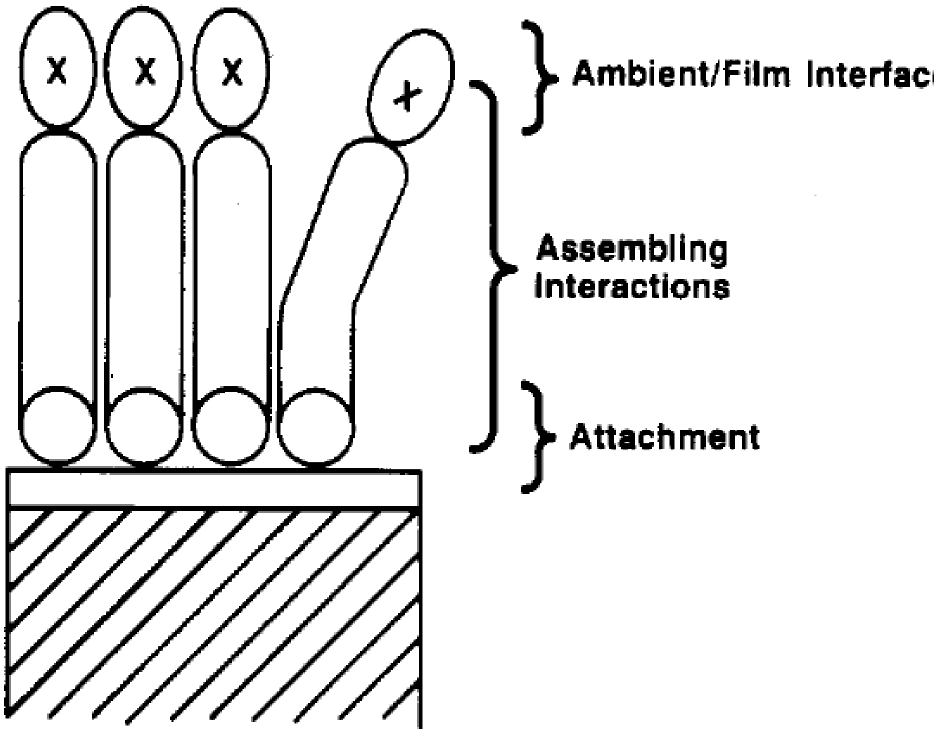

Figure 2: Molecules attached to a surface shown schematically. They consist of a head group attached to a substrate, some interior group (most often an alkyl chain) which provides cohesive in- teractions, and a terminal functional group (X) which interacts with the ambient phase. Adapted from Swalen, J., et al., 1987 [16].

rate specific receptor molecules (e.g., polymers, nanoparticles, biomolecules like enzymes or antibodies) make LB films highly suitable for detecting gases, chemicals in solution, and biological analytes with high sensitivity and selectivity [23, 22]. In biophysics and biomaterials science, LB films serve as valuable models for biological membranes [24, 25]. Lipid monolayers and multilayers mimic the structure of cell membranes, allowing researchers to study membrane fluidity, phase behavior, protein-lipid interactions, and the effects of drugs or toxins under controlled conditions. Furthermore, the technique can be used to immobilize proteins and other biomolecules with controlled orientation for applications in biosensors and biocompatible coatings [26, 27]. In optics and photonics, the precise control over thickness and refractive in-

dex enables the fabrication of optical waveguides, anti-reflection coatings, and structures for studying surface plasmon resonance or non-linear optical effects. The ability to incorporate dyes and other optically active molecules allows for investigations into energy transfer and the creation of specialized optical components. More broadly, in materials science and nanotechnology, the LB technique is a fundamental tool for creating well-defined nanostructures, studying self-assembly processes at interfaces, and exploring the properties of materials in two dimensions, contributing significantly to the concept of "nanoarchitectonics" [28, 29, 30]. The method is versatile, applicable not only to classical amphiphiles like fatty acids but also to polymers [7], nanoparticles, proteins, DNA [31], and even 2D materials like graphene or transition metal dichalcogenides, often requiring modifications to the standard procedures.

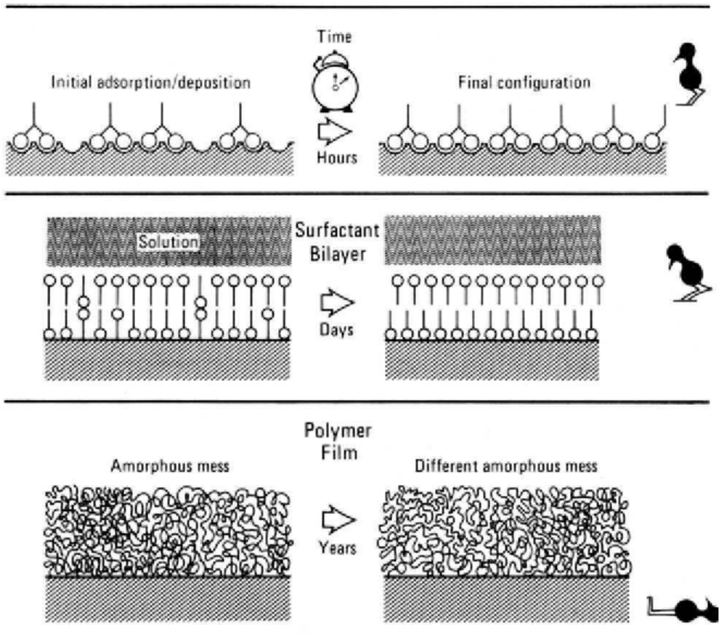

Figure 3: Effects of aging on thin films of surfactants and polymers shown schematically. Adapted from Swalen, J., et al., 1987 [16].

Despite their remarkable capabilities, LB films face several challenges and limitations that have hindered their widespread commercial application. One major issue is the mechanical stability and robustness of the films, particularly those made from simple, small amphiphilic molecules, which can be

prone to defects like pinholes and may degrade over time or under harsh conditions. Film properties can change over time due to aging effects, involving molecular rearrangement, desorption, or chemical degradation, as schematically shown in Figure 3. While using polymers or cross-linkable molecules can improve stability, it may compromise the degree of order. The deposition process itself can be slow and requires careful control of experimental parameters (subphase purity, temperature, vibration isolation, deposition speed, surface pressure), making large-scale, high-throughput fabrication difficult and potentially costly. Reproducibility can also be a concern. Furthermore, alternative thin-film fabrication techniques have emerged, such as self-assembled monolayers (SAMs) and layer-by-layer (LbL) assembly. SAMs offer robust, chemisorbed monolayers on specific substrates, while LbL assembly provides a simpler, more versatile method for building robust multilayer films from a wider range of (often water-soluble) materials, albeit typically with less molecular-level order than ideal LB films; these comparisons regarding robustness and manufacturing ease are frequently highlighted in the literature (e.g., Oliveira et al., Fang et al.). Consequently, while LB technology remains unparalleled for achieving exquisite control over molecular architecture in ultrathin films for research and specialized applications, these competing techniques are often preferred for applications demanding greater robustness or ease of manufacturing.

This survey aims to provide a comprehensive overview of the field of Langmuir and Langmuir-Blodgett films [32, 1, 5]. It will delve into the fundamental principles governing the behavior of molecules at the air-water interface [33], describe the various techniques used for film deposition [2], and discuss the array of methods employed for characterizing the structure and properties of these unique molecular assemblies [34, 35]. Emphasis will be placed on highlighting the diverse applications where LB films have made significant contributions or hold considerable potential, ranging from electronics [36, 37] and sensors [38, 39, 40] to biomimetic systems [5] and advanced materials [41]. The review will also touch upon recent advancements, emerging trends, inherent challenges, and future directions in this enduring area of surface and materials science.

The structure of this survey is organized as follows: Section 2 will cover the fundamental principles of Langmuir monolayers, including molecular interactions at the air-water interface, phase behavior, and thermodynamics. Section 3 will detail the Langmuir-Blodgett and related deposition techniques used to transfer monolayers onto solid substrates [5, 1]. Section 4 will dis-

cuss the various experimental techniques employed to characterize the structure, morphology, and properties of LB films [35, 42]. Section 5 will provide an overview of the key application areas of LB films [43, 44]. Section 6 will explore advanced topics and emerging trends, including the use of non-traditional materials and novel fabrication approaches [4, 45]. Section 7 will address the current challenges and outline future prospects for the field. Finally, Section 8 will summarize the key aspects discussed throughout the survey.

2 Fundamentals of Langmuir Monolayers

The study of Langmuir monolayers represents a cornerstone in surface science, providing a controllable system for investigating the behavior of molecules confined to a two-dimensional interface, typically the air-water interface [33]. These monolayers serve as precursors for Langmuir-Blodgett (LB) films [46, 1] and offer valuable insights into interfacial phenomena relevant to fields ranging from materials science to biophysics [47, 48, 49]. A Langmuir monolayer is fundamentally a single layer of molecules formed spontaneously or by controlled spreading at the boundary between two immiscible phases, most commonly air and water. The formation and stability of such monolayers depend critically on the molecular nature of the constituent species.

2.1 Formation and Molecular Requirements

Substances capable of forming stable Langmuir monolayers must generally satisfy two primary criteria: amphiphilicity and insolubility in the subphase (typically water). Amphiphilicity refers to the dual chemical nature of the molecule, possessing distinct hydrophilic (water-loving) and hydrophobic (water-fearing) parts. The classic examples are long-chain fatty acids, alcohols, or phospholipids, where a polar head group (e.g., $-COOH$, $-OH$, phosphate group) constitutes the hydrophilic moiety, and a long alkyl chain ($-C_nH_{2n+1}$) forms the hydrophobic tail. The hydrophobic part ensures the molecule's insolubility or extremely low solubility in the aqueous subphase, preventing it from dissolving into the bulk. Simultaneously, the hydrophilic head group must exhibit sufficient affinity for the water surface to anchor the molecule effectively at the interface.

The preparation of a Langmuir monolayer typically involves dissolving

the amphiphilic compound in a volatile organic solvent, such as chloroform, hexane, or a mixture thereof. This solution is then carefully deposited, often dropwise, onto the clean surface of the aqueous subphase contained within a specialized apparatus known as a Langmuir trough [50]. The trough is usually constructed from an inert material like polytetrafluoroethylene (PTFE, Teflon) to prevent contamination and ensure chemical inertness. For spreading to occur spontaneously and effectively, the chosen solvent must possess a positive spreading coefficient, S, defined in terms of interfacial tensions (γ) as $S = \gamma_{\mathrm{w/a}} - \gamma_{\mathrm{o/a}} - \gamma_{\mathrm{w/o}} > 0$, where w, a, and o denote water, air, and the organic solvent, respectively. A positive S indicates that the reduction in surface free energy upon spreading the solvent over the water surface is favorable [51]. As the volatile solvent evaporates (usually within minutes), the amphiphilic molecules remain, organizing themselves at the air-water interface [33]. The hydrophilic head groups immerse themselves in or interact strongly with the water surface via electrostatic forces (ion-dipole, dipole-dipole interactions, including hydrogen bonding), while the hydrophobic tails orient themselves away from the water, typically extending into the air phase [52].

The balance of forces dictates the initial state of the monolayer; if the adhesion forces (head group-water) dominate over lateral cohesion forces (tail-tail interactions), the molecules tend to spread out, potentially forming a dilute, gas-like phase across the available surface area. Van der Waals interactions between the hydrophobic tails become increasingly significant as the molecules are brought closer together, favoring molecular ordering and condensation [53, 54].

2.2 Surface Pressure-Area Isotherms

The thermodynamic state of a Langmuir monolayer is most commonly investigated by measuring its surface pressure-area ($\pi - A$) isotherm. This involves systematically varying the surface area available to a fixed number of molecules while monitoring the resulting change in surface pressure at a constant temperature. The Langmuir trough is equipped with one or more movable barriers, typically made of Teflon, which slide across the water surface to compress or expand the monolayer. The total surface area (A_{total}) is controlled, and knowing the number of molecules (N) deposited allows calculation of the area per molecule ($A = A_{\mathrm{total}}/N$), often expressed in units of Å^2/molecule or nm^2/molecule.

The surface pressure (π) is defined as the reduction in the surface ten-

sion of the pure subphase (γ_0) caused by the presence of the monolayer (γ): $\pi = \gamma_0 - \gamma$. It represents the two-dimensional analogue of pressure in a three-dimensional system and is typically measured in mN/m. The most common method for measuring surface tension (and thus surface pressure) is the Wilhelmy plate method [55, 56]. A thin plate, usually made of roughened platinum or filter paper to ensure zero contact angle ($\theta = 0$), is suspended from a sensitive electrobalance and partially immersed through the interface. The balance measures the downward force (F) exerted on the plate, which includes contributions from gravity, buoyancy, and surface tension acting along the wetted perimeter (p) of the plate. The surface tension is calculated as $\gamma = F/(p \cos\theta)$. Assuming $\cos\theta = 1$ for complete wetting, $\gamma = F/p$. By measuring γ_0 before spreading the monolayer and γ during the experiment, π can be determined continuously as the area per molecule A is varied.

A $\pi - A$ isotherm, obtained by plotting π versus A at constant temperature, provides fundamental thermodynamic information about the monolayer, analogous to a $P - V$ isotherm for a bulk substance [57]. The shape of the isotherm, including slopes, kinks, and plateaus, reveals the different two-dimensional phases the monolayer adopts upon compression and the transitions between these phases [58, 59, 60]. Figure 4 shows a typical experimental $\pi - A$ isotherm for pentadecanoic acid, illustrating features such as the initial low-pressure region (gaseous phase), the rise in pressure corresponding to the liquid-expanded phase, a plateau or region of changing slope indicating a phase transition (e.g., LE-LC) [61, 62], and a steep increase in the condensed phase region (LC or S) before potential collapse at very small areas [63, 64].

2.3 Phases and Phase Transitions

As a Langmuir monolayer is compressed from a large area per molecule, it typically passes through a sequence of phases analogous to the gas, liquid, and solid states of bulk matter, although the two-dimensional nature introduces unique characteristics and additional phases. An example of the features corresponding to these phases in an experimental isotherm is shown in Figure 4. At very large areas per molecule (low surface pressure), the monolayer exists in a *Gaseous (G)* phase. Here, molecules are far apart, interact weakly, and behave like a 2D gas, exerting minimal surface pressure. Upon compression, the surface pressure begins to rise more steeply, indicating entry into a *Liquid-Expanded (LE)* phase. In this phase, molecules are

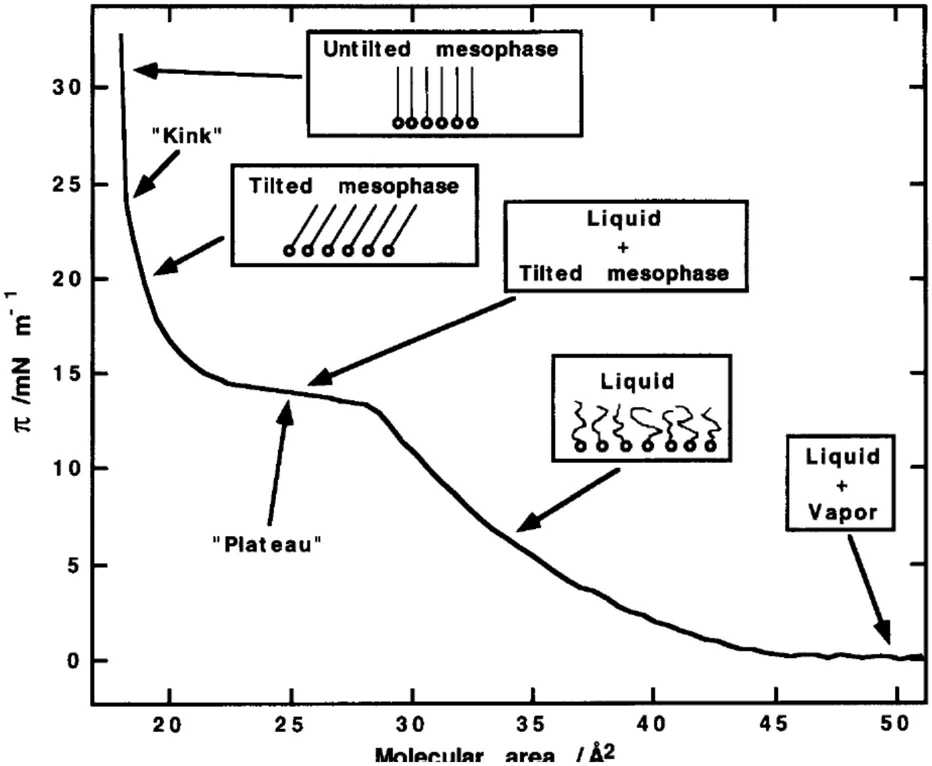

Figure 4: A surface pressure (π) vs. molecular area isotherm of a pentadecanoic acid Langmuir monolayer on pure water at about 30°C. Various features of the isotherm can be identified with different structural phases and the transitions between them. Adapted from Schwartz, D., 1997 [15].

closer together and interact more significantly, but the chains retain considerable conformational disorder and lateral mobility, behaving like a 2D liquid. Further compression often leads to a first-order phase transition to a more ordered *Liquid-Condensed (LC)* phase. This transition is frequently marked by a plateau or a region of significantly reduced slope in the $\pi - A$ isotherm (visible as a change in slope around 25 Å^2/molecule in Figure 4), indicating coexistence of the LE and LC phases. In the LC phase, molecules are packed more densely, often with alkyl tails exhibiting higher orientational order (less tilting or more uniform tilting) but still possessing some fluidity. Continued compression leads to a *Solid (S)* phase, characterized by a very steep slope in the isotherm, indicating low compressibility (seen at areas below approx.

21 Å^2/molecule in Figure 4). In this phase, molecules are closely packed in a 2D crystalline or quasi-crystalline lattice, with alkyl chains typically adopting an all-trans conformation and oriented nearly perpendicular or at a specific tilt angle to the interface. Positional and orientational correlations are long-ranged, although true long-range positional order may be limited in 2D systems, leading to hexatic phases. Finally, if compression continues beyond the stability limit of the solid monolayer, the surface pressure may level off or decrease abruptly. This signifies *Collapse*, where the 2D structure breaks down, and the monolayer buckles, folds, or transforms into multilayer structures or 3D aggregates to relieve the excess stress.

The compressibility of the monolayer in different phases can be quantified by the surface compressional modulus, C_s^{-1}, defined as $C_s^{-1} = -A(\partial\pi/\partial A)_T$. It is the 2D analogue of the bulk modulus and reflects the resistance of the monolayer to compression [65, 60]. Different phases exhibit characteristic ranges of C_s^{-1} values, as summarized in Table 1.

Table 1: Typical two-dimensional phases of Langmuir monolayers and their approximate compressional modulus (C_s^{-1}) ranges.

Phase	Compressional Modulus C_s^{-1} (mN/m)
Gaseous (G)	~ 0
Liquid-Expanded (LE)	$12.5 - 50$
Liquid-Condensed (LC)	$100 - 250$
Solid (S)	> 250

Note: These ranges are approximate and can vary depending on the specific amphiphile, temperature, and subphase conditions. The LE-LC transition region exhibits very low C_s^{-1} values.

Modern characterization techniques, particularly grazing-incidence X-ray diffraction (GIXD) [3] and optical microscopies, have revealed a richer phase behavior than suggested by simple G, LE, LC, S classification, especially for molecules like fatty acids and phospholipids. Several distinct liquid-crystalline or mesophases have been identified [66, 67], often characterized by different degrees of positional order, bond-orientational order, and molecular tilt (magnitude and direction relative to the lattice) [68]. For instance, phases like L_2, L_2', Ov, LS, S, and CS have been described for fatty acid monolayers [69, 70], exhibiting varying degrees of tilt towards nearest or next-nearest neighbors, or being untilted [71]. Detailed phase diagrams, such as

the one presented for n-alkanoic acids in Figure 2 of Schwartz (1997), map the complex interplay of these phases as a function of temperature and surface pressure.

2.4 Factors Affecting Monolayer Stability and Organization

The structure, phase behavior, and stability of Langmuir monolayers are sensitive to several experimental parameters. *Temperature* plays a crucial role, influencing molecular kinetic energy, chain fluidity, and the positions of phase boundaries. Increasing temperature generally shifts phase transitions to higher surface pressures or lower areas per molecule and can induce transitions from more ordered to less ordered phases (e.g., S to LC, LC to LE). The *pH of the aqueous subphase* is particularly important for amphiphiles with ionizable head groups, such as fatty acids ($-COOH$) or amines ($-NH_2$). The pH determines the degree of ionization of the head group (e.g., $-COOH \rightleftharpoons -COO^- + H^+$). Ionization introduces electrostatic repulsion between charged head groups, which tends to expand the monolayer and can significantly alter packing density, phase transitions, and stability. For instance, fatty acid monolayers are generally more expanded and less stable at high pH where the carboxylate form dominates. The *ionic strength and specific ion composition* of the subphase also exert strong influences. Dissolved ions can screen electrostatic repulsions between charged head groups, promoting condensation. Divalent cations (e.g., Ca^{2+}, Mg^{2+}, Cd^{2+}) can form specific complexes or bridges with anionic head groups (like carboxylates or phosphates), leading to much denser packing, increased stability, altered crystal structures, and potentially inducing ordered phases even at low surface pressures. The type and concentration of ions can thus be used to precisely tune monolayer properties. The interplay between pH and the presence of specific metal cations in forming fatty acid salts at the interface, which is critical for the structure and subsequent transferability in LB deposition, is illustrated by the data compiled in Figure 5, showing the percentage of salt formation as a function of subphase pH for arachidic acid in the presence of various divalent metal ions. Indeed, subphase ions significantly affect film formation, structure, stability, and even electrochemical properties through these interactions with the amphiphilic molecules.

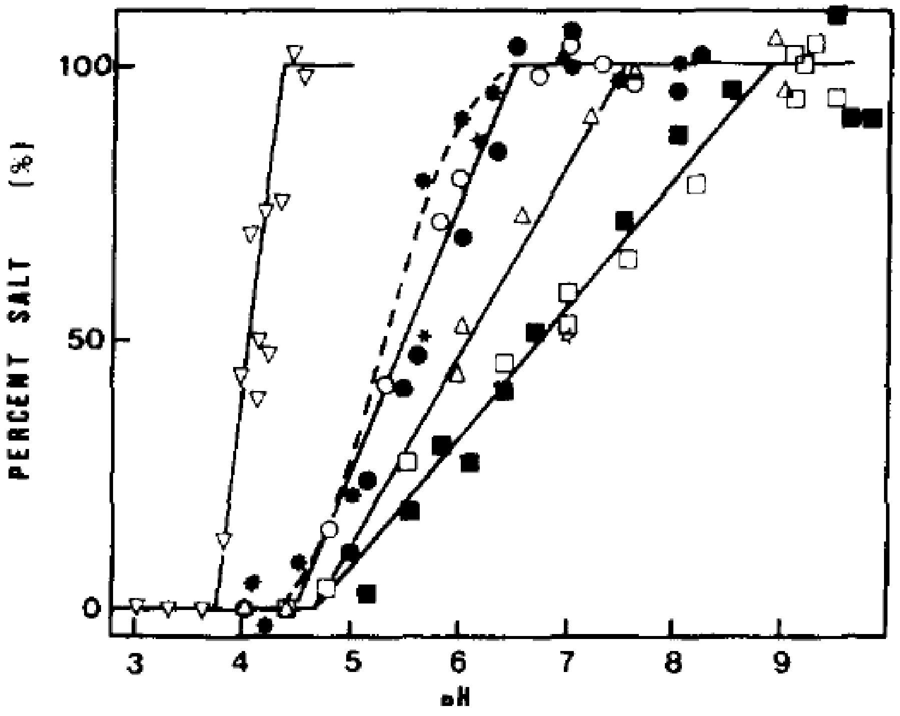

Figure 5: Effect of subphase pH on the percentage of salt formation for arachidic acid films deposited from solutions containing different divalent metal cations, based on various experimental techniques (XPS, FTIR, neutron activation analysis, photometry). Data points are compiled from studies by Kobayashi et al., Petrov et al., and Blodgett, as indicated by symbols. Adapted from Schwartz, D., 1997 [15].

2.5 Characterization Techniques for Langmuir Monolayers

While $\pi-A$ isotherms provide essential thermodynamic information, a deeper understanding of monolayer structure and morphology requires the application of specialized surface-sensitive characterization techniques performed *in situ* at the air-water interface. *Optical Microscopies*, such as Brewster Angle Microscopy (BAM) and Epifluorescence Microscopy (EFM), allow direct visualization of the monolayer morphology on mesoscopic length scales (micrometers to millimeters). BAM utilizes the reflection of p-polarized light near the Brewster angle of the pure subphase; the presence of a monolayer al-

ters the reflectivity, providing contrast based on local thickness and refractive index variations. EFM requires doping the monolayer with a small amount of fluorescent probe molecule; variations in probe partitioning or environment between different phases provide contrast, revealing domain shapes, sizes, and phase coexistence. These techniques are invaluable for studying phase transitions, domain growth, and texture. *X-ray Scattering Techniques*, particularly Grazing Incidence X-ray Diffraction (GIXD) and X-ray Reflectivity (XRR), performed using synchrotron radiation sources, provide detailed structural information at the molecular level. GIXD probes the in-plane molecular packing, yielding information about lattice parameters, symmetry, coherence lengths (domain sizes), and molecular tilt angles and directions. XRR measures the electron density profile perpendicular to the interface, giving precise values for monolayer thickness, roughness, and head group/tail electron densities. *Vibrational Spectroscopies*, such as Infrared Reflection-Absorption Spectroscopy (IRRAS) and Sum Frequency Generation (SFG) spectroscopy, probe the vibrational modes of the monolayer molecules. IRRAS, often performed using polarization modulation (PM-IRRAS) for enhanced surface sensitivity, provides information about the chemical composition, conformation (e.g., trans/gauche content of alkyl chains), and average orientation of specific molecular groups relative to the surface normal. SFG, a nonlinear optical technique, is inherently surface-specific and can provide similar information on orientation and conformation. *Surface Potential (SP) Measurements* determine the change in potential difference across the air-water interface upon formation of the monolayer. This potential arises from the oriented dipoles of the amphiphilic molecules and the reorientation of water molecules at the interface. SP measurements provide insights into the average molecular orientation and the electrical properties of the interface. Collectively, these techniques, often used in conjunction with isotherm measurements, have dramatically advanced our understanding of the complex structures, phases, and interactions within Langmuir monolayers over the past few decades. This detailed understanding of the precursor monolayer is crucial for controlling the structure and properties of the subsequently deposited Langmuir-Blodgett films.

3 Langmuir-Blodgett Film Deposition Techniques

Transferring the stable Langmuir monolayer from the air-water interface onto a solid substrate is the defining step in creating Langmuir-Blodgett (LB) films. This transfer process, illustrated schematically in Figure 6, allows the construction of ultrathin films with precisely controlled thickness and molecular architecture. Several methods exist for this transfer, each suited for different monolayer types and applications. The primary methods are vertical dipping (Langmuir-Blodgett) and horizontal lifting (Langmuir-Schaefer), and their success depends critically on understanding and controlling the factors governing the transfer process.

3.1 Vertical Dipping Method (Langmuir-Blodgett)

The classical Langmuir-Blodgett (LB) technique uses vertical substrate movement through the compressed Langmuir monolayer at the air-water interface (Figure 7). After spreading an amphiphile solution and evaporating the solvent, movable barriers compress the resulting monolayer to a desired surface pressure (π), typically within a condensed phase identified from the surface pressure-area (π-A) isotherm. The substrate is then immersed (downstroke) and withdrawn (upstroke) at a controlled speed while maintaining constant surface pressure. During these passages, the monolayer transfers onto the substrate.

The transfer characteristics depend on monolayer-substrate-subphase interactions. Y-type deposition, the most common mode, involves monolayer transfer during both downstroke and upstroke. For a hydrophilic substrate, the first layer typically adheres during the upstroke (heads to substrate). Subsequent layers add tail-to-tail on the downstroke and head-to-head on the upstroke, forming a centrosymmetric bilayer structure [73]. For a hydrophobic substrate, the first layer usually transfers on the downstroke (tails to substrate), followed by Y-type deposition.

Less common are X-type (downstroke only) and Z-type (upstroke only) depositions. While these could theoretically yield non-centrosymmetric films with uniform molecular orientation [74, 75], experimental evidence often shows that films formed by nominal X- or Z-type deposition rearrange into the more stable Y-type bilayer structure. This rearrangement is driven by the

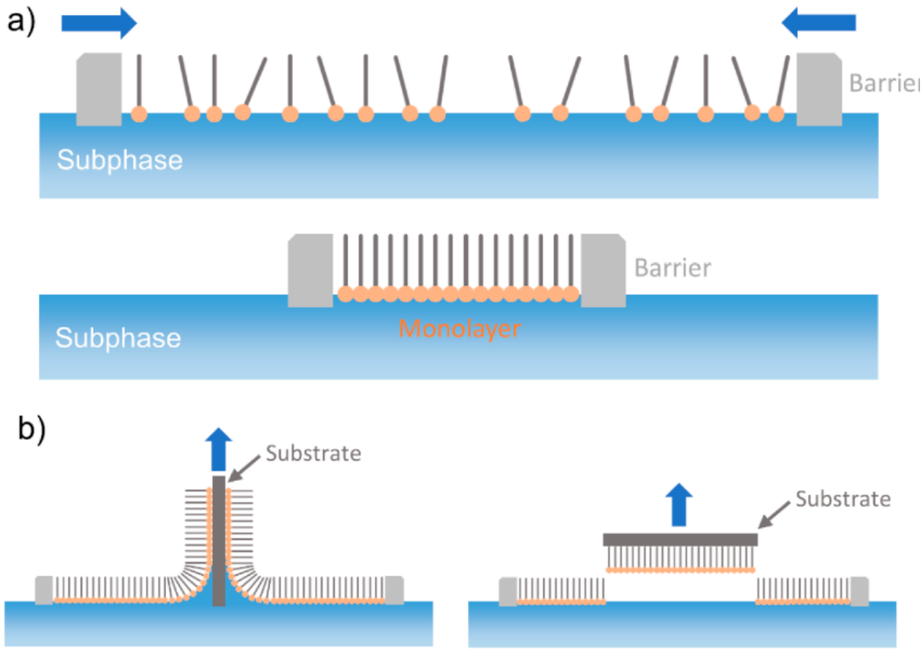

Figure 6: Schematic illustration of basic principles of Langmuir—Blodgett-type methods. (a) Formation of dense monolayer at air—liquid interface (commonly using water as subphase) with amphiphilic molecules (orange, hydrophilic head; gray, hydrophobic tail) upon barrier compression. (b) Transfer of monolayer from liquid surface to substrate through vertical (left) or horizontal deposition (right). (c) Illustration of a typical LB isotherm during compression. Adapted from Fang, C., et al., 2022 [72].

system's tendency to minimize thermodynamically unfavorable hydrophobic/hydrophilic contact mismatches inherent in the idealized X- or Z-type packing, favoring the stable bilayer arrangement found in Y-type films (as discussed by Schwartz, 1997). The stability of true X- or Z-type multilayers remains a significant challenge [13]. The orientation of the outermost layer also depends on the environment: hydrophobic tails typically face air, while polar headgroups face an aqueous phase. Attempts to create air-exposed polar surfaces by halting deposition before the final upstroke often fail due to the removal of the unstable outer layer.

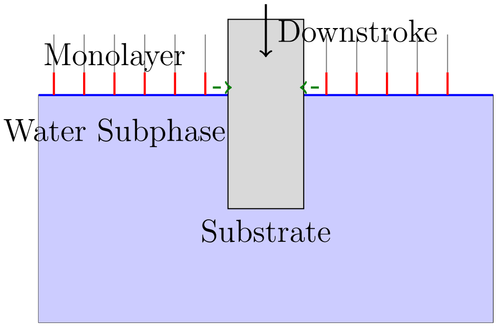

(a) Downstroke

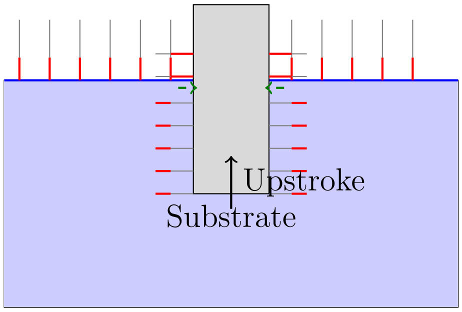

(b) Upstroke (Y-type)

Figure 7: Schematic illustration of the vertical dipping (Langmuir-Blodgett) deposition process. (a) During the downstroke, the substrate moves downwards through the monolayer at the air-water interface. (b) During the upstroke, the substrate moves upwards. In Y-type deposition shown here (assuming initial transfer on downstroke for a hydrophobic substrate), monolayers are transferred during both movements, leading to a bilayer structure.

3.2 Horizontal Lifting Method (Langmuir-Schaefer)

The Langmuir-Schaefer (LS) technique offers an alternative horizontal transfer method (Figure 9). Here, the substrate contacts the Langmuir monolayer surface horizontally, adheres to it, and is then lifted away. LS is particularly advantageous for rigid monolayers prone to fracture during vertical dipping or when specific non-centrosymmetric structures are targeted (though stability remains a factor).

One LS transfer typically deposits a single monolayer. Multilayers can be built by repeating the process, potentially requiring substrate drying be-

tween steps. Furthermore, LB and LS methods are versatile tools for assembling nanomaterials, such as aligning nanowires into ordered arrays or crossed structures on a substrate, as depicted in Figure 8 [76, 77]. The LS method generally provides a gentler transfer for delicate assemblies. A combination of LB and LS techniques has also been utilized, for instance, in the assembly of supported lipid bilayers (SLBs) which serve as models for cell membranes [24, 78, 79]; this combination allows for flexibility in creating potentially asymmetric bilayers or incorporating different lipid types that might be difficult to deposit using only one method (as explored by Oliveira Jr et al., 2022). Achieving high-quality LS films requires careful control of contact angle, lifting speed, and monolayer stability [80, 13].

3.3 Factors Influencing LB Film Transfer

The success and quality of LB or LS film deposition depend sensitively on various experimental parameters. Precise control is essential for achieving uniform, well-ordered films. Key factors include amphiphile properties, subphase composition, substrate characteristics, and deposition parameters.

The amphiphile's molecular structure (hydrophilic-hydrophobic balance) governs its interfacial behavior and substrate interaction. The Langmuir monolayer's phase behavior, revealed by the π-A isotherm (illustrated schematically in Figure 6c), dictates the optimal surface pressure for transfer [63]. Deposition typically occurs in a condensed phase (liquid-condensed or solid) ensuring stability and dense packing without collapse [13, 63]. The chosen transfer pressure significantly affects the packing, orientation, and morphology of the transferred molecules [82, 83].

Subphase composition is critical. pH influences headgroup ionization, affecting intermolecular forces and substrate adhesion [84, 85, 86, 87]. Ions (e.g., divalent cations for fatty acids) can stabilize monolayers via complexation [88, 89, 90] and act as ionic bridges, promoting adhesion between the monolayer and charged substrates or between successive layers (a strategy noted by Oliveira Jr et al., 2022). Temperature impacts molecular mobility, viscosity, and phase transitions, influencing transfer quality [91, 92]. High water purity is essential to avoid monolayer disruption.

Substrate properties like material, cleanliness, roughness [93], and surface energy (hydrophilicity/hydrophobicity) strongly affect adhesion. Meticulous cleaning [94, 95] and sometimes surface treatments (e.g., silanization) are employed to ensure uniform deposition.

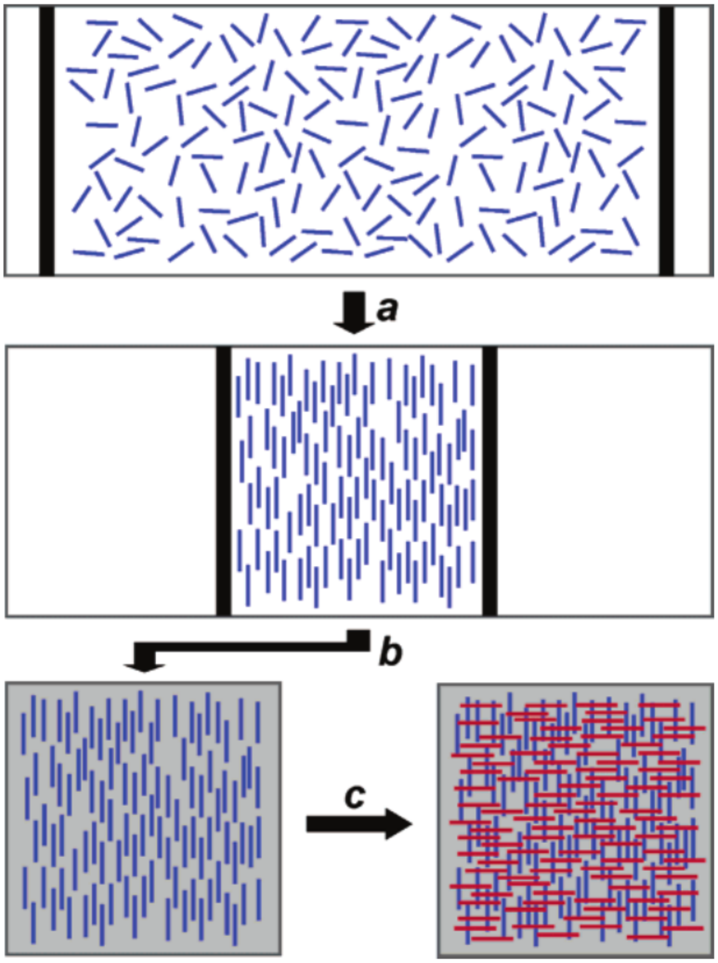

Figure 8: NWs (blue lines) in a monolayer of surfactant at the air—water interface are (a) compressed on a Langmuir—Blodgett trough to a specified pitch. (b) The aligned NWs are transferred to the surface of a substrate to make a uniform parallel array. (c) Crossed NW structures are formed by uniform transfer of a second layer of aligned parallel NWs (red lines) perpendicular to the first layer (blue lines). Adapted from Whang, D., et al., 2003 [81].

Operational parameters require optimization. Dipping/lifting speed must balance smooth transfer against practicality and monolayer stability (typically mm/min to cm/min) [96]. Compression rate during initial monolayer formation affects defect density [97]. Constant surface pressure during trans-

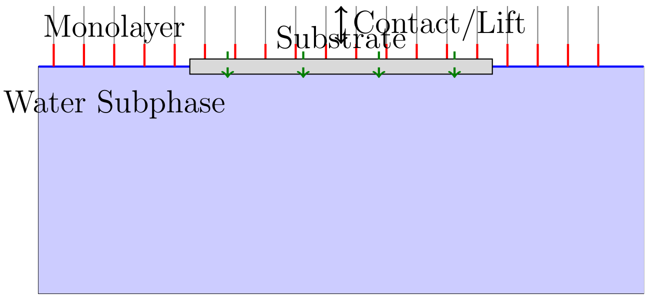

Langmuir-Schaefer Deposition

Figure 9: Schematic illustration of the horizontal lifting (Langmuir-Schaefer) deposition process. The substrate makes horizontal contact with the monolayer at the air-water interface, transferring the film upon lifting.

fer is crucial for uniformity [46]. Substrate drainage speed during LB withdrawal correlates with transfer quality [98]. Intermediate drying steps can sometimes improve multilayer formation.

3.4 Transfer Ratio and its Significance

The transfer ratio (TR) quantifies deposition efficiency and quality. It is the ratio of the Langmuir monolayer area decrease on the trough ($-\Delta A_{trough}$) to the substrate area coated ($A_{substrate}$) per pass:

$$TR = \frac{-\Delta A_{trough}}{A_{substrate}}$$

Instrumentation monitors ΔA_{trough} needed to maintain constant pressure during transfer. Ideal transfer yields TR = 1, meaning the monolayer area consumed perfectly matches the coated substrate area, indicating complete, conformal transfer without significant area changes due to rearrangement.

Deviations signal non-ideal transfer. TR ¡ 1 suggests incomplete adhesion or disruption. TR ¿ 1 might imply multilayer transfer (e.g., collapse) [99] or calibration issues. Negative TR indicates removal of previously deposited material due to poor adhesion.

Monitoring TR provides real-time feedback. Consistent TR $\approx$ 1 indicates high-quality, uniform growth. Fluctuations or low TR signal problems requiring parameter adjustments. While early researchers used area decrease

qualitatively, modern precise TR measurement is indispensable for process optimization [5, 17]. Note that even with TR ≈ 1, minor molecular packing density changes between the Langmuir monolayer and the LB film can occur, often within measurement uncertainty for well-packed films.

3.5 Techniques for Controlling Film Thickness and Uniformity

LB and LS techniques provide exceptional control over film thickness at the molecular scale. Thickness is primarily controlled by the number of deposition cycles. Each successful cycle (e.g., one up-and-down stroke in Y-type LB) adds a defined amount of material, typically a bilayer (2-5 nm). This allows fabrication of films from single monolayers to hundreds of layers with nanometer precision.

Achieving uniformity over large areas requires stable, optimized conditions throughout deposition. This includes Langmuir monolayer homogeneity and stability at the target pressure, preventing collapse or dissolution [63]. Precise control of subphase conditions (temperature [92], pH [86, 85, 100], ions [88]) and meticulous substrate preparation are vital. Consistent transfer speeds and accurate pressure maintenance via feedback control are critical.

Uniformity correlates with a consistent TR ≈ 1 for all layers. Inconsistent transfer causes thickness variations and defects. Careful solvent selection for initial spreading [51], sufficient equilibration time after compression, optimized transfer speed and pressure [101], and sometimes intermediate drying steps or surface modifications contribute to uniformity [102]. Subsequent characterization (discussed next) verifies the achieved thickness, uniformity, and structure.

4 Characterization of Langmuir-Blodgett Films

The successful fabrication and application of Langmuir-Blodgett (LB) films rely heavily on comprehensive characterization to elucidate their structure, morphology, thickness, molecular arrangement, and potential defects [103, 104, 105]. Given the nanoscale dimensions and often complex hierarchical organization of these films, a diverse suite of analytical techniques is employed, providing complementary information across different length scales

[106]. These methods range from spectroscopic probes sensitive to molecular bonding and orientation [107, 42, 108, 109], to scattering techniques revealing long-range order and layering [110, 111, 112], and microscopic imaging providing real-space visualization of the film's surface and structure [34, 113, 114, 115]. Understanding the capabilities and limitations of these techniques is crucial for correlating the deposition parameters with the resulting film properties and ultimately, their functional performance. Early studies laid the groundwork, but the advent of modern high-resolution methods, particularly synchrotron X-ray techniques and scanning probe microscopies, has revolutionized our understanding of LB film structure, revealing complexities and inhomogeneities previously inaccessible [116, 117, 118].

4.1 Spectroscopic Techniques

Spectroscopic methods are indispensable for probing the chemical composition, molecular conformation, and orientation within LB films. Fourier Transform Infrared (FTIR) spectroscopy is particularly powerful. In its simplest form, transmission FTIR provides information analogous to bulk or solution measurements, identifying characteristic vibrational modes of the constituent molecules and allowing assessment of chemical integrity and composition.

Figure 10 displays typical FTIR-ATR spectra for stearic acid LB films [120, 121, 122], showing characteristic peaks such as the carbonyl stretch (C=O) around 1700 cm^{-1} and methylene/methyl (CH_2/CH_3) stretching vibrations near 2800-3000 cm^{-1} [123, 124]. The intensity increase of these peaks with the number of deposited layers typically confirms consistent transfer and film buildup, while peak positions and shapes can offer insights into molecular packing and environment [125, 126, 127].

However, more specialized FTIR configurations yield richer structural information. Grazing Incidence Reflection-Absorption Spectroscopy (GIRAS), also known as Reflection-Absorption Infrared Spectroscopy (RAIRS), performed on reflective substrates (typically metals), exhibits high sensitivity to ultrathin films [128]. Due to the surface selection rule, only vibrational modes with a dipole moment component perpendicular to the reflective surface are strongly detected, making GIRAS exceptionally useful for determining the average orientation of molecular groups relative to the substrate [126, 129, 130]. Figure 11 shows a schematic of a typical experimental setup used for such reflection-absorption measurements. Attenuated Total Reflectance

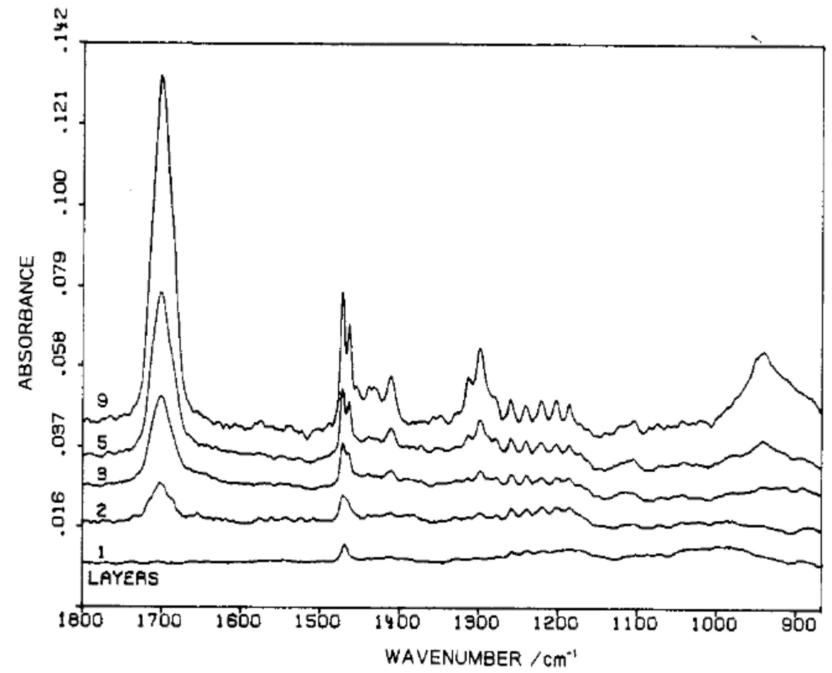

Figure 10: FTIR-ATR spectra showing the absorbance for Langmuir-Blodgett films of stearic acid deposited on a substrate, with increasing numbers of monolayers (1, 2, 3, 5, and 9 layers). Characteristic vibrational bands, such as C-H stretching modes (around 2800-3000 cm^{-1}) and the C=O stretching mode (around 1700 cm^{-1}), increase in intensity with the number of layers, indicating successful multilayer deposition. Adapted from Kimura, F., et al., 1986 [119].

(ATR) FTIR, where the infrared beam undergoes multiple internal reflections within an IR-transparent substrate coated with the LB film, also enhances surface sensitivity [131, 120]. By using polarized IR light, ATR-FTIR allows for the determination of dichroic ratios for specific absorption bands, which can be quantitatively analyzed to deduce average molecular orientation [132, 133]. Comparing transmission and reflection spectra of the same film can provide quantitative orientation data [109, 108], although careful consideration of optical effects is necessary [134]. FTIR studies have been instrumental in analyzing chain conformation (e.g., quantifying the ratio of trans to gauche conformers in alkyl chains) and verifying the structure of complex or functionalized LB films [135, 136].

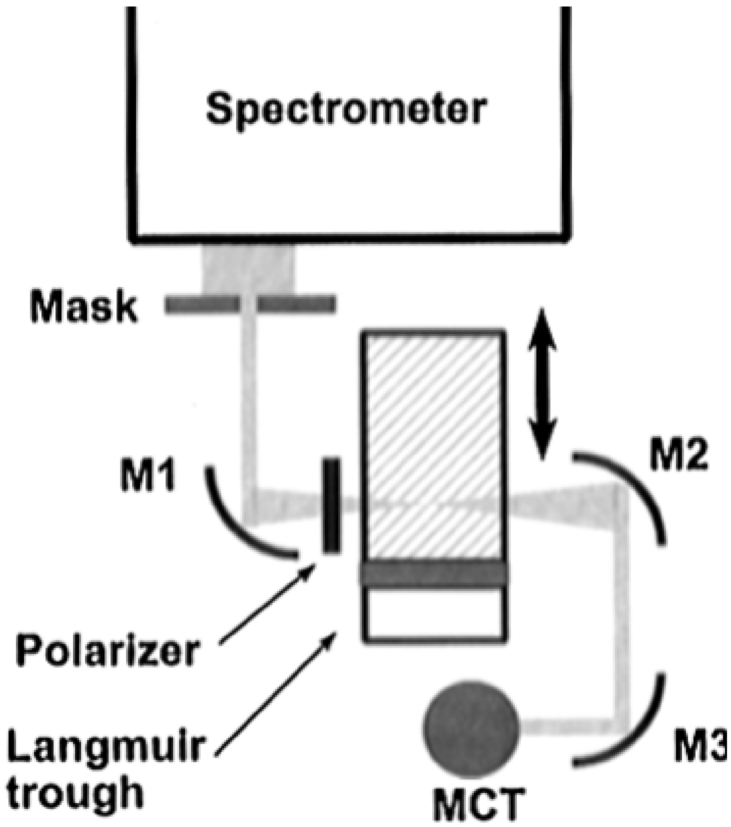

Figure 11: Schematic of the IRRAS setup. M1, M2, and M3 are off- axis parabolic mirrors. M1 and M2 each have focal lengths of 120 mm. MCT = mercury cadmium telluride detector. The arrow indicates the shuttling direction. Adapted from Flach, C., et al., 1997 [137].

Raman spectroscopy offers complementary vibrational information to FTIR. While conventional Raman scattering often suffers from low signal intensity for monolayers, Surface-Enhanced Raman Scattering (SERS) can provide enormous signal amplification when the LB film is deposited onto or near plasmonically active nanostructures (e.g., silver or gold nanoparticles or roughened surfaces) [138, 139]. SERS allows for the detection of minute quantities of material [140, 141, 142] and can provide structural information similar to FTIR, sometimes with different selection rules or sensitivities.

UV-Visible (UV-Vis) absorption spectroscopy probes electronic transitions within the molecules comprising the LB film [143, 144, 145]. It is

particularly useful for films containing chromophores [146, 147], providing information on their concentration, aggregation state (e.g., the formation of J-aggregates or H-aggregates, indicated by characteristic shifts and shape changes in the absorption bands) [148, 149], and interactions [150]. UV-Vis spectroscopy can also be used for monitoring film deposition uniformity across the substrate and, in some cases, estimating film thickness if the molar absorptivity is known.

4.2 X-ray Scattering and Diffraction Techniques

X-ray scattering techniques are paramount for determining the long-range order, layer structure, and molecular packing within LB films. X-ray Reflectivity (XRR) (illustrated in Figure 12) is a non-destructive technique that measures the specular reflection of X-rays at grazing incidence angles. The resulting interference pattern (Kiessig fringes) and the decay of reflectivity are highly sensitive to the film's electron density profile perpendicular to the surface. Analysis of XRR data yields precise measurements of the total film thickness, the thickness of individual layers in a multilayer stack, electron densities of the layers (related to material composition and packing density), and the roughness of the substrate-film and film-air interfaces, as well as internal interfaces.

X-ray Diffraction (XRD) probes the periodic arrangements of atoms and molecules [151]. For layered LB films, out-of-plane XRD measurements (often referred to as Small-Angle X-ray Scattering, SAXS, geometry) reveal Bragg peaks corresponding to the lamellar repeat distance (d-spacing) of the multilayer structure [152, 111, 153]. The positions of these (00l) reflections provide a direct measure of the bilayer or monolayer thickness, confirming the quality of the layered assembly [154]. The observation of multiple diffraction orders indicates a high degree of lamellar order [116, 110]. Remarkably, detailed structural information, including Bragg peaks and subsidiary maxima arising from interference effects in films with only a few layers, can be obtained with high-resolution measurements, providing insights into film perfection and substrate quality [155].

To probe the molecular arrangement *within* the layers (in-plane structure), Grazing Incidence X-ray Diffraction (GIXD) (illustrated in Figure 12) is employed, typically requiring high-intensity synchrotron radiation sources for sufficient signal from monolayers or thin multilayers [3, 156]. In GIXD, the incident X-ray beam impinges on the sample at a very small angle (be-

low the critical angle for total external reflection), generating an evanescent wave that propagates parallel to the surface, thus enhancing surface sensitivity. Diffracted beams exiting the surface provide information about the lateral packing of the molecules [157, 158]. Analysis of GIXD patterns reveals the two-dimensional unit cell parameters of the molecular lattice within the layers, the coherence length (related to the size of ordered domains), and, by analyzing the position and intensity distribution along the Bragg rods (diffraction normal to the surface), the tilt angle and direction of the molecules relative to the surface normal [3, 71]. Techniques like GIXD [159, 160] and orientation-sensitive spectroscopy [130, 161, 126] can yield detailed models of molecular arrangement, as schematically illustrated for cadmium stearate in Figure 13.

Other X-ray based techniques also provide valuable information. X-ray Photoelectron Spectroscopy (XPS) identifies the elemental composition and chemical bonding states at the film surface (typically probing the top few nanometers) [162, 163] but requires ultrahigh vacuum conditions. Near-Edge X-ray Absorption Fine Structure (NEXAFS) spectroscopy [164], often performed using synchrotron radiation [165], is sensitive to the orientation of specific chemical bonds relative to the polarization of the incident X-rays, providing complementary orientation information to FTIR [166].

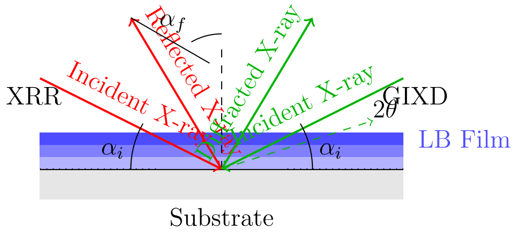

Figure 12: Schematic illustration of X-ray Reflectivity (XRR) and Grazing Incidence X-ray Diffraction (GIXD) geometries for characterizing LB films. In XRR, the specular reflection intensity is measured as a function of the incident angle α_i ($\alpha_f = \alpha_i$). In GIXD, the incident beam strikes at a fixed grazing angle α_i, and diffraction is measured at various in-plane (2θ) and out-of-plane (α_f) angles.

4.3 Microscopic Techniques

Microscopic techniques provide direct real-space visualization of the LB film's surface morphology, structure, and defects. Scanning Probe Microscopy (SPM) techniques have become particularly crucial. Atomic Force Microscopy (AFM) is arguably the most widely used technique for LB film characterization. It generates topographical maps of the surface with nanometer or even sub-nanometer vertical resolution and high lateral resolution, capable of resolving molecular lattices under optimal conditions. AFM can be operated in various environments, including ambient air and liquids, making it suitable for studying films under different conditions. It provides quantitative information on surface roughness, domain structures, and the presence and dimensions of defects, revealing surface complexities and inhomogeneities such as domain boundaries, pinholes, cracks, layer stacking faults, variations in molecular packing density across the film, and step heights corresponding to individual molecular layers. Friction Force Microscopy (FFM), often performed concurrently with AFM, measures the lateral forces between the scanning tip and the surface. FFM is sensitive to variations in material properties and surface chemistry, allowing it to differentiate between domains of different composition or molecular orientation that may not be apparent in the topography image. Scanning Tunneling Microscopy (STM) offers the highest lateral resolution, capable of imaging individual atoms and molecules. However, its application is generally restricted to conductive or semiconducting substrates and films, limiting its use for typical insulating LB films, although studies on monolayers or bilayers on suitable substrates have yielded valuable insights into molecular packing.

Electron microscopy techniques are also employed, though sometimes limited by sample preparation requirements or potential beam damage. Scanning Electron Microscopy (SEM) provides images of the surface topography, typically with lower resolution than AFM but over larger areas. For insulating LB films, a conductive coating is often required, or low-voltage/low-vacuum modes must be used to prevent charging artifacts. SEM is useful for assessing overall film coverage, large-scale morphology, and macroscopic defects. Transmission Electron Microscopy (TEM) requires electron-transparent samples. LB films can be transferred onto TEM grids or studied in cross-section (requiring complex preparation like ultramicrotomy). TEM offers very high resolution imaging of the film's internal structure or morphology. The Transmission Electron Diffraction (TED) mode within a TEM

provides crystallographic information (lattice parameters, symmetry) from selected small areas of the film [167, 168].

Optical microscopy techniques offer complementary information, often over larger areas and sometimes non-invasively. Polarizing optical microscopy can visualize micrometer-sized domains in thicker LB films that exhibit birefringence due to anisotropic molecular arrangement. Brewster Angle Microscopy (BAM) is a powerful non-invasive technique primarily used to visualize monolayers *at the air-water interface* during their formation and compression, revealing domain structures, phase transitions, and homogeneity [169, 170, 171, 172]. It relies on the principle that p-polarized light incident at the Brewster angle is not reflected from a pure water surface, but the presence of a monolayer alters the reflectivity, creating contrast. While less common for transferred films, it can still provide information. Fluorescence microscopy requires the incorporation of fluorescent probes or the use of intrinsically fluorescent molecules. It is widely used to visualize domain morphology, phase separation in mixed monolayers [62], and dynamic processes like lateral diffusion (using techniques like Fluorescence Recovery After Photobleaching, FRAP) [173], although the probe itself might perturb the film structure. Differential Interference Contrast (DIC) or Nomarski microscopy enhances contrast based on optical path differences, making it useful for visualizing subtle height variations and surface features in LB films.

4.4 Ellipsometry

Optical ellipsometry is a non-destructive technique widely used for accurately determining the thickness and optical constants (refractive index and extinction coefficient) of thin films, including LB films [174, 175]. It measures the change in the polarization state of light upon reflection from the sample surface. By analyzing this change, typically using an optical model that describes the sample structure (substrate, film layers), the thickness and refractive index of the film can be extracted with sub-nanometer precision for thickness. Single-wavelength ellipsometry provides thickness information assuming a known refractive index, while spectroscopic ellipsometry, which measures the polarization change over a range of wavelengths, allows for the simultaneous determination of both thickness and wavelength-dependent optical constants [176, 161]. This provides more detailed information about the material's electronic structure and can help resolve more complex multilayer structures. Ellipsometry is valuable for routine characterization of film

thickness and uniformity after deposition and can also be implemented for *in situ* monitoring during film growth or modification [177].

4.5 Other Characterization Methods

Several other techniques provide specialized information about LB films. Surface potential measurements, often performed using a Kelvin probe, measure the change in work function upon film deposition. This measurement is sensitive to the net dipole moment perpendicular to the surface, arising from the molecules themselves and their arrangement, as well as any surface charges. It provides insights into molecular orientation and interfacial electronic structure. The Quartz Crystal Microbalance (QCM) technique measures changes in the resonant frequency of a quartz crystal oscillator upon deposition of the LB film onto its surface. The frequency change is directly proportional to the added mass, allowing for highly sensitive measurement of the mass deposited per layer (areal density) and monitoring of the deposition process in real-time. QCM with dissipation monitoring (QCM-D) can also provide information about the viscoelastic properties of the film.

Neutron Reflectivity (NR) is analogous to XRR but uses neutrons instead of X-rays [178, 179]. Its key advantage lies in the strong difference in scattering length between hydrogen and deuterium [180, 181]. By selectively deuterating components of the LB film or the surrounding medium (e.g., water vapor), NR can provide unique structural information, such as the precise location of specific molecular groups, the extent of interdigitation between layers, or the penetration of solvent molecules into the film structure, which can be difficult to obtain with X-rays [182, 159, 183].

Non-linear optical techniques like Second Harmonic Generation (SHG) [184, 185, 186, 184] and Sum Frequency Generation (SFG) spectroscopy [187, 188] are inherently interface-specific. SHG is sensitive to media lacking inversion symmetry, making it a powerful probe for monitoring molecular orientation and order at surfaces and interfaces [189], particularly for non-centrosymmetric molecular arrangements often targeted in LB films for optical applications [190, 191]. SFG spectroscopy combines an infrared and a visible laser beam to generate light at the sum frequency [192]. It is vibrationally specific, providing information about the orientation and conformation of specific molecular groups directly at the interface [193, 194], similar to IR spectroscopy but with intrinsic surface specificity.

These techniques are particularly valuable for probing buried interfaces or

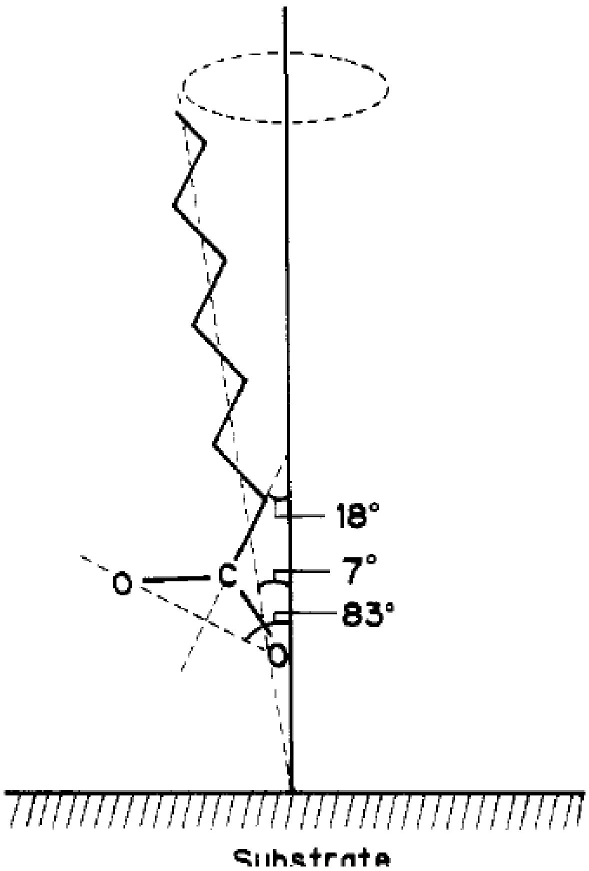

Figure 13: Schematic illustration of molecular orientation in 7-monolayer LB film of cadmium stearate. Adapted from Umumera, J., et al., 1990 [108].

for *in situ* studies. Collectively, this array of characterization tools provides a powerful means to unravel the intricate details of LB film structure and properties, guiding their rational design and application.

5 Applications of Langmuir-Blodgett Films

The unique ability of the Langmuir-Blodgett technique to fabricate ultra-thin films with precisely controlled thickness, composition, and molecular architecture has opened avenues for a wide array of applications across diverse scientific and technological domains [6, 1, 5]. The capacity to assemble molecules layer-by-layer allows for the creation of highly ordered functional

interfaces desirable for electronics, sensing, optics, and biomaterials, although historical challenges related to stability, defects, and scalability have limited widespread commercialization, LB films remain invaluable for research and proof-of-concept demonstrations, inspiring materials science and nanotechnology advancements [32]. The versatility in material choice, ranging from classical amphiphiles like fatty acids to polymers, nanoparticles, and complex biomolecules, further broadens the scope of potential applications [7, 195, 196]. This section explores some of the most significant areas where LB films have made contributions or hold considerable promise, including molecular electronics, sensors, optics, and biomaterials.

5.1 Molecular Electronics

The concept of using individual molecules or molecular assemblies as active components in electronic devices, often termed molecular electronics, emerged partly from the need to overcome the perceived miniaturization limits of conventional silicon-based technology. Langmuir-Blodgett films, offering nanometer-scale control over film structure, were identified early on as a promising platform for realizing molecular electronic components. Initial research efforts in the 1970s through the 1990s were significantly motivated by the prospect of creating devices like molecular rectifiers, as theoretically proposed, requiring the precise arrangement of specifically designed molecules, a level of control achievable with the LB technique. Indeed, rectification behaviour was demonstrated in metal/LB film/metal structures incorporating specific molecular designs, such as zwitterionic molecules, fueling expectations for a new paradigm in electronics, as noted in reviews by Oliveira et al. However, translating these concepts into practical devices faced significant hurdles. Fabricating defect-free molecular assemblies over large areas proved challenging, as did establishing reliable electrical contacts between the molecular layer and macroscopic electrodes without damaging the delicate film structure. Addressing the top contact electrode deposition remained a persistent difficulty in fabricating functional molecular junctions based on LB films.

Despite these challenges, LB films have found utility in the development of organic electronic devices, particularly organic field-effect transistors (OFETs) [19, 197]. The LB technique allows for the deposition of ultrathin, ordered layers of active semiconductor materials, such as conjugated polymers or functionalized nanomaterials, onto substrate surfaces [1, 20]. For

instance, regioregular poly(3-hexylthiophene) (RR-PHT), a well-known conjugated polymer, has been successfully deposited as LB films to fabricate OFETs [197, 198]. The controlled deposition can lead to improved molecular ordering within the film compared to other solution-processing techniques, potentially enhancing charge carrier mobility. Studies have shown that the morphology and molecular arrangement within the RR-PHT LB films, influenced by the deposition method, directly impact the resulting transistor performance, achieving mobilities comparable to or exceeding those of many other polymeric thin-film devices fabricated using LB methods. Similarly, monolayers of molecules like octadecyltrichlorosilane (ODTS), transferred using modified LB techniques, can serve as high-quality gate dielectric layers or surface modification layers in OFETs. The ability to deposit uniform, defect-minimized monolayers over large areas, even on flexible substrates, is advantageous for producing arrays of devices with consistent performance, as demonstrated by uniform mobility maps across wafer-scale OFET arrays based on ODTS monolayers. Figure 14 shows a schematic of a typical OFET structure incorporating an LB film. This bottom-gate, top-contact configuration, as illustrated in the work of Xu et al., typically consists of a substrate (e.g., silicon) acting as the base, upon which a gate electrode is patterned. A dielectric layer (e.g., silicon dioxide or a functional LB layer like ODTS) insulates the gate from the active semiconductor layer, which is deposited as an LB film (e.g., RR-PHT). Finally, source and drain electrodes are deposited on top of the LB semiconductor layer to complete the transistor structure.

Beyond transistors, the insulating properties of certain LB films, particularly those made from fatty acids or other long-chain aliphatic molecules, have been explored for applications in thin-film capacitors. The precise control over thickness down to the nanometer scale allows for the fabrication of ultrathin dielectric layers [9]. Early studies investigated the potential of LB films in metal-insulator-semiconductor (MIS) [199] and metal-insulator-metal (MIM) structures. While initial work often suffered from issues related to film defects (pinholes) and electrical stability, the potential for creating highly perfect insulating layers was recognized. Concepts such as "self-healing" capacitors, where electrical breakdown in one layer might be isolated, were explored, although practical implementation remained limited. Polymerized LB films were proposed to enhance the mechanical and thermal stability of these dielectric layers [200, 201].

While the initial vision of complex molecular circuits built entirely using LB films has not materialized into widespread technology, the technique con-

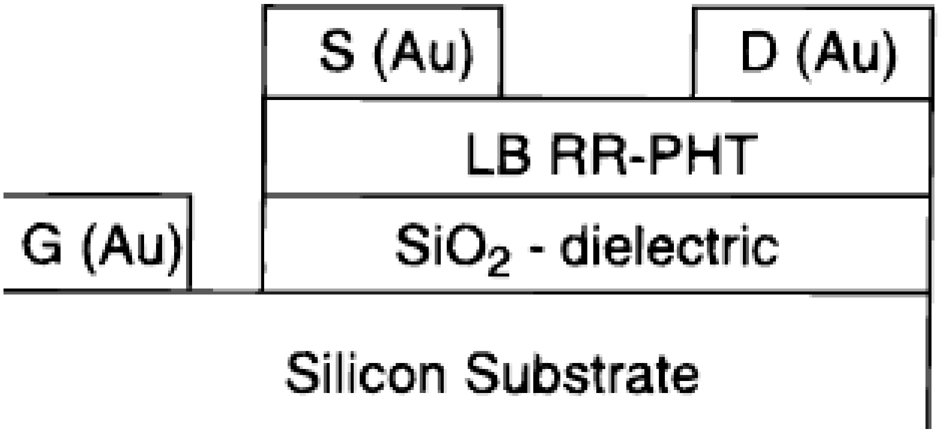

Figure 14: Schematic diagram illustrating the typical architecture of a bottom-gate, top-contact Organic Field-Effect Transistor (OFET) incorporating an LB film as the active semiconductor layer. Adapted from Xu, G., et al., 2000 [197].

tinues to be a valuable tool for fabricating and studying fundamental aspects of organic and molecular electronic devices [202, 203]. It provides a unique method for controlling molecular organization and film thickness [1, 2], enabling research into structure-property relationships crucial for advancing the field of organic electronics [10].

5.2 Sensors

The ability to create highly ordered thin films with controlled composition and thickness makes the Langmuir-Blodgett technique particularly well-suited for the fabrication of chemical and biological sensors. Sensing mechanisms often rely on interactions occurring at interfaces, and LB films provide a platform to precisely engineer these interfaces at the molecular level. The technique allows for the incorporation of a wide variety of sensing materials, including conductive polymers, nanoparticles, porphyrins, phthalocyanines, carbon nanomaterials, metal oxides, and biomolecules, often arranging them in structures that optimize their interaction with target analytes. The ultrathin nature of LB films facilitates rapid diffusion of analytes to the active sensing layer, potentially leading to faster response times compared to thicker films. Furthermore, the high degree of molecular order achievable can

enhance sensor sensitivity and selectivity.

Gas sensing represents a major application area for LB films. Early work, documented in reviews such as those by Oliveira et al. and Vincett, focused on using LB films of conducting polymers or macrocyclic compounds like phthalocyanines [204] and porphyrins [39], where changes in electrical conductivity or optical properties upon exposure to specific gases formed the basis of detection. More recently, research has expanded to include nanomaterials and composite films. For example, LB films incorporating polyaniline-functionalized multi-walled carbon nanotubes (PANI@MWCNTs) have been developed for ammonia (NH_3) detection [205]. The LB technique helps to overcome the aggregation tendency of CNTs and facilitates the formation of ordered films, enhancing sensor performance. Composite LB films containing nanosheets of black phosphorus modified with dyes have demonstrated sensitivity to acidic (HCl) and alkaline (NH_3) gases [206], exploiting the different responses of dye aggregates (H-type and J-type) formed within the ordered LB structure. Fatty acid LB films (e.g., arachidic acid, stearic acid) have been employed as sensitive coatings on surface acoustic wave (SAW) devices for the detection of volatile organic compounds like chloroform [207], showing enhanced selectivity compared to other solvents. The number of deposited layers can be precisely controlled to optimize sensitivity. Metal oxide nanowires, such as ZnO, can also be assembled into aligned arrays using LB techniques, creating films suitable for humidity sensing.

The alignment achieved via LB deposition can significantly influence the sensing properties compared to randomly oriented nanowire films. Figure 15 illustrates a typical automated experimental setup for testing gas sensors based on SAW devices coated with LB films, as depicted by Gu et al. This setup typically includes a gas source (e.g., an air generator) providing a carrier gas, flow meters (FM) to precisely control the flow rates of the carrier gas and the analyte vapor stream, valves (V1, V2, V3) for switching between pure carrier gas and analyte-containing gas mixtures, a bubbler or similar device to generate a controlled concentration of analyte vapor, and a measurement chamber housing the SAW sensor coated with the LB sensing film. The controlled gas mixture flows through the chamber, allowing interaction with the sensor, and the sensor's response is monitored.

In the realm of biosensing, LB films offer a powerful method for immobilizing biological recognition elements, such as enzymes or antibodies, onto transducer surfaces while maintaining their biological activity [208]. The ordered structure can provide a favorable microenvironment and po-

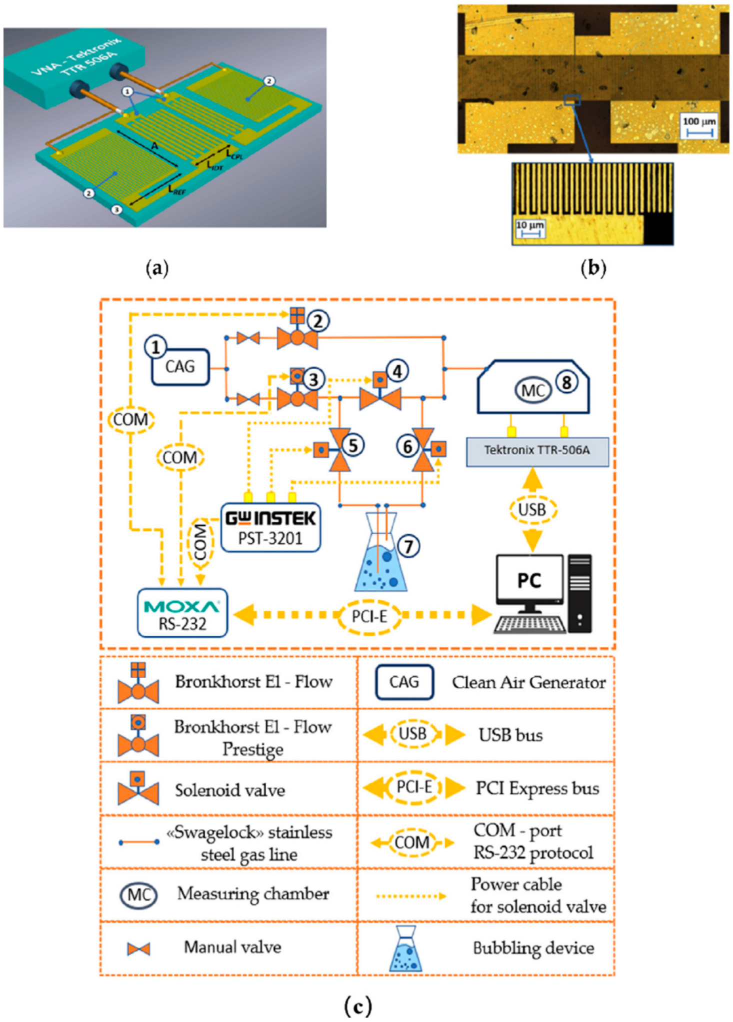

Figure 15: Schematic diagram of an automated measurement setup for gas sensing using Surface Acoustic Wave (SAW) devices coated with LB films. Controlled gas mixtures are passed through the measurement chamber containing the sensor. Adapted from Gu, W., et al., 2024 [5].

tentially control the orientation of the biomolecules, enhancing accessibility to target analytes. Enzyme-based biosensors have been developed using LB films containing, for example, glucose oxidase (GOx) for glucose detection [209, 210]. Composite LB films incorporating GOx and gold nanoparticles (AuNPs) have been explored, with different architectures (e.g., mixed lay-

ers, alternating layers) influencing the electrochemical response to glucose [211]. Immobilizing GOx within lipid monolayers, such as dipalmitoylphosphatidylethanolamine (DPPE), using the LB technique has also been shown to produce functional glucose sensors where film conductivity changes with glucose concentration [210]. Immunosensors based on LB films have also been demonstrated [212, 213]. Antibodies, such as Immunoglobulin G (IgG), can be transferred as LB films onto electrode surfaces [214, 215]. Studies have shown that LB-immobilized IgG can retain antigen-binding capabilities, potentially offering higher binding efficiency compared to traditional physical adsorption or chemical immobilization methods due to the controlled deposition and layering. Composite LB films of materials like molybdenum disulfide (MoS_2) and gold nanoparticles have been used as platforms for immobilizing antibodies specific to dengue non-structural protein 1 (NS1) antigen, creating sensors for dengue fever detection [216]. The combination of nanomaterials and biomolecules within the LB structure allows for sensitive signal transduction.

The versatility of the LB technique in incorporating diverse materials and controlling film architecture makes it a valuable platform for developing advanced sensors with improved sensitivity, selectivity, and response times for applications in environmental monitoring [5, 204], medical diagnostics [217, 218], and industrial process control [40, 43].

5.3 Optics

The precise control over film thickness and refractive index afforded by the Langmuir-Blodgett technique makes it attractive for various optical applications. The ability to construct multilayer structures with alternating materials or specific molecular orientations allows for the engineering of films with tailored optical properties. Historically, one of the earliest proposed applications was the use of fatty acid salt LB films as antireflection coatings, leveraging the ability to create layers with specific thicknesses related to the wavelength of light. Furthermore, the pioneering work by Kuhn and colleagues demonstrated the use of LB films to build complex supramolecular assemblies incorporating dye molecules, enabling fundamental studies of energy transfer processes between molecular layers separated by precisely controlled distances. These studies highlighted the potential of LB films as model systems for understanding and manipulating light-matter interactions at the nanoscale.

Integrated optics, which deals with guiding and manipulating light within thin films, is another area where LB films have been investigated [219, 220]. Fatty acid LB films, as well as films made from polymerizable amphiphiles, have been shown to function as optical waveguides [221]. The thickness of the LB film can be adjusted by the number of deposition cycles to meet the requirements for light confinement, and the refractive index can be tuned to some extent by the choice of molecule or by incorporating metal ions. Early experiments demonstrated waveguiding in fatty acid LB films with relatively low optical losses, particularly when using polymerizable materials to enhance film stability and quality. While achieving the thicknesses required for practical waveguiding (often hundreds of layers) can be challenging due to potential defect accumulation in thicker films, LB films have been proposed as effective post-fabrication cladding layers. Depositing a precisely controlled LB film onto a pre-existing waveguide allows for fine-tuning of its propagation characteristics, which is useful for devices like planar couplers or sensors. The ability to incorporate optically active molecules, such as dyes, into the LB structure also opens possibilities for creating active optical components like planar laser structures or mode filters, although challenges related to photostability and efficiency remain [222].

Nonlinear optics (NLO) is another field where the molecular ordering capabilities of LB films are potentially advantageous. Materials exhibiting second-order NLO effects require a non-centrosymmetric structure. The LB technique allows for the construction of such structures by depositing specific molecules in an oriented fashion or by building alternating layer structures (Y-type vs. Z-type or alternating) [74, 75]. Molecules like certain dyes [190, 185, 186, 223, 224], functionalized polymers [225, 226], or poly(diacetylenes) formed by polymerizing LB films of diacetylene amphiphiles [220, 227] have been investigated for NLO properties such as second harmonic generation (SHG) [228, 185, 186, 223, 224]. While promising results have been obtained in laboratory settings, practical applications have been limited by factors such as achieving sufficient long-range order, phase-matching, and material stability [13]. Functionalized phthalocyanines [229, 230, 231, 232, 233], known for their robustness, have also been incorporated into LB films and studied for their optical and electro-optical properties. Despite the challenges, LB films continue to serve as valuable model systems for studying NLO phenomena in highly organized molecular assemblies.

5.4 Biomaterials and Biomimicry

Langmuir-Blodgett films hold significant potential in the field of biomaterials and biomimicry due to their structural similarity to biological membranes and their ability to incorporate and organize biological molecules. Biological membranes, composed primarily of lipid bilayers with embedded proteins, are inherently two-dimensional structures that mediate crucial cellular processes. The LB technique provides a means to create artificial systems that mimic these natural structures under controlled laboratory conditions.

One of the most prominent applications is the use of LB films as model biological membranes [48, 234, 235]. Phospholipids, cholesterol, and other lipid components of natural membranes are often amphiphilic and can readily form stable Langmuir monolayers at the air-water interface [33]. These monolayers can be studied directly or transferred onto solid supports as LB films, often composed of single or multiple lipid layers, which serve to model or mimic the structure of biological lipid bilayers, as discussed in works by Schwartz and Oliveira et al. These model systems allow researchers to investigate fundamental aspects of membrane structure [236, 237], phase behavior [238], lipid-protein interactions [239], and the effects of drugs or other molecules on membrane properties using surface-sensitive techniques [240, 49]. Techniques like surface pressure-area isotherms, spectroscopy [241, 242], microscopy, and scattering methods can be applied to characterize these artificial membranes. By varying the lipid composition or incorporating specific proteins or peptides into the LB film (either by co-spreading with the lipids before forming the Langmuir monolayer or by adsorption from the subphase beneath the monolayer), researchers can create tailored models to address specific biological questions, such as the mechanisms of membrane fusion, ion transport, or signal transduction. The LB approach offers precise control over molecular density, orientation, and composition, providing advantages over other model membrane systems like liposomes for certain types of surface-sensitive studies.

Beyond fundamental membrane studies, LB films are being explored for more direct biomaterial applications. The controlled deposition of biocompatible polymers or bioactive molecules using the LB technique could be utilized in drug delivery systems [243]. While less explored compared to other applications, the potential exists to create ultrathin coatings or layered structures for controlled release applications, possibly leveraging the ordered structure to tune release kinetics.

Tissue engineering is another emerging area where the structural control offered by LB films may be beneficial. The ability to create surfaces with specific topographies and chemical functionalities at the nanoscale can influence cell adhesion, proliferation, and differentiation [244, 245, 246]. For instance, as highlighted by Oliveira et al., LB films composed of aligned nanomaterials, such as fullerene (C_{60}) nanowhiskers, have been investigated as scaffolds for cell culture [247, 248, 249]. The LB technique allows for the controlled alignment of these nanowhiskers into a one-dimensional structure on the substrate. Studies have shown that human mesenchymal stem cells cultured on such aligned LB scaffolds exhibit guided growth along the nanowhisker direction and maintain their crucial properties of self-renewal and multipotency for extended periods [250, 251, 252, 253]. This indicates that the precisely controlled nanoscale topography provided by the LB assembly acts as an effective physical cue, influencing cell behavior and fate, which is a key goal in designing advanced scaffolds for tissue regeneration or sophisticated cell culture platforms [254, 255].

Furthermore, the incorporation of enzymes or antibodies into LB films, as discussed in the context of biosensors, also represents an application in functional biomaterials [26, 256, 257, 258]. These proteo-lipidic nanostructures can serve as platforms for studying enzyme kinetics in membrane-mimicking environments [259] or for developing bioelectronic devices where biological recognition is coupled to an electronic signal output [260, 261]. The LB technique allows for the immobilization of these biomolecules in an organized fashion, potentially enhancing their stability and functional activity compared to random immobilization methods. The development of robust and functional biomimetic LB films remains an active area of research with implications for diagnostics, therapeutics, and fundamental biological understanding [262].

6 Advanced Topics and Emerging Trends

The Langmuir-Blodgett (LB) technique, while rooted in early 20th-century surface science, continues to evolve, driven by the demands of modern nanotechnology and materials science [1, 5, 17]. While initial studies predominantly focused on simple amphiphilic molecules like fatty acids [6, 263], the scope of LB has significantly broadened [32, 45]. Researchers are increasingly exploring advanced materials [4, 28], sophisticated control over molecular ar-

chitecture [264, 265], innovative deposition methodologies [266, 267], and integration with other fabrication techniques to unlock new functionalities and applications in emerging technological fields [43, 44].

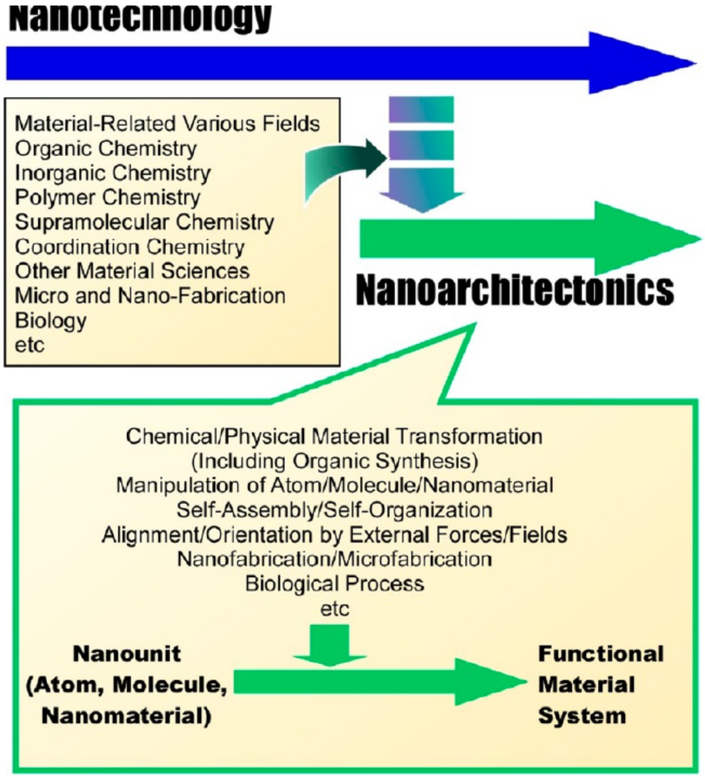

Figure 16: Outline of nanoarchitectonics concept. Adapted from Ariga, K., 2023 [45].

This progression marks a shift from using LB films primarily as model systems to harnessing their unique capabilities for creating functional devices and complex material interfaces. The inherent precision of the LB method at the molecular level makes it an invaluable tool in the nanoarchitectonics paradigm, which involves assembling nanoscale structural units into functional materials and systems. Figure 16 visually represents this concept, illustrating how individual nanoscale components can be organized into larger, functional architectures. This paradigm is highly relevant to advanced LB film fabrication, enabling the construction of highly ordered structures from a diverse palette of building blocks, extending far beyond traditional

amphiphiles. The exploration of these advanced topics and emerging trends underscores the enduring relevance and adaptability of LB technology in contemporary scientific research and technological development.

6.1 Novel Materials for Langmuir-Blodgett Films

The expansion of LB technology into new application domains has been largely fueled by the successful incorporation of non-traditional film-forming materials. While early work established the principles using fatty acids and other small amphiphiles, the limitations of these materials in terms of mechanical robustness and functional properties (e.g., electrical, optical) spurred investigations into alternatives. Polymers represented an early and significant advancement. Polymeric LB films can be fabricated using several strategies: (i) transferring a Langmuir film from the monomers onto the solid support, after which polymerization is realized; (ii) polymerizing monomers *in situ* within the Langmuir film at the air-water interface before transfer; or (iii) spreading pre-formed polymers directly to form the Langmuir film, which is then transferred. These approaches yield films with enhanced mechanical stability and diverse functionalities, suitable for applications like sensors and electronic devices developed extensively since the 1980s.

In recent years, the focus has shifted dramatically towards nanomaterials. The LB technique has proven remarkably versatile in organizing nanoscale building blocks at the air-water interface and transferring them as ordered films [1, 28, 4]. Carbon-based nanomaterials, including fullerenes, graphene, graphene oxide (GO), and carbon nanotubes (CNTs), have been successfully assembled using LB methods [268, 269, 270]. For instance, GO, owing to its amphiphilic nature arising from oxygen-containing functional groups, readily forms Langmuir films whose density and structure can be controlled via compression [271, 272]. Studies have investigated the phase transitions of GO Langmuir films using surface pressure-area isotherms and elastic modulus profiling [273]. However, correlating isotherm features directly with film morphology (e.g., wrinkles, overlaps) remains challenging for soft 2D materials like GO, primarily because the changes in elastic modulus during phase transitions can be subtle compared to those of traditional amphiphiles, often necessitating microscopic analysis for confirmation. Techniques combining modified CNTs, such as polyaniline-functionalized multi-walled carbon nanotubes (PANI@MWCNTs), with polymers have been used to create composite LB films with reduced aggregation and improved alignment, beneficial

for sensor applications [205, 274]. Single-walled carbon nanotubes (SWCNTs) have also been incorporated into polymer composites via LB, with the resulting film order depending on the SWCNT synthesis method [275, 276].

Beyond carbon nanomaterials, a wide array of inorganic and hybrid materials have been integrated into LB films. Metal nanoparticles (e.g., Au, Ag, Pt) [277, 278], semiconductor nanoparticles and quantum dots (e.g., CdSe, SnO_2), and magnetic nanoparticles (e.g., Fe_3O_4) [279] can be assembled into monolayers or multilayers. The LB compression allows tuning of interparticle spacing, density, and arrangement, influencing collective properties like plasmon coupling in metallic nanoparticle films [280, 141, 281, 140]. Inorganic nanowires (e.g., ZnO, Si, Ge, MnO_2) and nanorods (e.g., Au, $BaCrO_4$) [282] have also been organized using LB techniques, often resulting in aligned arrays over large areas. Two-dimensional materials beyond graphene, such as black phosphorus nanosheets (BPNS) [283, 206] and molybdenum disulfide (MoS_2), have been functionalized and assembled into LB films, sometimes in combination with dyes or nanoparticles, for applications in sensors and optoelectronics [284]. Metal-organic frameworks (MOFs) [285] and covalent organic frameworks (COFs) [286, 28], known for their high porosity and tunable structures, are emerging materials for LB film fabrication, offering potential in catalysis, sensing, and separation. Even complex biological molecules and structures, functionalized to possess amphiphilic character or co-spread with lipids, can be incorporated into Langmuir and LB films [261, 260].

Despite the successes, working with these novel materials presents challenges. Solubility in suitable volatile solvents can be problematic. Achieving uniform spreading at the air-water interface without significant aggregation or formation of 3D structures instead of monolayers requires careful optimization of solvents, spreading techniques [51], and subphase conditions. Furthermore, the rigidity or aggregation state of the Langmuir film can impede efficient transfer to solid substrates. Addressing these challenges often involves chemical modification of the nanomaterials (e.g., surface functionalization), using mixed solvent systems, employing co-spreading agents (like polymers or traditional amphiphiles), or developing alternative deposition protocols. The successful incorporation of this diverse range of materials significantly expands the functional capabilities of LB films, enabling applications in electronics [287], photonics, energy storage, catalysis, and biomedicine [243].

6.2 Advanced Control over Film Architecture

A defining characteristic and principal advantage of the LB technique is the high degree of control it offers over the structure and organization of the resulting thin films, often down to the molecular level. This control stems from the ability to manipulate the state of the precursor monolayer at the air-water interface prior to transfer. The surface pressure-area (π-A) isotherm provides critical information about the phase behavior of the monolayer during compression, allowing researchers to select specific phases (e.g., liquid-expanded, liquid-condensed, solid) with desired packing densities and molecular arrangements for transfer. By carefully controlling the surface pressure during deposition, the density and intermolecular spacing within the transferred LB film can be precisely tuned.

This control extends to the orientation of molecules or nanoscale objects within the film. For amphiphilic molecules, the interaction with the subphase and the compression process naturally induce orientation, typically with hydrophilic headgroups towards the water and hydrophobic tails towards the air. Upon transfer, this orientation is largely preserved. For anisotropic nanomaterials like nanowires or nanorods, the compression process and the hydrodynamics during transfer can induce alignment. For example, LB deposition has been used to create highly aligned arrays of ZnO nanowires [288] and other one-dimensional nanostructures over large areas. The degree of alignment can often be quantified using imaging techniques combined with orientation analysis. Similarly, studies on SWCNT-polymer composites have shown that the LB process can lead to ordered arrangements of the nanotubes within the polymer matrix [205].

Furthermore, the LB technique allows for the construction of complex multilayer structures with controlled layer sequencing and thickness [73]. By repeatedly dipping the substrate through the monolayer, multilayer films can be built up layer by layer [12, 289]. Using alternating troughs with different materials or modifying the subphase between deposition steps enables the fabrication of heterostructures with precisely defined composition and architecture in the dimension perpendicular to the substrate. This layer-by-layer control is crucial for creating devices requiring specific interfacial properties or spatially varying functionalities, such as quantum wells, superlattices, or complex sensor architectures.

The LB method also provides a unique environment for controlling intermolecular interactions and self-assembly processes within the two-dimensional

confinement of the air-water interface [28]. For certain molecules, particularly dyes, compression can induce the formation of specific aggregates, such as H-aggregates (face-to-face stacking, often leading to blue-shifted absorption) or J-aggregates (head-to-tail stacking, often leading to red-shifted, sharp absorption bands) [290]. The formation of these aggregates can be controlled by factors like surface pressure, subphase composition (pH, ions), and temperature [116, 90]. As a pertinent example, composite Langmuir films formed from modified black phosphorus nanosheets (BPNS) and specific dyes were prepared at the air-water interface. Upon compression and transfer, these formed LB films containing distinct H- and J-type aggregates. Crucially, these different aggregate structures exhibited distinct optical responses when exposed to acidic (HCl) and alkaline (NH_3) gases, demonstrating how controlling interfacial assembly via LB can tune the functional properties of the resulting film for applications like gas sensing. This ability to direct supramolecular assembly at the interface adds another layer of control over the film's properties [265]. The precise structural control afforded by the LB technique, encompassing packing density, molecular orientation, multilayer architecture, and directed self-assembly, remains a key advantage for fabricating highly ordered and functional thin films for advanced applications [1, 5, 17].

6.3 Innovations in Langmuir-Blodgett Deposition Methods

While the fundamental principles of LB deposition established by Langmuir and Blodgett remain central, ongoing research has led to innovations and modifications of the technique to address limitations, accommodate new materials, or achieve specific film structures. These advancements aim to improve film quality, expand the range of applicable materials, enhance deposition speed or area, and enable fabrication under non-standard conditions.

One area of innovation involves adapting the LB technique for materials that are difficult to handle using conventional methods. For instance, highly conjugated molecules or polymers, which are often desirable for electronic applications, may exhibit strong intermolecular attractions, leading to aggregation and poor film formation at room temperature. To overcome this, a high-temperature LB method has been developed [291, 292]. By using a high-boiling-point subphase like ethylene glycol instead of water, depo-

sition can be performed at temperatures up to or exceeding 100 °C. This elevated temperature can enhance molecular mobility, disrupt undesirable aggregation, and promote the formation of highly crystalline and oriented films, as demonstrated for poly[2,5-bis(3-tetradecylthiophene-2-yl)thiophene] (P3TT). Films prepared using this method exhibited significantly improved charge transport properties due to enhanced orientation and crystallinity.

Another novel approach deviates from the quasi-static compression typically employed in LB troughs. The "vortex LB" method, illustrated conceptually in Figure 17, utilizes vigorous rotational flow on the liquid surface to assist in the formation and deposition of uniform films, particularly from materials that might otherwise aggregate non-uniformly [293, 28]. This dynamic flow helps to distribute the material evenly across the interface before transfer. This technique was successfully used to create uniform carbon nanofilms from nanoring molecules spread on a rotating subphase, leading to homogeneous films even with complex molecular structures [294]. Subsequent high-temperature treatment of these films resulted in conductive, nitrogen-doped nanocarbon layers suitable for applications like fuel cells.

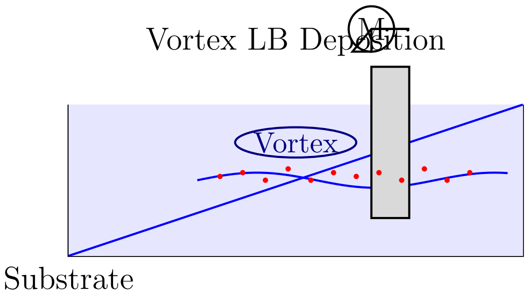

Figure 17: Conceptual illustration of the vortex LB method, where rotational flow in the subphase aids in the uniform distribution and assembly of film-forming materials prior to transfer onto a substrate. This dynamic approach contrasts with traditional quasi-static compression methods.

Efforts have also been directed towards scaling up the LB process for larger areas and potentially higher throughput, addressing a common criticism regarding its suitability for industrial applications. While traditional research troughs handle substrates of limited size, modifications involving trough design [295, 296], barrier systems, and automated dipping mechanisms have enabled deposition onto larger substrates, such as 3-inch or 4-inch wafers, as demonstrated in the fabrication of organic field-effect transistor

(OFET) arrays based on octadecyltrichlorosilane (ODTS) monolayers. Although true roll-to-roll LB deposition remains challenging and less developed compared to techniques like Layer-by-Layer (LbL) assembly [297, 11] or solution coating [298], the ability to achieve uniform deposition over wafer-scale areas represents a significant step towards practical applicability. The compatibility of LB with large-area fabrication is often cited as an advantage for potential applications [20, 299].

Furthermore, variations in the transfer geometry itself exist. While the vertical dipping (Langmuir-Blodgett) method is most common, horizontal transfer methods like the Langmuir-Schaefer (LS) technique, where the substrate touches the monolayer horizontally, are also widely used [291]. LS deposition is often preferred for transferring rigid monolayers or for depositing onto hydrophobic surfaces [282, 300], and it typically results in only monolayer deposition per transfer cycle. The choice between LB and LS depends on the specific material, substrate, and desired film structure. These innovations in deposition methodology demonstrate the continued refinement of the LB technique, expanding its capabilities and addressing some of its perceived limitations.

6.4 Emerging Technological Frontiers

The unique capabilities of LB films, particularly the precise control over structure and the ability to incorporate diverse functional materials, position them as enabling components in several emerging technological areas. While challenges related to stability and scalability persist for widespread industrial adoption, LB films serve as powerful platforms for proof-of-concept studies and niche applications.

One significant area is flexible and organic electronics. The ability to deposit highly ordered films of organic semiconductors, conducting polymers, or nanomaterials onto flexible substrates using modified LB techniques is attractive for creating devices like Organic Field-Effect Transistors (OFETs) [19, 18]. For example, defect-free monolayers of ODTS transferred onto silicon wafers via LB served as high-quality dielectric layers for OFET arrays, demonstrating uniform performance over large areas. Similarly, the high-temperature LB method yielded highly oriented semiconducting polymer films with enhanced charge mobility, crucial for efficient transistor operation [292, 291]. The precise thickness control offered by LB is also beneficial for optimizing device performance in thin-film transistors and other layered

electronic structures [20].

Energy storage, particularly in the context of rechargeable batteries, represents another frontier. LB methods are being explored for fabricating advanced battery components [72, 17, 5]. Nanostructured electrode materials with high surface area and controlled architecture can be prepared. For instance, nanocomposites of SnO_2 nanoparticles and reduced graphene oxide (RGO) sheets were fabricated layer-by-layer using LB transfer, resulting in binder-free anodes for lithium-ion batteries with high capacity [269, 268]. Hybrid multilayers of graphene nanosheets and SWCNTs, prepared via LB, also showed promise as high-performance anodes [301, 302, 303]. Beyond electrodes, LB films can be used to create functional coatings, such as protective layers for sensitive electrode materials like lithium metal [304]. Furthermore, LB-assembled structures serve as model systems for battery diagnosis. Aligned MnO_2 nanowire arrays fabricated by LB were integrated into on-chip microbatteries; the crucial *alignment* achieved through the LB technique enabled *in situ* monitoring of structural and conductivity changes during electrochemical cycling, providing valuable insights into capacity fading mechanisms and electrode degradation [305].

The historical connection between LB films and molecular electronics continues, albeit with a more nuanced perspective. While the original vision of complex molecular circuits built entirely with LB films remains largely unrealized due to fabrication challenges and stability issues, the technique is still valuable for fundamental studies and creating components for organic electronics [202, 10, 306]. LB films provide well-defined structures for investigating charge transport across molecular layers and interfaces [37, 307, 308]. They have been used to create large-area molecular electronic devices [20] and to study phenomena like rectification in metal/LB film/metal junctions [309]. The combination of LB films with nanomaterials like BPNS and dyes has shown potential for enhanced photoelectrochemical performance, relevant for light harvesting and conversion applications [206].

Advanced sensors, building upon the applications discussed previously, also benefit from novel materials and structures enabled by LB. The use of MOFs [310, 311], BPNS [206], functionalized CNTs [312], and precisely controlled nanoparticle assemblies in LB films allows for the development of highly sensitive and selective gas sensors [313, 314, 315], chemical sensors [229, 284, 204], and biosensors [26, 260, 261]. The ordered structure and high surface area achievable with LB films can enhance analyte interaction and signal transduction. For example, LB films incorporating MOFs have

shown promise for detecting H_2O_2 with high sensitivity and stability [316].

These emerging applications highlight the adaptability of the LB technique. By integrating novel materials and leveraging precise structural control, LB films contribute to advancing technologies ranging from flexible displays and wearable electronics to next-generation batteries and sophisticated sensing platforms.

6.5 Synergistic Integration with Other Nanofabrication Techniques

The Langmuir-Blodgett technique, while powerful on its own, can be significantly enhanced when combined synergistically with other nanofabrication methods. This integration allows for the creation of more complex, hierarchical, or patterned structures that leverage the strengths of multiple techniques. Such hybrid approaches expand the possibilities for designing functional materials and devices beyond what can be achieved with LB alone.

One common pairing is the combination of LB with Layer-by-Layer (LbL) assembly. While LB excels at creating highly ordered monolayers of often insoluble amphiphiles or nanoparticles, LbL is adept at building robust multilayer films from water-soluble species (like polyelectrolytes or charged nanoparticles) through alternating adsorption, typically driven by electrostatic interactions. These techniques can be used sequentially or complementarily. For example, an ordered base layer could be deposited by LB, followed by functional multilayers built via LbL. Alternatively, materials prepared by LB, such as reduced graphene oxide (RGO) sheets, can be incorporated into LbL-assembled structures, as seen in the fabrication of SnO_2/RGO composite electrodes where both techniques were employed in alternation. This combination allows for intricate control over both in-plane order (from LB) and layer composition/thickness (from LbL).

LB films can also serve as effective masks or templates for subsequent processing steps like etching. Monolayers of nanoparticles (e.g., silica spheres) assembled at the air-water interface using LB can be transferred onto a substrate to act as a lithographic mask. Subsequent reactive ion etching (RIE) can then transfer the pattern defined by the nanoparticle array into the underlying substrate or film. This approach has been used to fabricate arrays of silicon nanopillars by etching a silicon wafer masked by an LB film of SiO_2 nanoparticles. A variation involved using an LB nanoparticle mask on a poly-

imide film, followed by RIE steps to create vertically aligned nanochannels, which were then used to host lithium metal for stabilized battery anodes. This combination of bottom-up LB assembly and top-down etching enables the creation of well-defined periodic nanostructures over large areas.

Self-assembly processes, both at the air-water interface during Langmuir film formation and post-deposition, are often integral to LB-based fabrication. The LB technique itself relies on the self-assembly of molecules or particles at the interface driven by surface tension and intermolecular forces. The controlled formation of specific molecular aggregates (like H- or J-aggregates, discussed in 6.2) within Langmuir films is a prime example of directed self-assembly within the LB framework, which can be exploited for functional tuning.

Furthermore, LB deposition can be combined with chemical modification or synthesis steps. As mentioned earlier, polymerization of monomers within Langmuir or LB films is a well-established hybrid approach. Post-deposition treatments, such as thermal annealing (e.g., to reduce GO in LB films) or chemical cross-linking, can be used to modify the properties or enhance the stability of LB-fabricated structures.

These examples illustrate that LB is not an isolated technique but rather a versatile tool within the broader nanofabrication toolbox. By combining LB with LbL, etching, directed self-assembly, and chemical synthesis/modification, researchers can create sophisticated materials and device architectures with enhanced functionality and complexity, pushing the boundaries of nanotechnology.

7 Challenges and Future Directions

Despite nearly a century of development and demonstrated versatility across numerous scientific domains, the widespread practical application of Langmuir-Blodgett (LB) film technology faces several persistent challenges. Addressing these limitations while exploring new frontiers defines the future trajectory of this sophisticated thin-film fabrication technique. The inherent precision offered by LB methods in controlling molecular arrangement and film thickness remains a key driver for continued research, yet translating this control into robust, scalable, and cost-effective applications requires overcoming significant hurdles.

One of the primary challenges historically associated with LB films, par-

ticularly those derived from traditional small amphiphilic molecules like fatty acids, as highlighted in reviews discussing the historical limitations of LB films derived from such materials, is their mechanical fragility and limited long-term stability [6, 5, 17]. While these films serve as excellent model systems for fundamental studies, their lack of robustness often precludes their use in demanding real-world environments. Significant efforts have been directed towards enhancing mechanical strength and stability. The incorporation of preformed polymers [7, 317] or the *in situ* polymerization of monomeric Langmuir films represents a major strategy to create more resilient structures [318]. Polymer LB films generally exhibit improved mechanical properties compared to their small-molecule counterparts [200]. Furthermore, the integration of nanomaterials, such as carbon nanotubes, graphene oxide sheets, or inorganic nanoparticles, into the film structure can impart enhanced mechanical integrity and functional properties [319, 274, 320]. Chemical cross-linking of molecules within the deposited layers is another approach explored to stabilize the film structure post-deposition. However, ensuring long-term stability under operational conditions, including exposure to varying temperatures, humidity, or chemical environments, remains an area requiring further investigation and material innovation [321]. The amphiphilic nature of many LB film components, particularly the long aliphatic chains essential for monolayer formation at the air-water interface, can also act as insulating barriers, impeding electrical transport perpendicular to the film plane. This limitation is particularly relevant for electronic applications requiring efficient charge injection or transport across multiple layers. Strategies to mitigate this include the design of novel amphiphiles with conductive moieties or the incorporation of 'molecular wires', such as conjugated oligomers or polymers, intended to bridge the insulating gaps between functional layers [195, 322].

The scalability and throughput of the LB deposition process present another significant barrier to industrial adoption. The classical LB technique, involving the slow, layer-by-layer transfer of monolayers from a Langmuir trough onto a substrate, is inherently a low-throughput process. While suitable for laboratory-scale research and fabrication of specialized devices, it is often considered too slow and costly for mass production compared to alternatives like layer-by-layer (LbL) assembly or self-assembled monolayers (SAMs) [11, 323]. However, as noted by reviews comparing these techniques, neither LbL nor SAMs typically offer the same degree of precise control over molecular orientation and packing density, justifying the use of LB for appli-

cations demanding superior structural order [17, 6]. Developing novel instrumentation and methodologies for continuous or large-area LB deposition is crucial for enhancing throughput [324, 296]. Research into alternative deposition geometries or automated multi-substrate systems aims to address this challenge, potentially making LB technology more competitive for specific high-value applications where molecular precision is paramount. Compatibility with large-area substrates and integration with existing manufacturing processes are key considerations for future development.

Bridging the gap between the wealth of fundamental knowledge generated through LB research and tangible practical applications remains a central goal. While LB films have been instrumental in advancing our understanding of surface science, interfacial phenomena, molecular electronics, and biomimetic systems, their transition into commercial products has been limited. The stringent requirements for film perfection, the complexity of the deposition process, and concerns about stability and cost have often favored other techniques for industrial applications. Future efforts should focus on identifying niche applications where the unique advantages of LB films—precise thickness control, defined molecular orientation, ability to create complex multilayer architectures, and compatibility with diverse materials—outweigh the associated challenges. Areas such as highly specific chemical and biological sensors [21, 325], specialized optical coatings, model systems for biological membrane studies, templates for nanostructure fabrication, and components in molecular electronic devices represent promising avenues. Demonstrating reliability, reproducibility, and cost-effectiveness in these targeted applications is essential for fostering wider adoption. The concept of nanoarchitectonics [326, 327, 328, 329], which leverages the precise assembly capabilities of techniques like LB to build functional systems from nanoscale components, provides a powerful framework for designing next-generation materials and devices.

The exploration of new materials and increasingly complex architectures continues to expand the potential of LB technology. The initial focus on simple fatty acids has broadened dramatically to include polymers, proteins, dendrimers, liquid crystals, nanoparticles (metallic, semiconducting, magnetic), nanowires, carbon nanotubes, graphene, graphene oxide, metal-organic frameworks (MOFs), covalent organic frameworks (COFs), and various hybrid materials. Figure 18, for instance, showcases how LB techniques enable the precise assembly of diverse nanomaterials, such as the depicted nanocrystals and nanowires, into highly ordered close-packed monolayers

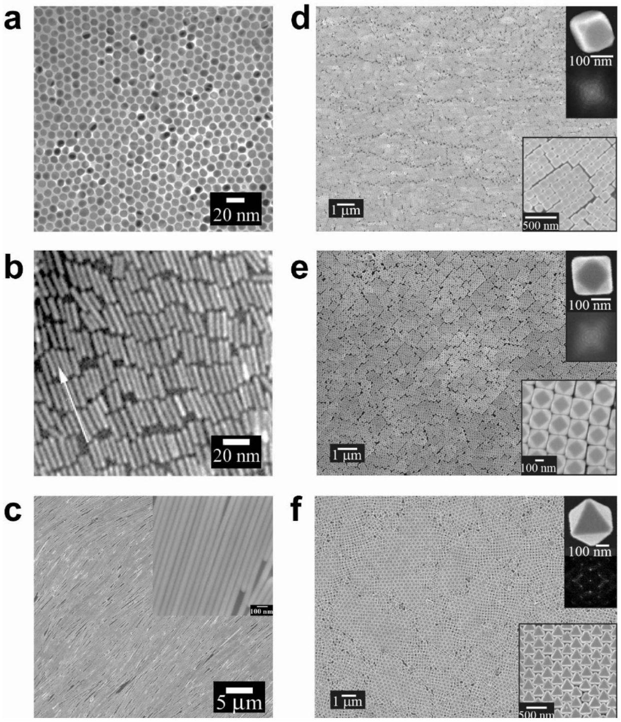

Figure 18: Selected examples of close-packed Langmuir—Blodgett monolayers of nanocrystals and nanowires: (a) Pt nanocrystal arrays use as 2D model catalysts; (b) $BaCrO_4$ nanorods arranged into liquid crystalline patterns; (c) densely packed Ag nanowire arrays for molecular sensing using surface-enhanced Raman spectroscopy—the inset shows an area of close-packed nanowires; (d—f) superlattice architectures assembled from Ag nanocrystal building blocks of different polyhedral shapes, (d) truncated cubes, (e) cuboctahedra, and (f) octahedra. The insets in panels d—f show individual nanocrystal geometry (top), fast Fourier transform images indicating long-range order within each monolayer (middle), and close-up views of the nanocrystal unit cell (bottom). Panels a and b are TEM images, while the microscopy graphs in (c—f) are SEM images. Adapted from Tao, A., et al., 2008 [330].

suitable for applications like model catalysis or surface-enhanced Raman spectroscopy sensing. Similarly, Figure 19 illustrates the application of interfacial assembly strategies, potentially combining LB principles with layer-by-layer approaches as suggested in the caption, to structure complex materials

like metal-organic frameworks (MOFs) into defined monolayers, expanding the technique far beyond traditional amphiphiles. This diversification allows

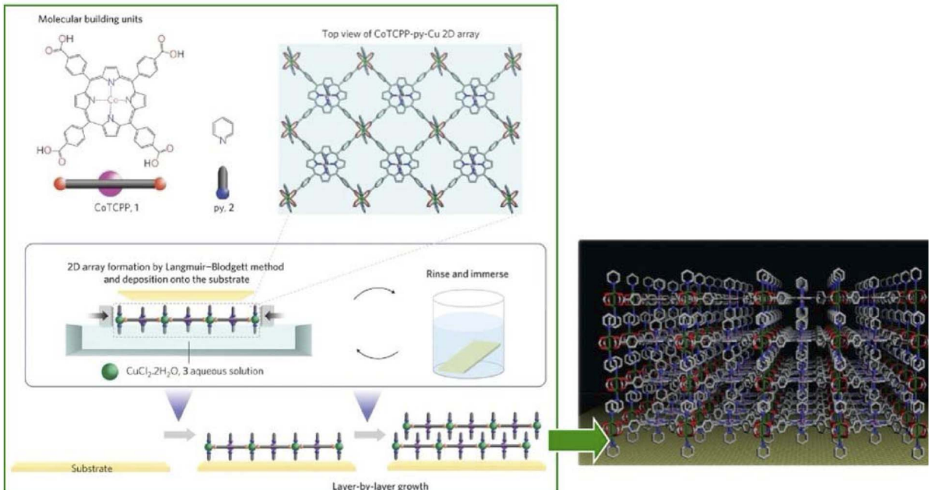

Figure 19: MOF monolayers obtained by developing 5,10,15,20-tetrakis(4-carboxyphenyl)porphyrinato-cobalt(II) and pyridine on a solution of $CuCl_2$:$2H_2O$ in chloroform/methanol that is multilayered by a sequential LbL stacking procedure Adapted from Song, Jingwen, et al., 2024 [331].

for the creation of films with tailored electronic, optical, magnetic, catalytic, and biological properties. Designing amphiphilic molecules specifically for LB deposition, potentially incorporating multiple functional units, offers a route to multifunctional films. Furthermore, the ability to construct multi-layer heterostructures with different materials in sequential layers, precisely controlling the composition and interface at the molecular level, is a unique strength of the LB technique, enabling the fabrication of complex organized supramolecular assemblies designed for specific functions, such as energy transfer cascades or multi-step catalytic reactions. The air-water interface itself, used for Langmuir monolayer formation, provides a unique environment for studying molecular recognition, self-assembly, and mimicking biological processes. As emphasized in recent reviews, this capability to model cell membrane interactions with drugs or proteins using Langmuir monolayers at the air-water interface is a distinct advantage unmatched by other techniques. Exploiting phenomena at this interface remains a highly promising direction, potentially leading to advancements in drug delivery, diagnostics, and fundamental biology.

Finally, the integration of computational methods, including artificial intelligence (AI) and machine learning (ML), holds significant potential for accelerating progress in LB film research and application. AI/ML algorithms can be employed for tasks such as the inverse design of novel amphiphilic molecules with desired properties, prediction of their behavior at the air-water interface, and optimization of LB deposition parameters (e.g., surface pressure, dipping speed, subphase conditions) for achieving specific film structures and functionalities. Simulations, particularly Molecular Dynamics (MD) [52] often coupled with Density Functional Theory (DFT) calculations, provide powerful tools for understanding molecular interactions, film stability, and transport properties within LB assemblies, complementing experimental characterization. AI can also assist in analyzing complex data from characterization techniques (e.g., microscopy, spectroscopy, scattering) to extract detailed structural information, perform tasks like property prediction or experimental parameter optimization, and correlate structure with film performance. This synergy between computational modeling and experimental work can guide material discovery, streamline process optimization, and ultimately facilitate the rational design of LB films for targeted applications, potentially shortening the path from fundamental research to practical implementation. The future of LB technology likely lies in leveraging its unique precision for specialized, high-value applications, continuously innovating in materials and methods, and integrating computational tools to overcome existing limitations.

8 Conclusion

This review has synthesized the evolution and current state of Langmuir and Langmuir-Blodgett (LB) film technology, emphasizing the foundational importance of understanding amphiphilic molecule behavior at fluid interfaces [33] and the meticulous nature of monolayer transfer techniques (LB and Langmuir-Schaefer) for constructing nanostructured films [2, 32, 6]. We have highlighted how a diverse suite of characterization tools, from surface manometry and microscopy [35, 34] to spectroscopy and scattering methods [241, 130], is essential for probing the intricate structural details, molecular orientation [109, 108], and overall quality of these nanometer-scale layers. The mastery over film formation and deposition translates directly into the ability to engineer materials with specific properties, layer by layer [11, 18].

The distinct advantages of this technology have catalyzed progress across numerous scientific frontiers. We examined how the fine-tuned assembly offered by LB methods significantly benefits sensor development, particularly for gas sensing [332, 40, 207], by enabling optimized architectures for sensitivity and selectivity. In electrochemistry, these films provide invaluable platforms for investigating interfacial electron transfer and modifying electrode functionalities. Furthermore, the unique capability of Langmuir monolayers to mimic biological interfaces makes them indispensable tools in biomimicry [33], facilitating studies of membrane biophysics [237], drug interactions [49], and the creation of sophisticated biosensors [218, 210] and biocompatible surfaces [243]. Emerging applications in energy storage [305, 319, 320] further underscore the versatility of LB techniques for engineering critical interfaces and materials.

While alternative approaches like self-assembled monolayers (SAMs) and layer-by-layer (LbL) assembly present practical benefits in robustness and scalability for specific industrial uses, Langmuir and LB methods occupy a vital niche. The Langmuir technique's unique capacity for studying dynamic interfacial phenomena, molecular recognition, and two-dimensional self-assembly in situ remains unparalleled. Concurrently, the LB method's fidelity in creating complex, predefined molecular architectures ensures its continued relevance for fundamental research and specialized applications demanding nanoscale architectural control. These techniques are powerful enabling tools within the nanoarchitectonics paradigm, allowing the integration of diverse components—polymers, biomolecules, and nanomaterials such as graphene or nanoparticles—into functional hierarchical structures.

Building upon this established significance, the future trajectory of Langmuir and Langmuir-Blodgett film research holds considerable promise, especially in areas demanding exquisite control at the molecular scale. Ongoing improvements in instrumentation, the development of novel film-forming materials, and the refinement of characterization techniques will undoubtedly expand the capabilities and applications of this technology. The synergy between LB methods and advanced nanomaterials is poised to drive the development of next-generation electronics, optoelectronics, highly specific sensors, and efficient energy systems. In the life sciences, the ability to construct sophisticated biomimetic interfaces ensures that Langmuir and LB films will remain crucial for advancing biophysical understanding, drug discovery platforms, and biomedical device engineering. Although challenges concerning large-area uniformity, defect mitigation, and industrial scalability persist, the

fundamental knowledge generated through LB research and the specialized technological avenues it opens will continue to fuel innovation. The increasing integration with computational modeling, such as molecular dynamics, will further enhance predictive design capabilities. In essence, Langmuir and Langmuir-Blodgett techniques, originating from fundamental surface science explorations, remain foundational pillars in nanoscience and materials engineering, essential for advancing materials design and enabling technological breakthroughs through the controlled construction of matter at the molecular level.

References

[1] Katsuhiko Ariga et al. "25th Anniversary Article: What Can Be Done with the Langmuir-Blodgett Method? Recent Developments and its Critical Role in Materials Science". In: *Advanced Materials* 25.45 (2013), 6477–6512. ISSN: 1521-4095. DOI: 10.1002/adma.201302283. URL: http://dx.doi.org/10.1002/adma.201302283.

[2] María Mercedes Velázquez et al. "Langmuir-Blodgett Methodology: A Versatile Technique to Build 2D Material Films". In: *Two-dimensional Materials - Synthesis, Characterization and Potential Applications*. InTech, 2016. ISBN: 9789535125556. DOI: 10.5772/63495. URL: http://dx.doi.org/10.5772/63495.

[3] Didier Jacquemain et al. "Two-Dimensional Crystallography of Amphiphilic Molecules at the Air–Water Interface". In: *Angewandte Chemie International Edition in English* 31.2 (1992), 130–152. ISSN: 0570-0833. DOI: 10.1002/anie.199201301. URL: http://dx.doi.org/10.1002/anie.199201301.

[4] Jin Young Park and Rigoberto C. Advincula. "Nanostructuring polymers, colloids, and nanomaterials at the air–water interface through Langmuir and Langmuir–Blodgett techniques". In: *Soft Matter* 7.21 (2011), p. 9829. ISSN: 1744-6848. DOI: 10.1039/c1sm05750b. URL: http://dx.doi.org/10.1039/c1sm05750b.

[5] Wenhui Gu et al. "Recent Progress in the Applications of Langmuir–Blodgett Film Technology". In: *Nanomaterials* 14.12 (2024), p. 1039. ISSN: 2079-4991. DOI: 10.3390/nano14121039. URL: http://dx.doi.org/10.3390/nano14121039.

[6] Thi Thao Vu et al. “EVALUATION OF RESEARCH PROGRESS, TRENDS, AND APPLICATIONS OF LANGMUIR-BLODGETT FILMS OF FATTY ACIDS”. In: *ChemChemTech* 68.2 (2024), 6–45. ISSN: 0579-2991. DOI: 10.6060/ivkkt.20256802.7002. URL: http://dx.doi.org/10.6060/ivkkt.20256802.7002.

[7] Frank Embs et al. “Preformed polymers for Langmuir–Blodgett films–molecular concepts”. In: *Advanced Materials* 3.1 (1991), 25–31. ISSN: 1521-4095. DOI: 10.1002/adma.19910030106. URL: http://dx.doi.org/10.1002/adma.19910030106.

[8] Kazue Kurihara. “Langmuir-Blodgett films in advanced molecular engineering”. In: *Colloids and Surfaces A: Physicochemical and Engineering Aspects* 123–124 (1997), 425–432. ISSN: 0927-7757. DOI: 10.1016/s0927-7757(96)03831-9. URL: http://dx.doi.org/10.1016/s0927-7757(96)03831-9.

[9] D. T. Amm et al. “Fabrication of ultrathin metal oxide films using Langmuir–Blodgett deposition”. In: *Applied Physics Letters* 61.5 (1992), 522–524. ISSN: 1077-3118. DOI: 10.1063/1.107875. URL: http://dx.doi.org/10.1063/1.107875.

[10] Harald Fuchs, Holger Ohst, and Werner Prass. “Ultrathin organic films: Molecular Architectures for advanced optical, electronic and bio-related systems”. In: *Advanced Materials* 3.1 (1991), 10–18. ISSN: 1521-4095. DOI: 10.1002/adma.19910030103. URL: http://dx.doi.org/10.1002/adma.19910030103.

[11] Katsuhiko Ariga et al. “Layer-by-layer Nanoarchitectonics: Invention, Innovation, and Evolution”. In: *Chemistry Letters* 43.1 (2013), 36–68. ISSN: 1348-0715. DOI: 10.1246/cl.130987. URL: http://dx.doi.org/10.1246/cl.130987.

[12] Gero Decher and Jong-Dal Hong. “Buildup of ultrathin multilayer films by a self-assembly process, 1 consecutive adsorption of anionic and cationic bipolar amphiphiles on charged surfaces”. In: *Makromolekulare Chemie. Macromolecular Symposia* 46.1 (1991), 321–327. ISSN: 0258-0322. DOI: 10.1002/masy.19910460145. URL: http://dx.doi.org/10.1002/masy.19910460145.

[13] Dawn Y. Takamoto et al. "Stable Ordering in Langmuir-Blodgett Films". In: *Science* 293.5533 (2001), 1292–1295. ISSN: 1095-9203. DOI: 10.1126/science.1060018. URL: http://dx.doi.org/10.1126/science.1060018.

[14] J. B. Peng et al. "The structure of ultrathin Langmuir-Blodgett films of cadmium behenate". In: *The Journal of Chemical Physics* 123.21 (2005). ISSN: 1089-7690. DOI: 10.1063/1.2131067. URL: http://dx.doi.org/10.1063/1.2131067.

[15] Daniel K. Schwartz. "Langmuir-Blodgett film structure". In: *Surface Science Reports* 27.7 (1997), pp. 245–334. ISSN: 0167-5729. DOI: https://doi.org/10.1016/S0167-5729(97)00003-4. URL: https://www.sciencedirect.com/science/article/pii/S0167572997000034.

[16] Jerome D. Swalen et al. "Molecular monolayers and films. A panel report for the Materials Sciences Division of the Department of Energy". In: *Langmuir* 3.6 (1987), pp. 932–950. ISSN: 0743-7463. DOI: 10.1021/la00078a011. URL: https://doi.org/10.1021/la00078a011.

[17] Wenhui Gu et al. "Recent Progress in the Applications of Langmuir–Blodgett Film Technology". In: *Nanomaterials* 14.12 (2024), p. 1039. ISSN: 2079-4991. DOI: 10.3390/nano14121039. URL: http://dx.doi.org/10.3390/nano14121039.

[18] Omar Azzaroni et al. "Field-effect transistors engineered via solution-based layer-by-layer nanoarchitectonics". In: *Nanotechnology* 34.47 (2023), p. 472001. ISSN: 1361-6528. DOI: 10.1088/1361-6528/acef26. URL: http://dx.doi.org/10.1088/1361-6528/acef26.

[19] J. Paloheimo et al. "Molecular field-effect transistors using conducting polymer Langmuir–Blodgett films". In: *Applied Physics Letters* 56.12 (1990), 1157–1159. ISSN: 1077-3118. DOI: 10.1063/1.103182. URL: http://dx.doi.org/10.1063/1.103182.

[20] Lucía Herrer, Santiago Martín, and Pilar Cea. "Nanofabrication Techniques in Large-Area Molecular Electronic Devices". In: *Applied Sciences* 10.17 (2020), p. 6064. ISSN: 2076-3417. DOI: 10.3390/app10176064. URL: http://dx.doi.org/10.3390/app10176064.

[21] Antonio Riul et al. "Nano-Assembled Films for Taste Sensor Application". In: *Artificial Organs* 27.5 (2003), 469–472. ISSN: 1525-1594. DOI: 10.1046/j.1525-1594.2003.07243.x. URL: http://dx.doi.org/10.1046/j.1525-1594.2003.07243.x.

[22] C. Medina-Plaza, J.A. de Saja, and M.L. Rodriguez-Mendez. "Bioelectronic tongue based on lipidic nanostructured layers containing phenol oxidases and lutetium bisphthalocyanine for the analysis of grapes". In: *Biosensors and Bioelectronics* 57 (2014), 276–283. ISSN: 0956-5663. DOI: 10.1016/j.bios.2014.02.023. URL: http://dx.doi.org/10.1016/j.bios.2014.02.023.

[23] Anerise de Barros et al. "Electrochemical sensors based on carbon nanomaterial using Langmuir–Blodgett and layer-by-layer thin films for chemical and biological analyses". In: *Electrochemical Sensors Based on Carbon Composite Materials*. IOP Publishing, 2022, 12–1–12–16. DOI: 10.1088/978-0-7503-5127-0ch12. URL: http://dx.doi.org/10.1088/978-0-7503-5127-0ch12.

[24] Tommy Dam et al. "Supported Lipid Bilayers and the Study of Two-Dimensional Binding Kinetics". In: *Frontiers in Molecular Biosciences* 9 (2022). ISSN: 2296-889X. DOI: 10.3389/fmolb.2022.833123. URL: http://dx.doi.org/10.3389/fmolb.2022.833123.

[25] Helmut Ringsdorf, Bernhard Schlarb, and Joachim Venzmer. "Molecular Architecture and Function of Polymeric Oriented Systems: Models for the Study of Organization, Surface Recognition, and Dynamics of Biomembranes". In: *Angewandte Chemie International Edition in English* 27.1 (1988), 113–158. ISSN: 0570-0833. DOI: 10.1002/anie.198801131. URL: http://dx.doi.org/10.1002/anie.198801131.

[26] Tetsu Tatsuma et al. "Bifunctional Langmuir-Blodgett film for enzyme immobilization and amperometric biosensor sensitization". In: *Thin Solid Films* 202.1 (1991), 145–150. ISSN: 0040-6090. DOI: 10.1016/0040-6090(91)90550-h. URL: http://dx.doi.org/10.1016/0040-6090(91)90550-h.

[27] A.P. Girard-Egrot, R.M. Morélis, and P.R. Coulet. "Bioactive nanostructure with glutamate dehydrogenase associated with LB films: protecting role of the enzyme molecules on the structural lipidic organization". In: *Thin Solid Films* 292.1–2 (1997), 282–289. ISSN: 0040-6090.

DOI: 10.1016/s0040-6090(96)08954-7. URL: http://dx.doi.org/10.1016/s0040-6090(96)08954-7.

[28] Katsuhiko Ariga et al. "Materials nanoarchitectonics at two-dimensional liquid interfaces". In: *Beilstein Journal of Nanotechnology* 10 (2019), 1559–1587. ISSN: 2190-4286. DOI: 10.3762/bjnano.10.153. URL: http://dx.doi.org/10.3762/bjnano.10.153.

[29] Katsuhiko Ariga. "Molecular Tuning Nanoarchitectonics for Molecular Recognition and Molecular Manipulation". In: *ChemNanoMat* 6.6 (2020), 870–880. ISSN: 2199-692X. DOI: 10.1002/cnma.202000137. URL: http://dx.doi.org/10.1002/cnma.202000137.

[30] Makoto Komiyama et al. "Chemistry Can Make Strict and Fuzzy Controls for Bio-Systems: DNA Nanoarchitectonics and Cell-Macromolecular Nanoarchitectonics". In: *Bulletin of the Chemical Society of Japan* 90.9 (2017), 967–1004. ISSN: 1348-0634. DOI: 10.1246/bcsj.20170156. URL: http://dx.doi.org/10.1246/bcsj.20170156.

[31] Tomohiro Murata et al. "Nanometer-Flat DNA-Featured Thin Films Prepared via Laser Molecular Beam Deposition under High-Vacuum for Selective Methanol Sensing". In: *Bulletin of the Chemical Society of Japan* 96.1 (2022), 29–34. ISSN: 1348-0634. DOI: 10.1246/bcsj.20220303. URL: http://dx.doi.org/10.1246/bcsj.20220303.

[32] Katsuhiko Ariga. "Don't Forget Langmuir–Blodgett Films 2020: Interfacial Nanoarchitectonics with Molecules, Materials, and Living Objects". In: *Langmuir* 36.26 (2020), 7158–7180. ISSN: 1520-5827. DOI: 10.1021/acs.langmuir.0c01044. URL: http://dx.doi.org/10.1021/acs.langmuir.0c01044.

[33] Katsuhiko Ariga and Jonathan P. Hill. "Monolayers at air-water interfaces: from origins-of-life to nanotechnology". In: *The Chemical Record* 11.4 (2011), 199–211. ISSN: 1528-0691. DOI: 10.1002/tcr.201100004. URL: http://dx.doi.org/10.1002/tcr.201100004.

[34] D. K. Schwartz, R. Viswanathan, and J. A. Zasadzinski. "Examining Langmuir-Blodgett Films with Atomic Force Microscopy". In: *Science* 263.5150 (1994), 1158–1158. ISSN: 1095-9203. DOI: 10.1126/science.263.5150.1158. URL: http://dx.doi.org/10.1126/science.263.5150.1158.

[35] Masamichi Fujihira and Hirosuke Kawate. “Structural study of Langmuir–Blodgett films by scanning surface potential microscopy”. In: *Journal of Vacuum Science amp; Technology B: Microelectronics and Nanometer Structures Processing, Measurement, and Phenomena* 12.3 (1994), 1604–1608. ISSN: 1520-8567. DOI: 10.1116/1.587242. URL: http://dx.doi.org/10.1116/1.587242.

[36] Michio SUGI. “Langmuir-Blodgett films and electronics.” In: *membrane* 11.3 (1986), 137–146. ISSN: 1884-6440. DOI: 10.5360/membrane.11.137. URL: http://dx.doi.org/10.5360/membrane.11.137.

[37] P.S. Vincett and G.G. Roberts. “Electrical and photoelectrical transport properties of Langmuir-Blodgett films and a discussion of possible device applications”. In: *Thin Solid Films* 68.1 (1980), 135–171. ISSN: 0040-6090. DOI: 10.1016/0040-6090(80)90143-1. URL: http://dx.doi.org/10.1016/0040-6090(80)90143-1.

[38] Toyosaka Moriizumi. “Langmuir-Blodgett films as chemical sensors”. In: *Thin Solid Films* 160.1–2 (1988), 413–429. ISSN: 0040-6090. DOI: 10.1016/0040-6090(88)90088-0. URL: http://dx.doi.org/10.1016/0040-6090(88)90088-0.

[39] Gabriele Giancane and Ludovico Valli. “State of art in porphyrin Langmuir–Blodgett films as chemical sensors”. In: *Advances in Colloid and Interface Science* 171–172 (2012), 17–35. ISSN: 0001-8686. DOI: 10.1016/j.cis.2012.01.001. URL: http://dx.doi.org/10.1016/j.cis.2012.01.001.

[40] Sanit Malik and Chandra Charu Tripathi. “Thin Film Deposition by Langmuir Blodgett Technique for Gas Sensing Applications”. In: *Journal of Surface Engineered Materials and Advanced Technology* 03.03 (2013), 235–241. ISSN: 2161-489X. DOI: 10.4236/jsemat.2013.33031. URL: http://dx.doi.org/10.4236/jsemat.2013.33031.

[41] R. H. Tredgold. *Order in Thin Organic Films*. Cambridge University Press, 1994. ISBN: 9780511524349. DOI: 10.1017/cbo9780511524349. URL: http://dx.doi.org/10.1017/cbo9780511524349.

[42] D. Blaudez et al. “Characterization of Langmuir-Blodgett monolayers using polarization modulated FTIR spectroscopy”. In: *Thin Solid Films* 210–211 (1992), 648–651. ISSN: 0040-6090. DOI: 10.1016/0040-6090(92)90364-h. URL: http://dx.doi.org/10.1016/0040-6090(92)90364-h.

[43] G.G Roberts, P.S Vincett, and W.A Barlow. "Technological applications of Langmuir-Blodgett films". In: *Physics in Technology* 12.2 (1981), 69–75. ISSN: 0305-4624. DOI: 10.1088/0305-4624/12/2/i02. URL: http://dx.doi.org/10.1088/0305-4624/12/2/i02.

[44] Epameinondas Leontidis. *Langmuir–Blodgett Films: Sensor and Biomedical Applications and Comparisons with the Layer-by-Layer Method.* 2016. DOI: 10.1002/9783527698813.ch5. URL: http://dx.doi.org/10.1002/9783527698813.ch5.

[45] Katsuhiko Ariga. "Chemistry of Materials Nanoarchitectonics for Two-Dimensional Films: Langmuir–Blodgett, Layer-by-Layer Assembly, and Newcomers". In: *Chemistry of Materials* 35.14 (2023), 5233–5254. ISSN: 1520-5002. DOI: 10.1021/acs.chemmater.3c01291. URL: http://dx.doi.org/10.1021/acs.chemmater.3c01291.

[46] Brian A. Pethica. "Experimental criteria for monolayer studies in relation to the formation of Langmuir-Blodgett multilayers". In: *Thin Solid Films* 152.1–2 (1987), 3–8. ISSN: 0040-6090. DOI: 10.1016/0040-6090(87)90407-x. URL: http://dx.doi.org/10.1016/0040-6090(87)90407-x.

[47] Cristina Stefaniu, Gerald Brezesinski, and Helmuth Möhwald. "Langmuir monolayers as models to study processes at membrane surfaces". In: *Advances in Colloid and Interface Science* 208 (2014), 197–213. ISSN: 0001-8686. DOI: 10.1016/j.cis.2014.02.013. URL: http://dx.doi.org/10.1016/j.cis.2014.02.013.

[48] Cristina Stefaniu, Gerald Brezesinski, and Helmuth Möhwald. "Langmuir monolayers as models to study processes at membrane surfaces". In: *Advances in Colloid and Interface Science* 208 (2014), 197–213. ISSN: 0001-8686. DOI: 10.1016/j.cis.2014.02.013. URL: http://dx.doi.org/10.1016/j.cis.2014.02.013.

[49] Monika Rojewska et al. "Langmuir Monolayer Techniques for the Investigation of Model Bacterial Membranes and Antibiotic Biodegradation Mechanisms". In: *Membranes* 11.9 (2021), p. 707. ISSN: 2077-0375. DOI: 10.3390/membranes11090707. URL: http://dx.doi.org/10.3390/membranes11090707.

[50] Kenjiro Miyano and Toshiteru Maeda. “Langmuir trough with four movable barriers”. In: *Review of Scientific Instruments* 58.3 (1987), 428–435. ISSN: 1089-7623. DOI: 10.1063/1.1139249. URL: http://dx.doi.org/10.1063/1.1139249.

[51] Matthew J. Large et al. “Understanding Solvent Spreading for Langmuir Deposition of Nanomaterial Films: A Hansen Solubility Parameter Approach”. In: *Langmuir* 33.51 (2017), 14766–14771. ISSN: 1520-5827. DOI: 10.1021/acs.langmuir.7b03867. URL: http://dx.doi.org/10.1021/acs.langmuir.7b03867.

[52] James P. Bareman and Michael L. Klein. “Molecular Dynamics Simulation of A Langmuir Monolayer”. In: *MRS Proceedings* 237 (1991). ISSN: 1946-4274. DOI: 10.1557/proc-237-271. URL: http://dx.doi.org/10.1557/proc-237-271.

[53] Paul C. Hiemenz. “The role of van der Waals forces in surface and colloid chemistry”. In: *Journal of Chemical Education* 49.3 (1972), p. 164. ISSN: 1938-1328. DOI: 10.1021/ed049p164. URL: http://dx.doi.org/10.1021/ed049p164.

[54] K. S. Birdi, F. Madsen, and K. Eberth. “Determination of van der Waals forces in monolayer films of lipids amp; biopolymers. Equation of state for two-dimensional films”. In: *Colloid amp; Polymer Science* 272.8 (1994), 1000–1004. ISSN: 1435-1536. DOI: 10.1007/bf00658899. URL: http://dx.doi.org/10.1007/bf00658899.

[55] Joseph T. Buontempo and Frank A. Novak. “An inexpensive Wilhelmy balance for the study of Langmuir monolayers”. In: *Review of Scientific Instruments* 63.12 (1992), 5707–5713. ISSN: 1089-7623. DOI: 10.1063/1.1143353. URL: http://dx.doi.org/10.1063/1.1143353.

[56] Agnes Pockels. “The Measurement of Surface Tension with the Balance”. In: *Science* 64.1656 (1926), 304–304. ISSN: 1095-9203. DOI: 10.1126/science.64.1656.304.a. URL: http://dx.doi.org/10.1126/science.64.1656.304.a.

[57] William D. Harkins, T. Fraser Young, and Edward Boyd. “The Thermodynamics of Films: Energy and Entropy of Extension and Spreading of Insoluble Monolayers”. In: *The Journal of Chemical Physics* 8.12 (1940), 954–965. ISSN: 1089-7690. DOI: 10.1063/1.1750610. URL: http://dx.doi.org/10.1063/1.1750610.

[58] C M Knobler and R C Desai. “Phase Transitions in Monolayers”. In: *Annual Review of Physical Chemistry* 43.1 (1992), 207–236. ISSN: 1545-1593. DOI: 10.1146/annurev.pc.43.100192.001231. URL: http://dx.doi.org/10.1146/annurev.pc.43.100192.001231.

[59] Vladimir M. Kaganer, Helmuth Möhwald, and Pulak Dutta. “Structure and phase transitions in Langmuir monolayers”. In: *Reviews of Modern Physics* 71.3 (1999), 779–819. ISSN: 1539-0756. DOI: 10.1103/revmodphys.71.779. URL: http://dx.doi.org/10.1103/revmodphys.71.779.

[60] D. G. Dervichian. “Changes of Phase and Transformations of Higher Order in Monolayers”. In: *The Journal of Chemical Physics* 7.10 (1939), 931–948. ISSN: 1089-7690. DOI: 10.1063/1.1750347. URL: http://dx.doi.org/10.1063/1.1750347.

[61] Brian G. Moore et al. “Phase diagram of Langmuir monolayers of pentadecanoic acid: quantitative comparison of surface pressure and fluorescence microscopy results”. In: *The Journal of Physical Chemistry* 94.11 (1990), 4588–4595. ISSN: 1541-5740. DOI: 10.1021/j100374a042. URL: http://dx.doi.org/10.1021/j100374a042.

[62] M. Lösche, E. Sackmann, and H. Möhwald. “A Fluorescence Microscopic Study Concerning the Phase Diagram of Phospholipids”. In: *Berichte der Bunsengesellschaft für physikalische Chemie* 87.10 (1983), 848–852. ISSN: 0005-9021. DOI: 10.1002/bbpc.19830871004. URL: http://dx.doi.org/10.1002/bbpc.19830871004.

[63] Ka Yee C. Lee. “Collapse Mechanisms of Langmuir Monolayers”. In: *Annual Review of Physical Chemistry* 59.1 (2008), 771–791. ISSN: 1545-1593. DOI: 10.1146/annurev.physchem.58.032806.104619. URL: http://dx.doi.org/10.1146/annurev.physchem.58.032806.104619.

[64] Jose Luis Fidalgo Rodriguez et al. “Phase transition beyond the monolayer collapse – The case of stearic acid spread at the air/water interface”. In: *Colloids and Surfaces A: Physicochemical and Engineering Aspects* 623 (2021), p. 126781. ISSN: 0927-7757. DOI: 10.1016/j.colsurfa.2021.126781. URL: http://dx.doi.org/10.1016/j.colsurfa.2021.126781.

[65] William D. Harkins and L. E. Copeland. "A Superliquid in Two Dimensions and a First-Order Change in a Condensed Monolayer I. Energy, Compressibility, and Order of Phase Transformations". In: *The Journal of Chemical Physics* 10.5 (1942), 272–286. ISSN: 1089-7690. DOI: 10.1063/1.1723720. URL: http://dx.doi.org/10.1063/1.1723720.

[66] R. M. Kenn et al. "Mesophases and crystalline phases in fatty acid monolayers". In: *The Journal of Physical Chemistry* 95.5 (1991), 2092–2097. ISSN: 1541-5740. DOI: 10.1021/j100158a034. URL: http://dx.doi.org/10.1021/j100158a034.

[67] R. M. Kenn et al. "Mesophases and crystalline phases in fatty acid monolayers". In: *The Journal of Physical Chemistry* 95.5 (1991), 2092–2097. ISSN: 1541-5740. DOI: 10.1021/j100158a034. URL: http://dx.doi.org/10.1021/j100158a034.

[68] Jeremy Schofield and Stuart A. Rice. "Backbone ordering in amphiphile monolayers". In: *The Journal of Chemical Physics* 103.13 (1995), 5792–5801. ISSN: 1089-7690. DOI: 10.1063/1.470460. URL: http://dx.doi.org/10.1063/1.470460.

[69] Salvador Ramos and Rolando Castillo. "Langmuir monolayers of C17,C19, and C21 fatty acids: Textures, phase transitions, and localized oscillations". In: *The Journal of Chemical Physics* 110.14 (1999), 7021–7030. ISSN: 1089-7690. DOI: 10.1063/1.478607. URL: http://dx.doi.org/10.1063/1.478607.

[70] Ellis Teer et al. "Optical measurements of the phase diagrams of Langmuir monolayers of fatty acid, ester, and alcohol mixtures by Brewster-angle microscopy". In: *The Journal of Chemical Physics* 106.5 (1997), 1913–1920. ISSN: 1089-7690. DOI: 10.1063/1.473312. URL: http://dx.doi.org/10.1063/1.473312.

[71] Sharon Grayer Wolf et al. "A Synchrotron X-ray Study of a Solid-Solid Phase Transition in a Two-Dimensional Crystal". In: *Science* 242.4883 (1988), 1286–1290. ISSN: 1095-9203. DOI: 10.1126/science.242.4883.1286. URL: http://dx.doi.org/10.1126/science.242.4883.1286.

[72] Chen Fang et al. "Recent Applications of Langmuir–Blodgett Technique in Battery Research". In: *ACS Applied Materials amp; Interfaces* 14.2 (2022), 2431–2439. ISSN: 1944-8252. DOI: 10.1021/acsami.1c19064. URL: http://dx.doi.org/10.1021/acsami.1c19064.

[73] Katharine B. Blodgett. "Films Built by Depositing Successive Monomolecular Layers on a Solid Surface". In: *Journal of the American Chemical Society* 57.6 (1935), 1007–1022. ISSN: 1520-5126. DOI: 10.1021/ja01309a011. URL: http://dx.doi.org/10.1021/ja01309a011.

[74] Geoffrey J. Ashwell, Paul D. Jackson, and Wendy A. Crossland. "Non-centrosymmetry and second-harmonic generation in Z-type Langmuir–Blodgett films". In: *Nature* 368.6470 (1994), 438–440. ISSN: 1476-4687. DOI: 10.1038/368438a0. URL: http://dx.doi.org/10.1038/368438a0.

[75] Geoffrey J. Ashwell, Paul D. Jackson, and Wendy A. Crossland. "Non-centrosymmetry and second-harmonic generation in Z-type Langmuir–Blodgett films". In: *Nature* 368.6470 (1994), 438–440. ISSN: 1476-4687. DOI: 10.1038/368438a0. URL: http://dx.doi.org/10.1038/368438a0.

[76] J. Alexander Liddle, Yi Cui, and Paul Alivisatos. "Lithographically directed self-assembly of nanostructures". In: *Journal of Vacuum Science amp; Technology B: Microelectronics and Nanometer Structures Processing, Measurement, and Phenomena* 22.6 (2004), 3409–3414. ISSN: 1520-8567. DOI: 10.1116/1.1821572. URL: http://dx.doi.org/10.1116/1.1821572.

[77] Claude Lévy-Clément, Xiaodong Wang, and Patrick Pittet. "Silicon Nanowire Arrays Combining Nanosphere Lithography and Metal-Assisted Etching". In: *ECS Transactions* 33.16 (2011), 15–22. ISSN: 1938-6737. DOI: 10.1149/1.3553151. URL: http://dx.doi.org/10.1149/1.3553151.

[78] James Kurniawan et al. "Preparation and Characterization of Solid-Supported Lipid Bilayers Formed by Langmuir–Blodgett Deposition: A Tutorial". In: *Langmuir* 34.51 (2018), 15622–15639. ISSN: 1520-5827. DOI: 10.1021/acs.langmuir.8b03504. URL: http://dx.doi.org/10.1021/acs.langmuir.8b03504.

[79] Ming Li et al. "AFM Studies of Solid-Supported Lipid Bilayers Formed at a Au(111) Electrode Surface Using Vesicle Fusion and a Combination of Langmuirâˆ'Blodgett and Langmuirâˆ'Schaefer Techniques".

In: *Langmuir* 24.18 (2008), 10313â€“10323. ISSN: 1520-5827. DOI: 10.1021/la800800m. URL: http://dx.doi.org/10.1021/la800800m.

[80] Nobuhiro GEMMA and Makoto AZUMA. “Analyses on the transfer process of LB films.” In: *Hyomen Kagaku* 11.4 (1990), 229–234. ISSN: 1881-4743. DOI: 10.1380/jsssj.11.229. URL: http://dx.doi.org/10.1380/jsssj.11.229.

[81] Dongmok Whang et al. “Large-Scale Hierarchical Organization of Nanowire Arrays for Integrated Nanosystems”. In: *Nano Letters* 3.9 (2003), 1255–1259. ISSN: 1530-6992. DOI: 10.1021/nl0345062. URL: http://dx.doi.org/10.1021/nl0345062.

[82] D. Blaudez et al. “Organization in pure and alternate deuterated cadmium arachidate monolayers on solid substrates and at the air/water interface studied by conventional and differential Fourier transform infrared spectroscopies”. In: *The Journal of Chemical Physics* 104.24 (1996), 9983–9993. ISSN: 1089-7690. DOI: 10.1063/1.471726. URL: http://dx.doi.org/10.1063/1.471726.

[83] M. C. Shih et al. “Structures of fatty acid monolayers transferred to glass substrates from various Langmuir monolayer phases”. In: *Langmuir* 9.3 (1993), 776–778. ISSN: 1520-5827. DOI: 10.1021/la00027a028. URL: http://dx.doi.org/10.1021/la00027a028.

[84] Izabela Brzozowska and Zbigniew A. Figaszewski. “The influence of pH on phosphatidylcholine monolayer at the air/aqueous solution interface”. In: *Colloids and Surfaces B: Biointerfaces* 27.4 (2003), 303–309. ISSN: 0927-7765. DOI: 10.1016/s0927-7765(02)00095-4. URL: http://dx.doi.org/10.1016/s0927-7765(02)00095-4.

[85] F. Peñacorada et al. “Monolayers and Multilayers of Uranyl Arachidate 2. Influence of the Subphase pH on the Structure and Stability of LangmuirBlodgett Films”. In: *Langmuir* 12.5 (1996), 1351–1356. ISSN: 1520-5827. DOI: 10.1021/la950709x. URL: http://dx.doi.org/10.1021/la950709x.

[86] M. C. Shih et al. “Pressure and pH dependence of the structure of a fatty acid monolayer with calcium ions in the subphase”. In: *The Journal of Chemical Physics* 96.2 (1992), 1556–1559. ISSN: 1089-7690. DOI: 10.1063/1.462139. URL: http://dx.doi.org/10.1063/1.462139.

[87] Barbara Krajewska, Paweł Wydro, and Agnieszka Kyzioł. “Chitosan as a subphase disturbant of membrane lipid monolayers. The effect of temperature at varying pH: I. DPPG”. In: *Colloids and Surfaces A: Physicochemical and Engineering Aspects* 434 (2013), 349–358. ISSN: 0927-7757. DOI: 10.1016/j.colsurfa.2013.03.015. URL: http://dx.doi.org/10.1016/j.colsurfa.2013.03.015.

[88] S. Bettarini et al. “Interactions between monolayers and metal ions at the water-air interface: conditions for the transferability as Langmuir-Blodgett multilayers”. In: *Thin Solid Films* 210–211 (1992), 42–45. ISSN: 0040-6090. DOI: 10.1016/0040-6090(92)90163-6. URL: http://dx.doi.org/10.1016/0040-6090(92)90163-6.

[89] Mehran Yazdanian et al. “Divalent cation-stearic acid monolayer interactions at the air/water interface”. In: *Langmuir* 8.2 (1992), 630–636. ISSN: 1520-5827. DOI: 10.1021/la00038a055. URL: http://dx.doi.org/10.1021/la00038a055.

[90] Adrien Sthoer et al. “La3+ and Y3+ interactions with the carboxylic acid moiety at the liquid/vapour interface: identification of binding complexes, charge reversal, and detection limits.” In: (2021). DOI: 10.26434/chemrxiv-2021-28p74. URL: http://dx.doi.org/10.26434/chemrxiv-2021-28p74.

[91] Paula Toimil et al. “A comparative study of F-DPPC/DPPC mixed monolayers. Influence of subphase temperature on F-DPPC and DPPC monolayers”. In: *Physical Chemistry Chemical Physics* 12.40 (2010), p. 13323. ISSN: 1463-9084. DOI: 10.1039/c0cp00506a. URL: http://dx.doi.org/10.1039/c0cp00506a.

[92] M M Qassime et al. “A studying of subphase temperature and dissolved ascorbic acid concentration influence on the process of Langmuir monolayer formation”. In: *Journal of Physics: Conference Series* 1124 (2018), p. 031010. ISSN: 1742-6596. DOI: 10.1088/1742-6596/1124/3/031010. URL: http://dx.doi.org/10.1088/1742-6596/1124/3/031010.

[93] E.S. Gadelmawla et al. “Roughness parameters”. In: *Journal of Materials Processing Technology* 123.1 (2002), 133–145. ISSN: 0924-0136. DOI: 10.1016/s0924-0136(02)00060-2. URL: http://dx.doi.org/10.1016/s0924-0136(02)00060-2.

[94] Irving. Langmuir. "Surface Chemistry." In: *Chemical Reviews* 13.2 (1933), 147–191. ISSN: 1520-6890. DOI: 10.1021/cr60045a001. URL: http://dx.doi.org/10.1021/cr60045a001.

[95] W. D. HARKINS. "SURFACE CHEMISTRY". In: *Nature* 148.3764 (1941), 743–746. ISSN: 1476-4687. DOI: 10.1038/148743a0. URL: http://dx.doi.org/10.1038/148743a0.

[96] M. J. Grundy et al. "Effect of dipping rate on alternating layer Langmuir-Blodgett film structure". In: *Langmuir* 6.2 (1990), 519–521. ISSN: 1520-5827. DOI: 10.1021/la00092a040. URL: http://dx.doi.org/10.1021/la00092a040.

[97] IR Peterson. "Defect density in a metal-monolayer-metal cell". In: *Australian Journal of Chemistry* 33.8 (1980), p. 1713. ISSN: 0004-9425. DOI: 10.1071/ch9801713. URL: http://dx.doi.org/10.1071/ch9801713.

[98] J.G Petrov, H Kuhn, and D Möbius. "Three-phase contact line motion in the deposition of spread monolayers". In: *Journal of Colloid and Interface Science* 73.1 (1980), 66–75. ISSN: 0021-9797. DOI: 10.1016/0021-9797(80)90123-x. URL: http://dx.doi.org/10.1016/0021-9797(80)90123-x.

[99] Herman E. Ries and Hewson Swift. "Twisted double-layer ribbons and the mechanism for monolayer collapse". In: *Langmuir* 3.5 (1987), 853–855. ISSN: 1520-5827. DOI: 10.1021/la00077a048. URL: http://dx.doi.org/10.1021/la00077a048.

[100] Kaushik Das and Sarathi Kundu. "Subphase pH induced monolayer to multilayer collapse of fatty acid Salt Langmuir monolayer at lower surface pressure". In: *Colloids and Surfaces A: Physicochemical and Engineering Aspects* 492 (2016), 54–61. ISSN: 0927-7757. DOI: 10.1016/j.colsurfa.2015.12.016. URL: http://dx.doi.org/10.1016/j.colsurfa.2015.12.016.

[101] O Albrecht et al. "Control of the homogeneity of Langmuir-Blodgett films using the deposition-induced flow in the monolayer". In: *Thin Solid Films* 221.1–2 (1992), 276–280. ISSN: 0040-6090. DOI: 10.1016/0040-6090(92)90827-x. URL: http://dx.doi.org/10.1016/0040-6090(92)90827-x.

[102] Kedar Joshi and James F. Gilchrist. “Effect of added surfactant on convective assembly of monosized microspheres”. In: *Applied Physics Letters* 116.8 (2020). ISSN: 1077-3118. DOI: 10.1063/1.5139962. URL: http://dx.doi.org/10.1063/1.5139962.

[103] Pierre Lesieur, André Barraud, and Michel Vandevyver. “Defect characterization and detection in Langmuir-Blodgett films”. In: *Thin Solid Films* 152.1–2 (1987), 155–164. ISSN: 0040-6090. DOI: 10.1016/0040-6090(87)90414-7. URL: http://dx.doi.org/10.1016/0040-6090(87)90414-7.

[104] H. Fuchs et al. “Defect structures of Langmuir-Blodgett films investigated by scanning force microscopy”. In: *Thin Solid Films* 210–211 (1992), 655–658. ISSN: 0040-6090. DOI: 10.1016/0040-6090(92)90366-j. URL: http://dx.doi.org/10.1016/0040-6090(92)90366-j.

[105] H. G. Hansma et al. “Imaging nanometer scale defects in Langmuir-Blodgett films with the atomic force microscope”. In: *Langmuir* 7.6 (1991), 1051–1054. ISSN: 1520-5827. DOI: 10.1021/la00054a005. URL: http://dx.doi.org/10.1021/la00054a005.

[106] M. Pomerantz. “Characterization of the structure of Langmuir-Blodgett films by short-wavelength radiations”. In: *Thin Solid Films* 152.1–2 (1987), 165–179. ISSN: 0040-6090. DOI: 10.1016/0040-6090(87)90415-9. URL: http://dx.doi.org/10.1016/0040-6090(87)90415-9.

[107] W. W. Zhao and F. J. Boerio. “Characterization of polyimide Langmuir-Blodgett films on silver using infrared, Raman and x-ray photoelectron spectroscopies”. In: *Surface and Interface Analysis* 26.4 (1998), 316–328. ISSN: 1096-9918. DOI: 10.1002/(sici)1096-9918(199804)26:4<316::aid-sia370>3.0.co;2-5. URL: http://dx.doi.org/10.1002/(SICI)1096-9918(199804)26:4<316::AID-SIA370>3.0.CO;2-5.

[108] Junzo. Umemura et al. “Quantitative evaluation of molecular orientation in thin Langmuir-Blodgett films by FT-IR transmission and reflection-absorption spectroscopy”. In: *The Journal of Physical Chemistry* 94.1 (1990), 62–67. ISSN: 1541-5740. DOI: 10.1021/j100364a009. URL: http://dx.doi.org/10.1021/j100364a009.

[109] Junzo. Umemura et al. “Quantitative evaluation of molecular orientation in thin Langmuir-Blodgett films by FT-IR transmission and reflection-absorption spectroscopy”. In: *The Journal of Physical Chemistry* 94.1 (1990), 62–67. ISSN: 1541-5740. DOI: 10.1021/j100364a009. URL: http://dx.doi.org/10.1021/j100364a009.

[110] Elisabetta Maccioni et al. “X-ray diffraction structural analysis of Langmuir-Blodgett films using a pattern recognition approach”. In: *Thin Solid Films* 265.1–2 (1995), 74–83. ISSN: 0040-6090. DOI: 10.1016/0040-6090(95)06620-9. URL: http://dx.doi.org/10.1016/0040-6090(95)06620-9.

[111] Yu. M. Lvov, V. I. Troitsky, and L. A. Feigin. “Structure Analysis of Langmuir-Blodgett Films with Alternating Bilayers by Means of Small-Angle X-ray Scattering and Electron Diffraction”. In: *Molecular Crystals and Liquid Crystals Incorporating Nonlinear Optics* 172.1 (1989), 89–97. ISSN: 1044-1859. DOI: 10.1080/00268948908042154. URL: http://dx.doi.org/10.1080/00268948908042154.

[112] J.K. Basu and M.K. Sanyal. “Ordering and growth of Langmuir–Blodgett films: X-ray scattering studies”. In: *Physics Reports* 363.1 (2002), 1–84. ISSN: 0370-1573. DOI: 10.1016/s0370-1573(01)00083-7. URL: http://dx.doi.org/10.1016/s0370-1573(01)00083-7.

[113] E. Meyer et al. “Molecular-resolution images of Langmuir–Blodgett films using atomic force microscopy”. In: *Nature* 349.6308 (1991), 398–400. ISSN: 1476-4687. DOI: 10.1038/349398a0. URL: http://dx.doi.org/10.1038/349398a0.

[114] E. Meyer et al. “Molecular-resolution images of Langmuir–Blodgett films using atomic force microscopy”. In: *Nature* 349.6308 (1991), 398–400. ISSN: 1476-4687. DOI: 10.1038/349398a0. URL: http://dx.doi.org/10.1038/349398a0.

[115] Barbara M. Goettgens et al. “Molecular order in polymerizable Langmuir-Blodgett films probed by microfluorescence and scanning force microscopy”. In: *Langmuir* 8.7 (1992), 1768–1774. ISSN: 1520-5827. DOI: 10.1021/la00043a014. URL: http://dx.doi.org/10.1021/la00043a014.

[116] Th. Geue et al. “Investigations of pH-dependent domain structure of lead arachidate Langmuir-Blodgett films by means of x-ray specular and diffuse scattering and atomic force microscopy”. In: *The Journal of Chemical Physics* 110.16 (1999), 8104–8111. ISSN: 1089-7690. DOI: 10.1063/1.478713. URL: http://dx.doi.org/10.1063/1.478713.

[117] Jack Y. Josefowicz et al. “Structure of Langmuir-Blodgett Films of Disk-Shaped Molecules Determined by Atomic Force Microscopy”. In: *Science* 260.5106 (1993), 323–326. ISSN: 1095-9203. DOI: 10.1126/science.260.5106.323. URL: http://dx.doi.org/10.1126/science.260.5106.323.

[118] L. Wolthaus et al. “Structural investigation of dipping lines in Langmuir-Blodgett films by scanning force microscopy”. In: *Thin Solid Films* 242.1–2 (1994), 170–173. ISSN: 0040-6090. DOI: 10.1016/0040-6090(94)90523-1. URL: http://dx.doi.org/10.1016/0040-6090(94)90523-1.

[119] Fumiko Kimura, Junzo Umemura, and Tohru Takenaka. “FTIR-ATR studies on Langmuir-Blodgett films of stearic acid with 1-9 monolayers”. In: *Langmuir* 2.1 (1986), pp. 96–101. ISSN: 0743-7463. DOI: 10.1021/la00067a017. URL: https://doi.org/10.1021/la00067a017.

[120] Fumiko Kimura, Junzo Umemura, and Tohru Takenaka. “FTIR-ATR studies on Langmuir-Blodgett films of stearic acid with 1-9 monolayers”. In: *Langmuir* 2.1 (1986), 96–101. ISSN: 1520-5827. DOI: 10.1021/la00067a017. URL: http://dx.doi.org/10.1021/la00067a017.

[121] Katsunori ONO et al. “3028 Study on surface energy of ultrathin film: Evaluation by using stearic acid LB film on glass substrate”. In: *The proceedings of the JSME annual meeting* 2007.7.0 (2007), 251–252. ISSN: 2433-1325. DOI: 10.1299/jsmemecjo.2007.7.0_251. URL: http://dx.doi.org/10.1299/jsmemecjo.2007.7.0_251.

[122] Phillip Sawunyama et al. “Photodecomposition of a LangmuiràíBlodgett Film of Stearic Acid on TiO2 Film Observed by in Situ Atomic Force Microscopy and FT-IR”. In: *The Journal of Physical Chemistry B* 101.51 (1997), 11000Àì11003. ISSN: 1520-5207. DOI: 10.1021/jp9730095. URL: http://dx.doi.org/10.1021/jp9730095.

[123] Takeshi Hasegawa, Junzo Umemura, and Tohru Takenaka. "Fourier transform infrared metal overlayer attenuated total reflection spectra of Langmuir-Blodgett films of 12-hydroxystearic acid and its cadmium salt". In: *Thin Solid Films* 210–211 (1992), 583–585. ISSN: 0040-6090. DOI: 10.1016/0040-6090(92)90347-e. URL: http://dx.doi.org/10.1016/0040-6090(92)90347-e.

[124] Richard A. Dluhy, Norman A. Wright, and Peter R. Griffiths. "In Situ Measurement of the FT-IR Spectra of Phospholipid Monolayers at the Air/Water Interface". In: *Applied Spectroscopy* 42.1 (1988), 138–141. ISSN: 1943-3530. DOI: 10.1366/0003702884428680. URL: http://dx.doi.org/10.1366/0003702884428680.

[125] Yasushi Koyama et al. "The relation between the crystal axes and the dipping direction of built-up films of octadecanoic acid as revealed by polarized infrared transmission spectroscopy". In: *Journal of Colloid and Interface Science* 61.3 (1977), 438–445. ISSN: 0021-9797. DOI: 10.1016/0021-9797(77)90462-3. URL: http://dx.doi.org/10.1016/0021-9797(77)90462-3.

[126] Pierre-Alain Chollet, Jean Messier, and Charles Rosilio. "Infrared determination of the orientation of molecules in stearamide monolayers". In: *The Journal of Chemical Physics* 64.3 (1976), 1042–1050. ISSN: 1089-7690. DOI: 10.1063/1.432313. URL: http://dx.doi.org/10.1063/1.432313.

[127] H. Sakai and J. Umemura. "Molecular Orientation in Langmuir Films of 12-Hydroxystearic Acid Studied by Infrared External-Reflection Spectroscopy". In: *Langmuir* 14.21 (1998), 6249–6255. ISSN: 1520-5827. DOI: 10.1021/la971016e. URL: http://dx.doi.org/10.1021/la971016e.

[128] F.J Boerio and S.L Chen. "Infrared spectra of adsorbed films on metal mirrors". In: *Journal of Colloid and Interface Science* 73.1 (1980), 176–185. ISSN: 0021-9797. DOI: 10.1016/0021-9797(80)90134-4. URL: http://dx.doi.org/10.1016/0021-9797(80)90134-4.

[129] J.F. Rabolt et al. "Molecular Orientation in thin Monolayer Films by Infrared Spectroscopy". In: *Vibrations at Surfaces, Proceedings of the Third International Conference*. Elsevier, 1983, 29–34. DOI: 10.1016/s0167-2991(09)61093-4. URL: http://dx.doi.org/10.1016/s0167-2991(09)61093-4.

[130] J. F. Rabolt et al. "Anisotropic orientation in molecular monolayers by infrared spectroscopy". In: *The Journal of Chemical Physics* 78.2 (1983), 946–952. ISSN: 1089-7690. DOI: 10.1063/1.444799. URL: http://dx.doi.org/10.1063/1.444799.

[131] Tatsuo Higashiyama and Tohru Takenaka. "Infrared attenuated total reflection spectra of adsorbed layers at the interface between a germanium electrode and an aqueous solution of sodium laurate". In: *The Journal of Physical Chemistry* 78.9 (1974), 941–947. ISSN: 1541-5740. DOI: 10.1021/j100602a016. URL: http://dx.doi.org/10.1021/j100602a016.

[132] Tohru Takenaka et al. "Studies on built-up films by means of the polarized infrared ATR spectrum I." In: *Journal of Colloid and Interface Science* 35.3 (1971), 395–402. ISSN: 0021-9797. DOI: 10.1016/0021-9797(71)90138-x. URL: http://dx.doi.org/10.1016/0021-9797(71)90138-x.

[133] Junzo Umemura et al. "Orientation evaluation of polyion complex Langmuir-Blodgett films by Fourier transform IR transmission and reflection-absorption spectroscopy". In: *Thin Solid Films* 178.1–2 (1989), 281–287. ISSN: 0040-6090. DOI: 10.1016/0040-6090(89)90313-1. URL: http://dx.doi.org/10.1016/0040-6090(89)90313-1.

[134] Alexander Michailov, Alexey Povolotskiy, and Vladimir Kuzmin. "Four-parameter model of thin surface layer contribution to reflectance-absorbance spectroscopy and ellipsometry". In: *Optics Express* 32.4 (2024), p. 6619. ISSN: 1094-4087. DOI: 10.1364/oe.514646. URL: http://dx.doi.org/10.1364/oe.514646.

[135] Takeshi Kawai et al. "FT-IR External Reflection Spectroscopy Study on Photochromic Monolayers at the Air-Water Interface". In: *Chemistry Letters* 28.5 (1999), 375–376. ISSN: 1348-0715. DOI: 10.1246/cl.1999.375. URL: http://dx.doi.org/10.1246/cl.1999.375.

[136] Luciano Caseli et al. "Investigation of the Conformational Changes of a Conducting Polymer in Gas Sensor Active Layers by Means of Polarization-Modulation Infrared Reflection Absorption Spectroscopy (PM-IRRAS)." In: *Langmuir* 29.8 (2013), 2640–2645. ISSN: 1520-5827. DOI: 10.1021/la3050797. URL: http://dx.doi.org/10.1021/la3050797.

[137] Carol R. Flach, Arne Gericke, and Richard Mendelsohn. "Quantitative Determination of Molecular Chain Tilt Angles in Monolayer Films at the Air/Water Interface: Infrared Reflection/Absorption Spectroscopy of Behenic Acid Methyl Ester". In: *The Journal of Physical Chemistry B* 101.1 (1997), 58–65. ISSN: 1520-5207. DOI: 10.1021/jp962288d. URL: http://dx.doi.org/10.1021/jp962288d.

[138] Hin Chu et al. "Surface Enhanced Raman Scattering Substrates Made by Oblique Angle Deposition: Methods and Applications". In: *Coatings* 7.2 (2017), p. 26. ISSN: 2079-6412. DOI: 10.3390/coatings7020026. URL: http://dx.doi.org/10.3390/coatings7020026.

[139] Christy L. Haynes et al. "Surface-enhanced Raman sensors: early history and the development of sensors for quantitative biowarfare agent and glucose detection". In: *Journal of Raman Spectroscopy* 36.6–7 (2005), 471–484. ISSN: 1097-4555. DOI: 10.1002/jrs.1376. URL: http://dx.doi.org/10.1002/jrs.1376.

[140] Somsubhra Saha, Manash Ghosh, and Joydeep Chowdhury. "Infused self-assembly on Langmuir–Blodgett Film: Fabrication of highly efficient SERS active substrates with controlled plasmonic aggregates". In: *Journal of Raman Spectroscopy* 50.3 (2018), 330–344. ISSN: 1097-4555. DOI: 10.1002/jrs.5529. URL: http://dx.doi.org/10.1002/jrs.5529.

[141] Somsubhra Saha et al. "Self-assembly of silver nanocolloids in the Langmuir–Blodgett Film of stearic acid: Evidence of an efficient SERS sensing platform". In: *Journal of Raman Spectroscopy* 47.2 (2015), 168–176. ISSN: 1097-4555. DOI: 10.1002/jrs.4771. URL: http://dx.doi.org/10.1002/jrs.4771.

[142] R. Griffith Freeman et al. "Self-Assembled Metal Colloid Monolayers: An Approach to SERS Substrates". In: *Science* 267.5204 (1995), 1629–1632. ISSN: 1095-9203. DOI: 10.1126/science.267.5204.1629. URL: http://dx.doi.org/10.1126/science.267.5204.1629.

[143] J. Alan Creighton and Desmond G. Eadon. "Ultraviolet–visible absorption spectra of the colloidal metallic elements". In: *J. Chem. Soc., Faraday Trans.* 87.24 (1991), 3881–3891. ISSN: 1364-5455. DOI: 10.1039/ft9918703881. URL: http://dx.doi.org/10.1039/ft9918703881.

[144] Andreas Klamt. "Calculation of UV/Vis Spectra in Solution". In: *The Journal of Physical Chemistry* 100.9 (1996), 3349–3353. ISSN: 1541-5740. DOI: 10.1021/jp950607f. URL: http://dx.doi.org/10.1021/jp950607f.

[145] Zhao Qing-nan et al. "UV-vis and photoluminescent spectra of TiO2 films". In: *Journal of Wuhan University of Technology-Mater. Sci. Ed.* 18.3 (2003), 36–39. ISSN: 1993-0437. DOI: 10.1007/bf02838454. URL: http://dx.doi.org/10.1007/bf02838454.

[146] Maria Rosaria di Nunzio et al. "Interrogating the Behaviour of a Styryl Dye Interacting with a Mesoscopic 2D-MOF and Its Luminescent Vapochromic Sensing". In: *International Journal of Molecular Sciences* 23.1 (2021), p. 330. ISSN: 1422-0067. DOI: 10.3390/ijms23010330. URL: http://dx.doi.org/10.3390/ijms23010330.

[147] Miharu Eguchi et al. "Adsorchromism: Molecular Nanoarchitectonics at 2D Nanosheets—Old Chemistry for Advanced Chromism". In: *Advanced Science* 8.14 (2021). ISSN: 2198-3844. DOI: 10.1002/advs.202100539. URL: http://dx.doi.org/10.1002/advs.202100539.

[148] Suqian Ma et al. "Organic molecular aggregates: From aggregation structure to emission property". In: *Aggregate* 2.4 (2021). ISSN: 2692-4560. DOI: 10.1002/agt2.96. URL: http://dx.doi.org/10.1002/agt2.96.

[149] Xing Feng et al. "Aggregation behaviour of pyrene-based luminescent materials, from molecular design and optical properties to application". In: *Chemical Society Reviews* 52.19 (2023), 6715–6753. ISSN: 1460-4744. DOI: 10.1039/d3cs00251a. URL: http://dx.doi.org/10.1039/d3cs00251a.

[150] Guilherme Nuñez Jaroque, Patrícia Sartorelli, and Luciano Caseli. "Interfacial vibrational spectroscopy and Brewster angle microscopy distinguishing the interaction of terpineol in cell membrane models at the air-water interface". In: *Biophysical Chemistry* 246 (2019), 1–7. ISSN: 0301-4622. DOI: 10.1016/j.bpc.2018.12.003. URL: http://dx.doi.org/10.1016/j.bpc.2018.12.003.

[151] B. E. Warren. "X-Ray Diffraction Methods". In: *Journal of Applied Physics* 12.5 (1941), 375–384. ISSN: 1089-7550. DOI: 10.1063/1.1712915. URL: http://dx.doi.org/10.1063/1.1712915.

[152] Yuji Sasanuma et al. “Characterization of long-periodic layered structures by X-ray diffraction II: Lattice distortion of Langmuir-Blodgett films of lead stearate”. In: *Thin Solid Films* 190.2 (1990), 325–334. ISSN: 0040-6090. DOI: 10.1016/0040-6090(89)90922-x. URL: http://dx.doi.org/10.1016/0040-6090(89)90922-x.

[153] H. J. Merle, Y. M. Lvov, and I. R. Peterson. “X-ray study of LB multilayers of cadmium soaps in the vicinity of the break up point”. In: *Makromolekulare Chemie. Macromolecular Symposia* 46.1 (1991), 271–275. ISSN: 0258-0322. DOI: 10.1002/masy.19910460137. URL: http://dx.doi.org/10.1002/masy.19910460137.

[154] Clifford Holley and Seymour Bernstein. “X-Ray Diffraction by a Film of Counted Molecular Layers”. In: *Physical Review* 49.5 (1936), 403–403. ISSN: 0031-899X. DOI: 10.1103/physrev.49.403.2. URL: http://dx.doi.org/10.1103/physrev.49.403.2.

[155] M. POMERANTZ and ARMIN SEGMÜLLER. “HIGH RESOLUTION X-RAY DIFFRACTION FROM SMALL NUMBERS OF LANGMUIR–BLODGETT LAYERS OF MANGANESE STEARATE”. In: *Langmuir–Blodgett Films*. Elsevier, 1980, 33–45. ISBN: 9780444419019. DOI: 10.1016/b978-0-444-41901-9.50010-0. URL: http://dx.doi.org/10.1016/b978-0-444-41901-9.50010-0.

[156] Tae Joo Shin and Hoichang Yang. “Grazing Incidence X-Ray Diffraction (GIXD)”. In: *Organic Field-Effect Transistors*. CRC Press, 2018, 253–276. ISBN: 9781315221687. DOI: 10.1201/9781420008012-8. URL: http://dx.doi.org/10.1201/9781420008012-8.

[157] C. Boehm et al. “Packing Characteristics of Crystalline Monolayers of Fatty Acid Salts, at the Air-Solution Interface, Studied by Grazing Incidence X-ray Diffraction”. In: *Langmuir* 10.3 (1994), 830–836. ISSN: 1520-5827. DOI: 10.1021/la00015a038. URL: http://dx.doi.org/10.1021/la00015a038.

[158] Kristian Kjaer. “Some simple ideas on X-ray reflection and grazing-incidence diffraction from thin surfactant films”. In: *Physica B: Condensed Matter* 198.1–3 (1994), 100–109. ISSN: 0921-4526. DOI: 10.1016/0921-4526(94)90137-6. URL: http://dx.doi.org/10.1016/0921-4526(94)90137-6.

[159] Jens Als-Nielsen et al. "Principles and applications of grazing incidence X-ray and neutron scattering from ordered molecular monolayers at the air-water interface". In: *Physics Reports* 246.5 (1994), 251–313. ISSN: 0370-1573. DOI: 10.1016/0370-1573(94)90046-9. URL: http://dx.doi.org/10.1016/0370-1573(94)90046-9.

[160] Bruno Berge, Pierre-François Lenne, and Anne Renault. "X-ray grazing incidence diffraction on monolayers at the surface of water". In: *Current Opinion in Colloid amp; Interface Science* 3.3 (1998), 321–326. ISSN: 1359-0294. DOI: 10.1016/s1359-0294(98)80079-4. URL: http://dx.doi.org/10.1016/s1359-0294(98)80079-4.

[161] Nobutaka Shioya et al. "Multiple-angle incidence resolution spectrometry: applications in nanoarchitectonics and applied physics". In: *Japanese Journal of Applied Physics* 63.6 (2024), p. 060102. ISSN: 1347-4065. DOI: 10.35848/1347-4065/ad4ad8. URL: http://dx.doi.org/10.35848/1347-4065/ad4ad8.

[162] Hikmet Sezen and Sefik Suzer. "XPS for chemical- and charge-sensitive analyses". In: *Thin Solid Films* 534 (2013), 1–11. ISSN: 0040-6090. DOI: 10.1016/j.tsf.2013.02.002. URL: http://dx.doi.org/10.1016/j.tsf.2013.02.002.

[163] Kazue Kurihara et al. "Elemental Analysis of Langmuir-Blodgett Films by X-ray Photoelectron Spectroscopy". In: *Langmuir* 11.4 (1995), 1408–1411. ISSN: 1520-5827. DOI: 10.1021/la00004a057. URL: http://dx.doi.org/10.1021/la00004a057.

[164] Adrian Gainar et al. "NEXAFS Sensitivity to Bond Lengths in Complex Molecular Materials: A Study of Crystalline Saccharides". In: *The Journal of Physical Chemistry B* 119.45 (2015), 14373–14381. ISSN: 1520-5207. DOI: 10.1021/acs.jpcb.5b07159. URL: http://dx.doi.org/10.1021/acs.jpcb.5b07159.

[165] Theyencheri Narayanan and Oleg Konovalov. "Synchrotron Scattering Methods for Nanomaterials and Soft Matter Research". In: *Materials* 13.3 (2020), p. 752. ISSN: 1996-1944. DOI: 10.3390/ma13030752. URL: http://dx.doi.org/10.3390/ma13030752.

[166] J.P. Rabe et al. "Near-edge X-ray absorption fine structure studies of oriented molecular chains in polyethylene and Langmuir-Blodgett monolayers on Si(111)". In: *Thin Solid Films* 159.1–2 (1988), 275–283.

ISSN: 0040-6090. DOI: 10.1016/0040-6090(88)90639-6. URL: http://dx.doi.org/10.1016/0040-6090(88)90639-6.

[167] V. Klechkovskaya et al. "Comparative analysis of high energy electron diffraction patterns from LB films of Cd- and Pb-stearates". In: *Thin Solid Films* 284–285 (1996), 208–210. ISSN: 0040-6090. DOI: 10.1016/s0040-6090(95)08307-3. URL: http://dx.doi.org/10.1016/s0040-6090(95)08307-3.

[168] A Fischer and E Sackmann. "Electron microscopy and electron diffraction study of coexisting phases of pure and mixed monolayers transferred onto solid substrates". In: *Journal of Colloid and Interface Science* 112.1 (1986), 1–14. ISSN: 0021-9797. DOI: 10.1016/0021-9797(86)90064-0. URL: http://dx.doi.org/10.1016/0021-9797(86)90064-0.

[169] Keith J. Stine. *Brewster Angle Microscopy.* 2012. DOI: 10.1002/9780470661345.smc040. URL: http://dx.doi.org/10.1002/9780470661345.smc040.

[170] S. Hénon and J. Meunier. "Microscope at the Brewster angle: Direct observation of first-order phase transitions in monolayers". In: *Review of Scientific Instruments* 62.4 (1991), 936–939. ISSN: 1089-7623. DOI: 10.1063/1.1142032. URL: http://dx.doi.org/10.1063/1.1142032.

[171] Dirk Honig, Gernot A. Overbeck, and Dietmar Mobius. "Morphology of pentadecanoic acid monolayers at the air/water interface studied by BAM". In: *Advanced Materials* 4.6 (1992), 419–424. ISSN: 1521-4095. DOI: 10.1002/adma.19920040610. URL: http://dx.doi.org/10.1002/adma.19920040610.

[172] U. Gehlert, S. Siegel, and D. Vollhardt. "Direct visualization of monolayer structures with a brewster angle microscope". In: *Trends in Colloid and Interface Science VII.* Steinkopff, 247–247. ISBN: 9783798516762. DOI: 10.1007/bfb0118534. URL: http://dx.doi.org/10.1007/bfb0118534.

[173] R Peters and K Beck. "Translational diffusion in phospholipid monolayers measured by fluorescence microphotolysis." In: *Proceedings of the National Academy of Sciences* 80.23 (1983), 7183–7187. ISSN: 1091-6490. DOI: 10.1073/pnas.80.23.7183. URL: http://dx.doi.org/10.1073/pnas.80.23.7183.

[174] Mitsumasa IWAMOTO, Masaaki SUZUKI, and Taro HINO. "Ellipsometoric study on LB films." In: *SHINKU* 28.9 (1985), 693–700. ISSN: 1880-9413. DOI: 10.3131/jvsj.28.693. URL: http://dx.doi.org/10.3131/jvsj.28.693.

[175] H. Knobloch, F. Peñacorada, and L. Brehmer. "Ellipsometric studies on uranyl arachidate Langmuir-Blodgett films". In: *Thin Solid Films* 295.1–2 (1997), 210–213. ISSN: 0040-6090. DOI: 10.1016/s0040-6090(96)09401-1. URL: http://dx.doi.org/10.1016/s0040-6090(96)09401-1.

[176] B. Lecourt, D. Blaudez, and J.M. Turlet. "Anisotropy in Langmuir–Blodgett films studied by generalized spectroscopic ellipsometry". In: *Thin Solid Films* 313–314 (1998), 790–794. ISSN: 0040-6090. DOI: 10.1016/s0040-6090(97)00996-6. URL: http://dx.doi.org/10.1016/s0040-6090(97)00996-6.

[177] B. Drevillon and R. Benferhat. "Infrared ellipsometry study of the vibrational properties and the growth of hydrogenated amorphous silicon ultrathin films". In: *Journal of Applied Physics* 63.10 (1988), 5088–5091. ISSN: 1089-7550. DOI: 10.1063/1.340408. URL: http://dx.doi.org/10.1063/1.340408.

[178] Xiao-Lin Zhou and Sow-Hsin Chen. "Theoretical foundation of X-ray and neutron reflectometry". In: *Physics Reports* 257.4–5 (1995), 223–348. ISSN: 0370-1573. DOI: 10.1016/0370-1573(94)00110-o. URL: http://dx.doi.org/10.1016/0370-1573(94)00110-o.

[179] A. Gibaud, M. S. Chebil, and T. Beuvier. "X-Ray Reflectivity". In: *Surface Science Techniques*. Springer Berlin Heidelberg, 2013, 191–216. ISBN: 9783642342431. DOI: 10.1007/978-3-642-34243-1_7. URL: http://dx.doi.org/10.1007/978-3-642-34243-1_7.

[180] Jielong Su et al. "Smooth deuterated cellulose films for the visualisation of adsorbed bio-macromolecules". In: *Scientific Reports* 6.1 (2016). ISSN: 2045-2322. DOI: 10.1038/srep36119. URL: http://dx.doi.org/10.1038/srep36119.

[181] Richard A. Campbell et al. "Structure of surfactant and phospholipid monolayers at the air/water interface modeled from neutron reflectivity data". In: *Journal of Colloid and Interface Science* 531 (2018), 98–108. ISSN: 0021-9797. DOI: 10.1016/j.jcis.2018.07.022. URL: http://dx.doi.org/10.1016/j.jcis.2018.07.022.

[182] N. Dent et al. “X-ray and neutron scattering from spread monolayers and lb films”. In: *Journal de Chimie Physique* 85 (1988), 1003–1008. ISSN: 0021-7689. DOI: 10.1051/jcp/1988851003. URL: http://dx.doi.org/10.1051/jcp/1988851003.

[183] J Penfold and R K Thomas. “The application of the specular reflection of neutrons to the study of surfaces and interfaces”. In: *Journal of Physics: Condensed Matter* 2.6 (1990), 1369–1412. ISSN: 1361-648X. DOI: 10.1088/0953-8984/2/6/001. URL: http://dx.doi.org/10.1088/0953-8984/2/6/001.

[184] I. R. Girling et al. “Second-harmonic generation in mixed hemicyanine: fatty-acid Langmuir–Blodgett monolayers”. In: *Journal of the Optical Society of America B* 4.6 (1987), p. 950. ISSN: 1520-8540. DOI: 10.1364/josab.4.000950. URL: http://dx.doi.org/10.1364/josab.4.000950.

[185] G. J. Ashwell et al. “Improved second-harmonic generation from Langmuir–Blodgett films of hemicyanine dyes”. In: *Nature* 357.6377 (1992), 393–395. ISSN: 1476-4687. DOI: 10.1038/357393a0. URL: http://dx.doi.org/10.1038/357393a0.

[186] G. J. Ashwell et al. “Improved second-harmonic generation from Langmuir–Blodgett films of hemicyanine dyes”. In: *Nature* 357.6377 (1992), 393–395. ISSN: 1476-4687. DOI: 10.1038/357393a0. URL: http://dx.doi.org/10.1038/357393a0.

[187] Y. R. Shen. “Surface properties probed by second-harmonic and sum-frequency generation”. In: *Nature* 337.6207 (1989), 519–525. ISSN: 1476-4687. DOI: 10.1038/337519a0. URL: http://dx.doi.org/10.1038/337519a0.

[188] Y.R Shen. “Surfaces probed by nonlinear optics”. In: *Surface Science* 299–300 (1994), 551–562. ISSN: 0039-6028. DOI: 10.1016/0039-6028(94)90681-5. URL: http://dx.doi.org/10.1016/0039-6028(94)90681-5.

[189] X. Zhuang et al. “Mapping molecular orientation and conformation at interfaces by surface nonlinear optics”. In: *Physical Review B* 59.19 (1999), 12632–12640. ISSN: 1095-3795. DOI: 10.1103/physrevb.59.12632. URL: http://dx.doi.org/10.1103/physrevb.59.12632.

[190] A. Laschewsky et al. “Amphiphilic dyes for NLO in LB-films”. In: *Makromolekulare Chemie. Macromolecular Symposia* 46.1 (1991), 205–210. ISSN: 0258-0322. DOI: 10.1002/masy.19910460126. URL: http://dx.doi.org/10.1002/masy.19910460126.

[191] F. Kajzar et al. “Optical third-harmonic generation from Langmuir-Blodgett merocyanine dye thin films”. In: *Electronics Letters* 22.23 (1986), 1230–1231. ISSN: 1350-911X. DOI: 10.1049/el:19860843. URL: http://dx.doi.org/10.1049/el:19860843.

[192] X. D. Zhu, Hajo Suhr, and Y. R. Shen. “Surface vibrational spectroscopy by infrared-visible sum frequency generation”. In: *Physical Review B* 35.6 (1987), 3047–3050. ISSN: 0163-1829. DOI: 10.1103/physrevb.35.3047. URL: http://dx.doi.org/10.1103/physrevb.35.3047.

[193] P. Guyot-Sionnest, J. H. Hunt, and Y. R. Shen. “Sum-frequency vibrational spectroscopy of a Langmuir film: Study of molecular orientation of a two-dimensional system”. In: *Physical Review Letters* 59.14 (1987), 1597–1600. ISSN: 0031-9007. DOI: 10.1103/physrevlett.59.1597. URL: http://dx.doi.org/10.1103/physrevlett.59.1597.

[194] Diogo Volpati et al. “Vibrational spectroscopy for probing molecular-level interactions in organic films mimicking biointerfaces”. In: *Advances in Colloid and Interface Science* 207 (2014), 199–215. ISSN: 0001-8686. DOI: 10.1016/j.cis.2014.01.014. URL: http://dx.doi.org/10.1016/j.cis.2014.01.014.

[195] Rebeca da Rocha Rodrigues et al. “Conjugated polymers as Langmuir and Langmuir-Blodgett films: Challenges and applications in nanostructured devices”. In: *Advances in Colloid and Interface Science* 285 (2020), p. 102277. ISSN: 0001-8686. DOI: 10.1016/j.cis.2020.102277. URL: http://dx.doi.org/10.1016/j.cis.2020.102277.

[196] Thiago E. Goto et al. “Langmuir–Blodgett films based on poly(p-phenylene vinylene) and protein-stabilised palladium nanoparticles: Implications in luminescent and conducting properties”. In: *Thin Solid Films* 540 (2013), 202–207. ISSN: 0040-6090. DOI: 10.1016/j.tsf.2013.05.106. URL: http://dx.doi.org/10.1016/j.tsf.2013.05.106.

[197] Guofeng Xu, Zhenan Bao, and John T. Groves. “Langmuir-Blodgett Films of Regioregular Poly(3-hexylthiophene) as Field-Effect Transistors”. In: *Langmuir* 16.4 (2000), 1834Äì1841. ISSN: 1520-5827. DOI: 10.1021/la9904455. URL: http://dx.doi.org/10.1021/la9904455.

[198] Masahiro Rikukawa et al. “Electrical properties of conductive Langmuir-Blodgett films comprised of head-to-tail poly (3-hexylthiophene)”. In: *Thin Solid Films* 284–285 (1996), 636–639. ISSN: 0040-6090. DOI: 10.1016/s0040-6090(95)08409-6. URL: http://dx.doi.org/10.1016/s0040-6090(95)08409-6.

[199] R. Shanley et al. “MIS hydrogen sensors containing LB film insulator layers”. In: *Sensors and Actuators B: Chemical* 2.1 (1990), 57–62. ISSN: 0925-4005. DOI: 10.1016/0925-4005(90)80009-o. URL: http://dx.doi.org/10.1016/0925-4005(90)80009-o.

[200] Tokuji MIYASHITA. “Polymer LB Films.” In: *Kobunshi* 47.3 (1998), 122–125. ISSN: 2185-9825. DOI: 10.1295/kobunshi.47.122. URL: http://dx.doi.org/10.1295/kobunshi.47.122.

[201] Kiyoshi Oguchi et al. “LB Films of Poly(vinyl acetal)”. In: *Polymer Journal* 18.11 (1986), 887–890. ISSN: 1349-0540. DOI: 10.1295/polymj.18.887. URL: http://dx.doi.org/10.1295/polymj.18.887.

[202] Richard L. McCreery and Adam Johan Bergren. “Progress with Molecular Electronic Junctions: Meeting Experimental Challenges in Design and Fabrication”. In: *Advanced Materials* 21.43 (2009), 4303–4322. ISSN: 1521-4095. DOI: 10.1002/adma.200802850. URL: http://dx.doi.org/10.1002/adma.200802850.

[203] C. Joachim, J. K. Gimzewski, and A. Aviram. “Electronics using hybrid-molecular and mono-molecular devices”. In: *Nature* 408.6812 (2000), 541–548. ISSN: 1476-4687. DOI: 10.1038/35046000. URL: http://dx.doi.org/10.1038/35046000.

[204] Ludovico Valli. “Phthalocyanine-based Langmuir–Blodgett films as chemical sensors”. In: *Advances in Colloid and Interface Science* 116.1–3 (2005), 13–44. ISSN: 0001-8686. DOI: 10.1016/j.cis.2005.04.008. URL: http://dx.doi.org/10.1016/j.cis.2005.04.008.

[205] Sukhananazerin Abdulla and Biji Pullithadathil. “Unidirectional Langmuir–Blodgett-Mediated Alignment of Polyaniline-Functionalized Multiwalled Carbon Nanotubes for NH3 Gas Sensor Applications”. In: *Langmuir* 36.39 (2020), 11618–11628. ISSN: 1520-5827. DOI: 10.1021/acs.langmuir.0c02200. URL: http://dx.doi.org/10.1021/acs.langmuir.0c02200.

[206] Cheng Qian et al. “Facile preparation of self-assembled black phosphorus-based composite LB films as new chemical gas sensors”. In: *Colloids and Surfaces A: Physicochemical and Engineering Aspects* 608 (2021), p. 125616. ISSN: 0927-7757. DOI: 10.1016/j.colsurfa.2020.125616. URL: http://dx.doi.org/10.1016/j.colsurfa.2020.125616.

[207] Ilya Gorbachev et al. “Langmuir-Blodgett Films of Arachidic and Stearic Acids as Sensitive Coatings for Chloroform HF SAW Sensors”. In: *Sensors* 23.1 (2022), p. 100. ISSN: 1424-8220. DOI: 10.3390/s23010100. URL: http://dx.doi.org/10.3390/s23010100.

[208] José R. Siqueira et al. “Immobilization of biomolecules on nanostructured films for biosensing”. In: *Biosensors and Bioelectronics* 25.6 (2010), 1254–1263. ISSN: 0956-5663. DOI: 10.1016/j.bios.2009.09.043. URL: http://dx.doi.org/10.1016/j.bios.2009.09.043.

[209] Hirohiko Tsuzuki et al. “A Novel Glucose Sensor with a Glucose Oxidase Monolayer Immobilized by the Langmuir–Blodgett Technique”. In: *Chemistry Letters* 17.8 (1988), 1265–1268. ISSN: 1348-0715. DOI: 10.1246/cl.1988.1265. URL: http://dx.doi.org/10.1246/cl.1988.1265.

[210] Ilya Gorbachev et al. “Langmuir–Blodgett Films with Immobilized Glucose Oxidase Enzyme Molecules for Acoustic Glucose Sensor Application”. In: *Sensors* 23.11 (2023), p. 5290. ISSN: 1424-8220. DOI: 10.3390/s23115290. URL: http://dx.doi.org/10.3390/s23115290.

[211] Ke-Hsuan Wang et al. “Architecture effects of glucose oxidase/Au nanoparticle composite Langmuir-Blodgett films on glucose sensing performance”. In: *Applied Surface Science* 366 (2016), 202–209. ISSN: 0169-4332. DOI: 10.1016/j.apsusc.2016.01.047. URL: http://dx.doi.org/10.1016/j.apsusc.2016.01.047.

[212] L. A. Feigin et al. “Langmuir-Blodgett films of immunoglobulins — perspective material for biosensors”. In: *Trends in Colloid and Interface Science VII*. Steinkopff, 276–276. ISBN: 9783798516762. DOI: 10.1007/bfb0118552. URL: http://dx.doi.org/10.1007/bfb0118552.

[213] V. V. Erokhin et al. “Langmuir-blodgett films of immunoglobulins as sensing elements”. In: *Il Nuovo Cimento D* 12.9 (1990), 1253–1258. ISSN: 0392-6737. DOI: 10.1007/bf02450391. URL: http://dx.doi.org/10.1007/bf02450391.

[214] I.V. Turko, I.S. Yurkevich, and V.L. Chashchin. “Langmuir-Blodgett films of immunoglobulin G for immunosensors”. In: *Thin Solid Films* 205.1 (1991), 113–116. ISSN: 0040-6090. DOI: 10.1016/0040-6090(91)90481-c. URL: http://dx.doi.org/10.1016/0040-6090(91)90481-c.

[215] A.P Girard-Egrot et al. “IgG1-glycolipidic LB films obtained by vertical deposition of an interfacial film formed through proteo-liposome spreading at the air/water interface”. In: *Colloids and Surfaces B: Biointerfaces* 23.4 (2002), 319–325. ISSN: 0927-7765. DOI: 10.1016/s0927-7765(01)00263-6. URL: http://dx.doi.org/10.1016/s0927-7765(01)00263-6.

[216] Shipra Solanki et al. “Langmuir–Blodgett Nanoassemblies of the MoS2–Au Composite at the Air–Water Interface for Dengue Detection”. In: *ACS Applied Materials amp; Interfaces* 10.3 (2018), 3020–3028. ISSN: 1944-8252. DOI: 10.1021/acsami.7b14391. URL: http://dx.doi.org/10.1021/acsami.7b14391.

[217] Joanna Cabaj, Jadwiga Sołoducho, and Anna Nowakowska-Oleksy. “Langmuir–Blodgett film based biosensor for estimation of phenol derivatives”. In: *Sensors and Actuators B: Chemical* 143.2 (2010), 508–515. ISSN: 0925-4005. DOI: 10.1016/j.snb.2009.09.047. URL: http://dx.doi.org/10.1016/j.snb.2009.09.047.

[218] Fatma Gür et al. “Preparation of bio-electrodes via Langmuir-Blodgett technique for pharmaceutical and waste industries and their biosensor application”. In: *Colloids and Surfaces A: Physicochemical and Engineering Aspects* 583 (2019), p. 124005. ISSN: 0927-7757. DOI: 10.1016/j.colsurfa.2019.124005. URL: http://dx.doi.org/10.1016/j.colsurfa.2019.124005.

[219] C.W. Pitt and L.M. Walpita. “Optical waveguiding in Langmuir films”. In: *Electronics Letters* 12.18 (1976), 479–481. ISSN: 1350-911X. DOI: 10.1049/el:19760363. URL: http://dx.doi.org/10.1049/el:19760363.

[220] F. Grunfeld and C.W. Pitt. “Diacetylene Langmuir-Blodgett layers for integrated optics”. In: *Thin Solid Films* 99.1–3 (1983), 249–255. ISSN: 0040-6090. DOI: 10.1016/0040-6090(83)90388-7. URL: http://dx.doi.org/10.1016/0040-6090(83)90388-7.

[221] Werner Hickel et al. “Optical waveguides from novel polymeric Langmuir-Blodgett multilayer assemblies”. In: *Langmuir* 6.8 (1990), 1403–1407. ISSN: 1520-5827. DOI: 10.1021/la00098a014. URL: http://dx.doi.org/10.1021/la00098a014.

[222] Sandeep Cherumukkil et al. “-Extended Bodipy Self-Assembly as Supramolecular Photonic Security Ink and Optical Waveguide”. In: *Advanced Functional Materials* 32.6 (2021). ISSN: 1616-3028. DOI: 10.1002/adfm.202109041. URL: http://dx.doi.org/10.1002/adfm.202109041.

[223] I.R. Girling et al. “Observation of second-harmonic generation form Langmuir-Blodgett multilayers of a hemicyanine dye”. In: *Thin Solid Films* 132.1–4 (1985), 101–112. ISSN: 0040-6090. DOI: 10.1016/0040-6090(85)90462-6. URL: http://dx.doi.org/10.1016/0040-6090(85)90462-6.

[224] I.R. Girling et al. “Observation of second-harmonic generation form Langmuir-Blodgett multilayers of a hemicyanine dye”. In: *Thin Solid Films* 132.1–4 (1985), 101–112. ISSN: 0040-6090. DOI: 10.1016/0040-6090(85)90462-6. URL: http://dx.doi.org/10.1016/0040-6090(85)90462-6.

[225] C. S. Willand and D. J. Williams. “Nonlinear optical properties of polymeric materials”. In: *Berichte der Bunsengesellschaft für physikalische Chemie* 91.11 (1987), 1304–1310. ISSN: 0005-9021. DOI: 10.1002/bbpc.19870911145. URL: http://dx.doi.org/10.1002/bbpc.19870911145.

[226] Christoph Bubeck, Dieter Neher, and Anke Kaltbeitzel. “Nonlinear optical properties of thin organic films”. In: *Makromolekulare Chemie. Macromolecular Symposia* 37.1 (1990), 239–245. ISSN: 0258-0322. DOI:

10.1002/masy.19900370120. URL: http://dx.doi.org/10.1002/masy.19900370120.

[227] P.-A. Chollet, F. Kajzar, and J. Messier. “Electric Field Induced Optical Second Harmonic Generation and Polarization Effects in Polydiacetylene Films”. In: *Polydiacetylenes*. Springer Netherlands, 1985, 317–324. ISBN: 9789401727136. DOI: 10.1007/978-94-017-2713-6_23. URL: http://dx.doi.org/10.1007/978-94-017-2713-6_23.

[228] M. Talukder et al. “Preparation of High Quality Thin Films. SHG Properties of Y-Type LB Films Prepared by Using Moving-Wall Type LB Deposition System”. In: *Organic Thin Films for Photonic Applications*. OTFA. Optica Publishing Group, 1995, p. MD.40. DOI: 10.1364/otfa.1995.md.40. URL: http://dx.doi.org/10.1364/otfa.1995.md.40.

[229] T Sauer et al. “Development of novel chemical sensor devices based on LB films from phthalocyaninato-polysiloxane polymers”. In: *Journal of Physics D: Applied Physics* 23.1 (1990), 79–84. ISSN: 1361-6463. DOI: 10.1088/0022-3727/23/1/013. URL: http://dx.doi.org/10.1088/0022-3727/23/1/013.

[230] Kiyoshi Yase et al. “Molecular orientations of substituted phthalocyaninato-polysiloxanes in Langmuir-Blodgett films”. In: *Thin Solid Films* 210–211 (1992), 22–25. ISSN: 0040-6090. DOI: 10.1016/0040-6090(92)90157-7. URL: http://dx.doi.org/10.1016/0040-6090(92)90157-7.

[231] S. Palacin and A. Barraud. “Highly ordered Langmuir–Blodgett films based on semi-amphiphilic phthalocyanines”. In: *J. Chem. Soc., Chem. Commun.* 1 (1989), 45–47. ISSN: 0022-4936. DOI: 10.1039/c39890000045. URL: http://dx.doi.org/10.1039/c39890000045.

[232] David O. Oluwole et al. “First Example of Nonlinear Optical Materials Based on Nanoconjugates of Sandwich Phthalocyanines with Quantum Dots”. In: *Chemistry – A European Journal* 23.12 (2017), 2820–2830. ISSN: 1521-3765. DOI: 10.1002/chem.201604401. URL: http://dx.doi.org/10.1002/chem.201604401.

[233] Nadine Witkowski et al. “Grafting, self-organization and reactivity of double-decker rare-earth phthalocyanine”. In: *Journal of Porphyrins and Phthalocyanines* 23.11n12 (2019), 1523–1534. ISSN: 1099-1409. DOI: 10.1142/s1088424619501736. URL: http://dx.doi.org/10.1142/s1088424619501736.

[234] Gerald Brezesinski and Helmuth Möhwald. “Langmuir monolayers to study interactions at model membrane surfaces”. In: *Advances in Colloid and Interface Science* 100–102 (2003), 563–584. ISSN: 0001-8686. DOI: 10.1016/s0001-8686(02)00071-4. URL: http://dx.doi.org/10.1016/s0001-8686(02)00071-4.

[235] Gerald Brezesinski and Helmuth Möhwald. “Langmuir monolayers to study interactions at model membrane surfaces”. In: *Advances in Colloid and Interface Science* 100–102 (2003), 563–584. ISSN: 0001-8686. DOI: 10.1016/s0001-8686(02)00071-4. URL: http://dx.doi.org/10.1016/s0001-8686(02)00071-4.

[236] Andreas Santamaria et al. “Investigation on the relationship between lipid composition and structure in model membranes composed of extracted natural phospholipids”. In: (2022). DOI: 10.26434/chemrxiv-2022-mrfmx. URL: http://dx.doi.org/10.26434/chemrxiv-2022-mrfmx.

[237] Xueying Guo and Wuge H. Briscoe. “Molecular interactions, elastic properties, and nanostructure of Langmuir bacterial-lipid monolayers: Towards solving the mystery in bacterial membrane asymmetry”. In: *Current Opinion in Colloid amp; Interface Science* 67 (2023), p. 101731. ISSN: 1359-0294. DOI: 10.1016/j.cocis.2023.101731. URL: http://dx.doi.org/10.1016/j.cocis.2023.101731.

[238] Arun Radhakrishnan and Harden McConnell. “Condensed complexes in vesicles containing cholesterol and phospholipids”. In: *Proceedings of the National Academy of Sciences* 102.36 (2005), 12662–12666. ISSN: 1091-6490. DOI: 10.1073/pnas.0506043102. URL: http://dx.doi.org/10.1073/pnas.0506043102.

[239] E. B. Watkins et al. “Membrane texture induced by specific protein binding and receptor clustering: active roles for lipids in cellular function”. In: *Proceedings of the National Academy of Sciences* 108.17 (2011), 6975–6980. ISSN: 1091-6490. DOI: 10.1073/pnas.1014579108. URL: http://dx.doi.org/10.1073/pnas.1014579108.

[240] André C. Machado and Luciano Caseli. “Interaction of nitrofurantoin with lipid langmuir monolayers as cellular membrane models distinguished with tensiometry and infrared spectroscopy”. In: *Colloids and Surfaces B: Biointerfaces* 188 (2020), p. 110794. ISSN: 0927-7765. DOI:

10.1016/j.colsurfb.2020.110794. URL: http://dx.doi.org/10.1016/j.colsurfb.2020.110794.

[241] Richard A. Dluhy et al. "Design and Interfacing of an Automated Langmuir-Type Film Balance to an FT-IR Spectrometer". In: *Applied Spectroscopy* 42.7 (1988), 1289–1293. ISSN: 1943-3530. DOI: 10.1366/0003702884430047. URL: http://dx.doi.org/10.1366/0003702884430047.

[242] Margaret H. Vaughan et al. "Fourier transform infrared spectroscopic studies on model biological membranes deposited by the Langmuir-Blodgett technique". In: *Thin Solid Films* 210–211 (1992), 574–576. ISSN: 0040-6090. DOI: 10.1016/0040-6090(92)90345-c. URL: http://dx.doi.org/10.1016/0040-6090(92)90345-c.

[243] João Borges et al. "Recent Developments in Layer-by-Layer Assembly for Drug Delivery and Tissue Engineering Applications". In: *Advanced Healthcare Materials* 13.8 (2024). ISSN: 2192-2659. DOI: 10.1002/adhm.202302713. URL: http://dx.doi.org/10.1002/adhm.202302713.

[244] Hyunah Ahn et al. "Hierarchical Topography with Tunable Micro- and Nanoarchitectonics for Highly Enhanced Cardiomyocyte Maturation via Multi-Scale Mechanotransduction". In: *Advanced Healthcare Materials* 12.12 (2023). ISSN: 2192-2659. DOI: 10.1002/adhm.202202371. URL: http://dx.doi.org/10.1002/adhm.202202371.

[245] Katsuhiko Ariga et al. "Materials Nanoarchitectonics as Cell Regulators". In: *ChemNanoMat* 5.6 (2019), 692–702. ISSN: 2199-692X. DOI: 10.1002/cnma.201900207. URL: http://dx.doi.org/10.1002/cnma.201900207.

[246] Jingwen Song, Xiaofang Jia, and Katsuhiko Ariga. "Methods with Nanoarchitectonics for Small Molecules and Nanostructures to Regulate Living Cells". In: *Small Methods* 4.10 (2020). ISSN: 2366-9608. DOI: 10.1002/smtd.202000500. URL: http://dx.doi.org/10.1002/smtd.202000500.

[247] Venkata Krishnan et al. "Vortex-Aligned Fullerene Nanowhiskers as a Scaffold for Orienting Cell Growth". In: *ACS Applied Materials amp; Interfaces* 7.28 (2015), 15667–15673. ISSN: 1944-8252. DOI: 10.1021/acsami.5b04811. URL: http://dx.doi.org/10.1021/acsami.5b04811.

[248] Kosuke Minami et al. “Highly Ordered 1D Fullerene Crystals for Concurrent Control of Macroscopic Cellular Orientation and Differentiation toward Large-Scale Tissue Engineering”. In: *Advanced Materials* 27.27 (2015), 4020–4026. ISSN: 1521-4095. DOI: 10.1002/adma.201501690. URL: http://dx.doi.org/10.1002/adma.201501690.

[249] Jingwen Song et al. “Large-Area Aligned Fullerene Nanocrystal Scaffolds as Culture Substrates for Enhancing Mesenchymal Stem Cell Self-Renewal and Multipotency”. In: *ACS Applied Nano Materials* 3.7 (2020), 6497–6506. ISSN: 2574-0970. DOI: 10.1021/acsanm.0c00973. URL: http://dx.doi.org/10.1021/acsanm.0c00973.

[250] Wenyan Lyu et al. “Manipulating the Dynamic Adaptivity of a Fluid Interface to Maintain the Multipotency of Mesenchymal Stromal Cells”. In: *Advanced Healthcare Materials* 12.24 (2023). ISSN: 2192-2659. DOI: 10.1002/adhm.202300666. URL: http://dx.doi.org/10.1002/adhm.202300666.

[251] Xiaofang Jia et al. “Adaptive liquid interfaces induce neuronal differentiation of mesenchymal stem cells through lipid raft assembly”. In: *Nature Communications* 13.1 (2022). ISSN: 2041-1723. DOI: 10.1038/s41467-022-30622-y. URL: http://dx.doi.org/10.1038/s41467-022-30622-y.

[252] Xiaofang Jia et al. “Modulation of Mesenchymal Stem Cells Mechanosensing at Fluid Interfaces by Tailored Self-Assembled Protein Monolayers”. In: *Small* 15.5 (2019). ISSN: 1613-6829. DOI: 10.1002/smll.201804640. URL: http://dx.doi.org/10.1002/smll.201804640.

[253] Xiaofang Jia et al. “Adaptive Liquid Interfacially Assembled Protein Nanosheets for Guiding Mesenchymal Stem Cell Fate”. In: *Advanced Materials* 32.4 (2019). ISSN: 1521-4095. DOI: 10.1002/adma.201905942. URL: http://dx.doi.org/10.1002/adma.201905942.

[254] Jingwen Song et al. “Bio-gel nanoarchitectonics in tissue engineering”. In: *Nanoscale* 16.28 (2024), 13230–13246. ISSN: 2040-3372. DOI: 10.1039/d4nr00609g. URL: http://dx.doi.org/10.1039/d4nr00609g.

[255] Rikako Hama et al. “Recent Developments in Biopolymer-Based Hydrogels for Tissue Engineering Applications”. In: *Biomolecules* 13.2 (2023), p. 280. ISSN: 2218-273X. DOI: 10.3390/biom13020280. URL: http://dx.doi.org/10.3390/biom13020280.

[256] T. Dubrovsky, S. Vakula, and C. Nicolini. “Preparation and immobilization of Langmuir-Blodgett films of antibodies conjugated to enzymes for potentiometric sensor application”. In: *Sensors and Actuators B: Chemical* 22.1 (1994), 69–73. ISSN: 0925-4005. DOI: 10.1016/0925-4005(94)01258-x. URL: http://dx.doi.org/10.1016/0925-4005(94)01258-x.

[257] M. Sriyudthsak, H. Yamagishi, and T. Moriizumi. “Enzyme-immobilized Langmuir-Blodgett film for a biosensor”. In: *Thin Solid Films* 160.1–2 (1988), 463–469. ISSN: 0040-6090. DOI: 10.1016/0040-6090(88)90092-2. URL: http://dx.doi.org/10.1016/0040-6090(88)90092-2.

[258] Agnès P. Girard-Egrot, Stéphanie Godoy, and Loïc J. Blum. “Enzyme association with lipidic Langmuir–Blodgett films: Interests and applications in nanobioscience”. In: *Advances in Colloid and Interface Science* 116.1–3 (2005), 205–225. ISSN: 0001-8686. DOI: 10.1016/j.cis.2005.04.006. URL: http://dx.doi.org/10.1016/j.cis.2005.04.006.

[259] Stéphanie Godoy et al. “Kinetics Study of Bungarus fasciatus Venom Acetylcholinesterase Immobilised on a Langmuir–Blodgett Proteo-Glycolipidic Bilayer”. In: *ChemBioChem* 6.2 (2005), 395–404. ISSN: 1439-7633. DOI: 10.1002/cbic.200400277. URL: http://dx.doi.org/10.1002/cbic.200400277.

[260] Camila Gouveia Barbosa et al. “Copolymers and enzymes blended as LB films changing the bioelectronics properties of interfaces”. In: *Colloid and Interface Science Communications* 27 (2018), 40–44. ISSN: 2215-0382. DOI: 10.1016/j.colcom.2018.10.001. URL: http://dx.doi.org/10.1016/j.colcom.2018.10.001.

[261] Felipe Tejada Araujo, Laura Oliveira Peres, and Luciano Caseli. “Conjugated Polymers Blended with Lipids and Galactosidase as Langmuir–Blodgett Films To Control the Biosensing Properties of Nanostructured Surfaces”. In: *Langmuir* 35.22 (2019), 7294–7303. ISSN: 1520-5827. DOI: 10.1021/acs.langmuir.9b00536. URL: http://dx.doi.org/10.1021/acs.langmuir.9b00536.

[262] Katsuhiko Ariga. “Interfaces Working for Biology: Solving Biological Mysteries and Opening Up Future Nanoarchitectonics”. In: *Chem-*

NanoMat 2.5 (2016), 333–343. ISSN: 2199-692X. DOI: 10.1002/cnma.201600053. URL: http://dx.doi.org/10.1002/cnma.201600053.

[263] Anna M. Bibo and Ian R. Peterson. "Phase diagrams of monolayers of the long chain fatty acids". In: *Advanced Materials* 2.6–7 (1990), 309–311. ISSN: 1521-4095. DOI: 10.1002/adma.19900020608. URL: http://dx.doi.org/10.1002/adma.19900020608.

[264] Katsuhiko Ariga. "Langmuir–Blodgett Nanoarchitectonics, Out of the Box". In: *Accounts of Materials Research* 3.4 (2021), 404–410. ISSN: 2643-6728. DOI: 10.1021/accountsmr.1c00240. URL: http://dx.doi.org/10.1021/accountsmr.1c00240.

[265] Syed Arshad Hussain et al. "Unique supramolecular assembly through Langmuir – Blodgett (LB) technique". In: *Heliyon* 4.12 (2018), e01038. ISSN: 2405-8440. DOI: 10.1016/j.heliyon.2018.e01038. URL: http://dx.doi.org/10.1016/j.heliyon.2018.e01038.

[266] V. I. Troitsky et al. "Instrument for depositing Langmuir–Blodgett films composed of alternating monolayers using a protective layer of water". In: *Review of Scientific Instruments* 67.12 (1996), 4216–4223. ISSN: 1089-7623. DOI: 10.1063/1.1147571. URL: http://dx.doi.org/10.1063/1.1147571.

[267] Luzhu Xu et al. "Continuous Langmuir–Blodgett Deposition and Transfer by Controlled Edge-to-Edge Assembly of Floating 2D Materials". In: *Langmuir* 35.1 (2018), 51–59. ISSN: 1520-5827. DOI: 10.1021/acs.langmuir.8b03173. URL: http://dx.doi.org/10.1021/acs.langmuir.8b03173.

[268] Hyun-Kyung Kim et al. "In Situ synthesis of Three-Dimensional Self-Assembled SnO2 - Reduced Graphene Oxide Architecture for Lithium Ion Batteries". In: *ECS Meeting Abstracts* MA2015-01.2 (2015), 573–573. ISSN: 2151-2043. DOI: 10.1149/ma2015-01/2/573. URL: http://dx.doi.org/10.1149/ma2015-01/2/573.

[269] Sriramprabha Ramasamy, Ponpandian Nagamony, and Viswanathan Chinnuswamy. "Self-assembled SnO2/reduced graphene oxide nanocomposites via Langmuir-Blodgett technique as anode materials for Li-ion batteries". In: *Materials Letters* 218 (2018), 295–298. ISSN: 0167-577X. DOI: 10.1016/j.matlet.2018.01.177. URL: http://dx.doi.org/10.1016/j.matlet.2018.01.177.

[270] Mohamed B. Zakaria et al. "Self-Construction from 2D to 3D: One-Pot Layer-by-Layer Assembly of Graphene Oxide Sheets Held Together by Coordination Polymers". In: *Angewandte Chemie* 128.29 (2016), 8566–8570. ISSN: 1521-3757. DOI: 10.1002/ange.201603223. URL: http://dx.doi.org/10.1002/ange.201603223.

[271] Yan Nie et al. "Controlled fabrication of biocompatible graphene oxide Langmuir–Blodgett films by size and surface property manipulation". In: *Journal of Dispersion Science and Technology* 43.12 (2021), 1747–1754. ISSN: 1532-2351. DOI: 10.1080/01932691.2021.1880430. URL: http://dx.doi.org/10.1080/01932691.2021.1880430.

[272] Katharine L. Harrison, Laura B. Biedermann, and Kevin R. Zavadil. "Mechanical Properties of Water-Assembled Graphene Oxide Langmuir Monolayers: Guiding Controlled Transfer". In: *Langmuir* 31.36 (2015), 9825–9832. ISSN: 1520-5827. DOI: 10.1021/acs.langmuir.5b01994. URL: http://dx.doi.org/10.1021/acs.langmuir.5b01994.

[273] Jongdeok Park et al. "Phase transitions and morphology control of Langmuir-Blodgett (LB) films of graphene oxide". In: *Journal of Colloid and Interface Science* 684 (2025), 215–224. ISSN: 0021-9797. DOI: 10.1016/j.jcis.2025.01.044. URL: http://dx.doi.org/10.1016/j.jcis.2025.01.044.

[274] Gurpreet Kaur Bhullar, Ramneek Kaur, and K.K. Raina. "Growth, Morphology, and Electrical Characterization of Polyaniline–ZnO Nanocomposite Langmuir–Blodgett Thin Films". In: *Journal of Electronic Materials* 44.10 (2015), 3422–3429. ISSN: 1543-186X. DOI: 10.1007/s11664-015-3868-4. URL: http://dx.doi.org/10.1007/s11664-015-3868-4.

[275] Xiaolin Li et al. "LangmuiràíBlodgett Assembly of Densely Aligned Single-Walled Carbon Nanotubes from Bulk Materials". In: *Journal of the American Chemical Society* 129.16 (2007), 4890Äì4891. ISSN: 1520-5126. DOI: 10.1021/ja071114e. URL: http://dx.doi.org/10.1021/ja071114e.

[276] Xiaolin Li et al. "LangmuiràíBlodgett Assembly of Densely Aligned Single-Walled Carbon Nanotubes from Bulk Materials". In: *Journal of the American Chemical Society* 129.16 (2007), 4890Äì4891. ISSN:

1520-5126. DOI: 10.1021/ja071114e. URL: http://dx.doi.org/10.1021/ja071114e.

[277] Michal Swierczewski and Thomas Bürgi. "Langmuir and Langmuir–Blodgett Films of Gold and Silver Nanoparticles". In: *Langmuir* 39.6 (2023), 2135–2151. ISSN: 1520-5827. DOI: 10.1021/acs.langmuir.2c02715. URL: http://dx.doi.org/10.1021/acs.langmuir.2c02715.

[278] S. Paul et al. "LangmuirBlodgett Film Deposition of Metallic Nanoparticles and Their Application to Electronic Memory Structures". In: *Nano Letters* 3.4 (2003), 533–536. ISSN: 1530-6992. DOI: 10.1021/nl034008t. URL: http://dx.doi.org/10.1021/nl034008t.

[279] Qijie Guo et al. "Patterned LangmuirBlodgett Films of Monodisperse Nanoparticles of Iron Oxide Using Soft Lithography". In: *Journal of the American Chemical Society* 125.3 (2002), 630–631. ISSN: 1520-5126. DOI: 10.1021/ja0275764. URL: http://dx.doi.org/10.1021/ja0275764.

[280] Konrad Cyprych et al. "Plasmonic Nanoparticles Driven Enhanced Light Amplification in a Local 2D and 3D Self-Assembly". In: *Nanomaterials* 8.12 (2018), p. 1051. ISSN: 2079-4991. DOI: 10.3390/nano8121051. URL: http://dx.doi.org/10.3390/nano8121051.

[281] Joydeep Chowdhury, Somsubhra Saha, and Manash Ghosh. "Self-assembly of metal nanocolloids entrapped in Langmuir Blodgett Film templates: Evidence of efficient SERS sensing platforms". In: *Materials Today: Proceedings* 5.3 (2018), 10071–10076. ISSN: 2214-7853. DOI: 10.1016/j.matpr.2017.11.001. URL: http://dx.doi.org/10.1016/j.matpr.2017.11.001.

[282] Chia-Chen Lin et al. "Langmuir-Blodgett Films of Alkanethiolate Gold Nanorods". In: *Journal of the Chinese Chemical Society* 50.5 (2003), 1015–1021. ISSN: 2192-6549. DOI: 10.1002/jccs.200300143. URL: http://dx.doi.org/10.1002/jccs.200300143.

[283] Ran Wang et al. "Self-Assembled Black Phosphorus-Based Composite Langmuir–Blodgett Films with an Enhanced Photocurrent Generation Capability and Surface-Enhanced Raman Scattering Properties". In: *ACS Omega* 6.6 (2021), 4430–4439. ISSN: 2470-1343. DOI: 10.1021/acsomega.0c05832. URL: http://dx.doi.org/10.1021/acsomega.0c05832.

[284] Na Li et al. "Chemical gas sensor, surface enhanced Raman scattering and photoelectrics of composite Langmuir-Blodgett films consisting of polypeptide and dye molecules". In: *Colloids and Surfaces A: Physicochemical and Engineering Aspects* 663 (2023), p. 131067. ISSN: 0927-7757. DOI: 10.1016/j.colsurfa.2023.131067. URL: http://dx.doi.org/10.1016/j.colsurfa.2023.131067.

[285] Rie Makiura. "Creation of metal–organic framework nanosheets by the Langmuir-Blodgett technique". In: *Coordination Chemistry Reviews* 469 (2022), p. 214650. ISSN: 0010-8545. DOI: 10.1016/j.ccr.2022.214650. URL: http://dx.doi.org/10.1016/j.ccr.2022.214650.

[286] Bin Bai, Dong Wang, and Li-Jun Wan. "Synthesis of Covalent Organic Framework Films at Interfaces". In: *Bulletin of the Chemical Society of Japan* 94.3 (2021), 1090–1098. ISSN: 1348-0634. DOI: 10.1246/bcsj.20200391. URL: http://dx.doi.org/10.1246/bcsj.20200391.

[287] Soraya Sangiao et al. "All-Carbon Electrode Molecular Electronic Devices Based on Langmuir–Blodgett Monolayers". In: *Small* 13.7 (2016). ISSN: 1613-6829. DOI: 10.1002/smll.201603207. URL: http://dx.doi.org/10.1002/smll.201603207.

[288] Camilla Baratto et al. "On the alignment of ZnO nanowires by Langmuir – Blodgett technique for sensing application". In: *Applied Surface Science* 528 (2020), p. 146959. ISSN: 0169-4332. DOI: 10.1016/j.apsusc.2020.146959. URL: http://dx.doi.org/10.1016/j.apsusc.2020.146959.

[289] G. Decher, J.D. Hong, and J. Schmitt. "Buildup of ultrathin multilayer films by a self-assembly process: III. Consecutively alternating adsorption of anionic and cationic polyelectrolytes on charged surfaces". In: *Thin Solid Films* 210–211 (1992), 831–835. ISSN: 0040-6090. DOI: 10.1016/0040-6090(92)90417-a. URL: http://dx.doi.org/10.1016/0040-6090(92)90417-a.

[290] Wieslaw I. Gruszecki, Mariusz Gagos, and Peter Kernen. "Polyene antibiotic amphotericin B in monomolecular layers: spectrophotometric and scanning force microscopic analysis". In: *FEBS Letters* 524.1–3 (2002), 92–96. ISSN: 1873-3468. DOI: 10.1016/s0014-5793(02)03009-0. URL: http://dx.doi.org/10.1016/s0014-5793(02)03009-0.

[291] Masato Ito et al. “Hyper 100 °C Langmuir–Blodgett (Langmuir–Schaefer) Technique for Organized Ultrathin Film of Polymeric Semiconductors”. In: *Langmuir* 38.17 (2021), 5237–5247. ISSN: 1520-5827. DOI: 10.1021/acs.langmuir.1c02596. URL: http://dx.doi.org/10.1021/acs.langmuir.1c02596.

[292] Masato Ito et al. “100 °C-Langmuir–Blodgett Method for Fabricating Highly Oriented, Ultrathin Films of Polymeric Semiconductors”. In: *ACS Applied Materials amp; Interfaces* 12.50 (2020), 56522–56529. ISSN: 1944-8252. DOI: 10.1021/acsami.0c18349. URL: http://dx.doi.org/10.1021/acsami.0c18349.

[293] Takatoshi Maeda et al. “Vortex Flow-controlled Circularly Polarized Luminescence of Achiral Pt(II) Complex Aggregates Assembled at the Air-Water Interface”. In: *Small Methods* 6.12 (2022). ISSN: 2366-9608. DOI: 10.1002/smtd.202200936. URL: http://dx.doi.org/10.1002/smtd.202200936.

[294] Taizo Mori et al. “Carbon Nanosheets by Morphology-Retained Carbonization of Two-Dimensional Assembled Anisotropic Carbon Nanorings”. In: *Angewandte Chemie International Edition* 57.31 (2018), 9679–9683. ISSN: 1521-3773. DOI: 10.1002/anie.201803859. URL: http://dx.doi.org/10.1002/anie.201803859.

[295] Mutsuo Matsumoto et al. “A trough with radial compression for studies of monolayers and fabrication of Langmuir-Blodgett films”. In: *Thin Solid Films* 280.1–2 (1996), 238–243. ISSN: 0040-6090. DOI: 10.1016/0040-6090(95)08243-3. URL: http://dx.doi.org/10.1016/0040-6090(95)08243-3.

[296] André Barraud and Michel Vandevyver. “A trough for continuous fabrication of Langmuir-Blodgett films”. In: *Thin Solid Films* 99.1–3 (1983), 221–225. ISSN: 0040-6090. DOI: 10.1016/0040-6090(83)90384-x. URL: http://dx.doi.org/10.1016/0040-6090(83)90384-x.

[297] Katsuhiko Ariga, Jonathan P. Hill, and Qingmin Ji. “Layer-by-layer assembly as a versatile bottom-up nanofabrication technique for exploratory research and realistic application”. In: *Physical Chemistry Chemical Physics* 9.19 (2007), p. 2319. ISSN: 1463-9084. DOI: 10.1039/b700410a. URL: http://dx.doi.org/10.1039/b700410a.

[298] Ming Zhou et al. "Controlled Synthesis of Large-Area and Patterned Electrochemically Reduced Graphene Oxide Films". In: *Chemistry – A European Journal* 15.25 (2009), 6116–6120. ISSN: 1521-3765. DOI: 10.1002/chem.200900596. URL: http://dx.doi.org/10.1002/chem.200900596.

[299] Elena Gorenskaia et al. "Fabrication of metallic and non-metallic top electrodes for large-area molecular junctions". In: *Nanoscale* 13.20 (2021), 9055–9074. ISSN: 2040-3372. DOI: 10.1039/d1nr00917f. URL: http://dx.doi.org/10.1039/d1nr00917f.

[300] Markus Retsch et al. "Fabrication of Large-Area, Transferable Colloidal Monolayers Utilizing Self-Assembly at the Air/Water Interface". In: *Macromolecular Chemistry and Physics* 210.3–4 (2009), 230–241. ISSN: 1521-3935. DOI: 10.1002/macp.200800484. URL: http://dx.doi.org/10.1002/macp.200800484.

[301] Hyeri Kim et al. "Spontaneous hybrids of graphene and carbon nanotube arrays at the liquid–gas interface for Li-ion battery anodes". In: *Chemical Communications* 54.41 (2018), 5229–5232. ISSN: 1364-548X. DOI: 10.1039/c8cc02148a. URL: http://dx.doi.org/10.1039/c8cc02148a.

[302] Hee-Sung Jeong et al. "Oriented layered assemblies of graphene nanosheets/Fe3O4 nanoparticles as a superior anode material for lithium ion batteries". In: *Applied Surface Science* 508 (2020), p. 144416. ISSN: 0169-4332. DOI: 10.1016/j.apsusc.2019.144416. URL: http://dx.doi.org/10.1016/j.apsusc.2019.144416.

[303] Hee-Sung Jeong et al. "Oriented wrinkle textures of free-standing graphene nanosheets: application as a high-performance lithium-ion battery anode". In: *Carbon Letters* 31.2 (2020), 277–285. ISSN: 2233-4998. DOI: 10.1007/s42823-020-00163-9. URL: http://dx.doi.org/10.1007/s42823-020-00163-9.

[304] Xin-Bing Cheng et al. "A Review of Solid Electrolyte Interphases on Lithium Metal Anode". In: *Advanced Science* 3.3 (2015). ISSN: 2198-3844. DOI: 10.1002/advs.201500213. URL: http://dx.doi.org/10.1002/advs.201500213.

[305] Qin Liu et al. "Langmuir–Blodgett Nanowire Devices for In Situ Probing of Zinc-Ion Batteries". In: *Small* 15.30 (2019). ISSN: 1613-6829. DOI: 10.1002/smll.201902141. URL: http://dx.doi.org/10.1002/smll.201902141.

[306] Mutsuyoshi Matsumoto, Hiroaki Tachibana, and Takayoshi Nakamura. "Applications of Organic Conductors: Molecular Electronics". In: *Organic Conductors*. CRC Press, 2022, 759–790. ISBN: 9780367811907. DOI: 10.1201/9780367811907-16. URL: http://dx.doi.org/10.1201/9780367811907-16.

[307] P.S. Vincett and G.G. Roberts. "Electrical and photoelectrical transport properties of Langmuir-Blodgett films and a discussion of possible device applications". In: *Thin Solid Films* 68.1 (1980), 135–171. ISSN: 0040-6090. DOI: 10.1016/0040-6090(80)90143-1. URL: http://dx.doi.org/10.1016/0040-6090(80)90143-1.

[308] Martin R. Bryce and Michael C. Petty. "Electrically conductive Langmuir–Blodgett films of charge-transfer materials". In: *Nature* 374.6525 (1995), 771–776. ISSN: 1476-4687. DOI: 10.1038/374771a0. URL: http://dx.doi.org/10.1038/374771a0.

[309] Robert M Metzger and Charles A Panetta. "Langmur-Blodgett films of potential organic rectifiers: New scanning tunneling microscopy and non-linear optics results". In: *Synthetic Metals* 42.1–2 (1991), 1407–1413. ISSN: 0379-6779. DOI: 10.1016/0379-6779(91)91868-b. URL: http://dx.doi.org/10.1016/0379-6779(91)91868-b.

[310] Douglas H. Banning et al. "ChemFET Anion Sensor Based on MOF Nanoparticles". In: *ChemPlusChem* 90.1 (2024). ISSN: 2192-6506. DOI: 10.1002/cplu.202400622. URL: http://dx.doi.org/10.1002/cplu.202400622.

[311] Miguel A. Andrés et al. "Methanol and Humidity Capacitive Sensors Based on Thin Films of MOF Nanoparticles". In: *ACS Applied Materials amp; Interfaces* 12.3 (2020), 4155–4162. ISSN: 1944-8252. DOI: 10.1021/acsami.9b20763. URL: http://dx.doi.org/10.1021/acsami.9b20763.

[312] Fabio A. Scholl et al. "Carbon Nanotubes Arranged As Smart Interfaces in Lipid Langmuir–Blodgett Films Enhancing the Enzymatic Properties of Penicillinase for Biosensing Applications". In: *ACS Ap-*

plied Materials amp; Interfaces 9.36 (2017), 31054–31066. ISSN: 1944-8252. DOI: 10.1021/acsami.7b08095. URL: http://dx.doi.org/10.1021/acsami.7b08095.

[313] Ritu Malik, Nirav Joshi, and Vijay K. Tomer. "Advances in the designs and mechanisms of MoO3 nanostructures for gas sensors: a holistic review". In: *Materials Advances* 2.13 (2021), 4190–4227. ISSN: 2633-5409. DOI: 10.1039/d1ma00374g. URL: http://dx.doi.org/10.1039/d1ma00374g.

[314] Na Li et al. "Gas-Responsive and Self-Powered Visual Composite Langmuir–Blodgett Films for Ultrathin Gas Sensors". In: *Langmuir* 38.21 (2022), 6761–6770. ISSN: 1520-5827. DOI: 10.1021/acs.langmuir.2c00835. URL: http://dx.doi.org/10.1021/acs.langmuir.2c00835.

[315] Tan Nhiem Ly and Sangkwon Park. "Highly sensitive gas sensor using hierarchically self-assembled thin films of graphene oxide and gold nanoparticles". In: *Journal of Industrial and Engineering Chemistry* 67 (2018), 417–428. ISSN: 1226-086X. DOI: 10.1016/j.jiec.2018.07.016. URL: http://dx.doi.org/10.1016/j.jiec.2018.07.016.

[316] Tong Zhang et al. "Fewer-layer conductive metal-organic Langmuir-Blodgett films as electrocatalysts enable an ultralow detection limit of H2O2". In: *Applied Surface Science* 539 (2021), p. 148255. ISSN: 0169-4332. DOI: 10.1016/j.apsusc.2020.148255. URL: http://dx.doi.org/10.1016/j.apsusc.2020.148255.

[317] C.S. Winter et al. "Langmuir-Blodgett films from preformed polymers: derivatives of octadec-1-ene-maleic anhydride copolymers". In: *Thin Solid Films* 134.1–3 (1985), 49–55. ISSN: 0040-6090. DOI: 10.1016/0040-6090(85)90116-6. URL: http://dx.doi.org/10.1016/0040-6090(85)90116-6.

[318] Motoko Uchida et al. "Control of surface chemical structure and functional property of Langmuir-Blodgett film composed of new polymerizable amphiphile with a sodium sulfonate". In: *Macromolecules* 24.11 (1991), 3238–3243. ISSN: 1520-5835. DOI: 10.1021/ma00011a031. URL: http://dx.doi.org/10.1021/ma00011a031.

[319] Fábio A. Scholl et al. "Exploring Langmuir-Blodgett films with phospholipid-graphene oxide/MnO2 as a hybrid nanostructured interface for supercapacitor applications". In: *Colloids and Surfaces A: Physicochemical and Engineering Aspects* 664 (2023), p. 131128. ISSN: 0927-7757. DOI: 10.1016/j.colsurfa.2023.131128. URL: http://dx.doi.org/10.1016/j.colsurfa.2023.131128.

[320] M. S. Kim et al. "Fabricating multifunctional nanoparticle membranes by a fast layer-by-layer Langmuir–Blodgett process: application in lithium–sulfur batteries". In: *Journal of Materials Chemistry A* 4.38 (2016), 14709–14719. ISSN: 2050-7496. DOI: 10.1039/c6ta06018h. URL: http://dx.doi.org/10.1039/c6ta06018h.

[321] Tianshui Zheng et al. "Stability of Langmuir-Blodgett film/alumina, and Langmuir-Blodgett film/gold multilayer structures". In: *Thin Solid Films* 197.1–2 (1991), 327–333. ISSN: 0040-6090. DOI: 10.1016/0040-6090(91)90243-q. URL: http://dx.doi.org/10.1016/0040-6090(91)90243-q.

[322] A.J. Pal et al. "Langmuir-Blodgett films of conjugated polymers: electroluminescence and charge transport mechanisms". In: *IEEE Journal of Selected Topics in Quantum Electronics* 4.1 (1998), 137–143. ISSN: 1077-260X. DOI: 10.1109/2944.669489. URL: http://dx.doi.org/10.1109/2944.669489.

[323] Gero Decher. "Fuzzy Nanoassemblies: Toward Layered Polymeric Multicomposites". In: *Science* 277.5330 (1997), 1232–1237. ISSN: 1095-9203. DOI: 10.1126/science.277.5330.1232. URL: http://dx.doi.org/10.1126/science.277.5330.1232.

[324] F. W. Embs, H. H. Winter, and G. Wegner. "Langmuir-Blodgett multilayer assembly by a continuous process using a steadily flowing subphase". In: *Langmuir* 9.7 (1993), 1618–1621. ISSN: 1520-5827. DOI: 10.1021/la00031a003. URL: http://dx.doi.org/10.1021/la00031a003.

[325] P. H. B. Aoki et al. "Layer-by-Layer Technique as a New Approach to Produce Nanostructured Films Containing Phospholipids as Transducers in Sensing Applications". In: *Langmuir* 25.4 (2009), 2331–2338. ISSN: 1520-5827. DOI: 10.1021/la802696j. URL: http://dx.doi.org/10.1021/la802696j.

[326] Katsuhiko Ariga. "Nanoarchitectonics Revolution and Evolution: From Small Science to Big Technology". In: *Small Science* 1.1 (2020). ISSN: 2688-4046. DOI: 10.1002/smsc.202000032. URL: http://dx.doi.org/10.1002/smsc.202000032.

[327] Katsuhiko Ariga and Yusuke Yamauchi. "Nanoarchitectonics from Atom to Life". In: *Chemistry – An Asian Journal* 15.6 (2020), 718–728. ISSN: 1861-471X. DOI: 10.1002/asia.202000106. URL: http://dx.doi.org/10.1002/asia.202000106.

[328] Katsuhiko Ariga and Rawil Fakhrullin. "Materials Nanoarchitectonics from Atom to Living Cell: A Method for Everything". In: *Bulletin of the Chemical Society of Japan* 95.5 (2022), 774–795. ISSN: 1348-0634. DOI: 10.1246/bcsj.20220071. URL: http://dx.doi.org/10.1246/bcsj.20220071.

[329] Katsuhiko Ariga. "Nanoarchitectonics: what's coming next after nanotechnology?" In: *Nanoscale Horizons* 6.5 (2021), 364–378. ISSN: 2055-6764. DOI: 10.1039/d0nh00680g. URL: http://dx.doi.org/10.1039/d0nh00680g.

[330] Andrea R. Tao, Jiaxing Huang, and Peidong Yang. "LangmuirBlodgettry of Nanocrystals and Nanowires". In: *Accounts of Chemical Research* 41.12 (2008), pp. 1662–1673. ISSN: 0001-4842. DOI: 10.1021/ar8000525. URL: https://doi.org/10.1021/ar8000525.

[331] Jingwen Song et al. "Lateral nanoarchitectonics from nano to life: ongoing challenges in interfacial chemical science". In: *Chemical Science* 15.45 (2024), 18715–18750. ISSN: 2041-6539. DOI: 10.1039/d4sc05575f. URL: http://dx.doi.org/10.1039/d4sc05575f.

[332] Michihiro Ohnishi et al. "Gassensitivity of Modified Surfaces of a Surface Acoustic Wave Device by Chemical Adsorption Technique and Langmuir-Blodgett Technique". In: *Japanese Journal of Applied Physics* 33.10R (1994), p. 5981. ISSN: 1347-4065. DOI: 10.1143/jjap.33.5981. URL: http://dx.doi.org/10.1143/jjap.33.5981.

S10 Second Paper generated using Strategy 2 (137 input papers)

Langmuir and Langmuir-Blodgett Films: A Survey of Fundamentals, Advances, and Applications in Molecular Assembly

April 23, 2025

Contents

1 Introduction 2

2 Fundamentals of Langmuir and Langmuir-Blodgett Films 8

3 Physicochemical Characterization Techniques for Langmuir Monolayers 14

3.1 Surface Potential Measurements 14

3.2 Conductance Measurements 19

3.3 Maxwell Displacement Current (MDC) Measurements 21

4 Microscopic Investigation of Langmuir Films 22

4.1 Fluorescence Microscopy 23

4.2 Brewster Angle Microscopy (BAM) 25

5 Spectroscopic Characterization of Langmuir Films 28

5.1 UV-Vis Spectroscopy 28

5.2 Infrared Spectroscopy (IR) 30

5.3 Raman Spectroscopy 34

6 Advanced Topics and Emerging Trends 35

6.1 Langmuir-Blodgett Films from Novel Materials 37

6.2 Langmuir-Blodgett Films in Advanced Applications 39

6.3 Influence of Subphase Composition 42
6.4 Molecular Machines and Responsive Systems 43

7 Challenges and Future Directions 45

8 Conclusion 49

Abstract

Langmuir (L) and Langmuir-Blodgett (LB) techniques represent pivotal methods for fabricating highly ordered ultrathin films with nanoscale precision over molecular arrangement and thickness. This survey provides a comprehensive overview of the field, tracing its historical development from early surface science studies to its current status. It delves into the fundamental principles governing the formation and behavior of Langmuir monolayers—typically amphiphilic molecules organized at the air-water interface—including their thermodynamics, phase transitions, and characterization via surface pressure-area isotherms. The subsequent controlled transfer of these monolayers onto solid substrates to create Langmuir-Blodgett films is detailed. A significant focus is placed on the array of advanced characterization techniques employed for probing monolayer structure *in situ* (e.g., Brewster Angle Microscopy, X-ray reflectivity/diffraction, vibrational spectroscopy) and analyzing the resultant LB films *ex situ*. The review further explores the expansion of L/LB techniques beyond classical amphiphiles to encompass polymers, nanoparticles, biomolecules, and other functional materials, highlighting their role in nanoarchitectonics for constructing complex supramolecular systems. Contemporary applications in molecular electronics, sensing, optics, energy storage, and biomimicry are discussed, showcasing the versatility of the technique. Finally, the survey addresses persistent challenges, including film stability, defect control, and scalability, comparing LB methods with alternative thin-film fabrication techniques, and outlines promising future directions focusing on novel materials, advanced interfacial studies, computational modeling, and integration into functional devices. The unique capability for precise molecular-level control ensures the continued relevance of L and LB films in fundamental research and specialized technological applications.

1 Introduction

Langmuir-Blodgett (LB) film technology represents a cornerstone technique for the fabrication of highly ordered molecular ultra-thin films, enabling precise control over molecular arrangement and film thickness at the nanometer scale. At its core, the method involves the formation of a Langmuir monolayer, a single layer of molecules, typically amphiphilic, organized at the air-water interface, followed by the controlled transfer of this monolayer onto a solid substrate. This process can be repeated to build multilayer structures with well-defined architecture and thickness. The resulting LB films, characterized by their high degree of structural order and precisely controlled thickness, have garnered significant attention across diverse scientific and technological domains. Their unique properties make them invaluable as model systems in fundamental research, exploring phenomena in surface science, complex fluids, and biophysics, and increasingly relevant for applications in materials science, molecular electronics, optics, sensing, and biomimicry. The ability to engineer molecular assemblies layer by layer provides unparalleled control over the chemical composition, molecular orientation, and overall supramolecular structure, establishing LB films as a key tool in nanotechnology and molecular engineering.

The historical roots of Langmuir and LB films trace back over a century, building upon initial observations of oil films spreading on water, famously documented by Benjamin Franklin in the 18th century. The scientific investigation began in earnest with Agnes Pockels, who, in the late 19th century, performed early surface film experiments using apparatus devised in her kitchen to study the behavior of surface films and quantify changes in surface tension. Her pioneering work laid the groundwork for Irving Langmuir, who systematically investigated the properties of monomolecular films at the air-water interface around 1917-1919. Langmuir introduced the concept of the Langmuir trough equipped with a movable barrier, allowing for the compression of the monolayer and the measurement of surface pressure as a function of the area available per molecule, leading to the characteristic surface pressure-area (π-A) isotherms [1]. Figure 1 illustrates a typical modern Langmuir trough setup, embodying the principles developed by Langmuir; it includes movable barriers for compressing the film [2] and a Wilhelmy plate connected to an electrobalance for precise measurement of the surface pressure, a key parameter established by Langmuir's work [3]. His fundamental insights into the phases and molecular organization within these

two-dimensional systems earned him the Nobel Prize in Chemistry in 1932 for his contributions to surface chemistry. Subsequently, Katharine Blodgett, working with Langmuir, developed the technique for transferring these Langmuir monolayers onto solid substrates around 1934. She demonstrated that stable, multilayer films could be built up by repeatedly dipping a substrate through the monolayer-covered interface, establishing the Langmuir-Blodgett deposition method [4, 5].

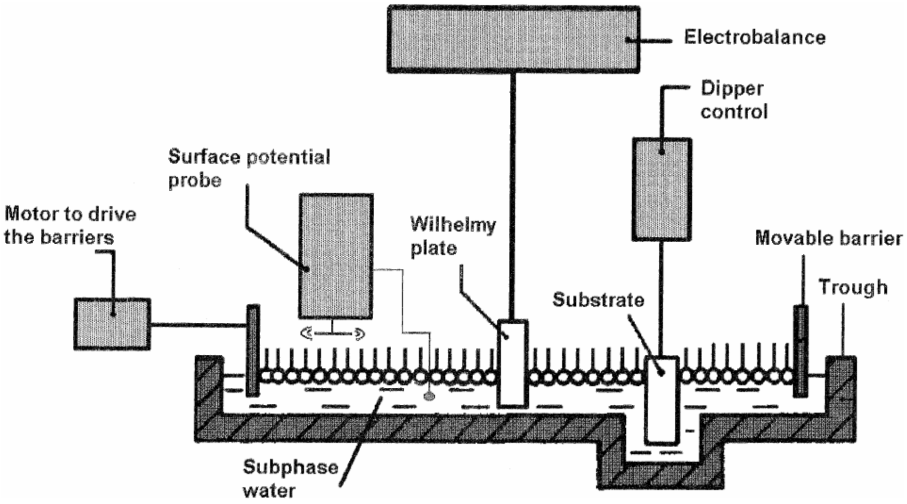

Figure 1: Schematic representation of a Langmuir trough that contains the Wilhelmy plate for measuring surface pressure using an electrobalance and a surface potential probe. Also shown is the dipper employed for transferring Langmuir monolayers onto a solid substrate to form Langmuir-Blodgett films. Adapted from Dynarowicz-Łatka, P., et al., 2001 [6].

This breakthrough opened the door to creating artificial structures with nanometer precision. In the 1960s, Hans Kuhn and his collaborators revitalized interest in LB films, envisioning their use in constructing complex, functional supramolecular systems by assembling layers of different molecules with specific functions, particularly for studying energy and electron transfer processes, thereby paving the way for concepts in molecular electronics. The field gained further momentum with the establishment of the International Conference on Ordered Molecular Films (ICOMF) in 1983 (initially focused on LB films, later broadening scope), fostering rapid development and interdisciplinary collaboration.

The fundamental principle underlying the formation of Langmuir monolayers relies on the behavior of amphiphilic molecules at the air-water interface [7]. These molecules possess distinct hydrophilic (water-loving) and hydrophobic (water-repelling) parts, typically a polar head group and a long nonpolar tail (e.g., a hydrocarbon chain), respectively. When dissolved in a volatile, water-immiscible organic solvent and carefully dispensed onto the surface of an aqueous subphase (usually pure water) within a Langmuir trough, the solvent spreads and evaporates, leaving the amphiphilic molecules at the interface. Due to their dual nature, the molecules spontaneously orient themselves: the hydrophilic heads immerse in the water, while the hydrophobic tails project upwards into the air, minimizing unfavorable interactions. Initially, at large surface areas, the molecules form a disordered, two-dimensional 'gas' phase (Region (a) in Figure 2). As the available surface area is reduced by moving the barriers of the trough, the molecules are compressed, increasing the surface pressure (π, the reduction in surface tension relative to the pure subphase). This compression induces phase transitions within the monolayer, progressing through liquid-expanded and liquid-condensed phases (Region (b)) to a quasi-solid condensed state (Region (c)) where the molecules are densely packed and often highly oriented, typically with their long axes tilted relative to the surface normal [8, 9]. Further compression can lead to monolayer collapse (Region (d)) [10].

The π-A isotherm, as exemplified for stearic acid in Figure 2, records these phase transitions as changes in slope and distinct plateau regions, providing critical information about the monolayer's state and compressibility.

Once a stable, condensed Langmuir monolayer is formed at a desired surface pressure (typically in the condensed phase), it can be transferred onto a solid substrate using the LB technique, as illustrated schematically in Figure 3. The figure shows the key steps: (a) spreading the amphiphilic solution onto the water surface, (b) compressing the resulting monolayer with barriers to achieve the desired packing density and surface pressure, and (c) transferring the monolayer onto a solid substrate, usually by vertical dipping. The most common method involves vertically dipping a hydrophilic or hydrophobic substrate through the monolayer. For a hydrophilic substrate, the first layer is typically transferred during the upstroke as the polar head groups adhere to the substrate surface. Subsequent layers are added during both the downstroke (hydrophobic tails adhering to the previously deposited hydrophobic tails) and the upstroke (hydrophilic heads adhering to the previously deposited hydrophilic heads), resulting in a Y-type depo-

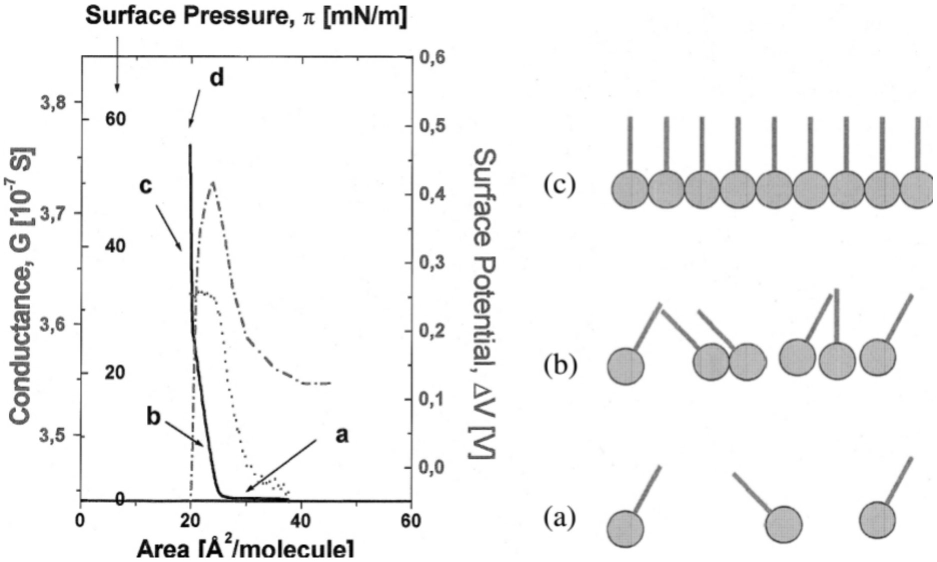

Figure 2: Surface pressure (π, solid line), surface potential (dotted line) and conductance (dashed line)—area (A) isotherms of stearic acid spread on water at 20°C. The π-A isotherm illustrates the various phases of the Langmuir monolayer during compression: (a) gaseous phase (large area per molecule, low pressure), (b) liquid-condensed phase (intermediate pressure, increasing order), (c) condensed phase (steep pressure increase, densely packed molecules), and (d) collapse (film buckles or forms multilayers). Schematic molecular arrangements corresponding to these phases are shown on the right. The figure exemplifies how the macroscopic measurement of surface pressure reflects the microscopic organization of molecules at the interface. Adapted from Dynarowicz-Łatka, P., et al., 2001 [6].

sition (shown simplified in Figure 3(c)), which creates a centrosymmetric multilayer structure with a bilayer as the repeating unit. Variations include X-type (deposition only on downstroke) and Z-type (deposition only on upstroke) transfer; however, structural studies have shown that films deposited via X- or Z-type methods often rearrange into the more energetically favorable bilayer (Y-type) structure during or after deposition [11, 12]. The Langmuir-Schaefer (LS) method offers an alternative, involving horizontal contact of the substrate with the monolayer for transfer. The success and quality of the transfer are often monitored by the transfer ratio, defined as the decrease in monolayer area on the water surface divided by the surface area

of the substrate coated. A transfer ratio close to unity indicates successful deposition. This layer-by-layer assembly allows for the creation of complex heterostructures with alternating layers of different materials, enabling the design of sophisticated functional devices [13, 14, 15].

This survey aims to provide a comprehensive overview of the fundamental principles, fabrication techniques, structural characteristics, and diverse applications of both Langmuir monolayers at the air-water interface and the transferred Langmuir-Blodgett films on solid substrates. We will explore the rich physics and chemistry of monolayers and the intricacies of the deposition process. Emphasis will be placed on the relationship between molecular architecture, film structure (both at the interface and on the substrate), and resulting properties. The survey will cover both traditional amphiphilic systems, such as fatty acids (the quintessential LB material) [16, 17], and more complex and non-traditional materials, including polymers [18, 19], nanoparticles [20], proteins [21, 22], and 2D materials like graphene and MOFs, which have significantly expanded the scope and potential of LB technology in recent years, often under the umbrella concept of "nanoarchitectonics" [23, 24, 25]. Furthermore, we will review the advanced characterization techniques employed to probe the structure and properties of these ultrathin films both *in situ* at the air-water interface [26, 27] and *ex situ* on solid supports [12, 28].

Finally, we will highlight key application areas, discuss current challenges, and outline future prospects for Langmuir monolayers and LB films in science and technology.

The structure of this survey is organized as follows: Section 2 delves into the fundamental aspects of Langmuir monolayers, including thermodynamics, phase behavior [17], and the details of the Langmuir-Blodgett and Langmuir-Schaefer deposition techniques [4, 13]. Section 3 provides an overview of the essential physicochemical characterization techniques used to study Langmuir monolayers *in situ* at the air-water interface, focusing on macroscopic measurements like surface pressure and surface potential isotherms [30, 31]. Sections 4 and 5 offer detailed discussions of microscopic [32, 33] and spectroscopic methods [34, 35], respectively, which provide crucial molecular-level information about monolayer structure, morphology, orientation, and chemical composition. Section 6 explores advanced topics and emerging trends, including the use of non-conventional materials (polymers [18], nanoparticles, biomolecules [36], 2D materials [25]), the concept of interfacial nanoarchitectonics [24], and specialized LB techniques [37]. Section

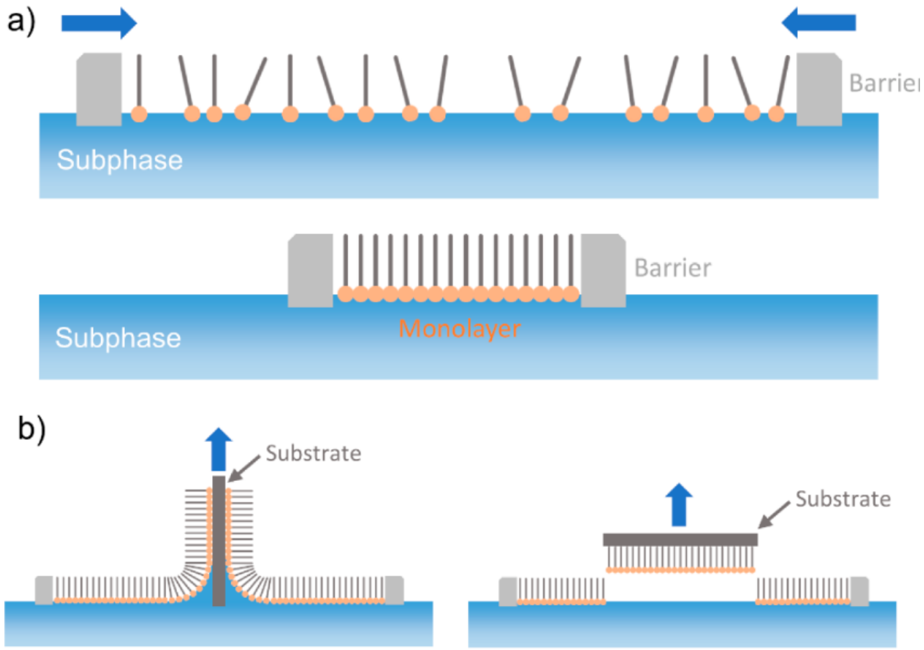

Figure 3: Schematic illustration of the Langmuir-Blodgett technique. (a) Amphiphilic molecules dissolved in a volatile solvent are spread onto the air-water interface. (b) The monolayer is compressed using movable barriers, forming an ordered Langmuir film at a specific surface pressure. (c) A solid substrate is vertically dipped and withdrawn through the interface to transfer the monolayer, building up a Langmuir-Blodgett film layer by layer (example shows simplified Y-type deposition). Adapted from Fang, C., et al., 2022 [29].

7 addresses the current challenges facing LB technology, such as film stability [11], defect control, and scalability, and discusses promising future directions and potential breakthroughs, particularly in areas like biomimetic systems and molecular electronics. Finally, Section 8 summarizes the key aspects covered in the survey and offers concluding remarks on the enduring importance and evolving landscape of Langmuir and Langmuir-Blodgett films [5, 38, 13].

2 Fundamentals of Langmuir and Langmuir-Blodgett Films

The study of Langmuir and Langmuir-Blodgett (LB) films is fundamentally rooted in the unique properties of amphiphilic molecules and their behaviour at interfaces. These molecules possess a dual chemical nature, characterised by a hydrophilic (water-loving) polar head group and a hydrophobic (water-repelling) non-polar tail, typically a long hydrocarbon chain such as those found in fatty acids or phospholipids. This amphiphilicity dictates their orientation and organisation at the air-water interface. When introduced to this interface, the hydrophilic head group seeks interaction with the aqueous subphase, anchoring the molecule, while the hydrophobic tail avoids the water, extending into the air phase. This spontaneous arrangement minimises the free energy of the system and is the basis for the formation of monomolecular layers, known as Langmuir monolayers.

The creation of a Langmuir monolayer typically begins by dissolving the amphiphilic substance in a volatile, water-immiscible organic solvent, such as chloroform or benzene. A precise volume of this dilute solution is carefully deposited onto the surface of an aqueous subphase contained within a specialised apparatus called a Langmuir trough [2]. The trough, often constructed from hydrophobic materials like Teflon to prevent wetting and ensure cleanliness, provides a defined area for the monolayer. For spontaneous spreading to occur, the solvent must possess a positive spreading coefficient, $S_s = \gamma_{w/air} - \gamma_{o/air} - \gamma_{w/o}$, where γ represents the interfacial tensions between water/air, organic solvent/air, and water/organic solvent, respectively. A positive S_s indicates that covering the water surface with the solvent lowers the overall interfacial energy, driving the spreading process. As the volatile solvent evaporates over a few minutes, the amphiphilic molecules remain, dispersed across the available water surface. Initially, at large surface areas per molecule, the amphiphiles behave like a two-dimensional gas, with minimal interaction between molecules. The hydrophilic heads are anchored by electrostatic interactions (ion-dipole, dipole-dipole, including hydrogen bonds) with the water, a process akin to physisorption. The hydrophobic tails experience repulsive forces from the water but attractive van der Waals forces amongst themselves, which favour ordering upon compression. These intermolecular forces are generally weak enough initially to permit spontaneous spreading rather than immediate aggregation. Maintaining stringent

experimental conditions is paramount for reproducible results, as highlighted by Dynarowicz-Latka et al. (2001). This includes using highly purified amphiphiles, spreading solvents, and subphase water (typically ultrapure), meticulous cleaning of the trough and barriers, accurate determination of the amount of material spread (accounting for any filtration losses), and operating in a clean, vibration-isolated environment with precise temperature control [39].

The primary tool for characterising the thermodynamic state and phase behaviour of Langmuir monolayers is the surface pressure-area (Π-A) isotherm. This measurement plots the surface pressure (Π) as a function of the average area available per molecule (A) at a constant temperature. Surface pressure is defined as the reduction in surface tension of the pure subphase (γ_0) caused by the presence of the monolayer (γ), i.e., $\Pi = \gamma_0 - \gamma$. It represents the two-dimensional analogue of pressure in a three-dimensional system and is typically measured in mN/m. The most common method for measuring surface tension (and thus surface pressure) is the Wilhelmy plate method [39, 40]. A thin plate, often made of platinum or hydrophilic filter paper to ensure a zero contact angle, is suspended from a sensitive electrobalance and partially immersed through the interface. The balance measures the downward force exerted by surface tension acting on the wetted perimeter of the plate. As noted by Dynarowicz-Latka et al. (2001), this provides an absolute measurement of surface tension/pressure. However, it can be susceptible to artefacts, especially with rigid monolayers, due to potential changes in contact angle if monolayer material adsorbs onto the plate or due to plate movement [3, 41]. Using fresh filter paper for each experiment can mitigate some issues. An alternative, the Langmuir balance, measures the *differential* surface pressure across a floating barrier separating the monolayer-covered surface from a clean water surface.

As the monolayer is compressed by moving barriers across the trough, reducing the available area per molecule, it typically transitions through several distinct phases, analogous to the gas, liquid, and solid phases of bulk matter [42]. These transitions are often indicated by changes in the slope (compressibility) or plateaus in the Π-A isotherm, as schematically illustrated in Figure 4. At very large areas (right side of the isotherm), the monolayer exists in a gaseous (G) phase, with molecules far apart and interacting weakly, resulting in near-zero surface pressure. Upon compression, it may enter a liquid-expanded (LE) phase, characterised by higher molecular density but still significant molecular motion and disorder in the alkyl tails; this region

shows a gradual increase in pressure with decreasing area. Further compression leads to a liquid-condensed (LC) phase, where molecules are more closely packed and tilted, exhibiting short-range positional order and potentially long-range orientational order (hexatic phases) [43]. The transition between LE and LC phases can appear as a distinct change in slope (a kink) or as a plateau region where pressure remains relatively constant over a range of areas, indicating a first-order phase transition [44]. Continued compression results in a solid (S) or condensed phase, where molecules are tightly packed, often in a near-vertical orientation, forming a two-dimensional crystalline or quasi-crystalline structure with high rigidity, reflected by a steep increase in pressure [45, 46]. The specific phases observed and the transition pressures depend critically on the molecular structure, temperature, subphase pH [47], and ionic strength, which affect head group interactions and tail packing. Beyond the solid phase, further compression leads to monolayer collapse, where the 2D structure breaks down, forming 3D aggregates, bilayers, or crystallites [10], often indicated by a plateau or drop in surface pressure at a characteristic collapse pressure (Π_c).

The stability of the monolayer up to its collapse pressure is crucial for subsequent applications like LB deposition.

The Langmuir-Blodgett (LB) technique, pioneered by Irving Langmuir and Katharine Blodgett, allows for the transfer of these organised Langmuir monolayers from the air-water interface onto a solid substrate. The process is illustrated schematically in Figure 5. Initially, the amphiphilic molecules are spread on the water surface to form the Langmuir monolayer (Panel a). The most common transfer method involves vertically dipping the substrate through the monolayer, which is held at a constant surface pressure (typically in a condensed phase) using the trough's feedback-controlled barriers [2] (Panel b shows the initial immersion/upstroke for a hydrophilic substrate). For a hydrophilic substrate, the first layer is typically deposited during the upstroke, with the polar head groups adhering to the substrate surface. Subsequent layers are added during both downstrokes (hydrophobic tails adhering to the previously deposited tails, Panel c) and upstrokes (hydrophilic heads adhering to previously deposited heads, Panel d). This alternating deposition results in a Y-type multilayer film, where the fundamental structural unit is a bilayer with head-to-head and tail-to-tail arrangement. The orientation depends on the stroke direction: molecules deposited on the upstroke have headgroups towards the substrate, while those deposited on the downstroke have tails towards the substrate. Less commonly, deposition might

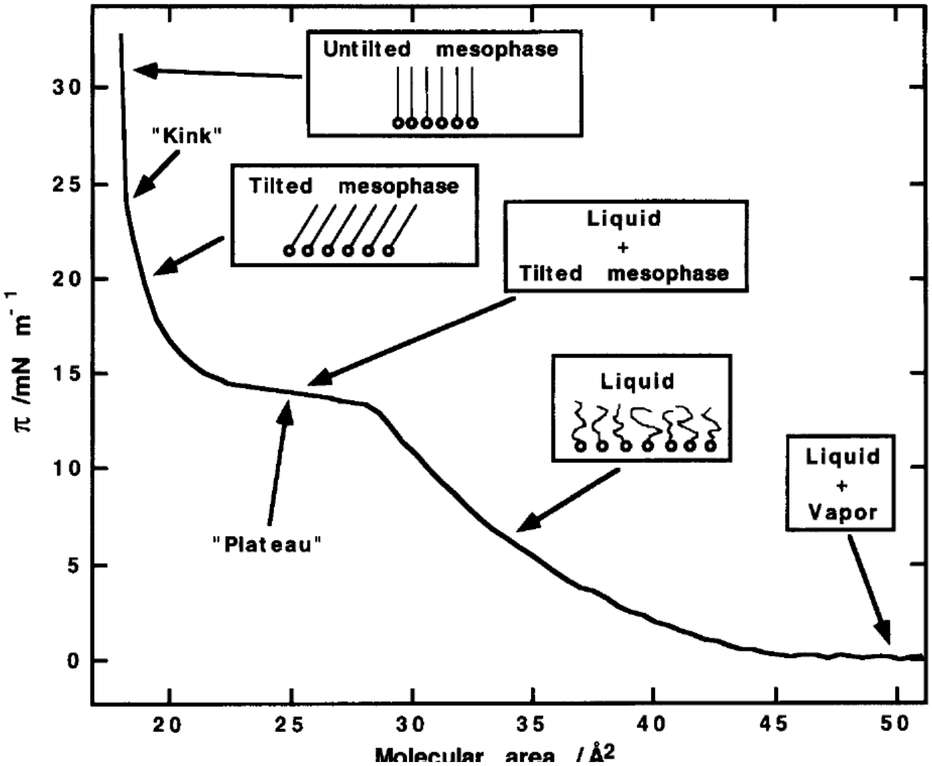

Figure 4: Schematic representation of a typical surface pressure-area (Π-A) isotherm for a Langmuir monolayer, illustrating the common phases: Gaseous (G), Liquid-Expanded (LE), Liquid-Condensed (LC), and Solid (S), leading to collapse. Adapted from Schwartz, D., 1997 [48].

occur only on the downstroke (X-type) or only on the upstroke (Z-type). However, even films prepared by X- or Z-type deposition often rearrange into the more thermodynamically stable Y-type bilayer structure [11, 49]. The quality and efficiency of the transfer are quantified by the transfer ratio (TR), defined as the ratio of the decrease in monolayer area on the trough to the area of the substrate coated during a stroke. An ideal transfer yields TR = 1.

Successful transfer depends on numerous factors, including the amphiphile's structure, subphase conditions (pH, ions which can act as bridges), deposition pressure, substrate properties (hydrophilic/hydrophobic), and dipping speed.

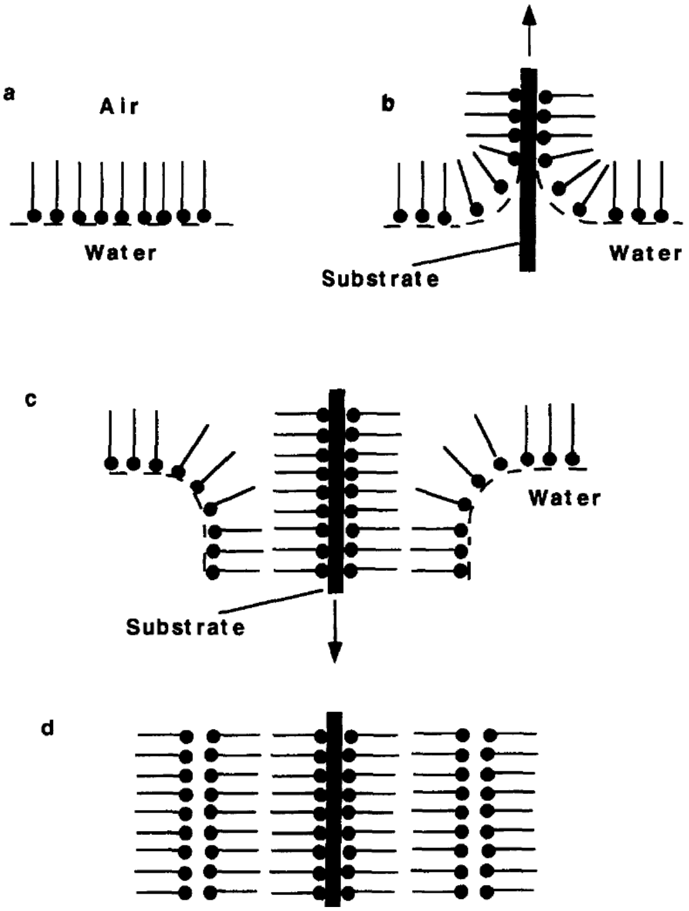

Figure 5: Conventional schematic of the LB technique using a hydrophilic substrate. In the first step (a), a suitable amphiphilic molecule is dissolved in a volatile solvent that is then spread at the air—water interface to form a Langmuir monolayer. With a barrier, the area of the trough can be altered to change the local density of the molecules, and usually the local organization and order as well. To form the LB film (b), a substrate is passed through the interface a given number of times, with each pass adding another monolayer to the LB film with alternating molecular orientations (c) and (d). Adapted from Schwartz, D., 1997 [48].

An alternative transfer method is the Langmuir-Schaefer (LS) technique, which involves horizontally touching the substrate to the monolayer surface. As mentioned by Oliveira et al. (2022) and Fang et al. (2021), this method is

often preferred for transferring rigid monolayers that might be disrupted by vertical dipping [50], or when specific film architectures are desired, such as avoiding the bilayer structure inherent in Y-type deposition [12]. Multilayers can also be built using repeated LS transfers [13].

Monolayer stability and potential hysteresis effects are critical considerations. Stability refers to the ability of the monolayer to maintain its structure and surface pressure over time at a given area, or maintain its area at a constant surface pressure. Instability can arise from factors like dissolution of the amphiphile into the subphase, evaporation from the surface (for more volatile components), chemical degradation, or slow rearrangement into 3D collapsed structures even below the nominal collapse pressure [10, 51, 52]. Stability is assessed by monitoring the change in surface pressure at constant area or the change in area at constant surface pressure over time. Hysteresis is often observed when comparing the Π-A isotherm recorded during compression with that recorded during subsequent expansion [53, 54, 55]. A hysteresis loop, where the expansion curve lies at lower pressures than the compression curve for a given area, can indicate slow relaxation processes, molecular reorganisation within the film [56], irreversible collapse, or loss of material to the subphase or through leakage past barriers. Understanding these aspects is crucial for selecting appropriate deposition conditions and ensuring the quality and reproducibility of the final LB films. The choice of deposition pressure, often guided by the isotherm, must balance achieving a densely packed, stable monolayer with avoiding proximity to the collapse region where defects are more likely.

3 Physicochemical Characterization Techniques for Langmuir Monolayers

Beyond the fundamental surface pressure-area isotherms discussed previously, a suite of complementary physicochemical techniques provides deeper insights into the structure, properties, and behavior of Langmuir monolayers at the air-water interface. These methods probe electrical characteristics, molecular organization [28, 57], and dynamic processes within the monolayer, offering a more comprehensive understanding essential for both fundamental studies and optimizing conditions for Langmuir-Blodgett film deposition. Among the most established electrical characterization methods are surface

potential measurements [58, 59, 60], conductance measurements [61], and Maxwell displacement current analysis [62, 63, 64, 65]. Each technique offers unique perspectives on the monolayer's state, although often yielding complementary information. The complexity of these systems, particularly when dealing with non-classical amphiphiles or multicomponent mixtures, frequently necessitates the combined application of several techniques to unravel the intricate interplay of molecular interactions, orientation, and phase behavior at the interface.

3.1 Surface Potential Measurements

The surface potential (ΔV) of a Langmuir monolayer is defined as the difference in electrical potential between the monolayer-covered subphase surface and the clean subphase surface [6, 58]. This potential arises from the collective contribution of oriented permanent electric dipoles of the film-forming molecules [60, 66], the reorientation of water molecules in the subphase induced by the monolayer's presence, and, for ionised monolayers, the electrical double layer formed between charged headgroups and counterions in the subphase. Measuring ΔV provides valuable information about molecular orientation [67], packing density, phase transitions, degree of ionisation, and interactions with subphase species [68].

Two primary methods are employed for measuring surface potential [6, 69]. The vibrating capacitor technique, a modification of the Kelvin probe method, measures the potential difference between the monolayer-covered surface and a reference electrode plate positioned parallel to it in the air above. One plate (typically the reference electrode) is made to vibrate perpendicularly to the interface, inducing an alternating current if a potential difference exists. In practice, a null method is often applied: an external bias voltage is adjusted until this alternating current is nullified. The required bias voltage directly corresponds to the contact potential difference, which is the surface potential ΔV. The second common method utilises an ionising probe, typically containing a weak radioactive source (e.g., Americium-241), placed above the air-water interface. This probe ionises the air gap between itself and the monolayer surface, rendering the air conductive. A reference electrode is placed within the subphase, and the potential difference is measured using a high-impedance electrometer. Similar to the vibrating capacitor, a null method can be employed where an external voltage cancels the potential difference, yielding ΔV. Both techniques are sensitive to experimental

artefacts, such as changes in the reference potential over time (mitigated by using an inert atmosphere) and stray capacitance effects (minimised by proper electrical shielding).

Theoretical interpretation of surface potential data often begins with the Helmholtz model, which treats the monolayer as a parallel plate capacitor consisting of a sheet of uniformly distributed dipoles [70]. The surface potential is given by:

$$\Delta V = \frac{\mu_\perp}{\epsilon_0 \epsilon_r A} \tag{1}$$

where $\mu_\perp$ is the average component of the molecular dipole moment perpendicular to the surface, A is the area per molecule, ϵ_0 is the permittivity of free space, and ϵ_r is the relative dielectric constant of the monolayer medium. Significant debate exists regarding the appropriate value for ϵ_r [70, 71]. This stems from the difficulty in applying a macroscopic dielectric constant to a molecularly thin film where the local molecular environment and screening effects differ significantly from the bulk material. Values ranging from 1 (for gaseous films or isolated dipoles) to values between 2 (typical for hydrocarbons) and 10 have been suggested or used [70, 72].

More sophisticated models refine the Helmholtz approach by considering distinct contributions from different parts of the system. Davies and Rideal proposed decomposing $\mu_\perp$ into contributions from the hydrophobic tail (e.g., terminal methyl group, μ_3), the polar head group (μ_2), and the reoriented water molecules beneath the monolayer (μ_1) [6]. Since μ_1 is difficult to determine independently and depends on the head group, it is often combined with μ_2.

Building on this, Vogel and Möbius, and later Demchak and Fort, developed multi-layer capacitor models (illustrated schematically in Figure 6) [6]. The Vogel-Möbius model treats the monolayer as a two-layer capacitor (hydrophobic tails and headgroup/water region), while the Demchak-Fort model considers three distinct layers (tails, headgroups, and oriented water). These models assign different dielectric constants to each layer, aiming for a more physically realistic description. For example, the Vogel-Möbius model expresses ΔV as:

$$\Delta V = \frac{1}{\epsilon_0} \left(\frac{\mu_{\perp 1}}{A \epsilon_{r1}} + \frac{\mu_{\perp 2}}{A \epsilon_{r2}} \right) \tag{2}$$

where indices 1 and 2 refer to the different layers (e.g., headgroup/water and tail regions) with their respective perpendicular dipole moment components and dielectric constants [6].

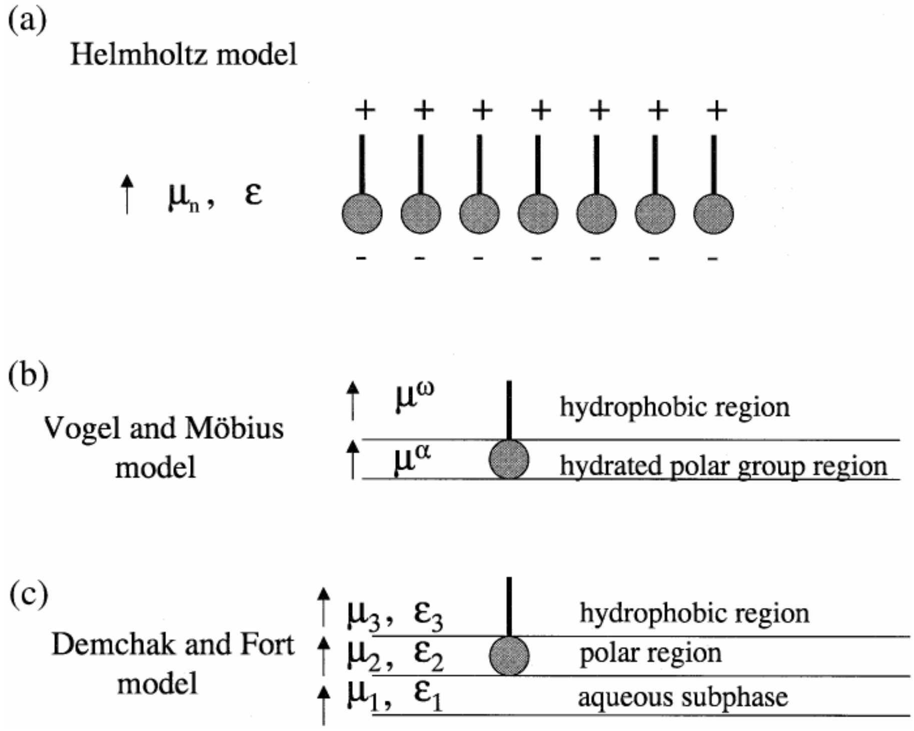

Figure 6: Models to explain the monolayer surface potential. The Helmholtz model assumes simply a layer of oriented dipoles in a medium of dielectric constant ¢. In the more sophisticated Vogel—Möbius, and Demchak-Fort models, the monolayer is treated as a two- or three-layer capacitor, respectively. Adapted from Dynarowicz-Łatka, P., et al., 2001 [6].

Figure 7 illustrates how these models can be used to theoretically decompose the total measured surface potential (ΔV) into its constituent contributions as a function of molecular area (A). For a molecule like stearic acid, the figure shows the calculated contributions attributed to the reorientation of water molecules (V_1), the dipole moment of the head group (V_2), the dipole moment of the tail group (V_3), and potentially the double layer potential (ψ_0, denoted V_4 in the figure's context, which becomes significant for ionised films), based on assumptions within models like the Vogel-Möbius or Demchak-Fort frameworks [6].

Surface potential isotherms (ΔV vs. A) are highly sensitive to molecular orientation. Changes in the slope or value of ΔV during compression often

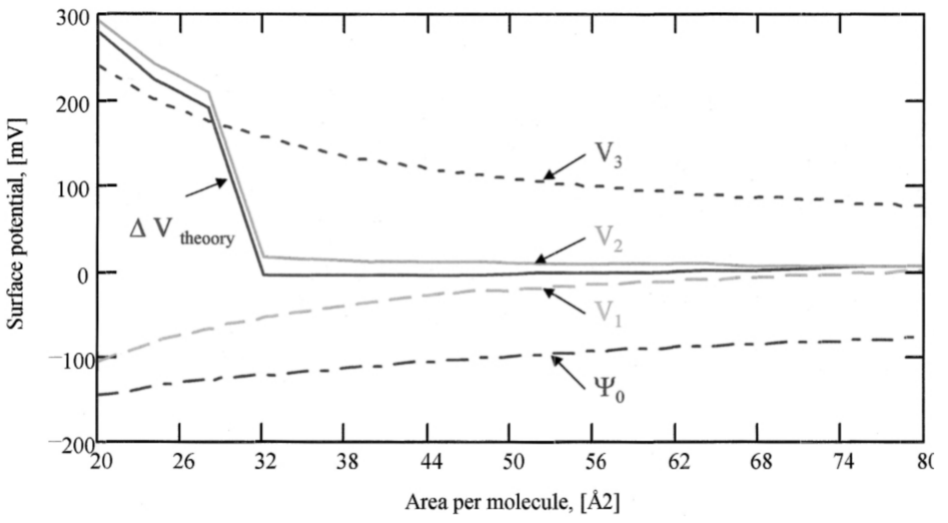

Figure 7: Theoretical surface potential-area (ΔV-A) isotherm for a stearic acid monolayer on pure water subphase. This illustrates the decomposition of the total measured potential (ΔV) into theoretical contributions from different molecular parts and the interface: V_1 (water reorientation), V_2 (head-group dipole), V_3 (tail group dipole), and V_4 (double layer potential ψ_0, relevant for ionised films, though shown conceptually here for a non-ionised example based on the model's framework). Adapted from Dynarowicz-Łatka, P., et al., 2001 [6].

indicate transitions in molecular tilt or conformation [6, 67]. For instance, transitions from horizontal to vertical orientations of alkyl chains or from face-on to edge-on orientations of planar molecules like phthalocyanines have been inferred from $\Delta V - A$ isotherms [6]. The technique has also been used to monitor photoinduced cis-trans isomerisation of azo chromophores, where the different isomers possess distinct dipole moments and orientations [6, 73]. For complex systems like polymers, quantitative correlation with group dipole moments is challenging due to conformational randomness, but ΔV measurements still provide qualitative insights into aggregation, domain formation (indicated by non-zero ΔV at large areas), and the influence of subphase conditions (e.g., pH-induced protonation affecting the double-layer potential in polyaniline monolayers) [6].

For ionisable monolayers, ΔV measurements provide a means to estimate the degree of dissociation (α) and the surface pK value [6, 74]. The potential

contribution from the ionic double layer, ψ_0, can be related to the surface charge density ($\sigma = e\alpha/A$) using the Gouy-Chapman theory [6, 75]:

$$\psi_0 = \frac{2kT}{e}\sinh^{-1}\left(\frac{\sigma}{\sqrt{8\epsilon_0\epsilon_w nkT}}\right) \approx \frac{51.4}{z}\sinh^{-1}\left(\frac{136\alpha}{A\sqrt{c}}\right) \quad \text{(mV at 20°C)} \tag{3}$$

where k is the Boltzmann constant, T is temperature, e is the elementary charge, ϵ_w is the dielectric constant of water, n is the bulk ion concentration, z is the ion valency, A is in Å^2/molecule, and c is the molar concentration of a 1:1 electrolyte. Although the applicability of the Gouy-Chapman model to highly charged, condensed monolayers is debated [6], particularly because its assumptions (point charges, uniformly smeared surface charge, neglect of specific ion interactions and sizes) may break down at high surface charge densities or high ionic strengths, it is widely used for qualitative and semi-quantitative analysis. By measuring ΔV at different subphase pH values and salt concentrations, and relating the surface pH (pH

3.2 Conductance Measurements

The possibility of enhanced electrical conduction along the plane of a Langmuir monolayer, particularly proton conduction, has been a subject of investigation and debate for several decades [6, 61]. Early studies by Teissié and coworkers, using pH-sensitive fluorescent probes embedded in monolayers, suggested rapid lateral proton transport when monolayers were compressed below a critical area, coinciding with the onset of surface potential changes [77, 78]. They proposed that protons could move efficiently along hydrogen-bonded networks formed by the polar head groups and associated water molecules at the interface [6, 79].

Direct electrical measurements attempting to quantify this lateral conductance followed. Sakurai and Kawamura, using a small cuvette, and Taylor and colleagues, using a standard Langmuir trough, employed two parallel electrodes (often platinum foils) partially immersed in the subphase, spanning the monolayer-covered surface (see Figure 8a). By applying a DC voltage and measuring the current, they reported significant increases in conductance upon monolayer compression, particularly for fatty acids and phospholipids, seemingly supporting the proton hopping mechanism along hydrogen-bonded chains [79, 77, 80, 81]. The observation of conductance even in neutral lipid monolayers appeared to rule out simple transport via excess ions in the electrical double layer [82, 61]. Theoretical modeling studies

also lent support to the feasibility of such hydrogen-bond controlled proton hopping mechanisms [83, 84].

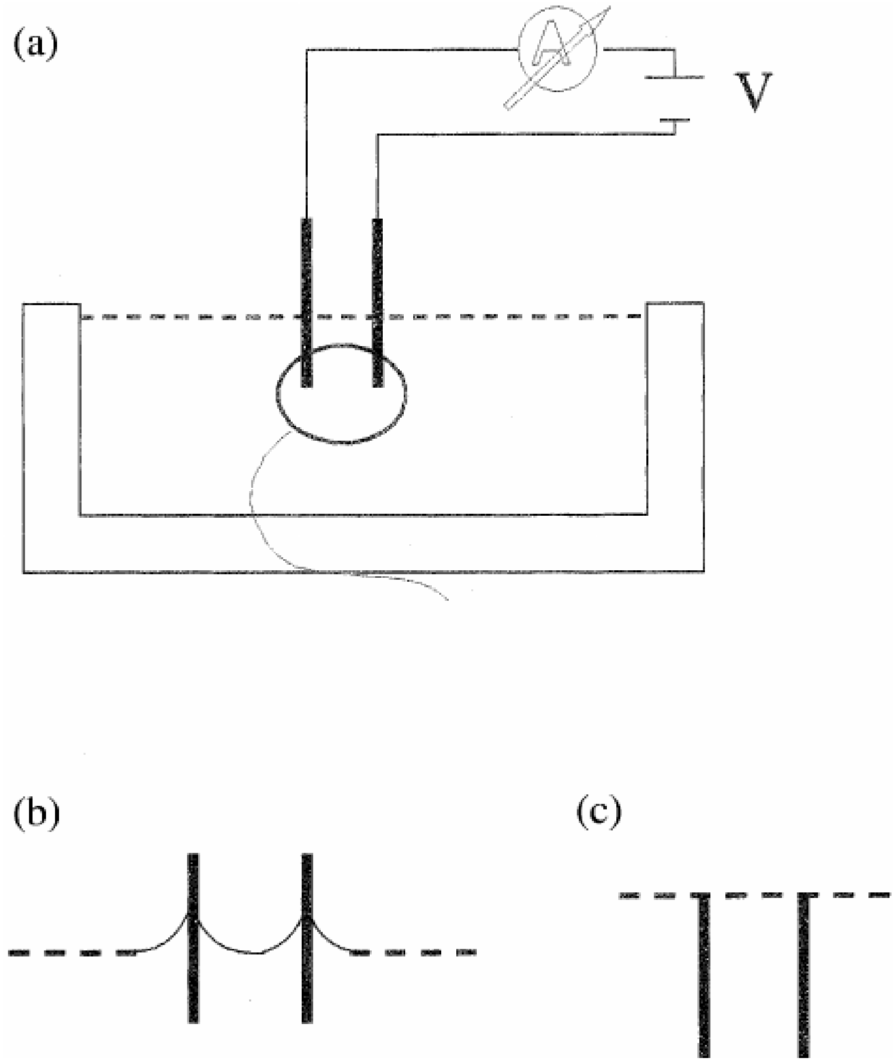

Figure 8: Experimental setup for measuring lateral conductance on a Langmuir monolayer. The meniscus effect is manifested with electrodes partially immersed in the subphase (a,b). In the new arrangement, proposed by Cavalli and Oliveira, the electrodes are fully immersed, thus eliminating the meniscus effect (c). Adapted from Dynarowicz-Łatka, P., et al., 2001 [6].

However, these findings were subsequently challenged [6]. Menger et al.,

and later Shapovalov and Il'ichev using AC and DC four-electrode techniques, failed to observe the reported enhanced conductivity for stearic acid and phospholipid monolayers [6]. Goryunov and Yupatov replicated earlier experiments and found that the sign of the conductance change near the critical area depended on whether the trough material was hydrophilic (glass) or hydrophobic (Teflon). They attributed these effects, particularly in small troughs, to changes in the meniscus shape around the electrodes and trough walls during compression (Figure 8b), which could alter the effective conduction path through the subphase, rather than indicating intrinsic monolayer conductance [6]. They noted that such meniscus effects might be less significant in the larger troughs used by Taylor's group [6].

The experimental challenges in reliably measuring the very small conductance changes associated with a single molecular layer are significant. Factors like electrode placement, meniscus effects, subphase purity, and potential artefacts from the measurement setup contribute to the difficulty in obtaining consistent and universally accepted results. Cavalli and Oliveira later addressed the meniscus issue by proposing a differential measurement system with fully immersed electrodes (Figure 8c), designed to eliminate artefacts related to water level changes near the electrodes during compression [6, 61]. The ongoing controversy surrounding lateral conductance measurements underscores the high sensitivity of experiments on monomolecular films to subtle artefacts related to the experimental setup and emphasizes the need for rigorous experimental design and cautious interpretation of results when probing transport properties in such confined geometries [85].

3.3 Maxwell Displacement Current (MDC) Measurements

The Maxwell displacement current (MDC) technique, pioneered by Iwamoto and coworkers, provides another avenue for probing the electrical properties and dynamic behavior of Langmuir monolayers [6]. This method measures the electrical current that flows between an electrode suspended in the air above the monolayer and another electrode immersed in the subphase when the monolayer's properties (e.g., area per molecule, molecular orientation) change over time [64, 62, 86]. The setup is similar to that used for surface potential measurements with an ionising probe, but instead of measuring a static potential, MDC measures the transient current generated by changes

in the electric displacement field across the air gap [87, 88].

The displacement current I is related to the rate of change of the total charge Q induced on the top electrode, which in turn depends on the perpendicular component of the molecular dipole moment $\mu_\perp$ and the number of molecules N under the electrode: $I = dQ/dt$. During monolayer compression or expansion at a constant rate, the number of molecules under the electrode changes ($dN/dt \neq 0$), and potentially their average orientation ($\mu_\perp$) also changes ($d\mu_\perp/dt \neq 0$). Thus, MDC is sensitive to variations in both molecular density and orientation over time. While the information derived from MDC is closely related to that obtained from surface potential measurements (as both depend fundamentally on $\mu_\perp$), MDC specifically measures the *rate of change* of the electric displacement. This makes it particularly sensitive to the *dynamics* of molecular reorientation [89, 90], phase transitions [62, 86], chemical reactions, and aggregation/disaggregation processes occurring within the monolayer during external perturbations like compression/expansion or photoisomerization [6, 91, 63].

MDC has been extensively used to investigate various phenomena in Langmuir films [6]. It is particularly sensitive to the early stages of molecular organization and phase transitions, sometimes revealing features not apparent in surface pressure isotherms, especially in the region of very low surface pressures before significant pressure develops. For example, MDC measurements on mesogenic liquid crystal monolayers (like cyanobiphenyls, nCBs) have been used to study orientational phase transitions (e.g., normal-director to tilted-director phases) and dielectric relaxation phenomena [92, 93, 94]. The dependence of MDC signals on compression rates can indicate non-equilibrium processes. By analysing MDC data using molecular models (e.g., rod-like or biaxial models), information about dielectric anisotropy and relaxation times as a function of molecular area can be extracted [95, 88, 64].

The technique has also proven useful in studying photoinduced processes, such as the cis-trans isomerisation of azobenzene derivatives in monolayers, where the change in molecular dipole orientation upon photo-switching generates a measurable displacement current [96, 91, 97]. Furthermore, MDC has demonstrated sensitivity to chirality [98, 99, 100, 101]. For chiral phospholipids, the amplitude and sign of MDC peaks observed during phase transitions were found to depend on the molecular chirality, even when the surface pressure isotherms were identical [102, 103, 104, 105]. This effect was interpreted in terms of chiral-dependent molecular tilt or twist within the monolayer domains, highlighting MDC's potential for probing subtle structural

arrangements related to molecular stereochemistry at the air-water interface [106, 107, 108, 109].

4 Microscopic Investigation of Langmuir Films

Understanding the structure and morphology of Langmuir monolayers at the air-water interface is crucial for controlling their properties and optimizing their transfer onto solid substrates for Langmuir-Blodgett (LB) film fabrication. While thermodynamic measurements like surface pressure-area isotherms provide macroscopic information about phase transitions, microscopic techniques offer direct visualization of the film's organization at mesoscopic length scales. These methods reveal details about domain formation, phase coexistence, film homogeneity, defects, and dynamic processes occurring within the monolayer. Early attempts using electron microscopy provided limited insights; these studies primarily showed film inhomogeneity at low pressures and large, seemingly homogeneous patches at high pressures. Significant limitations included the inability to perform in-situ observations of the dynamic air-water interface due to vacuum requirements and potential artifacts introduced during the necessary sample transfer and preparation processes. Modern microscopic techniques, particularly fluorescence microscopy and Brewster angle microscopy (BAM), overcome many of these limitations and offer significantly more detailed in-situ information about the monolayer structure.

4.1 Fluorescence Microscopy

Fluorescence microscopy visualizes Langmuir monolayer morphology by incorporating a small amount (typically less than 1 mol%) of a fluorescent probe molecule into the monolayer. When the monolayer is illuminated with excitation light of an appropriate wavelength, the probe molecules fluoresce, and the emitted light is collected through a microscope objective to form an image of the interface. The key principle behind contrast generation in fluorescence microscopy of Langmuir films is the differential partitioning or solubility of the fluorescent probe between different phases or domains within the monolayer. Often, the probe's fluorescence properties (intensity, lifetime, spectrum) are sensitive to its local environment, including polarity, viscosity, and molecular packing density.

A classic example is the study of phospholipid monolayers, such as dipalmitoylphosphatidylcholine (DPPC). In DPPC monolayers, the transition between the liquid-expanded (LE) and liquid-condensed (LC) phases is clearly visualized using specific fluorescent probes [110, 111]. Some probes are preferentially soluble in the more fluid LE phase, leading to a bright, relatively homogeneous image in this state. Upon compression into the LE-LC coexistence region, domains of the more ordered LC phase nucleate and grow. If the probe is insoluble or significantly less soluble in the LC phase, these domains appear dark against the bright background of the surrounding LE phase, as observed for DPPC where the dye is squeezed out from the LC state. Conversely, probes soluble in the LC phase would show bright domains on a dark background. The shapes of these domains can vary significantly depending on the material, temperature, compression rate, and intermolecular interactions, ranging from circular to complex dendritic or spiral patterns [112, 113, 114]. The evolution of domain size and shape during compression or relaxation provides insights into line tension, dipole-dipole interactions, and phase transition kinetics [115].

Fluorescence microscopy is not limited to single-component systems and has been instrumental in studying miscibility and phase separation in mixed monolayers [116, 117, 118]. It allows investigation of complex interactions in biologically relevant systems, such as mixed lipid monolayers mimicking cell membranes [119, 120] or monolayers containing proteins or peptides [121, 122]. The technique has also visualized the squeezing-out of material upon high compression.

Furthermore, fluorescence techniques can probe molecular recognition events at the interface [123, 124, 125]. For example, studies involving amphiphilic nucleobases used specific probes like octadecyl rhodamine B (C_{18}RhB) and octadecylacridine orange (C_{18}AO). C_{18}RhB acted as a negative stain, revealing dark crystalline domains in a mixed octadecyladenine/octadecylthymine monolayer from which it was excluded. C_{18}AO, capable of intercalating into base pairs, provided evidence for Watson-Crick pairing through specific fluorescence emission changes. The complexation of guanosine from the subphase to an octadecylcytosine monolayer was also inferred using C_{18}AO intercalation [126, 127, 128].

Analysis of fluorescence intensity and spectra can provide additional information. Changes in fluorescence intensity during compression can reflect alterations in the probe's environment or its orientation relative to the interface plane and the polarization of the excitation/emission light. However,

interpreting intensity changes requires caution, as factors like probe aggregation (e.g., excimer formation), energy transfer, fluorescence quenching (e.g., by oxygen), and potential probe segregation can complicate the analysis. Excimer formation, detected by characteristic spectral shifts, can indicate intermolecular interactions and aggregation, as seen in studies with 12-(1-pyrenyl)dodecanoic acid (PDA) [129, 130]. Measurements are often performed under a nitrogen atmosphere to minimize oxygen quenching, although experiments in air can sometimes provide information about the shielding of the chromophore by the monolayer structure.

Fluorescence recovery after photobleaching (FRAP) is a powerful extension of the technique used to measure the lateral diffusion coefficients of the probe molecules within the monolayer [131]. By photobleaching a small spot and monitoring the recovery of fluorescence as unbleached probes diffuse into the area, information about the fluidity of different monolayer phases can be obtained. Studies on phospholipid monolayers deposited on substrates have shown significant decreases in diffusion coefficients upon transitioning from fluid/disordered to more ordered phases, confirming the changes in molecular mobility associated with phase transitions [115, 119]. For instance, diffusion coefficients can change sharply, spanning ranges from approximately 10^{-10} cm^2s^{-1} in ordered states to 10^{-8} cm^2s^{-1} or higher in disordered states at elevated temperatures, reflecting the transition from solid-like to fluid-like behavior within the monolayer, as detailed in studies referenced by Schwartz.

Despite its utility, the primary limitation of fluorescence microscopy is the requirement for extrinsic probes. These probes, even at low concentrations, can potentially perturb the monolayer structure, phase behavior, or domain morphology, leading to artifacts [110, 132]. Careful selection of probes and comparison with probe-less techniques are often necessary to validate the observations.

4.2 Brewster Angle Microscopy (BAM)

Brewster angle microscopy (BAM) provides a powerful alternative for visualizing Langmuir monolayers without the need for fluorescent probes, thus circumventing the potential artifacts associated with probe incorporation. The technique is based on the principles of reflection of polarized light at an interface. When p-polarized light (light polarized parallel to the plane of incidence) strikes the interface between two dielectric media (like air and water) at a specific angle, known as the Brewster angle (θ_B), there is no

reflection. For the air-water interface, θ_B is approximately 53.1°. The introduction of a thin film, such as a Langmuir monolayer, onto the water surface alters the local refractive index and disrupts the Brewster angle condition. This results in a non-zero reflectivity, allowing the monolayer to be imaged against a dark background.

The intensity of the reflected light in BAM depends on several factors, including the thickness of the monolayer, the surface density of the molecules, the molecular orientation (specifically the tilt angle of anisotropic molecules relative to the surface normal) [133], and the difference in refractive index between the monolayer and the subphase. Because the technique uses polarized light, it is sensitive to optical anisotropy within the film, which can arise from the collective tilt and ordering of the amphiphilic molecules [57]. This sensitivity allows BAM to provide information not only on the presence and morphology of the monolayer but also on the average molecular orientation within different domains or phases [134, 135].

BAM is highly effective in probing the two-dimensional organization of Langmuir films at mesoscopic scales (micrometers to millimeters) [135]. It allows direct visualization of domain formation during phase transitions, similar to fluorescence microscopy [110, 132], but without probes [134]. For example, the LE-LC phase transition in fatty acids or phospholipids can be observed as the growth of brighter domains (corresponding to the denser, thicker LC phase) within a darker background (the LE phase). The technique can clearly distinguish domain shapes, sizes, and their evolution under compression or shear. Dendritic domain shapes, for instance, are often observed [136] and indicate anisotropic growth kinetics, suggesting that molecular cohesion is favored along specific directions within the domain. BAM can also visualize film heterogeneity, defects such as cracks or holes, and the process of monolayer collapse, which often manifests as the formation of three-dimensional structures, folds, or fractures appearing as distinct bright features or regions with altered texture [10].

Quantitative analysis of BAM images can provide information on the relative thickness of different monolayer domains or phases, as the reflectivity is related to film thickness and refractive index [135]. By analyzing the reflectivity contrast, researchers have estimated relative thickness changes during compression, correlating them with phase transitions and collapse points, as demonstrated for food emulsifiers like monopalmitin and monoolein. Coupling BAM with ellipsometry allows for more precise determination of absolute film thickness and optical properties within specific domains [137].

BAM observations often correlate well with data obtained from surface pressure-area isotherms and surface potential measurements [67, 59]. For example, a seemingly 'solid' Langmuir film characterized by a steep rise in the isotherm might be revealed by BAM to contain significant defects or holes, consistent with noisy surface potential readings, indicating molecular aggregation rather than a perfect crystalline lattice, as observed for certain radical-bearing conjugated molecules. Conversely, a uniform BAM image is typically observed for liquid-expanded films exhibiting a monotonic increase in surface pressure, suggesting weaker intermolecular interactions and a more homogeneous structure [135, 134].

The technique has been applied to a wide range of materials, including classical amphiphiles like fatty acids and phospholipids, polymers, nanoparticles spread at the interface, proteins, and complex mixtures. Studies on amphiphilic Eu(III) complexes showed how morphology and collapse behavior depend on molecular structure (alkyl chain length, head group hydrophilicity). BAM has also been used to monitor in-situ reactions at the air-water interface, such as the photoinduced cycloaddition of a p-phenylenediacrylic acid derivative, where changes in reflectivity were correlated with the loss of conjugation upon reaction. Investigations of charge-transfer complexes mixed with arachidic acid and phase diagrams of various fatty acids at different temperatures have also benefited from BAM analysis [135, 134, 133].

Figure 9 illustrates the versatility of BAM in visualizing diverse Langmuir film structures without probes [135, 134]. Image (a) shows the characteristic coexistence of liquid-expanded (darker background) and liquid-condensed (brighter, distinct domains) phases in a DPPC monolayer [110, 119], highlighting BAM's ability to resolve phase transitions. Image (b) reveals the textured morphology of a poly(2-chloride-p-phenylenevinylene) film, demonstrating its utility for characterizing polymer monolayers. Image (c) visualizes a more complex biological assembly, specifically immortalized endothelial cells functionalized with antibodies at the interface, showcasing BAM's applicability to biological systems and adsorbed materials. These examples underscore BAM's power to provide direct morphological insights across different material classes at the air-water interface.

In essence, BAM complements thermodynamic measurements by providing direct visual evidence of monolayer organization, phase behavior [135, 134, 139], and defects [140, 141], which is crucial for understanding the fundamental properties of Langmuir films and optimizing their use in LB deposition and other applications [13, 38].

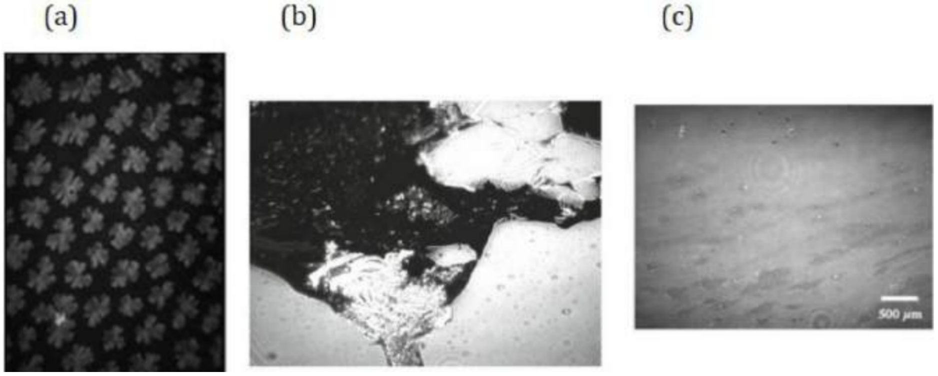

Figure 9: BAM images for a monolayer of DPPC (a), poly(2-chloride-p-phenylenevinylene) (b), and immortalized rabbit-aorta-derived endothelial cells with monoclonal antibody trastuzumab (c). Adapted from Oliveira Jr., O., et al., 2022 [138].

5 Spectroscopic Characterization of Langmuir Films

Spectroscopic techniques provide invaluable molecular-level information about the composition, structure, orientation, and interactions within Langmuir monolayers at the air-water interface. While the monomolecular nature of these films presents challenges due to inherently weak signals, advancements in instrumentation and methodology have enabled detailed 'in situ' characterization using various forms of spectroscopy, primarily operating in the ultraviolet-visible (UV-Vis), infrared (IR), and Raman scattering regimes. These methods are often used in conjunction with surface pressure and potential measurements to build a comprehensive understanding of monolayer behavior. Understanding these characteristics is crucial for optimizing the Langmuir film structure prior to its potential transfer onto solid substrates via Langmuir-Blodgett deposition.

5.1 UV-Vis Spectroscopy

The application of UV-Vis spectroscopy to Langmuir films is relatively straightforward compared to other techniques, principally because the aqueous subphase does not significantly absorb light in this spectral region. This mini-

mizes interference from the bulk liquid. However, the extremely small path length through the monolayer (typically nanometers) results in very weak absorption signals. Consequently, UV-Vis spectroscopy is most effective for monolayers composed of molecules containing strongly absorbing chromophores, such as dyes or porphyrins.

'In situ' UV-Vis measurements, often performed in reflection mode, allow for the direct monitoring of changes occurring within the monolayer during compression or reaction. The enhancement of reflection (ΔR) from the air-water interface due to the presence of a monolayer can be related to the monolayer's reflectivity (ρ) and absorption (A), often simplified for weakly reflecting monolayers where ΔR is approximately proportional to A [142]. For instance, the reflection intensity at the absorption maximum of a chromophore generally increases linearly with the surface density of molecules as the film is compressed, providing a spectroscopic correlate to the surface pressure-area isotherm. This was observed for mixed monolayers containing porphyrin, where the reflection at the Soret band maximum increased linearly with decreasing area per molecule, reflecting the increasing number of chromophores per unit area.

A key application of UV-Vis spectroscopy is the study of molecular aggregation within the Langmuir film [143, 144]. By comparing the spectrum of the monolayer to that of the constituent molecules dissolved in a dilute solution (where they exist as isolated monomers), shifts in the absorption maxima can reveal the formation of aggregates. A blue shift (hypsochromic shift) relative to the monomer absorption is typically indicative of H-aggregate formation (often associated with face-to-face stacking), while a red shift (bathochromic shift) suggests J-aggregate formation (often associated with head-to-tail arrangements) [145]. For example, studies on N-confused tetraphenylporphyrin mixed with methyl octadecanoate showed distinct spectral shifts upon complexation with halide ions in the subphase. A red shift observed with iodide ions indicated J-aggregation, whereas blue shifts in water or other salt solutions pointed to H-aggregation, demonstrating the specific role of the anion in directing the aggregation pathway. Similarly, the complexation between azobenzene-containing amphiphiles and cellulose polyelectrolytes has been monitored using UV-Vis reflection spectroscopy [146, 147, 148].

Furthermore, UV-Vis spectroscopy can monitor chemical reactions occurring at the interface if these reactions involve changes in the chromophore's electronic structure or environment. An example is the metallation of a porphyrin base in a mixed monolayer with arachidic acid, which was followed

by observing changes in the reflection spectrum [149, 150]. Interestingly, this reaction was found to be sensitive to the nature of the co-lipid, as no complexation occurred when the porphyrin was mixed with methyl arachidate instead of arachidic acid, highlighting the influence of the local interfacial environment on reactivity. Time-dependent changes in reflection spectra can thus provide kinetic information about interfacial processes [142, 151].

5.2 Infrared Spectroscopy (IR)

Infrared spectroscopy probes the vibrational modes of molecules, providing information based on the principle that a vibration is IR active if it induces a change in the molecular dipole moment. The frequencies of IR absorption bands are characteristic of specific chemical bonds and functional groups (e.g., C-H, C=O, O-H, N-H), while band intensities and precise frequencies offer insights into molecular conformation (e.g., gauche vs. all-trans hydrocarbon chains), intermolecular interactions (e.g., hydrogen bonding), ionization state, and molecular orientation relative to the incident electric field.

Applying IR spectroscopy to Langmuir monolayers presents significant experimental challenges, primarily the difficulty in distinguishing the weak monolayer signal from the strong IR absorption of the bulk water subphase and atmospheric water vapor [152]. Standard transmission IR is thus impractical. Reflection-based techniques are necessary [153, 154, 155], though focusing an IR beam onto the water surface carries a potential risk of localized heating [156].

Several reflection IR techniques are employed for Langmuir film studies:

- **External Reflection Fourier Transform Infrared (ER-FTIR) Spectroscopy**: This technique involves reflecting an IR beam off the air-water interface at a specific angle. For studies at the air-water interface, this method is very often referred to interchangeably as **Infrared Reflection-Absorption Spectroscopy (IRRAS)** [153, 157, 158]. Modern setups often use dedicated Langmuir troughs within the FTIR spectrometer [26] or external reflection accessories [159]. To minimize water vapor interference, spectra are typically obtained by alternating measurements between the monolayer-covered surface (sample) and the pure water surface (reference), often using a two-compartment trough, or by employing rapid background acquisition strategies. The resulting

spectrum is commonly presented as reflection-absorbance, $-log(R/R_0)$, where R and R_0 are the reflectivities of the monolayer-covered and bare water surfaces, respectively. The angle of incidence and the polarization of the IR beam (p-polarized: electric field parallel to the plane of incidence; s-polarized: electric field perpendicular to the plane of incidence) critically influence the observed spectra [160]. Analyzing band intensities measured with different polarizations or angles allows deduction of the average orientation of molecular transition dipoles, and hence molecular orientation [161, 162, 57].

For instance, studies on phospholipid monolayers like DPPC using polarized ER-FTIR (as shown in Figure 10) revealed splitting of the methylene C-H stretching bands ($\nu_{as}(CH_2)$ and $\nu_s(CH_2)$) at low surface pressures, attributed to the coexistence of ordered (all-trans) and disordered (gauche) chain conformations. It is noteworthy that this splitting, clearly visible in the polarized spectra, was reportedly not observed in earlier unpolarised monolayer spectra. The frequencies of these C-H stretching modes are sensitive indicators of chain packing: lower frequencies (e.g., ∼2918 cm^{-1} for $\nu_{as}(CH_2)$ and ∼2850 cm^{-1} for $\nu_s(CH_2)$) indicate highly ordered, crystalline-like packing, while higher frequencies signify more disordered, liquid-like states. ER-FTIR/IRRAS has been instrumental in studying conformational changes in interfacial polypeptides like poly(β-benzyl-L-aspartate) (PBLA), revealing α-helix stability and sensitivity to subphase conditions. It has also quantified functional group orientation, such as the lift-off of the hydroxyl group in 12-hydroxystearic acid monolayers, monitored interfacial reactions like barbituric acid head group cleavage, and investigated chain orientation in nylon oligomers and polymers.

- **Attenuated Total Reflectance (ATR)-FTIR Spectroscopy**: While ER-FTIR/IRRAS is more common for *in situ* Langmuir film analysis, ATR-FTIR has also been adapted for this purpose [163, 164]. This typically involves positioning an internal reflection element (IRE), like a hydrophobized germanium crystal, just beneath the air-water interface. The IR beam undergoes multiple internal reflections within the IRE, creating an evanescent wave that probes the monolayer. This approach can enhance the signal-to-noise ratio. Results obtained using this method for *in situ* studies of phospholipid Langmuir films have shown similarities to ATR-IR results reported for transferred Langmuir-

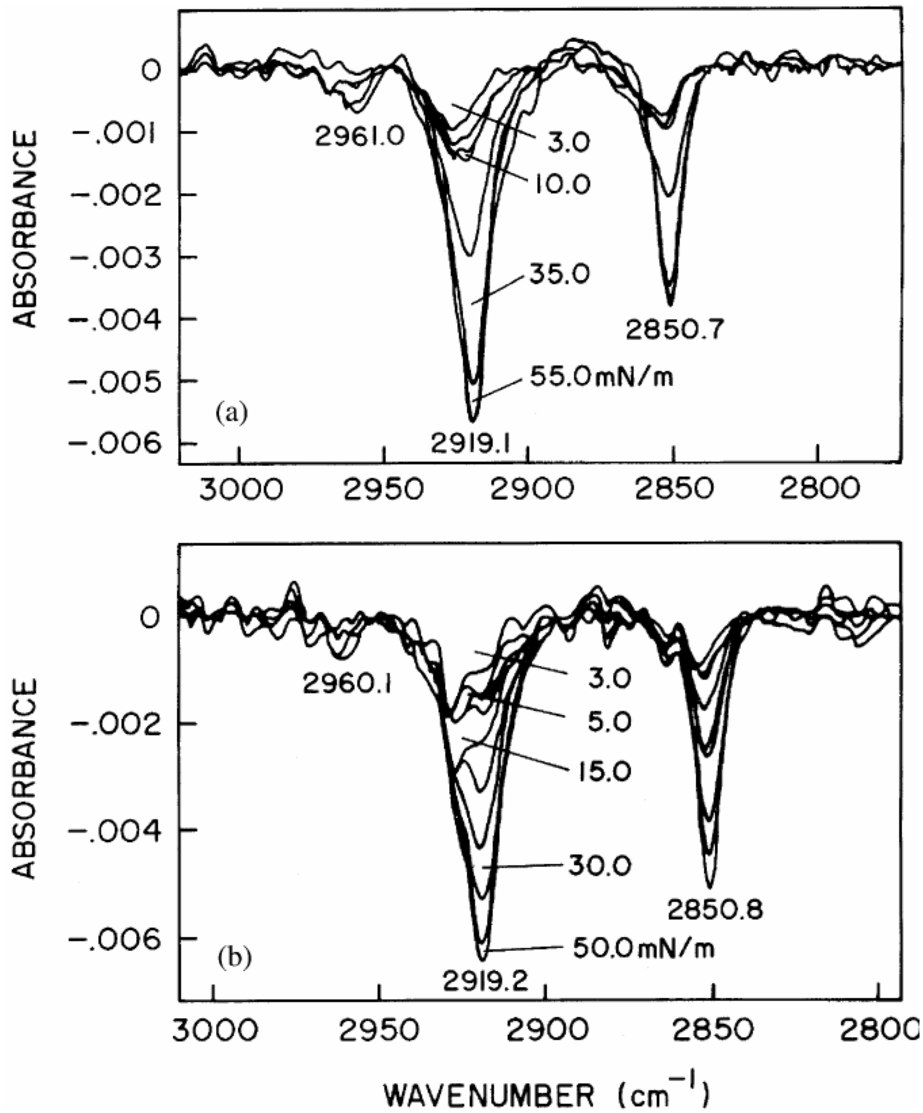

Figure 10: IR external reflection-absorbance spectra at the air/water interfaces as a function of surface pressure for DPPC at a 30° angle of incidence. (a) s-polarized (perpendicular) spectra. (b) p-polarized (parallel) spectra. Adapted from Dynarowicz-Łatka, P., et al., 2001 [6].

Blodgett (LB) films on solid IREs [165].

- **Polarization Modulated Infrared Reflection Absorption Spec-**

troscopy (PM-IRRAS): This advanced differential technique significantly boosts sensitivity and simplifies orientation analysis [166]. PM-IRRAS rapidly alternates the polarization of the incident IR beam between p- and s-states and detects the differential reflectivity signal ($\Delta R/R = (R_p - R_s)/(R_p + R_s)$). Because atmospheric absorptions and instrumental drifts impact both polarizations similarly, they are effectively cancelled in the differential signal, enabling high-quality spectra acquisition with significantly fewer scans compared to conventional ER-FTIR [155]. Critically, PM-IRRAS directly yields information on molecular orientation: vibrational modes with transition dipoles perpendicular to the surface (out-of-plane) typically show negative bands in the ΔR spectrum (at grazing incidence), while modes with parallel (in-plane) transition dipoles yield positive bands [162]. This allows immediate qualitative orientation assessment. For example, PM-IRRAS analysis of fatty acid salt monolayers clearly distinguishes the out-of-plane symmetric COO^- stretch (negative band) from the in-plane antisymmetric COO^- stretch (positive band), offering insights into head group anchoring [28]. Studies on cellulose alkyl ethers used PM-IRRAS to confirm the perpendicular orientation of alkyl side chains via positive methylene stretching bands [167].

Quantitative orientation analysis from IR spectra often involves comparing experimental data with theoretical optical models of the air-monolayer-water system [153, 168]. These models account for experimental parameters (angle of incidence, polarization) and film properties (thickness, refractive index, transition dipole orientation) to simulate spectra. Fitting simulations to data can yield average molecular tilt angles [169, 170, 133]. Comparing reflection spectra with transmission spectra (e.g., of transferred LB films) or isotropic bulk spectra also aids analysis [57, 162], but requires careful consideration of optical effects inherent to reflection measurements [145, 171].

5.3 Raman Spectroscopy

Raman spectroscopy, based on inelastic light scattering, provides vibrational information complementary to IR. A vibration is Raman active if it changes the molecule's polarizability. Since IR and Raman spectroscopy have different selection rules (dipole moment change vs. polarizability change), they often reveal different vibrational modes or the same modes with different

intensities, offering a more complete vibrational profile.

Applying Raman spectroscopy to Langmuir monolayers is challenged by the inherently weak Raman scattering effect [172, 27, 173]. Detecting signals from a monolayer requires high sensitivity and often enhancement techniques [174, 35, 175, 176]. Fluorescence from the sample or impurities can also interfere, potentially overwhelming the Raman signal, especially with visible excitation. However, Raman offers key advantages: water is a weak Raman scatterer, minimizing subphase interference, and the use of visible/near-IR light imposes fewer restrictions on trough materials than IR.

Several Raman approaches are used for Langmuir films:

- **Total Internal Reflection (TIR) Raman Spectroscopy**: An evanescent wave generated by total internal reflection of the excitation laser at the interface probes the monolayer. Early TIR Raman studies, often using perdeuterated amphiphiles (like stearic acid-d_{35}) to reduce fluorescence and shift signals, provided insights into monolayer order [177]. For example, narrowing of the CD_2 stretching bandwidth in stearic acid-d_{35} monolayers upon compression indicated an ordering transition from gauche-rich to all-trans conformations.

- **Surface-Enhanced Raman Spectroscopy (SERS)**: SERS uses nanostructured metal surfaces (Ag, Au) to dramatically amplify the Raman signal of nearby molecules via localized surface plasmon resonance [176, 175]. For Langmuir films, SERS is achieved by spreading the monolayer over metal colloids in the subphase [178] or by growing a rough metal film under the monolayer. SERS provides much better signal-to-noise for monolayer spectra. SERS studies on stearic acid and phospholipids have probed both hydrocarbon chain and head group regions [179, 180]. Comparisons between SERS spectra of stearic acid monolayers and solid stearic acid Raman spectra (Figure 11) revealed broader, higher-frequency peaks in the monolayer, suggesting a less ordered, possibly liquid-crystalline state compared to the bulk solid.

- **Resonance Raman Spectroscopy (RRS)**: If the excitation laser wavelength falls within an electronic absorption band of the monolayer's chromophore, the Raman scattering intensity for vibrations coupled to that electronic transition can be significantly enhanced (by orders of magnitude) [181]. This technique is particularly useful for studying chromophore-containing monolayers, selectively amplifying their

signals over non-absorbing components or the subphase [35].

- **Fourier Transform (FT)-Raman Spectroscopy**: Using near-infrared (NIR) excitation (e.g., 1064 nm) largely eliminates fluorescence interference. While NIR excitation results in inherently weaker Raman scattering (intensity $\propto \nu^4$), FT interferometry and sensitive detectors can compensate, making FT-Raman suitable for fluorescent samples. However, its application directly to Langmuir monolayers is less common due to sensitivity limitations for single layers compared to enhanced techniques [172, 27].

Overall, Raman spectroscopy, especially when enhanced (SERS, RRS), offers valuable complementary vibrational data to IR, particularly benefiting from low water interference. The combined application of UV-Vis, IR, and Raman spectroscopies provides a powerful suite of tools for detailed physicochemical characterization of molecular organization, conformation, orientation, and interactions within Langmuir films at the air-water interface.

6 Advanced Topics and Emerging Trends

The Langmuir-Blodgett (LB) technique, while rooted in the study of classical amphiphilic molecules at the air-water interface, has continually evolved, embracing new materials, exploring sophisticated applications, and delving into more complex interfacial phenomena. The precise layer-by-layer control inherent to the LB method makes it an invaluable tool in nanotechnology and materials science, allowing for the construction of highly ordered ultrathin films with tailored functionalities. This section explores recent advancements, focusing on the expansion of LB techniques to encompass novel material systems, their integration into cutting-edge technological applications, the critical influence of the subphase environment, and the development of dynamic, responsive systems akin to molecular machines. These emerging trends highlight the enduring relevance and adaptability of LB technology in addressing contemporary scientific and engineering challenges, pushing the boundaries of thin-film fabrication and interfacial science. The ability to assemble diverse components, from polymers and nanoparticles to complex biomolecules, into well-defined architectures opens up avenues for creating materials and devices with unprecedented properties and functions, moving far beyond the initial scope of simple fatty acid monolayers.

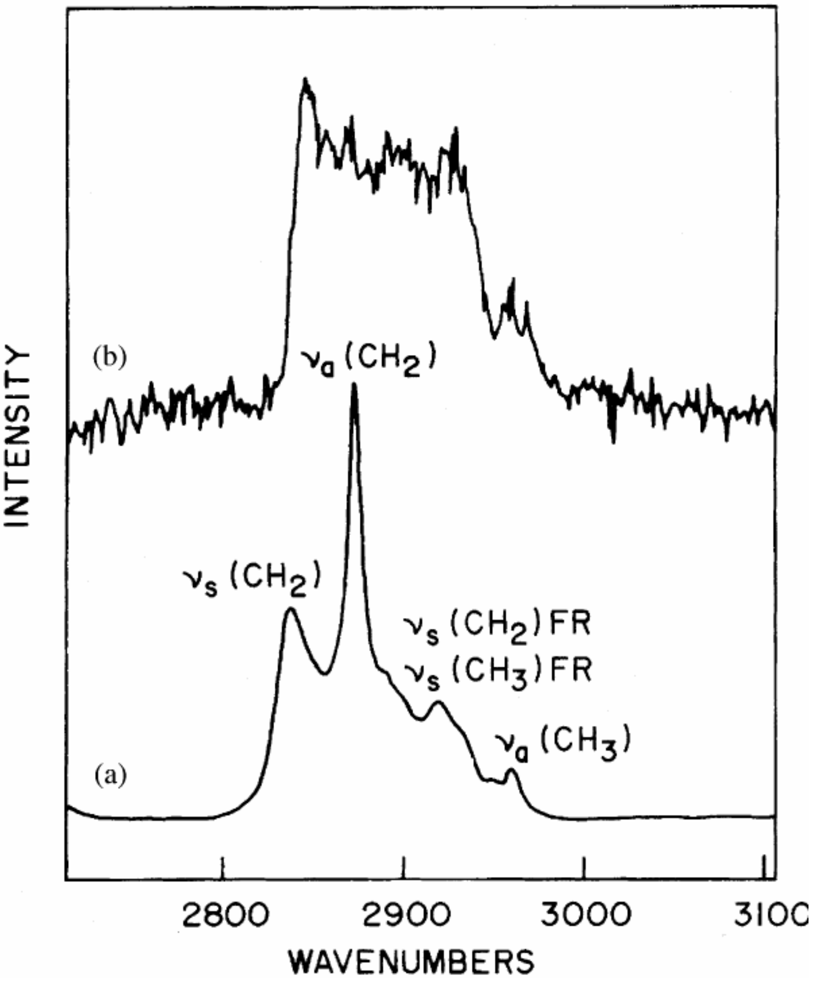

Figure 11: Raman spectra in the ν(CH) region of (a) solid stearic acid and (b) Langmuir monolayer of stearic acid at 35 mN m^{-1}. Adapted from Dynarowicz-Łatka, Patrycja, et al., 2001.

6.1 Langmuir-Blodgett Films from Novel Materials

While the foundational work on Langmuir and LB films centered on small amphiphilic molecules like fatty acids, the quest for materials with enhanced mechanical stability, diverse electronic and optical properties, and specific functionalities has driven the extension of the technique to a wide array of non-traditional materials. This expansion includes polymers, macromolecules,

various types of nanomaterials, and two-dimensional (2D) materials such as graphene, metal-organic frameworks (MOFs), and covalent organic frameworks (COFs). The primary motivation behind incorporating these materials is the potential to create robust films with tailored properties suitable for demanding applications in electronics, sensing, catalysis, and energy storage. Polymers, for instance, offer improved mechanical strength compared to monomeric amphiphiles. Polymeric LB films can be fabricated using several strategies: transferring monomeric Langmuir films followed by in-situ polymerization on the substrate, polymerizing monomers directly at the air-water interface before transfer, or spreading preformed polymers. The choice of method depends critically on the polymer's properties and the desired film architecture.

The integration of nanomaterials into Langmuir and LB films represents a significant frontier [13, 23, 25]. Various nanomaterial types, including nanoparticles (e.g., Au, Ag, CdSe) [182, 183, 184, 185, 186], nanorods (e.g., $BaCrO_4$) [50], nanowires (e.g., ZnO, Si), and carbon-based nanomaterials (e.g., carbon nanotubes, graphene, black phosphorus nanosheets) [187, 188, 189], have all been successfully incorporated into Langmuir monolayers and subsequently transferred as LB films. These nanomaterials can be spread directly if appropriately functionalized to possess amphiphilic character or co-spread with traditional amphiphiles or polymers that act as dispersing and transferring agents [18]. For example, studies have shown that functionalizing multi-walled carbon nanotubes (MWCNTs) with polyaniline (PANI) reduces their aggregation and facilitates directional assembly at the air-water interface, leading to ordered PANI@MWCNT LB films [187, 190]. Similarly, single-walled carbon nanotubes (SWCNTs) have been assembled into SWCNT-polymer composite monolayers and transferred as LB films, with the ordering dependent on the SWCNT production method [188, 189]. Graphene oxide, with its amphiphilic nature due to oxygen-containing functional groups, readily forms Langmuir films whose density can be controlled via compression [191, 192]. The compression process itself can induce self-assembly and ordering of nanoparticles at the air-water interface, as illustrated in Figure 12, where silver nanoparticles form a structured "2-D foam" phase under sufficient surface pressure [184, 185].

However, extending the LB technique to these novel materials is not without challenges. Solubility can be an issue, as many polymers and nanomaterials do not readily dissolve in the volatile organic solvents (like chloroform) typically used for spreading. Mixtures of solvents or pre-dissolution

steps may be necessary. Achieving uniform spreading at the air-water interface can also be difficult; cohesive forces between nanoparticles or polymer chains can lead to the formation of 3D aggregates rather than true monolayers. Furthermore, the resulting Langmuir films, especially those containing rigid components like nanowires or aggregated nanoparticles, can be brittle or non-uniform, making efficient transfer onto solid substrates problematic. High transfer ratios and homogeneous deposition require careful optimization of spreading conditions, surface pressure, subphase composition, and transfer speed. Strategies to overcome these hurdles include chemical functionalization to enhance solubility and interfacial activity (e.g., alkylation of chitosans or nucleotides), the use of assisting surfactants or polymers, and precise control over the compression process to manage film rheology. Despite these challenges, the ability to create highly organized mono- and multilayer films from these advanced materials, often exhibiting unique collective properties arising from their controlled assembly, continues to drive research in this area, opening possibilities for fabricating functional surfaces and devices with nanoscale precision [13, 5, 38, 23]. For instance, highly oriented films of semiconducting polymers have been achieved using specialized approaches like high-temperature LB deposition [193], demonstrating the potential for controlling molecular arrangement critical for electronic applications [194, 195, 196].

6.2 Langmuir-Blodgett Films in Advanced Applications

The unique capability of the Langmuir-Blodgett technique to construct ultrathin films with molecular-level precision in thickness and architecture has positioned it as a key enabling technology for a variety of advanced applications. While widespread industrial adoption remains limited due to factors like scalability and cost compared to methods like layer-by-layer assembly or self-assembled monolayers, LB films serve as crucial platforms for fundamental research and proof-of-principle demonstrations in diverse fields, including molecular electronics, sensing, energy storage, and biomimetic systems.

In molecular electronics, the initial excitement surrounding LB films stemmed from the possibility of creating highly ordered structures to function as components in nanoscale devices, potentially overcoming the miniaturization limits of conventional silicon technology [198, 199, 13]. Research explored the use of LB films made from conducting polymers [195], functionalized organic molecules [200, 85, 201], and nanoparticles [20] as active layers in organic

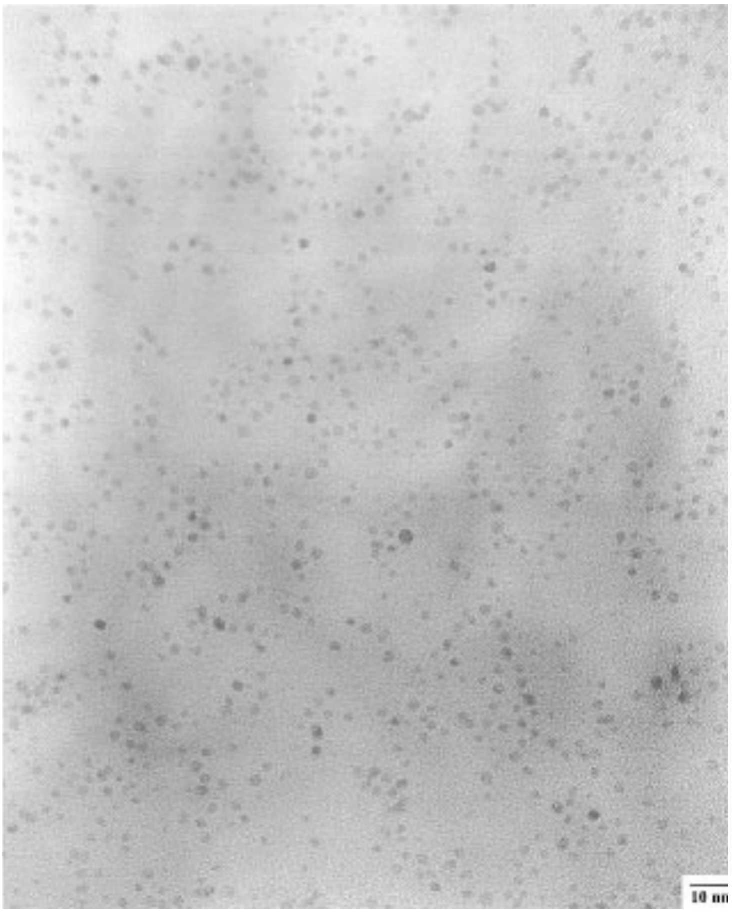

Figure 12: Compressed phase (20 mN/m; 50% coverage; 25 ˚C) of case Id particles (1.8 nm diameter Ag; C12 cap). Note the similarity of this "2-D foam" phase to that shown in Figure 4, bottom. Adapted from Heath, James R., et al., 1997 [197].

field-effect transistors (OFETs) [202, 203], diodes, and memory elements [204]. The precise control over layer thickness and molecular orientation offered by the LB method is particularly advantageous for studying charge transport phenomena in thin organic films and at interfaces [23]. For example, LB deposition has been used to create highly uniform monolayers that serve as gate dielectrics or semiconductor channels in OFETs, allowing for systematic investigation of structure-property relationships [202].

Sensing represents one of the most explored application areas for LB

films. The ability to create ultrathin, high-surface-area films with controlled composition and structure is ideal for detecting analytes in gas or liquid phases. Gas sensors based on LB films of conducting polymers [205], phthalocyanines [206], porphyrins [207], metal oxides [208], or composite materials (e.g., polymer-nanotube composites, MOF films, black phosphorus nanosheets mixed with dyes) [209, 210] have demonstrated enhanced sensitivity, selectivity, and response times towards various target analytes, including acidic/alkaline vapors and volatile organic compounds (VOCs) [211]. The LB technique allows for optimization of the film structure to maximize interaction with target gas molecules. For instance, fatty acid LB films deposited on surface acoustic wave (SAW) devices have shown selective responses to chloroform [212]. Furthermore, LB films can be integrated into flexible or wearable sensor platforms by deposition onto substrates like fabric (e.g., linen, cotton), enabling applications in environmental monitoring or personal health tracking. Biosensors frequently utilize LB films as matrices for immobilizing biological recognition elements like enzymes or antibodies [213, 214, 215]. Lipid or phospholipid LB films serve as excellent mimics of cell membranes, providing a biocompatible environment for enzyme activity. Glucose sensors have been developed using LB films containing glucose oxidase (GOx) [216], often combined with nanoparticles (e.g., AuNPs) to enhance electrochemical signal transduction. Similarly, LB films incorporating antibodies have been used for detecting disease markers, such as dengue NS1 antigen, often employing composite films (e.g., MoS_2/AuNP) to improve sensitivity.

In the realm of energy storage, particularly for lithium-based batteries, the LB technique offers precise control over electrode architecture and interfacial properties [13, 5, 38]. LB assembly has been employed to fabricate electrode materials with well-defined nanostructures, such as hybrid films of graphene nanosheets (GNS) and single-walled carbon nanotubes (SWNTs) [217], or nanocomposites of tin oxide (SnO_2) and reduced graphene oxide (RGO) [218, 219]. These LB-fabricated electrodes often exhibit improved performance, including higher specific capacity, better rate capability, and enhanced cycling stability, attributed to the controlled dispersion of active materials, optimized conductive networks, and accommodation of volume changes during cycling [220, 221]. The technique can also be used to create artificial solid electrolyte interphase (SEI) layers [222, 223] or protective coatings on electrodes [224], potentially improving battery safety and longevity [225].

Biomimetic systems heavily rely on LB films due to their structural similarity to biological membranes [226]. Langmuir monolayers of lipids, phospholipids, cholesterol, and proteins serve as powerful models for studying membrane structure, fluidity, and interactions with drugs, peptides, or other biomolecules [227, 228, 229, 230, 231, 232]. By controlling the composition and surface pressure of the monolayer, researchers can mimic different physiological states of cell membranes and investigate molecular mechanisms underlying biological processes at the interface [120, 233, 234]. These studies provide valuable insights for drug design [235] and understanding membrane protein function. Beyond model systems, the potential exists to use LB technology to build more complex biomimetic structures for applications in tissue engineering [15, 236] or as components of artificial organs, leveraging the technique's ability to create biocompatible, layered assemblies. The versatility of the LB method in incorporating diverse molecules and nanomaterials into highly organized thin films continues to fuel innovation across these advanced application domains [23, 14, 237].

6.3 Influence of Subphase Composition

The aqueous subphase in the Langmuir trough is far more than a passive support for the floating monolayer; its composition plays a crucial and active role in determining the structure, stability, phase behavior, and ultimate properties of both the Langmuir film at the air-water interface and the subsequently transferred LB film. Variations in pH, ionic content, temperature, and the presence of dissolved species can profoundly influence the interactions between amphiphilic molecules and between the molecules and the subphase, offering a powerful means to tune film characteristics.

The pH of the subphase directly affects the ionization state of ionizable head groups present in many amphiphiles, such as carboxylic acids (e.g., fatty acids) or amines. For a fatty acid monolayer, lowering the pH below its pKa suppresses ionization of the carboxyl head group [76, 74], reducing electrostatic repulsion and potentially leading to more condensed film structures [47]. Conversely, increasing the pH promotes ionization [76, 74], increasing head group repulsion and expanding the film [47]. This pH-dependent behavior influences the surface pressure-area isotherm, film compressibility, domain morphology [238], and stability against collapse [239]. Precise pH control is therefore essential for reproducible film formation and for studying pH-responsive systems.

The presence of ions in the subphase, particularly multivalent cations like Ca^{2+}, Mg^{2+}, Cd^{2+}, or Ba^{2+}, can have dramatic effects on monolayers containing negatively charged head groups (e.g., ionized fatty acids, phospholipids like phosphatidylserine) [240, 241, 242]. These cations can bind to the head groups, acting as bridges between adjacent molecules, screening electrostatic repulsion, and inducing significant changes in molecular packing [243, 244], film condensation, mechanical properties (e.g., increasing rigidity), and stability [245]. The specific type and concentration of ions can alter the phase behavior of the monolayer [47] and even influence the type of multilayer structure formed during LB deposition (e.g., promoting Y-type vs. X- or Z-type transfer) [246, 247, 11]. For instance, divalent cations are often required to form stable, transferable films of certain phospholipids. Careful selection of subphase ions allows for fine-tuning of intermolecular interactions and film structure.

Temperature is another critical parameter, although its manipulation is often constrained by the need to minimize water evaporation from the subphase in standard setups. Temperature affects the kinetic energy of the molecules and the fluidity of the monolayer. Increasing temperature generally leads to more expanded film phases and can influence the kinetics of domain formation and relaxation processes. While challenging, specialized LB troughs have been developed to operate at elevated temperatures, sometimes using alternative subphases with lower vapor pressure, such as ethylene glycol. High-temperature LB deposition can be advantageous for processing materials, like certain conjugated polymers, that tend to aggregate undesirably at room temperature, potentially leading to more uniform and highly oriented films with improved properties (e.g., charge carrier mobility).

Beyond adjusting bulk properties like pH and ionic strength, the subphase can serve as a reservoir for introducing specific molecules intended to interact directly with the Langmuir monolayer. This includes proteins, peptides, drugs, polymers, or nanoparticles dissolved in the subphase. These dissolved species can adsorb to the interface, bind specifically to components of the monolayer, or even penetrate into the film, leading to the formation of complex interfacial structures. This approach is widely used in biomimetic studies to investigate interactions between soluble proteins and lipid membranes or to study the effect of drugs on membrane models. It also provides a route for creating composite LB films where one component is introduced via the subphase rather than being co-spread. Understanding and controlling the subphase composition is therefore paramount for tailoring the properties

of Langmuir and LB films for specific applications and for conducting meaningful interfacial studies.

6.4 Molecular Machines and Responsive Systems

The air-water interface, as manipulated within a Langmuir trough, provides a unique environment for assembling and controlling molecular systems that can respond to external stimuli, functioning akin to molecular machines or receptors. The ability to precisely control the area available to molecules (via barrier compression/expansion) and to monitor interfacial properties (surface pressure, surface potential) allows for the investigation and manipulation of molecular behavior in a constrained 2D environment. This concept extends to the transferred LB films, which can retain the responsiveness engineered at the interface.

A key idea is the coupling between macroscopic mechanical actions (barrier movement) and molecular-level events. Compressing a Langmuir film can induce phase transitions, alter molecular orientation, force molecules into specific arrangements, or trigger conformational changes. This mechanical control can be used to operate molecular machines assembled at the interface. The mechanism often involves altering intermolecular distances or inducing specific molecular conformations through compression, which in turn triggers a desired function, such as binding or release of a guest molecule, or a change in optical or electronic properties. Hans Kuhn's pioneering work envisioned building complex supramolecular assemblies layer-by-layer using the LB technique, where different molecular components would interact to perform specific functions like energy or electron transfer, laying the groundwork for functional molecular systems.

Responsiveness can be triggered by various external stimuli beyond mechanical force. Light is a common stimulus used in photo-responsive Langmuir and LB films. Incorporating photochromic molecules, such as azobenzene derivatives, allows for reversible control over film properties. Upon irradiation with specific wavelengths of light, these molecules undergo isomerization (e.g., trans-cis), leading to changes in their shape and dipole moment. This molecular change can translate into macroscopic alterations in the film's area, surface pressure, surface potential, or wettability. Such photoswitchable films have potential applications in optical data storage, command surfaces for liquid crystal alignment, or light-controlled release systems.

Chemical stimuli, often mediated through changes in the subphase, can

also induce responses. As discussed previously, altering the subphase pH can reversibly change the ionization state and interactions of pH-sensitive molecules within the film, leading to structural reorganizations. Similarly, the specific binding of analytes (ions, molecules) from the subphase to receptor molecules incorporated into the Langmuir monolayer can trigger conformational changes or signaling events. This principle is fundamental to the operation of many LB-based chemical sensors and biosensors, where molecular recognition at the interface leads to a measurable output signal. The air-water interface provides an ideal platform for studying molecular recognition events in a controlled manner, allowing researchers to tune receptor density and orientation. An example of such a system operating at the air-water interface is illustrated in Figure 13, where a steroid cyclophane undergoes conformational changes, driven by interfacial conditions or external stimuli, to capture or release guest molecules, effectively acting as a molecular machine.

The concept of interfacial nanoarchitectonics leverages the air-water interface and LB transfer to construct dynamic and functional systems. By carefully designing molecular components and controlling the interfacial environment, researchers aim to create systems where molecular motion and interactions can be precisely controlled and utilized. This includes efforts to build molecular rotors, switches, or shuttles within the confined geometry of a Langmuir film. The dynamic nature of the interface allows for structural optimization of receptor molecules or the operation of molecular machines through macroscopic actions.

These responsive systems hold promise for applications in smart materials, adaptive coatings, controlled drug delivery, and advanced sensing platforms, demonstrating the power of the LB technique not just for static film deposition but also for creating dynamic, functional molecular assemblies.

7 Challenges and Future Directions

Despite the precise control over molecular architecture offered by Langmuir (L) and Langmuir-Blodgett (LB) techniques and their century-long contributions [13, 5], significant challenges hinder widespread industrial adoption. Overcoming limitations related to film stability [11, 248, 249], defect control [140, 141], scalability, reproducibility, and fundamental understanding is crucial for future progress. This requires interdisciplinary efforts bridging

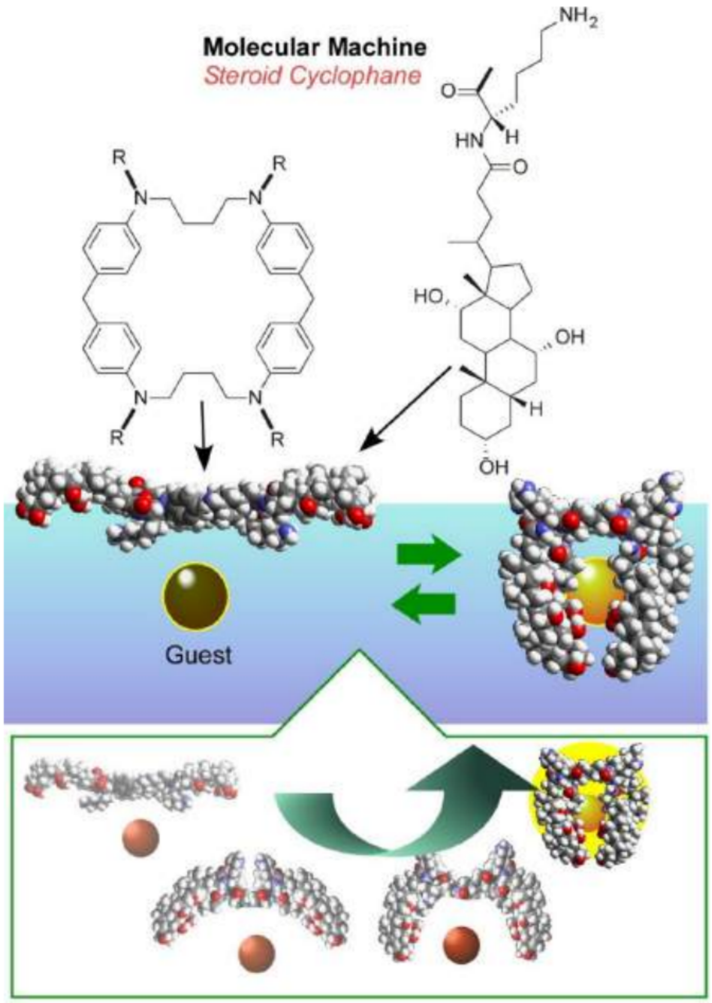

Figure 13: Capture and release of guest molecules upon conformational changes of steroid cyclophane as a molecular machine at the air—water interface. Adapted from Oliveira Jr., O., et al., 2022 [138].

chemistry, physics, biology, materials science, and engineering.

A persistent challenge relates to film stability. While classical films from small amphiphiles were known for fragility, ensuring mechanical robustness and thermal stability remains crucial even with modern materials like polymers and nanomaterials. These advanced materials, despite improvements, can still face issues during complex assembly or under operational stress, restricting practical utility in demanding applications. Strategies to enhance

stability include using preformed polymers [18], in-situ polymerization, chemical cross-linking, or developing hybrid structures. Indeed, many modern LB applications involve materials beyond classical amphiphiles, such as functionalized polymers, nanoparticles [250], carbon nanomaterials (graphene, nanotubes) [251], metal-organic frameworks (MOFs) [23, 252], covalent organic frameworks (COFs) [23, 252], and hybrid materials. Forming stable, transferable Langmuir films from these complex systems necessitates tailored strategies for dissolution, spreading, compression, and transfer, presenting unique challenges compared to traditional fatty acids. Figure 14 exemplifies the capability of LB techniques for advanced materials, illustrating how precisely aligned nanowires can be transferred from the air-water interface onto a substrate, forming ordered arrays crucial for nanoelectronic device fabrication and demonstrating the principles of nanoarchitectonics [253, 14, 23].

The presence of defects within LB films, such as pinholes, domain boundaries, vacancies, stacking faults, and impurities, is another critical issue compromising structural integrity and functional performance. These defects arise from various factors including material purity, spreading/compression conditions, transfer instabilities, and substrate quality. Advanced characterization techniques like Scanning Probe Microscopy (SPM), Brewster Angle Microscopy (BAM), and scattering methods are vital for understanding defect formation. Future work must focus on refining these techniques and developing strategies to minimize defects through optimized processes and environmental control.

Scalability, cost-effectiveness, and ensuring consistent reproducibility represent major hurdles for industrial application. Conventional LB deposition is slow, equipment-intensive, and often limited to small areas. While alternative techniques like self-assembled monolayers (SAMs) and layer-by-layer (LbL) assembly offer advantages such as simplicity, robustness [14, 237, 255, 253], and a wider range of materials (especially water-soluble ones for LbL) [15, 256, 257, 258], the LB method retains unique strengths in achieving precise control over molecular orientation and packing density for insoluble or complex amphiphiles [13, 259, 260]. Future efforts should target novel large-area deposition methods [198, 261, 262], continuous processing [263], and protocol optimization to enhance throughput and cost-competitiveness for high-value applications demanding molecular precision.

A deeper fundamental understanding of interfacial processes and film transfer is essential. The correlation between Langmuir monolayer structure

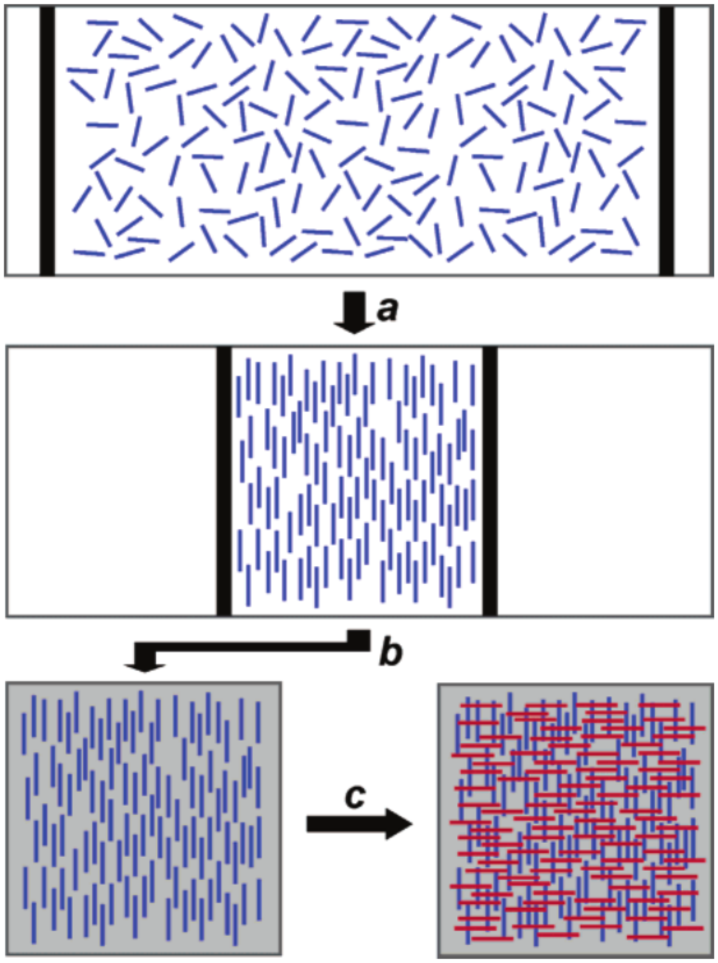

Figure 14: NWs (blue lines) in a monolayer of surfactant at the air—water interface are (a) compressed on a Langmuir—Blodgett trough to a specified pitch. (b) The aligned NWs are transferred to the surface of a substrate to make a uniform parallel array. (c) Crossed NW structures are formed by uniform transfer of a second layer of aligned parallel NWs (red lines) perpendicular to the first layer (blue lines). Adapted from Whang, D., et al., 2003 [254].

at the interface and the final LB film properties requires careful study, as reorganization can occur during transfer [264]. Establishing clear structure-property relationships necessitates combining *in situ* characterization of Langmuir films (surface pressure-area isotherms, surface potential [265], BAM

[139], GIXD [266]) with *ex situ* analysis of LB films. A key future direction involves the development and integration of advanced *in-situ* characterization techniques alongside predictive computational modeling, including molecular dynamics (MD) [267] and quantum methods, to enable rational design and overcome current limitations.

Looking ahead, future research will continue exploring novel materials with tailored functionalities and improved stability. Nanoarchitectonics, using LB to build complex supramolecular systems, holds promise for advanced devices in molecular electronics, sensors (gas, bio, electronic noses/tongues, flexible/wearable), and energy applications [268, 269, 23, 270]. The use of Langmuir monolayers to mimic biological membranes remains invaluable for biophysical studies, drug discovery, and diagnostics, particularly with increasingly realistic models [233, 36, 271]. Furthermore, exploiting the air-water interface directly for 'interfacial nanoarchitectonics'—studying molecular recognition and assembling functional supramolecular systems *at* the interface rather than solely relying on transferred films—represents a distinct and promising avenue [23, 272, 273]. Addressing these challenges and opportunities requires strong interdisciplinary collaboration. While widespread industrial use may be limited compared to simpler techniques, the unique capabilities of L and LB methods ensure their continued relevance in fundamental research and specialized applications where precise molecular control is paramount, guaranteeing a rich future for this field [13, 5, 38].

8 Conclusion

This survey has reviewed the field of Langmuir and Langmuir-Blodgett (LB) films, examining their historical development, beginning with the foundational work of pioneers like Langmuir and Blodgett, through fundamental principles, contemporary characterization methods, advanced applications, and future prospects [16, 274, 5]. The review covered the essential concepts: the controlled formation of ordered monomolecular Langmuir films via amphiphilic self-assembly at the air-water interface, governed by surface pressure, and their subsequent transfer onto solid substrates to create LB films [4, 275, 276]. The thermodynamics and phase behavior of these quasi-two-dimensional systems were explored, emphasizing the influence of molecular structure, subphase conditions, and deposition parameters on the final film properties [17, 277, 278].

A significant portion focused on the physicochemical characterization techniques used to probe Langmuir monolayers *in situ*. Macroscopic methods like surface pressure and potential measurements provide thermodynamic insights, while microscopic techniques such as Brewster Angle Microscopy (BAM) [135, 134] and interface-specific Atomic Force Microscopy (AFM) [32, 279, 280, 281] visualize film morphology. Spectroscopic tools, including Infrared Reflection-Absorption Spectroscopy (IRRAS) [153, 158, 157] and Polarization Modulation IRRAS (PM-IRRAS) [166, 282], reveal molecular orientation and composition. Synchrotron X-ray reflectivity and Grazing Incidence X-ray Diffraction (GIXD) [266] precisely determine structural parameters like thickness and molecular packing. The synergy of these techniques yields a comprehensive understanding crucial for fabricating high-quality LB films.

The discussion extended to advanced topics, highlighting the expansion beyond classical amphiphiles to include polymers, nanoparticles, carbon nanomaterials (e.g., graphene oxide, nanotubes), metal-organic frameworks (MOFs), and biomolecules. This diversification aligns with the principles of nanoarchitectonics, utilizing the LB technique for bottom-up construction of complex, functional supramolecular assemblies. Emerging applications in sensing, molecular electronics, energy storage, and biomimetic systems were examined, where the precise control offered by LB methods remains advantageous. The distinct characteristics and applications of Langmuir-Blodgett versus Langmuir-Schaefer deposition were also considered, assuming detailed comparison within the main body.

Finally, the survey addressed inherent challenges and future directions. While issues of mechanical stability, long-term durability, defect control, and process scalability persist—the latter viewed in context with alternative techniques like self-assembled monolayers (SAMs) and layer-by-layer (LbL) assembly suitable for certain industrial needs—progress continues through material innovation and hybrid structures. Future research will likely focus on novel stable materials, improved deposition for large areas, integration into complex devices, and leveraging the air-water interface for fundamental studies in biomimicry and molecular recognition, aided by computational modeling.

In summary, Langmuir and Langmuir-Blodgett film technology remains a dynamic research area. Its unique capability for creating highly ordered ultrathin films with molecular precision offers a vital platform for fundamental science and the development of advanced materials and devices. Continuous

innovation ensures that Langmuir and LB films will significantly contribute to diverse scientific and technological frontiers, driven by the fundamental control over molecular assembly that underpins nanotechnology and materials design.

References

[1] A S Martin et al. "Measurement of a Langmuir monomolecular film pressure-area compression isotherm using laser light diffracted from surface capillary waves". In: *European Journal of Physics* 14.1 (1993), 19–22. ISSN: 1361-6404. DOI: 10.1088/0143-0807/14/1/004. URL: http://dx.doi.org/10.1088/0143-0807/14/1/004.

[2] Kenjiro Miyano and Toshiteru Maeda. "Langmuir trough with four movable barriers". In: *Review of Scientific Instruments* 58.3 (1987), 428–435. ISSN: 1089-7623. DOI: 10.1063/1.1139249. URL: http://dx.doi.org/10.1063/1.1139249.

[3] C. J. L. Constantino, A. Dhanabalan, and O. N. Oliveira. "Experimental artifacts in the surface pressure measurement for lignin monolayers in Langmuir troughs". In: *Review of Scientific Instruments* 70.9 (1999), 3674–3680. ISSN: 1089-7623. DOI: 10.1063/1.1149949. URL: http://dx.doi.org/10.1063/1.1149949.

[4] María Mercedes Velázquez et al. "Langmuir-Blodgett Methodology: A Versatile Technique to Build 2D Material Films". In: *Two-dimensional Materials - Synthesis, Characterization and Potential Applications.* InTech, 2016. ISBN: 9789535125556. DOI: 10.5772/63495. URL: http://dx.doi.org/10.5772/63495.

[5] Wenhui Gu et al. "Recent Progress in the Applications of Langmuir–Blodgett Film Technology". In: *Nanomaterials* 14.12 (2024), p. 1039. ISSN: 2079-4991. DOI: 10.3390/nano14121039. URL: http://dx.doi.org/10.3390/nano14121039.

[6] Patrycja Dynarowicz-Łatka, Anantharaman Dhanabalan, and Osvaldo N. Oliveira. "Modern physicochemical research on Langmuir monolayers". In: *Advances in Colloid and Interface Science* 91.2 (2001), 221–293. ISSN: 0001-8686. DOI: 10.1016/s0001-8686(99)00034-2. URL: http://dx.doi.org/10.1016/s0001-8686(99)00034-2.

[7] Katsuhiko Ariga and Jonathan P. Hill. "Monolayers at air-water interfaces: from origins-of-life to nanotechnology". In: *The Chemical Record* 11.4 (2011), 199–211. ISSN: 1528-0691. DOI: 10.1002/tcr.201100004. URL: http://dx.doi.org/10.1002/tcr.201100004.

[8] Zhong-hou Cai and Stuart A. Rice. "Comment on the tilting transition in Langmuir monolayers". In: *The Journal of Chemical Physics* 96.8 (1992), 6229–6235. ISSN: 1089-7690. DOI: 10.1063/1.462614. URL: http://dx.doi.org/10.1063/1.462614.

[9] Kyle-Pierre Bell and Stuart A. Rice. "A molecular dynamics study of the structure of a long chain amphiphile monolayer adsorbed on ice Ih". In: *The Journal of Chemical Physics* 99.5 (1993), 4160–4167. ISSN: 1089-7690. DOI: 10.1063/1.466112. URL: http://dx.doi.org/10.1063/1.466112.

[10] Ka Yee C. Lee. "Collapse Mechanisms of Langmuir Monolayers". In: *Annual Review of Physical Chemistry* 59.1 (2008), 771–791. ISSN: 1545-1593. DOI: 10.1146/annurev.physchem.58.032806.104619. URL: http://dx.doi.org/10.1146/annurev.physchem.58.032806.104619.

[11] Dawn Y. Takamoto et al. "Stable Ordering in Langmuir-Blodgett Films". In: *Science* 293.5533 (2001), 1292–1295. ISSN: 1095-9203. DOI: 10.1126/science.1060018. URL: http://dx.doi.org/10.1126/science.1060018.

[12] J. B. Peng et al. "The structure of ultrathin Langmuir-Blodgett films of cadmium behenate". In: *The Journal of Chemical Physics* 123.21 (2005). ISSN: 1089-7690. DOI: 10.1063/1.2131067. URL: http://dx.doi.org/10.1063/1.2131067.

[13] Katsuhiko Ariga et al. "25th Anniversary Article: What Can Be Done with the Langmuir-Blodgett Method? Recent Developments and its Critical Role in Materials Science". In: *Advanced Materials* 25.45 (2013), 6477–6512. ISSN: 1521-4095. DOI: 10.1002/adma.201302283. URL: http://dx.doi.org/10.1002/adma.201302283.

[14] Katsuhiko Ariga et al. "Layer-by-layer Nanoarchitectonics: Invention, Innovation, and Evolution". In: *Chemistry Letters* 43.1 (2013), 36–68. ISSN: 1348-0715. DOI: 10.1246/cl.130987. URL: http://dx.doi.org/10.1246/cl.130987.

[15] João Borges et al. "Recent Developments in Layer-by-Layer Assembly for Drug Delivery and Tissue Engineering Applications". In: *Advanced Healthcare Materials* 13.8 (2024). ISSN: 2192-2659. DOI: 10.1002/adhm.202302713. URL: http://dx.doi.org/10.1002/adhm.202302713.

[16] Thi Thao Vu et al. "EVALUATION OF RESEARCH PROGRESS, TRENDS, AND APPLICATIONS OF LANGMUIR-BLODGETT FILMS OF FATTY ACIDS". In: *ChemChemTech* 68.2 (2024), 6–45. ISSN: 0579-2991. DOI: 10.6060/ivkkt.20256802.7002. URL: http://dx.doi.org/10.6060/ivkkt.20256802.7002.

[17] Anna M. Bibo and Ian R. Peterson. "Phase diagrams of monolayers of the long chain fatty acids". In: *Advanced Materials* 2.6–7 (1990), 309–311. ISSN: 1521-4095. DOI: 10.1002/adma.19900020608. URL: http://dx.doi.org/10.1002/adma.19900020608.

[18] Frank Embs et al. "Preformed polymers for Langmuir–Blodgett films–molecular concepts". In: *Advanced Materials* 3.1 (1991), 25–31. ISSN: 1521-4095. DOI: 10.1002/adma.19910030106. URL: http://dx.doi.org/10.1002/adma.19910030106.

[19] Tokuji Miyashita. "Recent studies on functional ultrathin polymer films prepared by the Langmuir-Blodgett technique". In: *Progress in Polymer Science* 18.2 (1993), 263–294. ISSN: 0079-6700. DOI: 10.1016/0079-6700(93)90027-a. URL: http://dx.doi.org/10.1016/0079-6700(93)90027-a.

[20] S. Paul et al. "LangmuirBlodgett Film Deposition of Metallic Nanoparticles and Their Application to Electronic Memory Structures". In: *Nano Letters* 3.4 (2003), 533–536. ISSN: 1530-6992. DOI: 10.1021/nl034008t. URL: http://dx.doi.org/10.1021/nl034008t.

[21] T.S. Berzina et al. "Langmuir-Blodgett films composed of monolayers of amphiphilic molecules and adsorbed soluble proteins". In: *Thin Solid Films* 284–285 (1996), 757–761. ISSN: 0040-6090. DOI: 10.1016/s0040-6090(95)08439-8. URL: http://dx.doi.org/10.1016/s0040-6090(95)08439-8.

[22] A. Tronin, T. Dubrovsky, and C. Nicolini. "Comparative Study of Langmuir Monolayers of Immunoglobulin G Formed at the Air-Water Interface and Covalently Immobilized on Solid Supports". In: *Lang-*

muir 11.2 (1995), 385–389. ISSN: 1520-5827. DOI: 10.1021/la00002a001. URL: http://dx.doi.org/10.1021/la00002a001.

[23] Katsuhiko Ariga et al. "Materials nanoarchitectonics at two-dimensional liquid interfaces". In: *Beilstein Journal of Nanotechnology* 10 (2019), 1559–1587. ISSN: 2190-4286. DOI: 10.3762/bjnano.10.153. URL: http://dx.doi.org/10.3762/bjnano.10.153.

[24] Katsuhiko Ariga. "Don't Forget Langmuir–Blodgett Films 2020: Interfacial Nanoarchitectonics with Molecules, Materials, and Living Objects". In: *Langmuir* 36.26 (2020), 7158–7180. ISSN: 1520-5827. DOI: 10.1021/acs.langmuir.0c01044. URL: http://dx.doi.org/10.1021/acs.langmuir.0c01044.

[25] Katsuhiko Ariga. "Chemistry of Materials Nanoarchitectonics for Two-Dimensional Films: Langmuir–Blodgett, Layer-by-Layer Assembly, and Newcomers". In: *Chemistry of Materials* 35.14 (2023), 5233–5254. ISSN: 1520-5002. DOI: 10.1021/acs.chemmater.3c01291. URL: http://dx.doi.org/10.1021/acs.chemmater.3c01291.

[26] Richard A. Dluhy et al. "Design and Interfacing of an Automated Langmuir-Type Film Balance to an FT-IR Spectrometer". In: *Applied Spectroscopy* 42.7 (1988), 1289–1293. ISSN: 1943-3530. DOI: 10.1366/0003702884430047. URL: http://dx.doi.org/10.1366/0003702884430047.

[27] N. Castaings et al. "Raman spectroscopy of LB films and monolayers at the air-water interface". In: *Thin Solid Films* 284–285 (1996), 631–635. ISSN: 0040-6090. DOI: 10.1016/s0040-6090(95)08408-8. URL: http://dx.doi.org/10.1016/s0040-6090(95)08408-8.

[28] D. Blaudez et al. "Organization in pure and alternate deuterated cadmium arachidate monolayers on solid substrates and at the air/water interface studied by conventional and differential Fourier transform infrared spectroscopies". In: *The Journal of Chemical Physics* 104.24 (1996), 9983–9993. ISSN: 1089-7690. DOI: 10.1063/1.471726. URL: http://dx.doi.org/10.1063/1.471726.

[29] Chen Fang et al. "Recent Applications of Langmuir–Blodgett Technique in Battery Research". In: *ACS Applied Materials amp; Interfaces* 14.2 (2022), 2431–2439. ISSN: 1944-8252. DOI: 10.1021/acsami.1c19064. URL: http://dx.doi.org/10.1021/acsami.1c19064.

[30] Karl Spratte and Hans Riegler. "Steady State Morphology and Composition of Mixed Monomolecular Films (Langmuir Monolayers) at the Air/Water Interface in the Vicinity of the Three-Phase Line: Model Calculations and Experiments". In: *Langmuir* 10.9 (1994), 3161–3173. ISSN: 1520-5827. DOI: 10.1021/la00021a045. URL: http://dx.doi.org/10.1021/la00021a045.

[31] Yuh-Lang Lee, Jing-Yi Lin, and Chien-Hsiang Chang. "Thermodynamic characteristics and Langmuir–Blodgett deposition behavior of mixed DPPA/DPPC monolayers at air/liquid interfaces". In: *Journal of Colloid and Interface Science* 296.2 (2006), 647–654. ISSN: 0021-9797. DOI: 10.1016/j.jcis.2005.09.050. URL: http://dx.doi.org/10.1016/j.jcis.2005.09.050.

[32] D. K. Schwartz, R. Viswanathan, and J. A. Zasadzinski. "Examining Langmuir-Blodgett Films with Atomic Force Microscopy". In: *Science* 263.5150 (1994), 1158–1158. ISSN: 1095-9203. DOI: 10.1126/science.263.5150.1158. URL: http://dx.doi.org/10.1126/science.263.5150.1158.

[33] L.F. Chi et al. "Investigations of phase-separated Langmuir-Blodgett films by atomic force microscopy". In: *Thin Solid Films* 242.1–2 (1994), 151–156. ISSN: 0040-6090. DOI: 10.1016/0040-6090(94)90520-7. URL: http://dx.doi.org/10.1016/0040-6090(94)90520-7.

[34] D. Blaudez et al. "Characterization of Langmuir-Blodgett monolayers using polarization modulated FTIR spectroscopy". In: *Thin Solid Films* 210–211 (1992), 648–651. ISSN: 0040-6090. DOI: 10.1016/0040-6090(92)90364-h. URL: http://dx.doi.org/10.1016/0040-6090(92)90364-h.

[35] J. Souto, R. Aroca, and J. A. Desaja. "Surface-enhanced Raman spectra of langmuir–blodgett monolayers of (hexadecamethyltetralino) porphyrazines". In: *Journal of Raman Spectroscopy* 25.6 (1994), 435–440. ISSN: 1097-4555. DOI: 10.1002/jrs.1250250610. URL: http://dx.doi.org/10.1002/jrs.1250250610.

[36] Monika Rojewska et al. "Langmuir Monolayer Techniques for the Investigation of Model Bacterial Membranes and Antibiotic Biodegradation Mechanisms". In: *Membranes* 11.9 (2021), p. 707. ISSN: 2077-0375. DOI: 10.3390/membranes11090707. URL: http://dx.doi.org/10.3390/membranes11090707.

[37] V. I. Troitsky et al. "Instrument for depositing Langmuir–Blodgett films composed of alternating monolayers using a protective layer of water". In: *Review of Scientific Instruments* 67.12 (1996), 4216–4223. ISSN: 1089-7623. DOI: 10.1063/1.1147571. URL: http://dx.doi.org/10.1063/1.1147571.

[38] Wenhui Gu et al. "Recent Progress in the Applications of Langmuir–Blodgett Film Technology". In: *Nanomaterials* 14.12 (2024), p. 1039. ISSN: 2079-4991. DOI: 10.3390/nano14121039. URL: http://dx.doi.org/10.3390/nano14121039.

[39] Joseph T. Buontempo and Frank A. Novak. "An inexpensive Wilhelmy balance for the study of Langmuir monolayers". In: *Review of Scientific Instruments* 63.12 (1992), 5707–5713. ISSN: 1089-7623. DOI: 10.1063/1.1143353. URL: http://dx.doi.org/10.1063/1.1143353.

[40] K. Halperin, J. B. Ketterson, and P. Dutta. "A study of the mechanical behavior of surface monolayers using orthogonal Wilhelmy plates". In: *Langmuir* 5.1 (1989), 161–164. ISSN: 1520-5827. DOI: 10.1021/la00085a030. URL: http://dx.doi.org/10.1021/la00085a030.

[41] Siji Sudheesh and Jamil Ahmad. "Effect of Wilhelmy Plate Material on Hysteresis of Langmuir Film Isotherms". In: *Asian Journal of Chemistry* 25.7 (2013), 3535–3538. ISSN: 0975-427X. DOI: 10.14233/ajchem.2013.13213. URL: http://dx.doi.org/10.14233/ajchem.2013.13213.

[42] D. G. Dervichian. "Changes of Phase and Transformations of Higher Order in Monolayers". In: *The Journal of Chemical Physics* 7.10 (1939), 931–948. ISSN: 1089-7690. DOI: 10.1063/1.1750347. URL: http://dx.doi.org/10.1063/1.1750347.

[43] F. Schmid and M. Schick. "Liquid phases of Langmuir monolayers". In: *The Journal of Chemical Physics* 102.5 (1995), 2080–2091. ISSN: 1089-7690. DOI: 10.1063/1.468729. URL: http://dx.doi.org/10.1063/1.468729.

[44] J. P. Legré, G. Albinet, and A. Caillé. "Models for the phase transition in a monolayer of amphiphilic bipolar molecules on an aqueous substrate". In: *Canadian Journal of Physics* 60.6 (1982), 893–900. ISSN: 1208-6045. DOI: 10.1139/p82-123. URL: http://dx.doi.org/10.1139/p82-123.

[45] Sharon Grayer Wolf et al. "A Synchrotron X-ray Study of a Solid-Solid Phase Transition in a Two-Dimensional Crystal". In: *Science* 242.4883 (1988), 1286–1290. ISSN: 1095-9203. DOI: 10.1126/science.242.4883.1286. URL: http://dx.doi.org/10.1126/science.242.4883.1286.

[46] Jaroslaw Majewski et al. "The Structural Properties of Uncompressed Crystalline Monolayers of Alcohols CnH2n+1 OH (n = 13–31) on Water and Their Role as Ice Nucleators". In: *Chemistry – A European Journal* 1.5 (1995), 304–311. ISSN: 1521-3765. DOI: 10.1002/chem.19950010507. URL: http://dx.doi.org/10.1002/chem.19950010507.

[47] M. C. Shih et al. "Pressure and pH dependence of the structure of a fatty acid monolayer with calcium ions in the subphase". In: *The Journal of Chemical Physics* 96.2 (1992), 1556–1559. ISSN: 1089-7690. DOI: 10.1063/1.462139. URL: http://dx.doi.org/10.1063/1.462139.

[48] Daniel K. Schwartz. "Langmuir-Blodgett film structure". In: *Surface Science Reports* 27.7 (1997), pp. 245–334. ISSN: 0167-5729. DOI: https://doi.org/10.1016/S0167-5729(97)00003-4. URL: https://www.sciencedirect.com/science/article/pii/S0167572997000034.

[49] F. Kopp et al. "Spontaneous Rearrangement in Langmuir-Blodgett Layers of Tripalmitin Studied by Means of ATR Infrared Spectroscopy and Electron Microscopy". In: *Zeitschrift für Naturforschung C* 30.11–12 (1975), 711–717. ISSN: 0939-5075. DOI: 10.1515/znc-1975-11-1202. URL: http://dx.doi.org/10.1515/znc-1975-11-1202.

[50] Chia-Chen Lin et al. "Langmuir-Blodgett Films of Alkanethiolate Gold Nanorods". In: *Journal of the Chinese Chemical Society* 50.5 (2003), 1015–1021. ISSN: 2192-6549. DOI: 10.1002/jccs.200300143. URL: http://dx.doi.org/10.1002/jccs.200300143.

[51] Christophe Ybert et al. "Collapse of a Monolayer by Three Mechanisms". In: *The Journal of Physical Chemistry B* 106.8 (2002), 2004–2008. ISSN: 1520-5207. DOI: 10.1021/jp013173z. URL: http://dx.doi.org/10.1021/jp013173z.

[52] Christophe Ybert et al. “Collapse of a Monolayer by Three Mechanisms”. In: *The Journal of Physical Chemistry B* 106.8 (2002), 2004–2008. ISSN: 1520-5207. DOI: 10.1021/jp013173z. URL: http://dx.doi.org/10.1021/jp013173z.

[53] Siji Sudheesh, Jamil Ahmad, and Girija S. Singh. “Hysteresis of Isotherms of Mixed Monolayers of N-Octadecyl-N-phenylthiourea and Stearic Acid at Air/Water Interface”. In: *ISRN Physical Chemistry* 2012 (2012), 1–6. ISSN: 2090-7761. DOI: 10.5402/2012/835397. URL: http://dx.doi.org/10.5402/2012/835397.

[54] Hiromichi Nakahara et al. “Hysteresis behavior of amphiphilic model peptide in lung lipid monolayers at the air–water interface by an IR-RAS measurement”. In: *Colloids and Surfaces B: Biointerfaces* 68.1 (2009), 61–67. ISSN: 0927-7765. DOI: 10.1016/j.colsurfb.2008.09.013. URL: http://dx.doi.org/10.1016/j.colsurfb.2008.09.013.

[55] Tz. Ivanova et al. “Surface pressure—area hysteresis of surface films formed by spreading of phospholipid liposomes”. In: *Colloids and Surfaces* 60 (1991), 263–273. ISSN: 0166-6622. DOI: 10.1016/0166-6622(91)80281-r. URL: http://dx.doi.org/10.1016/0166-6622(91)80281-r.

[56] B. Lin et al. “Kinetics of a structural phase transition in Langmuir monolayers studied using x-ray diffraction”. In: *The Journal of Chemical Physics* 90.4 (1989), 2393–2397. ISSN: 1089-7690. DOI: 10.1063/1.456653. URL: http://dx.doi.org/10.1063/1.456653.

[57] J. F. Rabolt et al. “Anisotropic orientation in molecular monolayers by infrared spectroscopy”. In: *The Journal of Chemical Physics* 78.2 (1983), 946–952. ISSN: 1089-7690. DOI: 10.1063/1.444799. URL: http://dx.doi.org/10.1063/1.444799.

[58] D.M. Taylor and G.F. Bayes. “The surface potential of Langmuir monolayers”. In: *Materials Science and Engineering: C* 8–9 (1999), 65–71. ISSN: 0928-4931. DOI: 10.1016/s0928-4931(99)00064-8. URL: http://dx.doi.org/10.1016/s0928-4931(99)00064-8.

[59] A. Dhanabalan et al. “Surface Pressure and Surface Potential Isotherms of Ytterbium Bisphthalocyanine Langmuir Monolayers”. In: *Langmuir* 15.11 (1999), 3944–3949. ISSN: 1520-5827. DOI: 10.1021/la9815188. URL: http://dx.doi.org/10.1021/la9815188.

[60] Masamichi Fujihira and Hirosuke Kawate. "Structural study of Langmuir–Blodgett films by scanning surface potential microscopy". In: *Journal of Vacuum Science amp; Technology B: Microelectronics and Nanometer Structures Processing, Measurement, and Phenomena* 12.3 (1994), 1604–1608. ISSN: 1520-8567. DOI: 10.1116/1.587242. URL: http://dx.doi.org/10.1116/1.587242.

[61] Ailton Cavalli and Osvaldo N. Oliveira. "An improved system for measuring the lateral conductance of Langmuir monolayers". In: *Review of Scientific Instruments* 66.12 (1995), 5567–5569. ISSN: 1089-7623. DOI: 10.1063/1.1146022. URL: http://dx.doi.org/10.1063/1.1146022.

[62] Mitsumasa Iwamoto, Tohru Kubota, and Ou-Yang Zhong-can. "Maxwell-displacement-current across phospholipid monolayers due to phase transition". In: *The Journal of Chemical Physics* 104.2 (1996), 736–741. ISSN: 1089-7690. DOI: 10.1063/1.470798. URL: http://dx.doi.org/10.1063/1.470798.

[63] Xiaobin Xu Xiaobin Xu and Mitsumasa Iwamoto Mitsumasa Iwamoto. "Molecular Switching in Phospholipid and Azobenzene Mixed Monolayers Using Maxwell-Displacement-Current-Measuring Technique". In: *Japanese Journal of Applied Physics* 36.12R (1997), p. 7348. ISSN: 1347-4065. DOI: 10.1143/jjap.36.7348. URL: http://dx.doi.org/10.1143/jjap.36.7348.

[64] Chen-Xu Wu, Shin-ichi Kuragasaki Shin-ichi Kuragasaki, and Mitsumasa Iwamoto Mitsumasa Iwamoto. "Maxwell Displacement Current Across Monolayers with Dielectric Anisotropy due to Biaxiality on a Water Surface". In: *Japanese Journal of Applied Physics* 36.5R (1997), p. 2775. ISSN: 1347-4065. DOI: 10.1143/jjap.36.2775. URL: http://dx.doi.org/10.1143/jjap.36.2775.

[65] Kyung-Sup Lee and Mitsumasa Iwamoto. "Maxwell Displacement Current across Phospholipid Monolayers at the Air/Water Interface". In: *Journal of Colloid and Interface Science* 177.2 (1996), 414–418. ISSN: 0021-9797. DOI: 10.1006/jcis.1996.0053. URL: http://dx.doi.org/10.1006/jcis.1996.0053.

[66] Viola Vogel and Dietmar Möbius. "Local surface potentials and electric dipole moments of lipid monolayers: Contributions of the water/lipid and the lipid/air interfaces". In: *Journal of Colloid and In-*

terface Science 126.2 (1988), 408–420. ISSN: 0021-9797. DOI: 10.1016/0021-9797(88)90140-3. URL: http://dx.doi.org/10.1016/0021-9797(88)90140-3.

[67] A. V. Hughes, D. M. Taylor, and A. E. Underhill. "Molecular Orientation in Langmuir Monolayers Containing the [M(dmit)2]n-Anion Determined from Surface Pressure and Surface Potential Isotherms". In: *Langmuir* 15.7 (1999), 2477–2483. ISSN: 1520-5827. DOI: 10.1021/la9807154. URL: http://dx.doi.org/10.1021/la9807154.

[68] Adrien Sthoer et al. "La3+ and Y3+ interactions with the carboxylic acid moiety at the liquid/vapour interface: identification of binding complexes, charge reversal, and detection limits." In: (2021). DOI: 10.26434/chemrxiv-2021-28p74. URL: http://dx.doi.org/10.26434/chemrxiv-2021-28p74.

[69] J. T. Davies and Sir Eric Rideal. "INTERFACIAL POTENTIALS". In: *Canadian Journal of Chemistry* 33.5 (1955), 947–960. ISSN: 1480-3291. DOI: 10.1139/v55-114. URL: http://dx.doi.org/10.1139/v55-114.

[70] Daniel Schuhmann. "Electrical properties of adsorbed or spread films: The effective value of permittivities in the Helmholtz equation (plane distribution of point dipoles)". In: *Journal of Colloid and Interface Science* 134.1 (1990), 152–160. ISSN: 0021-9797. DOI: 10.1016/0021-9797(90)90261-l. URL: http://dx.doi.org/10.1016/0021-9797(90)90261-l.

[71] Mitsumasa Iwamoto, Yoshinobu Mizutani, and Akihiko Sugimura. "Calculation of the dielectric constant of monolayer films on a material surface". In: *Physical Review B* 54.11 (1996), 8186–8190. ISSN: 1095-3795. DOI: 10.1103/physrevb.54.8186. URL: http://dx.doi.org/10.1103/physrevb.54.8186.

[72] Usha Khanna and V.K. Srivastava. "Studies of dielectric constant of some "built-up" molecular films". In: *Thin Solid Films* 12.1 (1972), S25–S28. ISSN: 0040-6090. DOI: 10.1016/0040-6090(72)90419-1. URL: http://dx.doi.org/10.1016/0040-6090(72)90419-1.

[73] Hiroo Nakahara and Kiyoshige Fukuda. "Orientation of chromophores in monolayers and multilayers of azobenzene derivatives with long alkyl chains". In: *Journal of Colloid and Interface Science* 93.2 (1983),

530–539. ISSN: 0021-9797. DOI: 10.1016/0021-9797(83)90437-x. URL: http://dx.doi.org/10.1016/0021-9797(83)90437-x.

[74] J. J. Betts and B. A. Pethica. “The ionization characteristics of monolayers of weak acids and bases”. In: *Transactions of the Faraday Society* 52 (1956), p. 1581. ISSN: 0014-7672. DOI: 10.1039/tf9565201581. URL: http://dx.doi.org/10.1039/tf9565201581.

[75] Steven L. Carnie et al. “The structure of electrolytes at charged surfaces: The primitive model”. In: *The Journal of Chemical Physics* 74.2 (1981), 1472–1478. ISSN: 1089-7690. DOI: 10.1063/1.441161. URL: http://dx.doi.org/10.1063/1.441161.

[76] J. V. SANDERS and J. A. SPINK. “Ionization in Fatty Acid Monolayers on Pure Water”. In: *Nature* 175.4458 (1955), 644–645. ISSN: 1476-4687. DOI: 10.1038/175644a0. URL: http://dx.doi.org/10.1038/175644a0.

[77] Michel PRATS, Jean-François TOCANNE, and Justin TEISSIÉ. “Lateral proton conduction at a lipid/water interface: Its modulation by physical parameters. Experimental and mathematical approaches”. In: *European Journal of Biochemistry* 149.3 (1985), 663–668. ISSN: 1432-1033. DOI: 10.1111/j.1432-1033.1985.tb08975.x. URL: http://dx.doi.org/10.1111/j.1432-1033.1985.tb08975.x.

[78] H. Morgan, D.M. Taylor, and O.N. Oliveira. “Lateral conduction at a monolayer-water interface”. In: *Thin Solid Films* 178.1–2 (1989), 73–79. ISSN: 0040-6090. DOI: 10.1016/0040-6090(89)90288-5. URL: http://dx.doi.org/10.1016/0040-6090(89)90288-5.

[79] J Teissié et al. “Evidence for conduction of protons along the interface between water and a polar lipid monolayer.” In: *Proceedings of the National Academy of Sciences* 82.10 (1985), 3217–3221. ISSN: 1091-6490. DOI: 10.1073/pnas.82.10.3217. URL: http://dx.doi.org/10.1073/pnas.82.10.3217.

[80] Michel Prats, Justin Teissié, and Jean-François Tocanne. “Lateral proton conduction at lipid–water interfaces and its implications for the chemiosmotic-coupling hypothesis”. In: *Nature* 322.6081 (1986), 756–758. ISSN: 1476-4687. DOI: 10.1038/322756a0. URL: http://dx.doi.org/10.1038/322756a0.

[81] Jean-François Tocanne and Justin Teissié. "Ionization of phospholipids and phospholipid-supported interfacial lateral diffusion of protons in membrane model systems". In: *Biochimica et Biophysica Acta (BBA) - Reviews on Biomembranes* 1031.1 (1990), 111–142. ISSN: 0304-4157. DOI: 10.1016/0304-4157(90)90005-w. URL: http://dx.doi.org/10.1016/0304-4157(90)90005-w.

[82] F. M. Menger, S. D. Richardson, and G. R. Bromley. "Ion conductance along lipid monolayers". In: *Journal of the American Chemical Society* 111.17 (1989), 6893–6894. ISSN: 1520-5126. DOI: 10.1021/ja00199a089. URL: http://dx.doi.org/10.1021/ja00199a089.

[83] H. Morgan, D.M. Taylor, and O.N. Oliveira. "Two-dimensional proton conduction at a membrane surface: Influence of molecular packing and hydrogen bonding". In: *Chemical Physics Letters* 150.3–4 (1988), 311–314. ISSN: 0009-2614. DOI: 10.1016/0009-2614(88)80048-4. URL: http://dx.doi.org/10.1016/0009-2614(88)80048-4.

[84] B. Gabriel and J. Teissie. "Proton lateral conduction along lipid monolayers is present only in the liquid-expanded state". In: *Journal of the American Chemical Society* 113.23 (1991), 8818–8821. ISSN: 1520-5126. DOI: 10.1021/ja00023a033. URL: http://dx.doi.org/10.1021/ja00023a033.

[85] Richard L. McCreery and Adam Johan Bergren. "Progress with Molecular Electronic Junctions: Meeting Experimental Challenges in Design and Fabrication". In: *Advanced Materials* 21.43 (2009), 4303–4322. ISSN: 1521-4095. DOI: 10.1002/adma.200802850. URL: http://dx.doi.org/10.1002/adma.200802850.

[86] Mitsumasa Iwamoto, Tohru Kubota, and Muhamad Rasat Muhamad. "Maxwell displacement current due to phase transitions in liquid crystals on a water surface". In: *Thin Solid Films* 293.1–2 (1997), 299–302. ISSN: 0040-6090. DOI: 10.1016/s0040-6090(96)08984-5. URL: http://dx.doi.org/10.1016/s0040-6090(96)08984-5.

[87] Yoko Sato et al. "Determination of the Piezoelectric Coefficient of Monolayers on Water Surface by Maxwell-Displacement-Current Measurement". In: *Japanese Journal of Applied Physics* 37.1R (1998), p. 215. ISSN: 1347-4065. DOI: 10.1143/jjap.37.215. URL: http://dx.doi.org/10.1143/jjap.37.215.

[88] Yoko Sato et al. "Determination of Dielectric Relaxation Time of Langmuir-Films by a Whole-Curve Method Using the Maxwell Displacement Current". In: *Japanese Journal of Applied Physics* 37.10R (1998), p. 5655. ISSN: 1347-4065. DOI: 10.1143/jjap.37.5655. URL: http://dx.doi.org/10.1143/jjap.37.5655.

[89] Chen-Xu Wu, Yutaka Majima, and Mitsumasa Iwamoto. "Analysis of dielectric relaxation phenomena of monolayer films by monolayer compression". In: *Thin Solid Films* 327–329 (1998), 228–231. ISSN: 0040-6090. DOI: 10.1016/s0040-6090(98)00634-8. URL: http://dx.doi.org/10.1016/s0040-6090(98)00634-8.

[90] Shunichi Arisawa and Ryoichi Yamamoto. "Control of molecular orientations by a pulsating electric field applied to a monolayer at the air-water interface". In: *Langmuir* 9.4 (1993), 1028–1030. ISSN: 1520-5827. DOI: 10.1021/la00028a024. URL: http://dx.doi.org/10.1021/la00028a024.

[91] Keun-Ho Park and Mitsumasa Iwamoto. "Maxwell Displacement Current across Langmuir Phospholipid Monolayers Mixed with Azobenzene by Photoisomerization". In: *Journal of Colloid and Interface Science* 193.1 (1997), 71–76. ISSN: 0021-9797. DOI: 10.1006/jcis.1997.5035. URL: http://dx.doi.org/10.1006/jcis.1997.5035.

[92] Chen-Xu Wu, Zhong-can Ou-Yang, and Mitsumasa Iwamoto. "Polar orientational phase transition and differential dielectric constant of smectic monolayers on a water surface". In: *The Journal of Chemical Physics* 109.11 (1998), 4552–4561. ISSN: 1089-7690. DOI: 10.1063/1.477059. URL: http://dx.doi.org/10.1063/1.477059.

[93] Yutaka Majima et al. "Investigation of dielectric relaxation phenomena in liquid crystal monolayer at the air–water interface". In: *Thin Solid Films* 327–329 (1998), 232–235. ISSN: 0040-6090. DOI: 10.1016/s0040-6090(98)00635-x. URL: http://dx.doi.org/10.1016/s0040-6090(98)00635-x.

[94] Yutaka Majima, Yoko Sato Yoko Sato, and Mitsumasa Iwamoto Mitsumasa Iwamoto. "Dielectric Relaxation Phenomena of a Liquid Crystal Monolayer at the Air-Water Interface". In: *Japanese Journal of Applied Physics* 36.8R (1997), p. 5237. ISSN: 1347-4065. DOI: 10.1143/jjap.36.5237. URL: http://dx.doi.org/10.1143/jjap.36.5237.

[95] Chen-Xu Wu Chen-Xu Wu and Mitsumasa Iwamoto Mitsumasa Iwamoto. “Calculation of Molecular Orientational Order and Maxwell Displacement Current of Biaxial Molecules at the Air-liquid Interface”. In: *Japanese Journal of Applied Physics* 36.2R (1997), p. 824. ISSN: 1347-4065. DOI: 10.1143/jjap.36.824. URL: http://dx.doi.org/10.1143/jjap.36.824.

[96] Jürgen Maack et al. “Molecular cis-trans switching in amphiphilic monolayers containing azobenzene moieties”. In: *Thin Solid Films* 242.1–2 (1994), 122–126. ISSN: 0040-6090. DOI: 10.1016/0040-6090(94)90514-2. URL: http://dx.doi.org/10.1016/0040-6090(94)90514-2.

[97] Xiao Bin Xu, Yutaka Majima, and Mitsumasa Iwamoto. “Molecular switching in phospholipid-azobenzene mixed monolayers by photoisomerization”. In: *Thin Solid Films* 331.1–2 (1998), 239–247. ISSN: 0040-6090. DOI: 10.1016/s0040-6090(98)00925-0. URL: http://dx.doi.org/10.1016/s0040-6090(98)00925-0.

[98] N. Nandi and D. Vollhardt. “Chiral Discrimination Effects in Langmuir Monolayers: Monolayers of Palmitoyl Aspartic Acid,N-Stearoyl Serine Methyl Ester, andN-Tetradecyl-,-dihydroxypentanoic Acid Amide”. In: *The Journal of Physical Chemistry B* 107.15 (2003), 3464–3475. ISSN: 1520-5207. DOI: 10.1021/jp0218591. URL: http://dx.doi.org/10.1021/jp0218591.

[99] Jonathan G. Heath and Edward M. Arnett. “Chiral molecular recognition in monolayers of diastereomeric N-acylamino acid methyl esters at the air/water interface”. In: *Journal of the American Chemical Society* 114.12 (1992), 4500–4514. ISSN: 1520-5126. DOI: 10.1021/ja00038a009. URL: http://dx.doi.org/10.1021/ja00038a009.

[100] D. Vollhardt et al. “Chiral Discrimination and Pattern Formation in N-Dodecylmannonamide Monolayers at the AirWater Interface”. In: *Langmuir* 12.23 (1996), 5659–5663. ISSN: 1520-5827. DOI: 10.1021/la960471s. URL: http://dx.doi.org/10.1021/la960471s.

[101] M. Dvolaitzky and M. A. Guedeau-Boudeville. “Chiral Discrimination in Monolayer Packing of Hexade-Canol-Thiophosphoryl-2-Phenylglycinol with two Chiral Center in the Polar Head-Group”. In: *New Trends in Physics and Physucal Chemistry of Polymers*. Springer US, 1989,

81–87. ISBN: 9781461305439. DOI: 10.1007/978-1-4613-0543-9_8. URL: http://dx.doi.org/10.1007/978-1-4613-0543-9_8.

[102] U. Gehlert et al. "Chiral Discrimination in Monolayers of Monoglycerides". In: *Langmuir* 12.20 (1996), 4892–4896. ISSN: 1520-5827. DOI: 10.1021/la960382f. URL: http://dx.doi.org/10.1021/la960382f.

[103] R. Rietz et al. "Monolayer behaviour of chiral compounds at the air-water interface: 4-hexadecyloxy-butane-1,2-diol". In: *Thin Solid Films* 284–285 (1996), 211–215. ISSN: 0040-6090. DOI: 10.1016/s0040-6090(95)08366-9. URL: http://dx.doi.org/10.1016/s0040-6090(95)08366-9.

[104] Roland Rietz, Gerald Brezesinski, and Helmuth Möhwald. "Separation of Enantiomers in a Monolayer of Racemic 3-Hexadecyl-oxy-propane-1,2-diol". In: *Berichte der Bunsengesellschaft für physikalische Chemie* 97.10 (1993), 1394–1398. ISSN: 0005-9021. DOI: 10.1002/bbpc.19930971036. URL: http://dx.doi.org/10.1002/bbpc.19930971036.

[105] Martin V. Stewart and Edward M. Arnett. *Chiral Monolayers at the Air-Water Interface.* 1982. DOI: 10.1002/9780470147221.ch3. URL: http://dx.doi.org/10.1002/9780470147221.ch3.

[106] Ou-Yang Zhong-can et al. "Molecular twist transition in chiral and racemic phospholipid monolayers detected by Maxwell-displacement-current measurements". In: *Physical Review E* 59.2 (1999), 2105–2108. ISSN: 1095-3787. DOI: 10.1103/physreve.59.2105. URL: http://dx.doi.org/10.1103/physreve.59.2105.

[107] C. Böhm et al. "Influence of chirality on the structure of phospholipid monolayers". In: *Biophysical Journal* 64.2 (1993), 553–559. ISSN: 0006-3495. DOI: 10.1016/s0006-3495(93)81386-9. URL: http://dx.doi.org/10.1016/s0006-3495(93)81386-9.

[108] Shigeru Negi et al. "Highly Ordered Monolayers of an Optically Active Amphiphilic Pyrene Derivative at the Air–Water Interface". In: *Bulletin of the Chemical Society of Japan* 95.11 (2022), 1537–1545. ISSN: 1348-0634. DOI: 10.1246/bcsj.20220233. URL: http://dx.doi.org/10.1246/bcsj.20220233.

[109] Shigeru Negi et al. "Monolayer Formation and Chiral Recognition of Binaphthyl Amphiphiles at the Air–Water Interface". In: *Bulletin of the Chemical Society of Japan* 96.1 (2022), 48–56. ISSN: 1348-0634. DOI: 10.1246/bcsj.20220286. URL: http://dx.doi.org/10.1246/bcsj.20220286.

[110] M. Lösche, E. Sackmann, and H. Möhwald. "A Fluorescence Microscopic Study Concerning the Phase Diagram of Phospholipids". In: *Berichte der Bunsengesellschaft für physikalische Chemie* 87.10 (1983), 848–852. ISSN: 0005-9021. DOI: 10.1002/bbpc.19830871004. URL: http://dx.doi.org/10.1002/bbpc.19830871004.

[111] J. Teissié, J.F. Tocanne, and A. Baudras. "Phase transitions in phospholipid monolayers at the air—water interface: A fluorescence study". In: *FEBS Letters* 70.1–2 (1976), 123–126. ISSN: 1873-3468. DOI: 10.1016/0014-5793(76)80740-5. URL: http://dx.doi.org/10.1016/0014-5793(76)80740-5.

[112] Michael Seul and David Andelman. "Domain Shapes and Patterns: The Phenomenology of Modulated Phases". In: *Science* 267.5197 (1995), 476–483. ISSN: 1095-9203. DOI: 10.1126/science.267.5197.476. URL: http://dx.doi.org/10.1126/science.267.5197.476.

[113] T. K. Vanderlick and Helmuth. Moehwald. "Mode selection and shape transitions of phospholipid monolayer domains". In: *The Journal of Physical Chemistry* 94.2 (1990), 886–890. ISSN: 1541-5740. DOI: 10.1021/j100365a070. URL: http://dx.doi.org/10.1021/j100365a070.

[114] D. J. Keller, J. P. Korb, and H. M. McConnell. "Theory of shape transitions in two-dimensional phospholipid domains". In: *The Journal of Physical Chemistry* 91.25 (1987), 6417–6422. ISSN: 1541-5740. DOI: 10.1021/j100309a021. URL: http://dx.doi.org/10.1021/j100309a021.

[115] A. Miller and H. Möhwald. "Diffusion limited growth of crystalline domains in phospholipid monolayers". In: *The Journal of Chemical Physics* 86.7 (1987), 4258–4265. ISSN: 1089-7690. DOI: 10.1063/1.451886. URL: http://dx.doi.org/10.1063/1.451886.

[116] K. Kjaer et al. "Ordering in Lipid Monolayers Studied by Synchrotron X-Ray Diffraction and Fluorescence Microscopy". In: *Physical Review Letters* 59.6 (1987), 744–744. ISSN: 0031-9007. DOI: 10.1103/

physrevlett.59.744. URL: http://dx.doi.org/10.1103/physrevlett.59.744.

[117] Takamasa Ishikawa et al. "Maze Pattern at Nanometer-Scale in a Mixed Langmuir Monolayer of Fatty Acids". In: *Bulletin of the Chemical Society of Japan* 94.12 (2021), 2967–2969. ISSN: 1348-0634. DOI: 10.1246/bcsj.20210335. URL: http://dx.doi.org/10.1246/bcsj.20210335.

[118] J. Huang and G.W. Feigenson. "Monte Carlo simulation of lipid mixtures: finding phase separation". In: *Biophysical Journal* 65.5 (1993), 1788–1794. ISSN: 0006-3495. DOI: 10.1016/s0006-3495(93)81234-7. URL: http://dx.doi.org/10.1016/s0006-3495(93)81234-7.

[119] Jonas Korlach et al. "Characterization of lipid bilayer phases by confocal microscopy and fluorescence correlation spectroscopy". In: *Proceedings of the National Academy of Sciences* 96.15 (1999), 8461–8466. ISSN: 1091-6490. DOI: 10.1073/pnas.96.15.8461. URL: http://dx.doi.org/10.1073/pnas.96.15.8461.

[120] Andreas Santamaria et al. "Investigation on the relationship between lipid composition and structure in model membranes composed of extracted natural phospholipids". In: (2022). DOI: 10.26434/chemrxiv-2022-mrfmx. URL: http://dx.doi.org/10.26434/chemrxiv-2022-mrfmx.

[121] R. Verger and F. Pattus. "Lipid-protein interactions in monolayers". In: *Chemistry and Physics of Lipids* 30.2–3 (1982), 189–227. ISSN: 0009-3084. DOI: 10.1016/0009-3084(82)90052-4. URL: http://dx.doi.org/10.1016/0009-3084(82)90052-4.

[122] H BROCKMAN. "Lipid monolayers: why use half a membrane to characterize protein-membrane interactions?" In: *Current Opinion in Structural Biology* 9.4 (1999), 438–443. ISSN: 0959-440X. DOI: 10.1016/s0959-440x(99)80061-x. URL: http://dx.doi.org/10.1016/s0959-440x(99)80061-x.

[123] Katsuhiko Ariga and Toyoki Kunitake. "Molecular Recognition at AirWater and Related Interfaces: Complementary Hydrogen Bonding and Multisite Interaction". In: *Accounts of Chemical Research* 31.6 (1998), 371–378. ISSN: 1520-4898. DOI: 10.1021/ar970014i. URL: http://dx.doi.org/10.1021/ar970014i.

[124] Katsuhiko Ariga. “Molecular recognition at the air–water interface: nanoarchitectonic design and physicochemical understanding”. In: *Physical Chemistry Chemical Physics* 22.43 (2020), 24856–24869. ISSN: 1463-9084. DOI: 10.1039/d0cp04174b. URL: http://dx.doi.org/10.1039/d0cp04174b.

[125] Katsuhiko ARIGA and Toyoki KUNITAKE. “Molecular Recognition at Interfaces”. In: *Journal of Japan Oil Chemists' Society* 43.11 (1994), 914–920. ISSN: 1884-2003. DOI: 10.5650/jos1956.43.914. URL: http://dx.doi.org/10.5650/jos1956.43.914.

[126] Debora Berti et al. “Molecular Recognition in Monolayers. Complementary Base Pairing in Dioleoylphosphatidyl Derivatives of Adenosine, Uridine, and Cytidine”. In: *Langmuir* 13.13 (1997), 3438–3444. ISSN: 1520-5827. DOI: 10.1021/la970334a. URL: http://dx.doi.org/10.1021/la970334a.

[127] Takaharu Kawahara, Kazue Kurihara, and Toyoki Kunitake. “Cooperative Binding of Adenine via Complementary Hydrogen Bonding to an Imide Functionalized Monolayer at the Air-Water Interface”. In: *Chemistry Letters* 21.9 (1992), 1839–1842. ISSN: 1348-0715. DOI: 10.1246/cl.1992.1839. URL: http://dx.doi.org/10.1246/cl.1992.1839.

[128] Kazuhiro Taguchi, Katsuhiko Ariga, and Toyoki Kunitake. “Multi-site Recognition of Flavin Adenine Dinucleotide by Mixed Monolayers on Water”. In: *Chemistry Letters* 24.8 (1995), 701–702. ISSN: 1348-0715. DOI: 10.1246/cl.1995.701. URL: http://dx.doi.org/10.1246/cl.1995.701.

[129] Thomas Loughran et al. “Monomer–eximer dynamics in spread monolayers. I. Lateral diffusion of pyrene dodecanoic acid at the air–water interface”. In: *The Journal of Chemical Physics* 72.11 (1980), 5791–5797. ISSN: 1089-7690. DOI: 10.1063/1.439101. URL: http://dx.doi.org/10.1063/1.439101.

[130] Franz Grieser, Peter J. Thistlethwaite, and Robert S. Urquhart. “Pressure-area isotherms and fluorescence behaviour of 12-(1-pyrenyl)dodecanoic acid at the air-aqueous solution interface”. In: *Chemical Physics Letters* 141.1–2 (1987), 108–114. ISSN: 0009-2614. DOI: 10.1016/0009-2614(87)80101-x. URL: http://dx.doi.org/10.1016/0009-2614(87)80101-x.

[131] R Peters and K Beck. "Translational diffusion in phospholipid monolayers measured by fluorescence microphotolysis." In: *Proceedings of the National Academy of Sciences* 80.23 (1983), 7183–7187. ISSN: 1091-6490. DOI: 10.1073/pnas.80.23.7183. URL: http://dx.doi.org/10.1073/pnas.80.23.7183.

[132] Brian Moore et al. "Studies of phase transitions in Langmuir monolayers by fluorescence microscopy". In: *Journal of the Chemical Society, Faraday Transactions 2* 82.10 (1986), p. 1753. ISSN: 0300-9238. DOI: 10.1039/f29868201753. URL: http://dx.doi.org/10.1039/f29868201753.

[133] Kazukuni Hosoi et al. "Quantitative Image Analysis in Brewster Angle Microscopy: Molecular Tilt of a Stearic Acid Monolayer on Water". In: *Japanese Journal of Applied Physics* 32.1A (1993), p. L135. ISSN: 1347-4065. DOI: 10.1143/jjap.32.l135. URL: http://dx.doi.org/10.1143/jjap.32.l135.

[134] Dirk Honig, Gernot A. Overbeck, and Dietmar Mobius. "Morphology of pentadecanoic acid monolayers at the air/water interface studied by BAM". In: *Advanced Materials* 4.6 (1992), 419–424. ISSN: 1521-4095. DOI: 10.1002/adma.19920040610. URL: http://dx.doi.org/10.1002/adma.19920040610.

[135] Keith J. Stine. *Brewster Angle Microscopy.* 2012. DOI: 10.1002/9780470661345.smc040. URL: http://dx.doi.org/10.1002/9780470661345.smc040.

[136] Silvère Akamatsu et al. "Two-dimensional dendritic growth in Langmuir monolayers of d-myristoyl alanine". In: *Physical Review A* 46.8 (1992), R4504–R4507. ISSN: 1094-1622. DOI: 10.1103/physreva.46.r4504. URL: http://dx.doi.org/10.1103/physreva.46.r4504.

[137] Lucile Gambut et al. "Ellipsometry, Brewster Angle Microscopy, and Thermodynamic Studies of Monomolecular Films of Cryptophanes at the AirWater Interface". In: *Langmuir* 12.22 (1996), 5407–5412. ISSN: 1520-5827. DOI: 10.1021/la960245i. URL: http://dx.doi.org/10.1021/la960245i.

[138] Osvaldo N. Oliveira Jr., Luciano Caseli, and Katsuhiko Ariga. "The Past and the Future of Langmuir and Langmuir–Blodgett Films". In: *Chemical Reviews* 122.6 (2022), pp. 6459–6513. ISSN: 0009-2665. DOI:

10.1021/acs.chemrev.1c00754. URL: https://doi.org/10.1021/acs.chemrev.1c00754.

[139] W. J. Foster, M. C. Shih, and P. S. Pershan. "The structure of a Langmuir monolayer of methyl eicosanoate as determined by x-ray diffraction and Brewster angle microscopy". In: *The Journal of Chemical Physics* 105.8 (1996), 3307–3315. ISSN: 1089-7690. DOI: 10.1063/1.472185. URL: http://dx.doi.org/10.1063/1.472185.

[140] H. G. Hansma et al. "Imaging nanometer scale defects in Langmuir-Blodgett films with the atomic force microscope". In: *Langmuir* 7.6 (1991), 1051–1054. ISSN: 1520-5827. DOI: 10.1021/la00054a005. URL: http://dx.doi.org/10.1021/la00054a005.

[141] Pierre Lesieur, André Barraud, and Michel Vandevyver. "Defect characterization and detection in Langmuir-Blodgett films". In: *Thin Solid Films* 152.1–2 (1987), 155–164. ISSN: 0040-6090. DOI: 10.1016/0040-6090(87)90414-7. URL: http://dx.doi.org/10.1016/0040-6090(87)90414-7.

[142] H. Grüniger, D. Möbius, and H. Meyer. "Enhanced light reflection by dye monolayers at the air–water interface". In: *The Journal of Chemical Physics* 79.8 (1983), 3701–3710. ISSN: 1089-7690. DOI: 10.1063/1.446290. URL: http://dx.doi.org/10.1063/1.446290.

[143] A. K. Dutta, T. N. Misra, and A. J. Pal. "A Spectroscopic Study of Nonamphiphilic Pyrene Assembled in LangmuirBlodgett Films: Formation of Aggregates". In: *Langmuir* 12.2 (1996), 459–465. ISSN: 1520-5827. DOI: 10.1021/la940678q. URL: http://dx.doi.org/10.1021/la940678q.

[144] A. K. Dutta. "Aggregation-Induced Reabsorption of p-Quaterphenyl Assembled in Langmuir-Blodgett Films: A Fluorescence Study". In: *The Journal of Physical Chemistry* 99.40 (1995), 14758–14763. ISSN: 1541-5740. DOI: 10.1021/j100040a027. URL: http://dx.doi.org/10.1021/j100040a027.

[145] Michel Orrit et al. "Reflection and transmission of light by dye monolayers". In: *The Journal of Chemical Physics* 85.9 (1986), 4966–4979. ISSN: 1089-7690. DOI: 10.1063/1.451735. URL: http://dx.doi.org/10.1063/1.451735.

[146] Takashi Ubukata, Takahiro Seki, and Kunihiro Ichimura. “Molecular hybrids of azobenzene polymer and liquid crystal molecule. A new approach for understanding the command surface system”. In: *Macromolecular Symposia* 137.1 (1999), 25–31. ISSN: 1521-3900. DOI: 10.1002/masy.19991370104. URL: http://dx.doi.org/10.1002/masy.19991370104.

[147] Katsuhiko Nishiyama, Masa-Aki Kurihara, and Masamichi Fujihira. “Photochromism of an amphiphilic azobenzene derivative in its Langmuir-Blodgett films prepared as polyion complexes with ionic polymers”. In: *Thin Solid Films* 179.1–2 (1989), 477–483. ISSN: 0040-6090. DOI: 10.1016/0040-6090(89)90224-1. URL: http://dx.doi.org/10.1016/0040-6090(89)90224-1.

[148] Takeshi Kawai, Junzo Umemura, and Tohru Takenaka. “UV absorption spectra of azobenzene-containing long-chain fatty acids and their barium salts in spread monolayers and Langmuir-Blodgett films”. In: *Langmuir* 5.6 (1989), 1378–1383. ISSN: 1520-5827. DOI: 10.1021/la00090a020. URL: http://dx.doi.org/10.1021/la00090a020.

[149] R. Loschek and D. Möbius. “Metallation of porphyrins in lipid monolayers at the air/water interface”. In: *Chemical Physics Letters* 151.1–2 (1988), 176–182. ISSN: 0009-2614. DOI: 10.1016/0009-2614(88)80091-5. URL: http://dx.doi.org/10.1016/0009-2614(88)80091-5.

[150] R.H. Schmehl, G.L. Shaw, and D.G. Whitten. “Modification of interfacial reactivity by the microenvironment. Metallation of porphyrins in monolayer films and assemblies”. In: *Chemical Physics Letters* 58.4 (1978), 549–552. ISSN: 0009-2614. DOI: 10.1016/0009-2614(78)80016-5. URL: http://dx.doi.org/10.1016/0009-2614(78)80016-5.

[151] Miharu Eguchi et al. “Adsorchromism: Molecular Nanoarchitectonics at 2D Nanosheets—Old Chemistry for Advanced Chromism”. In: *Advanced Science* 8.14 (2021). ISSN: 2198-3844. DOI: 10.1002/advs.202100539. URL: http://dx.doi.org/10.1002/advs.202100539.

[152] Young Shin Kim et al. “Development of a Planar Array Infrared Reflection Spectrograph for Reflection—Absorption Spectroscopy of Thin Films at Metal and Water Surfaces”. In: *Applied Spectroscopy*

61.9 (2007), 916–920. ISSN: 1943-3530. DOI: 10.1366/000370207781745865. URL: http://dx.doi.org/10.1366/000370207781745865.

[153] Richard Mendelsohn, Joseph W. Brauner, and Arne Gericke. "External Infrared Reflection Absorption Spectrometry of Monolayer Films at the Air-Water Interface". In: *Annual Review of Physical Chemistry* 46.1 (1995), 305–334. ISSN: 1545-1593. DOI: 10.1146/annurev.pc.46.100195.001513. URL: http://dx.doi.org/10.1146/annurev.pc.46.100195.001513.

[154] Richard A. Dluhy, Norman A. Wright, and Peter R. Griffiths. "In Situ Measurement of the FT-IR Spectra of Phospholipid Monolayers at the Air/Water Interface". In: *Applied Spectroscopy* 42.1 (1988), 138–141. ISSN: 1943-3530. DOI: 10.1366/0003702884428680. URL: http://dx.doi.org/10.1366/0003702884428680.

[155] D. Blaudez et al. "Polarization-Modulated FT-IR Spectroscopy of a Spread Monolayer at the Air/Water Interface". In: *Applied Spectroscopy* 47.7 (1993), 869–874. ISSN: 1943-3530. DOI: 10.1366/0003702934415273. URL: http://dx.doi.org/10.1366/0003702934415273.

[156] Hiroshi Sakai and Junzo Umemura. "Effect of Infrared Radiation and Air Flow on Fourier Transform Infrared External Reflection Spectra of Langmuir Monolayers". In: *Langmuir* 13.3 (1997), 502–505. ISSN: 1520-5827. DOI: 10.1021/la960915k. URL: http://dx.doi.org/10.1021/la960915k.

[157] Richard Mendelsohn, Guangru Mao, and Carol R. Flach. "Infrared reflection–absorption spectroscopy: Principles and applications to lipid–protein interaction in Langmuir films". In: *Biochimica et Biophysica Acta (BBA) - Biomembranes* 1798.4 (2010), 788–800. ISSN: 0005-2736. DOI: 10.1016/j.bbamem.2009.11.024. URL: http://dx.doi.org/10.1016/j.bbamem.2009.11.024.

[158] Richard Mendelsohn, Guangru Mao, and Carol R. Flach. "Infrared reflection–absorption spectroscopy: Principles and applications to lipid–protein interaction in Langmuir films". In: *Biochimica et Biophysica Acta (BBA) - Biomembranes* 1798.4 (2010), 788–800. ISSN: 0005-2736. DOI: 10.1016/j.bbamem.2009.11.024. URL: http://dx.doi.org/10.1016/j.bbamem.2009.11.024.

[159] Carol R. Flach et al. "Improved IRRAS Apparatus for Studies of Aqueous Monolayer Films: Determination of the Orientation of Each Chain in a Fatty-Acid Homogeneous Ceramide 2". In: *Applied Spectroscopy* 55.8 (2001), 1060–1066. ISSN: 1943-3530. DOI: 10.1366/0003702011952965. URL: http://dx.doi.org/10.1366/0003702011952965.

[160] Arne Gericke, Alexander V. Michailov, and Heinrich Hühnerfuss. "Polarized external infrared reflection-absorption spectrometry at the air/water interface: Comparison of experimental and theoretical results for different angles of incidence". In: *Vibrational Spectroscopy* 4.3 (1993), 335–348. ISSN: 0924-2031. DOI: 10.1016/0924-2031(93)80007-3. URL: http://dx.doi.org/10.1016/0924-2031(93)80007-3.

[161] Nobutaka Shioya et al. "Multiple-angle incidence resolution spectrometry: applications in nanoarchitectonics and applied physics". In: *Japanese Journal of Applied Physics* 63.6 (2024), p. 060102. ISSN: 1347-4065. DOI: 10.35848/1347-4065/ad4ad8. URL: http://dx.doi.org/10.35848/1347-4065/ad4ad8.

[162] Pierre-Alain Chollet, Jean Messier, and Charles Rosilio. "Infrared determination of the orientation of molecules in stearamide monolayers". In: *The Journal of Chemical Physics* 64.3 (1976), 1042–1050. ISSN: 1089-7690. DOI: 10.1063/1.432313. URL: http://dx.doi.org/10.1063/1.432313.

[163] Ralph T Yang et al. "Infrared study of adsorption in situ at the liquid/solid interface. IV. The utility of internal reflection techniques". In: *Journal of Colloid and Interface Science* 44.2 (1973), 249–258. ISSN: 0021-9797. DOI: 10.1016/0021-9797(73)90217-8. URL: http://dx.doi.org/10.1016/0021-9797(73)90217-8.

[164] N. J. Harrick and Paul Kirkpatrick. "Internal Reflection Spectroscopy". In: *American Journal of Physics* 36.10 (1968), 924–924. ISSN: 1943-2909. DOI: 10.1119/1.1974328. URL: http://dx.doi.org/10.1119/1.1974328.

[165] Timo I. Lotta et al. "Characterization of Langmuir-Blodgett films of 1,2-dipalmitoyl-sn-glycero-3-phosphatidylcholine and 1-palmitoyl-2-[10-(pyren-1-yl)decanoyl]-sn-glycero-3-phosphatidylcholine by FTIR-ATR". In: *Chemistry and Physics of Lipids* 46.1 (1988), 1–12. ISSN: 0009-3084. DOI: 10.1016/0009-3084(88)90107-7. URL: http://dx.doi.org/10.1016/0009-3084(88)90107-7.

[166] T. Buffeteau, B. Desbat, and J. M. Turlet. "Polarization Modulation FT-IR Spectroscopy of Surfaces and Ultra-Thin Films: Experimental Procedure and Quantitative Analysis". In: *Applied Spectroscopy* 45.3 (1991), 380–389. ISSN: 1943-3530. DOI: 10.1366/0003702914337308. URL: http://dx.doi.org/10.1366/0003702914337308.

[167] Yufang Xiao et al. "Polarisation modulation IRRAS investigation of a cellulose ether at the air/water interface". In: *Thin Solid Films* 327–329 (1998), 299–302. ISSN: 0040-6090. DOI: 10.1016/s0040-6090(98)00649-x. URL: http://dx.doi.org/10.1016/s0040-6090(98)00649-x.

[168] Alexander Michailov, Alexey Povolotskiy, and Vladimir Kuzmin. "Four-parameter model of thin surface layer contribution to reflectance-absorbance spectroscopy and ellipsometry". In: *Optics Express* 32.4 (2024), p. 6619. ISSN: 1094-4087. DOI: 10.1364/oe.514646. URL: http://dx.doi.org/10.1364/oe.514646.

[169] Carol R. Flach, Arne Gericke, and Richard Mendelsohn. "Quantitative Determination of Molecular Chain Tilt Angles in Monolayer Films at the Air/Water Interface: Infrared Reflection/Absorption Spectroscopy of Behenic Acid Methyl Ester". In: *The Journal of Physical Chemistry B* 101.1 (1997), 58–65. ISSN: 1520-5207. DOI: 10.1021/jp962288d. URL: http://dx.doi.org/10.1021/jp962288d.

[170] Carol R. Flach, Arne Gericke, and Richard Mendelsohn. "Quantitative Determination of Molecular Chain Tilt Angles in Monolayer Films at the Air/Water Interface: Infrared Reflection/Absorption Spectroscopy of Behenic Acid Methyl Ester". In: *The Journal of Physical Chemistry B* 101.1 (1997), 58–65. ISSN: 1520-5207. DOI: 10.1021/jp962288d. URL: http://dx.doi.org/10.1021/jp962288d.

[171] D. L. Allara, A. Baca, and C. A. Pryde. "Distortions of Band Shapes in External Reflection Infrared Spectra of Thin Polymer Films on Metal Substrates". In: *Macromolecules* 11.6 (1978), 1215–1220. ISSN: 1520-5835. DOI: 10.1021/ma60066a028. URL: http://dx.doi.org/10.1021/ma60066a028.

[172] James R. Chamberlain and Jeanne E. Pemberton. "Raman Spectroscopy of Langmuir Monolayers at the AirWater Interface". In: *Langmuir* 13.12 (1997), 3074–3079. ISSN: 1520-5827. DOI: 10.1021/la9609478. URL: http://dx.doi.org/10.1021/la9609478.

[173] Daniel Blaudez et al. “Infrared and Raman spectroscopies of monolayers at the air–water interface”. In: *Current Opinion in Colloid amp; Interface Science* 4.4 (1999), 265–272. ISSN: 1359-0294. DOI: 10.1016/s1359-0294(99)90006-7. URL: http://dx.doi.org/10.1016/s1359-0294(99)90006-7.

[174] G.G. Siu et al. “Surface-enhanced Raman spectroscopy of monolayer C60 Langmuir-Blodgett films”. In: *Thin Solid Films* 274.1–2 (1996), 147–149. ISSN: 0040-6090. DOI: 10.1016/0040-6090(95)07079-6. URL: http://dx.doi.org/10.1016/0040-6090(95)07079-6.

[175] Christy L. Haynes et al. “Surface-enhanced Raman sensors: early history and the development of sensors for quantitative biowarfare agent and glucose detection”. In: *Journal of Raman Spectroscopy* 36.6–7 (2005), 471–484. ISSN: 1097-4555. DOI: 10.1002/jrs.1376. URL: http://dx.doi.org/10.1002/jrs.1376.

[176] Hin Chu et al. “Surface Enhanced Raman Scattering Substrates Made by Oblique Angle Deposition: Methods and Applications”. In: *Coatings* 7.2 (2017), p. 26. ISSN: 2079-6412. DOI: 10.3390/coatings7020026. URL: http://dx.doi.org/10.3390/coatings7020026.

[177] Takeshi Kawai, Junzo Umemura, and Tohru Takenaka. “Non-resonance Raman studies on spread monolayers of stearic acid-d35 and cadmium stearate-d35 on water surfaces and thin LB films”. In: *Chemical Physics Letters* 162.3 (1989), 243–247. ISSN: 0009-2614. DOI: 10.1016/0009-2614(89)85132-2. URL: http://dx.doi.org/10.1016/0009-2614(89)85132-2.

[178] R. Griffith Freeman et al. “Self-Assembled Metal Colloid Monolayers: An Approach to SERS Substrates”. In: *Science* 267.5204 (1995), 1629–1632. ISSN: 1095-9203. DOI: 10.1126/science.267.5204.1629. URL: http://dx.doi.org/10.1126/science.267.5204.1629.

[179] M. Saint-Pierre Chazalet et al. “Surface-enhanced Raman scattering studies of lipid planar bilayers in water”. In: *Thin Solid Films* 244.1–2 (1994), 852–856. ISSN: 0040-6090. DOI: 10.1016/0040-6090(94)90585-1. URL: http://dx.doi.org/10.1016/0040-6090(94)90585-1.

[180] Chad L Leverette and Richard A Dluhy. "Vibrational characterization of a planar-supported model bilayer system utilizing surface-enhanced Raman scattering (SERS) and infrared reflection–absorption spectroscopy (IRRAS)". In: *Colloids and Surfaces A: Physicochemical and Engineering Aspects* 243.1–3 (2004), 157–167. ISSN: 0927-7757. DOI: 10.1016/j.colsurfa.2004.05.020. URL: http://dx.doi.org/10.1016/j.colsurfa.2004.05.020.

[181] R. Anchisini et al. "Resonance Raman Scattering in Langmuir-Blodgett Films of a New Phthalocyanine". In: *Molecular Crystals and Liquid Crystals Science and Technology. Section A. Molecular Crystals and Liquid Crystals* 235.1 (1993), 161–168. ISSN: 1058-725X. DOI: 10.1080/10587259308055189. URL: http://dx.doi.org/10.1080/10587259308055189.

[182] Michal Swierczewski and Thomas Bürgi. "Langmuir and Langmuir–Blodgett Films of Gold and Silver Nanoparticles". In: *Langmuir* 39.6 (2023), 2135–2151. ISSN: 1520-5827. DOI: 10.1021/acs.langmuir.2c02715. URL: http://dx.doi.org/10.1021/acs.langmuir.2c02715.

[183] Shujuan Huang et al. "Experimental conditions for a highly ordered monolayer of gold nanoparticles fabricated by the Langmuir–Blodgett method". In: *Journal of Vacuum Science amp; Technology B: Microelectronics and Nanometer Structures Processing, Measurement, and Phenomena* 19.6 (2001), 2045–2049. ISSN: 1520-8567. DOI: 10.1116/1.1410943. URL: http://dx.doi.org/10.1116/1.1410943.

[184] Somsubhra Saha et al. "Self-assembly of silver nanocolloids in the Langmuir–Blodgett Film of stearic acid: Evidence of an efficient SERS sensing platform". In: *Journal of Raman Spectroscopy* 47.2 (2015), 168–176. ISSN: 1097-4555. DOI: 10.1002/jrs.4771. URL: http://dx.doi.org/10.1002/jrs.4771.

[185] Joydeep Chowdhury, Somsubhra Saha, and Manash Ghosh. "Self-assembly of metal nanocolloids entrapped in Langmuir Blodgett Film templates: Evidence of efficient SERS sensing platforms". In: *Materials Today: Proceedings* 5.3 (2018), 10071–10076. ISSN: 2214-7853. DOI: 10.1016/j.matpr.2017.11.001. URL: http://dx.doi.org/10.1016/j.matpr.2017.11.001.

[186] Alina Mogilevsky and Raz Jelinek. "Gold Nanoparticle Self-Assembly in Two-Component Lipid Langmuir Monolayers". In: *Langmuir* 27.4 (2010), 1260–1268. ISSN: 1520-5827. DOI: 10.1021/la103718v. URL: http://dx.doi.org/10.1021/la103718v.

[187] Sukhananazerin Abdulla and Biji Pullithadathil. "Unidirectional Langmuir–Blodgett-Mediated Alignment of Polyaniline-Functionalized Multiwalled Carbon Nanotubes for NH3 Gas Sensor Applications". In: *Langmuir* 36.39 (2020), 11618–11628. ISSN: 1520-5827. DOI: 10.1021/acs.langmuir.0c02200. URL: http://dx.doi.org/10.1021/acs.langmuir.0c02200.

[188] Xiaolin Li et al. "LangmuiràíBlodgett Assembly of Densely Aligned Single-Walled Carbon Nanotubes from Bulk Materials". In: *Journal of the American Chemical Society* 129.16 (2007), 4890Àì4891. ISSN: 1520-5126. DOI: 10.1021/ja071114e. URL: http://dx.doi.org/10.1021/ja071114e.

[189] Xiaolin Li et al. "LangmuiràíBlodgett Assembly of Densely Aligned Single-Walled Carbon Nanotubes from Bulk Materials". In: *Journal of the American Chemical Society* 129.16 (2007), 4890Àì4891. ISSN: 1520-5126. DOI: 10.1021/ja071114e. URL: http://dx.doi.org/10.1021/ja071114e.

[190] A. Dhanabalan, A. Riul, and O.N. Oliveira. "Composite Langmuir–Blodgett (LB) films of polyaniline and cadmium stearate". In: *Supramolecular Science* 5.1–2 (1998), 75–81. ISSN: 0968-5677. DOI: 10.1016/s0968-5677(97)00071-0. URL: http://dx.doi.org/10.1016/s0968-5677(97)00071-0.

[191] Jongdeok Park et al. "Phase transitions and morphology control of Langmuir-Blodgett (LB) films of graphene oxide". In: *Journal of Colloid and Interface Science* 684 (2025), 215–224. ISSN: 0021-9797. DOI: 10.1016/j.jcis.2025.01.044. URL: http://dx.doi.org/10.1016/j.jcis.2025.01.044.

[192] Katharine L. Harrison, Laura B. Biedermann, and Kevin R. Zavadil. "Mechanical Properties of Water-Assembled Graphene Oxide Langmuir Monolayers: Guiding Controlled Transfer". In: *Langmuir* 31.36 (2015), 9825–9832. ISSN: 1520-5827. DOI: 10.1021/acs.langmuir.5b01994. URL: http://dx.doi.org/10.1021/acs.langmuir.5b01994.

[193] Masato Ito et al. “Hyper 100 °C Langmuir–Blodgett (Langmuir–Schaefer) Technique for Organized Ultrathin Film of Polymeric Semiconductors”. In: *Langmuir* 38.17 (2021), 5237–5247. ISSN: 1520-5827. DOI: 10.1021/acs.langmuir.1c02596. URL: http://dx.doi.org/10.1021/acs.langmuir.1c02596.

[194] Masanao Era et al. “Preparation of Highly Oriented Poly(p-phenylenevinylene) Thin Film by Using Langmuir–Blodgett Technique”. In: *Chemistry Letters* 17.7 (1988), 1097–1100. ISSN: 1348-0715. DOI: 10.1246/cl.1988.1097. URL: http://dx.doi.org/10.1246/cl.1988.1097.

[195] Rebeca da Rocha Rodrigues et al. “Conjugated polymers as Langmuir and Langmuir-Blodgett films: Challenges and applications in nanostructured devices”. In: *Advances in Colloid and Interface Science* 285 (2020), p. 102277. ISSN: 0001-8686. DOI: 10.1016/j.cis.2020.102277. URL: http://dx.doi.org/10.1016/j.cis.2020.102277.

[196] Niels Reitzel et al. “Self-Assembly of Conjugated Polymers at the Air/Water Interface. Structure and Properties of Langmuir and LangmuirBlodgett Films of Amphiphilic Regioregular Polythiophenes”. In: *Journal of the American Chemical Society* 122.24 (2000), 5788–5800. ISSN: 1520-5126. DOI: 10.1021/ja9924501. URL: http://dx.doi.org/10.1021/ja9924501.

[197] James R. Heath, Charles M. Knobler, and Daniel V. Leff. “Pressure/Temperature Phase Diagrams and Superlattices of Organically Functionalized Metal Nanocrystal Monolayers: The Influence of Particle Size, Size Distribution, and Surface Passivant”. In: *The Journal of Physical Chemistry B* 101.2 (1997), 189–197. ISSN: 1520-5207. DOI: 10.1021/jp9611582. URL: http://dx.doi.org/10.1021/jp9611582.

[198] Lucía Herrer, Santiago Martín, and Pilar Cea. “Nanofabrication Techniques in Large-Area Molecular Electronic Devices”. In: *Applied Sciences* 10.17 (2020), p. 6064. ISSN: 2076-3417. DOI: 10.3390/app10176064. URL: http://dx.doi.org/10.3390/app10176064.

[199] Harald Fuchs, Holger Ohst, and Werner Prass. “Ultrathin organic films: Molecular Architectures for advanced optical, electronic and bio-related systems”. In: *Advanced Materials* 3.1 (1991), 10–18. ISSN: 1521-4095. DOI: 10.1002/adma.19910030103. URL: http://dx.doi.org/10.1002/adma.19910030103.

[200] Soraya Sangiao et al. "All-Carbon Electrode Molecular Electronic Devices Based on Langmuir–Blodgett Monolayers". In: *Small* 13.7 (2016). ISSN: 1613-6829. DOI: 10.1002/smll.201603207. URL: http://dx.doi.org/10.1002/smll.201603207.

[201] Luz M. Ballesteros et al. "From an Organometallic Monolayer to an Organic Monolayer Covered by Metal Nanoislands: A Simple Thermal Protocol for the Fabrication of the Top Contact Electrode in Molecular Electronic Devices". In: *Advanced Materials Interfaces* 1.9 (2014). ISSN: 2196-7350. DOI: 10.1002/admi.201400128. URL: http://dx.doi.org/10.1002/admi.201400128.

[202] J. Paloheimo et al. "Molecular field-effect transistors using conducting polymer Langmuir–Blodgett films". In: *Applied Physics Letters* 56.12 (1990), 1157–1159. ISSN: 1077-3118. DOI: 10.1063/1.103182. URL: http://dx.doi.org/10.1063/1.103182.

[203] Guofeng Xu, Zhenan Bao, and John T. Groves. "LangmuiràíBlodgett Films of Regioregular Poly(3-hexylthiophene) as Field-Effect Transistors". In: *Langmuir* 16.4 (2000), 1834Äì1841. ISSN: 1520-5827. DOI: 10.1021/la9904455. URL: http://dx.doi.org/10.1021/la9904455.

[204] C. P. Collier et al. "Electronically Configurable Molecular-Based Logic Gates". In: *Science* 285.5426 (1999), 391–394. ISSN: 1095-9203. DOI: 10.1126/science.285.5426.391. URL: http://dx.doi.org/10.1126/science.285.5426.391.

[205] Antonio Riul et al. "Nano-Assembled Films for Taste Sensor Application". In: *Artificial Organs* 27.5 (2003), 469–472. ISSN: 1525-1594. DOI: 10.1046/j.1525-1594.2003.07243.x. URL: http://dx.doi.org/10.1046/j.1525-1594.2003.07243.x.

[206] Ludovico Valli. "Phthalocyanine-based Langmuir–Blodgett films as chemical sensors". In: *Advances in Colloid and Interface Science* 116.1–3 (2005), 13–44. ISSN: 0001-8686. DOI: 10.1016/j.cis.2005.04.008. URL: http://dx.doi.org/10.1016/j.cis.2005.04.008.

[207] Gabriele Giancane and Ludovico Valli. "State of art in porphyrin Langmuir–Blodgett films as chemical sensors". In: *Advances in Colloid and Interface Science* 171–172 (2012), 17–35. ISSN: 0001-8686. DOI: 10.1016/j.cis.2012.01.001. URL: http://dx.doi.org/10.1016/j.cis.2012.01.001.

[208] Piotr Nowak et al. “SnO2/TiO2 Thin Film n-n Heterostructures of Improved Sensitivity to NO2”. In: *Sensors* 20.23 (2020), p. 6830. ISSN: 1424-8220. DOI: 10.3390/s20236830. URL: http://dx.doi.org/10.3390/s20236830.

[209] Cheng Qian et al. “Facile preparation of self-assembled black phosphorus-based composite LB films as new chemical gas sensors”. In: *Colloids and Surfaces A: Physicochemical and Engineering Aspects* 608 (2021), p. 125616. ISSN: 0927-7757. DOI: 10.1016/j.colsurfa.2020.125616. URL: http://dx.doi.org/10.1016/j.colsurfa.2020.125616.

[210] Na Li et al. “Chemical gas sensor, surface enhanced Raman scattering and photoelectrics of composite Langmuir-Blodgett films consisting of polypeptide and dye molecules”. In: *Colloids and Surfaces A: Physicochemical and Engineering Aspects* 663 (2023), p. 131067. ISSN: 0927-7757. DOI: 10.1016/j.colsurfa.2023.131067. URL: http://dx.doi.org/10.1016/j.colsurfa.2023.131067.

[211] Inci Capan et al. “Sensing behaviors of lipophilic calix[4]arene phosphonate based Langmuir-Blodgett thin films for detection of volatile organic vapors”. In: *Sensors and Actuators A: Physical* 347 (2022), p. 113947. ISSN: 0924-4247. DOI: 10.1016/j.sna.2022.113947. URL: http://dx.doi.org/10.1016/j.sna.2022.113947.

[212] Ilya Gorbachev et al. “Langmuir-Blodgett Films of Arachidic and Stearic Acids as Sensitive Coatings for Chloroform HF SAW Sensors”. In: *Sensors* 23.1 (2022), p. 100. ISSN: 1424-8220. DOI: 10.3390/s23010100. URL: http://dx.doi.org/10.3390/s23010100.

[213] Tetsu Tatsuma et al. “Bifunctional Langmuir-Blodgett film for enzyme immobilization and amperometric biosensor sensitization”. In: *Thin Solid Films* 202.1 (1991), 145–150. ISSN: 0040-6090. DOI: 10.1016/0040-6090(91)90550-h. URL: http://dx.doi.org/10.1016/0040-6090(91)90550-h.

[214] Camila Gouveia Barbosa et al. “Copolymers and enzymes blended as LB films changing the bioelectronics properties of interfaces”. In: *Colloid and Interface Science Communications* 27 (2018), 40–44. ISSN: 2215-0382. DOI: 10.1016/j.colcom.2018.10.001. URL: http://dx.doi.org/10.1016/j.colcom.2018.10.001.

[215] Felipe Tejada Araujo, Laura Oliveira Peres, and Luciano Caseli. "Conjugated Polymers Blended with Lipids and Galactosidase as Langmuir–Blodgett Films To Control the Biosensing Properties of Nanostructured Surfaces". In: *Langmuir* 35.22 (2019), 7294–7303. ISSN: 1520-5827. DOI: 10.1021/acs.langmuir.9b00536. URL: http://dx.doi.org/10.1021/acs.langmuir.9b00536.

[216] Ilya Gorbachev et al. "Langmuir–Blodgett Films with Immobilized Glucose Oxidase Enzyme Molecules for Acoustic Glucose Sensor Application". In: *Sensors* 23.11 (2023), p. 5290. ISSN: 1424-8220. DOI: 10.3390/s23115290. URL: http://dx.doi.org/10.3390/s23115290.

[217] Hyeri Kim et al. "Spontaneous hybrids of graphene and carbon nanotube arrays at the liquid–gas interface for Li-ion battery anodes". In: *Chemical Communications* 54.41 (2018), 5229–5232. ISSN: 1364-548X. DOI: 10.1039/c8cc02148a. URL: http://dx.doi.org/10.1039/c8cc02148a.

[218] Sriramprabha Ramasamy, Ponpandian Nagamony, and Viswanathan Chinnuswamy. "Self-assembled SnO2/reduced graphene oxide nanocomposites via Langmuir-Blodgett technique as anode materials for Li-ion batteries". In: *Materials Letters* 218 (2018), 295–298. ISSN: 0167-577X. DOI: 10.1016/j.matlet.2018.01.177. URL: http://dx.doi.org/10.1016/j.matlet.2018.01.177.

[219] Hyun-Kyung Kim et al. "In Situ synthesis of Three-Dimensional Self-Assembled SnO2 - Reduced Graphene Oxide Architecture for Lithium Ion Batteries". In: *ECS Meeting Abstracts* MA2015-01.2 (2015), 573–573. ISSN: 2151-2043. DOI: 10.1149/ma2015-01/2/573. URL: http://dx.doi.org/10.1149/ma2015-01/2/573.

[220] Shuo Zhou et al. "Carbon Coating on Silicon for High-Performance Anode in Lithium-Ion Batteries". In: *ECS Meeting Abstracts* MA2021-01.2 (2021), 124–124. ISSN: 2151-2043. DOI: 10.1149/ma2021-012124mtgabs. URL: http://dx.doi.org/10.1149/ma2021-012124mtgabs.

[221] Shuo Zhou et al. "Highly Ordered Carbon Coating Prepared with Polyvinylidene Chloride Precursor for High-Performance Silicon Anodes in Lithium-Ion Batteries". In: *Batteries amp; Supercaps* 4.1 (2020), 240–247. ISSN: 2566-6223. DOI: 10.1002/batt.202000193. URL: http://dx.doi.org/10.1002/batt.202000193.

[222] Mun Sek Kim et al. "Langmuir–Blodgett artificial solid-electrolyte interphases for practical lithium metal batteries". In: *Nature Energy* 3.10 (2018), 889–898. ISSN: 2058-7546. DOI: 10.1038/s41560-018-0237-6. URL: http://dx.doi.org/10.1038/s41560-018-0237-6.

[223] Qing Ai et al. "Artificial Solid Electrolyte Interphase Coating to Reduce Lithium Trapping in Silicon Anode for High Performance Lithium-Ion Batteries". In: *Advanced Materials Interfaces* 6.21 (2019). ISSN: 2196-7350. DOI: 10.1002/admi.201901187. URL: http://dx.doi.org/10.1002/admi.201901187.

[224] Junhyeok Kim et al. "Controllable Solid Electrolyte Interphase in Nickel-Rich Cathodes by an Electrochemical Rearrangement for Stable Lithium-Ion Batteries". In: *Advanced Materials* 30.5 (2017). ISSN: 1521-4095. DOI: 10.1002/adma.201704309. URL: http://dx.doi.org/10.1002/adma.201704309.

[225] Jingnan Li et al. "Exploration of New Electrolyte Additive for Solid Electrolyte Interphase (SEI) Modification". In: *ECS Meeting Abstracts* MA2019-01.1 (2019), 43–43. ISSN: 2151-2043. DOI: 10.1149/ma2019-01/1/43. URL: http://dx.doi.org/10.1149/ma2019-01/1/43.

[226] Agnès P. Girard-Egrot and Loïc J. Blum. "Langmuir-Blodgett Technique for Synthesis of Biomimetic Lipid Membranes". In: *Nanobiotechnology of Biomimetic Membranes*. Springer US, 23–74. ISBN: 9780387377407. DOI: 10.1007/0-387-37740-9_2. URL: http://dx.doi.org/10.1007/0-387-37740-9_2.

[227] Cristina Stefaniu, Gerald Brezesinski, and Helmuth Möhwald. "Langmuir monolayers as models to study processes at membrane surfaces". In: *Advances in Colloid and Interface Science* 208 (2014), 197–213. ISSN: 0001-8686. DOI: 10.1016/j.cis.2014.02.013. URL: http://dx.doi.org/10.1016/j.cis.2014.02.013.

[228] Cristina Stefaniu, Gerald Brezesinski, and Helmuth Möhwald. "Langmuir monolayers as models to study processes at membrane surfaces". In: *Advances in Colloid and Interface Science* 208 (2014), 197–213. ISSN: 0001-8686. DOI: 10.1016/j.cis.2014.02.013. URL: http://dx.doi.org/10.1016/j.cis.2014.02.013.

[229] Gerald Brezesinski and Helmuth Möhwald. "Langmuir monolayers to study interactions at model membrane surfaces". In: *Advances in Colloid and Interface Science* 100–102 (2003), 563–584. ISSN: 0001-8686. DOI: 10.1016/s0001-8686(02)00071-4. URL: http://dx.doi.org/10.1016/s0001-8686(02)00071-4.
[230] Gerald Brezesinski and Helmuth Möhwald. "Langmuir monolayers to study interactions at model membrane surfaces". In: *Advances in Colloid and Interface Science* 100–102 (2003), 563–584. ISSN: 0001-8686. DOI: 10.1016/s0001-8686(02)00071-4. URL: http://dx.doi.org/10.1016/s0001-8686(02)00071-4.
[231] Thatyane M. Nobre et al. "Interactions of bioactive molecules amp; nanomaterials with Langmuir monolayers as cell membrane models". In: *Thin Solid Films* 593 (2015), 158–188. ISSN: 0040-6090. DOI: 10.1016/j.tsf.2015.09.047. URL: http://dx.doi.org/10.1016/j.tsf.2015.09.047.
[232] Thatyane M. Nobre et al. "Interactions of bioactive molecules amp; nanomaterials with Langmuir monolayers as cell membrane models". In: *Thin Solid Films* 593 (2015), 158–188. ISSN: 0040-6090. DOI: 10.1016/j.tsf.2015.09.047. URL: http://dx.doi.org/10.1016/j.tsf.2015.09.047.
[233] Xueying Guo and Wuge H. Briscoe. "Molecular interactions, elastic properties, and nanostructure of Langmuir bacterial-lipid monolayers: Towards solving the mystery in bacterial membrane asymmetry". In: *Current Opinion in Colloid amp; Interface Science* 67 (2023), p. 101731. ISSN: 1359-0294. DOI: 10.1016/j.cocis.2023.101731. URL: http://dx.doi.org/10.1016/j.cocis.2023.101731.

[234] Katarzyna Hac-Wydro et al. "Towards the understanding of the behavior of single-chained ether phospholipids in model biomembranes: Interactions with phosphatidylethanolamines in Langmuir monolayers". In: *Colloids and Surfaces B: Biointerfaces* 97 (2012), 162–170. ISSN: 0927-7765. DOI: 10.1016/j.colsurfb.2012.04.023. URL: http://dx.doi.org/10.1016/j.colsurfb.2012.04.023.

[235] André C. Machado and Luciano Caseli. "Interaction of nitrofurantoin with lipid langmuir monolayers as cellular membrane models distinguished with tensiometry and infrared spectroscopy". In: *Colloids and Surfaces B: Biointerfaces* 188 (2020), p. 110794. ISSN: 0927-7765. DOI:

10.1016/j.colsurfb.2020.110794. URL: http://dx.doi.org/10.1016/j.colsurfb.2020.110794.

[236] Anna Vikulina et al. "A lipid membrane supported on an artificial extracellular matrix made of polyelectrolyte multilayers: towards nanoarchitectonics at the cellular interface". In: *Nanoscale* 15.5 (2023), 2197–2205. ISSN: 2040-3372. DOI: 10.1039/d2nr05186a. URL: http://dx.doi.org/10.1039/d2nr05186a.

[237] Gero Decher. "Fuzzy Nanoassemblies: Toward Layered Polymeric Multicomposites". In: *Science* 277.5330 (1997), 1232–1237. ISSN: 1095-9203. DOI: 10.1126/science.277.5330.1232. URL: http://dx.doi.org/10.1126/science.277.5330.1232.

[238] Th. Geue et al. "Investigations of pH-dependent domain structure of lead arachidate Langmuir-Blodgett films by means of x-ray specular and diffuse scattering and atomic force microscopy". In: *The Journal of Chemical Physics* 110.16 (1999), 8104–8111. ISSN: 1089-7690. DOI: 10.1063/1.478713. URL: http://dx.doi.org/10.1063/1.478713.

[239] Kaushik Das and Sarathi Kundu. "Subphase pH induced monolayer to multilayer collapse of fatty acid Salt Langmuir monolayer at lower surface pressure". In: *Colloids and Surfaces A: Physicochemical and Engineering Aspects* 492 (2016), 54–61. ISSN: 0927-7757. DOI: 10.1016/j.colsurfa.2015.12.016. URL: http://dx.doi.org/10.1016/j.colsurfa.2015.12.016.

[240] E.M. El Mashak, F. Lakhdar-Ghazal, and J.F. Tocanne. "Effect of pH, mono- and divalent cations on the mixing of phosphatidylglycerol with phosphatidylcholine. A monolayer (, V) and fluorescence study". In: *Biochimica et Biophysica Acta (BBA) - Biomembranes* 688.2 (1982), 465–474. ISSN: 0005-2736. DOI: 10.1016/0005-2736(82)90358-3. URL: http://dx.doi.org/10.1016/0005-2736(82)90358-3.

[241] P.W.M van Dijck et al. "Comparative studies on the effects of pH and Ca2+ on bilayers of various negatively charged phospholipids and their mixtures with phosphatidylcholine". In: *Biochimica et Biophysica Acta (BBA) - Biomembranes* 512.1 (1978), 84–96. ISSN: 0005-2736. DOI: 10.1016/0005-2736(78)90219-5. URL: http://dx.doi.org/10.1016/0005-2736(78)90219-5.

[242] Raphael Zidovetzki, Azmi W. Atiya, and Hilde De Boeck. "Effect of Divalent Cations on the Structure of Dipalmitoylphosphatidylcholine and Phosphatidylcholine/Phosphatidylglycerol Bilayers: An2H-NMR Study". In: *Membrane Biochemistry* 8.3 (1989), 177–186. ISSN: 0149-046X. DOI: 10.3109/09687688909025830. URL: http://dx.doi.org/10.3109/09687688909025830.

[243] Aparna Rajagopal et al. "The effect of different metal cation incorporation in arachidic acid Langmuir-Blodgett (LB) monolayer films". In: *Applied Surface Science* 125.2 (1998), 178–186. ISSN: 0169-4332. DOI: 10.1016/s0169-4332(97)00391-7. URL: http://dx.doi.org/10.1016/s0169-4332(97)00391-7.

[244] A. Dhanabalan et al. "Variation of monolayer behaviour and molecular packing in zinc arachidate LB films with subphase pH". In: *Thin Solid Films* 327–329 (1998), 787–791. ISSN: 0040-6090. DOI: 10.1016/s0040-6090(98)00787-1. URL: http://dx.doi.org/10.1016/s0040-6090(98)00787-1.

[245] S. Kundu, A. Datta, and S. Hazra. "Effect of Metal Ions on Monolayer Collapses". In: *Langmuir* 21.13 (2005), 5894–5900. ISSN: 1520-5827. DOI: 10.1021/la0505770. URL: http://dx.doi.org/10.1021/la0505770.

[246] S. Bettarini et al. "Interactions between monolayers and metal ions at the water-air interface: conditions for the transferability as Langmuir-Blodgett multilayers". In: *Thin Solid Films* 210–211 (1992), 42–45. ISSN: 0040-6090. DOI: 10.1016/0040-6090(92)90163-6. URL: http://dx.doi.org/10.1016/0040-6090(92)90163-6.

[247] F. Peñacorada et al. "Monolayers and Multilayers of Uranyl Arachidate 2. Influence of the Subphase pH on the Structure and Stability of LangmuirBlodgett Films". In: *Langmuir* 12.5 (1996), 1351–1356. ISSN: 1520-5827. DOI: 10.1021/la950709x. URL: http://dx.doi.org/10.1021/la950709x.

[248] Sinzi Matuoka et al. "Stability and characterization of phospholipid Langmuir-Blodgett films". In: *Thin Solid Films* 180.1–2 (1989), 123–127. ISSN: 0040-6090. DOI: 10.1016/0040-6090(89)90063-1. URL: http://dx.doi.org/10.1016/0040-6090(89)90063-1.

[249] Tianshui Zheng et al. "Stability of Langmuir-Blodgett film/alumina, and Langmuir-Blodgett film/gold multilayer structures". In: *Thin Solid Films* 197.1–2 (1991), 327–333. ISSN: 0040-6090. DOI: 10.1016/0040-6090(91)90243-q. URL: http://dx.doi.org/10.1016/0040-6090(91)90243-q.

[250] M. S. Kim et al. "Fabricating multifunctional nanoparticle membranes by a fast layer-by-layer Langmuir–Blodgett process: application in lithium–sulfur batteries". In: *Journal of Materials Chemistry A* 4.38 (2016), 14709–14719. ISSN: 2050-7496. DOI: 10.1039/c6ta06018h. URL: http://dx.doi.org/10.1039/c6ta06018h.

[251] Yanju Wu et al. "Langmuir-Blodgett Assembly of Carboxylic Multi-walled Carbon Nanotubes-Nafion for Amperometric Sensing of Codeine". In: *Journal of The Electrochemical Society* 166.13 (2019), H592–H597. ISSN: 1945-7111. DOI: 10.1149/2.0371913jes. URL: http://dx.doi.org/10.1149/2.0371913jes.

[252] Bin Bai, Dong Wang, and Li-Jun Wan. "Synthesis of Covalent Organic Framework Films at Interfaces". In: *Bulletin of the Chemical Society of Japan* 94.3 (2021), 1090–1098. ISSN: 1348-0634. DOI: 10.1246/bcsj.20200391. URL: http://dx.doi.org/10.1246/bcsj.20200391.

[253] Katsuhiko Ariga, Yuri Lvov, and Gero Decher. "There is still plenty of room for layer-by-layer assembly for constructing nanoarchitectonics-based materials and devices". In: *Physical Chemistry Chemical Physics* 24.7 (2022), 4097–4115. ISSN: 1463-9084. DOI: 10.1039/d1cp04669a. URL: http://dx.doi.org/10.1039/d1cp04669a.

[254] Dongmok Whang et al. "Large-Scale Hierarchical Organization of Nanowire Arrays for Integrated Nanosystems". In: *Nano Letters* 3.9 (2003), 1255–1259. ISSN: 1530-6992. DOI: 10.1021/nl0345062. URL: http://dx.doi.org/10.1021/nl0345062.

[255] Katsuhiko Ariga, Jonathan P. Hill, and Qingmin Ji. "Layer-by-layer assembly as a versatile bottom-up nanofabrication technique for exploratory research and realistic application". In: *Physical Chemistry Chemical Physics* 9.19 (2007), p. 2319. ISSN: 1463-9084. DOI: 10.1039/b700410a. URL: http://dx.doi.org/10.1039/b700410a.

[256] Omar Azzaroni et al. "Field-effect transistors engineered via solution-based layer-by-layer nanoarchitectonics". In: *Nanotechnology* 34.47 (2023), p. 472001. ISSN: 1361-6528. DOI: 10.1088/1361-6528/acef26. URL: http://dx.doi.org/10.1088/1361-6528/acef26.

[257] Mitsuru Akashi and Takami Akagi. "Composite Materials by Building Block Chemistry Using Weak Interaction". In: *Bulletin of the Chemical Society of Japan* 94.7 (2021), 1903–1921. ISSN: 1348-0634. DOI: 10.1246/bcsj.20210089. URL: http://dx.doi.org/10.1246/bcsj.20210089.

[258] Yuri Lvov, Katsuhiko Ariga, and Toyoki Kunitake. "Layer-by-Layer Assembly of Alternate Protein/Polyion Ultrathin Films". In: *Chemistry Letters* 23.12 (1994), 2323–2326. ISSN: 1348-0715. DOI: 10.1246/cl.1994.2323. URL: http://dx.doi.org/10.1246/cl.1994.2323.

[259] Samuel H. Gyepi-Garbrah and Roberta Šilerová. "The first direct comparison of self-assembly and Langmuir–Blodgett deposition techniques: Two routes to highly organized monolayers". In: *Physical Chemistry Chemical Physics* 4.14 (2002), 3436–3442. ISSN: 1463-9084. DOI: 10.1039/b201698b. URL: http://dx.doi.org/10.1039/b201698b.

[260] Syed Arshad Hussain et al. "Unique supramolecular assembly through Langmuir – Blodgett (LB) technique". In: *Heliyon* 4.12 (2018), e01038. ISSN: 2405-8440. DOI: 10.1016/j.heliyon.2018.e01038. URL: http://dx.doi.org/10.1016/j.heliyon.2018.e01038.

[261] Markus Retsch et al. "Fabrication of Large-Area, Transferable Colloidal Monolayers Utilizing Self-Assembly at the Air/Water Interface". In: *Macromolecular Chemistry and Physics* 210.3–4 (2009), 230–241. ISSN: 1521-3935. DOI: 10.1002/macp.200800484. URL: http://dx.doi.org/10.1002/macp.200800484.

[262] Luzhu Xu et al. "Continuous Langmuir–Blodgett Deposition and Transfer by Controlled Edge-to-Edge Assembly of Floating 2D Materials". In: *Langmuir* 35.1 (2018), 51–59. ISSN: 1520-5827. DOI: 10.1021/acs.langmuir.8b03173. URL: http://dx.doi.org/10.1021/acs.langmuir.8b03173.

[263] F. W. Embs, H. H. Winter, and G. Wegner. "Langmuir-Blodgett multilayer assembly by a continuous process using a steadily flowing subphase". In: *Langmuir* 9.7 (1993), 1618–1621. ISSN: 1520-5827.

DOI: 10.1021/la00031a003. URL: http://dx.doi.org/10.1021/la00031a003.

[264] Nobuhiro GEMMA and Makoto AZUMA. "Analyses on the transfer process of LB films." In: *Hyomen Kagaku* 11.4 (1990), 229–234. ISSN: 1881-4743. DOI: 10.1380/jsssj.11.229. URL: http://dx.doi.org/10.1380/jsssj.11.229.

[265] Andrzej Boguta et al. "Characterization of interfacial effects in organic macrocycles Langmuir and Langmuir–Blodgett layers studied by surface potential and FT–IR spectroscopy examination". In: *Materials Science and Engineering: B* 113.1 (2004), 99–105. ISSN: 0921-5107. DOI: 10.1016/j.mseb.2004.07.005. URL: http://dx.doi.org/10.1016/j.mseb.2004.07.005.

[266] Didier Jacquemain et al. "Two-Dimensional Crystallography of Amphiphilic Molecules at the Air–Water Interface". In: *Angewandte Chemie International Edition in English* 31.2 (1992), 130–152. ISSN: 0570-0833. DOI: 10.1002/anie.199201301. URL: http://dx.doi.org/10.1002/anie.199201301.

[267] James P. Bareman and Michael L. Klein. "Molecular Dynamics Simulation of A Langmuir Monolayer". In: *MRS Proceedings* 237 (1991). ISSN: 1946-4274. DOI: 10.1557/proc-237-271. URL: http://dx.doi.org/10.1557/proc-237-271.

[268] Katsuhiko Ariga. "Nanoarchitectonics Revolution and Evolution: From Small Science to Big Technology". In: *Small Science* 1.1 (2020). ISSN: 2688-4046. DOI: 10.1002/smsc.202000032. URL: http://dx.doi.org/10.1002/smsc.202000032.

[269] Katsuhiko Ariga and Rawil Fakhrullin. "Materials Nanoarchitectonics from Atom to Living Cell: A Method for Everything". In: *Bulletin of the Chemical Society of Japan* 95.5 (2022), 774–795. ISSN: 1348-0634. DOI: 10.1246/bcsj.20220071. URL: http://dx.doi.org/10.1246/bcsj.20220071.

[270] Katsuhiko Ariga. "Molecular nanoarchitectonics: unification of nanotechnology and molecular/materials science". In: *Beilstein Journal of Nanotechnology* 14 (2023), 434–453. ISSN: 2190-4286. DOI: 10.3762/bjnano.14.35. URL: http://dx.doi.org/10.3762/bjnano.14.35.

[271] Helmut Ringsdorf, Bernhard Schlarb, and Joachim Venzmer. "Molecular Architecture and Function of Polymeric Oriented Systems: Models for the Study of Organization, Surface Recognition, and Dynamics of Biomembranes". In: *Angewandte Chemie International Edition in English* 27.1 (1988), 113–158. ISSN: 0570-0833. DOI: 10.1002/anie.198801131. URL: http://dx.doi.org/10.1002/anie.198801131.

[272] Katsuhiko Ariga. "Interfaces Working for Biology: Solving Biological Mysteries and Opening Up Future Nanoarchitectonics". In: *ChemNanoMat* 2.5 (2016), 333–343. ISSN: 2199-692X. DOI: 10.1002/cnma.201600053. URL: http://dx.doi.org/10.1002/cnma.201600053.

[273] Katsuhiko Ariga, Masaki Ishii, and Taizo Mori. "2D Nanoarchitectonics: Soft Interfacial Media as Playgrounds for Microobjects, Molecular Machines, and Living Cells". In: *Chemistry – A European Journal* 26.29 (2020), 6461–6472. ISSN: 1521-3765. DOI: 10.1002/chem.202000789. URL: http://dx.doi.org/10.1002/chem.202000789.

[274] Mutsuyoshi MATSUMOTO and Yasujiro KAWABATA. "Recent Progress in Langmuir-Blodgett Films". In: *Journal of the Japan Society of Colour Material* 61.7 (1988), 385–393. ISSN: 0010-180X. DOI: 10.4011/shikizai1937.61.385. URL: http://dx.doi.org/10.4011/shikizai1937.61.385.

[275] Jin Young Park and Rigoberto C. Advincula. "Nanostructuring polymers, colloids, and nanomaterials at the air–water interface through Langmuir and Langmuir–Blodgett techniques". In: *Soft Matter* 7.21 (2011), p. 9829. ISSN: 1744-6848. DOI: 10.1039/c1sm05750b. URL: http://dx.doi.org/10.1039/c1sm05750b.

[276] Rajkumar Sunil Singh. "Langmuir and Langmuir–Blodgett films of aromatic amphiphiles". In: *Soft Materials* 20.1 (2021), 57–98. ISSN: 1539-4468. DOI: 10.1080/1539445x.2021.1922443. URL: http://dx.doi.org/10.1080/1539445x.2021.1922443.

[277] A. Angelova et al. "Control of the structure of Langmuir-Blodgett films of a discotic liquid crystalline compound via the subphase composition and the adjacent molecular environment". In: *Thin Solid Films* 242.1–2 (1994), 289–294. ISSN: 0040-6090. DOI: 10.1016/0040-6090(94)90547-9. URL: http://dx.doi.org/10.1016/0040-6090(94)90547-9.

[278] HANS RIEGLER and KARL SPRATTE. “On-Line Structure Control of Langmuir-Blodgett Films”. In: *Organic Thin Films and Surfaces: Directions for the Nineties*. Elsevier, 1995, 349–364. DOI: 10.1016/b978-0-12-523485-6.50023-7. URL: http://dx.doi.org/10.1016/b978-0-12-523485-6.50023-7.

[279] Jack Y. Josefowicz et al. “Structure of Langmuir-Blodgett Films of Disk-Shaped Molecules Determined by Atomic Force Microscopy”. In: *Science* 260.5106 (1993), 323–326. ISSN: 1095-9203. DOI: 10.1126/science.260.5106.323. URL: http://dx.doi.org/10.1126/science.260.5106.323.

[280] E. Meyer et al. “Molecular-resolution images of Langmuir–Blodgett films using atomic force microscopy”. In: *Nature* 349.6308 (1991), 398–400. ISSN: 1476-4687. DOI: 10.1038/349398a0. URL: http://dx.doi.org/10.1038/349398a0.

[281] E. Meyer et al. “Molecular-resolution images of Langmuir–Blodgett films using atomic force microscopy”. In: *Nature* 349.6308 (1991), 398–400. ISSN: 1476-4687. DOI: 10.1038/349398a0. URL: http://dx.doi.org/10.1038/349398a0.

[282] Chengshan Wang et al. “Infrared Reflectionâˆ’Absorption Spectroscopy and Polarization-Modulated Infrared Reflectionâˆ’Absorption Spectroscopy Studies of the Aequorin Langmuir Monolayer”. In: *The Journal of Physical Chemistry B* 112.13 (2008), 4146â€“4151. ISSN: 1520-5207. DOI: 10.1021/jp710953j. URL: http://dx.doi.org/10.1021/jp710953j.

S11 Paper generated for Nuclear Magnetic Ressonance (NMR) using Stratey 2 (58 input papers)

Nuclear Magnetic Resonance in the Oil and Gas Industry: Unveiling Subsurface Complexity Through Advanced Characterization and Emerging Technologies

June 27, 2025

Contents

1 Introduction **2**

2 Fundamentals of Nuclear Magnetic Resonance **6**
2.1 Relaxation Mechanisms 8
2.2 Pulse Sequences 11
2.3 Mathematical Background and Signal Processing 13

3 NMR Applications in Petrophysics **14**
3.1 Porosity Determination 15
3.2 Pore Size Distribution 16
3.3 Permeability Estimation 19
3.4 Wettability and Fluid Typing 22

4 NMR in Enhanced Oil Recovery (EOR) Monitoring **24**
4.1 Chemical EOR Monitoring 26
4.2 Gas Injection EOR Monitoring 27
4.3 Thermal EOR Monitoring 28
4.4 Techniques for Saturation and Fluid Distribution 28
4.5 Advantages and Limitations of NMR in EOR 32

5 NMR in Unconventional Reservoirs **33**
5.1 Shale Characterization 33
5.2 Tight Gas Sands 37
5.3 Heavy Oil and Oil Sands 38

6 Advanced NMR Techniques and Future Trends **39**
6.1 Multi-Dimensional NMR 40
6.2 High-Field NMR 43
6.3 NMR Imaging (MRI) 45
6.4 Future Directions in NMR Research 47

7 Conclusion **49**

Abstract

Nuclear Magnetic Resonance (NMR) has emerged as an indispensable, non-destructive analytical technique for characterizing porous media and fluid behavior across the oil and gas industry. This comprehensive survey reviews the fundamental principles of NMR, elucidating its diverse applications from laboratory core analysis to field-scale operations. We detail its pivotal role in petrophysical characterization, including the quantification of porosity, pore size distribution, permeability, and fluid saturation, and its unique ability to differentiate fluid phases and assess wettability. The paper highlights NMR's significant contributions to Enhanced Oil Recovery (EOR) monitoring, providing granular insights into fluid displacement and residual oil saturation, and its critical application in challenging unconventional reservoirs for detailed pore structure and fluid typing. Furthermore, its integration into Logging While Drilling (LWD) operations for real-time formation evaluation is discussed. While offering unparalleled insights, NMR applications in complex geological systems face challenges such as internal magnetic field gradients, pore coupling effects, and data interpretation complexities in low-porosity formations. Addressing these, we explore future directions in NMR research, including the optimization of pulse sequences, the integration of advanced computational methods like Machine Learning and Artificial Intelligence for data interpretation, the development of more efficient LWD hardware, and the necessity for multi-modal data integration and reservoir-representative measurement conditions. This review underscores NMR's continued evolution as a transformative tool, driving advancements in hydrocarbon exploration, development, and production optimization.

1 Introduction

Nuclear Magnetic Resonance (NMR), a highly significant and versatile analytical tool rooted in the fundamental quantum mechanical property of nuclear spin, offers unparalleled, non-destructive insights into the molecular structure, dynamics, and environment of materials [1, 2, 3]. By detecting the magnetic response of atomic nuclei when placed in a strong static magnetic field and perturbed by radiofrequency pulses, NMR provides unique information often inaccessible through other analytical methods, making it indispensable for both fundamental research and industrial applications. Its ability to probe the microscopic properties of matter, particularly the behavior of hydrogen-containing fluids, has cemented its role in diverse fields ranging from medical imaging, where it underpins Magnetic Resonance Imaging (MRI) [4], to materials science and chemical analysis [1, 4], and in the oil and gas industry [5, 6, 7, 8]. Figure 1 illustrates a fundamental aspect of NMR data acquisition and processing: the transformation of raw Carr-Purcell-Meiboom-Gill (CPMG) signals into T2 relaxation spectra [9, 10, 11, 12]. While this specific example is from a biological sample (sea cucumber), the underlying principles of acquiring decaying echo trains and inverting them to obtain a distribution of relaxation times are universally applicable across all NMR measurements, including those performed in the oil and gas industry for characterizing porous media and fluids.

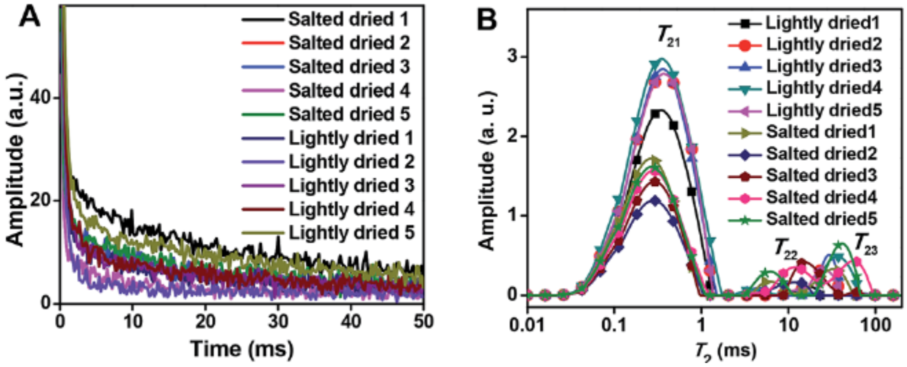

Figure 1: The CPMG signals (A) and 72 relaxation spectra (B) acquired from the raw data obtained for lightly dried and salted dried sea cucumber. Adapted from Hriberšek, Matej, 2024 [13].

In the realm of the oil and gas industry, NMR has emerged as a transfor-

mative technology, revolutionizing the way reservoirs are characterized and fluids are analyzed, both in laboratory settings and at the field scale. Its application in earth sciences historically dates back decades, with its principles being employed in proton-precession magnetometers for over 50 years and in borehole and core exploration tools for over 40 years. The development of early nuclear magnetism logging (NML) tools, which responded solely to formation fluids, marked a significant advancement. As shown in Figure 2, these tools provided continuous 'Free Fluid Index' (FFI) logs. The FFI represents the volume of mobile fluids (typically water and hydrocarbons) that can be freely produced from the formation, distinguishing them from irreducible bound fluids. Comparing the FFI curve with core-analysis porosity and conventional logs, as depicted, demonstrated the early utility of NML in directly identifying fluid-bearing zones and estimating effective porosity, offering insights into permeability and wettability that were less directly obtainable from other logs. The inherent relationship between the NMR signal and the hydrogen nuclei present in formation fluids (oil, water, and gas) allows for direct measurements of critical petrophysical properties that are crucial for effective hydrocarbon exploration, production, and reservoir management. Unlike conventional logging tools that often rely on indirect measurements influenced by lithology, NMR offers direct information about the pore structure and fluid properties, independent of the rock matrix itself. This distinct advantage enables a more accurate and comprehensive understanding of complex porous media, which is paramount for optimizing recovery strategies and assessing reservoir potential.

The profound importance of NMR in the oil and gas sector necessitates a thorough understanding of its fundamental principles and diverse applications. Accurate determination of petrophysical properties such as porosity, pore size distribution, fluid saturation, permeability, capillary pressure, and wettability is vital for evaluating oil and gas reservoirs and precisely estimating reserves and potential recovery approaches. Figure 3 exemplifies how NMR data can be used to derive pore size distributions, often in conjunction with Mercury Intrusion Porosimetry (MICP). By correlating the NMR T2 relaxation time distribution, which is sensitive to pore geometry, with the direct pore throat size measurements from MICP on a subset of samples, a robust relationship can be established. This enables the non-destructive and efficient determination of pore size distribution for other samples from the same formation, providing crucial insights into the reservoir's storage capacity and fluid flow characteristics. Low-field NMR, in particular, has

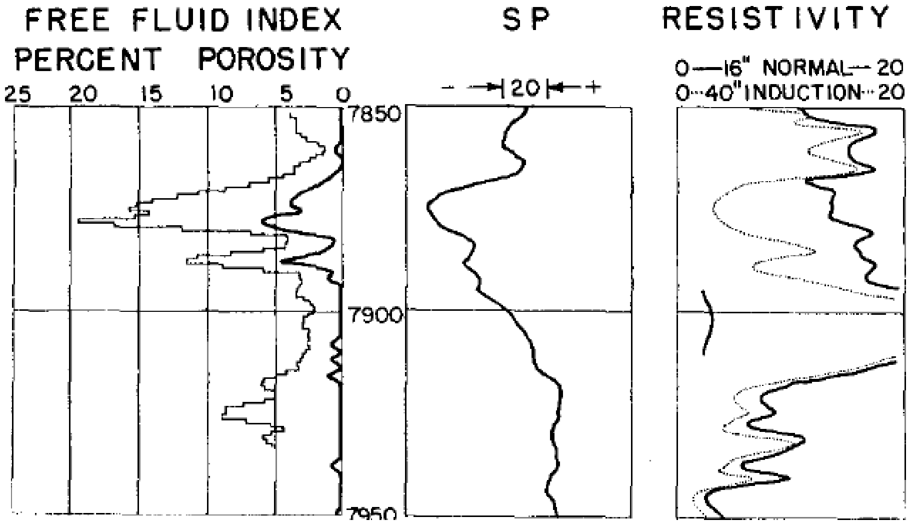

Figure 2: Comparison oF Free Fiuip Inpex (Smoora Curve) To Core-Analysis Porosity and to conventional Locs TurovucH Suaty Lime Sectton. Adapted from Brown, R.J.S., et al., 1960 [14].

proven its robustness by offering capabilities for both field-scale logging and detailed laboratory-scale core analysis. This dual capability allows for reliable cross-validation between core data and logging measurements, enhancing the overall accuracy of reservoir characterization. Furthermore, NMR's sensitivity to rock-fluid interactions makes it an invaluable tool for evaluating Enhanced Oil Recovery (EOR) techniques, providing a more detailed assessment of fluid recovery from different pore systems compared to macroscopic techniques that only yield bulk recovery data. The continuous advancements in NMR technology, including the development of advanced pulse sequences and improved hardware for logging while drilling (LWD) operations, continue to expand its utility and impact within the industry. A comprehensive grasp of these aspects is essential for leveraging NMR to its full potential in tackling the complex challenges associated with modern hydrocarbon exploration and production, especially in increasingly complex and unconventional reservoirs.

This survey aims to provide a comprehensive overview of Nuclear Magnetic Resonance technology and its extensive applications within the oil and gas industry. The subsequent sections will delve into various facets of NMR, building from its foundational principles to its most advanced applications. Section 2 will establish the theoretical groundwork, explaining the

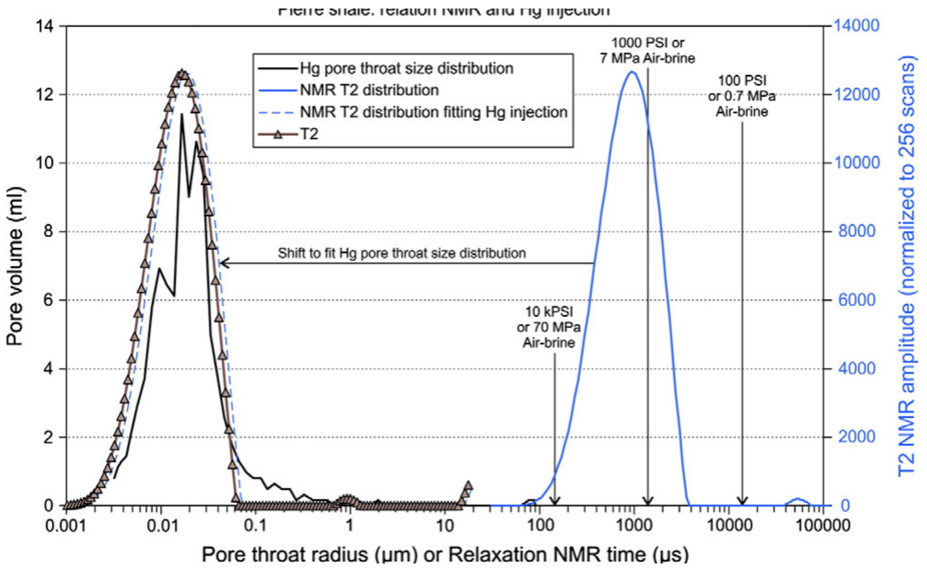

Figure 3: Pore size distribution from NMR and MICP from Pierre Shale: The MICP curve (solid black line) can be used to shift the original NMR T¿ time relaxation distribution (solid blue curve), into alignment with a quantitative measure of pore size. The time abscissa axis from the original NMR T¿ distribution becomes a pore throat size abscissa axis by this shifting method. This shift of time for NMR on one sample can then be applied in all the NMR data on a group of samples from the same formation to access the pore throat size distribution with a minimal number of destructive and time consuming MICP measurements. Adapted from Josh, M., et al., 2012 [15].

core physics governing NMR phenomena and the different relaxation mechanisms. Following this, Section 3 will explore the diverse applications of NMR in petrophysical characterization, detailing how it is used to determine key reservoir properties such as porosity, pore size distribution, permeability, fluid saturation, and wettability. Section 4 will then focus on NMR's crucial role in monitoring and evaluating Enhanced Oil Recovery (EOR) processes, highlighting its ability to track fluid displacement and assess recovery efficiency. The application of NMR in the challenging domain of unconventional reservoirs, including shale oil and gas, will be discussed in Section 5, emphasizing its unique capabilities in characterizing tight formations. Finally, Section 6 will examine advanced NMR techniques, such as multi-dimensional NMR and magnetic resonance imaging, and discuss future research directions and

potential advancements in the field, before concluding the survey in Section 7.

2 Fundamentals of Nuclear Magnetic Resonance

Nuclear Magnetic Resonance (NMR) stands as a powerful analytical technique, fundamentally rooted in the quantum mechanical properties of atomic nuclei. The core principle of NMR hinges on the inherent magnetism of certain nuclei, which possess both a nuclear spin angular momentum and a magnetic moment. These properties are quantified by two critical constants: the nuclear spin quantum number, J, and the gyromagnetic ratio, γ. A nucleus is considered "NMR active" if its nuclear spin quantum number, J, is greater than zero. While a high gyromagnetic ratio, γ, is beneficial for achieving a strong detectable signal, the fundamental criterion for NMR activity is the non-zero nuclear spin. In the context of oil and gas applications, hydrogen nuclei, or protons (^{1}H), are predominantly the species of interest due to their high natural abundance in formation fluids and favorable NMR properties [5, 14, 7, 6]. Other common elements found in geological formations, such as carbon, oxygen, magnesium, silicon, sulfur, and calcium, largely consist of isotopes that lack a magnetic moment or spin, rendering them NMR inactive. Even elements like potassium, iron, sodium, and aluminum, which may possess weak magnetic moments, typically offer low detection efficiency in typical NMR logging scenarios. Consequently, the NMR signal in these applications primarily originates from hydrogen nuclei present in the fluid phase within porous media.

Figure 4 provides a schematic overview of a typical NMR apparatus, highlighting key components such as the static magnet, RF coils, and detection system, which are essential for generating and detecting the NMR signal.

The fundamental concept of an NMR experiment involves placing a sample containing these NMR-active nuclei within a strong, static external magnetic field, conventionally denoted as B_z [4, 3]. In this magnetic field, the nuclear spins align either parallel or anti-parallel to the field, creating a net macroscopic magnetization. This net magnetization, proportional to the total number of spins within the sampled volume, is in thermal equilibrium with the static field. The nuclei also undergo a precessional motion around

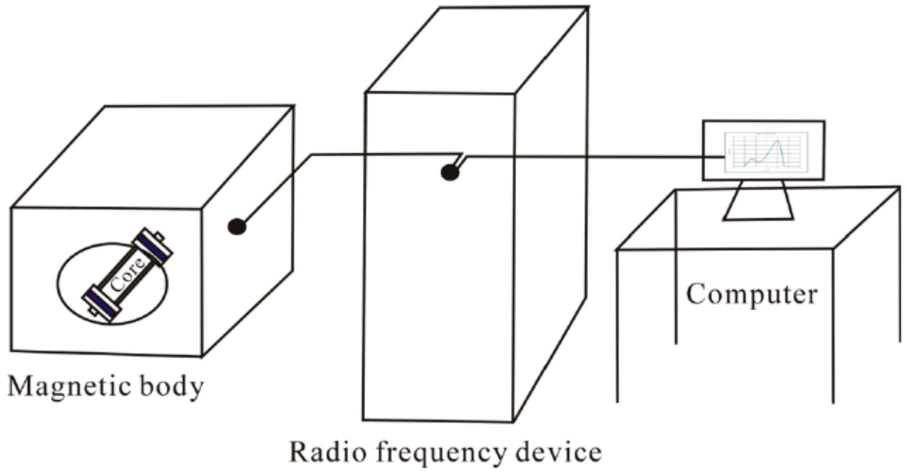

Figure 4: Schematic of the NMR apparatus used for the NMR test. Adapted from Gao, Hui, et al., 2015 [16].

the B_z field, akin to a spinning top in a gravitational field. The frequency of this precession, known as the Larmor frequency (f), is directly proportional to the strength of the static magnetic field and the gyromagnetic ratio of the nucleus, as described by the Larmor equation:

$$f = \frac{\gamma}{2\pi} B_z \tag{1}$$

where f is the Larmor frequency (typically in MHz), γ is the gyromagnetic ratio of the nucleus (in MHz/T), and B_z is the strength of the static magnetic field (in T). To induce a detectable NMR signal, a second, oscillating magnetic field, B_1, in the form of radio-frequency (RF) pulses, is applied perpendicular to the static B_z field. This B_1 field, when applied at the Larmor frequency, excites the nuclear spins, tipping the net magnetization away from its equilibrium alignment into the transverse ($x - y$) plane. Upon removal of the RF pulse, the excited spins begin to precess coherently in the transverse plane, generating a detectable oscillating signal. This process marks the initiation of the relaxation phenomenon, where the nuclear spins return to their equilibrium state, and the decay of this oscillating signal provides crucial information about molecular dynamics, adsorption, confinement, and pore characteristics within porous media [6, 17, 18, 19].

2.1 Relaxation Mechanisms

The return of the nuclear magnetization to its equilibrium state after excitation is characterized by two distinct relaxation processes: longitudinal relaxation (T_1) and transverse relaxation (T_2). These relaxation times are highly sensitive probes of the molecular environment and interactions within a sample.

Longitudinal relaxation, also known as spin-lattice relaxation, describes the exponential recovery of the magnetization component parallel to the static magnetic field (B_z) back to its thermal equilibrium value. This process involves the exchange of energy between the spin system and its surrounding thermal environment, or "lattice," which includes the molecular motion of the fluid and the solid matrix. The recovery of longitudinal magnetization, M_z, over time, t_I, can be expressed as:

$$\frac{M_z(t_I)}{M_0} = 1 - 2e^{-t_I/T_1} \tag{2}$$

where M_0 is the equilibrium magnetization. The T_1 relaxation is influenced by the statistical likelihood of magnetic interactions between a proton and other nearby magnetic entities, including the pore walls. For fluids confined within porous media, T_1 values are typically shorter in smaller pores and longer in larger pores, reflecting the extent of interaction with the pore surfaces [20, 17, 21, 22].

Transverse relaxation, or spin-spin relaxation, describes the irreversible decay of the magnetization in the transverse ($x - y$) plane due to the dephasing of individual nuclear spins [23, 24, 25]. This dephasing arises from spin-spin interactions and local magnetic field inhomogeneities experienced by each nucleus. The decay of transverse magnetization, M_{xy}, can be described by:

$$\frac{M_{xy}(t)}{M_0} = e^{-t/T_2} \tag{3}$$

In homogeneous bulk liquids, T_1 and T_2 are often similar. However, in porous media, T_2 is invariably shorter than T_1 because it is additionally affected by static magnetic field gradients inherent in the sample, which cause faster dephasing [26, 27, 28, 29].

Several factors intricately affect both longitudinal and transverse relaxation times. Molecular motion and fluid viscosity play a significant role, as described by the Bloembergen-Purcell-Pound (BPP) theory [23, 30, 31].

Generally, higher viscosity or reduced molecular mobility leads to shorter relaxation times [32, 33, 30]. When liquid molecules adsorb onto a solid surface, their mobility is restricted, consequently enhancing relaxation and leading to reduced T_1 and T_2 values [18, 34, 35, 36].

Surface interactions are particularly critical in porous media. NMR relaxation in such systems is often modeled by a biphasic fast-exchange mechanism, where fluid molecules rapidly exchange between a surface-adsorbed layer and the bulk fluid within the pore [20, 31, 30, 17]. The observed relaxation rate ($1/T_{obs}$) is a weighted average of the bulk fluid relaxation rate ($1/T_{bulk}$) and the surface relaxation rate ($1/T_{surface}$):

$$\frac{1}{T_{obs}} = P\frac{1}{T_{surface}} + (1-P)\frac{1}{T_{bulk}} \tag{4}$$

where P represents the population fraction of the adsorbed surface layer. This population can be related to the pore structure's surface-to-volume ratio (S/V) by $P = \delta(S/V)$, where δ is the characteristic thickness of the adsorbed layer. This general form accounts for contributions from both surface and bulk fluid environments. For situations where fluid molecules diffuse rapidly between the surface and bulk regions within a pore (known as the "fast diffusion limit") [37, 17], and the surface relaxation rate significantly dominates over the bulk fluid relaxation rate, Equation (4) simplifies to:

$$\frac{1}{T_{obs}} \approx \rho_s\frac{S}{V} \tag{5}$$

Here, ρ_s is the surface relaxivity, a crucial parameter that quantifies the ability of the pore wall to enhance relaxation and provides a direct link between the observed relaxation rate and pore size [38, 21, 39, 12]. Thus, in water-saturated rocks, shorter T_1 and T_2 values indicate smaller pores due to the increased S/V ratio [40, 41, 42, 22].

Paramagnetic substances and internal magnetic field gradients also profoundly influence relaxation [26, 43, 29, 44]. Paramagnetic ions, often present on pore surfaces, possess unpaired electrons with very large magnetic moments (the electron gyromagnetic ratio is approximately 650 times that of a proton). These strong local magnetic fields significantly accelerate relaxation, especially T_2 [43, 45, 46, 44]. Furthermore, magnetic susceptibility differences between the solid rock matrix and the fluid phases induce internal magnetic field gradients within the pores. These gradients cause additional dephasing

of nuclear spins, leading to a further reduction in T_2 (but not T_1) [26, 27, 29, 47]. The effective transverse relaxation time, T_2^E, in the presence of such inhomogeneities, is given by:

$$\frac{1}{T_2^E} = \frac{1}{T_2} + \frac{1}{12}\gamma^2 G^2 D t_e^2 \tag{6}$$

Here, the $1/T_2$ term in the denominator represents the transverse relaxation rate in the absence of internal magnetic field gradients, encompassing contributions from both bulk fluid relaxation and surface relaxation [48, 29, 28, 49]. Furthermore, G is the internal magnetic field gradient, D is the diffusion coefficient, and t_e is the echo time. The effects of static field inhomogeneities can be mitigated through specific pulse sequences, enabling the measurement of the true T_2 [48, 27, 50, 29]. The ratio T_1/T_2 can also provide valuable insights into pore structure, mineralogy, and even the strength of surface adsorption interactions [18, 43, 51, 36].

2.2 Pulse Sequences

The manipulation and detection of nuclear magnetization in NMR experiments are achieved through precisely timed sequences of RF pulses, known as pulse sequences. These sequences are designed to selectively excite, evolve, and detect the NMR signal, allowing the extraction of specific physical parameters.

The most fundamental detection method is the Free Induction Decay (FID) [14, 52, 53], which involves applying a 90-degree RF pulse to tip the magnetization into the transverse plane, followed by the direct observation of the decaying signal as the spins dephase. While simple, FID is highly sensitive to magnetic field inhomogeneities [26, 54, 27, 55].

To measure T_1 relaxation, the Inversion Recovery (IR) pulse sequence is commonly employed [56, 22, 12, 57]. This sequence begins with a 180-degree RF pulse that inverts the equilibrium longitudinal magnetization. The system is then allowed to recover for a variable inversion time, t_I. Following this recovery period, a 90-degree RF pulse is applied to flip the recovering longitudinal magnetization into the transverse plane, where the signal amplitude is detected. By varying t_I and measuring the corresponding signal amplitude, the exponential recovery curve can be fitted to determine the T_1 relaxation time.

For T_2 measurements, the Carr-Purcell-Meiboom-Gill (CPMG) pulse sequence is the standard [58, 59, 60]. This sequence effectively mitigates the dephasing effects caused by static magnetic field inhomogeneities [58]. The CPMG sequence starts with a 90-degree RF pulse that tips the magnetization into the transverse plane. This is followed by a series of 180-degree refocusing pulses, each separated by an echo time (t_e). Each 180-degree pulse inverts the phases of the spins, causing them to rephase and form spin echoes at times $2t_e$, $4t_e$, and so on, after the initial 90-degree pulse [53, 58]. The amplitude of these spin echoes decays exponentially due to true T_2 relaxation and other irreversible dephasing mechanisms [9, 27]. The CPMG sequence allows for the accurate determination of T_2 by fitting the envelope of these echo amplitudes [61, 9].

$$M_{xy}(i \cdot t_e) = M_0 e^{-(i \cdot t_e)/T_2} \tag{7}$$

where i is the echo number.

Beyond these basic sequences, advanced two-dimensional (2D) NMR techniques offer more comprehensive insights by correlating different NMR parameters [62, 57, 63]. These methods typically involve encoding one parameter in an indirect dimension and detecting another in a direct dimension. For instance, $T_1 - T_2$ correlation, achieved by combining an IR component followed by a CPMG train, provides a 2D probability density map showing combinations of T_1 and T_2 values [64, 11, 65]. This map is particularly useful for differentiating fluid types and pore sizes [62, 66, 50, 12]. The acquired signal for $T_1 - T_2$ correlation can be expressed as:

$$M(t_I, t_e) = \sum_{j=1}^{N_1} \sum_{k=1}^{N_2} F(T_{1j}, T_{2k})(1 - 2e^{-t_I/T_{1j}})e^{-t_e/T_{2k}} \tag{8}$$

where $F(T_{1j}, T_{2k})$ is the 2D distribution function.

Another powerful 2D technique is $T_2 - D$ correlation, which maps transverse relaxation time against the diffusion coefficient. This is typically achieved by combining a Pulsed Field Gradient Spin Echo (PGSE) or Adiabatic Pulsed Gradient Spin Echo (APGSTE) sequence for diffusion encoding with a CPMG sequence for T_2 detection. The $T_2 - D$ maps are invaluable for robustly differentiating oil, gas, and water signals in complex fluid mixtures. The signal acquired from such a sequence is given by:

$$S(g, t_e) = \sum_{j=1}^{N_1} \sum_{k=1}^{N_2} F(D_j, T_{2k}) e^{-D_j \gamma^2 g^2 \delta^2 (\Delta - \delta/3)} e^{-t_e/T_{2k}} \tag{9}$$

where g is the magnetic field gradient strength, δ is the duration of the gradient pulse, and Δ is the diffusion time.

Finally, T_2-T_2 exchange spectroscopy, achieved by two CPMG echo trains separated by a mixing time (Δ_{mixing}), is used to study diffusive and chemical exchange processes between different pore environments. The appearance of off-diagonal peaks in the 2D map signifies exchange, with their intensity changes over varying Δ_{mixing} providing information on the rate and extent of this exchange. The acquired 2D data for this sequence is given by:

$$S(t_e^{(1)}, t_e^{(2)}, \Delta_{mixing}) = \sum_{j=1}^{N_1} \sum_{k=1}^{N_2} F(T_{2j}^{(1)}, T_{2k}^{(2)}) e^{-t_e^{(1)}/T_{2j}^{(1)}} e^{-t_e^{(2)}/T_{2k}^{(2)}} \tag{10}$$

These advanced sequences, particularly 2D NMR, are computationally intensive but, with techniques like Singular Value Decomposition (SVD) and kernel separability, have become tractable on standard computing platforms.

2.3 Mathematical Background and Signal Processing

The raw NMR signal acquired in the time domain represents the decay or recovery of magnetization. To extract meaningful physical parameters such as relaxation time distributions (spectra), these time-domain signals must be transformed and processed. The underlying mathematical framework for this transformation is often based on the Fredholm integral equation of the first kind:

$$\phi(t) = \int K(t, T) F(T) dT + \epsilon(t) \tag{11}$$

Here, $\phi(t)$ represents the experimentally acquired signal, $F(T)$ is the desired distribution of relaxation times (e.g., T_1 or T_2), $K(t, T)$ is the kernel function describing the expected exponential decay or growth based on the specific pulse sequence, and $\epsilon(t)$ accounts for experimental noise. For T_1 measurements, the kernel typically represents an exponential growth, while for T_2 it represents an exponential decay.

To solve for the distribution $F(T)$, the integral equation is typically discretized and rewritten in a vector-matrix form:

$$M = KF + \epsilon \tag{12}$$

where M is the acquired data vector, K is the kernel matrix, F is the target probability distribution vector, and ϵ is the noise vector. The goal is to find

an F that minimizes the difference $||M - KF||^2$. However, this problem is inherently "ill-posed" in the presence of noise, meaning that small variations in the input data can lead to large, unphysical oscillations in the solution, and an infinite number of solutions may exist.

To obtain a stable and physically meaningful solution, regularization techniques are applied. Tikhonov regularization is a widely used method that adds a penalty term to the minimization function, promoting smoothness in the solution:

$$\min ||M - KF||^2 + \alpha||F||^2 \tag{13}$$

The parameter α, known as the smoothing parameter, controls the trade-off between fitting the data faithfully and ensuring the smoothness of the solution. Its optimal value is often determined using robust algorithms like generalized cross-validation (GCV). Additionally, physical constraints, such as non-negativity of the distribution $F(T)$ and defining a sensible range for relaxation times (e.g., 10^{-4} to 10^4 seconds), are incorporated into the inversion process.

For 2D NMR data, the computational complexity significantly increases, as the kernel matrix can become exceptionally large. To overcome this, data compression techniques like Singular Value Decomposition (SVD) are employed to reduce the dimensionality of the problem by identifying and discarding less significant data components. Furthermore, the concept of kernel separability ($K = K_1 \otimes K_2$) is leveraged for 2D measurements, where the relaxation characteristics (e.g., T_1 and T_2) occur at separable time scales. This allows SVD to be performed on the individual components, making the 2D inversion problem manageable on standard computing systems.

Beyond inversion, general data processing techniques are crucial for improving the quality of NMR measurements. Enhancing the signal-to-noise ratio (SNR) is paramount for accurate measurements. This is often achieved by repeating the measurement cycle multiple times and stacking (averaging) the acquired signals, which causes the random noise to average out while the coherent NMR signal accumulates. These sophisticated mathematical and signal processing approaches are vital for transforming raw NMR data into interpretable distributions, enabling a deeper understanding of fluid properties and porous media characteristics.

3 NMR Applications in Petrophysics

Nuclear Magnetic Resonance (NMR) technology has emerged as an indispensable tool in the field of petrophysics, offering non-invasive and comprehensive characterization of reservoir rocks and their contained fluids [67, 6, 68, 69]. This section explores the fundamental applications of NMR in determining critical petrophysical properties, including porosity [70, 71, 72, 73], pore size distribution [22, 21, 12, 74], permeability [70, 7, 8, 75], and the assessment of wettability [76, 35, 77, 78] and fluid types [79, 80, 81, 82]. The insights derived from NMR measurements are crucial for effective reservoir evaluation, modeling, and optimizing hydrocarbon recovery strategies [67, 7, 8, 83].

3.1 Porosity Determination

Porosity, defined as the fraction of void space within a rock, represents the primary storage capacity of a reservoir and is a fundamental petrophysical property. NMR measurements are highly effective for quantifying porosity because the technique primarily detects hydrogen nuclei present in the fluids saturating the rock pores. When a rock sample is fully saturated with a single fluid, such as water, the magnitude of the detected NMR signal is directly proportional to the total pore volume of the rock, thereby providing a direct measure of its porosity. The accuracy and reliability of NMR porosity measurements are well-established, as demonstrated by comparisons with conventional methods. For instance, 5 illustrates the strong agreement between NMR-derived porosity values and shipboard moisture and density (MAD) measurements across various sites, validating NMR as a precise technique for quantifying the total pore volume.

In heterogeneous carbonate reservoirs, which often exhibit a wide range of pore sizes from sub-micron to centimeter-scale vugs (Hidajat et al. 2004), NMR can effectively capture the total pore volume. However, the accuracy of NMR total porosity measurements is influenced by several experimental and fluid-specific factors. These include the echo spacing (τ_e), which is the time between successive 180-degree radiofrequency pulses in a Carr-Purcell-Meiboom-Gill (CPMG) sequence; the strength of the static magnetic field (B_0); the hydrogen index (HI) of the fluid within the pores, which quantifies the hydrogen density relative to pure water; the repetition time (RT) between successive pulse sequences; and the rock temperature. Careful consideration

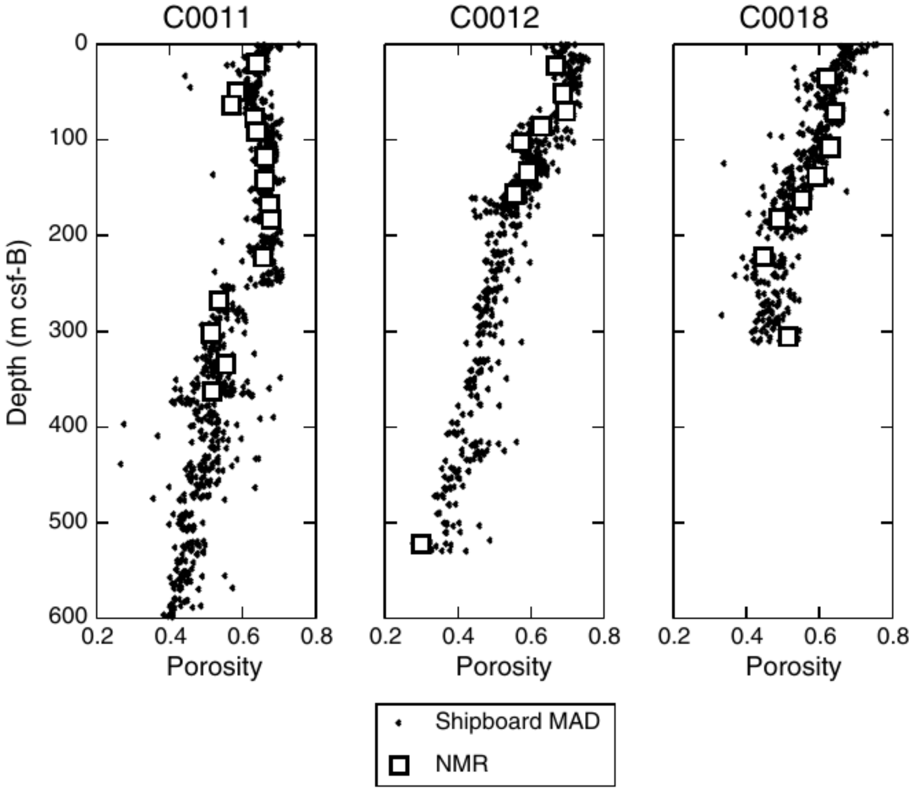

Figure 5: Comparison of NMR porosity values with shipboard moisture and density (MAD) measurements for Sites C0011, C0012, and C0018. The NMR measurements match the MAD data very well. Adapted from Daigle, Hugh, et al., 2014 [84].

and calibration of these parameters are essential to ensure the precision of porosity measurements.

When multiple fluid phases, such as oil, water, and gas, coexist within the pore space, NMR can differentiate and quantify the saturation of each phase [50, 12, 35, 66]. This is achieved by leveraging the distinct NMR signatures of different fluids, which often vary in their relaxation times and diffusion coefficients [35, 62, 85, 82]. The initial amplitude of the recorded echo signal is indicative of the total amount of hydrogen-containing fluids in the sample, from which the overall fluid saturation and total porosity can be derived [20, 7, 6, 86]. For instance, in an air-water system, as water-filled pores are emptied and replaced by air (which contains no hydrogen nuclei), the

NMR signal amplitude decreases, reflecting the loss of hydrogen-containing fluid. This sensitivity to fluid content makes NMR an invaluable tool for understanding fluid distribution and saturation profiles within complex pore systems [12, 50, 87, 67].

3.2 Pore Size Distribution

One of the most significant applications of NMR in petrophysics is its ability to provide detailed information about the pore size distribution (PSD) within porous media. The transverse relaxation time (T_2) of fluids confined within pores is intimately related to the pore geometry, specifically the surface-to-volume ratio (S/V) of the pores. Smaller pores, with their higher S/V ratios, lead to faster surface relaxation and thus shorter T_2 relaxation times, while larger pores correspond to longer T_2 times. This fundamental relationship allows the conversion of measured T_2 relaxation time distributions into pore size distributions. This relationship is conceptually illustrated in 6, which depicts how shorter T_2 times correspond to smaller pores (e.g., micropores or clay-bound water), while longer T_2 times are indicative of larger pores (e.g., macropores or free fluids).

The general equation governing the transverse relaxation rate ($1/T_2$) in porous media is given by:

$$\frac{1}{T_2} = \frac{1}{T_{2,\text{bulk}}} + \rho_s \left(\frac{S}{V}\right) + \frac{D\gamma^2 G^2 \tau_e^2}{12} \tag{14}$$

Here, $T_{2,\text{bulk}}$ represents the bulk fluid relaxation time, ρ_s is the surface relaxivity constant, S/V is the surface-to-volume ratio of the pores, D is the confined fluid diffusion coefficient, γ is the gyromagnetic ratio of the proton nuclei, G is the internal magnetic field gradient, and τ_e is the echo time. In many practical scenarios, particularly at low magnetic fields and short echo times, the bulk relaxation component (often very long, typically greater than 3000 ms) and the diffusion relaxation component (which can be minimized by reducing τ_e and performing experiments at low magnetic fields) become negligible [34, 27, 29, 26]. In such cases, the relaxation rate is dominated by surface interactions, simplifying the equation to $1/T_2 \approx \rho_s(S/V)$. For idealized spherical pores, $S/V = 3/r$, where r is the pore radius. This leads to a direct proportionality between T_2 and pore radius: $T_2 = C \cdot r$, where $C = 1/(3\rho_s)$ is a conversion factor [17, 21, 12, 40].

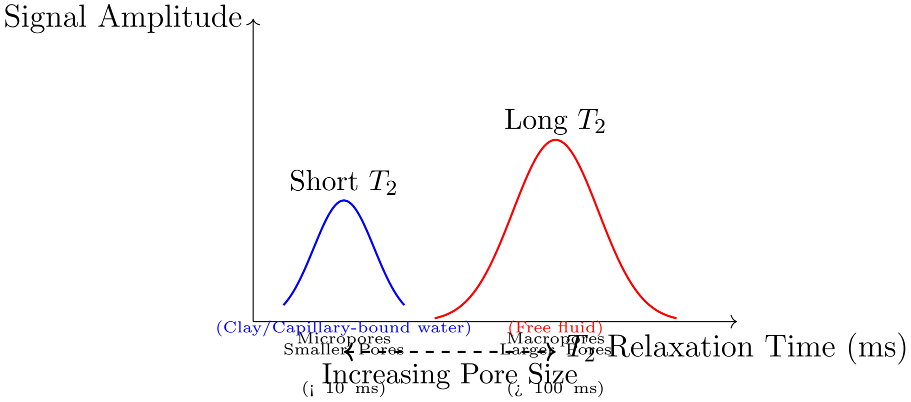

Figure 6: Conceptual illustration of NMR T_2 relaxation time distribution and its relationship to pore size. Shorter T_2 times correspond to smaller pores (e.g., micropores or clay-bound water), while longer T_2 times are indicative of larger pores (e.g., macropores or free fluids).

Despite its utility, challenges exist in accurately deriving PSD from NMR T_2 distributions. One significant issue is pore diffusive coupling, especially prevalent in pores smaller than 1 μm or in multi-modal pore systems like carbonates with distinct macro- and micropores (Fleury and Romero-Sarmiento, 2016)[88, 89, 90, 42]. In such cases, fluid molecules can diffuse between pores of different sizes during the relaxation measurement, leading to a single average relaxation time that does not accurately reflect the true PSD. Another major hurdle is the insufficient knowledge of the rock's surface relaxivity (ρ_s). While it is often assumed to be constant for a given sample, research indicates that ρ_s can vary within a sample (Arns et al. 2006; Zhao et al. 2020a)[37, 39, 26], impacting the accuracy of the T_2-to-pore-size conversion. Extensive efforts, including the integration of micro-computed tomography (μCT) imaging with NMR, have been made to better evaluate surface relaxivity and achieve accurate overlap between Mercury Intrusion Porosimetry (MIP) and NMR relaxation distributions (Benavides et al. 2020; Connolly et al. 2019; Luo et al. 2015)[91, 92, 93, 94].

The T_2 spectrum itself can be decomposed into multiple independent component spectra, typically ranging from two to five, by fitting the distribution with Gaussian functions on a logarithmic T_2 axis [95, 41, 72, 96]. Each component spectrum can be assigned petrophysical significance, representing different pore types and fluid states [6, 93]. Common components include

clay-bound water, capillary-bound fluid, and fluids within micropores and macropores [97, 98, 93, 42]. This decomposition provides a more granular understanding of the complex pore structures, which is particularly beneficial for characterizing tight sandstones and other unconventional reservoirs where pore connectivity and fluid mobility are critical [93, 99, 100, 101].

3.3 Permeability Estimation

Permeability, representing the ease with which fluids flow through a porous medium, is a crucial property for reservoir performance prediction. The basic principle of NMR application in rock permeability determination is the relationship that exists between NMR relaxation times and the pore geometry. While NMR does not directly measure fluid flow, it provides static petrophysical properties such as porosity and pore geometry, which can be empirically linked to permeability (Yang et al. 2019). This indirect estimation makes NMR a valuable tool for determining permeability both in laboratory core analysis and through downhole logging tools, enabling in-situ formation permeability estimation.

Several empirical correlations and models have been developed to estimate permeability from NMR measurements [6, 75, 71, 102]. One of the earliest approaches was presented by Seevers (1966), who combined the Kozeny equation for permeability with NMR relaxation times [20, 70]. Subsequent research by Banavar and Schwartz (1987), and Kenyon et al. (1988), further established relationships between permeability, NMR relaxation times, and porosity, often expressed in the general form [103, 104, 56, 105]:

$$k = a\phi^n T_2^m \tag{15}$$

where k is permeability, ϕ is porosity, T_2 is the transverse relaxation time (often the geometric mean T_2), and a, n, and m are empirically derived constants.

Two widely recognized NMR-based permeability models are the Timur-Coates (T-C) model [75] and the Schlumberger Doll Research (SDR) model [104, 105]. The Timur model (Timur 1969), building upon the principle that all pores contribute to fluid transport based on their surface-to-volume ratio [20, 22, 21], is commonly expressed as:

$$k = C_T\phi^4(T_{2,GM})^2 \tag{16}$$

where C_T is an empirical constant and $T_{2,GM}$ is the geometric mean of the T_2 distribution.

The Coates et al. model (Coates et al., 1991 [75, 101]), often referred to as the Free Fluid Index (FFI) model, relates permeability to the ratio of movable fluids to irreducible fluids. It is formulated as:

$$k = C_C \phi^4 \left(\frac{FFI}{BVI} \right)^2 \tag{17}$$

where C_C is an empirical parameter, FFI is the free movable water (bulk volume movable, BVM), and BVI is the bulk volume irreducible (non-producible fluids) [20, 98, 106, 72]. The ratio BVI/BVM serves as a measure of the specific internal pore surface [20, 31, 12].

Another frequently employed model is the SDR model:

$$k = C_{SDR} \phi^2 T_{2,GM}^2 \tag{18}$$

where C_{SDR} is an empirical constant.

The practical application and comparison of these models are critical for accurate permeability prediction. For example, 7 presents a comparison of NMR-derived hydraulic conductivity calculated using both the SDR and Timur-Coates equations. It highlights how these models, when calibrated with optimized empirical constants, can provide reliable estimates of hydraulic conductivity, which are essential for reservoir flow simulations and production forecasting. Both the T-C and SDR equations require empirically determined constants, which are typically calibrated through laboratory studies on consolidated materials to yield reliable permeability estimates in reservoir settings.

Despite the widespread use of these models, several factors can affect the accuracy of NMR-derived permeability. Most correlations assume a constant surface relaxivity for rock samples, which may not hold true across heterogeneous formations, necessitating corrections for variations in ρ_s. Furthermore, these empirical models are primarily based on porosity-permeability relationships, which can be misleading in cases where high porosity does not translate to high permeability due to poor pore connectivity or small pore throats. Wettability of the porous medium also significantly influences permeability estimation (Ji et al. 2020; Elsayed et al. 2021a). To enhance the accuracy of NMR-derived permeability, it is crucial to calibrate the model parameters $(a, n, m, C_T, C_C, C_{SDR})$ to local reservoir data and to consider

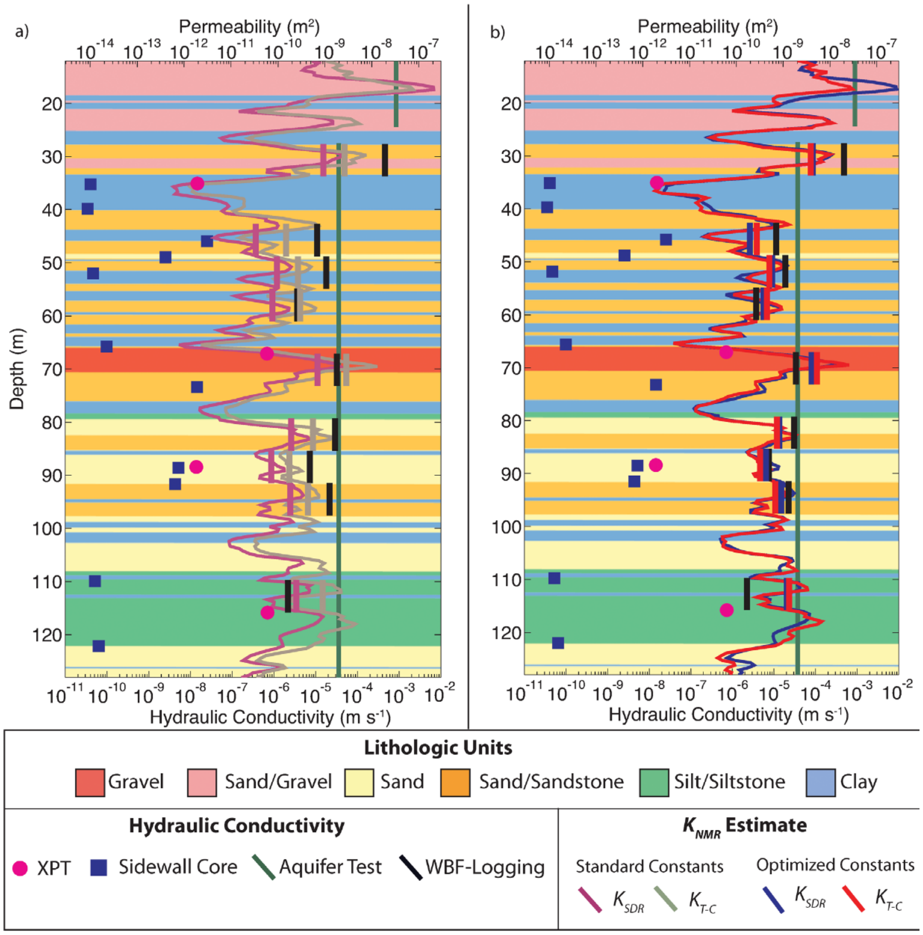

Figure 7: (a) NMR-derived hydraulic conductivity calculated using the SDR and T-C equations with the standard empirical constants and a cutoff time of 33 ms. The K pr estimates are shown in purple and the Ky.c estimates are shown in gray. The Kymr estimates are upscaled so that they can be compared with Kwer-togging estimates. (b) NMR-derived hydraulic conductivity calculated using the SDR and T-C equations with the optimized empirical constants and a cutoff time of 33 ms. The optimized K pp esti- mates are shown in blue and the optimized Ky.c estimates are shown in red. Adapted from Dlubac, Katherine, et al., 2013 [107].

the NMR T_2 cutoff values that differentiate between movable and irreducible fluids, rather than relying solely on the bulk T_2 distribution. Incorporating

additional parameters that account for pore throat size and pore connectivity can further improve the predictive capabilities of these models.

3.4 Wettability and Fluid Typing

The wetting state of a reservoir rock, which describes the preference of the rock surface for one fluid over another, is a critical parameter influencing fluid distribution, flow dynamics, and ultimately, oil recovery efficiency. NMR offers robust capabilities for determining wettability indices and identifying fluid types (oil, water, and gas) within porous media, which is invaluable for characterizing mixed-wet and oil-wet reservoirs.

The interpretation of NMR T_2 distributions is pivotal for understanding the wetting state, based on the assumption of a relationship between pore throat and pore body sizes. This interpretation requires a thorough understanding of the fluids and rock properties involved. NMR relaxation times (T_1 and T_2) are sensitive not only to the size of the pores but also to the types of fluids present and the mineralogy of the pore walls (Kleinberg et al. 1994). This sensitivity allows NMR to differentiate between various fluid phases and their distribution within the pore network.

For fluid typing, the T_2 spectrum is particularly informative. As previously noted in the Porosity Determination subsection, the initial amplitude of the recorded echo signal directly indicates the total amount of hydrogen-containing fluids in a rock sample (Isah et al. 2021a, 2021b), enabling the calculation of overall fluid saturation and porosity. When a rock is fully saturated, the T_2 spectrum typically exhibits its highest amplitude, with larger pores corresponding to longer relaxation times. In contrast, during desaturation processes, such as air-water displacement, the amplitude of the longer T_2 components decreases as hydrogen-containing fluids are replaced by air (which yields no NMR signal) (Coates et al. 1997; Howard et al. 1995; Toumelin et al. 2002). This direct correlation between signal amplitude, relaxation time, and fluid content enables the precise determination of fluid saturation.

Advanced NMR techniques, such as two-dimensional (2D) NMR maps, significantly enhance the ability to characterize fluid distribution and proton mobility. For instance, 8 demonstrates the utility of 2D NMR mappings in characterizing shales of different maturities, showcasing how these advanced techniques can reveal subtle differences in pore structure and fluid behavior that are critical for understanding reservoir quality in unconventional plays.

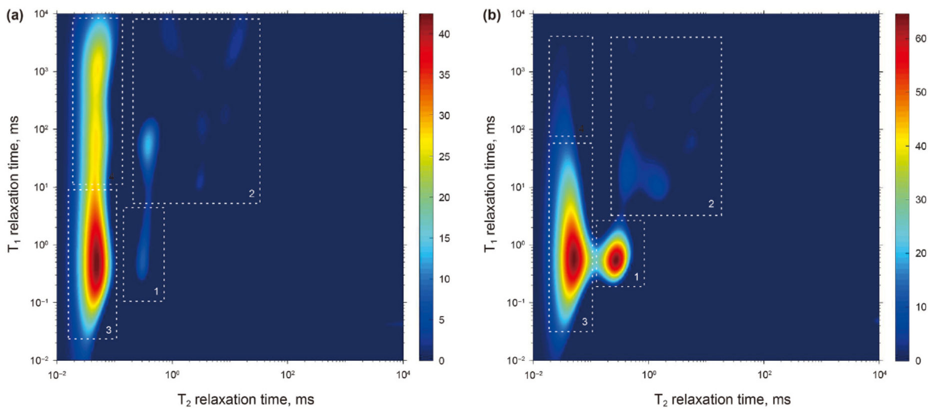

Figure 8: Comparison of NMR 2-D mappings of shales in low-medium maturity (a) and high maturity (b). (a) the sample from the Chang 7 member in the Ordos Basin, R, = 0.8%, TOC = 11.47%, clay mineral content = 43.4%; (b) the sample from the Qingshankou Formation in the Songliao Basin, Rp = 1.3%, TOC = 1.97%, clay mineral content = 52%. Adapted from Zhang, Jing-Ya, et al., 2023 [108].

$T_1 - T_2$ maps, for example, correlate longitudinal and transverse relaxation times, providing unique signatures for different fluids and their interactions with the pore surface (Habina et al. 2017; Tinni et al. 2015). The signal from these maps corresponds to hydrogen nuclei from the movable liquid fraction, making them useful for investigating both conventional and unconventional rocks. Similarly, T_2-Diffusion ($T_2 - D$) maps improve fluid separation compared to T_2 relaxation alone, leading to more accurate calculations of effective surface relaxivities of fluids (Flaum et al. 2005; Liang et al. 2019; Minh et al. 2015).

The T_1/T_2 ratio is widely used for qualitatively assessing wettability (Katika et al. 2017; Valori et al. 2017; J. Wang et al. 2018a, b). The principle is based on the observation that molecules in bulk, non-viscous fluids exhibit fast, isotropic motion, resulting in a T_1/T_2 ratio close to unity. However, as molecular motion becomes slower or anisotropic, such as in highly viscous fluids or fluids strongly interacting with pore surfaces (i.e., wetting fluids), the T_1/T_2 ratio tends to be greater than one. This makes the T_1/T_2 ratio a better predictor of wettability, especially when diffusion relaxation is significant. A notable challenge with this technique arises when dealing with heavy oils, particularly those containing asphaltenes, as their inherent high

viscosity can yield T_1/T_2 ratios greater than one, potentially complicating wettability interpretation (Valori et al. 2017; Valori and Nicot 2019). Nevertheless, NMR holds great potential for in-situ wettability evaluation (Valori et al. 2018), with ongoing research employing simulation tools to investigate NMR responses in multiphase rock core conditions (Al-Muthana et al. 2012; Looyestijn 2008; Mohnke et al. 2015; Wang et al. 2018a, b).

Furthermore, as discussed in the Pore Size Distribution subsection, the decomposition of the T_2 spectrum into distinct components provides a powerful method for identifying fluid properties (Zhong Jibin et al.), even in complex low-porosity and low-permeability reservoirs where fluid signatures often overlap. Each component, identified by fitting the T_2 spectrum with Gaussian functions, corresponds to different fluid environments such as clay-bound water, capillary-bound fluid, micropore fluid, and macropore fluid. Analysis of the free relaxation characteristics of crude oil and formation water, along with core-scale oil-water displacement experiments, allows for the precise identification of fluid types. For example, specific ranges of T_2 component spectral peaks can be defined for oil-bearing reservoirs, enabling accurate fluid identification. This advanced spectral decomposition significantly enhances the diagnostic capabilities of NMR in complex geological settings.

4 NMR in Enhanced Oil Recovery (EOR) Monitoring

Nuclear Magnetic Resonance (NMR) technology serves as a highly effective and versatile tool for characterizing and monitoring various Enhanced Oil Recovery (EOR) operations, both in laboratory settings and at the field scale [5, 109, 110, 7]. Its capability to provide detailed insights into fluid behavior within porous media allows for a granular understanding of displacement mechanisms, delineating oil and gas recovery from distinct pore systems within the rock matrix [5, 111, 12]. A primary objective of applying NMR in EOR is to screen various chemicals for their efficacy in applications such as carbon dioxide (CO_2), surfactant, and polymer flooding [5, 112, 113, 114]. Typically, the distribution of the T_2 relaxation time is employed to generate saturation profiles as a function of distance or along treated rock samples [109, 111, 115, 116].

Figure 9 illustrates the T_2 spectrum distributions of a core sample before

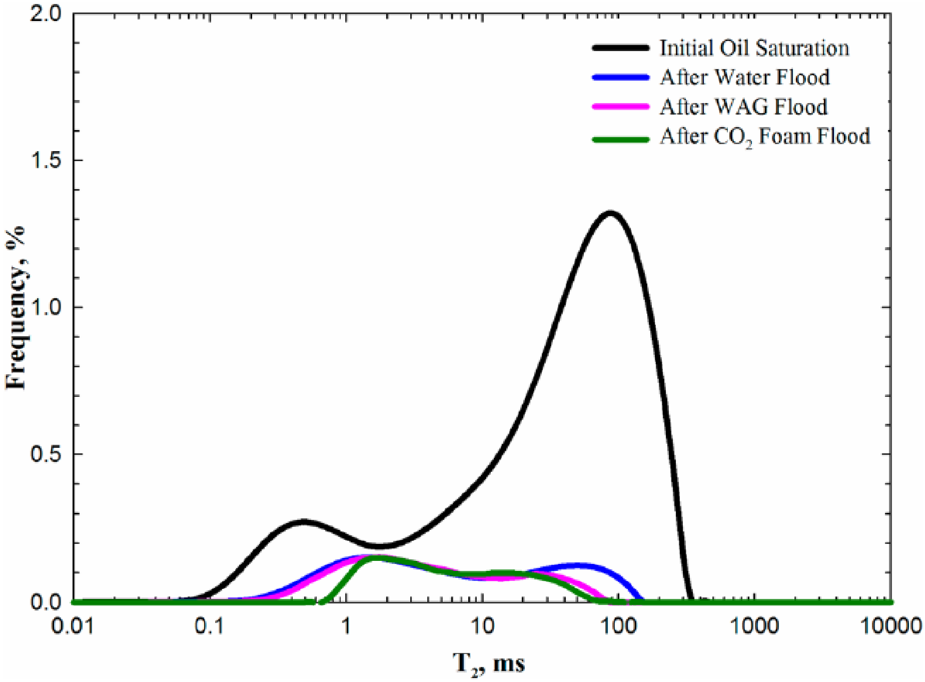

Figure 9: Measured T_2 spectrum distributions of core no. 1 before and after various floods. Adapted from Gao, Hui, et al., 2015 [16].

and after various flooding scenarios, including water flood, CO_2-foam flood, and water-alternating-gas (WAG) injection. It clearly depicts how different EOR methods selectively impact oil saturation across various pore throat sizes. Initially, a significant portion of oil is present in moderate pore throats. After water flooding, the residual oil is primarily concentrated in smaller pore throats [117, 47, 16]. Subsequent CO_2-foam flooding effectively targets and recovers oil from these smaller pores [118, 119], while WAG is more efficient in larger pore throats [113]. The combined application of these methods demonstrates the highest overall recovery by addressing oil in a broader range of pore sizes. By acquiring T_2 relaxation time profiles before and after an EOR treatment, researchers can accurately quantify the remaining oil saturation and gain valuable information regarding its distribution within the reservoir [111, 109, 112, 116].

4.1 Chemical EOR Monitoring

In chemical EOR (CEOR) processes, NMR plays a crucial role in monitoring the progress of injected chemicals, particularly surfactants and polymers. The technique allows for the evaluation of changes in pore surface wettability, which are often induced by surfactant adsorption onto the rock matrix. This real-time monitoring capability is vital for optimizing CEOR performance. For instance, studies have utilized spatial T_2 profiles to track oil saturation during alkaline surfactant (AS) flooding in carbonate rocks. By co-injecting brine and AS under simulated reservoir conditions of high pressure and high temperature, NMR tools can effectively track the oil-AS interface, thereby enhancing the understanding of oil recovery mechanisms for different surfactant formulations. Furthermore, NMR analysis can help identify injectivity issues during AS flooding by pinpointing plugged pores where oil displacement by injected chemicals is hindered.

Low-field NMR tools have been instrumental in laboratory settings for monitoring oil saturation during water flooding and sulfonate-based nano-surfactant (NS) injection in high-permeability carbonate core samples [112, 111, 115, 16]. The primary goal of such NMR analyses is to elucidate the underlying oil recovery mechanisms [112, 111, 113, 120]. Experiments employing various injection schemes and soaking periods have demonstrated that sequential injection of nano-surfactant followed by water flooding can yield the highest oil recovery [112]. NMR analysis in these cases has revealed that NS injection significantly enhances oil production by mobilizing both trapped and adsorbed oil from the rock surface [112].

For polymer flooding, NMR offers a comprehensive approach for characterization at various stages [114, 121, 5]. This includes assessing changes in the polymer's chemical structure, tracking its movement within the porous media [116], and identifying instances of polymer plugging [122]. The ability to evaluate gelation parameters, such as gel strength and gelation time, in-situ using NMR measurements further underscores its utility [114, 116]. A significant advantage of NMR in polymer flooding operations is its non-destructive nature, which facilitates quick and reliable measurements, providing critical data for optimizing these complex processes [5, 67]. While extensive research has been conducted, the interaction between oil and injected fluids during polymer flooding remains an area requiring further in-depth NMR investigation to fully disclose the intricate rock and fluid behaviors.

4.2 Gas Injection EOR Monitoring

Gas injection EOR methods, particularly those involving CO_2 flooding, benefit significantly from NMR monitoring. The technique is employed to characterize hydrocarbon flow and pore structures in tight sands during EOR operations. By determining oil recovery and optimizing soaking periods during huff-n-puff (HnP) cycles, NMR provides valuable profiles of free fluid (FF), capillary bound fluid (CAF), and clay-bound fluid (CBF) based on T_2 relaxation times. This allows for detailed analysis of fluid displacements across different pore sizes and system pressures. For example, studies have shown that during the initial cycles of CO_2 injection, free fluid and a substantial portion of capillary bound fluid are recovered from small and medium pores, while clay-bound fluid remains largely unaffected. Such detailed saturation profiles are crucial for optimizing CO_2 injection strategies.

The interaction between injected CO_2 and reservoir minerals is another critical aspect that NMR helps to unravel [6, 68, 113, 85]. Different mineral compositions within tight cores can significantly influence tight oil recovery [123, 27, 97]. For instance, in illite-dominated cores, CO_2 may not efficiently extract oil from smaller pores when the injection pressure is below the minimum miscible pressure (MMP) [123, 124]. However, if the injection pressure surpasses the MMP, crude oil can be effectively recovered from both small and large pores [123, 124, 125]. Conversely, in montmorillonite-dominated cores, oil saturation in medium pores might increase upon CO_2 injection, while in quartz-dominated cores, oil residing in both small and large pores can be substantially recovered [123, 126]. These pore-scale insights into mineral-fluid interactions are fundamental to understanding the mechanisms of CO_2 flooding [113, 125, 123].

Beyond CO_2 injection, NMR is also applied to more complex gas injection schemes such as CO_2-foam flooding and water-alternating-gas (WAG) injection [125, 127, 5]. These advanced EOR methods aim to improve sweep efficiency and mobilize residual oil. NMR tests can quantitatively determine the initial and residual oil distributions, revealing how different flood schemes impact microscopic oil recovery [109, 111, 16, 117]. For example, after a water flood, residual oil is predominantly found in smaller pore throats [117, 113, 94, 92]. CO_2-foam flooding has demonstrated effectiveness in recovering residual oil from these smaller pore throats, whereas WAG is more efficient at recovering oil from larger pore throats. The synergistic combination of CO_2-foam flooding and WAG has been shown to provide the highest overall

recovery efficiency by effectively reducing oil saturation across a wide range of pore sizes.

4.3 Thermal EOR Monitoring

Thermal EOR techniques, such as steam injection, are vital for heavy oil reservoirs where high viscosity hinders conventional production. While the provided references offer a general mention of NMR's application in thermal EOR, specific details on steam injection monitoring are limited. However, the broader utility of NMR in high-temperature environments is evident. Low-field NMR relaxometry, in conjunction with nonlinear least squares analysis, has been successfully employed for in-situ heavy oil viscosity prediction at elevated temperatures. This capability is paramount for understanding how thermal energy impacts oil properties and mobility within the reservoir, which is a core aspect of thermal EOR. NMR's sensitivity to fluid properties and phase behavior makes it an invaluable tool for assessing changes in oil viscosity and monitoring the overall effectiveness of thermal recovery processes. The ability to conduct these measurements under high-pressure and high-temperature conditions, representative of actual reservoir environments, is crucial for obtaining reliable and actionable data.

4.4 Techniques for Saturation and Fluid Distribution

NMR's adaptability stems from its diverse measurement techniques tailored for EOR applications. The T_2 distribution is a common approach for capturing changes in the rock porosity system and monitoring fluid saturations throughout EOR experiments.

Figure 10 illustrates a comparison between pore size distributions (PSD) obtained from NMR and those derived from Mercury Injection Capillary Pressure (MICP). This comparison is crucial in EOR monitoring as it provides a comprehensive understanding of the reservoir's pore architecture. While NMR offers insights into the distribution of fluids within the pore network, MICP provides details on the pore throat sizes. Correlating these two methods helps to accurately characterize the porous medium, which is vital for predicting how injected EOR fluids will flow, distribute, and displace oil. Understanding the pore structure aids in optimizing EOR strategies by matching fluid properties to the dominant pore sizes, thereby enhancing recovery efficiency.

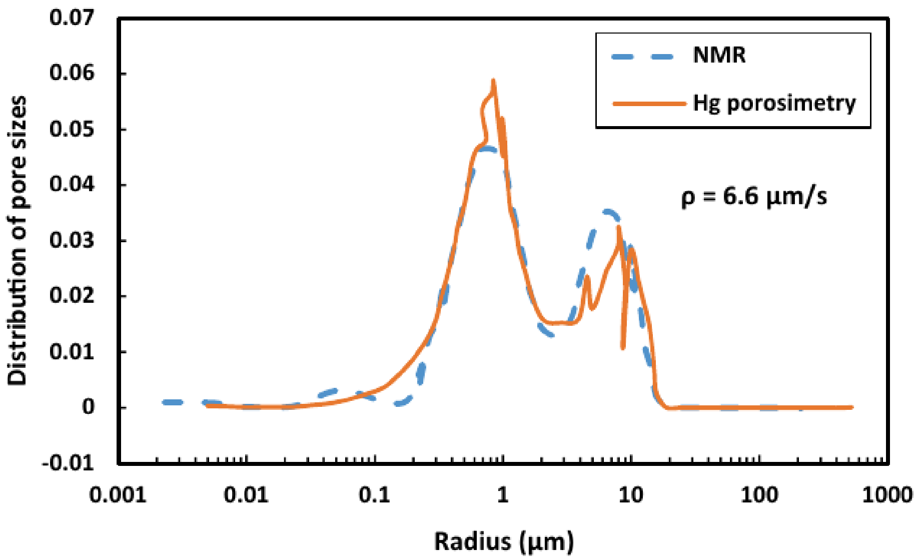

Figure 10: Comparison of NMR and MICP pore size distributions. Adapted from Elsayed, Mahmoud, et al., 2022 [5].

Beyond T_2, various other techniques are employed to determine oil saturation [67, 111, 109, 115], including chemical selective imaging [119, 116], complete signal suppression [112, 35], and paramagnetic doping [35, 50]. The choice of the most appropriate technique is contingent upon factors such as rock type, composition, and the specific chemical agents involved in the EOR process.

The Pulsed Field Gradient (PFG) NMR technique is particularly valuable for determining the diffusion coefficient of various fluids [128, 129, 130, 50]. This information can be utilized at early stages of EOR treatment to select suitable methods based on the pore network characteristics [5, 111, 112]. Diffusion measurements also aid in assessing pore coupling, which is critical for screening chemicals before their application in EOR [88, 90, 89]. For instance, in reservoirs with poor connectivity, chemicals that enhance pore connectivity might be preferred, while highly viscous fluids that necessitate high injection pressures could be excluded [100, 131, 85]. Mapping oil and water distributions in porous media through apparent diffusion coefficients is highly beneficial for designing and screening fluid systems for EOR [50, 35, 12].

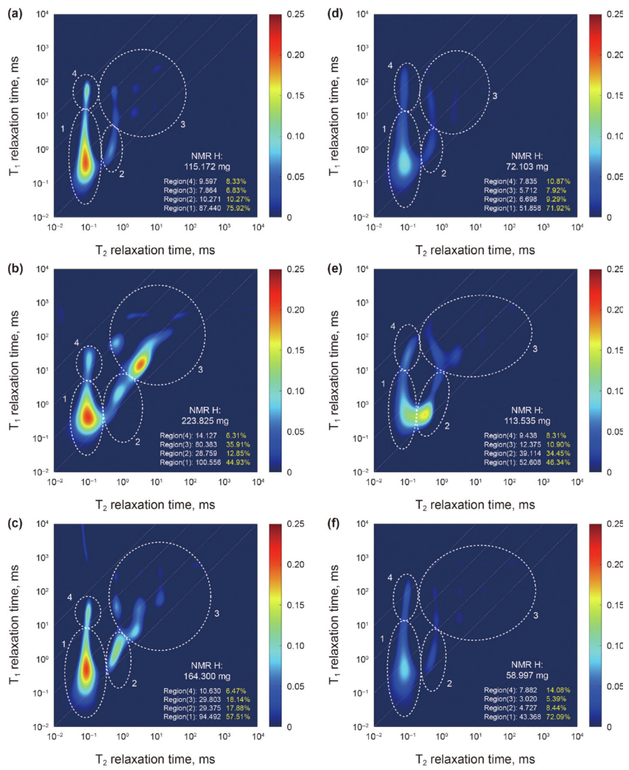

Shale sample depth	Portion	Status	NMR H, mg								
			Region 1		Region 2		Region 3		Region 4		All map
2138.60 m	Oil	Native	87.440	75.92%	10.271	10.27%	7.864	6.83%	9.597	8.33%	115.172
		Imbibed	100.556	44.93%	28.759	12.85%	80.383	36.91%	14.127	6.31%	223.825
		Dried	94.492	57.51%	29.375	17.88%	29.803	18.14%	10.63	6.47%	164.300
	Brine	Native	51.858	71.92%	6.698	9.29%	5.712	7.92%	7.835	10.87%	72.103
		Imbibed	52.608	46.34%	39.114	34.45%	12.375	10.90%	9.438	8.31%	113.535
		Dried	43.368	72.09%	4.727	8.44%	3.020	5.39%	7.882	14.08%	58.997

Figure 11: NMR T_1-T_2 maps of shale samples at different stages (native, imbibed, dried) for oil and brine portions. Adapted from Liu, Bo, et al., 2022 [132].

Figure 11 showcases NMR T_1-T_2 maps, which are powerful tools for characterizing fluid phases (oil and brine) and their intricate behavior within shale samples under various EOR-relevant conditions, such as native, imbibed, and dried states. These 2D correlation maps provide a distinct separation of different fluid components based on their unique relaxation properties. For instance, by comparing the maps of oil and brine portions under native conditions (a and d), imbibed conditions (b and e), and dried conditions (c and f), researchers can precisely track fluid saturation changes, identify trapped fluids, and assess the effectiveness of imbibition processes. This detailed fluid

characterization is essential for understanding the complex fluid-rock interactions in unconventional reservoirs and for optimizing EOR strategies to mobilize otherwise inaccessible hydrocarbons.

Magnetic Resonance Imaging (MRI) further augments NMR's capabilities by spatially resolving the distribution of fluid phases (water, oil, and gas) within the core samples [133, 134, 135, 50]. Laboratory-scale MRI is routinely used to assess the performance of different oil recovery strategies, including those involving chemicals (acids, polymers), supercritical CO_2, or miscible gases, prior to pilot operations in a reservoir [5, 109, 111, 113]. The ability to probe nuclei other than ^{1}H, such as ^{23}Na, allows for studies of miscible fluid injection, like analyzing oil recovery with varying salinity brine [68, 112]. Current research focuses on spatially resolved relaxation and diffusion measurements to generate wettability maps, an essential tool for core analysis, leading to a deeper understanding of oil recovery processes [77, 35, 76, 66]. Recent advancements in MRI techniques also enable the simultaneous measurement of rock structure (pore and grain size distributions) and fluid flow (flow propagators) on the same core plug [68, 12, 133, 136]. These laboratory-scale data are indispensable for refining models that predict the reservoir-scale effectiveness of EOR technologies, promising future insights into the intricate structure-transport interactions governing fluid flow in reservoirs [5, 109, 111].

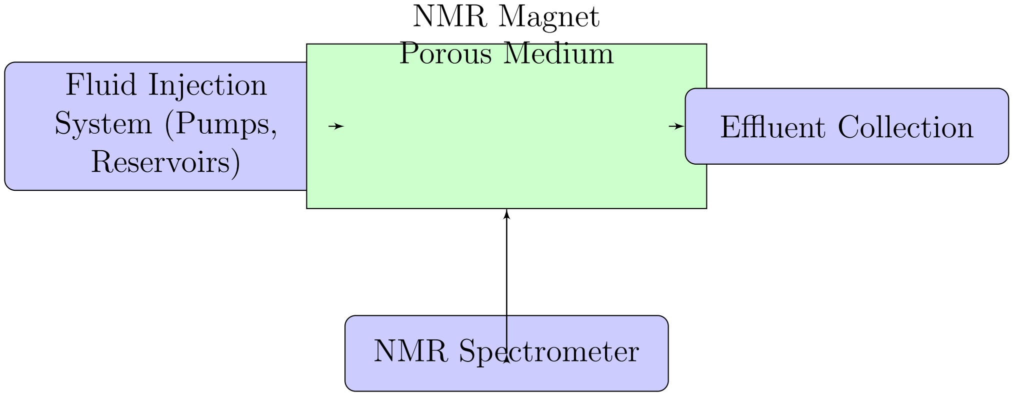

Figure 12: Schematic representation of a typical Nuclear Magnetic Resonance (NMR) integrated core flooding setup for Enhanced Oil Recovery (EOR) studies.

Figure 12 illustrates a typical integrated core flooding setup with an NMR

system, commonly used in laboratory-scale EOR studies. This schematic highlights the key components: a fluid injection system (comprising pumps and reservoirs) that supplies EOR fluids to a core holder containing the rock sample. The core holder is positioned within an NMR magnet, which is connected to an NMR spectrometer for data acquisition and analysis. An effluent collection system gathers fluids after they pass through the core. This integrated setup is critical for EOR research because it allows for real-time, non-invasive monitoring of fluid displacement, saturation changes, and fluid-rock interactions within the core sample under controlled conditions, directly correlating injection strategies with their microscopic effects on oil recovery.

4.5 Advantages and Limitations of NMR in EOR

A significant advantage of employing NMR for evaluating EOR operations is its capacity for detailed and continuous monitoring of remaining oil saturation, essential for optimizing performance. This capability extends to field applications, where NMR logging tools provide continuous in-situ oil saturation profiles due to their high acquisition speed. The integration of laboratory NMR coreflooding experiments with field logging data offers a comprehensive assessment of remaining oil saturation, thereby enhancing EOR strategies.

Despite these benefits, benchtop NMR instruments commonly used in laboratories present a key limitation: their typical design accommodates small core samples (2-4 inches). This restricts the analysis of longer core samples (6-20 inches), which are often preferred in coreflooding experiments to minimize capillary end effects and ensure more reliable results. A practical approach to overcome this involves cutting longer cores into smaller pieces and assembling composite cores for chemical flooding studies. Furthermore, while NMR has demonstrated broad utility in the energy sector, its application in EOR, particularly for a detailed understanding of rock-fluid interactions during polymer flooding, is still an evolving area with ongoing research needs. Future advancements are anticipated to bridge the gap between laboratory findings and field conditions, including the development of more efficient logging-while-drilling (LWD) hardware capable of operating effectively at high-pressure and high-temperature reservoir environments.

5 NMR in Unconventional Reservoirs

Unconventional reservoirs, encompassing shales, tight gas sands, and heavy oil/oil sands, present unique challenges for characterization due to their complex pore structures, varied fluid compositions, and low permeability [5, 69, 6, 34]. Nuclear Magnetic Resonance (NMR) has emerged as an indispensable tool for understanding these intricate systems, offering non-invasive and non-destructive analysis of critical petrophysical properties and fluid behavior [6, 34, 5]. Interpreting NMR signals in these formations is inherently more complex than in conventional reservoirs, primarily because the solid matrix and fluids exhibit distinct characteristics [34, 80, 137]. For instance, the hydrogen content within organic matter contributes significantly to the NMR signal in shales, making the resulting signal less dependent on lithology alone [34, 80, 138, 85]. This section delves into the specific applications of NMR across these diverse unconventional reservoir types, highlighting its utility in characterizing their unique properties.

5.1 Shale Characterization

Shale rocks, known for their fine-grained nature, high total organic content (typically exceeding 2%), and inherently low porosity and permeability [139, 140, 141, 34], present significant challenges for characterization. Their complex pore networks, comprising both organic and inorganic structures [142, 143, 94, 144], necessitate advanced analytical techniques like NMR [6, 34]. The inorganic component primarily consists of minerals such as silica, various clays, carbonates, and pyrite [140, 145]. These diverse pore types are visually exemplified in 13, which illustrates the presence of organic pores within organic matter, intergranular and intragranular dissolution pores, intercrystalline pores in pyrite, and various micro-fractures, all contributing to the complex pore architecture of shales [142, 143, 94, 144]. Conversely, the organic fraction encompasses a spectrum of materials, including kerogen (porous, insoluble organics), bitumen (soluble, highly viscous organics), low viscosity oils, and natural gas [34, 85, 146, 147]. The precise quantification of these organic components is paramount for accurately assessing shale reservoir quality and maturity, playing a vital role in establishing the economic viability of these unconventional resources [79, 148, 149, 94].

The relaxation dynamics observed in NMR experiments within shale rocks are notably intricate [6, 34]. In the inorganic pore structures, traditional

surface relaxivity is generally expected to be the dominant mechanism [31, 17, 21]. This phenomenon arises from the interaction of fluid molecules adsorbed onto pore surfaces with small quantities of paramagnetic metal species present at these surfaces [39, 27, 44, 26]. However, the relaxation mechanisms within the organic pore structures of shales are considerably more complex and remain a subject of ongoing discussion in the literature [34]. Several factors are understood to influence relaxation in these organic pores, including the thermal maturity of kerogen, where the solid proton density can significantly affect adsorbed surface relaxation [150, 36]. Furthermore, the nano-sized nature of kerogen pores introduces additional relaxation contributions due to molecular confinement effects [30, 74]. Diffusive coupling between organic and inorganic pore structures can also lead to an averaging of relaxation signals, potentially masking the presence of two distinct pore sizes, particularly when gas diffuses between these compartments [89, 88, 42, 34]. The origin of the organic matter itself also plays a crucial role, as it often contains paramagnetic metalloporphyrins such as Fe^{3+} and Mn^{2+} [151]. The presence and distribution of these paramagnetic minerals can substantially alter both longitudinal (T_1) and transverse (T_2) relaxation times, further complicating interpretation [44, 151, 26]. Lastly, the wetting characteristics of the pore structures are significant; inorganic pore structures are typically hydrophilic (water-wet), while organic pore structures are generally hydrophobic (oil-wet) [76, 152, 94].

For reliable characterization of shale rocks using NMR, understanding these relaxation considerations is vital. Two-dimensional (2D) NMR relaxometry, particularly T_1-T_2 maps, offers a powerful approach for identifying and quantifying different fluid phases, including water, oil, and solid organics, within shale samples [65, 153, 154, 132]. This technique is superior to one-dimensional T_2 distributions alone, as the latter can suffer from overlapping signals from oil, water, organic matter, and bound water, making differentiation challenging in unconventional cores. Fig. 11 illustrates a global T_1/T_2 map for fluid typing in shale rocks, which guides the interpretation of fluid behavior. Free bulk fluids typically exhibit long relaxation times, with T_1 and T_2 values being nearly identical, thus plotting close to the $T_1/T_2 = 1$ diagonal line [51, 18, 35]. Any signal detected in the region where T_1 is less than T_2 is considered physically unreasonable and often attributed to experimental artifacts. In T_1-T_2 maps, the water region tends to be concentrated along the diagonal line, indicating a T_1/T_2 ratio close to unity. In contrast, the oil region often displays a more complex and broad distribution of T_1/T_2 ratios,

typically ranging from 5 to 100, influenced by factors such as pore-size distribution, connectivity, organic matter content, and wettability of the shales [85, 35, 76, 94]. Molecules of heavy or solid organics, being less mobile, are characterized by very short T_2 values and relatively long T_1 values, leading to high T_1/T_2 ratios, which occupy distinct regions on the map [147, 154, 150, 85].

The application of higher-field NMR measurements, such as those at 19 MHz or 22 MHz, has significantly advanced shale characterization compared to traditional 2 MHz low-field NMR [68, 156, 27]. These higher frequencies offer improved signal-to-noise ratios (SNR), reduce detection times, and crucially, enhance the capability to measure bound water and solid organics, which typically exhibit very short T_2 relaxation times [68, 156, 27]. Higher-field NMR boasts a sensitivity 30 to 50 times greater than low-field NMR in distinguishing hydrogen signals from various proton populations, including hydroxyl groups, solid organic matter, oil/bitumen, and water [138, 137, 154, 65]. This enhanced resolution is critical for detailed analysis of shale oil occurrence characteristics and its mobility, which are often poorly understood despite their importance for resource evaluation and optimization of exploration targets [85, 80, 94, 79].

Beyond fluid typing, NMR is instrumental in characterizing the complex pore structure of shales [67, 69, 149]. Shales possess a significant proportion of pores in the nanometer size range, which are beyond the resolution limits of conventional microscopy techniques [74, 157, 158]. NMR, combined with methods like Mercury Intrusion Porosimetry (MIP) or nitrogen adsorption, provides a comprehensive understanding of pore size distribution [93, 74, 159, 92]. Fractal analysis, which accounts for the self-similar and irregular nature of pore structures, has also been successfully applied using NMR T_2 spectra to quantify micropores, mesopores, and macropores in tight formations [93, 159, 29]. Furthermore, NMR techniques can assess the wettability of shales [76, 152, 94], revealing that illitic shales tend to be strongly water-wet, while the presence of kaolinitic clays can impart an oil-wet tendency, with significant implications for oil and gas recovery strategies [160].

5.2 Tight Gas Sands

Tight gas sandstones are characterized by exceptionally narrow pore throats, intricate pore structures, and pronounced heterogeneity at both microscopic and macroscopic scales. The ultra-low permeability inherent to these forma-

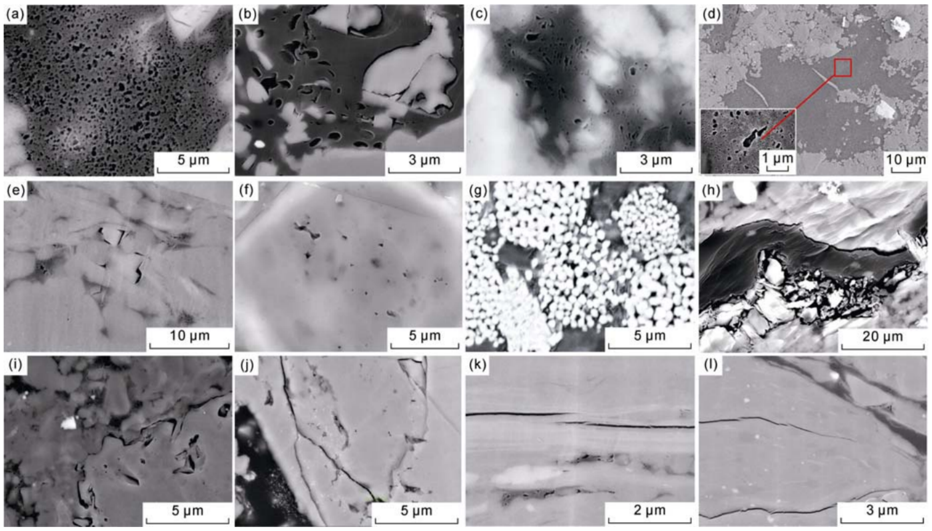

Figure 13: Organic pores, inorganic pores, and micro-fractures in shale reservoirs of the Wufeng Formation—Long1; sub-member in western Chongqing area. (a) Well Z208, 4366.05 m, a large number of round organic pores distributed in the organic matter; (b) Well Z201, 4356.77 m, organic pores not developed in organic matter partially next to the fracture; (c) Well 2205, 4270.20 m, organic pores deform due to structural movement or compaction; (d) Well Z206, 4268.64 m, connected large pores developed in the organic matter; (e) Well Z201, 4359.60 m, intergranular dissolution pores; (f) Well Z205, 3327.59 m, intragranular dissolution pores; (g) Well Z202, 3890.50 m, a large number of intercrystalline pores developed in the pyrite; (h) Well Z205, 3346.12 m, micro-fractures formed on the contact edge of organic matter and minerals; (i) Well Z203, 4105.07 m, curved dissolution fractures; (j) Well Z208, 4366.05 m, micro-fractures formed by compaction; (k) Well Z201, 4359.60 m, organic pores developed in migrating organic matter strips; (L) Well Z201, 4362.58 m, no organic pores developed in the migrating organic matter connected with micro-fractures. Adapted from Fu, Yonghong, et al., 2021 [155].

tions poses significant challenges for effective exploitation. In such reservoirs, the pore structure exerts a dominant influence on reservoir properties and fluid seepage characteristics, directly impacting the distribution, storage capacity, and ultimate recovery of natural gas. Consequently, quantitative studies of microscopic pore structures in tight sandstones have garnered sub-

stantial attention.

NMR stands out as a rapid, non-destructive, and repeatable method for characterizing the pore structures and fluid states within these challenging porous media. It facilitates high-precision measurement of micro-nano scale porosity in tight rocks and supports the high-throughput continuous testing required for efficient reservoir evaluation. The NMR transverse relaxation time (T_2) spectrum is commonly employed to infer the pore size distribution. For tight oil sandstones, the relationship between NMR T_2 and pore size is often found to be more accurately described by power function relations rather than linear ones, which is crucial for deriving precise pore size distributions from NMR data. Advances in methodology include the development of new approaches to determine the conversion factor from NMR T_2 distribution to pore size distribution, yielding results that are more suitable for tight sandstones when compared with traditional methods like MIP. The integration of NMR with MIP, which provides capillary pressure curves and a wide range of pore sizes, allows for an advanced fractal analysis of the complex pore networks. The fractal dimensions calculated from the NMR T_2 spectrum have proven effective for petrophysical properties analysis, providing a quantitative measure of the complexity and irregularity of the pore space.

Furthermore, NMR measurements are instrumental in estimating permeability in tight gas sands, with models often integrating grain size information to improve prediction accuracy, as grain size is a critical factor influencing NMR response and permeability. Beyond static properties, NMR is also applied to evaluate movable fluid distribution (MFD) in tight reservoirs. This involves combining NMR tests with centrifugation experiments to analyze fluid changes within the pores. Through such studies, an optimum centrifugal force can be determined to establish the threshold radius for fluid flow. Research indicates that movable fluids in tight sandstones are predominantly controlled by pore throats with radii smaller than 1 μm, particularly those ranging from 0.3 to 1 μm. These movable fluids are primarily stored in pores corresponding to the movable peak of a bimodal NMR T_2 distribution, typically with radii between 10 and 100 μm, which represent residual interparticle pores and dissolution pores. While direct studies on hydraulic fracturing impact on tight gas sands specifically using NMR are less detailed in the provided context, the general application of NMR to monitor changes in shale microstructure during EOR operations, such as Huff-n-Puff, suggests its potential for assessing alterations in pore throat size and surface area in-

duced by fracturing, which is a key process in unconventional gas recovery. This ability to detect subtle changes in pore geometry underscores NMR's value in understanding the effects of reservoir stimulation.

5.3 Heavy Oil and Oil Sands

Nuclear Magnetic Resonance also plays a critical role in the characterization and monitoring of heavy oil and oil sands reservoirs, which present distinct challenges due to the high viscosity and often low mobility of their hydrocarbon components. NMR T_1-T_2 spectra have been successfully applied in heavy oil reservoirs to gain insights into their complex fluid systems. A significant application of low-field NMR relaxometry in this domain is the in-situ prediction of heavy oil viscosity, even at elevated temperatures, which is crucial for optimizing production strategies given the strong temperature dependence of heavy oil viscosity. Moreover, NMR tools have been utilized for detailed heavy oil characterization during the precise placement of horizontal injectors at tar/oil interfaces, providing valuable information for field development and preventing issues like tar mat detection.

The ability of NMR to distinguish different fluid types, such as free water, capillary-bound water, and various oil fractions, is particularly advantageous in heavy oil systems. This fluid typing capability is fundamental for understanding the distribution and mobility of hydrocarbons within the reservoir pore space. Beyond static characterization, NMR is increasingly employed for monitoring Enhanced Oil Recovery (EOR) techniques in heavy oil and oil sands, including thermal-EOR processes such as Steam-Assisted Gravity Drainage (SAGD). NMR provides a unique capability to define the recovery of oil and gas from different pore systems within the rocks, offering a more granular understanding compared to macroscopic techniques that only assess bulk recovery. For instance, NMR allows for the monitoring of oil saturation using spatial T_1 profiles during chemical flooding operations, helping to track the oil-chemical interface and understand recovery mechanisms. This includes assessing injectivity problems by locating plugged pores where oil displacement is hindered. The technique has also been used to monitor oil saturation during water flooding and nano-surfactant injection, revealing how such treatments can mobilize trapped and adsorbed oil from rock surfaces. Furthermore, NMR facilitates the characterization of hydrocarbon flow and pore structures during EOR operations, such as CO_2 injection, by providing profiles of free fluid, capillary bound fluid, and clay-bound fluid based on

T_2 relaxation times. This allows for detailed analysis of fluid displacements across different pore sizes and pressures, aiding in the optimization of injection schemes. The utility of NMR extends to observing microstructural changes in the reservoir rock induced by EOR treatments, such as an increase in pore throat size and pore surface area following gas injection into shales, which provides critical insights for the design and application of EOR operations in tight and unconventional reservoirs. The characterization of oilfield emulsions, frequently associated with heavy oil production, through techniques like Pulsed Field Gradient (PFG) NMR, which measures restricted diffusion to determine emulsion droplet size, further demonstrates NMR's versatility in addressing production challenges in these complex systems.

6 Advanced NMR Techniques and Future Trends

Nuclear Magnetic Resonance (NMR) technology continues to evolve, with significant advancements in multi-dimensional techniques [62, 50, 67], high-field instrumentation [68], and sophisticated imaging capabilities [4, 133]. These developments aim to overcome limitations of conventional NMR methods, offering enhanced resolution, sensitivity, and comprehensive insights into complex porous media and fluid systems. The ongoing research and development in these areas are crucial for pushing the boundaries of NMR applications, particularly in the oil and gas industry [67, 8, 7, 82], where detailed understanding of reservoir properties and fluid behavior is paramount [161, 131, 79, 75]. Furthermore, the integration of cutting-edge computational methods [50, 162] and hardware innovations [7, 163, 164, 165] is shaping the future landscape of NMR, promising more efficient and accurate characterization tools.

6.1 Multi-Dimensional NMR

Multi-dimensional NMR techniques represent a significant leap forward from traditional one-dimensional measurements, providing richer and more detailed information about fluid properties and pore structures within complex systems. Among these, T_1-T_2 correlation spectroscopy and diffusion-weighted NMR are particularly prominent for their ability to resolve overlapping signals and characterize intricate transport phenomena.

T_1-T_2 correlation spectroscopy, often referred to as 2D NMR, leverages

the distinct relaxation behaviors of different fluids and their interactions with pore surfaces to provide a more definitive fluid typing and a more accurate characterization of pore size distribution[62, 57, 11, 85]. Unlike 1D T_2 spectra, which can suffer from overlapping signals from various fluid phases (e.g., oil and water in low-porosity reservoirs), 2D NMR maps allow for improved separation of these components[62, 57, 11, 85]. For instance, studies have demonstrated the effectiveness of T_1-T_2 maps in identifying and quantifying water, oil, and solid organics in shale samples, offering both qualitative and quantitative insights (Fleury and Romero-Sarmiento, 2016; Khatibi et al., 2019)[85]. The T_1/T_2 ratio, a key parameter derived from these measurements, has also proven to be a more robust indicator for wettability prediction, especially when diffusion relaxation effects are significant (Katika et al., 2017; Valori et al., 2017)[78, 51, 18, 166]. This is because 2D NMR provides a clearer separation of fluid populations, allowing for a more accurate assessment of how molecular motion and surface interactions influence the T_1/T_2 ratio, which deviates from unity for viscous or wetting fluids.

Figure 14 exemplifies the utility of T_1-T_2 maps in monitoring dynamic processes within porous media, showing the changes in fluid distribution within a shale sample during imbibition over several days. Such maps visually represent the evolution of water and oil signals, providing direct insights into fluid movement and saturation changes within different pore environments over time.

Beyond T_1-T_2 correlation, T_2-D NMR techniques, which map transverse relaxation time against the diffusion coefficient, offer another powerful approach for fluid characterization and separation [62, 35, 57, 66]. This method is particularly effective for distinguishing fluids based on their molecular mobility, which is influenced by both fluid viscosity and pore confinement [35, 136, 131, 130]. The improved fluid separation achieved with T_2-D NMR also contributes to more accurate calculations of effective surface relaxivities of fluids (Minh et al., 2015). The ability of 2D NMR techniques to better distinguish between different fluid phases and their confinement states is invaluable for understanding fluid distribution in heterogeneous porous media and for evaluating enhanced oil recovery (EOR) processes [35, 113, 85, 50].

Diffusion-weighted NMR, particularly techniques involving Pulsed Field Gradients (PFG), provides critical information about fluid mobility and pore connectivity [167, 128, 130, 67]. The PFG NMR method is non-invasive and allows for the determination of diffusion coefficients for various fluids within porous media (Elsayed et al., 2021b; Johnson, 1999; Willis et al., 2016) [136,

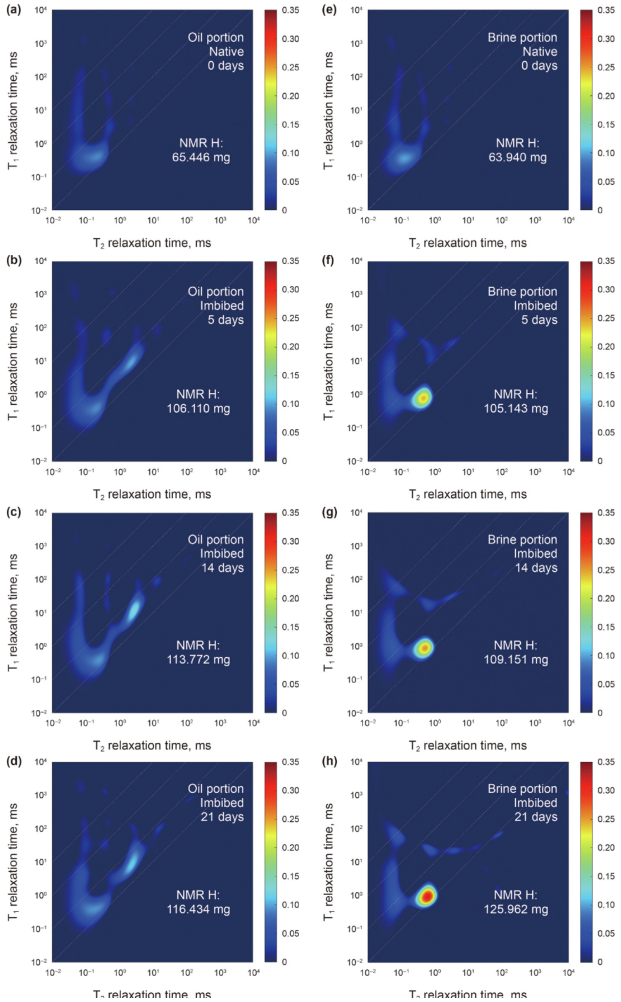

Figure 14: NMR T-T2 maps of the shale sample from 2107.59 m depth at different imbibed stages of 0 days, 5 days, 14 days and 21 days. Adapted from Liu, Bo, et al., 2022 [132].

168, 129, 81]. This is particularly useful in the early stages of EOR treatments, as it can help in selecting suitable methods based on the pore network characteristics. For example, in reservoirs with poor connectivity, chemicals designed to improve pore connectivity might be favored, while highly viscous fluids requiring high injection pressures could be excluded (Elsayed et al., 2021b) [112, 67, 100, 169]. Diffusion measurements can also assess pore

coupling phenomena, where fluid molecules can exchange between pores of different sizes, especially in multi-modal pore systems like those found in carbonates [88, 89, 90, 42]. Understanding these diffusion coupling effects is essential for accurately estimating pore sizes from NMR relaxation times (Johnson and Schwartz, 2014; Yu et al., 2019) [90, 40, 29, 26]. The presence of internal magnetic field gradients, arising from susceptibility differences at fluid-solid interfaces, can significantly influence diffusion measurements and relaxation times (Hürlimann, 1998; Mitchell et al., 2010; Tandon and Heidari, 2018) [29, 26, 27, 39]. Advanced PFG techniques and numerical simulations are being developed to account for and utilize these internal gradients for more precise characterization of porous media (Connolly et al., 2019; Zhang et al., 1998) [50, 90, 26, 129]. The mapping of oil and water distributions in porous media using apparent diffusion coefficients is another powerful application, aiding in the design and screening of various fluid systems for EOR applications (Elsayed et al., 2021b) [50, 81, 82, 170].

6.2 High-Field NMR

The choice of magnetic field strength in NMR experiments significantly impacts the signal-to-noise ratio (SNR), resolution, and the types of phenomena that can be observed. High-field NMR spectrometers, characterized by very strong magnetic fields (typically exceeding 3 Tesla), offer distinct advantages for certain applications, particularly in the detailed characterization of complex fluid systems and heterogeneous rock structures.

The primary advantage of high-field NMR lies in its enhanced sensitivity and resolution. A stronger magnetic field generally leads to a higher polarization of nuclear spins, resulting in a significantly stronger NMR signal and thus a higher SNR (Hoult and Richards, 1976; Mitchell et al., 2013). This improved SNR allows for shorter acquisition times, reducing the overall experimental duration, and enables the detection of weaker signals from less abundant nuclei or smaller sample volumes. For instance, in the context of unconventional reservoirs, higher field NMR systems (e.g., 22 MHz compared to the more common 2 MHz low-field systems) can bring substantial benefits in terms of SNR and reduced detection time, while also providing the capability to measure bound water and solid organics that have very short T_2 relaxation times [156, 69, 80, 76]. This is crucial for characterizing the extremely small pores and complex organic matter in tight shales [74, 150, 79, 26].

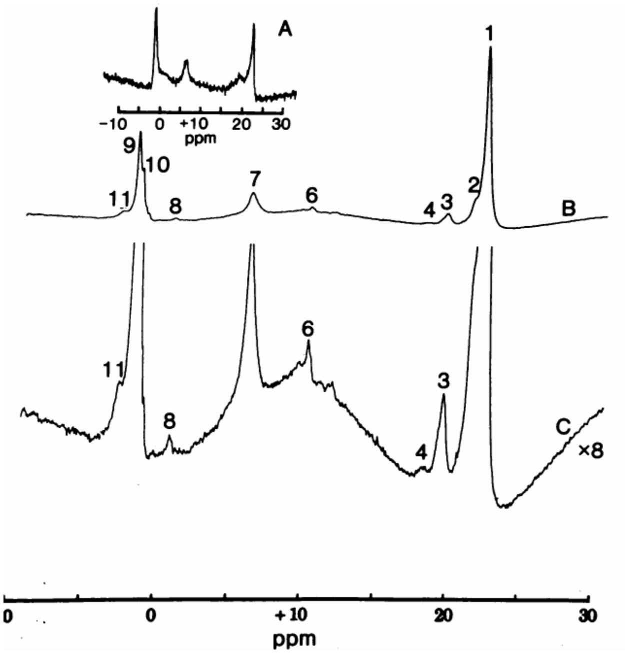

Figure 15: Field dependence of 50% suspension of yeast cells in their stationary phase of growth. (A) Spectrum taken at 40.5 MHz by pulsed FT NMR; 12 mm sample tube, pH 6.4. This spectrum was recorded after 2924 accumulations with a repetition time of 2 sec per pulse, an HO lock, and without proton decoupling. (B) Spectrum taken at 145.7 MHz by pulsed FT NMR; 10 mm sample tube, pH 3.5, 4800 accumulations with a repetition time of 0.6 sec. The sample contained about 10% D2O to lock the magnetic field. (C) Same spectrum as B, after turning up the gain a factor of 8. Adapted from Salhany, J M, et al., 1975 [171].

The benefits of high-field systems are visually demonstrated in Figure 15, which illustrates how increasing the magnetic field strength from 40.5 MHz to 145.7 MHz dramatically improves the spectral resolution and signal definition, allowing for clearer distinction of molecular components. Although this specific example is from a biological system, the principle of enhanced resolution and sensitivity directly translates to the characterization of complex fluid mixtures and pore environments in petroleum applications.

The enhanced resolution of high-field NMR allows for finer discrimination of chemical shifts and relaxation times, which is essential for studying complex fluid mixtures, such as crude oils with varying compositions, or flu-

ids within highly heterogeneous pore networks [27, 68, 100]. This capability can provide detailed insights into the molecular dynamics of fluids confined within pores, as well as their interactions with different mineral surfaces [88, 37, 18]. For example, high-field NMR can be used to probe different nuclei beyond ^{1}H, such as ^{23}Na, to monitor miscible fluid injection processes and analyze oil recovery with varying salinity brine (Yang and Kausik, 2016). The ability to acquire high-resolution spectra and relaxation data at high fields opens avenues for more sophisticated characterization of fluid-rock interactions, which is critical for optimizing various petroleum engineering applications, including EOR and formation damage assessment [67, 111, 112, 172].

6.3 NMR Imaging (MRI)

Nuclear Magnetic Resonance Imaging (MRI) extends the capabilities of traditional NMR by providing spatially resolved information, allowing for the visualization of fluid distribution and flow pathways within porous media. This non-invasive technique is invaluable for understanding complex phenomena that occur at the pore scale and for assessing macroscopic fluid transport in rock samples.

At the laboratory scale, MRI is extensively utilized to provide spatially resolved insights into the performance of various oil recovery strategies before their field implementation [67, 109, 110, 111]. Unlike bulk measurements, MRI directly visualizes the dynamic distribution of fluid phases—water, oil, and gas—within the rock matrix during processes such as chemical injections (e.g., acids, polymers) [112, 121], supercritical CO_2 flooding, or miscible gas injection [119, 173, 174, 113] (Lai et al., 2020; Li et al., 2017; Zhao et al., 2020a, b). This capability is particularly important for understanding the mechanisms of improved oil recovery (IOR) and EOR techniques, as it allows for direct observation of displacement fronts, identification of bypassed oil, and visualization of the impact of treatments on the pore structure at a macroscopic level [67, 109, 111, 135].

Beyond fluid distribution, recent advancements in MRI techniques enable the measurement of rock structure, including pore and grain size distributions, and fluid flow properties, such as flow propagators, on the same core-plug (Karlsons et al., 2021). This integrated approach provides a more comprehensive understanding of the structure-transport interactions that govern fluid flow in reservoirs. Spatially resolved relaxation and diffusion measure-

ments, facilitated by MRI, are also crucial for generating wettability maps, which are key tools for core analysis and lead to a better understanding of oil recovery processes (Karlsons et al., 2021). These laboratory-scale data are vital for enhancing predictive models that forecast the reservoir-scale effectiveness of oil recovery technologies.

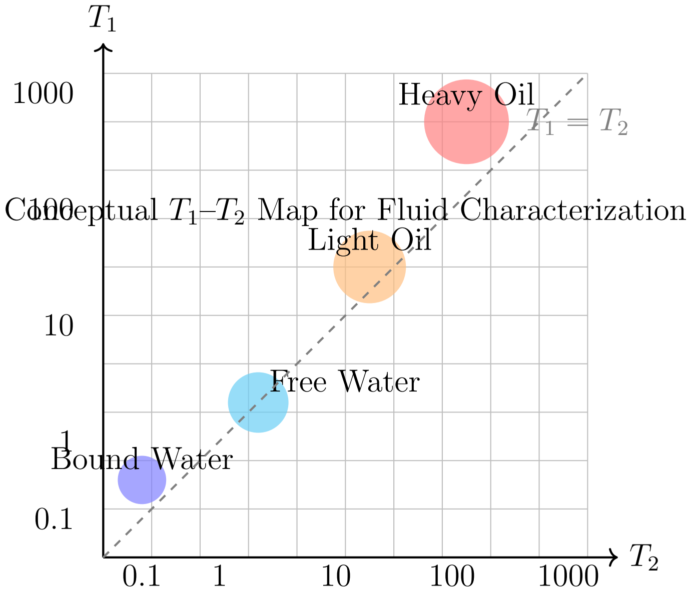

Figure 16: Conceptual representation of a T_1–T_2 correlation map, illustrating how different fluid types (bound water, free water, light oil, heavy oil) can be distinguished based on their distinct relaxation characteristics. The diagonal line represents the ideal $T_1 = T_2$ condition, with deviations indicating varying degrees of molecular restriction and surface interaction. This visualization greatly aids in fluid typing and understanding pore environments.

The application of MRI is not limited to laboratory core analysis. Surface Nuclear Magnetic Resonance (SNMR), also known as Magnetic Resonance Sounding (MRS), is a surface-based geophysical method that utilizes NMR principles to directly map groundwater aquifers without the need for boreholes. While earlier SNMR practices primarily relied on 1D inversion strategies, recent developments have introduced very fast 2D SNMR tomographic inversion schemes, which can more accurately reconstruct complex subsurface water distributions (Hertrich et al., 2007; Legchenko et al., 2011). The capabilities of SNMR are further exemplified by Figure 17, which

presents a 3D tomographic reconstruction of water distribution in a glacier. This figure demonstrates SNMR's power to map subsurface water bodies in a non-invasive manner, providing crucial spatial information about aquifer geometry and water content, which is directly translatable to groundwater exploration and management in various geological settings. This capability to obtain NMR measurements from the Earth's surface revolutionizes non-invasive evaluation of groundwater resources, providing a direct link to the presence and amount of water in the pore space through the detection of hydrogen nuclei magnetization (Knight et al., 2016).

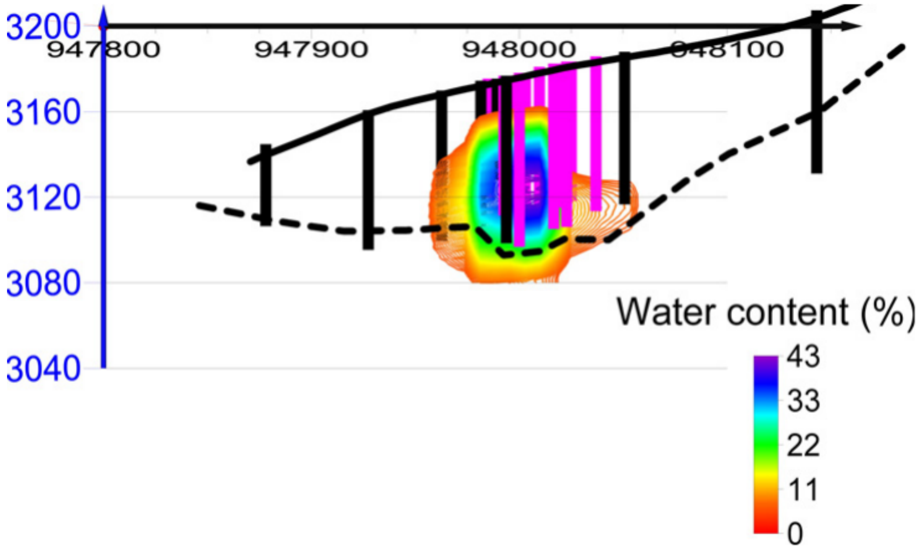

Figure 17: West-East cross-section of the water distribution derived from 3D-SNMR measurements (see also figure 14). Black columns show boreholes that did not detect water; pink columns show boreholes that intersected water-filled caverns. Adapted from Legchenko, A, et al., 2011 [175].

These advancements underscore the potential of MRI to provide crucial, spatially resolved data for both reservoir characterization and environmental applications.

6.4 Future Directions in NMR Research

The field of Nuclear Magnetic Resonance is dynamic, with ongoing research focused on enhancing its capabilities and expanding its applications across

various scientific and industrial domains. Several key areas are poised for significant advancements, promising to revolutionize how we understand and characterize porous media and fluid systems.

One critical area of future development is the optimization and modification of NMR pulse sequences and data processing techniques. The current standard experiments, such as 2D NMR diffusion measurements, can be lengthy, which poses challenges for operational efficiency, especially in field applications. Developing robust numerical simulation models that account for all relaxation phenomena, including the effects of bulk and internal gradients, is a promising avenue to examine various scenarios and reduce the need for time-consuming physical experiments. Furthermore, enhancing the signal-to-noise ratio (SNR) is a key parameter for obtaining accurate measurements, and continuous efforts are directed towards designing pulse sequences that maximize signal acquisition while minimizing noise (Chen et al., 2018b; Sun et al., 2020). This involves sophisticated inversion algorithms and data reconstruction methods that can extract maximum information from acquired signals, even under challenging conditions.

Another vital direction involves the integration of NMR data with other geophysical and petrophysical measurements. While NMR provides unique insights into rock-fluid interactions, its full potential is realized when combined with complementary data sources. For instance, cross-validation between laboratory rock core measurements and field logging data is essential for building reliable predictive models for reservoir properties. Efforts to accurately overlap Mercury Intrusion Capillary Pressure (MICP) and NMR relaxation distributions, often by utilizing advanced imaging techniques like X-ray micro-computed tomography (μCT), aim to better evaluate surface relaxivity and pore size distributions (Connolly et al., 2019; Lyu et al., 2020). This multi-modal data integration approach helps to overcome the inherent limitations of individual techniques and provides a more comprehensive understanding of complex reservoir characteristics.

The application of machine learning (ML) and artificial intelligence (AI) is rapidly emerging as a transformative force in NMR data interpretation. Given the large volumes of complex, multi-dimensional data generated by advanced NMR experiments, conventional analysis methods can be time-consuming and may not fully capture subtle correlations. While specific applications of ML/AI to NMR in the context of this review's provided literature are still emerging, the overarching need for "accurate predictions" and "robust numerical simulation models" in reservoir characterization strongly

points towards the increasing adoption of these advanced computational methods. ML and AI algorithms can be trained to recognize patterns, classify fluid types, predict petrophysical properties, and even automate the inversion of NMR relaxation data, leading to faster and more accurate interpretations. These technologies hold the potential to significantly streamline the NMR workflow, from data acquisition to final interpretation, making NMR a more accessible and powerful tool for reservoir characterization and monitoring.

Finally, the development of more efficient Logging While Drilling (LWD) hardware is crucial for bringing laboratory findings to field-scale applications. LWD-NMR tools have shown great potential in providing real-time information about formation lithology, fluid viscosity, and pore systems, aiding in geosteering and optimizing well placement (Jachmann et al., 2020; Prammer et al., 2000a; Sun et al., 2020). Future research will focus on improving the robustness, resolution, and data acquisition speed of these downhole tools to mimic the accuracy and detail achievable in laboratory settings, thereby providing more reliable real-time insights during drilling operations. These combined efforts in technique refinement, data integration, computational intelligence, and hardware innovation are paving the way for NMR to become an even more indispensable tool in the energy industry and beyond.

7 Conclusion

Nuclear Magnetic Resonance (NMR) technology has emerged as an indispensable tool across various facets of the oil and gas industry, revolutionizing the understanding and characterization of porous media and fluid behavior from laboratory to field scales [109, 176, 68]. This comprehensive survey has elucidated the fundamental principles governing NMR measurements, its diverse applications in petrophysics [82, 81, 70, 71], enhanced oil recovery (EOR) monitoring [109, 114], and unconventional reservoir characterization [69, 177, 80, 94], alongside its significant role in logging while drilling (LWD) operations and geosteering [7, 165, 161, 178]. The unique capabilities of NMR provide insights that are often unattainable or less precise with conventional methods, thereby enhancing decision-making in exploration, development, and production phases.

The profound impact of NMR stems from its ability to collectively provide a comprehensive and non-invasive characterization of reservoir rocks and their contained fluids [7, 8, 82, 68]. Unlike traditional techniques that may

alter samples or provide only bulk-averaged properties, NMR offers detailed information on porosity [20, 161], pore size distribution [87, 93, 74], permeability [75, 8], fluid saturation [81, 79], capillary pressure [169, 92], and wettability [76, 179]. For instance, the transverse relaxation time, T_2, distribution directly correlates with pore size, allowing for a precise mapping of the pore network architecture [21, 39, 40, 180]. This direct measurement of fluid-filled pore volumes, independent of mineralology, provides a distinct advantage over density or neutron logs, which can be influenced by lithological variations [161, 7, 86]. Furthermore, NMR's sensitivity to fluid properties, such as viscosity and diffusion [81, 176, 42], enables the differentiation of various fluid phases (water, oil, gas) [82, 8, 80] and their mobility [82, 101] within the complex pore systems of reservoir rocks. This is particularly crucial for identifying bypassed pay zones and evaluating reservoir quality in challenging environments [79, 7], offering a synergistic understanding of reservoir potential.

In the context of Enhanced Oil Recovery (EOR), NMR has proven to be a superior technique for monitoring fluid displacements and residual oil saturation. Traditional macroscopic methods often provide only bulk recovery data, whereas NMR allows for the quantification of oil and gas recovery from different pore systems, providing a more granular understanding of EOR efficiency. The ability to track fluid saturation profiles over time, both in laboratory coreflooding experiments and through NMR logging, enables continuous monitoring of EOR treatments. This real-time insight into the progress of injected fluids, such as surfactants, polymers, or carbon dioxide, helps in optimizing chemical formulations and injection strategies. The pulsed field gradient (PFG) NMR technique, for example, offers a non-invasive means to determine diffusion coefficients, aiding in the selection of suitable EOR methods based on pore network connectivity and ultimately improving recovery factors.

The impact of NMR extends significantly to unconventional reservoirs, where the intricate pore structures and heterogeneous compositions pose considerable challenges for characterization. NMR provides valuable information on pore size distribution, fluid typing, and organic matter content in shale formations, which is critical for assessing hydrocarbon storage and producibility. The development of advanced NMR techniques, such as two-dimensional (2D) NMR relaxometry (e.g., T_1-T_2 maps), has further enhanced the ability to distinguish between different fluid phases and solid organic matter, even in tight and complex unconventional systems. This multidimensional

approach improves the accuracy of wettability characterization and helps in understanding the complex interactions between fluids and the rock matrix, providing a holistic view essential for unconventional resource development.

Despite its numerous advantages, the sophisticated nature of NMR applications, while yielding profound insights, also introduces inherent complexities and challenges that demand continuous advancement. A concise summary of these key benefits and challenges is provided in Table 1.

One significant challenge in NMR measurements, particularly in porous media, is the influence of internal magnetic field gradients and pore coupling effects. These phenomena arise from the heterogeneity of porous media, causing fluid molecules to experience varying local magnetic fields or to exchange between pores, which can lead to averaged or distorted relaxation signals that complicate accurate interpretation. While techniques exist to minimize these effects, such as using short echo times or low magnetic fields, their complete elimination remains an area of active investigation. Furthermore, benchtop NMR instruments used for core analysis often have sample size limitations, typically accommodating cores of only 2 to 4 inches. This can restrict the analysis of longer core samples, which are preferred for coreflooding experiments to avoid capillary end effects, often necessitating the use of composite cores.

Table 1: Key Benefits and Challenges of NMR in Oil and Gas Applications

**Benefits of NMR**	**Challenges and Limitations**
Non-invasive and non-destructive analysis	Sensitivity to internal magnetic field gradients
Accurate porosity independent of lithology	Pore coupling effects in heterogeneous media
Detailed pore size distribution	Benchtop limitations for long core samples
Quantification of fluid saturation and mobility	Complex T_1/T_2 interpretation for heavy oils
Real-time monitoring of EOR processes	Overlapping T_2 spectra in low-porosity reservoirs
Characterization of unconventional reservoirs	Influence of drilling fluids on LWD data
Geosteering and well placement optimization	Need for enhanced signal-to-noise ratio in LWD

Another challenge pertains to the interpretation of wettability using T_1/T_2 ratios, especially in the presence of heavy oils or asphaltenes, where the ratio may deviate from expected values due to fluid properties rather than solely surface interactions. For low-porosity and low-permeability reservoirs, traditional NMR fluid identification methods can suffer from low accuracy due to significant overlap in the T_2 spectra of oil and water. This overlap makes it difficult to unambiguously differentiate between oil and water signals, especially in tight formations where fluid mobility and pore sizes are restricted. In field applications, LWD-NMR tools, while providing real-time data, can be influenced by drilling fluids in the borehole and radial vibrations during drilling, which may affect the reliability and accuracy of T_2 measurements. The development of robust numerical simulation models that account for all relaxation phenomena, including bulk and internal gradient effects, is crucial for saving time in lengthy experiments and improving predictive capabilities.

Looking ahead, the future of NMR technology in the oil and gas industry is poised for continuous innovation and broader application. A key direction involves the further development and widespread adoption of multi-dimensional NMR techniques, such as T_1-T_2 and diffusion-T_2 maps, which offer superior fluid separation and provide more comprehensive information on pore connectivity and wettability. Enhancing the signal-to-noise ratio (SNR) of collected data, particularly during LWD operations, will be vital for improving measurement accuracy and reducing acquisition time, thereby lowering operational costs. Research efforts are also focused on developing more efficient LWD hardware that can mimic laboratory results, enabling more precise real-time formation evaluation downhole. To fully unlock the potential of these advanced NMR techniques and address complex reservoir problems comprehensively, fostering robust interdisciplinary collaboration among physicists, engineers, and geoscientists will be increasingly crucial.

Moreover, there is a growing need for NMR measurements to be conducted under reservoir-representative conditions, including high pressure and high temperature, using longer core samples to provide more reliable and scalable results for EOR evaluations. Investigations into the rock-fluid interactions during advanced EOR methods, such as polymer flooding, using NMR are also critical. The integration of NMR data with other advanced characterization techniques, such as X-ray computed tomography (CT) scanning, mercury intrusion porosimetry (MIP), and electrical resistivity measurements, will lead to a more holistic understanding of reservoir properties. The exploration of NMR applications involving nuclei other than hydrogen,

such as ^{13}C or ^{23}Na, could unlock new insights into specific chemical processes and fluid behaviors. Overall, NMR stands at the forefront of reservoir characterization, offering unparalleled detail and dynamic monitoring capabilities that will continue to drive advancements in hydrocarbon exploration and production optimization.

References

[1] Komal Zia et al. "Nuclear Magnetic Resonance Spectroscopy for Medical and Dental Applications: A Comprehensive Review". In: *European Journal of Dentistry* 13.01 (2019), 124–128. ISSN: 1305-7464. DOI: 10.1055/s-0039-1688654. URL: http://dx.doi.org/10.1055/s-0039-1688654.

[2] Joseph P. Hornak. "Teaching NMR Using Online Textbooks". In: *Molecules* 4.12 (1999), 353–365. ISSN: 1420-3049. DOI: 10.3390/41200353. URL: http://dx.doi.org/10.3390/41200353.

[3] William S. Price. *NMR Studies of Translational Motion: Principles and Applications*. Cambridge University Press, 2009. ISBN: 9780511770487. DOI: 10.1017/cbo9780511770487. URL: http://dx.doi.org/10.1017/cbo9780511770487.

[4] Paul T Callaghan. *Principles of Nuclear Magnetic Resonance Microscopy*. Oxford University PressOxford, 1991. ISBN: 9781383026610. DOI: 10.1093/oso/9780198539445.001.0001. URL: http://dx.doi.org/10.1093/oso/9780198539445.001.0001.

[5] Mahmoud Elsayed et al. "A review on the applications of nuclear magnetic resonance (NMR) in the oil and gas industry: laboratory and field-scale measurements". In: *Journal of Petroleum Exploration and Production Technology* 12.10 (2022), 2747–2784. ISSN: 2190-0566. DOI: 10.1007/s13202-022-01476-3. URL: http://dx.doi.org/10.1007/s13202-022-01476-3.

[6] Jian-Chun Guo et al. "Advances in low-field nuclear magnetic resonance (NMR) technologies applied for characterization of pore space inside rocks: a critical review". In: *Petroleum Science* 17.5 (2020), 1281–1297. ISSN: 1995-8226. DOI: 10.1007/s12182-020-00488-0. URL: http://dx.doi.org/10.1007/s12182-020-00488-0.

[7] Kirill Kuptsov, Roger Griffiths, and David Maggs. “Technology Update: Magnetic Resonance-While-Drilling System Improves Understanding of Complex Reservoirs”. In: *Journal of Petroleum Technology* 67.12 (2015), 26–29. ISSN: 1944-978X. DOI: 10.2118/1215-0026-jpt. URL: http://dx.doi.org/10.2118/1215-0026-jpt.

[8] Gabor Hursan, Andre Silva, and Mohamed L. Zeghlache. “Evaluation and Development of Complex Clastic Reservoirs using NMR”. In: *SPE Annual Technical Conference and Exhibition*. 16ATCE. SPE, 2016. DOI: 10.2118/181525-ms. URL: http://dx.doi.org/10.2118/181525-ms.

[9] Stefan Menger and Manfred Prammer. “A New Algorithm for Analysis of NMR Logging Data”. In: *SPE Annual Technical Conference and Exhibition*. 98SPE. SPE, 1998. DOI: 10.2118/49013-ms. URL: http://dx.doi.org/10.2118/49013-ms.

[10] Wei Shao and Ron Balliet. “NMR Logging Data Processing”. In: *Petrophysics – The SPWLA Journal of Formation Evaluation and Reservoir Description* 63.3 (2022), 300–338. ISSN: 2641-4112. DOI: 10.30632/pjv63n3-2022a3. URL: http://dx.doi.org/10.30632/pjv63n3-2022a3.

[11] Jiangfeng Guo et al. “Nuclear Magnetic Resonance T1–T2 Spectra in Heavy Oil Reservoirs”. In: *Energies* 12.12 (2019), p. 2415. ISSN: 1996-1073. DOI: 10.3390/en12122415. URL: http://dx.doi.org/10.3390/en12122415.

[12] Hsie-Keng Liaw et al. “Characterization of fluid distributions in porous media by NMR techniques”. In: *AIChE Journal* 42.2 (1996), 538–546. ISSN: 1547-5905. DOI: 10.1002/aic.690420223. URL: http://dx.doi.org/10.1002/aic.690420223.

[13] Matej Hriberšek. “Predgovor”. In: *Clotho* 6.1 (2024), 7–8. ISSN: 2670-6210. DOI: 10.4312/clotho.6.1.7-8. URL: http://dx.doi.org/10.4312/clotho.6.1.7-8.

[14] R.J.S. Brown and B.W. Gamson. “Nuclear Magnetism Logging”. In: *Transactions of the AIME* 219.01 (1960), 201–209. ISSN: 0081-1696. DOI: 10.2118/1305-g. URL: http://dx.doi.org/10.2118/1305-g.

[15] M. Josh et al. “Laboratory characterisation of shale properties”. In: *Journal of Petroleum Science and Engineering* 88–89 (2012), 107–124. ISSN: 0920-4105. DOI: 10.1016/j.petrol.2012.01.023. URL: http://dx.doi.org/10.1016/j.petrol.2012.01.023.

[16] Hui Gao et al. “Impact of Secondary and Tertiary Floods on Microscopic Residual Oil Distribution in Medium-to-High Permeability Cores with NMR Technique”. In: *Energy amp; Fuels* 29.8 (2015), 4721–4729. ISSN: 1520-5029. DOI: 10.1021/acs.energyfuels.5b00394. URL: http://dx.doi.org/10.1021/acs.energyfuels.5b00394.

[17] Morrel H. Cohen and Kenneth S. Mendelson. “Nuclear magnetic relaxation and the internal geometry of sedimentary rocks”. In: *Journal of Applied Physics* 53.2 (1982), 1127–1135. ISSN: 1089-7550. DOI: 10.1063/1.330526. URL: http://dx.doi.org/10.1063/1.330526.

[18] Carmine D’Agostino et al. “Interpretation of NMR Relaxation as a Tool for Characterising the Adsorption Strength of Liquids inside Porous Materials”. In: *Chemistry – A European Journal* 20.40 (2014), 13009–13015. ISSN: 1521-3765. DOI: 10.1002/chem.201403139. URL: http://dx.doi.org/10.1002/chem.201403139.

[19] Kenneth S. Mendelson. “Nuclear Magnetic Relaxation in Porous Media”. In: *Journal of The Electrochemical Society* 133.3 (1986), 631–633. ISSN: 1945-7111. DOI: 10.1149/1.2108633. URL: http://dx.doi.org/10.1149/1.2108633.

[20] A. Timur. “Pulsed Nuclear Magnetic Resonance Studies of Porosity, Movable Fluid, and Permeability of Sandstones”. In: *Journal of Petroleum Technology* 21.06 (1969), 775–786. ISSN: 1944-978X. DOI: 10.2118/2045-pa. URL: http://dx.doi.org/10.2118/2045-pa.

[21] James J. Howard, William E. Kenyon, and Chris Straley. “Proton Magnetic Resonance and Pore Size Variations in Reservoir Sandstones”. In: *SPE Formation Evaluation* 8.03 (1993), 194–200. ISSN: 2469-8512. DOI: 10.2118/20600-pa. URL: http://dx.doi.org/10.2118/20600-pa.

[22] S. Davies and K. J. Packer. “Pore-size distributions from nuclear magnetic resonance spin-lattice relaxation measurements of fluid-saturated porous solids. I. Theory and simulation”. In: *Journal of Applied Physics* 67.6 (1990), 3163–3170. ISSN: 1089-7550. DOI: 10.1063/1.345395. URL: http://dx.doi.org/10.1063/1.345395.

[23] N. Bloembergen, E. M. Purcell, and R. V. Pound. "Relaxation Effects in Nuclear Magnetic Resonance Absorption". In: *Physical Review* 73.7 (1948), 679–712. ISSN: 0031-899X. DOI: 10.1103/physrev.73.679. URL: http://dx.doi.org/10.1103/physrev.73.679.

[24] H. A. Resing and H. C. Torrey. "Nuclear Spin Relaxation by Translational Diffusion. III. Spin-Spin Relaxation". In: *Physical Review* 131.3 (1963), 1102–1104. ISSN: 0031-899X. DOI: 10.1103/physrev.131.1102. URL: http://dx.doi.org/10.1103/physrev.131.1102.

[25] B. Kanchibotla et al. "Transverse spin relaxation time in organic molecules". In: *Physical Review B* 78.19 (2008). ISSN: 1550-235X. DOI: 10.1103/physrevb.78.193306. URL: http://dx.doi.org/10.1103/physrevb.78.193306.

[26] Saurabh Tandon and Zoya Heidari. "Effect of Internal Magnetic-Field Gradients on Nuclear-Magnetic-Resonance Measurements and Nuclear-Magnetic-Resonance-Based Pore-Network Characterization". In: *SPE Reservoir Evaluation amp; Engineering* 21.03 (2018), 609–625. ISSN: 1930-0212. DOI: 10.2118/181532-pa. URL: http://dx.doi.org/10.2118/181532-pa.

[27] Stian Almenningen et al. "Effect of Mineral Composition on Transverse Relaxation Time Distributions and MR Imaging of Tight Rocks from Offshore Ireland". In: *Minerals* 10.3 (2020), p. 232. ISSN: 2075-163X. DOI: 10.3390/min10030232. URL: http://dx.doi.org/10.3390/min10030232.

[28] Paul R.J. Connolly et al. "Simulation and experimental measurements of internal magnetic field gradients and NMR transverse relaxation times (T2) in sandstone rocks". In: *Journal of Petroleum Science and Engineering* 175 (2019), 985–997. ISSN: 0920-4105. DOI: 10.1016/j.petrol.2019.01.036. URL: http://dx.doi.org/10.1016/j.petrol.2019.01.036.

[29] Hugh Daigle, Andrew Johnson, and Brittney Thomas. "Determining fractal dimension from nuclear magnetic resonance data in rocks with internal magnetic field gradients". In: *GEOPHYSICS* 79.6 (2014), D425–D431. ISSN: 1942-2156. DOI: 10.1190/geo2014-0325.1. URL: http://dx.doi.org/10.1190/geo2014-0325.1.

[30] J.-P. Korb, Shu Xu, and J. Jonas. "Confinement effects on dipolar relaxation by translational dynamics of liquids in porous silica glasses". In: *The Journal of Chemical Physics* 98.3 (1993), 2411–2422. ISSN: 1089-7690. DOI: 10.1063/1.464169. URL: http://dx.doi.org/10.1063/1.464169.

[31] J.D. Loren and J.D. Robinson. "Relations Between Pore Size Fluid and Matrix Properties, and NML Measurements". In: *Society of Petroleum Engineers Journal* 10.03 (1970), 268–278. ISSN: 0197-7520. DOI: 10.2118/2529-pa. URL: http://dx.doi.org/10.2118/2529-pa.

[32] Yang Chen et al. "Experimental study on the rheological characteristics and viscosity-enhanced factors of super-viscous heavy oil". In: *Liquid and Gaseous Energy Resources* 3.2 (2023), 67–75. ISSN: 2669-2791. DOI: 10.21595/lger.2023.23660. URL: http://dx.doi.org/10.21595/lger.2023.23660.

[33] K. Allsopp et al. "Determination of Oil and Water Compositions of Oil/Water Emulsions Using Low Field NMR Relaxometry". In: *Journal of Canadian Petroleum Technology* 40.07 (2001). ISSN: 0021-9487. DOI: 10.2118/01-07-05. URL: http://dx.doi.org/10.2118/01-07-05.

[34] Kathryn E. Washburn. "Relaxation mechanisms and shales". In: *Concepts in Magnetic Resonance Part A* 43A.3 (2014), 57–78. ISSN: 1552-5023. DOI: 10.1002/cmr.a.21302. URL: http://dx.doi.org/10.1002/cmr.a.21302.

[35] R. Freedman et al. "Wettability, Saturation, and Viscosity From NMR Measurements". In: *SPE Journal* 8.04 (2003), 317–327. ISSN: 1930-0220. DOI: 10.2118/87340-pa. URL: http://dx.doi.org/10.2118/87340-pa.

[36] Saurabh Tandon and Zoya Heidari. "Improved Analysis of Nuclear-Magnetic-Resonance Measurements in Organic-Rich Mudrocks Through Experimental Quantification of the Hydrocarbon/Kerogen Intermolecular-Interfacial-Relaxation Mechanism". In: *SPE Journal* 25.05 (2020), 2547–2563. ISSN: 1930-0220. DOI: 10.2118/202480-pa. URL: http://dx.doi.org/10.2118/202480-pa.

[37] Kristina Keating and Rosemary Knight. "The effect of spatial variation in surface relaxivity on nuclear magnetic resonance relaxation rates". In: *GEOPHYSICS* 77.5 (2012), E365–E377. ISSN: 1942-2156. DOI: 10.1190/geo2011-0462.1. URL: http://dx.doi.org/10.1190/geo2011-0462.1.

[38] Peiqiang Zhao et al. "Nuclear magnetic resonance surface relaxivity and its advanced application in calculating pore size distributions". In: *Marine and Petroleum Geology* 111 (2020), 66–74. ISSN: 0264-8172. DOI: 10.1016/j.marpetgeo.2019.08.002. URL: http://dx.doi.org/10.1016/j.marpetgeo.2019.08.002.

[39] Elliot Grunewald and Rosemary Knight. "The effect of pore size and magnetic susceptibility on the surface NMR relaxation parameter". In: *Near Surface Geophysics* 9.2 (2010), 169–178. ISSN: 1873-0604. DOI: 10.3997/1873-0604.2010062. URL: http://dx.doi.org/10.3997/1873-0604.2010062.

[40] Z. R. Hinedi et al. "Quantification of microporosity by nuclear magnetic resonance relaxation of water imbibed in porous media". In: *Water Resources Research* 33.12 (1997), 2697–2704. ISSN: 1944-7973. DOI: 10.1029/97wr02408. URL: http://dx.doi.org/10.1029/97wr02408.

[41] John Doveton and Lynn Watney. "Textural and pore size analysis of carbonates from integrated core and nuclear magnetic resonance logging: An Arbuckle study". In: *Interpretation* 3.1 (2015), SA77–SA89. ISSN: 2324-8866. DOI: 10.1190/int-2014-0050.1. URL: http://dx.doi.org/10.1190/int-2014-0050.1.

[42] Heng Wang et al. "Low-Field Nuclear Magnetic Resonance Characterization of Carbonate and Sandstone Reservoirs From Rock Spring Uplift of Wyoming". In: *Journal of Geophysical Research: Solid Earth* 123.9 (2018), 7444–7460. ISSN: 2169-9356. DOI: 10.1029/2018jb015779. URL: http://dx.doi.org/10.1029/2018jb015779.

[43] Carmine D'Agostino et al. "Effect of paramagnetic species on T1, T2 and T1/T2 NMR relaxation times of liquids in porous CuSO4/Al2O3". In: *RSC Advances* 7.57 (2017), 36163–36167. ISSN: 2046-2069. DOI: 10.1039/c7ra07165e. URL: http://dx.doi.org/10.1039/c7ra07165e.

[44] Kristina Keating and Rosemary Knight. "A laboratory study to determine the effect of iron oxides on proton NMR measurements". In: *GEOPHYSICS* 72.1 (2007), E27–E32. ISSN: 1942-2156. DOI: 10.1190/1.2399445. URL: http://dx.doi.org/10.1190/1.2399445.

[45] I. Foley, S.A. Farooqui, and R.L. Kleinberg. "Effect of Paramagnetic Ions on NMR Relaxation of Fluids at Solid Surfaces". In: *Journal of Magnetic Resonance, Series A* 123.1 (1996), 95–104. ISSN: 1064-1858. DOI: 10.1006/jmra.1996.0218. URL: http://dx.doi.org/10.1006/jmra.1996.0218.

[46] Vivek Anand and George J. Hirasaki. "Paramagnetic relaxation in sandstones: Distinguishing T1 and T2 dependence on surface relaxation, internal gradients and dependence on echo spacing". In: *Journal of Magnetic Resonance* 190.1 (2008), 68–85. ISSN: 1090-7807. DOI: 10.1016/j.jmr.2007.09.019. URL: http://dx.doi.org/10.1016/j.jmr.2007.09.019.

[47] Paul R. J. Connolly et al. "Capillary trapping quantification in sandstones using ¡scp¿NMR¡/scp¿ relaxometry". In: *Water Resources Research* 53.9 (2017), 7917–7932. ISSN: 1944-7973. DOI: 10.1002/2017wr020829. URL: http://dx.doi.org/10.1002/2017wr020829.

[48] E. O. Stejskal and J. E. Tanner. "Spin Diffusion Measurements: Spin Echoes in the Presence of a Time-Dependent Field Gradient". In: *The Journal of Chemical Physics* 42.1 (1965), 288–292. ISSN: 1089-7690. DOI: 10.1063/1.1695690. URL: http://dx.doi.org/10.1063/1.1695690.

[49] J. Mitchell et al. "Nuclear magnetic resonance relaxation and diffusion in the presence of internal gradients: The effect of magnetic field strength". In: *Physical Review E* 81.2 (2010). ISSN: 1550-2376. DOI: 10.1103/physreve.81.026101. URL: http://dx.doi.org/10.1103/physreve.81.026101.

[50] E. Toumelin, C. Torres-Verdin, and S. Chen. "Quantification of Multi-Phase Fluid Saturations in Complex Pore Geometries from Simulations of Nuclear Magnetic Resonance Measurements". In: *SPE Annual Technical Conference and Exhibition*. 02ATCE. SPE, 2002. DOI: 10.2118/77399-ms. URL: http://dx.doi.org/10.2118/77399-ms.

[51] R.L. Kleinberg, S.A. Farooqui, and M.A. Horsfield. “T1/T2 Ratio and Frequency Dependence of NMR Relaxation in Porous Sedimentary Rocks”. In: *Journal of Colloid and Interface Science* 158.1 (1993), 195–198. ISSN: 0021-9797. DOI: 10.1006/jcis.1993.1247. URL: http://dx.doi.org/10.1006/jcis.1993.1247.

[52] R.J.S. Brown and B.W. Gamson. “Nuclear Magnetism Logging”. In: *Transactions of the AIME* 219.01 (1960), 201–209. ISSN: 0081-1696. DOI: 10.2118/1305-g. URL: http://dx.doi.org/10.2118/1305-g.

[53] Erwin L. Hahn. “Spin Echoes”. In: *Physics Today* 3.12 (1950), 21–21. ISSN: 1945-0699. DOI: 10.1063/1.3066708. URL: http://dx.doi.org/10.1063/1.3066708.

[54] Arnold L. Bloom. “Nuclear Induction in Inhomogeneous Fields”. In: *Physical Review* 98.4 (1955), 1105–1111. ISSN: 0031-899X. DOI: 10.1103/physrev.98.1105. URL: http://dx.doi.org/10.1103/physrev.98.1105.

[55] Rosemary Knight et al. “Field experiment provides ground truth for surface nuclear magnetic resonance measurement”. In: *Geophysical Research Letters* 39.3 (2012). ISSN: 1944-8007. DOI: 10.1029/2011gl050167. URL: http://dx.doi.org/10.1029/2011gl050167.

[56] W. E. Kenyon et al. “A Three-Part Study of NMR Longitudinal Relaxation Properties of Water-Saturated Sandstones”. In: *SPE Formation Evaluation* 3.03 (1988), 622–636. ISSN: 2469-8512. DOI: 10.2118/15643-pa. URL: http://dx.doi.org/10.2118/15643-pa.

[57] A. E. English et al. “Quantitative Two-Dimensional time Correlation Relaxometry”. In: *Magnetic Resonance in Medicine* 22.2 (1991), 425–434. ISSN: 1522-2594. DOI: 10.1002/mrm.1910220250. URL: http://dx.doi.org/10.1002/mrm.1910220250.

[58] S. Meiboom and D. Gill. “Modified Spin-Echo Method for Measuring Nuclear Relaxation Times”. In: *Review of Scientific Instruments* 29.8 (1958), 688–691. ISSN: 1089-7623. DOI: 10.1063/1.1716296. URL: http://dx.doi.org/10.1063/1.1716296.

[59] Rieko Ishima. “CPMG Relaxation Dispersion”. In: *Protein Dynamics*. Humana Press, 2013, 29–49. ISBN: 9781627036580. DOI: 10.1007/978-1-62703-658-0_2. URL: http://dx.doi.org/10.1007/978-1-62703-658-0_2.

[60] Martin Blanz et al. "Nuclear Magnetic Resonance Logging While Drilling (NMR-LWD): from an experiment to a day-to-day Service for the oil industry". In: *Diffusion Fundamentals* 14 (2010). ISSN: 1862-4138. DOI: 10.62721/diffusion-fundamentals.14.412. URL: http://dx.doi.org/10.62721/diffusion-fundamentals.14.412.

[61] S. O. Chan. "Detailed echo-shape fitting in NMR spin-echo experiments". In: *The Journal of Chemical Physics* 62.6 (1975), 2031–2038. ISSN: 1089-7690. DOI: 10.1063/1.430791. URL: http://dx.doi.org/10.1063/1.430791.

[62] Guangzhi Liao, Lizhi Xiao, and Ranhong Xie. "Method and experimental study of 2-D NMR logging". In: *Diffusion Fundamentals* 10 (2009). ISSN: 1862-4138. DOI: 10.62721/diffusion-fundamentals.10.448. URL: http://dx.doi.org/10.62721/diffusion-fundamentals.10.448.

[63] David Linton Johnson and Lawrence M. Schwartz. "Analytic theory of two-dimensional NMR in systems with coupled macro- and micropores". In: *Physical Review E* 90.3 (2014). ISSN: 1550-2376. DOI: 10.1103/physreve.90.032407. URL: http://dx.doi.org/10.1103/physreve.90.032407.

[64] Y.-Q. Song et al. "T1–T2 Correlation Spectra Obtained Using a Fast Two-Dimensional Laplace Inversion". In: *Journal of Magnetic Resonance* 154.2 (2002), 261–268. ISSN: 1090-7807. DOI: 10.1006/jmre.2001.2474. URL: http://dx.doi.org/10.1006/jmre.2001.2474.

[65] Marc Fleury and Maria Romero-Sarmiento. "Characterization of shales using T1–T2 NMR maps". In: *Journal of Petroleum Science and Engineering* 137 (2016), 55–62. ISSN: 0920-4105. DOI: 10.1016/j.petrol.2015.11.006. URL: http://dx.doi.org/10.1016/j.petrol.2015.11.006.

[66] Zulkuf Azizoglu et al. "Simultaneous Assessment of Wettability and Water Saturation Through Integration of 2D NMR and Electrical Resistivity Measurements". In: *SPE Annual Technical Conference and Exhibition*. 20ATCE. SPE, 2020. DOI: 10.2118/201519-ms. URL: http://dx.doi.org/10.2118/201519-ms.

[67] Mahmoud Elsayed et al. “A review on the applications of nuclear magnetic resonance (NMR) in the oil and gas industry: laboratory and field-scale measurements”. In: *Journal of Petroleum Exploration and Production Technology* 12.10 (2022), 2747–2784. ISSN: 2190-0566. DOI: 10.1007/s13202-022-01476-3. URL: http://dx.doi.org/10.1007/s13202-022-01476-3.

[68] Jonathan Mitchell and Edmund J. Fordham. “Contributed Review: Nuclear magnetic resonance core analysis at 0.3 T”. In: *Review of Scientific Instruments* 85.11 (2014). ISSN: 1089-7623. DOI: 10.1063/1.4902093. URL: http://dx.doi.org/10.1063/1.4902093.

[69] Yi-Qiao Song and Ravinath Kausik. “NMR application in unconventional shale reservoirs – A new porous media research frontier”. In: *Progress in Nuclear Magnetic Resonance Spectroscopy* 112–113 (2019), 17–33. ISSN: 0079-6565. DOI: 10.1016/j.pnmrs.2019.03.002. URL: http://dx.doi.org/10.1016/j.pnmrs.2019.03.002.

[70] A. Timur. “Pulsed Nuclear Magnetic Resonance Studies of Porosity, Movable Fluid, and Permeability of Sandstones”. In: *Journal of Petroleum Technology* 21.06 (1969), 775–786. ISSN: 1944-978X. DOI: 10.2118/2045-pa. URL: http://dx.doi.org/10.2118/2045-pa.

[71] Hiba M. Al-Janaee and Muwafaq F. Al-shahwan. “Estimation of Porosity and Permeability by using Conventional Logs and NMR Log in Mishrif Formation/Buzurgan Oil Field”. In: *Journal of Petroleum Research and Studies* 9.3 (2019), 75–89. ISSN: 2220-5381. DOI: 10.52716/jprs.v9i3.315. URL: http://dx.doi.org/10.52716/jprs.v9i3.315.

[72] A. Mai and A. Kantzas. “Porosity Distributions in Carbonate Reservoirs Using Low-Field NMR”. In: *Journal of Canadian Petroleum Technology* 46.07 (2007). ISSN: 0021-9487. DOI: 10.2118/07-07-02. URL: http://dx.doi.org/10.2118/07-07-02.

[73] David Walsh et al. “A Small-Diameter ¡scp¿NMR¡/scp¿ Logging Tool for Groundwater Investigations”. In: *Groundwater* 51.6 (2013), 914–926. ISSN: 1745-6584. DOI: 10.1111/gwat.12024. URL: http://dx.doi.org/10.1111/gwat.12024.

[74] Richard F. Sigal. “Pore-Size Distributions for Organic-Shale-Reservoir Rocks From Nuclear-Magnetic-Resonance Spectra Combined With Adsorption Measurements”. In: *SPE Journal* 20.04 (2015), 824–830. ISSN: 1930-0220. DOI: 10.2118/174546-pa. URL: http://dx.doi.org/10.2118/174546-pa.

[75] Razieh Solatpour and Apostolos Kantzas. “Application of nuclear magnetic resonance permeability models in tight reservoirs”. In: *The Canadian Journal of Chemical Engineering* 97.5 (2019), 1191–1207. ISSN: 1939-019X. DOI: 10.1002/cjce.23354. URL: http://dx.doi.org/10.1002/cjce.23354.

[76] Elijah Odusina, Carl Sondergeld, and Chandra Rai. “An NMR Study on Shale Wettability”. In: *Canadian Unconventional Resources Conference*. 11CURC. SPE, 2011. DOI: 10.2118/147371-ms. URL: http://dx.doi.org/10.2118/147371-ms.

[77] Saurabh Tandon, Chelsea Newgord, and Zoya Heidari. “Wettability Quantification in Mixed-Wet Rocks Using a New NMR-Based Method”. In: *SPE Reservoir Evaluation amp; Engineering* 23.03 (2020), 0896–0916. ISSN: 1930-0212. DOI: 10.2118/191509-pa. URL: http://dx.doi.org/10.2118/191509-pa.

[78] Andrea Valori, Farhan Ali, and Wael Abdallah. “Downhole Wettability: The Potential of NMR”. In: *SPE EOR Conference at Oil and Gas West Asia*. 18OGWA. SPE, 2018. DOI: 10.2118/190332-ms. URL: http://dx.doi.org/10.2118/190332-ms.

[79] Tianmin Jiang et al. “Evaluating Producible Hydrocarbons and Reservoir Quality in Organic Shale Reservoirs using Nuclear Magnetic Resonance (NMR) Factor Analysis”. In: *SPE/CSUR Unconventional Resources Conference*. 15URC. SPE, 2015. DOI: 10.2118/175893-ms. URL: http://dx.doi.org/10.2118/175893-ms.

[80] A.. Tinni et al. “Nuclear-Magnetic-Resonance Response of Brine, Oil, and Methane in Organic-Rich Shales”. In: *SPE Reservoir Evaluation amp; Engineering* 18.03 (2015), 400–406. ISSN: 1930-0212. DOI: 10.2118/168971-pa. URL: http://dx.doi.org/10.2118/168971-pa.

[81] R. Freedman, N. Heaton, and M. Flaum. “Field Applications of a New Nuclear Magnetic Resonance Fluid Characterization Method”. In: *SPE Annual Technical Conference and Exhibition*. 01ATCE. SPE,

2001. DOI: 10.2118/71713-ms. URL: http://dx.doi.org/10.2118/71713-ms.

[82] Gabor G. Hursan, James S. Deering, and Francis N. Kelly. "NMR Logs Help Formation Testing and Evaluation". In: *SPE Saudi Arabia Section Annual Technical Symposium and Exhibition.* 15SATS. SPE, 2015. DOI: 10.2118/177974-ms. URL: http://dx.doi.org/10.2118/177974-ms.

[83] Mauro Torres Ribeiro et al. "Integrated Petrophysics and Geosteering Reservoir Characterization in the Initial Development Phase of a Carbonate Reservoir - Campos Basin, Offshore Brazil". In: *All Days.* 11OBRA. OTC, 2011. DOI: 10.4043/22738-ms. URL: http://dx.doi.org/10.4043/22738-ms.

[84] Hugh Daigle et al. "Nuclear magnetic resonance characterization of shallow marine sediments from the Nankai Trough, Integrated Ocean Drilling Program Expedition 333". In: *Journal of Geophysical Research: Solid Earth* 119.4 (2014), pp. 2631–2650. DOI: https://doi.org/10.1002/2013JB010784. eprint: https://agupubs.onlinelibrary.wiley.com/doi/pdf/10.1002/2013JB010784. URL: https://agupubs.onlinelibrary.wiley.com/doi/abs/10.1002/2013JB010784.

[85] Son T. Dang, C. H. Sondergeld, and C. S. Rai. "Interpretation of Nuclear-Magnetic-Resonance Response to Hydrocarbons: Application to Miscible Enhanced-Oil-Recovery Experiments in Shales". In: *SPE Reservoir Evaluation amp; Engineering* 22.01 (2018), 302–309. ISSN: 1930-0212. DOI: 10.2118/191144-pa. URL: http://dx.doi.org/10.2118/191144-pa.

[86] RanHong Xie et al. "The influence factors of NMR logging porosity in complex fluid reservoir". In: *Science in China Series D: Earth Sciences* 51.S2 (2008), 212–217. ISSN: 1862-2801. DOI: 10.1007/s11430-008-6002-0. URL: http://dx.doi.org/10.1007/s11430-008-6002-0.

[87] O. Mohnke et al. "Understanding NMR relaxometry of partially water-saturated rocks". In: (2014). DOI: 10.5194/hessd-11-12697-2014. URL: http://dx.doi.org/10.5194/hessd-11-12697-2014.

[88] Elliot Grunewald and Rosemary Knight. "A laboratory study of NMR relaxation times and pore coupling in heterogeneous media". In: *GEOPHYSICS* 74.6 (2009), E215–E221. ISSN: 1942-2156. DOI: 10.1190/1.3223712. URL: http://dx.doi.org/10.1190/1.3223712.

[89] Elliot Grunewald and Rosemary Knight. "A laboratory study of NMR relaxation times in unconsolidated heterogeneous sediments". In: *GEOPHYSICS* 76.4 (2011), G73–G83. ISSN: 1942-2156. DOI: 10.1190/1.3581094. URL: http://dx.doi.org/10.1190/1.3581094.

[90] Jonathan Mitchell et al. "A finite element approach to forward modeling of nuclear magnetic resonance measurements in coupled pore systems". In: *The Journal of Chemical Physics* 150.15 (2019). ISSN: 1089-7690. DOI: 10.1063/1.5092159. URL: http://dx.doi.org/10.1063/1.5092159.

[91] I. Hidajat et al. "Study of Vuggy Carbonates Using NMR and X-Ray CT Scanning". In: *SPE Reservoir Evaluation amp; Engineering* 7.05 (2004), 365–377. ISSN: 1930-0212. DOI: 10.2118/88995-pa. URL: http://dx.doi.org/10.2118/88995-pa.

[92] Huawei Zhao et al. "Applicability Comparison of Nuclear Magnetic Resonance and Mercury Injection Capillary Pressure in Characterisation Pore Structure of Tight Oil Reservoirs". In: *SPE Asia Pacific Unconventional Resources Conference and Exhibition*. 15URCE. SPE, 2015. DOI: 10.2118/177026-ms. URL: http://dx.doi.org/10.2118/177026-ms.

[93] FUYONG WANG, KUN YANG, and JIANCHAO CAI. "FRACTAL CHARACTERIZATION OF TIGHT OIL RESERVOIR PORE STRUCTURE USING NUCLEAR MAGNETIC RESONANCE AND MERCURY INTRUSION POROSIMETRY". In: *Fractals* 26.02 (2018), p. 1840017. ISSN: 1793-6543. DOI: 10.1142/s0218348x18400170. URL: http://dx.doi.org/10.1142/s0218348x18400170.

[94] Erik Rylander et al. "NMR T2 Distributions in the Eagle Ford Shale: Reflections on Pore Size". In: *SPE Unconventional Resources Conference-USA*. 13URC. SPE, 2013. DOI: 10.2118/164554-ms. URL: http://dx.doi.org/10.2118/164554-ms.

[95] Jibin ZHONG et al. "A decomposition method of nuclear magnetic resonance T2 spectrum for identifying fluid properties". In: *Petroleum Exploration and Development* 47.4 (2020), 740–752. ISSN: 1876-3804. DOI: 10.1016/s1876-3804(20)60089-1. URL: http://dx.doi.org/10.1016/s1876-3804(20)60089-1.

[96] Chaohui Lyu et al. "Application of NMRT2to Pore Size Distribution and Movable Fluid Distribution in Tight Sandstones". In: *Energy amp; Fuels* 32.2 (2018), 1395–1405. ISSN: 1520-5029. DOI: 10.1021/acs.energyfuels.7b03431. URL: http://dx.doi.org/10.1021/acs.energyfuels.7b03431.

[97] Ziyue Li et al. "An NMR-based clay content evaluation method for tight oil reservoirs". In: *Journal of Geophysics and Engineering* 16.1 (2019), 116–124. ISSN: 1742-2140. DOI: 10.1093/jge/gxy010. URL: http://dx.doi.org/10.1093/jge/gxy010.

[98] R.L. Kleinberg and A. Boyd. "Tapered Cutoffs for Magnetic Resonance Bound Water Volume". In: *SPE Annual Technical Conference and Exhibition*. 97SPE. SPE, 1997. DOI: 10.2118/38737-ms. URL: http://dx.doi.org/10.2118/38737-ms.

[99] Quanpei Zhang et al. "Study of pore-throat structure characteristics and fluid mobility of Chang 7 tight sandstone reservoir in Jiyuan area, Ordos Basin". In: *Open Geosciences* 15.1 (2023). ISSN: 2391-5447. DOI: 10.1515/geo-2022-0534. URL: http://dx.doi.org/10.1515/geo-2022-0534.

[100] Lu Chi, Kai Cheng, and Zoya Heidari. "Improved Assessment of Interconnected Porosity in Multiple-Porosity Rocks by Use of Nanoparticle Contrast Agents and Nuclear-Magnetic-Resonance Relaxation Measurements". In: *SPE Reservoir Evaluation amp; Engineering* 19.01 (2015), 095–107. ISSN: 1930-0212. DOI: 10.2118/170792-pa. URL: http://dx.doi.org/10.2118/170792-pa.

[101] Na Zhang et al. "Application of Multifractal Theory for Determination of Fluid Movability of Coal-Measure Sedimentary Rocks Using Nuclear Magnetic Resonance (NMR)". In: *Fractal and Fractional* 7.7 (2023), p. 503. ISSN: 2504-3110. DOI: 10.3390/fractalfract7070503. URL: http://dx.doi.org/10.3390/fractalfract7070503.

[102] H. Pape et al. "Permeability Prediction for Low Porosity Rocks by Mobile NMR". In: *Pure and Applied Geophysics* 166.5–7 (2009), 1125–1163. ISSN: 1420-9136. DOI: 10.1007/s00024-009-0481-6. URL: http://dx.doi.org/10.1007/s00024-009-0481-6.

[103] Keh-Jim Dunn, Gerald A. LaTorraca, and David J. Bergman. "Permeability relation with other petrophysical parameters for periodic porous media". In: *GEOPHYSICS* 64.2 (1999), 470–478. ISSN: 1942-2156. DOI: 10.1190/1.1444552. URL: http://dx.doi.org/10.1190/1.1444552.

[104] Katherine Dlubac, Rosemary Knight, and Kristina Keating. "A numerical study of the relationship between NMR relaxation and permeability in sands and gravels". In: *Near Surface Geophysics* 12.2 (2013), 219–230. ISSN: 1873-0604. DOI: 10.3997/1873-0604.2013042. URL: http://dx.doi.org/10.3997/1873-0604.2013042.

[105] Rosemary Knight et al. "¡scp¿NMR¡/scp¿ Logging to Estimate Hydraulic Conductivity in Unconsolidated Aquifers". In: *Groundwater* 54.1 (2015), 104–114. ISSN: 1745-6584. DOI: 10.1111/gwat.12324. URL: http://dx.doi.org/10.1111/gwat.12324.

[106] Hui Gao and Huazhou Li. "Determination of movable fluid percentage and movable fluid porosity in ultra-low permeability sandstone using nuclear magnetic resonance (NMR) technique". In: *Journal of Petroleum Science and Engineering* 133 (2015), 258–267. ISSN: 0920-4105. DOI: 10.1016/j.petrol.2015.06.017. URL: http://dx.doi.org/10.1016/j.petrol.2015.06.017.

[107] Katherine Dlubac et al. "Use of NMR logging to obtain estimates of hydraulic conductivity in the High Plains aquifer, Nebraska, USA". In: *Water Resources Research* 49.4 (2013), 1871–1886. ISSN: 1944-7973. DOI: 10.1002/wrcr.20151. URL: http://dx.doi.org/10.1002/wrcr.20151.

[108] Jing-Ya Zhang et al. "Microscopic oil occurrence in high-maturity lacustrine shales: Qingshankou Formation, Gulong Sag, Songliao Basin". In: *Petroleum Science* 20.5 (2023), 2726–2746. ISSN: 1995-8226. DOI: 10.1016/j.petsci.2023.08.026. URL: http://dx.doi.org/10.1016/j.petsci.2023.08.026.

[109] Jonathan Mitchell et al. "Quantitative Remaining Oil Interpretation Using Magnetic Resonance: From the Laboratory to the Pilot". In: *SPE EOR Conference at Oil and Gas West Asia*. 12OGWA. SPE, 2012. DOI: 10.2118/154704-ms. URL: http://dx.doi.org/10.2118/154704-ms.

[110] Jonathan Mitchell et al. "Quantitative In Situ Enhanced Oil Recovery Monitoring Using Nuclear Magnetic Resonance". In: *Transport in Porous Media* 94.3 (2012), 683–706. ISSN: 1573-1634. DOI: 10.1007/s11242-012-0019-8. URL: http://dx.doi.org/10.1007/s11242-012-0019-8.

[111] Jonathan Mitchell et al. "Monitoring Chemical EOR Processes". In: *SPE Improved Oil Recovery Symposium*. 14IOR. SPE, 2014. DOI: 10.2118/169155-ms. URL: http://dx.doi.org/10.2118/169155-ms.

[112] Ahmad M. Al Harbi et al. "The Study of Nanosurfactant EOR in Carbonates by Advanced NMR Technique". In: *Abu Dhabi International Petroleum Exhibition amp; Conference*. 17ADIP. SPE, 2017. DOI: 10.2118/188710-ms. URL: http://dx.doi.org/10.2118/188710-ms.

[113] Haitao Wang et al. "Nuclear Magnetic Resonance Study on Mechanisms of Oil Mobilization in Tight Reservoir Exposed to CO2 in Pore Scale". In: *SPE Improved Oil Recovery Conference*. 16IOR. SPE, 2016. DOI: 10.2118/179554-ms. URL: http://dx.doi.org/10.2118/179554-ms.

[114] Dao-Yi Zhu, Zhi-Hua Deng, and Si-Wei Chen. "A review of nuclear magnetic resonance (NMR) technology applied in the characterization of polymer gels for petroleum reservoir conformance control". In: *Petroleum Science* 18.6 (2021), 1760–1775. ISSN: 1995-8226. DOI: 10.1016/j.petsci.2021.09.008. URL: http://dx.doi.org/10.1016/j.petsci.2021.09.008.

[115] J. Mitchell, A.M. Howe, and A. Clarke. "Real-time oil-saturation monitoring in rock cores with low-field NMR". In: *Journal of Magnetic Resonance* 256 (2015), 34–42. ISSN: 1090-7807. DOI: 10.1016/j.jmr.2015.04.011. URL: http://dx.doi.org/10.1016/j.jmr.2015.04.011.

[116] Hyung Tae Kwak, Jinxun Wang, and Abdulkarim M. AlSofi. "Close Monitoring of Gel Based Conformance Control by NMR Techniques". In: *SPE Middle East Oil amp; Gas Show and Conference*. 17MEOS. SPE, 2017. DOI: 10.2118/183719-ms. URL: http://dx.doi.org/10.2118/183719-ms.

[117] Ping Yang, Hekun Guo, and Daoyong Yang. "Determination of Residual Oil Distribution during Waterflooding in Tight Oil Formations with NMR Relaxometry Measurements". In: *Energy amp; Fuels* 27.10 (2013), 5750–5756. ISSN: 1520-5029. DOI: 10.1021/ef400631h. URL: http://dx.doi.org/10.1021/ef400631h.

[118] Shuang Liang et al. "Study on EOR method in offshore oilfield: Combination of polymer microspheres flooding and nitrogen foam flooding". In: *Journal of Petroleum Science and Engineering* 178 (2019), 629–639. ISSN: 0920-4105. DOI: 10.1016/j.petrol.2019.03.078. URL: http://dx.doi.org/10.1016/j.petrol.2019.03.078.

[119] Yuechao Zhao et al. "CO2 flooding enhanced oil recovery evaluated using magnetic resonance imaging technique". In: *Energy* 203 (2020), p. 117878. ISSN: 0360-5442. DOI: 10.1016/j.energy.2020.117878. URL: http://dx.doi.org/10.1016/j.energy.2020.117878.

[120] Baharak B. Alamdari, Mojtaba Kiani, and Hossein Kazemi. "Experimental and Numerical Simulation of Surfactant-Assisted Oil Recovery in Tight Fractured Carbonate Reservoir Cores". In: *SPE Improved Oil Recovery Symposium*. 12IOR. SPE, 2012. DOI: 10.2118/153902-ms. URL: http://dx.doi.org/10.2118/153902-ms.

[121] Ming Li et al. "Polymer Flooding Enhanced Oil Recovery Evaluated with Magnetic Resonance Imaging and Relaxation Time Measurements". In: *Energy amp; Fuels* 31.5 (2017), 4904–4914. ISSN: 1520-5029. DOI: 10.1021/acs.energyfuels.7b00030. URL: http://dx.doi.org/10.1021/acs.energyfuels.7b00030.

[122] Bin Zou et al. "Mechanisms of Permeability Alteration via Gel Based on Nuclear Magnetic Resonance". In: *Processes* 13.2 (2025), p. 497. ISSN: 2227-9717. DOI: 10.3390/pr13020497. URL: http://dx.doi.org/10.3390/pr13020497.

[123] Xing Huang et al. “Influence of Typical Core Minerals on Tight Oil Recovery during CO2 Flooding Using the Nuclear Magnetic Resonance Technique”. In: *Energy amp; Fuels* 33.8 (2019), 7147–7154. ISSN: 1520-5029. DOI: 10.1021/acs.energyfuels.9b01220. URL: http://dx.doi.org/10.1021/acs.energyfuels.9b01220.

[124] Bo Ren et al. “Laboratory Assessment and Field Pilot of Near Miscible CO2 Injection for IOR and Storage in a Tight Oil Reservoir of ShengLi Oilfield China”. In: *SPE Enhanced Oil Recovery Conference*. 11EORC. SPE, 2011. DOI: 10.2118/144108-ms. URL: http://dx.doi.org/10.2118/144108-ms.

[125] Peng Chen et al. “Experimental Investigation on CO2 Injection in Block M”. In: *Journal of Chemistry* 2018 (2018), 1–7. ISSN: 2090-9071. DOI: 10.1155/2018/8623020. URL: http://dx.doi.org/10.1155/2018/8623020.

[126] X. Dong et al. “Investigating Impacts of Fluid Types and Pore Sizes on Oil Recovery of N2-CO2 Huff-n-Puff with NMR”. In: *82nd EAGE Annual Conference amp; Exhibition*. European Association of Geoscientists Engineers, 2020, 1–5. DOI: 10.3997/2214-4609.202010154. URL: http://dx.doi.org/10.3997/2214-4609.202010154.

[127] K. Asghari et al. “Development of a Correlation Between Performance of CO2 Flooding and the Past Performance of Waterflooding in Weyburn Oil Field”. In: *SPE/DOE Symposium on Improved Oil Recovery*. 06IOR. SPE, 2006. DOI: 10.2118/99789-ms. URL: http://dx.doi.org/10.2118/99789-ms.

[128] William S. Price. “Pulsed-field gradient nuclear magnetic resonance as a tool for studying translational diffusion: Part 1. Basic theory”. In: *Concepts in Magnetic Resonance* 9.5 (1997), 299–336. ISSN: 1099-0534. DOI: 10.1002/(sici)1099-0534(1997)9:5<299::aid-cmr2>3.0.co;2-u. URL: http://dx.doi.org/10.1002/(SICI)1099-0534(1997)9:5<299::AID-CMR2>3.0.CO;2-U.

[129] R.M Cotts et al. “Pulsed field gradient stimulated echo methods for improved NMR diffusion measurements in heterogeneous systems”. In: *Journal of Magnetic Resonance (1969)* 83.2 (1989), 252–266. ISSN: 0022-2364. DOI: 10.1016/0022-2364(89)90189-3. URL: http://dx.doi.org/10.1016/0022-2364(89)90189-3.

[130] Mahmoud Elsayed et al. “New Technique for Evaluating Fracture Geometry and Preferential Orientation Using Pulsed Field Gradient Nuclear Magnetic Resonance”. In: *SPE Journal* 26.05 (2021), 2880–2893. ISSN: 1930-0220. DOI: 10.2118/205505-pa. URL: http://dx.doi.org/10.2118/205505-pa.

[131] Gabor Hursan et al. “Oil Viscosity Estimation from NMR Logs for In-Situ Heavy Oil Characterization”. In: *SPE Annual Technical Conference and Exhibition*. 16ATCE. SPE, 2016. DOI: 10.2118/181600-ms. URL: http://dx.doi.org/10.2118/181600-ms.

[132] Bo Liu et al. “Investigation of oil and water migrations in lacustrine oil shales using 20 MHz 2D NMR relaxometry techniques”. In: *Petroleum Science* 19.3 (2022), 1007–1018. ISSN: 1995-8226. DOI: 10.1016/j.petsci.2021.10.011. URL: http://dx.doi.org/10.1016/j.petsci.2021.10.011.

[133] B.A. Baldwin and W.S. Yamanashi. “NMR imaging of fluid dynamics in reservoir core”. In: *Magnetic Resonance Imaging* 6.5 (1988), 493–500. ISSN: 0730-725X. DOI: 10.1016/0730-725x(88)90123-3. URL: http://dx.doi.org/10.1016/0730-725x(88)90123-3.

[134] J. Mitchell et al. “Magnetic resonance imaging in laboratory petrophysical core analysis”. In: *Physics Reports* 526.3 (2013), 165–225. ISSN: 0370-1573. DOI: 10.1016/j.physrep.2013.01.003. URL: http://dx.doi.org/10.1016/j.physrep.2013.01.003.

[135] M. P. Enwere and J. S. Archer. “NMR Imaging for Water/Oil Displacement in Cores Under Viscous-Capillary Force Control”. In: *SPE/DOE Enhanced Oil Recovery Symposium*. 92EOR. SPE, 1992. DOI: 10.2118/24166-ms. URL: http://dx.doi.org/10.2118/24166-MS.

[136] B. Afsahi and A. Kantzas. “Advances in Diffusivity Measurement of Solvents in Oil Sands”. In: *Canadian International Petroleum Conference*. 06CIPC. PETSOC, 2006. DOI: 10.2118/2006-130. URL: http://dx.doi.org/10.2118/2006-130.

[137] Mohamed Mehana and Ilham El-monier. “Shale characteristics impact on Nuclear Magnetic Resonance (NMR) fluid typing methods and correlations”. In: *Petroleum* 2.2 (2016), 138–147. ISSN: 2405-6561. DOI: 10.1016/j.petlm.2016.02.002. URL: http://dx.doi.org/10.1016/j.petlm.2016.02.002.

[138] I. Habina et al. "Insight into oil and gas-shales compounds signatures in low field 1H NMR and its application in porosity evaluation". In: *Microporous and Mesoporous Materials* 252 (2017), 37–49. ISSN: 1387-1811. DOI: 10.1016/j.micromeso.2017.05.054. URL: http://dx.doi.org/10.1016/j.micromeso.2017.05.054.

[139] Sun Hao et al. "Understanding Shale Gas Production Mechanisms Through Reservoir Simulation". In: *SPE/EAGE European Unconventional Resources Conference and Exhibition*. 14UNCV. SPE, 2014. DOI: 10.2118/167753-ms. URL: http://dx.doi.org/10.2118/167753-ms.

[140] Utpalendu Kuila and Manika Prasad. "Specific surface area and pore-size distribution in clays and shales". In: *Geophysical Prospecting* 61.2 (2013), 341–362. ISSN: 1365-2478. DOI: 10.1111/1365-2478.12028. URL: http://dx.doi.org/10.1111/1365-2478.12028.

[141] Scott H. Stevens, Keith D. Moodhe, and Vello A. Kuuskraa. "China Shale Gas and Shale Oil Resource Evaluation and Technical Challenges". In: *SPE Asia Pacific Oil and Gas Conference and Exhibition*. 13APOG. SPE, 2013. DOI: 10.2118/165832-ms. URL: http://dx.doi.org/10.2118/165832-ms.

[142] NIE Haikuan, ZHANG Jinchuan, and JIANG Shengling. "Types and Characteristics of the Lower Silurian Shale Gas Reservoirs in and Around the Sichuan Basin". In: *Acta Geologica Sinica - English Edition* 89.6 (2015), 1973–1985. ISSN: 1755-6724. DOI: 10.1111/1755-6724.12611. URL: http://dx.doi.org/10.1111/1755-6724.12611.

[143] Zhanlei Wang et al. "Nanoscale Pore Characteristics of the Lower Permian Shanxi Formation Transitional Facies Shale, Eastern Ordos Basin, North China". In: *Frontiers in Earth Science* 10 (2022). ISSN: 2296-6463. DOI: 10.3389/feart.2022.842955. URL: http://dx.doi.org/10.3389/feart.2022.842955.

[144] Gareth R. Chalmers, R. Marc Bustin, and Ian M. Power. "Characterization of gas shale pore systems by porosimetry, pycnometry, surface area, and field emission scanning electron microscopy/transmission electron microscopy image analyses: Examples from the Barnett, Woodford, Haynesville, Marcellus, and Doig units". In: *AAPG Bulletin* 96.6 (2012), 1099–1119. ISSN: 0149-1423. DOI: 10.1306/10171111052. URL: http://dx.doi.org/10.1306/10171111052.

[145] Jin Lai et al. "Diagenesis and reservoir quality in tight gas sandstones: The fourth member of the Upper Triassic Xujiahe Formation, Central Sichuan Basin, Southwest China". In: *Geological Journal* 53.2 (2017). Ed. by A Ruffell, 629–646. ISSN: 1099-1034. DOI: 10.1002/gj.2917. URL: http://dx.doi.org/10.1002/gj.2917.

[146] Kaishuo Yang et al. "Shale rock core analysis using NMR: Effect of bitumen and water content". In: *Journal of Petroleum Science and Engineering* 195 (2020), p. 107847. ISSN: 0920-4105. DOI: 10.1016/j.petrol.2020.107847. URL: http://dx.doi.org/10.1016/j.petrol.2020.107847.

[147] F. Panattoni et al. "Improved Description of Organic Matter in Shales by Enhanced Solid Fraction Detection with Low-Field 1H NMR Relaxometry". In: *Energy amp; Fuels* 35.22 (2021), 18194–18209. ISSN: 1520-5029. DOI: 10.1021/acs.energyfuels.1c02386. URL: http://dx.doi.org/10.1021/acs.energyfuels.1c02386.

[148] Minglong Li et al. "Differences of Organic Matter Types of High Maturity Marine Organic-Rich Shale from Wufeng-Longmaxi Formation: Implication for Shale Gas "Sweet Spots" Prediction". In: *Energy Exploration amp; Exploitation* 41.4 (2023), 1325–1343. ISSN: 2048-4054. DOI: 10.1177/01445987231171407. URL: http://dx.doi.org/10.1177/01445987231171407.

[149] Xinhua Ma et al. "Insights into NMR response characteristics of shales and its application in shale gas reservoir evaluation". In: *Journal of Natural Gas Science and Engineering* 84 (2020), p. 103674. ISSN: 1875-5100. DOI: 10.1016/j.jngse.2020.103674. URL: http://dx.doi.org/10.1016/j.jngse.2020.103674.

[150] Seyedalireza Khatibi et al. "NMR relaxometry a new approach to detect geochemical properties of organic matter in tight shales". In: *Fuel* 235 (2019), 167–177. ISSN: 0016-2361. DOI: 10.1016/j.fuel.2018.07.100. URL: http://dx.doi.org/10.1016/j.fuel.2018.07.100.

[151] Kurt Livo, Milad Saidian, and Manika Prasad. "Effect of paramagnetic mineral content and distribution on nuclear magnetic resonance surface relaxivity in organic-rich Niobrara and Haynesville shales". In: *Fuel* 269 (2020), p. 117417. ISSN: 0016-2361. DOI: 10.1016/j.fuel.

2020.117417. URL: http://dx.doi.org/10.1016/j.fuel.2020.117417.

[152] Ismail Sulucarnain, Carl H. Sondergeld, and Chandra S. Rai. "An NMR Study of Shale Wettability and Effective Surface Relaxivity". In: *SPE Canadian Unconventional Resources Conference*. 12CURC. SPE, 2012. DOI: 10.2118/162236-ms. URL: http://dx.doi.org/10.2118/162236-ms.

[153] Jinbu Li et al. "Adsorbed and free hydrocarbons in unconventional shale reservoir: A new insight from NMR T1-T2 maps". In: *Marine and Petroleum Geology* 116 (2020), p. 104311. ISSN: 0264-8172. DOI: 10.1016/j.marpetgeo.2020.104311. URL: http://dx.doi.org/10.1016/j.marpetgeo.2020.104311.

[154] Emilia V. Silletta et al. "Organic matter detection in shale reservoirs using a novel pulse sequence for T1-T2 relaxation maps at 2 MHz". In: *Fuel* 312 (2022), p. 122863. ISSN: 0016-2361. DOI: 10.1016/j.fuel.2021.122863. URL: http://dx.doi.org/10.1016/j.fuel.2021.122863.

[155] Yonghong FU et al. "Microscopic pore-fracture configuration and gas-filled mechanism of shale reservoirs in the western Chongqing area, Sichuan Basin, China". In: *Petroleum Exploration and Development* 48.5 (2021), 1063–1076. ISSN: 1876-3804. DOI: 10.1016/s1876-3804(21)60091-5. URL: http://dx.doi.org/10.1016/s1876-3804(21)60091-5.

[156] Donghan Yang and Ravinath Kausik. "23Na and 1H NMR Relaxometry of Shale at High Magnetic Field". In: *Energy amp; Fuels* 30.6 (2016), 4509–4519. ISSN: 1520-5029. DOI: 10.1021/acs.energyfuels.6b00130. URL: http://dx.doi.org/10.1021/acs.energyfuels.6b00130.

[157] C.R.. R. Clarkson et al. "Nanopore-Structure Analysis and Permeability Predictions for a Tight Gas Siltstone Reservoir by Use of Low-Pressure Adsorption and Mercury-Intrusion Techniques". In: *SPE Reservoir Evaluation amp; Engineering* 15.06 (2012), 648–661. ISSN: 1930-0212. DOI: 10.2118/155537-pa. URL: http://dx.doi.org/10.2118/155537-pa.

[158] C.R. Clarkson et al. “Pore structure characterization of North American shale gas reservoirs using USANS/SANS, gas adsorption, and mercury intrusion”. In: *Fuel* 103 (2013), 606–616. ISSN: 0016-2361. DOI: 10.1016/j.fuel.2012.06.119. URL: http://dx.doi.org/10.1016/j.fuel.2012.06.119.

[159] Fu-Yong Wang, Kun Yang, and Yun Zai. “Multifractal characteristics of shale and tight sandstone pore structures with nitrogen adsorption and nuclear magnetic resonance”. In: *Petroleum Science* 17.5 (2020), 1209–1220. ISSN: 1995-8226. DOI: 10.1007/s12182-020-00494-2. URL: http://dx.doi.org/10.1007/s12182-020-00494-2.

[160] Artem Borysenko et al. “Experimental investigations of the wettability of clays and shales”. In: *Journal of Geophysical Research: Solid Earth* 114.B7 (2009). ISSN: 0148-0227. DOI: 10.1029/2008jb005928. URL: http://dx.doi.org/10.1029/2008jb005928.

[161] A. M. Serry, U. Herz, and L. Tagarieva. “Reservoir Characterization while Drilling; A Real Time Geosteering Answer to Maximize Well Values. A Case Study, Offshore Abu Dhabi.” In: *Abu Dhabi International Petroleum Exhibition amp; Conference*. 16ADIP. SPE, 2016. DOI: 10.2118/183092-ms. URL: http://dx.doi.org/10.2118/183092-ms.

[162] Wenjun Zhao et al. “Approaches of Combining Machine Learning with NMR-based Pore Structure Characterization for Reservoir Evaluation”. In: (2023). DOI: 10.20944/preprints202312.0444.v1. URL: http://dx.doi.org/10.20944/preprints202312.0444.v1.

[163] L. DePavia et al. “A Next-Generation Wireline NMR Logging Tool”. In: *SPE Annual Technical Conference and Exhibition*. 03ATCE. SPE, 2003. DOI: 10.2118/84482-ms. URL: http://dx.doi.org/10.2118/84482-ms.

[164] Ridvan Akkurt et al. “Collaborative Development of a Slim LWD NMR Tool: From Concept to Field Testing”. In: *SPE Saudi Arabia Section Technical Symposium*. 09TSSA. SPE, 2009. DOI: 10.2118/126041-ms. URL: http://dx.doi.org/10.2118/126041-ms.

[165] Nicholas Heaton et al. “New Generation Magnetic Resonance While Drilling”. In: *SPE Annual Technical Conference and Exhibition*. 12ATCE. SPE, 2012. DOI: 10.2118/160022-ms. URL: http://dx.doi.org/10.2118/160022-ms.

[166] Jie Wang et al. "Theoretical investigation of heterogeneous wettability in porous media using NMR". In: *Scientific Reports* 8.1 (2018). ISSN: 2045-2322. DOI: 10.1038/s41598-018-31803-w. URL: http://dx.doi.org/10.1038/s41598-018-31803-w.

[167] Klaas Nicolay et al. "Diffusion NMR spectroscopy". In: *NMR in Biomedicine* 14.2 (2001), 94–111. ISSN: 1099-1492. DOI: 10.1002/nbm.686. URL: http://dx.doi.org/10.1002/nbm.686.

[168] Clint P. Aichele et al. "Water in oil emulsion droplet size characterization using a pulsed field gradient with diffusion editing (PFG-DE) NMR technique". In: *Journal of Colloid and Interface Science* 315.2 (2007), 607–619. ISSN: 0021-9797. DOI: 10.1016/j.jcis.2007.07.057. URL: http://dx.doi.org/10.1016/j.jcis.2007.07.057.

[169] Abubakar Isah et al. "Characterization of Fluid Drainage Mechanism at Core and Pore Scales: an NMR Capillary Pressure–Based Saturation Exponent Prediction". In: *SPE Europec featured at 82nd EAGE Conference and Exhibition*. 21EURO. SPE, 2021. DOI: 10.2118/205176-ms. URL: http://dx.doi.org/10.2118/205176-ms.

[170] Debora Salomon Marques et al. "Benchmarking of Pulsed Field Gradient Nuclear Magnetic Resonance as a Demulsifier Selection Tool with Arabian Light Crude Oils". In: *SPE Production amp; Operations* 36.02 (2020), 368–374. ISSN: 1930-1863. DOI: 10.2118/203820-pa. URL: http://dx.doi.org/10.2118/203820-pa.

[171] J M Salhany et al. "High resolution 31P nuclear magnetic resonance studies of intact yeast cells." In: *Proceedings of the National Academy of Sciences* 72.12 (1975), 4966–4970. ISSN: 1091-6490. DOI: 10.1073/pnas.72.12.4966. URL: http://dx.doi.org/10.1073/pnas.72.12.4966.

[172] Gabor Hursan et al. "Study of OBM Invasion on NMR Logging - Mechanisms and Applications". In: *SPE Kingdom of Saudi Arabia Annual Technical Symposium and Exhibition*. 18SATS. SPE, 2018. DOI: 10.2118/192218-ms. URL: http://dx.doi.org/10.2118/192218-ms.

[173] Tetsuya Suekane et al. "Application of MRI in the Measurement of Two-Phase Flow of Supercritical CO2 and Water in Porous Rocks". In: *Journal of Porous Media* 12.2 (2009), 143–154. ISSN: 1091-028X.

DOI: 10.1615/jpormedia.v12.i2.40. URL: http://dx.doi.org/10.1615/jpormedia.v12.i2.40.

[174] Yuechao Zhao et al. “Visualization of CO2 and oil immiscible and miscible flow processes in porous media using NMR micro-imaging”. In: *Petroleum Science* 8.2 (2011), 183–193. ISSN: 1995-8226. DOI: 10.1007/s12182-011-0133-1. URL: http://dx.doi.org/10.1007/s12182-011-0133-1.

[175] A Legchenko et al. “Three-dimensional magnetic resonance imaging for groundwater”. In: *New Journal of Physics* 13.2 (2011), p. 025022. ISSN: 1367-2630. DOI: 10.1088/1367-2630/13/2/025022. URL: http://dx.doi.org/10.1088/1367-2630/13/2/025022.

[176] J. Bryan et al. “In Situ Viscosity of Heavy Oil: Core and Log Calibrations”. In: *Journal of Canadian Petroleum Technology* 46.11 (2007). ISSN: 0021-9487. DOI: 10.2118/07-11-04. URL: http://dx.doi.org/10.2118/07-11-04.

[177] Zhengshuai Liu et al. “Application of nuclear magnetic resonance (NMR) in coalbed methane and shale reservoirs: A review”. In: *International Journal of Coal Geology* 218 (2020), p. 103261. ISSN: 0166-5162. DOI: 10.1016/j.coal.2019.103261. URL: http://dx.doi.org/10.1016/j.coal.2019.103261.

[178] Majed F. Kanfar. “Real-Time Integrated Petrophysics: Geosteering in Challenging Geology amp; Fluid Systems”. In: *SPE Saudi Arabia section Young Professionals Technical Symposium*. 12YPTS. SPE, 2012. DOI: 10.2118/160922-ms. URL: http://dx.doi.org/10.2118/160922-ms.

[179] S. Tandon, A. Rostami, and Z. Heidari. “A New NMR-Based Method for Wettability Assessment in Mixed-Wet Rocks”. In: *SPE Annual Technical Conference and Exhibition*. 17ATCE. SPE, 2017. DOI: 10.2118/187373-ms. URL: http://dx.doi.org/10.2118/187373-ms.

[180] Chenglin Li et al. “Nuclear magnetic resonance pore radius transformation method and fluid mobility characterization of shale oil reservoirs”. In: *Geoenergy Science and Engineering* 221 (2023), p. 211403. ISSN: 2949-8910. DOI: 10.1016/j.geoen.2022.211403. URL: http://dx.doi.org/10.1016/j.geoen.2022.211403.

References

[1] Patrick Lewis, Ethan Perez, Aleksandra Piktus, Fabio Petroni, Vladimir Karpukhin, Naman Goyal, Heinrich Küttler, Mike Lewis, Wen-tau Yih, Tim Rocktäschel, et al. Retrieval-augmented generation for knowledge-intensive nlp tasks. In *Advances in Neural Information Processing Systems*, volume 33, pages 9459–9474, 2020.

[2] Jeff Johnson, Matthijs Douze, and Herve Jegou. Billion-scale similarity search with gpus. *IEEE Transactions on Big Data*, 7(3):535–547, July 2021.

[3] Stephen Robertson and Hugo Zaragoza. The probabilistic relevance framework: Bm25 and beyond. *Foundations and Trends® in Information Retrieval*, 3(4):333–389, 2009.

[4] Shengjie Liu, Jing Wu, Jingyuan Bao, Wenyi Wang, Naira Hovakimyan, and Christopher G. Healey. Towards a robust retrieval-based summarization system. *ArXiv*, abs/2403.19889, 2024.

[5] Jordi Bayarri-Planas, Ashwin Kumar Gururajan, and Dario Garcia-Gasulla. Cost-effective, high-performance open-source llms via optimized context retrieval, 2024.

[6] Katsuhiko Ariga. Chemistry of materials nanoarchitectonics for two-dimensional films: Langmuir–blodgett, layer-by-layer assembly, and newcomers. *Chemistry of Materials*, 35(14):5233–5254, July 2023.

[7] Chen Fang, Insun Yoon, Dion Hubble, Thanh-Nhan Tran, Robert Kostecki, and Gao Liu. Recent applications of langmuir–blodgett technique in battery research. *ACS Applied Materials & Interfaces*, 14(2):2431–2439, January 2022.

[8] Wenhui Gu, Qing Li, Ran Wang, Lexin Zhang, Zhiwei Liu, and Tifeng Jiao. Recent progress in the applications of langmuir–blodgett film technology. *Nanomaterials*, 14(12):1039, June 2024.

[9] J. D. Swalen. *Langmuir-Blodgett Films: Past, Present and Future*, page 41–59. Springer US, 1991.

[10] Jingwen Song, Anna Jancik-Prochazkova, Kohsaku Kawakami, and Katsuhiko Ariga. Lateral nanoarchitectonics from nano to life: ongoing challenges in interfacial chemical science. *Chemical Science*, 15(45):18715–18750, 2024.

[11] Zahra Alinia, Elmahdy Abdulhamied, Serxho Selmani, Renaud Miclette Lamarche, S. Holger Eichhorn, and Christine E. DeWolf. Amphiphilicity of tetraazaporphyrins containing four terminal carboxylic acid and four alkyl groups promotes face-on orientation in langmuir films. *Langmuir*, 40(50):26672–26684, December 2024.

[12] Andrey V. Arakcheev, Maxim A. Shcherbina, Alexander V. Naumkin, Oleg A. Raitman, Ekaterina V. Raitman, Yuriy L. Repchenko, Oleg Yu. Grafov, Alexander G. Martynov, Yulia G. Gorbunova, Sergey N. Chvalun, and Sofiya L. Selektor. X-ray induced redox-isomeric transformations of lanthanide bis-phthalocyaninates at the air-water interface. *Surfaces and Interfaces*, 56:105682, January 2025.

[13] Alain M. Bapolisi, Anne-Catherine Lehnen, Rainhard Machatschek, Gaetano Mangiapia, Eric Mark, Jean-Francois Moulin, Petra Wendler, Stephen C. L. Hall, and Matthias Hartlieb. Antimicrobial polymers at the membrane interface: Impact of macromolecular architecture. *Small*, 21(8), December 2024.

[14] Binita Devi Basel, Sutapa Deb, Tanmoy Debnath, Ranendu Kumar Nath, and Arghyadeep Bhattacharyya. From aqueous solution to langmuir–blodgett films: Tuning the excimer-coupled aggregation-induced emission behavior of pyrene-1-carboxaldehyde. *Langmuir*, 41(1):563–573, December 2024.

[15] Zahra Sadeghi Farshi, Farzaneh Bayat, Pouria Chaghamirzaei, Murtadha Jawad, and Ali Reza Amani-Ghadim. Effect of au nanoparticles' arrangement on melamine detection sensitivity in localized surface plasmon resonance sensors. *Microchemical Journal*, 208:112532, January 2025.

[16]

[17] Iwona Golonka, Izabela W. Łukasiewicz, Aleksandra Sebastiańczyk, Katarzyna E. Greber, Wiesław Sawicki, and Witold Musiał. The influence of the amphiphilic properties of peptides on the phosphatidylinositol monolayer in the presence of ascorbic acid. *International Journal of Molecular Sciences*, 25(23):12484, November 2024.

[18] Takashi Iwahashi, Doseok Kim, and Yukio Ouchi. A sum-frequency generation vibrational spectroscopy studies on buried liquid/liquid interfaces of ccl4/[cnmim][tfsa] (n = 4 and 8) hydrophobic ionic liquids. *The Journal of Chemical Physics*, 162(1), January 2025.

[19] Victoria M. Katata, Celina M. Miyazaki, Coral Salvo-Comino, Maria Luz Rodríguez-Méndez, and Priscila Alessio. Molecular imprinted lipid membranes towards the fabrication of electrochemical sensor for methylene blue. *Applied Surface Science*, 684:161887, March 2025.

[20] Cathy E. McNamee, Daisuke Usui, Yuto Yamada, Hiroaki Shigekura, and Shinpei Yamamoto. Use of nanoparticle concentration and magnetic fields to control the structures of superparamagnetic fe3o4 nanoparticle langmuir films. *Colloid and Interface Science Communications*, 64:100817, January 2025.

[21] Taizo Mori. Mechanical control of molecular machines at an air–water interface: manipulation of molecular pliers, paddles. *Science and Technology of Advanced Materials*, 25(1), April 2024.

[22] Anabadya Pal, Priyabrata Maity, Md Azaharuddin, Soumajit Chakrabarty, Susmita Nandi, Abhijit Das, Sourav Ghosh, Upasana Sett, Pathikrit Bandopadhyay, Sanchita Nandy, Joydeep Chowdhury, and Tarakdas Basu. Silver nano-colloid particles embedded on langmuir-blodgett film matrix of stearic acid serving as a sers active sensor for detecting the herbicide 'paraquat'. *Spectrochimica Acta Part A: Molecular and Biomolecular Spectroscopy*, 329:125514, March 2025.

[23] Jongdeok Park, Md. Mahbubur Rahman, Sang Jung Ahn, and Jae-Joon Lee. Phase transitions and morphology control of langmuir-blodgett (lb) films of graphene oxide. *Journal of Colloid and Interface Science*, 684:215–224, April 2025.

[24] Thi Thao Vu, Tri Duc Luong, Duc Cuong Nguyen, Vu-Toan Le, Thi Thu Thuy Bui, Duc Dong Tran, Thi Hoa Hoang, Duy Minh Vu, and Dmitry B. Berezin. Evaluation of research progress, trends, and applications of langmuir-blodgett films of fatty acids. *ChemChemTech*, 68(2):6–45, December 2024.

[25] Ci-Neng Ye, Yu-Luo Shen, Guo-Ping Chen, Wei-Zhi Wang, and Dong-Jin Qian. Hydrogen bond driven assembly for langmuir-blodgett films of multiporphyrin-sensitized carbon nitrides as both photosensitizers and catalysts for hydrogen evolution. *International Journal of Hydrogen Energy*, 98:1119–1130, January 2025.

[26] Olga Zamyshlyayeva, Darina Malygina, Dmitry Orekhov, Oleg Kazantsev, Polina Podkopaeva, Tatiana Mishchenko, Maria Vedunova, Maxim Batenkin, and Nina Melnikova. The surface behavior of mixed monolayers and lb films of betulin-containing polymers with lipids at the air-ce3+ aqueous solution interface and on solid substrate. *Polymer Bulletin*, 82(6):1799–1823, December 2024.

[27] Patrycja Dynarowicz-Latka, Anantharaman Dhanabalan, and Osvaldo N. Oliveira. Modern physicochemical research on langmuir monolayers. *Advances in Colloid and Interface Science*, 91(2):221–293, May 2001.

[28] Robert M. Metzger, Bo Chen, Ulf Höpfner, M. V. Lakshmikantham, Dominique Vuillaume, Tsuyoshi Kawai, Xiangli Wu, Hiroaki Tachibana, Terry V. Hughes, Hiromi Sakurai, Jeffrey W. Baldwin, Christina Hosch, Michael P. Cava, Ludwig Brehmer, and Geoffrey J. Ashwell. Unimolecular electrical rectification in hexadecylquinolinium tricyanoquinodimethanide. *Journal of the American Chemical Society*, 119(43):10455–10466, October 1997.

[29] StÃ©phane Reculusa and Serge Ravaine. Synthesis of colloidal crystals of controllable thickness through the langmuirâˆ’blodgett technique. *Chemistry of Materials*, 15(2):598â€“605, January 2003.

[30] I.R. Girling, N.A. Cade, P.V. Kolinsky, J.D. Earls, G.H. Cross, and I.R. Peterson. Observation of second-harmonic generation form langmuir-blodgett multilayers of a hemicyanine dye. *Thin Solid Films*, 132(1–4):101–112, October 1985.

[31] Daniel K. Schwartz. Langmuir-blodgett film structure. *Surface Science Reports*, 27(7–8):245–334, January 1997.

[32] Katharine B. Blodgett. Monomolecular films of fatty acids on glass. *Journal of the American Chemical Society*, 56(2):495–495, February 1934.

[33] Dongmok Whang, Song Jin, Yue Wu, and Charles M. Lieber. Large-scale hierarchical organization of nanowire arrays for integrated nanosystems. *Nano Letters*, 3(9):1255–1259, August 2003.

[34] K. Kjaer, J. Als-Nielsen, C. A. Helm, P. Tippman-Krayer, and H. Moehwald. Synchrotron x-ray diffraction and reflection studies of arachidic acid monolayers at the air-water interface. *The Journal of Physical Chemistry*, 93(8):3200–3206, April 1989.

[35] Kristian Kjaer. Some simple ideas on x-ray reflection and grazing-incidence diffraction from thin surfactant films. *Physica B: Condensed Matter*, 198(1–3):100–109, April 1994.

[36] Xiaolin Li, Guangyu Zhang, Xuedong Bai, Xiaoming Sun, Xinran Wang, Enge Wang, and Hongjie Dai. Highly conducting graphene sheets and langmuir–blodgett films. *Nature Nanotechnology*, 3(9):538–542, August 2008.

[37] Yaw S. Obeng and Allen J. Bard. Langmuir films of c60 at the air-water interface. *Journal of the American Chemical Society*, 113(16):6279–6280, July 1991.

[38] Fumiko Kimura, Junzo Umemura, and Tohru Takenaka. Ftir-atr studies on langmuir-blodgett films of stearic acid with 1-9 monolayers. *Langmuir*, 2(1):96–101, January 1986.

[39] M.K. Sanyal. *Molecular Monolayers and Thin Films*, page 14–20. Elsevier, 2005.

[40] James R. Heath, Charles M. Knobler, and Daniel V. Leff. Pressure/temperature phase diagrams and superlattices of organically functionalized metal nanocrystal monolayers: the influence of particle size, size distribution, and surface passivant. *The Journal of Physical Chemistry B*, 101(2):189–197, January 1997.

[41] R. M. Kenn, C. Boehm, A. M. Bibo, I. R. Peterson, H. Moehwald, J. Als-Nielsen, and K. Kjaer. Mesophases and crystalline phases in fatty acid monolayers. *The Journal of Physical Chemistry*, 95(5):2092–2097, March 1991.

[42] Yoshiaki Yasuda, Yoshiki Hirata, Hiroaki Sugino, Masami Kumei, Masayuki Hara, Jun Miyake, and Misamichi Fujihira. Langmuir-blodgett films of reaction centers of rhodopseudomonas viridis: photoelectric characteristics. *Thin Solid Films*, 210–211:733–735, April 1992.

[43] I.V. Turko, I.S. Yurkevich, and V.L. Chashchin. Langmuir-blodgett films of immunoglobulin g for immunosensors. *Thin Solid Films*, 205(1):113–116, October 1991.

[44] Junzo. Umemura, Toshihide. Kamata, Takeshi. Kawai, and Tohru. Takenaka. Quantitative evaluation of molecular orientation in thin langmuir-blodgett films by ft-ir transmission and reflection-absorption spectroscopy. *The Journal of Physical Chemistry*, 94(1):62–67, January 1990.

[45] Michel Vandevyver, André Barraud, Raudel-Teixier, Philippe Maillard, and Charles Gianotti. Structure of porphyrin multilayers obtained by the langmuir blodgett technique. *Journal of Colloid and Interface Science*, 85(2):571–585, February 1982.

[46] S. Baker, M.C. Petty, G.G. Roberts, and M.V. Twigg. The preparation and properties of stable metal-free phthalocyanine langmuir-blodgett films. *Thin Solid Films*, 99(1–3):53–59, January 1983.

[47] Andrea Tao, Franklin Kim, Christian Hess, Joshua Goldberger, Rongrui He, Yugang Sun, Younan Xia, and Peidong Yang. Langmuirâˆ’blodgett silver nanowire monolayers for molecular sensing using surface-enhanced raman spectroscopy. *Nano Letters*, 3(9):1229â€“1233, August 2003.

[48] GÜNTER LIESER, BERND TIEKE, and GERHARD WEGNER. *STRUCTURE, PHASE TRANSITIONS AND POLYMERIZABILITY OF MULTILAYERS OF SOME DIACETYLENE MONOCARBOXYLIC ACIDS*, page 77–90. Elsevier, 1980.

[49] Tsutomu Miyasaka and Koichi Koyama. Rectified photocurrents from purple membrane langmuir-blodgett films at the electrode-electrolyte interface. *Thin Solid Films*, 210–211:146–149, April 1992.

[50] Laura J. Cote, Franklin Kim, and Jiaxing Huang. Langmuiràíblodgett assembly of graphite oxide single layers. *Journal of the American Chemical Society*, 131(3):1043Äì1049, October 2008.

[51] Marjo Ikonen, Jouko Peltonen, Elina Vuorimaa, and Helge Lemmetyinen. Study of photocycle and spectral properties of bacteriorhodopsin in langmuir-blodgett films. *Thin Solid Films*, 213(2):277–284, June 1992.

[52] Yoshiaki Yasuda, Hiroaki Sugino, Hideki Toyotama, Yoshiki Hirata, Masayuki Hara, and Jun Miyake. Control of protein orientation in molecular photoelectric devices using langmuir—blodgett films of photosynthetic reaction centers from rhodopseudomonas viridis. *Bioelectrochemistry and Bioenergetics*, 34(2):135–139, September 1994.

[53] Eugenia Pechkova and Claudio Nicolini. Accelerated protein crystal growth by protein thin film template. *Journal of Crystal Growth*, 231(4):599–602, October 2001.

[54] Guillermo Alegria and P. Leslie Dutton. I. langmuir-blodgett monolayer films of bacterial photosynthetic membranes and isolated reaction centers: preparation, spectrophotometric and electrochemical characterization. *Biochimica et Biophysica Acta (BBA) - Bioenergetics*, 1057(2):239–257, March 1991.

[55] Paolo Facci, Victor Erokhin, and Claudio Nicolini. Nanogravimetric gauge for surface density measurements and deposition analysis of langmuir-blodgett films. *Thin Solid Films*, 230(2):86–89, August 1993.

[56] Eugenia Pechkova and Claudio Nicolini. Protein nanocrystallography: a new approach to structural proteomics. *Trends in Biotechnology*, 22(3):117–122, March 2004.

[57] Claudio Nicolini, Victor Erokhin, Francesco Antolini, Paolo Catasti, and Paolo Facci. Thermal stability of protein secondary structure in langmuir-blodgett films. *Biochimica et Biophysica Acta (BBA) - General Subjects*, 1158(3):273–278, November 1993.

[58] T. Furuno, K. Takimoto, T. Kouyama, A. Ikegami, and H. Sasabe. Photovoltaic properties of purple membrane langmuir-blodgett films. *Thin Solid Films*, 160(1–2):145–151, June 1988.

[59] Arkadi L. Litvin, Suresh Valiyaveettil, David L. Kaplan, and Stephen Mann. Template-directed synthesis of aragonite under supramolecular hydrogen-bonded langmuir monolayers. *Advanced Materials*, 9(2):124–127, February 1997.

[60] Frank R Meeks. Periodic precipitation: A microcomputer analysis of transport and reaction processes in diffusion media, with software development. *Journal of Colloid and Interface Science*, 147(1):289, November 1991.

[61] Brigid R. Heywood and Stephen Mann. Template-directed inorganic crystallization: oriented nucleation of barium sulfate under langmuir monolayers of an aliphatic long chain phosphonate. *Langmuir*, 8(5):1492–1498, May 1992.

[62] E. Meir Landau, S. Grayer Wolf, M. Levanon, L. Leiserowitz, M. Lahav, and J. Sagiv. Stereochemical studies in crystal nucleation. oriented crystal growth of glycine at interfaces covered with langmuir and langmuir-blodgett films of resolved .alpha.-amino acids. *Journal of the American Chemical Society*, 111(4):1436–1445, February 1989.

[63] Brigid R. Heywood and Stephen Mann. Template-directed nucleation and growth of inorganic materials. *Advanced Materials*, 6(1):9–20, January 1994.

[64] Brigid R. Heywood and Stephen Mann. Organic template-directed inorganic crystallization: oriented nucleation of barium sulfate under compressed langmuir monolayers. *Journal of the American Chemical Society*, 114(12):4681–4686, June 1992.

[65] Brigid R. Heywood and Stephen Mann. Molecular construction of oriented inorganic materials: Controlled nucleation of calcite and aragonite under compressed langmuir monolayers. *Chemistry of Materials*, 6(3):311–318, March 1994.

[66] F. C. Meldrum, N. A. Kotov, and J. H. Fendler. Utilization of surfactant-stabilized colloidal silver nanocrystallites in the construction of mono- and multiparticulate langmuir-blodgett films. *Langmuir*, 10(7):2035–2040, July 1994.

[67] Ehud M. Landau, Moshe Levanon, Leslie Leiserowitz, Meir Lahav, and Jacob Sagiv. Transfer of structural information from langmuir monolayers to three-dimensional growing crystals. *Nature*, 318(6044):353–356, November 1985.

[68] S. Paul, C. Pearson, A. Molloy, M. A. Cousins, M. Green, S. Kolliopoulou, P. Dimitrakis, P. Normand, D. Tsoukalas, and M. C. Petty. Langmuir-blodgett film deposition of metallic nanoparticles and their application to electronic memory structures. *Nano Letters*, 3(4):533–536, March 2003.

[69] Giuseppe Liistro, Myriam Dury, Valérie-Anne Chantrain, and Gimbada B. Mwenge. Unexpected large pressure increases during inspiration with new generation cpap devices. In *Sleep and control of breathing*, page PA2002. European Respiratory Society, September 2019.

[70] Carol R. Flach, Arne Gericke, and Richard Mendelsohn. Quantitative determination of molecular chain tilt angles in monolayer films at the air/water interface: infrared reflection/absorption spectroscopy of behenic acid methyl ester. *The Journal of Physical Chemistry B*, 101(1):58–65, January 1997.

[71] Patrycja Dynarowicz-Latka and Katarzyna Hac-Wydro. Interactions between phosphatidylcholines and cholesterol in monolayers at the air/water interface. *Colloids and Surfaces B: Biointerfaces*, 37(1–2):21–25, August 2004.

[72] Gernot A. Overbeck and Dietmar Moebius. A new phase in the generalized phase diagram of monolayer films of long-chain fatty acids. *The Journal of Physical Chemistry*, 97(30):7999–8004, July 1993.

[73] Ke Gong, Si-Shen Feng, Mei Lin Go, and Pei Hsing Soew. Effects of ph on the stability and compressibility of dppc/cholesterol monolayers at the air–water interface. *Colloids and Surfaces A: Physicochemical and Engineering Aspects*, 207(1–3):113–125, July 2002.

[74] Anna M. Bibo and Ian R. Peterson. Phase diagrams of monolayers of the long chain fatty acids. *Advanced Materials*, 2(6–7):309–311, June 1990.

[75] N. A. Kotov, F. C. Meldrum, C. Wu, and J. H. Fendler. Monoparticulate layer and langmuir-blodgett-type multiparticulate layers of size-quantized cadmium sulfide clusters: A colloid-chemical approach to superlattice construction. *The Journal of Physical Chemistry*, 98(11):2735–2738, March 1994.

[76] Agnès P. Girard-Egrot, Stéphanie Godoy, and Loïc J. Blum. Enzyme association with lipidic langmuir–blodgett films: Interests and applications in nanobioscience. *Advances in Colloid and Interface Science*, 116(1–3):205–225, November 2005.

[77] P. Tippmann-Krayer, R.M. Kenn, and H. Möhwald. Thickness and temperature dependent structure of cd arachidate langmuir-blodgett films. *Thin Solid Films*, 210–211:577–582, April 1992.

[78] Guofeng Xu, Zhenan Bao, and John T. Groves. Langmuiràíblodgett films of regioregular poly(3-hexylthiophene) as field-effect transistors. *Langmuir*, 16(4):1834Äì1841, January 2000.

[79] Qingbin Zheng, Wai Hing Ip, Xiuyi Lin, Nariman Yousefi, Kan Kan Yeung, Zhigang Li, and Jang-Kyo Kim. Transparent conductive films consisting of ultralarge graphene sheets produced by langmuir–blodgett assembly. *ACS Nano*, 5(7):6039–6051, June 2011.

[80] N. R. Pallas and B. A. Pethica. Liquid-expanded to liquid-condensed transition in lipid monolayers at the air/water interface. *Langmuir*, 1(4):509–513, July 1985.

[81] Miguel Clemente-León, Christophe Mingotaud, Bóatrice Agricole, Carlos J. Gómez-Garcia, Eugenio Coronado, and Pierre Delhaès. Application of the langmuir–blodgett technique to polyoxometalates: Towards new magnetic films. *Angewandte Chemie International Edition in English*, 36(10):1114–1116, May 1997.

[82] Julio Gun and Jacob Sagiv. On the formation and structure of self-assembling monolayers. *Journal of Colloid and Interface Science*, 112(2):457–472, August 1986.

[83] Stefan Schwiegk, Thomas Vahlenkamp, Yuanze Xu, and Gerhard Wegner. Origin of orientation phenomena observed in layered langmuir-blodgett structures of hairy-rod polymers. *Macromolecules*, 25(9):2513–2525, April 1992.

[84] Christophe Ybert, Weixing Lu, Gunter Möller, and Charles M. Knobler. Collapse of a monolayer by three mechanisms. *The Journal of Physical Chemistry B*, 106(8):2004–2008, January 2002.

[85] Tokuji Miyashita. Recent studies on functional ultrathin polymer films prepared by the langmuir-blodgett technique. *Progress in Polymer Science*, 18(2):263–294, January 1993.

[86] Geoffrey J. Ashwell, Paul D. Jackson, and Wendy A. Crossland. Non-centrosymmetry and second-harmonic generation in z-type langmuir–blodgett films. *Nature*, 368(6470):438–440, March 1994.

[87] Hiroo Nakahara, Kiyoshige Fukuda, Dietmar Moebius, and Hans Kuhn. Two-dimensional arrangement of chromophores in j aggregates of long-chain merocyanines and its effect on energy transfer in monolayer systems. *The Journal of Physical Chemistry*, 90(23):6144–6148, November 1986.

[88] Thomas Sauer, Thomas Arndt, David N. Batchelder, Alexei A. Kalachev, and Gerhard Wegner. The structure of langmuir–blodgett films from substituted phthalocyaninato-polysiloxanes. *Thin Solid Films*, 187(2):357–374, June 1990.

[89] Hiroaki Tachibana, Takayoshi Nakamura, Mutsuyoshi Matsumoto, Hideo Komizu, Eiichiro Manda, Hiroyuki Niino, Akira Yabe, and Yasujiro Kawabata. Photochemical switching in conductive langmuir-blodgett films. *Journal of the American Chemical Society*, 111(8):3080–3081, April 1989.

[90] A. Cemel, Tomlinson Fort, and J. B. Lando. Polymerization of vinyl stearate multilayers. *Journal of Polymer Science Part A-1: Polymer Chemistry*, 10(7):2061–2083, July 1972.

[91] Takahiro Seki, Masako Sakuragi, Yuji Kawanishi, Takashi Tamaki, Ryoichi Fukuda, Kunihiro Ichimura, and Yasuzo Suzuki. “command surfaces” of langmuir-blodgett films. photoregulations of liquid crystal alignment by molecularly tailored surface azobenzene layers. *Langmuir*, 9(1):211–218, January 1993.

[92] Xiaolin Li, Li Zhang, Xinran Wang, Iwao Shimoyama, Xiaoming Sun, Won-Seok Seo, and Hongjie Dai. Langmuiràíblodgett assembly of densely aligned single-walled carbon nanotubes from bulk materials. *Journal of the American Chemical Society*, 129(16):4890Äì4891, March 2007.

[93] Michio Sugi and Sigeru Iizima. Anisotropic photoconduction in dye-sensitized langmuir films. *Thin Solid Films*, 68(1):199–204, May 1980.

[94] Ernst Orthmann and Gerhard Wegner. Preparation of ultrathin layers of molecularly controlled architecture from polymeric phthalocyanines by the langmuir-blodgett-technique. *Angewandte Chemie International Edition in English*, 25(12):1105–1107, December 1986.

[95] Takayoshi Nakamura, Hiroaki Tachibana, Motoo Yumura, Mutsuyoshi Matsumoto, Reiko Azumi, Motoo Tanaka, and Yasujiro Kawabata. Formation of langmuir-blodgett films of a fullerene. *Langmuir*, 8(1):4–6, January 1992.

[96] M. Fujihira, K. Nishiyama, and H. Yamada. Photoelectrochemical responses of optically transparent electrodes modified with langmuir-blodgett films consisting of surfactant derivatives of electron donor, acceptor and sensitizer molecules. *Thin Solid Films*, 132(1–4):77–82, October 1985.

[97] M. POMERANTZ and ARMIN SEGMÜLLER. *HIGH RESOLUTION X-RAY DIFFRACTION FROM SMALL NUMBERS OF LANGMUIR–BLODGETT LAYERS OF MANGANESE STEARATE*, page 33–45. Elsevier, 1980.

[98] Martin R. Bryce and Michael C. Petty. Electrically conductive langmuir–blodgett films of charge-transfer materials. *Nature*, 374(6525):771–776, April 1995.

[99] Vera Cimrová, Marcus Remmers, Dieter Neher, and Gerhard Wegner. Polarized light emission from leds prepared by the langmuir-blodgett technique. *Advanced Materials*, 8(2):146–149, February 1996.

[100] G. J. Ashwell, R. C. Hargreaves, C. E. Baldwin, G. S. Bahra, and C. R. Brown. Improved second-harmonic generation from langmuir–blodgett films of hemicyanine dyes. *Nature*, 357(6377):393–395, June 1992.

[101] P.S. Vincett and G.G. Roberts. Electrical and photoelectrical transport properties of langmuir-blodgett films and a discussion of possible device applications. *Thin Solid Films*, 68(1):135–171, May 1980.

[102] *Analytica Chimica Acta*, 300(1–3):339–340, January 1995.

[103] Alfred Blume. A comparative study of the phase transitions of phospholipid bilayers and monolayers. *Biochimica et Biophysica Acta (BBA) - Biomembranes*, 557(1):32–44, October 1979.

[104] M. Clemente-León, B. Agricole, C. Mingotaud, C. J. Gómez-García, E. Coronado, and P. Delhaes. Toward new organic/inorganic superlattices: keggin polyoxometalates in langmuir and langmuirblodgett films. *Langmuir*, 13(8):2340–2347, April 1997.

[105] Masatsugu Shimomura and Toyoki Kunitake. Preparation of langmuir-blodgett films of azobenzene amphiphiles as polyion complexes. *Thin Solid Films*, 132(1–4):243–248, October 1985.

[106] Cristina Stefaniu, Gerald Brezesinski, and Helmuth Möhwald. Langmuir monolayers as models to study processes at membrane surfaces. *Advances in Colloid and Interface Science*, 208:197–213, June 2014.

[107] G. Duda, A.J. Schouten, T. Arndt, G. Lieser, G.F. Schmidt, C. Bubeck, and G. Wegner. Preparation and characterization of monolayers and multilayers of preformed polymers. *Thin Solid Films*, 159(1–2):221–230, May 1988.

[108] E. Meyer, L. Howald, R. M. Overney, H. Heinzelmann, J. Frommer, H.-J. Güntherodt, T. Wagner, H. Schier, and S. Roth. Molecular-resolution images of langmuir–blodgett films using atomic force microscopy. *Nature*, 349(6308):398–400, January 1991.

[109] Antony S. Dimitrov and Kuniaki Nagayama. Continuous convective assembling of fine particles into two-dimensional arrays on solid surfaces. *Langmuir*, 12(5):1303–1311, January 1996.

[110] D. L. Allara and J. D. Swalen. An infrared reflection spectroscopy study of oriented cadmium arachidate monolayer films on evaporated silver. *The Journal of Physical Chemistry*, 86(14):2700–2704, July 1982.

[111] Franklin Kim, Serena Kwan, Jennifer Akana, and Peidong Yang. Langmuiràíblodgett nanorod assembly. *Journal of the American Chemical Society*, 123(18):4360Àì4361, April 2001.

[112] Andrea R. Tao, Jiaxing Huang, and Peidong Yang. Langmuirâˆ'blodgettry of nanocrystals and nanowires. *Accounts of Chemical Research*, 41(12):1662â€“1673, August 2008.

[113] Mitsuru Yoneyama, Michio Sugi, Mitsuyoshi Saito, Keiichi Ikegami, Shinichi Kuroda, and Sigeru Iizima. Photoelectric properties of copper phthalocyanine langmuir-blodgett film. *Japanese Journal of Applied Physics*, 25(7R):961, July 1986.

[114] Masa-aki Kakimoto, Masa-aki Suzuki, Toru Konishi, Yoshio Imai, Mitsumasa Iwamoto, and Taro Hino. Preparation of mono- and multilayer films of aromatic polyimides using langmuir–blodgett technique. *Chemistry Letters*, 15(5):823–826, May 1986.

[115] D. K. Schwartz, J. Garnaes, R. Viswanathan, and J. A. N. Zasadzinski. Surface order and stability of langmuir-blodgett films. *Science*, 257(5069):508–511, July 1992.

[116] C. P. Collier, R. J. Saykally, J. J. Shiang, S. E. Henrichs, and J. R. Heath. Reversible tuning of silver quantum dot monolayers through the metal-insulator transition. *Science*, 277(5334):1978–1981, September 1997.

[117] E. Sackmann. Supported membranes: Scientific and practical applications. *Science*, 271(5245):43–48, January 1996.

[118] David Andelman, Françoise Broçhard, and Jean-François Joanny. Phase transitions in langmuir monolayers of polar molecules. *The Journal of Chemical Physics*, 86(6):3673–3681, March 1987.

[119] Richard Mendelsohn, Joseph W. Brauner, and Arne Gericke. External infrared reflection absorption spectrometry of monolayer films at the air-water interface. *Annual Review of Physical Chemistry*, 46(1):305–334, October 1995.

[120] C. Naselli, J. F. Rabolt, and J. D. Swalen. Order–disorder transitions in langmuir–blodgett monolayers. i. studies of two-dimensional melting by infrared spectroscopy. *The Journal of Chemical Physics*, 82(4):2136–2140, February 1985.

[121] J. F. Rabolt, F. C. Burns, N. E. Schlotter, and J. D. Swalen. Anisotropic orientation in molecular monolayers by infrared spectroscopy. *The Journal of Chemical Physics*, 78(2):946–952, January 1983.

[122] Bernhard Mann and Hans Kuhn. Tunneling through fatty acid salt monolayers. *Journal of Applied Physics*, 42(11):4398–4405, October 1971.

[123] J. A. Zasadzinski, R. Viswanathan, L. Madsen, J. Garnaes, and D. K. Schwartz. Langmuir-blodgett films. *Science*, 263(5154):1726–1733, March 1994.

[124] G.G. Roberts. An applied science perspective of langmuir-blodgett films. *Advances in Physics*, 34(4):475–512, January 1985.

[125] *Langmuir-Blodgett Films*. Springer US, 1990.

[126] Brigid R. Heywood, Sundara Rajam, and Stephen Mann. Oriented crystallization of caco3under compressed monolayers. part 2.—morphology, structure and growth of immature crystals. *J. Chem. Soc., Faraday Trans.*, 87(5):735–743, 1991.

[127] Tsutomu Miyasaka, Koichi Koyama, and Isamu Itoh. Quantum conversion and image detection by a bacteriorhodopsin-based artificial photoreceptor. *Science*, 255(5042):342–344, January 1992.

[128] Sundara Rajam, Brigid R. Heywood, Justin B. A. Walker, Stephen Mann, Roger J. Davey, and J. Derek Birchall. Oriented crystallization of caco3under compressed monolayers. part 1.—morphological studies of mature crystals. *J. Chem. Soc., Faraday Trans.*, 87(5):727–734, 1991.

[129] Amir Berman, Dong June Ahn, Anna Lio, Miquel Salmeron, Anke Reichert, and Deborah Charych. Total alignment of calcite at acidic polydiacetylene films: Cooperativity at the organic-inorganic interface. *Science*, 269(5223):515–518, July 1995.

[130] Vladimir M. Kaganer, Helmuth Möhwald, and Pulak Dutta. Structure and phase transitions in langmuir monolayers. *Reviews of Modern Physics*, 71(3):779–819, April 1999.

[131] G. G. Roberts. Langmuir-blodgett films. *Contemporary Physics*, 25(2):109–128, March 1984.

[132] Márta Szekeres, Olexiy Kamalin, Robert A. Schoonheydt, Kurt Wostyn, Koen Clays, André Persoons, and Imre Dékány. Ordering and optical properties of monolayers and multilayers of silica spheres deposited by the langmuir–blodgett method. *J. Mater. Chem.*, 12(11):3268–3274, 2002.

[133] Jonathan V. Selinger, Zhen-Gang Wang, Robijn F. Bruinsma, and Charles M. Knobler. Chiral symmetry breaking in langmuir monolayers and smectic films. *Physical Review Letters*, 70(8):1139–1142, February 1993.

[134] Ka Yee C. Lee. Collapse mechanisms of langmuir monolayers. *Annual Review of Physical Chemistry*, 59(1):771–791, May 2008.

[135] V. M. Kaganer, I. R. Peterson, R. M. Kenn, M. C. Shih, M. Durbin, and P. Dutta. Tilted phases of fatty acid monolayers. *The Journal of Chemical Physics*, 102(23):9412–9422, June 1995.

[136] I. R. Girling, D. B. Neal, W. J. Feast, M. C. Petty, N. A. Cade, P. V. Kolinsky, G. G. Roberts, R. J. Jones, I. R. Peterson, and M. M. Ahmad. Second-harmonic generation in mixed hemicyanine: fatty-acid langmuir–blodgett monolayers. *Journal of the Optical Society of America B*, 4(6):950, June 1987.

[137] Donald Lupo, Andre Laschewsky, Isabelle Ledoux, Helmut Ringsdorf, Werner Prass, and Ude Scheunemann. Second-harmonic generation in langmuir–blodgett monolayers of stilbazium salt and phenylhydrazone dyes. *Journal of the Optical Society of America B*, 5(2):300, February 1988.

[138] P. Dutta, J. B. Peng, B. Lin, J. B. Ketterson, M. Prakash, P. Georgopoulos, and S. Ehrlich. X-ray diffraction studies of organic monolayers on the surface of water. *Physical Review Letters*, 58(21):2228–2231, May 1987.

[139] Naoto Minari, Keiichi Ikegami, Shin-ichi Kuroda, Kazuhiro Saito, Mitsuyoshi Saito, and Michio Sugi. Origin of the in-plane anisotropy in langmuir-blodgett films. *Journal of the Physical Society of Japan*, 58(1):222–231, January 1989.

[140] Deborah H. Charych, Jon O. Nagy, Wayne Spevak, and Mark D. Bednarski. Direct colorimetric detection of a receptor-ligand interaction by a polymerized bilayer assembly. *Science*, 261(5121):585–588, July 1993.

[141] Daniel Blaudez, Jean-Marie Turlet, Jean Dufourcq, Delphine Bard, Thierry Buffeteau, and Bernard Desbat. Investigations at the air/water interface using polarization modulation ir spectroscopy. *Journal of the Chemical Society, Faraday Transactions*, 92(4):525, 1996.

[142] S. Palto, L. Blinov, A. Bune, E. Dubovik, V. Fridkin, N. Petukhova, K. Verkhovskaya, and S. Yudin. Ferroelectric langmuir-blodgett films. *Ferroelectrics*, 184(1):127–129, August 1996.

[143] Stephen Ducharme, V. M. Fridkin, A. V. Bune, S. P. Palto, L. M. Blinov, N. N. Petukhova, and S. G. Yudin. Intrinsic ferroelectric coercive field. *Physical Review Letters*, 84(1):175–178, January 2000.
